# Gold(I)-Catalyzed Tandem 1,2-Indole Migration–Cyclopropanation Reactions of 3‑Propargylindoles with Olefins

**DOI:** 10.1021/acs.orglett.5c02596

**Published:** 2025-07-28

**Authors:** Lorena Renedo, Marta Solas, Raquel Hernández-Ruiz, Samuel Suárez-Pantiga, Roberto Sanz

**Affiliations:** Área de Química Orgánica, Departamento de Química, Facultad de Ciencias, 16725 Universidad de Burgos, Pza. Misael Bañuelos s/n, 09001, Burgos, Spain

## Abstract

3-Propargylindoles with a terminal alkyne, efficiently prepared from direct alkylation of indoles, undergo a tandem 1,2-indole migration/cyclopropanation reaction under gold­(I) catalysis. Reaction conditions have been developed for suitable access to indole-substituted vinylcyclopropanes from 3-propargyl indoles and olefins. The corresponding 2-alkenyl-functionalized substrates evolve through an intramolecular cyclopropanation allowing the synthesis of various polycyclic indole derivatives. The presence of silver salts modifies the diastereoselectivity observed.

## Graphical abstract

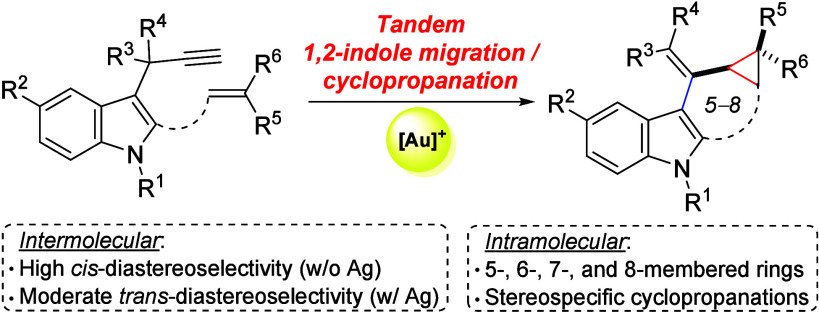

The generation and subsequent reaction of stable metal carbenes to form cyclopropanes is probably the most widely used methodology to achieve these strained, interesting building blocks.^1^ In particular, vinylcyclopropanes (VCPs) are synthetically relevant targets that are also widely employed as intermediates in organic synthesis.^2^ In this area, homogeneous Au-catalyzed cyclopropanations have been extensively developed in recent years, becoming a convenient synthetic tool that nicely complements other metal-catalyzed cyclopropanations.^3^ Typically, enynes,^4^ propargyl esters,^5^ cyclopropenes,^6^ cycloheptatrienes,^7^ alkynes,^8^ and sulfonium ylides,^9^ besides more hazardous diazo compounds,^10^ are employed as carbenoid precursors in these processes.

In this context, we have described that readily available 3-propargylindoles^11^ undergo a 1,2-indole migration in the presence of cationic gold­(I) catalysts, leading to an α,β-unsaturated gold-carbene **A**. This highly reactive intermediate evolves through different pathways depending on the substituents at the propargylic and terminal positions of the alkyne (Scheme 1).^12^ When an aromatic group is located at the propargylic position, an aura-iso-Nazarov cyclization occurs (path *a*). However, when the aromatic moiety is on the alkyne and alkyl substituents are present at the propargylic positions, **A** evolves through an aura-Nazarov cyclization (path *b*). In both cases, interesting 3-(inden-2-yl)­indoles,^13^ containing two structural domains with different biological functions,^14^ are formed. In addition, when no aromatic groups are present at any of these positions (R^2^–R^4^), an alternative 1,2-hydride migration takes place, leading to 2-indol-3-yl-1,3-butadiene derivatives (path *c*).^12b^ Furthermore, we have recently reported a tandem 1,3-indole migration/oxidation of 3-propargylindoles in the presence of external oxidants, which allows access to α-indolyl-α,β-unsaturated carbonyls (path *d*).^15^ At this point, we envisaged that highly electrophilic gold-carbene intermediates **A** could alternatively evolve through inter- and intramolecular reactions with alkenes, which would lead to cyclopropanation reactions forming interesting indole derivatives bearing cyclopropane scaffolds (Scheme 1).

**Scheme 1:**
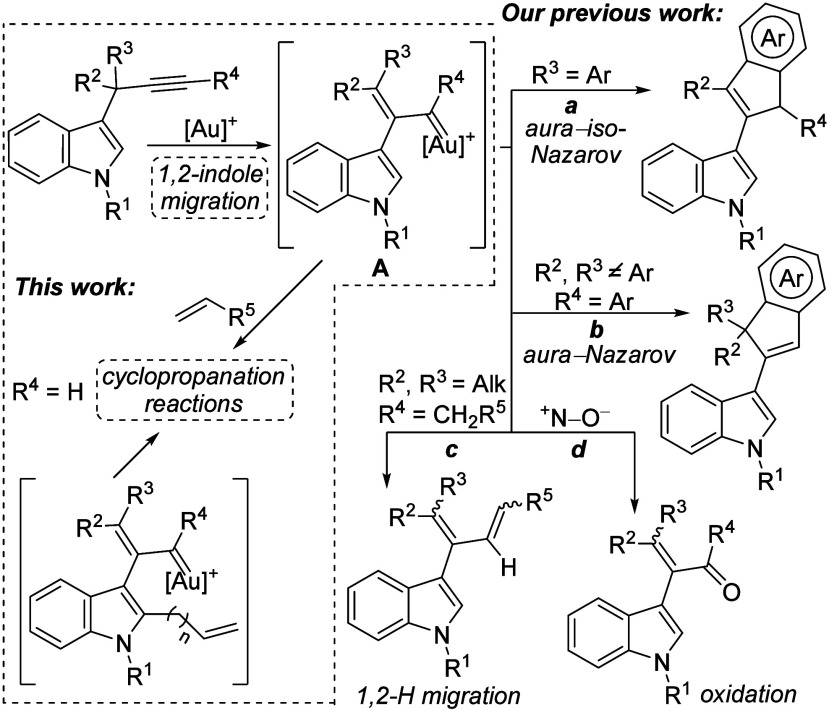
Known Reactivity of 3-Propargylindoles *Via* Gold–Carbene Intermediates and Proposed Work

3-Propargylindole **1a** and styrene as alkene were selected as model substrates and their reactivity was evaluated under catalysis with different gold­(I) complexes (Table 1). Based on our previous results,^15^ IPrAuNTf_2_ was initially selected as the catalyst and the desired tandem indole migration–cyclopropanation occurred efficiently, yielding the cyclopropane derivative **2a**, although as a ∼ 1:1 mixture of *cis*/*trans* diastereoisomers (entry 1). Seeking to improve the stereoselectivity of the reaction, other gold complexes with phosphine ligands were also investigated (entries 2–4). No remarkable improvement was observed when employing a gold catalyst bearing either MorDalPhos or JohnPhos ligands (entries 2 and 3). Gratifyingly, we found that the use of BrettPhosAuNTf_2_, bearing a bulky electron-donating ligand, gave rise to **2a** with a high yield and diastereoselectivity toward the *cis* isomer (entry 4). Next, the counteranion of the cationic gold­(I) complex, determined by the addition of a silver salt, was also investigated (entries 5–7). Surprisingly, the treatment of **1a** with the catalyst generated from BrettPhosAuCl and AgNTf_2_ resulted in a switch of the selectivity, affording **2a** in good yield but favoring the *trans* isomer (entry 5). Other silver salts led to similar or even better results regarding the diastereoselectivity (entries 6 and 7).^16^ The key role of silver in reversing selectivity was further supported by the use of NaBARF, as chloride scavenger, which resulted again in favoring *cis*-**2a** (entry 8). The presence of silver in the reaction media favors the isomerization of *cis*-**2a** to *trans*-**2a** as shown in additional experiments.^17^

**Table 1:**
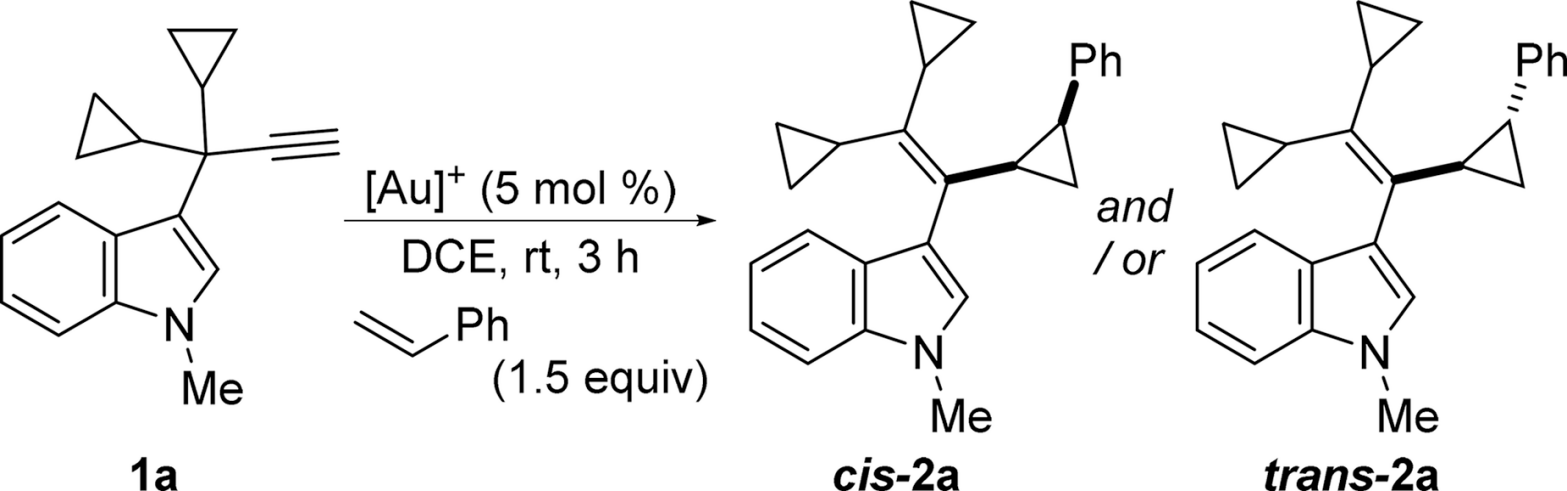
**Optimization of the Conditions for the Au-Catalyzed 1,2-Indole Migration–Cyclopropanation of 1a with Styrene** ^a^

entry	[Au]^+^	d.r. (*cis*/*trans*)^b^	yield (%)^c^
1	IPrAuNTf_2_	1/1	71
2	MorDalPhosAuNTf_2_	1/1	47
3	JohnPhosAuNTf_2_	2.5/1	63
4	BrettPhosAuNTf_2_	15/1	85
5	BrettPhosAuCl/AgNTf_2_	1/11	75
6	BrettPhosAuCl/AgOTf	1/18	50
7	BrettPhosAuCl/AgSbF_6_	1/15	82
8	BrettPhosAuCl/NaBARF	9/1	80

a Reaction conditions: **1a** (0.2 mmol), gold catalyst (5 mol%), styrene (0.3 mmol) in DCE (2 mL) at rt for 3 h. Complete conversion was observed in all cases.

b Determined by^1^H NMR analysis of the crude mixture.

c Determined by^1^H NMR analysis using 1,3,5-trimethoxybenzene as internal standard.

With an optimized set of conditions in hand, we proceeded to study the scope of the tandem process using different 3-propargyl indoles **1**, bearing a tertiary center at the propargylic position without aromatic groups and a terminal alkyne, and different olefins (Scheme 2). These indoles **1** avoid the competitive tandem process involving 1,2-indole migration followed by (iso)­Nazarov cyclizations, or 1,2-H migrations, to give 3-indenylindoles or 3-dienylindoles, respectively, which was shown to be preferred.^12,18^ First, model substrate **1a** was tested with a variety of functionalized styrenes, using BrettPhosAuNTf_2_ as catalyst, leading to the corresponding α-indol-3-yl vinylcyclopropanes **2a**–**d**, which were obtained with high *cis*-selectivity. Not unexpectedly, when α-methylstyrene was employed the indole derivative **2e** was obtained in good yield but with unappreciable stereoselectivity. In addition, symmetric 1,1-disubstituted olefins, such as methylenecyclohexane or 2-ethyl-1-butene, were also tested, providing vinylcyclopropanes **2f** and **2g** with high yields. Then, we decided to use the cationic gold catalyst generated from BrettPhosAuCl and AgSbF_6_ to obtain the corresponding *trans*-**2** vinylcyclopropane derivatives. Disappointingly, although high *trans*-selectivity was observed for styrene leading almost exclusively to the *trans*-**2a** diastereoisomer, for the other related tested styrenes, this silver effect was not so pronounced, and the corresponding VCPs **2b**–**d** were obtained with only moderate *trans* selectivity. Then, other 3-propargylindoles **1** were reacted with selected olefins. *N*H-indole **1b** proved to be a successful substrate, leading to the corresponding derivatives *cis*-**2h**–**j** with high stereoselectivity. In this case, *trans*-**2h** and *trans*-**2i** could be obtained with moderate to high stereoselectivity by carrying the reactions in the presence of silver. Replacing one or both of the cyclopropyl groups at the propargylic positions with methyl ones, as illustrated by substrates **1c**–**e**, has no significant effect on the efficiency of the reaction. As expected, the reaction of **1c** with 2-ethyl-1-butene afforded the corresponding derivative **2k** as a mixture of *E*/*Z* diastereoisomers. The reactions of styrene with indoles **1d,e**, bearing two methyl groups at the propargylic positions, required heating and provided the expected VCPs **2l,m** with slightly lower stereoselectivity compared to **1a**. Finally, functional groups, such as ester or bromine, at the C5 position of the starting indole **1f,g** were also tolerated, resulting in the corresponding cyclopropane functionalized indole derivatives **2n**–**r** with good yields and diastereoselectivities, except for α-methylstyrene (**2p**). Disappointingly, 1,2-disubstituted alkenes, such as cyclohexene and *cis*- or *trans*-stilbene, failed to react.

**Scheme 2:**
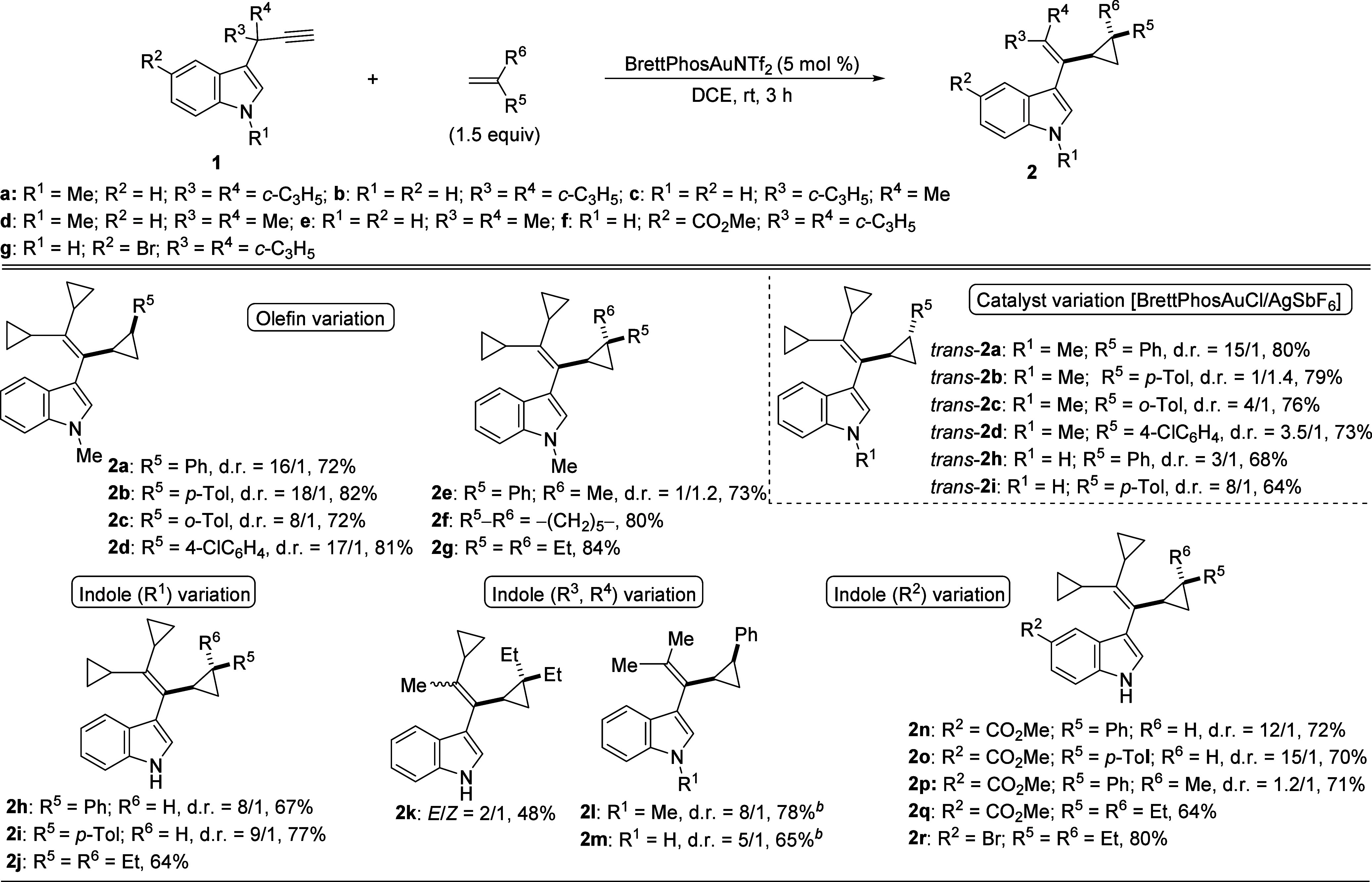
Synthesis of 3-(1-Cyclopropylvinyl)-1*H*-indole Derivatives 2^a^

a Reaction conditions: **1** (0.5 mmol), BrettPhosAuNTf_2_ (5 mol%), olefin (0.75 mmol) in DCE (2 mL) at rt for 3 h unless otherwise established. The d.r. was determined by ^1^H NMR analysis of the crude reaction mixtures. The reported yields are isolated ones after column chromatography.
b Carried out at 80 °C for 2 h.

In view of the results obtained for the reaction of terminal 3-propargylindoles **1** with different olefins, we considered expanding the scope of this transformation by using substrates bearing an alkene moiety at the C2 position. This could lead to intramolecular cyclopropanation reactions, resulting in fused cyclic compounds of variable sizes with a cyclopropane ring, depending on the length of the chain containing the alkene at C2.

Thus, we moved on to optimizing the cyclopropanation of model 3-(1,1-dicyclopropylprop-2-yn-1-yl)-1-methyl-2-styryl-1*H*-indole **3a** (Table 2). The reaction of **3a** (∼ 1:1 ratio of *E/Z* isomers) in the presence of BrettPhosAuNTf_2_ yielded tetrahydro-1*H*-cyclopropa­[4,5]­cyclopenta­[1,2-*b*]­indole **4a** as around 1:3.7 mixture of *cis*- and *trans*-diastereoisomers that could be isolated independently (entry 1). Although approximately 1:1 ratio was expected considering a stereospecific cyclopropanation, the ratio of *cis* **-4a** was much lower, likely due to its decomposition over time under the reaction conditions. Fortunately, this issue was solved by decreasing the concentration up to 0.025 M and increasing the reaction time (entry 2). Under these reoptimized conditions, it was additionally checked that the reaction of (*E*)**-3a** provided exclusively *cis* **-4a** (entry 3). Similarly, the experiment conducted with (*Z*)-**3a** led to the indole derivative **4a** with a *trans* configuration (entry 4), in both cases with good yields. To further test the scope of the process, we explored the reaction of 2-alkenyl-3-propargylindoles with different aromatic substituents at the olefin. Apart from conducting some experiments starting from the *E* isomers of **3** (entries 5, 6, 8 and 10), we also carried out reactions with substrates having the *Z-*configuration at the C=C bond (entries 7 and 9), or with a mixture of (*E*/*Z*)-3-propargylated indoles (entry 11). In view of these results, it has been demonstrated that the cyclopropanation is highly stereospecific, as the configurational information on the olefin in the starting indole is transferred to the final cyclopropane. The tandem 1,2-indole migration–intramolecular cyclopropanation worked efficiently resulting in tetrahydro-1*H*-cyclopropa­[4,5]­cyclopenta­[1,2-*b*]­indoles **4a**–**f**, which were isolated with good yields. The intramolecular cyclopropanation was compatible with substrates **3** bearing a terminal olefin and alkenes possessing aliphatic groups, allowing access to indole derivatives **4g**–**j** with good yields (entries 12–15). Furthermore, the reaction also proceeded with a β,β-disubstituted alkene-containing indole, **3k**, giving rise to the expected cycloadduct **4k** (entry 16). Finally, even a substrate containing an allylic alcohol as alkene counterpart, **3l**, yielded the corresponding indole derivative **4l**, although with a moderate yield (entry 17). Again, for related substrates **3**, bearing internal alkynes or an aryl group at the propargylic position, the competing (iso)­Nazarov cyclization or 1,2-hydride migration was favored over the intramolecular cyclopropanation.^18^

**Table 2:**
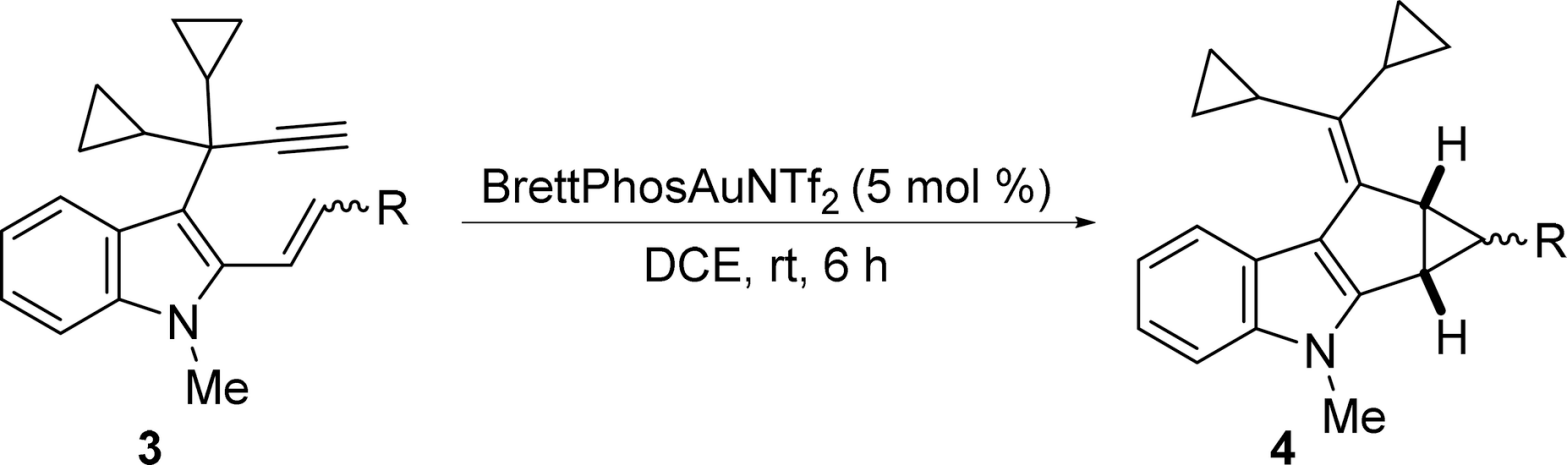
Synthesis of Tetrahydro-1*H*-cyclopropa­[4,5]­cyclopenta­[1,2-*b*]­indole Derivatives 4^a^

entry	**3**	d.r. (*E*/*Z*)	R	product	d.r.^b^ (*cis*/*trans*)	yield (%)^c^
1^d^	**3a**	1.1/1	Ph	**4a**	1/3.7	46
2	**3a**	1.1/1	Ph	**4a**	1/1.1	69
3	**3a**	1/0	Ph	*cis* **-4a**	1/0	70
4	**3a**	1/20	Ph	*trans* **-4a**	0/1	67
5^e^	**3b**	1/0	2-MeC_6_H_4_	*cis* **-4b**	1/0	64
6	**3c**	1/0	3-MeOC_6_H_4_	*cis* **-4c**	1/0	66
7	**3c**	0/1	3-MeOC_6_H_4_	*trans* **-4c**	0/1	55
8	**3d**	1/0	3,5-(MeO)_2_C_6_H_3_	*cis* **-4d**	1/0	69
9	**3d**	1/8	3,5-(MeO)_2_C_6_H_3_	*trans* **-4d**	1/7.5	70^f^
10	**3e**	1/0	3-BrC_6_H_4_	*cis* **-4e**	1/0	68
11	**3f**	1/2	3-ClC_6_H_4_	**4f**	1/1.5	70^g^
12^e^	**3g**	–	H	**4g**	–	67
13	**3h**	1.4/1	Me	**4h**	1.2/1	81
14	**3i**	1.7/1	Et	**4i**	1.8/1	61
15^h^	**3j**	1/8	*i*-Pr	*trans* **-4j**	1/10	82^f^
16^i^	**3k**	–	Me/Me	**4k**	–	67
17^j^	**3l**	1/0	CH_2_OH	*cis* **-4l**	1/0	34

a Reaction conditions: **3** (0.5 mmol), BretthosAuNTf_2_ (5 mol%), in DCE (20 mL) at rt for 6 h.

b Determined by^1^H NMR analysis of the crude mixture.

c Isolated yield after column chromatography.

d Carried out at 0.25 M for 3 h.

e Reaction time = 24 h.

f The major *trans* **-4** was isolated in pure form.

g Overall yield: *cis* **-4f** and *trans* **-4f** were isolated independently.

h Reaction time = 48 h.

i **3k** bears a β,β-dimethyl substituted alkene.

j Reaction time = 8 h.

Next, we decided to study the reactivity of 2-allyl-3-propargyl indole **5** (Scheme 3). At room temperature, substrate **5** yielded a mixture of **6**, derived from the expected tandem 1,2-indole migration–intramolecular cyclopropanation, and a new compound **7**. After a brief reoptimization of the reaction conditions, it was found that at 0 °C, product **6** was selectively obtained in good yield. Fortunately, spiro derivative **7**, derived from the ring expansion of one of the cyclopropane groups^2b^ at the exocyclic alkene of the indole derivative **6**, could be selectively and readily obtained as a single diastereoisomer by heating the reaction mixture at 80 °C for 6 h. We additionally checked that treatment of isolated **6** under gold-catalysis at rt for 4 h also provided **7** (Scheme 3). The structures of both compounds were further supported by X-ray analysis (CCDC 2446429 and 2446430).^18^

**Scheme 3:**
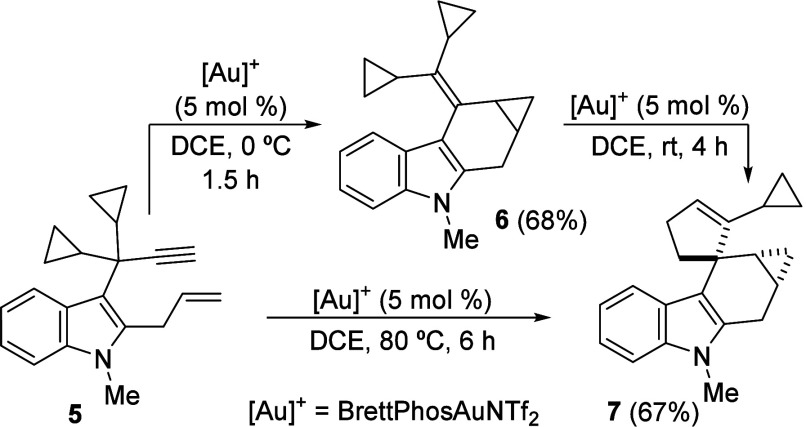
Au­(I)-Catalyzed Intramolecular Cyclopropanation of 2-Allyl-3-propargylindole 5

Finally, we decided to test various 3-propargyl indoles **8** in which the alkene at the C2 position was lengthened. Under the established conditions, related polycyclic compounds **9**, with seven- or eight-membered rings fused with cyclopropane and the indole nucleus, were synthesized with moderate to good yields (Scheme 4). Analogously to the substrates previously used, this process tolerates the presence of alkyl and aromatic groups at different positions of the olefin in the starting material.

**Scheme 4:**
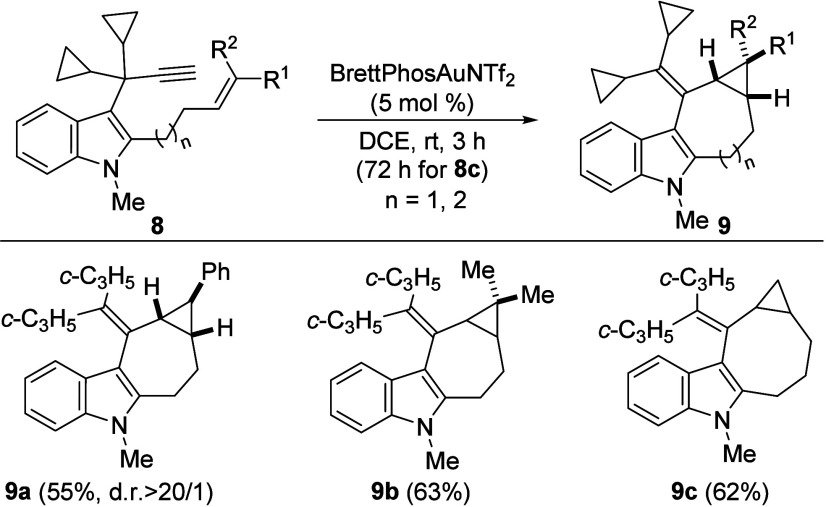
Synthesis of 7- and 8-Membered Polycyclic Indole Derivatives 9

In conclusion, we have developed the inter and intramolecular cyclopropanation of olefins employing terminal 3-propargylindoles as gold carbene sources. The intermolecular version takes place with high *cis*-selectivity, which could be interestingly switched in the presence of silver salts to the corresponding *trans*-cyclopropane derivatives. 3-Propargylindoles bearing alkenyl chains at C-2 evolve through intramolecular cyclopropanations, leading to polycyclic indole derivatives in a stereospecific way. In addition, the results presented herein further support the intermediacy of carbene intermediates in the Au-catalyzed 1,2-indole migrations in 3-propargylindoles.

## Supplementary Material





## Data Availability

The data underlying this study are available in the published article, in its Supporting Information, and openly available in zenodo at https://zenodo.org/records/15696479?preview%20=%201&token%20=%20eyJhbGciOiJIUzUxMiJ9.eyJpZCI6ImI5Mjk5YTUwLWM1M2EtNDU5YS04ZWRjLWU1Nzc5ZTA5MmJmMiIsImRhdGEiOnt9LCJyYW5kb20iOiI5OTVkNWJlY2JiMTFmZTIwM2QyODQ5ODdmMzc1%20N%20mU1YSJ9.b6hwdAnxGIhBqSuZHAYBXpVBVqCe2MkdHhdidXmn07sAFX2VoHyR18-t2GE33KzXXFyb1--p73X_6dTduTo6nQ.

## Notes

FAIR data, including the primary NMR FID files, for compounds S1f,i,k, (*Z*)-S1j, S3a,b, S4, S6a,b, S9a-c, S10–S12, *cis*-2a-d,h,i,l-o, *trans*-2a-d,h,i, 2b,e-g,j,k,p-r, 3a,f-i,k, (*E*)-3a-e,l, (*Z*)-3a,c,d,j, *cis*-4a-f,l, *trans*-4a,c,d,f,j, 4g,h,i,k, 5–7, 8a-c, 9a-c (ZIP)
Experimental details, characterization and NMR data, NMR spectra and X-ray structures (PDF)
