# Supplementary figures and images for: Gold(I)-Catalyzed Tandem 1,2-Indole Migration–Cyclopropanation Reactions of 3‑Propargylindoles with Olefins
Source: Org Lett. 2025 Jul 28;27(31):8662–7. doi: 10.1021/acs.orglett.5c02596 (PMC12340974; doi:10.1021/acs.orglett.5c02596)

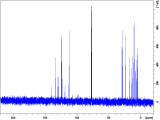

Supplement: Supplementary file 1 [file ol5c02596_si_001.zip › 2r/CNMR/pdata/1/thumb.png]

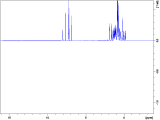

Supplement: Supplementary file 1 [file ol5c02596_si_001.zip › 2r/HNMR/pdata/1/thumb.png]

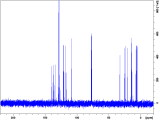

Supplement: Supplementary file 1 [file ol5c02596_si_001.zip › cis-2b/CNMR/pdata/1/thumb.png]

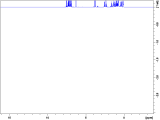

Supplement: Supplementary file 1 [file ol5c02596_si_001.zip › cis-2b/HNMR/pdata/1/thumb.png]

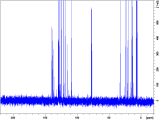

Supplement: Supplementary file 1 [file ol5c02596_si_001.zip › cis-2c/CNMR/pdata/1/thumb.png]

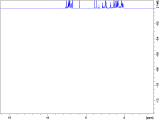

Supplement: Supplementary file 1 [file ol5c02596_si_001.zip › cis-2c/HNMR/pdata/1/thumb.png]

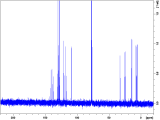

Supplement: Supplementary file 1 [file ol5c02596_si_001.zip › cis-2d/CNMR/pdata/1/thumb.png]

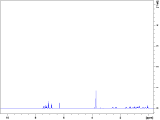

Supplement: Supplementary file 1 [file ol5c02596_si_001.zip › cis-2d/HNMR/pdata/1/thumb.png]
