# Supplementary material for: Gold(I)-Catalyzed Tandem 1,2-Indole Migration–Cyclopropanation Reactions of 3‑Propargylindoles with Olefins
Source: Org Lett. 2025 Jul 28;27(31):8662–7. doi: 10.1021/acs.orglett.5c02596 (PMC12340974; doi:10.1021/acs.orglett.5c02596)

Gold(I)-Catalyzed Tandem 1,2-Indole Migration–Cyclopropanation Reactions of 3-Propargylindoles with Olefins

Lorena Renedo, Marta Solas, Raquel Hernández-Ruiz, Samuel Suárez-Pantiga, Roberto Sanz*

Área de Química Orgánica, Departamento de Química, Facultad de Ciencias, Universidad de Burgos, Pza. Misael Bañuelos s/n, 09001-Burgos, Spain

Supporting Information

Table of Contents

General information S2

Synthesis of 3-propargylindoles 1a-g S2

General procedure A for the synthesis of 3-propargylindoles 1a-c,f,g S2

Synthesis of 3-propargylindole 1d S3

Synthesis of 3-propargylindole 1e S3

Synthesis and characterization data of 2-substituted indoles S1, S2 and S3 S3

General procedure B for the synthesis of 2-alkenyl-indoles S1 S3

General procedure C for the synthesis of (E)-2-alkenylindoles S1b,e,l S6

Synthesis of 2-allyl-1-methyl-1H-indole S2 S7

General procedure D for the synthesis of 2-butenyl and 2-pentenyl-1H-indoles S3 S7

Synthesis and characterization data of terminal 3-propargylindoles 3, 5 and 8 S8

General procedure E for the synthesis of 3-propargylindoles 3, S4, S9, 5 and 8 S8

Synthesis of 3-propargyl indole 3l S16

Intermolecular cyclopropanation reactions: Synthesis and characterization data of 3-(1-cyclopropylvinyl)-1*H*-indole derivatives 2 S17

Optimization studies S17

Study of the silver effect on changing diastereselectivity obtained S20

General procedures F for the synthesis of 3-(1-cyclopropylvinyl)-1H-indole derivatives 2 S21

Limitations on the scope of the intermolecular reaction S29

Intramolecular cyclopropanation reactions of 2-alkenyl-3-propargylindoles 3: Synthesis and characterization data of cyclopenta[1,2-*b*]indole derivatives 4 S31

General procedure G for the synthesis of tetrahydro-1H-cyclopropa[4,5]cyclopenta[1,2-b]indole derivatives 4 S31

Limitations on the scope of the intermolecular reaction S37

Intramolecular cyclopropanation reactions of 2-allyl-3-propargylindole 5 S39

Optimization studies S39

Au(I)-catalyzed intramolecular cyclopropanation of 2-allyl-3-propargyl indole 5 S40

Intramolecular cyclopropanation reactions of 2-(bis)homoallyl-3-propargylindoles 8: Synthesis and characterization data of 7- and 8-membered polycyclic indole derivatives 9 S41

General procedure H for the synthesis cyclohepta and cycloocta[1,2-b]indoles 9 S41

Synthetic procedure for the preparation of *cis*-2a (1 mmol scale): S42

X-Ray Crystallographic Data for 6 and 7 S43

^{1}H and ^{13}C NMR Spectra S45

General information

General methods: All common reagents and solvents were obtained from commercial suppliers and used without any further purification. TLC was performed on aluminum-backed plates coated with silica gel 60 with F_{254} indicator; the chromatograms were visualized under ultraviolet light and/or by staining with a Ce/Mo reagent and subsequent heating. NMR spectra were measured on Varian Mercury-Plus 300 MHz, Bruker Avance 300 MHz and Varian Inova-400 MHz spectrometers. ^{1}H NMR: splitting pattern abbreviations are: s, singlet; br s, broad singlet; d, doublet; t, triplet; q, quartet; dd, double doublet; ddd, doublets of doublets of doublets; ddt, double doublet of triplets; dt, doublet of triplets; dq, doublet of quartets; td, triplet of doublets; qd, quartet of doublets; quin, quintuplet; sext, sextet; hept, heptet; ad, apparent doublet; at, apparent triplet; aq, apparent quartet; as, apparent singlet; m, multiplet; the chemical shifts are reported in ppm using residual solvent peak as reference. ^{13}C NMR spectra were recorded at 75.4 MHz or 100.6 MHz using broadband proton decoupling and chemical shifts are reported in ppm using residual solvent peaks as reference ($CDCl_3$: δ 77.16). Structural assignments were made with additional information from gCOSY, gHSQC, and gHMBC experiments. High resolution mass spectra (HRMS) were recorded on a LC-MS instrument (1260 Infinity, Agilent) equipped with a QTOF analyzer using ESI (+). Low resolution mass spectra (LRMS) measurements were recorded on an Agilent 6890N/5973 Network GC System, equipped with a HP-5MS column. Melting points were measured on a Gallenkamp apparatus using open capillary tubes and are uncorrected.

Synthesis of 3-propargylindoles 1a-g

General procedure A for the synthesis of 3-propargylindoles 1a-c,f,g

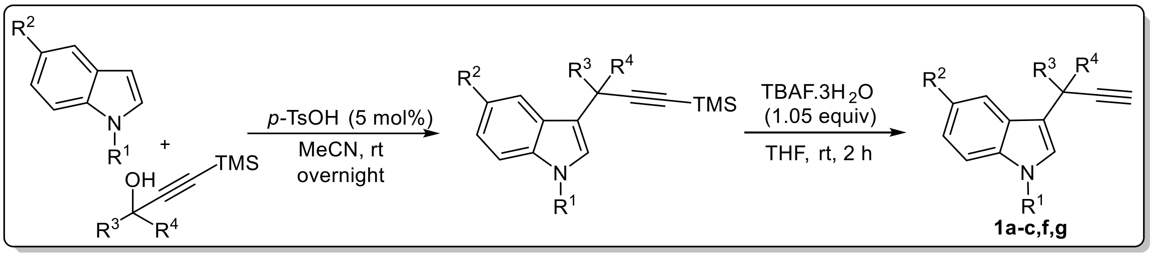

p-TsOH (39 mg, 0.2 mmol, 0.05 equiv) was added to a mixture of the corresponding alkynol (6 mmol, 1.2 equiv) and indole derivative (5 mmol, 1 equiv) in analytical-grade MeCN (5 mL, 1 M). The reaction mixture was stirred at rt until the starting indole had been consumed, as determined by GC–MS and/or TLC (typically overnight). The crude reaction mixture was neutralized by the addition of 1 M NaOH (5 mL). The mixture was extracted with Et_2O (3 × 20 mL) and the combined organic layers were dried over anhydrous Na_2SO_4. The crude trimethylsilyl-functionalized indole intermediate is then treated with TBAF·$3H_2O$ (1.66 g, 5.25 mmol, 1.05 equiv) in THF (5 mL, 1 M) at rt for 2 h. The crude reaction mixture was quenched by the addition of H_2O. The mixture was extracted with Et_2O (3 × 15 mL) and the combined organic layers were dried over anhydrous Na_2SO_4. The solvent was removed under reduced pressure and the residue was purified by silica gel column chromatography using mixtures of hexane and EtOAc as eluents to obtain the corresponding 3-propargylated indoles **1**. The spectroscopic data of indoles **1a-d,f,g** match with those reported in our previous report.[1]

[1]Renedo, L.; Álvarez, E.; Solas, M.; Suárez-Pantiga, S.; Fernández-Rodríguez, M. A.; Sanz, R. *Adv. Synth. Catal.* **2024**, *366*, 2079–2089.

Synthesis of 3-propargylindole 1d

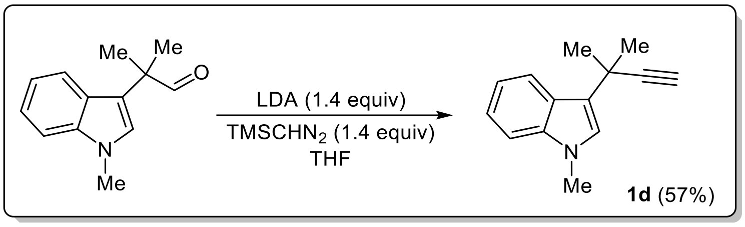

n-BuLi (0.56 mL of a 2.5 M solution in hexane, 1.4 mmol) was added to a solution of *i*Pr_2NH (0.20 mL,1.4 mmol,) in THF (4 mL) at 0 °C and the resulting solution was stirred for 30 min at this temperature. The mixture was cooled to –78 °C and (trimethylsilyl)diazomethane (0.7 mL of a 2 M solution in hexane, 1.4 mmol) was added. After stirring for 30 min, a solution of 2-methyl-2-(1-methyl-1*H*-indol-3-yl)propanal[2] (201 mg, 1 mmol) in THF (2 mL) was added, and the resulting mixture was stirred for 1 h at –78 °C and for 3 h at rt. The mixture was poured over ice-water and extracted with Et_2O (3 × 10 mL). The combined organic layers were dried over anhydrous Na_2SO_4, and the solvent was evaporated under reduced pressure. The crude product was purified by column chromatography (hexane/EtOAc, 50/1) affording pure **1d** as a colorless liquid (112 mg, 57%). The spectroscopic data of indole **1d** match with those reported in our previous report.[1]

Synthesis of 3-propargylindole 1e

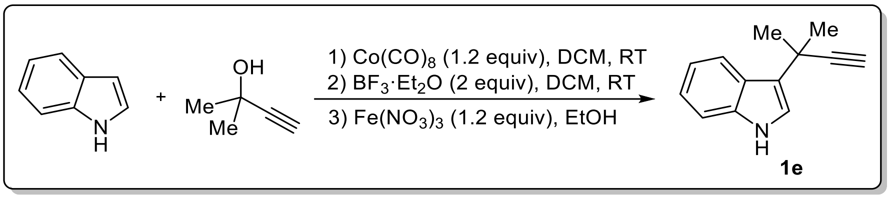

3-(2-Methylbut-3-yn-2-yl)-1*H*-indole (1e): This product was prepared as previously described in the literature,[3] although we obtained it with a low overall yield (ca. 10%), after the three steps starting from commercially available 1,1-dimethylpropargyl alcohol.

Synthesis and characterization data of 2-substituted indoles S1, S2 and S3

General procedure B for the synthesis of 2-alkenyl-indoles S1

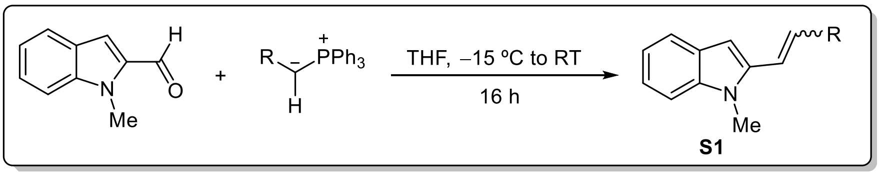

n-BuLi (5.2 mL of a 2.5 M solution in hexane, 13 mmol, 1.3 equiv) was added dropwise to a solution of the corresponding phosphonium bromide (12 mmol, 1.2 equiv) in anhydrous THF (10 mL, 1.2 M) at –15 °C under a nitrogen atmosphere. The mixture was stirred at this temperature for 30 min. A solution of 1-methyl-1*H*-indole-2-carbaldehyde (1.59 g, 10 mmol, 1 equiv), in anhydrous THF (15 mL, 0.66 M) was then added

[2]Reddy, R.; Jaquith, J. B.; Neelagiri, V. R.; Hanna, S. S.; Durst, T. *Org. Lett.* **2002**, *4*, 695–697.
[3]Nakagawa, M.; Ma, J.; Hino, T. *Heterocycles* **1990**, *30*, 451–462.

dropwise to the ylide solution at –15 °C, and the mixture was further stirred at rt overnight. The crude reaction mixture was quenched with water, and the mixture was extracted with Et_2O (3 × 25 mL). The combined organic layers were dried over anhydrous Na_2SO_4, and the solvent was removed under reduced pressure. The crude residue was purified by silica gel column chromatography using mixtures of hexane and EtOAc as eluents to afford the corresponding C2-alkenyl indoles **S1** as a variable mixture of geometrical isomers, which were carefully purified, trying to obtain one of the geometrical isomers in pure form.

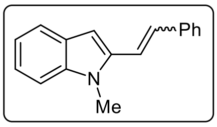

1-Methyl-2-styryl-1*H*-indole (S1a):[4] Following the general procedure B with benzyltriphenylphosphonium bromide (5.18 g, 12 mmol), the crude product was purified by column chromatography (hexane/EtOAc, 10/1). R_f = 0.29 (hexane/EtOAc, 10/1). Obtained as a ca. 1/1.1 mixture of *E/Z* isomers, which were independently isolated by column chromatography, yielding pure (***E***)-**S1a** (932 mg, 40%) and (***Z***)-**S1a** (1.10 g, 45%). The spectroscopic data of these compounds match those reported in the literature.

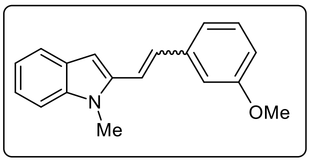

2-(3-Methoxystyryl)-1-methyl-1*H*-indole (S1c):[5] Following the general procedure B with (3-methoxybenzyl)triphenylphosphonium bromide (5.56 g, 12 mmol), the crude product was purified by column chromatography (hexane/EtOAc, 10/1). R_f = 0.36 (hexane/EtOAc, 10/1). Obtained as a ca. 1/1 mixture of *E*/*Z* isomers, which were independently isolated by column chromatography, yielding pure (***E***)-**S1c** (921 mg, 35%) and (***Z***)-**S1c** (1 g, 38%). The spectroscopic data of these compounds match with those reported in the literature.

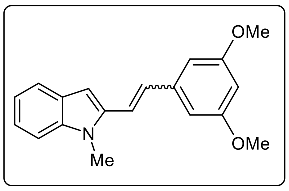

2-(3,5-Dimethoxystyryl)-1-methyl-1*H*-indole (S1d):[4] Following the general procedure B with (3,5-methoxybenzyl)triphenylphosphonium bromide (5.90 g, 12 mmol), the crude product was purified by column chromatography (hexane/EtOAc, 10/1). R_f = 0.23 (hexane/EtOAc, 5/1). Obtained as a ca. 1/1.6 mixture of *E/Z* isomers, which were independently isolated by column chromatography, yielding pure (***E***)-**S1d** (879 mg, 30%) and a 8/1 mixture of (***Z***)/(***E***)-**S1d** (1.40 g, 48%). The spectroscopic data of these compounds match with those reported in the literature.

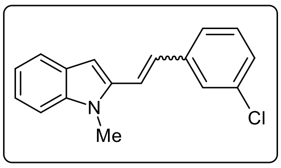

2-(3-Chlorostyryl)-1-methyl-1*H*-indole (S1f): Following the general procedure B with (3-chlorobenzyl)triphenylphosphonium bromide (5.60 g, 12 mmol), the crude product was purified by column chromatography (hexane/EtOAc, 10/1) affording pure **S1f** as a yellow oil (2.20 g, 81%). R_f = 0.21 (hexane/EtOAc, 10/1). Obtained and isolated as a ca. 1/1 mixture of *E/Z* isomers. Data for both diastereoisomers: **^{1}H NMR** (300 MHz, $CDCl_3$) *δ* (ppm): 8.05 (ad, *J* = 7.7 Hz, 1H), 7.94 (ad, *J* = 7.7 Hz, 1H), 7.84 (as, 1H), 7.74 (as, 1H), 7.65–7.49 (m, 11H), 7.43–7.36 (m, 1H), 7.34 (as, 2H), 7.16 (as, 1H), 6.90–6.84 (m, 3H).3.82 (s, 3H), 3.76 (s, 3H). **^{13}C NMR** (75.4 MHz, $CDCl_3$) *δ* (ppm): 138.9, 138.8, 138.1, 137.5, 137.2, 135.4, 134.4, 134.0, 130.8, 129.8, 129.5, 128.6, 127.8, 127.7, 127.5, 127.3, 126.4, 125.9, 124.5, 121.9, 121.8, 120.5, 120.5, 120.2, 119.9, 119.7, 118.1, 109.23, 109.20, 101.9, 99.6, 29.6, 29.3. **HRMS** (ESI-TOF) *m/z*: calcd for $C_{17}H_{15}NCl^+$ $[M+H]^+$ 268.0897; found 268.0888.

---

[4] a) Masuda, K.; Ohmura, T.; Suginome, M. *Organometallics.* **2011**, *30*, 6, 1322–1325. b) Rojas-Martín, J.; Veguillas, M.; Ribagorda, M.; Carreño, M. C. *Org. Lett.* **2013**, *15*, 22, 5686–5689.

[5] Zhao, G.; Bignon, J.; Levaique, H.; Dubois, J.; Alami, M.; Provot, O. *J. Org. Chem.* **2018**, *83*, 24, 15323–15332.

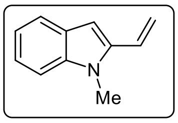

1-Methyl-2-vinyl-1*H*-indole (S1g):[6] Following the general procedure B with $Ph_3P^+MeBr^-$ (4.28 g, 12 mmol), the crude product was purified by column chromatography (hexane/EtOAc, 5/1), affording pure **S1g** as a light yellow oil (801 mg, 51%). R_f = 0.25 (hexane/EtOAc, 5/1). The spectroscopic data of this compound match with those reported in the literature.

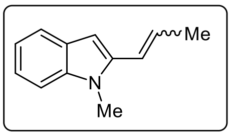

1-Methyl-2-(prop-1-en-1-yl)-1*H*-indole (S1h):[7] Following the general procedure B with ethyltriphenylphosphonium bromide (4.44 g, 12 mmol), the crude product was purified by column chromatography (hexane/EtOAc, 10/1), affording pure **S1h** as a light yellow oil (1.50 g, 87%). Obtained and isolated as a ca. 1/1.7 mixture of *E/Z* isomers. R_f = 0.36 (hexane/EtOAc, 10/1). The spectroscopic data of this compound match with those reported in the literature.

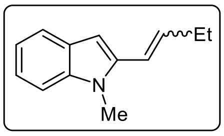

2-(But-1-en-1-yl)-1-methyl-1*H*-indole (S1i): Following the general procedure B with propyltriphenylphosphonium bromide (4.62 g, 12 mmol), the crude product was purified by column chromatography (hexane/EtOAc, 10/1) affording pure **S1i** as a yellow oil (1.55 g, 84%). R_f = 0.24 (hexane/EtOAc, 10/1). Obtained and isolated as a ca. 1/1 mixture of *E/Z* isomers. Data for both diastereoisomers: **^{1}H NMR** (300 MHz, $CDCl_3$) δ (ppm): 7.78–7.63 (m, 2H), 7.43–7.20 (m, 6H), 6.75–6.61 (m, 2H), 6.57–6.40 (m, 3H), 6.04–5.91 (m, 1H), 3.80 (s, 3H), 3.77 (s, 3H), 2.67–2.48 (m, 2H), 2.48–2.36 (m, 2H), 1.40–1.16 (m, 6H). **^{13}C NMR** (75.4 MHz, $CDCl_3$) δ (ppm): 138.9, 137.7, 137.5, 137.1, 136.4, 136.1, 128.1, 128.0, 121.5, 121.2, 120.4, 120.2, 119.7, 119.6, 118.0, 117.3, 109.1, 101.9, 97.9, 29.79, 29.75, 26.5, 22.8, 14.2, 13.6. **HRMS** (ESI-TOF) *m/z*: calcd for $C_{13}H_{16}N^+$ $[M+H]^+$ 186.1277; found 186.1278.

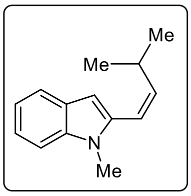

(*Z*)-1-Methyl-2-(3-methylbut-1-en-1-yl)-1*H*-indole ((*Z*)-S1j): Following the general procedure B with isobutyltriphenylphosphonium bromide (4.80 g, 12 mmol), but using NaHMDS as base, the crude product was purified by column chromatography (hexane/EtOAc, 10/1), affording pure **S1j** as a yellow oil (1.40 g, 69%). R_f = 0.22 (hexane/EtOAc, 10/1). Obtained and isolated as a ca. 1/8 mixture of *E/Z* isomers. Data for the major *Z*-diastereoisomer: **^{1}H NMR** (300 MHz, $CDCl_3$) δ (ppm): 7.74 (ad, J = 7.8 Hz, 1H), 7.40 (ad, J = 8.0 Hz, 1H), 7.33 (at, J = 7.5 Hz, 1H), 7.24 (at, J = 6.8 Hz, 1H), 6.66 (as, 1H), 6.40 (d, J = 11.6 Hz, 1H), 5.88–5.77 (m, 1H), 3.78 (s, 3H), 3.30–3.15 (m, 1H), 1.26 (s, 3H), 1.23 (s, 3H). **^{13}C NMR** (75.4 MHz, $CDCl_3$) δ (ppm): 143.4, 137.1, 136.4, 128.0, 121.5, 120.4, 119.6, 115.6, 109.2, 101.4, 29.8, 28.0, 22.9. **HRMS** (ESI-TOF) *m/z*: calcd for $C_{14}H_{17}N^+$ $[M+H]^+$ 200.1434; found 200.1441.

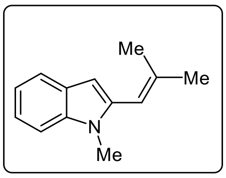

1-Methyl-2-(2-methylprop-1-en-1-yl)-1*H*-indole (S1k): Following the general procedure B with isopropyltriphenylphosphonium iodide (5.18 g, 12 mmol), the crude product was purified by column chromatography (hexane/EtOAc, 10/1), affording pure **S1k** as a yellow oil (1.70 g, 92%). R_f = 0.25 (hexane/EtOAc, 10/1). **^{1}H NMR** (500 MHz, $CDCl_3$) δ (ppm): 7.82–7.73 (m, 1H), 7.46–7.40 (m, 1H), 7.40–7.32 (m, 1H), 7.32–7.24 (m, 1H), 6.67–6.53 (m, 1H), 6.42–6.33 (m, 1H), 3.79 (s, 3H), 2.17 (s, 3H), 2.16 (s, 3H). **^{13}C NMR** (125 MHz, $CDCl_3$) δ (ppm): 139.6 (C),

---

[6] Cao,Y.-J.; Cheng, H.-G.; Lu, L.-Q.; Zhang, J.-J.; Cheng, Y.; Chen, J.-R.; Xiao, W.-J. *Adv. Synth. Catal.* **2011**, *353*, 617–623.
[7] Wang, X.-F.; Chen, J.-R.; Cao, Y.-J.; Cheng, H.-G.; Xiao, W.-J. *Org. Lett.* **2010**, *12*, 1140–1143.

137.6, 136.8, 128.1, 121.1, 120.1, 119.4, 114.6, 109.0, 101.2, 29.7, 27.0, 20.3. **HRMS** (ESI-TOF) *m/z*: calcd for $C_{13}H_{16}N^+$ $[M+H]^+$ 186.1277; found 186.1279.

General procedure C for the synthesis of (E)-2-alkenylindoles S1b,e,l

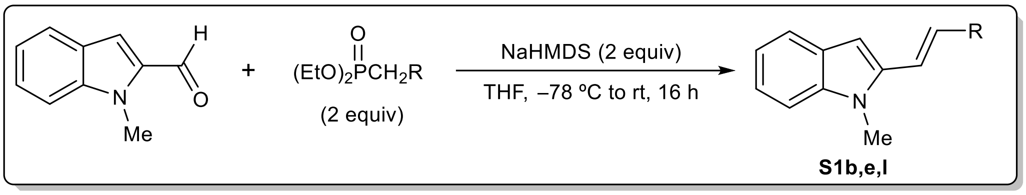

NaHMDS (9 mL of a 1 M solution in hexanes, 9 mmol, 1.2 equiv) was added to a solution of the appropriate diethyl phosphonate (9 mmol, 1.2 equiv) in anhydrous THF (30 mL, 0.3 M) at –78 °C, and the resulting solution was stirred at rt for 30 min. After cooling to –50 °C, a solution of 1-methyl-1*H*-indole-2-carbaldehyde (1.19 g, 7.5 mmol, 1 equiv) in anhydrous THF (7.5 mL, 1 M) was added dropwise. The cooling bath was removed, and the reaction mixture was further stirred at rt until complete consumption of the aldehyde, as monitored by GC–MS. The reaction was quenched by the addition of water, and the mixture was extracted with Et_2O (3 × 20 mL). The combined organic layers were dried over anhydrous Na_2SO_4, and the solvent was removed under reduced pressure. The crude residue was purified by silica gel column chromatography using mixtures of hexane and EtOAc as eluents to obtain the corresponding C2-alkenyl indoles **S1** as pure geometrical *E* isomers.

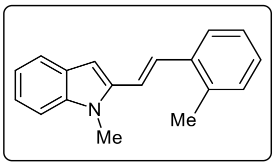

(*E*)-1-Methyl-2-(2-methylstyryl)-1*H*-indole ((*E*)-S1b):[4] Following the general procedure C with diethyl 2-methylbenzylphosphonate (2.18 g, 9 mmol), the crude product was purified by column chromatography (hexane/EtOAc, 10/1) affording pure (***E***)-**S1b** as a light yellow oil (1.07 g, 58%). R_f = 0.25 (hexane/EtOAc, 10/1). The spectroscopic data of this compound match with those reported in the literature.

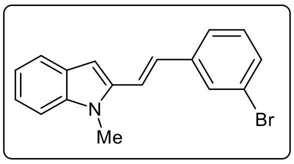

(*E*)-2-(3-Bromostyryl)-1-methyl-1*H*-indole ((*E*)-S1e): Following the general procedure C with diethyl 3-bromobenzylphosphonate (2.76 g, 9 mmol), the crude product was obtained in pure form without further purification as an orange oil (1.45 g, 62%). R_f = 0.20 (hexane/EtOAc, 10/1). **^{1}H NMR** (300 MHz, $CDCl_3$) δ (ppm): 7.78–7.65 (m, 2H), 7.52–7.38 (m, 2H), 7.36–7.27 (m, 2H), 7.27–7.18 (m, 2H), 7.13 (d, *J* = 4.3 Hz, 2H), 6.89 (s, 1H), 3.77 (s, 3H). **^{13}C NMR** (75.4 MHz, $CDCl_3$) δ (ppm): 139.4, 138.4, 137.8, 130.6, 130.3, 129.1, 129.0, 128.0, 125.3, 123.1, 122.2, 120.7, 120.2, 118.5, 109.4, 99.8, 29.9. **HRMS** (ESI-TOF) *m/z*: calcd for $C_{17}H_{15}BrN^+$ $[M+H]^+$ 312.0382; found 312.0382.

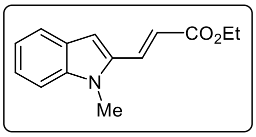

Ethyl (*E*)-3-(1-methyl-1*H*-indol-2-yl)acrylate ((*E*)-S1l):[8] Following the general procedure C with triethyl phosphonoacetate (1.87 g, 9 mmol), the crude product was purified by column chromatography (hexane/EtOAc, 5/1) affording pure (***E***)-**S1l** as a light yellow oil (1.22 g, 71%). R_f = 0.24 (hexane/EtOAc, 5/1). The spectroscopic data of this compound match with those reported in the literature.

[8] Rajasekar,S.; Anbarasan, P. *J. Org. Chem*. **2019**, *84*, 7747–7761.

Synthesis of 2-allyl-1-methyl-1H-indole S2

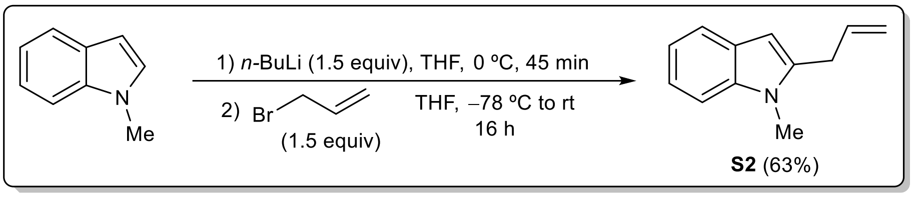

n-BuLi (3 mL of a 2.5 M solution in hexane, 7.5 mmol) was slowly added to a solution of 1-methyl-1*H*-indole (655 mg, 5 mmol) in anhydrous THF (10 mL) at 0 °C under a nitrogen atmosphere. The resulting mixture was stirred at this temperature for 45 min. Allyl bromide (908 mg, 7.5 mmol) was added at –78 °C, and the reaction mixture was allowed to warm to rt and and stirred overnight. The reaction was quenched with aqueous NH_4Cl (10 mL), and the mixture was extracted with Et_2O (3 × 15 mL). The combined organic layers were dried over anhydrous Na_2SO_4, and the solvent was removed under reduced pressure. The residue was purified by silica gel column chromatography using a 20/1 mixture of hexane and EtOAc as eluent to afford 2-allyl-1-methyl-1*H*-indole **S2** as an orange oil (627 mg, 63%). The spectroscopic data of this compound match with those reported in the literature.[9]

General procedure D for the synthesis of 2-butenyl and 2-pentenyl-1H-indoles S3[10]

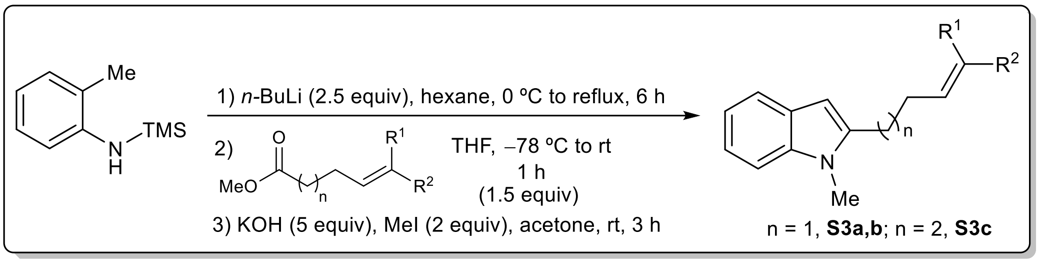

n-BuLi (5 mL of a 2.5 M solution in hexane, 12.5 mmol, 2.5 equiv) was slowly added to a solution of *N*-trimethylsilyl-*o*-toluidine[11] (897 mg, 5 mmol, 1 equiv) in anhydrous *n*-hexane (30 mL, 0.16 M) at 0 °C under a nitrogen atmosphere. The resulting mixture was refluxed (oil bath) for 6 h. The corresponding ester derivative (7.5 mmol, 1.5 equiv) in THF (5 mL, 1.5 M) was then added at –78 °C, and the reaction mixture was stirred at rt until the starting aniline was fully consumed, as monitored by GC–MS and/or TLC. The crude reaction mixture was quenched with aqueous NH_4Cl (15 mL), and the mixture was extracted with Et_2O (3 × 15 mL). The combined organic layers were dried over anhydrous Na_2SO_4, and the solvent was removed under reduced pressure. The residue, containing the corresponding crude 2-butenyl or 2-pentenyl-1*H*-indole, was dissolved in acetone (15 mL, 0.33 M) and treated with MeI (0.62 mL, 10 mmol, 2 equiv) and KOH (1.40 g, 25 mmol, 5 equiv). The resulting mixture was stirred at rt for 3 h. The reaction was then quenched with water (15 mL), and extracted with Et_2O (3 × 10 mL). The combined organic layers were dried over anhydrous Na_2SO_4, and the solvent was removed under reduced pressure. The residue was purified by silica gel column chromatography using mixtures of hexane and EtOAc as eluents to afford indole derivatives **S3**.

---

[9] Ishikura, M.; Terashima, M. *J. Chem. Soc., Chem. Commun*. **1989**, 727–728.
[10] Liu, C.; Han, X.; Wang, X.; Widenhoefer, R. A. *J. Am. Chem. Soc.* **2004**, *126*, 3700–3701.
[11] Smith, A. B. III; Visnick, M.; Haseltine, J. N.; Sprengeler, P. A. *Tetrahedron* **1986**, *42*, 2957–2969.

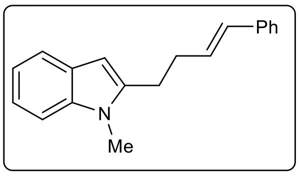

(*E*)-1-Methyl-2-(4-phenylbut-3-en-1-yl)-1*H*-indole (S3a): Following the general procedure D with ethyl (*E*)-5-phenylpent-4-enoate[12] (1.53 g, 7.5 mmol), the crude product was purified by column chromatography (hexane/EtOAc, 7/1), affording **S3a** as a yellow oil (802 mg, 41%). This compound could not be properly purified and unknown impurities were present. R_f = 0.20 (hexane/EtOAc, 5/1). **^{1}H NMR** (300 MHz, $CDCl_3$) δ (ppm): 7.59 (d, *J* = 7.7 Hz, 1H), 7.42–7.30 (m, 5H), 7.30–7.16 (m, 2H), 7.12 (t, *J* = 7.3 Hz, 1H), 6.54 (d, *J* = 15.9 Hz, 1H), 6.44–6.31 (m, 2H), 3.73 (s, 3H), 3.02–2.91 (m, 2H), 2.70 (dd, *J* = 14.8, 7.0 Hz, 2H). **^{13}C NMR** (75.4 MHz, $CDCl_3$) δ (ppm): 140.5, 137.6, 137.5, 131.0, 129.5, 128.7, 128.0, 127.3, 126.2, 120.8, 120.0, 119.4, 108.9, 99.1, 32.2, 29.6, 27.0. **HRMS** (ESI-TOF) *m/z*: calcd for $C_{19}H_{20}N^+$ $[M+H]^+$ 262.1590; found 262.1594.

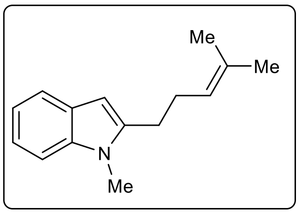

1-Methyl-2-(4-methylpent-3-en-1-yl)-1*H*-indole (S3b): Following the general procedure with ethyl 5-methylhex-4-enoate[13] (1.17 g, 7.5 mmol), the crude product was purified by column chromatography (hexane/EtOAc, 7/1), affording pure **S3b** as a yellow oil (639 mg, 40%). R_f = 0.23 (hexane/EtOAc, 10/1). **^{1}H NMR** (500 MHz, $CDCl_3$) δ (ppm): 7.62 (d, *J* = 7.7 Hz, 1H), 7.34 (d, *J* = 8.1 Hz, 1H), 7.23 (t, *J* = 7.5 Hz, 1H), 7.15 (t, *J* = 7.3 Hz, 1H), 6.35 (s, 1H), 5.34 (dt, *J* = 8.2, 4.1 Hz, 1H), 3.73 (s, 3H), 2.87–2.79 (m, 2H), 2.51 (q, *J* = 7.4 Hz, 2H), 1.81 (s, 3H), 1.73 (s, 3H). **^{13}C NMR** (125 MHz, $CDCl_3$) δ (ppm): 141.2, 137.5, 132.7, 128.1, 123.5, 120.6, 119.9, 119.3, 108.8, 98.8, 29.5, 27.4, 27.2, 25.8, 17.9. **LRMS** *m/z* (%): 213 (M^+, 19), 144 (100), 115 (12). **HRMS** (ESI-TOF) *m/z*: calcd for $C_{15}H_{20}N^+$ $[M+H]^+$ 214.1590; found 214.1594.

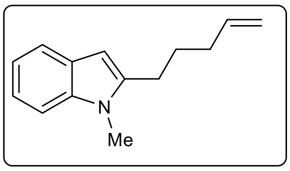

1-Methyl-2-(pent-4-en-1-yl)-1*H*-indole (S3c): Following the general procedure with methyl 5-hexenoate (1.06 g, 7.5 mmol), the crude product was purified by column chromatography (hexane/EtOAc, 7/1), affording pure **S3c** as a yellow oil (627 mg, 42%). R_f = 0.21 (hexane/EtOAc, 10/1). The spectroscopic data of this compound match with those reported in the literature.[14]

Synthesis and characterization data of terminal 3-propargylindoles 3, 5 and 8

General procedure E for the synthesis of 3-propargylindoles 3, S4, S9, 5 and 8

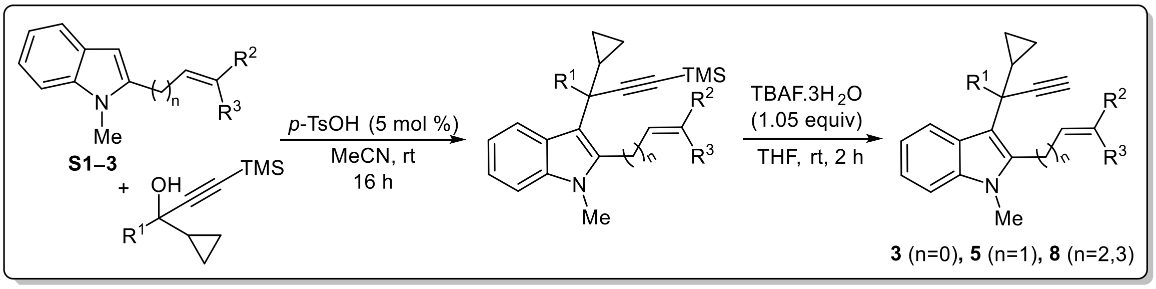

---

[12] Cruz, P. De J.; Crawford, E. T.; Liu, S.; Johnson, J. S. *J. Am. Chem. Soc.* **2021**, *143*, 16264–16273.
[13] Li, Z.; García-Domínguez, A.; Nevado, C. *Angew. Chem. Int. Ed.* **2016**, *55*, 6938–6941.
[14] Li, C.; Liu, H.; Liao, J.; Cao, Y.; Liu, X.; Xiao, W. *Org. Lett.* **2007**, *9*, 1847–1850.

p-TSOH (23 mg, 0.15 mmol, 0.05 equiv) was added to a mixture of the corresponding alkynol (3.6 mmol, 1.2 equiv) and indole derivative (3 mmol, 1 equiv) in analytical-grade MeCN (5 mL, 0.6 M). The reaction mixture was stirred at rt until complete consumption of the starting indole, as determined by GC–MS and/or TLC. The crude reaction mixture was neutralized by the addition of 1 M NaOH (5 mL). The mixture was extracted with Et_2O (3 × 15 mL), and the combined organic layers were dried over anhydrous Na_2SO_4. The resulting trimethylsilyl-functionalized indole intermediate was then treated with TBAF·$3H_2O$ (996 mg, 3.15 mmol, 1.05 equiv) in THF (5 mL, 0.6 M) at rt for 2 h. The reaction was quenched by the addition of water, and the mixture was extracted with Et_2O (3 × 15 mL). The combined organic layers were dried over anhydrous Na_2SO_4, and the solvent was removed under reduced pressure. The residue was purified by silica gel column chromatography using mixtures of hexane and EtOAc as eluents to afford the corresponding 3-propargylated indoles **3**, **5** and **8**.

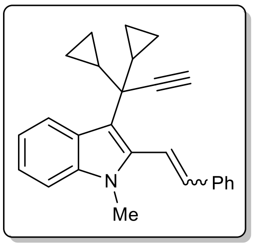

3-(1,1-Dicyclopropylprop-2-yn-1-yl)-1-methyl-2-styryl-1*H*-indole (3a): Following the general procedure E with **S1a** (699 mg, 3 mmol) and 1,1-dicyclopropyl-3-(trimethylsilyl)prop-2-yn-1-ol (750 mg, 3.6 mmol), the crude product was purified by column chromatography (hexane/EtOAc, 10/1) affording pure **3a** as a light yellow oil (800 mg, 76%). R_f = 0.37 (hexane/EtOAc, 10/1). Obtained and isolated as a ca. 1.2/1 mixture of *E/Z* diastereoisomers. Data for both diastereoisomers: **^{1}H NMR** (300 MHz, $CDCl_3$): δ (ppm) = 8.31 (ad, *J* = 8.0 Hz, 1H), 8.23 (ad, *J* = 8.2 Hz, 1H), 7.75 (d, *J* = 16.7 Hz, 1H), 7.56 (ad, *J* = 7.3 Hz, 2H), 7.44 (at, *J* = 7.4 Hz, 2H), 7.36 (ad, *J* = 7.5 Hz, 2H), 7.31–7.24 (m, 3H), 7.23–7.13 (m, 5H), 7.05–6.97 (m, 2H), 6.98 (d, *J* = 12.2 Hz, 1H), 6.82 (d, *J* = 12.2 Hz, 1H), 6.67 (d, *J* = 16.7 Hz, 1H), 3.80 (s, 3H), 3.36 (s, 3H), 2.33 (s, 1H), 2.29 (s, 1H), 1.71–1.59 (m, 4H), 0.88–0.71 (m, 4H), 0.72–0.35 (m, 12H). **^{13}C NMR** (75.4 MHz, $CDCl_3$): δ (ppm) = 138.2, 137.4, 137.2, 136.7, 134.70, 134.66, 134.3, 132.7, 129.0, 128.8, 128.6, 128.1, 127.9, 127.1, 126.9, 126.5, 122.20, 122.17, 121.9, 121.8, 121.4, 120.8, 119.2, 119.0, 116.5, 115.4, 109.7, 84.4, 83.5, 72.5, 72.5, 43.2, 43.1, 31.5, 29.9, 21.2, 3.7, 3.3, 2.7, 2.6. **EI-LRMS** *m/z* (%): 351 (M^+, 100), 281 (27), 245 (20). **HRMS** (ESI-TOF) *m/z*: calcd for $C_{26}H_{26}N^+$ $[M+H]^+$ 352.2060; found 352.2061.

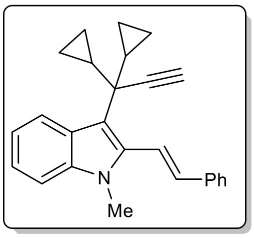

(*E*)-3-(1,1-Dicyclopropylprop-2-yn-1-yl)-1-methyl-2-styryl-1*H*-indole ((*E*)-3a): Following the general procedure E with (***E***)**-S1a** (699 mg, 3 mmol) and 1,1-dicyclopropyl-3-(trimethylsilyl)prop-2-yn-1-ol (750 mg, 3.6 mmol), the crude product was purified by column chromatography (hexane/EtOAc, 10/1) affording pure (***E***)**-3a** as a light yellow solid (674 mg, 64%). R_f = 0.34 (hexane/EtOAc, 10/1); m.p. = 133–135 °C. **^{1}H NMR** (300 MHz, $CDCl_3$) δ (ppm): 8.44 (ad, *J* = 8.2 Hz, 1H), 7.95 (d, *J* = 16.8 Hz, 1H), 7.75–7.69 (m, 2H), 7.59 (at, *J* = 7.5 Hz, 2H), 7.53–7.42 (m, 3H), 7.38–7.32 (m, 1H), 6.83 (d, *J* = 16.8 Hz, 1H), 3.91 (s, 3H), 2.49 (s, 1H), 1.95–1.74 (m, 2H), 1.10–0.96 (m, 2H), 0.91–0.75 (m, 4H), 0.73–0.62 (m, 2H). **^{13}C NMR** (75.4 MHz, $CDCl_3$) δ (ppm): 138.1, 137.1, 134.6, 134.5, 128.9, 128.0, 126.8, 126.4, 122.1, 121.8, 120.7, 119.2, 116.4, 109.7, 84.3, 72.5, 43.1, 31.4, 21.2, 3.7, 2.7. **EI-LRMS** *m/z* (%): 351 (M^+, 100), 281 (27), 245 (20). **HRMS** (ESI-TOF) *m/z*: calcd for $C_{26}H_{26}N^+$ $[M+H]^+$ 352.2060; found 352.2061.

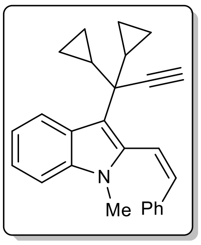

(*Z*)-3-(1,1-Dicyclopropylprop-2-yn-1-yl)-1-methyl-2-styryl-1*H*-indole ((*Z*)-3a): Following the general procedure E with (***Z***)-**S1a** (699 mg, 3 mmol) and 1,1-dicyclopropyl-3-(trimethylsilyl)prop-2-yn-1-ol (750 mg, 3.6 mmol), the crude product was purified by column chromatography (hexane/EtOAc, 10/1) affording pure (***Z***)-**3a** as a light yellow solid (569 mg, 54%). R_f = 0.35 (hexane/EtOAc, 10/1); m.p. = 120–122 °C. Obtained and isolated as a ca. 1/>20 mixture of *E/Z* diastereoisomers. Data for the major (*Z*) diastereoisomer: **^{1}H NMR** (300 MHz, $CDCl_3$) δ (ppm): 8.36 (d, *J* = 8.0 Hz, 1H), 7.34–7.29 (m, 2H), 7.29–7.17 (m, 4H), 7.09–7.06 (m, 2H), 7.02 (d, *J* = 12.3 Hz, 1H), 6.86 (d, *J* = 12.2 Hz, 1H), 3.40 (s, 3H), 2.33 (s, 1H), 1.77–1.62 (m, 2H), 0.90–0.79 (m, 2H), 0.78–0.65 (m, 2H), 0.65–0.54 (m, 2H), 0.54–0.39 (m, 2H). **^{13}C NMR** (75.4 MHz, $CDCl_3$) δ (ppm): 137.4, 136.6, 134.3, 132.7, 128.8, 128.6, 127.8, 127.1, 122.2, 121.9, 121.4, 119.0, 115.4, 109.7, 83.5, 72.5, 43.1, 29.9, 21.2, 20.7, 3.3, 2.6. **EI-LRMS** *m/z* (%): 351 (M^+, 67), 323 (48), 322 (100). **HRMS** (ESI-TOF) *m/z*: calcd for $C_{26}H_{26}N^+$ $[M+H]^+$ 352.2060; found 352.2062.

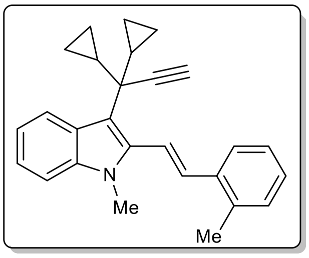

(*E*)-3-(1,1-Dicyclopropylprop-2-yn-1-yl)-1-methyl-2-(2-methylstyryl)-1*H*-indole ((*E*)-3b): Following the general procedure E with (***E***)-**S1b** (741 mg, 3 mmol) and 1,1-dicyclopropyl-3-(trimethylsilyl)prop-2-yn-1-ol (750 mg, 3.6 mmol), the crude product was purified by column chromatography (hexane/EtOAc, 10/1) affording pure (***E***)-**3b** as a light yellow solid (788 mg, 72%). R_f = 0.37 (hexane/EtOAc, 10/1)1; m.p. = 142–144 °C. **^{1}H NMR** (300 MHz, $CDCl_3$): δ (ppm) = 8.39 (ad, *J* = 8.1 Hz, 1H), 7.80 (ad, *J* = 6.9 Hz, 1H), 7.75 (d, *J* = 16.6 Hz, 1H).7.52–7.34 (m, 5H), 7.34–7.21 (m, 1H), 7.05 (d, *J* = 16.6 Hz, 1H), 3.92 (s, 3H), 2.59 (s, 3H), 2.45 (s, 1H), 1.86–1.75 (m, 2H), 1.05–0.93 (m, 2H), 0.84–0.70 (m, 4H), 0.67–0.49 (m, 2H). **^{13}C NMR** (75.4 MHz, $CDCl_3$): δ (ppm) = 138.0, 136.2, 135.8, 134.9, 132.9, 130.7, 128.0, 126.9, 126.5, 125.1, 122.2, 121.8, 119.2, 116.3, 109.7, 84.3, 72.6, 43.2, 31.5, 21.2, 20.0, 3.6, 2.7. **EI-LRMS** *m/z* (%): 365 (M^+, 25), 363 (37), 335 (100). **HRMS** (ESI-TOF) *m/z*: calcd for $C_{27}H_{28}N^+$ $[M+H]^+$ 366.2216; found 366.2222.

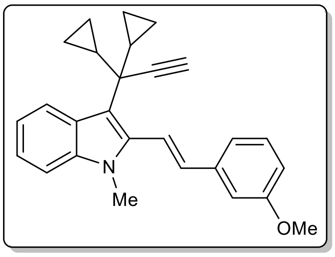

(*E*)-3-(1,1-Dicyclopropylprop-2-yn-1-yl)-2-(3-methoxystyryl)-1-methyl-1*H*-indole ((*E*)-3c): Following the general procedure E with (***E***)-**S1c** (789 mg, 3 mmol) and 1,1-dicyclopropyl-3-(trimethylsilyl)prop-2-yn-1-ol (750 mg, 3.6 mmol), the crude product was purified by column chromatography (hexane/EtOAc, 10/1) affording pure (***E***)-**3c** as a light yellow oil (514 mg, 45%). R_f = 0.26 (hexane/EtOAc, 10/1). **^{1}H NMR** (300 MHz, $CDCl_3$): δ (ppm) = 8.30 (ad, *J* = 8.2 Hz, 1H), 7.82 (d, *J* = 16.7 Hz, 1H), 7.45–7.37 (m, 2H), 7.36–7.28 (m, 1H), 7.25–7.13 (m, 3H), 6.96 (ad, *J* = 8.2 Hz, 1H), 6.70 (d, *J* = 16.7 Hz, 1H), 3.94 (s, 3H), 3.84 (s, 3H), 2.39 (s, 1H), 1.78–1.66 (m, 2H), 0.93–0.86 (m, 2H), 0.76–0.62 (m, 4H), 0.61–0.50 (m, 2H). **^{13}C NMR** (75.4 MHz, $CDCl_3$): δ (ppm) = 160.1, 138.6, 138.2, 134.5, 134.4, 129.9, 126.8, 122.2, 121.8, 121.1, 119.2, 119.1, 116.6, 113.4, 112.0, 109.7, 84.3, 72.5, 55.3, 43.1, 31.5, 21.2, 3.7, 2.7. **EI-LRMS** *m/z* (%): 381 (M^+, 70), 262 (100), 218 (91). **HRMS** (ESI-TOF) *m/z*: calcd for $C_{27}H_{28}NO^+$ $[M+H]^+$ 382.2165; found 382.2168.

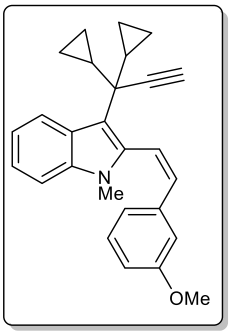

(*Z*)-3-(1,1-Dicyclopropylprop-2-yn-1-yl)-2-(3-methoxystyryl)-1-methyl-1*H*-indole ((*Z*)-3c): Following the general procedure E with (***Z***)-**S1c** (789 mg, 3 mmol) and 1,1-dicyclopropyl-3-(trimethylsilyl)prop-2-yn-1-ol (750 mg, 3.6 mmol), the crude product was purified by column chromatography (hexane/EtOAc, 10/1) affording pure (***Z***)-**3c** as a light orange oil (502 mg, 44%). R_f = 0.34 (hexane/EtOAc, 10/1). Obtained and isolated as a ca. 1/>20 mixture of *E/Z* diastereoisomers. Data for the major (*Z*) diastereoisomer: **^{1}H NMR** (300 MHz, $CDCl_3$): δ (ppm) = 8.24 (d, *J* = 8.1 Hz, 1H), 7.31–7.19 (m, 2H), 7.15–7.05 (m, 2H), 6.97 (d, *J* = 12.0 Hz, 1H), 6.78 (d, *J* = 12.0 Hz, 1H), 6.70 (t, *J* = 7.6 Hz, 2H), 6.32–6.27 (m, 1H), 3.35 (s, 3H), 3.18 (s, 3H), 2.26 (s, 1H), 1.69–1.58 (m, 2H), 0.86–0.71 (m, 2H), 0.71–0.58 (m, 2H), 0.58–0.45 (m, 2H), 0.45–0.35 (m, 2H). **^{13}C NMR** (75.4 MHz, $CDCl_3$): δ (ppm) = 159.6, 137.9, 137.4, 134.4, 132.6, 129.4, 127.0, 122.11, 122.09, 121.9, 121.5, 119.0, 115.6, 115.2, 111.8, 109.6, 83.5, 72.5, 54.6, 43.1, 29.9, 20.7, 20.8, 3.3, 2.6. **EI-LRMS** *m/z* (%): 381 (M^+, 48), 352 (81), 262 (100). **HRMS** (ESI-TOF) *m/z*: calcd for $C_{27}H_{28}NO^+$ $[M+H]^+$ 382.2165; found 382.2166.

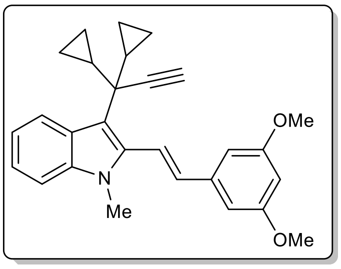

(*E*)-3-(1,1-Dicyclopropylprop-2-yn-1-yl)-2-(3,5-dimethoxystyryl)-1-methyl-1*H*-indole ((*E*)-3d): Following the general procedure E with (***E***)-**S1d** (879 mg, 3 mmol) and 1,1-dicyclopropyl-3-(trimethylsilyl)prop-2-yn-1-ol (750 mg, 3.6 mmol), the crude product was purified by column chromatography (hexane/EtOAc, 10/1) affording pure (***E***)-**3d** as a light yellow oil (986 mg, 80%). R_f = 0.25 (hexane/EtOAc, 10/1). **^{1}H NMR** (300 MHz, $CDCl_3$): δ (ppm) = 8.26 (ad, *J* = 8.2 Hz, 1H), 7.78 (d, *J* = 16.7 Hz, 1H), 7.38 (ad, *J* = 8.2 Hz, 1H), 7.30 (at, *J* = 7.5 Hz, 1H), 7.17 (at, *J* = 7.5 Hz, 1H), 6.74 (ad, *J* = 1.8 Hz, 2H), 6.63 (d, *J* = 16.7 Hz, 1H), 6.51 (as, 1H), 3.90 (s, 6H), 3.82 (s, 3H), 2.37 (s, 1H), 1.89–1.57 (m, 2H), 0.93–0.77 (m, 2H), 0.77–0.59 (m, 4H), 0.59–0.32 (m, 2H). **^{13}C NMR** (75.4 MHz, $CDCl_3$): δ (ppm) = 161.2, 139.2, 138.2, 134.4, 126.8, 122.2, 121.8, 121.4, 119.2, 116.7, 109.7, 104.6, 100.1, 84.3, 72.5, 55.5, 43.1, 31.5, 21.2, 3.7, 2.7. **EI-LRMS** *m/z* (%): 411 (M^+, 6), 264 (100), 234 (64). **HRMS** (ESI-TOF) *m/z*: calcd for $C_{28}H_{30}NO_2^+$ $[M+H]^+$ 412.2271; found 412.2273.

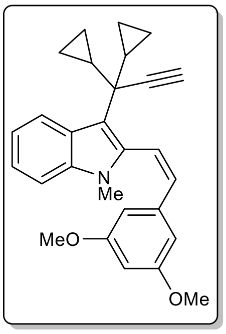

(*Z*)-3-(1,1-Dicyclopropylprop-2-yn-1-yl)-2-(3,5-dimethoxystyryl)-1-methyl-1*H*-indole ((*Z*)-3d): Following the general procedure E with a 8/1 mixture of (***Z***)/(***E***)-**S1d** (879 mg, 3 mmol) and 1,1-dicyclopropyl-3-(trimethylsilyl)prop-2-yn-1-ol (750 mg, 3.6 mmol), the crude product was purified by column chromatography (hexane/EtOAc, 10/1) affording pure **3d** as a light yellow oil (961 mg, 78%). R_f = 0.28 (hexane/EtOAc, 10/1). Obtained and isolated as a ca. 8/1 mixture of *Z/E* diastereoisomers. Data for the major (*Z*) diastereoisomer: **^{1}H NMR** (300 MHz, $CDCl_3$): δ (ppm) = 8.28 (d, *J* = 8.1 Hz, 1H), 7.32–7.23 (m, 2H), 7.20–7.10 (m, 1H), 7.03 (d, *J* = 11.9 Hz, 1H), 6.78 (d, *J* = 12.0 Hz, 1H), 6.34 (t, *J* = 2.2 Hz, 1H), 6.12 (d, *J* = 2.2 Hz, 2H), 3.40 (s, 3H), 3.35 (s, 6H), 2.31 (s, 1H), 1.75–1.63 (m, 2H), 0.89–0.78 (m, 2H), 0.77–0.64 (m, 2H), 0.64–0.53 (m, 2H), 0.53–0.40 (m, 2H). **^{13}C NMR** (75.4 MHz, $CDCl_3$): δ (ppm) = 160.6, 138.4, 137.3, 134.5, 132.4, 126.9, 122.3, 122.0, 121.5, 118.9, 115.6, 109.4, 106.2, 101.1, 83.4, 72.5, 54.8, 43.1, 29.8, 21.2, 3.3, 2.5. **EI-LRMS** *m/z* (%): 411 (M^+, 20), 382 (21), 292 (100). **HRMS** (ESI-TOF) *m/z*: calcd for $C_{28}H_{30}NO_2^+$ $[M+H]^+$ 412.2271; found 412.2272.

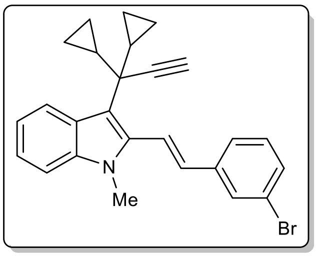

(*E*)-2-(3-Bromostyryl)-3-(1,1-dicyclopropylprop-2-yn-1-yl)-1-methyl-1*H*-indole ((*E*)-3e): Following the general procedure E with (***E***)-**S1e** (936 mg, 3 mmol) and 1,1-dicyclopropyl-3-(trimethylsilyl)prop-2-yn-1-ol (750 mg, 3.6 mmol), the crude product was purified by column chromatography (hexane/EtOAc, 10/1) affording pure (***E***)-**3e** as a light yellow oil (774 mg, 60%). R_f = 0.28 (hexane/EtOAc, 10/1). Obtained and isolated as a ca. 18/1 mixture of *E/Z* diastereoisomers. Data for the major (*E*) diastereoisomer: **^{1}H NMR** (300 MHz, $CDCl_3$): *δ* (ppm) = 8.23 (d, *J* = 8.2 Hz, 1H), 7.80 (d, *J* = 16.7 Hz, 1H), 7.74–7.66 (m, 1H), 7.50–7.44 (m, 2H), 7.36 (d, *J* = 8.1 Hz, 1H), 7.34–7.24 (m, 2H), 7.21–7.10 (m, 1H), 6.59 (d, *J* = 16.7 Hz, 1H), 3.79 (s, 3H), 2.35 (s, 1H), 1.71–1.59 (m, 2H), 0.89–0.76 (m, 2H), 0.69–0.55 (m, 4H), 0.55–0.40 (m, 2H). **^{13}C NMR** (75.4 MHz, $CDCl_3$): *δ* (ppm) = 139.4, 138.3, 134.1, 132.8, 130.8, 130.4, 129.5, 126.8, 124.9, 123.2, 122.4, 122.3, 122.1, 119.4, 117.2, 109.8, 84.3, 72.7, 43.2, 31.6, 21.3, 3.7, 2.7. **EI-LRMS** *m/z* (%): 431 (M^+, 64), 230 (78), 218 (100). **HRMS** (ESI-TOF) *m/z*: calcd for $C_{26}H_{24}BrNNa^+$ $[M+Na]^+$ 452.0984; found 452.0987.

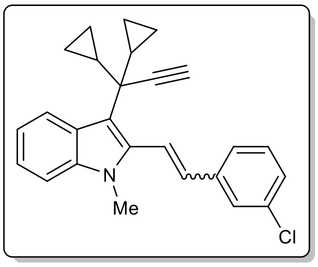

2-(3-Chlorostyryl)-3-(1,1-dicyclopropylprop-2-yn-1-yl)-1-methyl-1*H*-indole (3f): Following the general procedure E with **S1f** (801 mg, 3 mmol) and 1,1-dicyclopropyl-3-(trimethylsilyl)prop-2-yn-1-ol (750 mg, 3.6 mmol), the crude product was purified by column chromatography (hexane/EtOAc, 10/1) affording pure **3f** as a light yellow solid (936 mg, 81%). R_f = 0.35 (hexane/EtOAc, 10/1); m.p. = 124–126 °C. Obtained and isolated as a ca. 1/2 mixture of *E/Z* diastereoisomers. Data for both diastereoisomers: **^{1}H NMR** (300 MHz, $CDCl_3$): *δ* (ppm) = 8.34 (ad, *J* = 8.1 Hz, 1H), 8.28 (ad, *J* = 8.2 Hz, 1H), 7.85 (d, *J* = 16.7 Hz, 1H), 7.57 (as, 1H), 7.49–7.27 (m, 6H), 7.26–7.14 (m, 5H), 7.12–7.04 (m, 2H), 6.81 (as, 1H), 6.78 (d, *J* = 11.9 Hz, 1H), 6.64 (d, *J* = 16.7 Hz, 1H), 3.82 (s, 3H), 3.39 (s, 3H), 2.39 (s, 1H), 2.33 (s, 1H), 1.77–1.72 (m, 4H), 0.92–0.77 (m, 4H), 0.77–0.40 (m, 12H). **^{13}C NMR** (75.4 MHz, $CDCl_3$): *δ* (ppm) = 139.1, 138.4, 138.3, 137.4, 134.9, 134.3, 134.1, 132.9, 132.8, 131.9, 130.2, 129.9, 129.5, 127.9, 127.8, 126.9, 126.8, 126.4, 126.0, 124.5, 123.5, 122.3, 122.2, 122.0, 121.6, 119.3, 119.1, 117.1, 115.9, 109.8, 109.7, 84.3, 83.3, 72.7, 72.6, 43.2, 43.0, 31.6, 29.9, 21.3, 20.7, 3.7, 3.3, 2.7, 2.6. **EI-LRMS** *m/z* (%): 385 (M^+, 62), 358 (43), 356 (100). **HRMS** (ESI-TOF) *m/z*: calcd for $C_{26}H_{25}ClN^+$ $[M+H]^+$ 386.1670; found 386.1675.

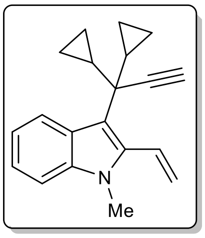

3-(1,1-Dicyclopropylprop-2-yn-1-yl)-1-methyl-2-vinyl-1*H*-indole (3g): Following the general procedure E with **S1g** (471 mg, 3 mmol) and 1,1-dicyclopropyl-3-(trimethylsilyl)prop-2-yn-1-ol (750 mg, 3.6 mmol), the crude product was purified by column chromatography (hexane/EtOAc, 7/1) affording pure **3g** as a light white solid (487 mg, 59%). R_f = 0.50 (hexane/EtOAc, 7/1); m.p. = 120–122 °C. **^{1}H NMR** (300 MHz, $CDCl_3$): *δ* (ppm) = 8.40 (ad, *J* = 8.1 Hz, 1H), 7.50–7.38 (m, 3H), 7.34–7.24 (m, 1H), 5.81 (dd, *J* = 11.6, 1.8 Hz, 1H), 5.53 (dd, *J* = 18.0, 1.8 Hz, 1H), 3.83 (s, 3H), 2.43 (s, 1H), 1.86–1.71 (m, 2H), 1.04–0.85 (m, 2H), 0.85–0.70 (m, 4H), 0.70–0.56 (m, 2H). **^{13}C NMR** (75.4 MHz, $CDCl_3$): *δ* (ppm) = 137.7, 134.7, 129.5, 126.7, 122.2, 121.6, 121.2, 119.0, 115.2, 109.6, 84.1, 72.4, 42.9, 31.0, 21.1, 3.5, 2.7. **EI-LRMS** *m/z* (%): 275 (M^+, 100), 246 (44), 220 (50). **HRMS** (ESI-TOF) *m/z*: calcd for $C_{20}H_{22}N^+$ $[M+H]^+$ 276.1747; found 276.1749.

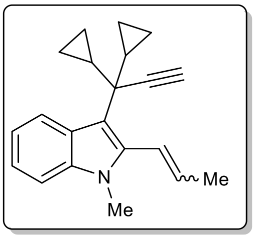

3-(1,1-Dicyclopropylprop-2-yn-1-yl)-1-methyl-2-(prop-1-en-1-yl)-1*H*-indole (3h): Following the general procedure E with **S1h** (513 mg, 3 mmol) and 1,1-dicyclopropyl-3-(trimethylsilyl)prop-2-yn-1-ol (750 mg, 3.6 mmol), the crude product was purified by column chromatography (hexane/EtOAc, 10/1) affording pure **3h** as a light yellow oil (694 mg, 80%). R_f = 0.37 (hexane/EtOAc, 10/1). Obtained and isolated as a ca. 1.4/1 mixture of *E/Z* diastereoisomers. Data for both diastereoisomers: **^{1}H NMR** (300 MHz, $CDCl_3$): δ (ppm) = 8.26 (ad, *J* = 8.2 Hz, 1H), 8.22 (ad, *J* = 8.2 Hz, 1H), 7.38–7.28 (m, 2H), 7.28–7.20 (m, 2H), 7.18–7.05 (m, 2H), 6.84 (dd, *J* = 16.2, 1.7 Hz, 1H), 6.70 (dd, *J* = 11.0, 1.7 Hz, 1H), 6.09 (dq, *J* = 11.0, 6.8 Hz, 1H), 5.82 (dq, *J* = 16.1, 6.6 Hz, 1H), 3.69 (s, 3H), 3.62 (s, 3H), 2.29 (s, 1H), 2.27 (s, 1H), 2.00 (dd, *J* = 6.6, 1.7 Hz, 3H), 1.63 (dd, *J* = 6.8, 1.7 Hz, 3H), 1.61–1.54 (m, 4H), 0.88–0.70 (m, 4H), 0.67–0.49 (m, 8H), 0.49–0.36 (m, 4H). **^{13}C NMR** (75.4 MHz, $CDCl_3$): δ (ppm) = 137.1, 136.9, 134.6, 132.8, 132.4, 132.2, 127.0, 126.8, 123.24, 123.15, 121.9, 121.0, 120.9, 118.7, 118.6, 114.4, 114.2, 109.3, 109.2, 84.0, 83.6, 72.1, 72.0, 42.8, 42.7, 30.5, 29.5, 21.1, 20.7, 18.7, 15.1, 3.4, 3.0, 2.5, 2.4. **EI-LRMS** *m/z* (%): 289 (M^+, 32), 248 (100), 233 (28). **HRMS** (ESI-TOF) *m/z*: calcd for $C_{21}H_{24}N^+$ $[M+H]^+$ 290.1903; found 290.1907.

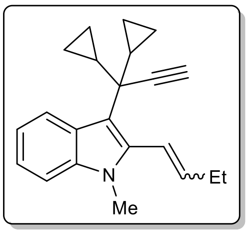

2-(But-1-en-1-yl)-3-(1,1-dicyclopropylprop-2-yn-1-yl)-1-methyl-1*H*-indole (3i): Following the general procedure E with **S1i** (555 mg, 3 mmol) and 1,1-dicyclopropyl-3-(trimethylsilyl)prop-2-yn-1-ol (750 mg, 3.6 mmol), the crude product was purified by column chromatography (hexane/EtOAc, 10/1) affording pure **3i** as a light orange oil (627 mg, 69%). R_f = 0.43 (hexane/EtOAc, 10/1). Obtained and isolated as a ca. 1.7/1 mixture of *E/Z* diastereoisomers. Data for both diastereoisomers: **^{1}H NMR** (300 MHz, $CDCl_3$): δ (ppm) = 8.23 (at, *J* = 7.9 Hz, 2H), 7.35–7.29 (m, 2H), 7.28–7.20 (m, 2H), 7.18–7.08 (m, 2H), 6.84 (ad, *J* = 16.3 Hz, 1H), 6.65 (d, *J* = 11.1 Hz, 1H), 6.02–5.79 (m, 2H), 3.70 (s, 3H), 3.63 (s, 3H), 2.42–2.31 (m, 1H), 2.29 (s, 1H), 2.26 (s, 1H), 2.07–1.95 (m, 2H), 1.68–1.54 (m, 4H), 1.19 (t, *J* = 7.5 Hz, 3H), 1.03 (t, *J* = 7.5 Hz, 3H), 0.85–0.71 (m, 5H), 0.65–0.51 (m, 7H), 0.50–0.39 (m, 5H). **^{13}C NMR** (75.4 MHz, $CDCl_3$): δ (ppm) = 139.8, 139.6, 137.4, 137.1, 135.0, 132.9, 127.1, 126.9, 122.08, 122.05, 121.5, 121.2, 121.1, 118.9, 118.8, 114.8, 114.4, 109.5, 109.4, 84.3, 83.8, 72.2, 72.1, 43.0, 42.9, 30.9, 30.1, 26.5, 23.0, 21.1, 20.8, 13.4, 13.3, 3.5, 3.1, 2.6, 2.5. **EI-LRMS** *m/z* (%): 303 (M^+, 21), 262 (100), 232 (22). **HRMS** (ESI-TOF) *m/z*: calcd for $C_{22}H_{26}N^+$ $[M+H]^+$ 304.2060; found 304.2066.

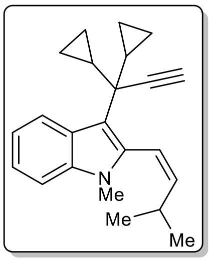

(*Z*)-3-(1,1-Dicyclopropylprop-2-yn-1-yl)-1-methyl-2-(3-methylbut-1-en-1-yl)-1*H*-indole ((*Z*)-3j): Following the general procedure E with a 8/1 mixture of (***Z***)/(***E***)-**S1j** (597 mg, 3 mmol) and 1,1-dicyclopropyl-3-(trimethylsilyl)prop-2-yn-1-ol (750 mg, 3.6 mmol), the crude product was purified by column chromatography (hexane/EtOAc, 10/1) affording pure **3j** as a light yellow oil (592 mg, 62%). R_f = 0.32 (hexane/EtOAc, 10/1). Obtained and isolated as a ca. 8/1 mixture of *Z/E* diastereoisomers. Data for the major (*Z*) diastereoisomer: **^{1}H NMR** (300 MHz, $CDCl_3$): δ (ppm) = 8.41 (ad, *J* = 8.1 Hz, 1H), 7.48 (ad, *J* = 8.0 Hz, 1H), 7.41 (at, *J* = 7.4 Hz, 1H), 7.32 (at, *J* = 7.4 Hz, 1H), 6.74 (ad, *J* = 11.0 Hz, 1H), 5.92 (at, *J* = 11.0 Hz, 1H), 3.80 (s, 3H), 2.62–2.48 (m, 1H), 2.39 (s, 1H), 1.88–1.69 (m, 2H), 1.20 (d, *J* = 6.5 Hz, 6H), 1.04–0.94 (m, 2H), 0.92–0.81 (m, 2H), 0.81–0.69 (m, 2H), 0.69–0.59 (m, 2H). **^{13}C NMR** (75.4 MHz, $CDCl_3$): δ (ppm) = 144.5, 137.1, 132.5, 127.0, 122.1, 121.1, 119.5, 118.7, 115.4, 109.4, 83.2, 72.4, 43.0, 30.4, 28.6, 22.3, 21.1, 20.8, 3.1, 2.7. **EI-LRMS** *m/z*

(%): 317 (M^+, 62), 346 (63), 233 (100). **HRMS** (ESI-TOF) *m/z*: calcd for $C_{23}H_{28}N^+$ $[M+H]^+$ 318.2216; found 318.2228.

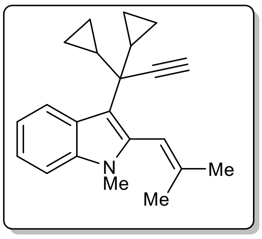

3-(1,1-Dicyclopropylprop-2-yn-1-yl)-1-methyl-2-(2-methylprop-1-en-1-yl)-1*H*-indole (3k): Following the general procedure E with **S1k** (555 mg, 3 mmol) and 1,1-dicyclopropyl-3-(trimethylsilyl)prop-2-yn-1-ol (750 mg, 3.6 mmol), the crude product was purified by column chromatography (hexane/EtOAc, 10/1) affording pure **3k** as a light orange oil (722 mg, 74%). R_f = 0.40 (hexane/EtOAc, 10/1). **^{1}H NMR** (300 MHz, $CDCl_3$): δ (ppm) = 8.22 (d, *J* = 8.1 Hz, 1H), 7.31 (d, *J* = 8.1 Hz, 1H), 7.21 (t, *J* = 7.5 Hz, 1H), 7.18–7.08 (m, 1H), 6.38 (s, 1H), 3.58 (s, 3H), 2.24 (s, 1H), 1.97 (s, 3H), 1.58 (s, 3H), 1.63–1.51 (m, 2H), 0.79–0.68 (m, 2H), 0.67–0.46 (m, 4H), 0.46–0.33 (m, 2H). **^{13}C NMR** (75.4 MHz, $CDCl_3$): δ (ppm) = 140.7, 136.9, 134.1, 127.0, 122.0, 120.9, 118.7, 118.2, 114.5, 109.3, 83.9, 71.9, 42.9, 29.8, 25.1, 20.2, 3.1, 2.3. **EI-LRMS** *m/z* (%): 303 (M^+, 30), 263 (22), 262 (100). **HRMS** (ESI-TOF) *m/z*: calcd for $C_{22}H_{25}NNa^+$ $[M+Na]^+$ 326.1879; found 326.1881.

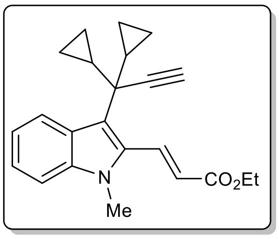

Ethyl (*E*)-3-(3-(1,1-dicyclopropylprop-2-yn-1-yl)-1-methyl-1*H*-indol-2-yl)acrylate (S4): Following the general procedure E with (***E***)-**S1l** (687 mg, 3 mmol) and 1,1-dicyclopropyl-3-(trimethylsilyl)prop-2-yn-1-ol (750 mg, 3.6 mmol), the crude product was purified by column chromatography (hexane/EtOAc, 10/1) affording pure **S4** as a light orange oil (843 mg, 81%). R_f = 0.25 (hexane/EtOAc, 10/1). **^{1}H NMR** (300 MHz, $CDCl_3$): δ (ppm) = 8.76 (d, *J* = 16.4 Hz, 1H), 8.31 (d, *J* = 8.3 Hz, 1H), 7.40–7.28 (m, 2H), 7.26–7.06 (m, 1H), 6.12 (d, *J* = 16.4 Hz, 1H), 4.37 (q, *J* = 7.1 Hz, 2H), 3.79 (s, 3H), 2.43 (s, 1H), 1.74–1.55 (m, 2H), 1.44 (t, *J* = 7.1 Hz, 3H), 1.01–0.83 (m, 2H), 0.78–0.62 (m, 4H), 0.62–0.46 (m, 2H). **^{13}C NMR** (75.4 MHz, $CDCl_3$): δ (ppm) = 166.6, 139.2, 136.4, 131.8, 126.3, 123.3, 122.8, 121.4, 121.0, 119.6, 109.9, 83.4, 73.2, 60.6, 43.3, 32.0, 21.3, 14.4, 3.8, 2.8. **HRMS** (ESI-TOF) *m/z*: calcd for $C_{23}H_{26}NO_2^+$ $[M+H]^+$ 348.1958; found 348.1969.

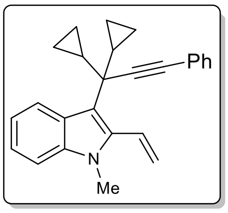

3-(1,1-Dicyclopropyl-3-phenylprop-2-yn-1-yl)-1-methyl-2-vinyl-1*H*-indole (S9a): Following the general procedure E with **S1g** (471 mg, 3 mmol) and 1,1-dicyclopropyl-3-phenylprop-2-yn-1-ol (764 mg, 3.6 mmol), the crude product was purified by column chromatography (hexane/EtOAc, 10/1) affording pure **S9a** as a orange oil (558 mg, 53%). R_f = 0.30 (hexane/EtOAc, 10/1). **^{1}H NMR** (300 MHz, $CDCl_3$): δ (ppm) = 8.29 (d, *J* = 8.1 Hz, 1H), 7.49–7.44 (m, 2H), 7.39–7.31 (m, 4H), 7.31–7.22 (m, 1H), 7.20–7.10 (m, 1H), 5.68 (dd, *J* = 11.6, 1.7 Hz, 1H), 5.42 (dd, *J* = 18.0, 1.7 Hz, 1H), 3.76 (s, 3H), 1.93–1.63 (m, 2H), 1.04–0.77 (m, 2H), 0.77–0.59 (m, 2H), 0.59–0.32 (m, 2H).**^{13}C NMR** (75.4 MHz, $CDCl_3$): δ (ppm) = 137.8, 134.9, 131.6, 129.7, 128.3 , 127.8, 126.9, 124.0, 122.3, 121.6, 121.1, 119.1, 116.2, 109.7, 90.5, 84.6, 43.8, 31.2, 21.6, 3.6, 2.6. **EI-LRMS** *m/z* (%): 351 (M^+, 35), 195 (21), 194 (100), 182 (26). **HRMS** (ESI-TOF) *m/z*: calcd for $C_{26}H_{26}N^+$ $[M+H]^+$ 352.2060; found 352.2053.

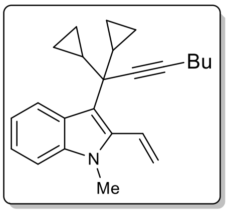

3-(1,1-Dicyclopropylhept-2-yn-1-yl)-1-methyl-2-vinyl-1*H*-indole (S9b): Following the general procedure E with **S1g** (471 mg, 3 mmol) and 1,1-dicyclopropylhept-2-yn-1-ol (691 mg, 3.6 mmol), the crude product was purified by column chromatography (hexane/EtOAc, 10/1) affording pure **S9b** as a yellow oil (536 mg, 54%). R_f = 0.28 (hexane/EtOAc, 10/1). **^{1}H NMR** (300 MHz, $CDCl_3$): δ (ppm) = 8.30 (d, *J* = 8.2 Hz, 1H), 7.45–7.33 (m, 2H), 7.32–7.26 (m, 1H), 7.23–7.13 (m, 1H), 5.71 (d, *J* = 11.6 Hz, 1H), 5.42 (d, *J* = 18.1 Hz, 1H), 3.78 (s, 3H), 2.33 (t, *J* = 6.8 Hz, 2H), 1.72–1.46 (m, 6H), 1.04 (t, *J* = 7.1 Hz, 3H), 0.89–0.75 (m, 2H), 0.69–0.54 (m, 4H), 0.54–0.43 (m, 2H). **^{13}C NMR** (75.4 MHz, $CDCl_3$): δ (ppm) = 137.8, 134.5, 129.8, 126.9, 122.5, 121.5, 120.5, 118.9, 117.2, 109.5, 84.4, 79.6, 43.2, 31.1, 22.2, 21.6, 18.5, 13.8, 3.4, 2.5. **EI-LRMS** *m/z* (%): 331 (M^+, 20), 291 (22), 290 (100), 246 (24). **HRMS** (ESI-TOF) *m/z*: calcd for $C_{24}H_{30}N^+$ $[M+H]^+$ 332.2373; found 332.2369.

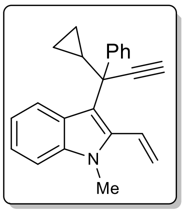

3-(1-Cyclopropyl-1-phenylprop-2-yn-1-yl)-1-methyl-2-vinyl-1*H*-indole (S9c): Following the general procedure E with **S1g** (471 mg, 3 mmol) and 1-cyclopropyl-1-phenyl-3-(trimethylsilyl)prop-2-yn-1-ol (879 mg, 3.6 mmol), the crude product was purified by column chromatography (hexane/EtOAc, 10/1) affording **S9c**, which was isolated along with an unidentified impurity, as a yellow oil (410 mg, 44%). R_f = 0.32 (hexane/EtOAc, 10/1). **^{1}H NMR** (300 MHz, $CDCl_3$): δ (ppm) = 7.76–7.63 (m, 3H), 7.44–7.36 (m, 3H), 7.36–7.26 (m, 2H), 7.17–7.03 (m,2H), 5.64 (d, *J* = 11.7 Hz, 1H), 5.46 (d, *J* = 18.0 Hz, 1H), 3.83 (s, 3H), 2.59 (s, 1H), 1.98–1.84 (m, 1H), 1.12–1.02 (m, 1H), 1.01–0.89 (m, 1H), 0.89–0.77 (m, 1H), 0.77–0.68 (m, 1H). **^{13}C NMR** (75.4 MHz, $CDCl_3$): δ (ppm) = 146.7, 137.9, 135.4, 132.8, 128.6, 128.0, 127.2, 126.4, 121.8, 121.7, 120.6, 116.5, 119.3, 109.5, 84.9, 73.7, 47.2, 31.2, 22.1, 5.8, 4.3. **EI-LRMS** *m/z* (%): 311 (M^+, 100), 282 (65), 267 (59), 133 (47). **HRMS** (ESI-TOF) *m/z*: calcd for $C_{23}H_{22}N^+$ $[M+H]^+$ 312.1747; found 312.1746.

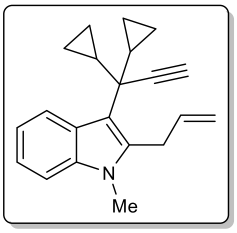

2-Allyl-3-(1,1-dicyclopropylprop-2-yn-1-yl)-1-methyl-1*H*-indole (5): Following the general procedure E with **S2** (513 mg, 3 mmol) and 1,1-dicyclopropyl-3-(trimethylsilyl)prop-2-yn-1-ol (750 mg, 3.6 mmol), the crude product was purified by column chromatography (hexane/EtOAc, 10/1) affording pure **5** as a light yellow oil (529 mg, 61%). R_f = 0.45 (hexane/EtOAc, 10/1). **^{1}H NMR** (300 MHz, $CDCl_3$): δ (ppm) = 8.16 (d, *J* = 8.1 Hz, 1H), 7.30 (d, *J* = 8.2 Hz, 1H), 7.20 (t, *J* = 7.1 Hz, 1H), 7.09 (t, *J* = 8.0 Hz, 1H), 6.11–5.93 (m, 1H), 5.13 (dd, *J* = 10.2, 1.7 Hz, 1H), 4.98 (dd, *J* = 17.2, 1.7 Hz, 1H), 4.06–4.00 (m, 2H), 3.66 (s, 3H), 2.27 (s, 1H), 1.73–1.60 (m, 2H), 0.86–0.75 (m, 2H), 0.68–0.51 (m, 4H), 0.51–0.39 (m, 2H).**^{13}C NMR** (75.4 MHz, $CDCl_3$): δ (ppm) = 137.0, 136.3, 134.3, 126.8, 121.9, 120.8, 118.7, 116.1, 114.3, 108.9, 84.4, 72.4, 43.0, 30.2, 29.6, 20.9, 3.6, 2.7. **EI-LRMS** *m/z* (%): 289 (M^+, 100), 261 (94), 248 (79). **HRMS** (ESI-TOF) *m/z*: calcd for $C_{21}H_{24}N^+$ $[M+H]^+$ 290.1903; found 290.1906.

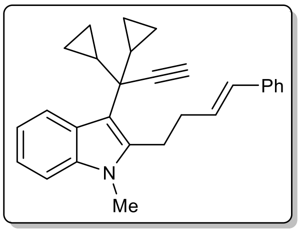

(*E*)-3-(1,1-Dicyclopropylprop-2-yn-1-yl)-1-methyl-2-(4-phenylbut-3-en-1-yl)-1*H*-indole (8a): Following the general procedure E with **S3a** (780 mg, 3 mmol) and 1,1-dicyclopropyl-3-(trimethylsilyl)prop-2-yn-1-ol (750 mg, 3.6 mmol), the crude product was purified by column chromatography (hexane/EtOAc, 10/1) affording pure **8a** as a light yellow oil (580 mg, 51%). R_f = 0.45 (hexane/EtOAc, 10/1). **^{1}H NMR** (300 MHz, $CDCl_3$): δ (ppm) = 8.16 (ad, *J* = 8.2 Hz, 1H), 7.46–7.19 (m, 7H), 7.17–7.06 (m, 1H), 6.55 (d, *J* = 15.9 Hz, 1H), 6.46–6.30 (m, 1H), 3.76 (s, 3H), 3.46–3.31 (m, 2H), 2.69–

2.54 (m, 2H), 2.32 (s, 1H), 1.85–1.69 (m, 2H), 0.92–0.81 (m, 2H), 0.69–0.53 (m, 4H), 0.56–0.45 (m, 2H). **^{13}C NMR** (75.4 MHz, $CDCl_3$): *δ* (ppm) = 137.6, 136.9, 136.8, 130.4, 129.7, 128.7, 127.3, 126.8, 126.1, 121.8, 120.7, 118.8, 113.3, 109.0, 84.2, 72.3, 43.1, 34.4, 29.6, 25.5, 21.0, 3.6, 2.6. **EI-LRMS** *m/z* (%): 379 (M^+, 26), 262 (65), 260 (100). **HRMS** (ESI-TOF) *m/z*: calcd for $C_{28}H_{30}N^+$ $[M+H]^+$ 380.2373; found 380.2376.

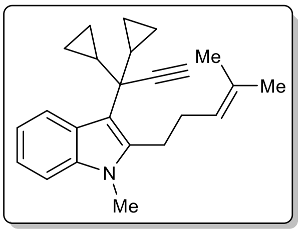

3-(1,1-Dicyclopropylprop-2-yn-1-yl)-1-methyl-2-(4-methylpent-3-en-1-yl)-1*H*-indole (8b): Following the general procedure E with **S3b** (639 mg, 3 mmol) and 1,1-dicyclopropyl-3-(trimethylsilyl)prop-2-yn-1-ol (750 mg, 3.6 mmol), the crude product was purified by column chromatography (hexane/EtOAc, 10/1) affording pure **8b** as a light orange oil (734 mg, 74%). R_f = 0.43 (hexane/EtOAc, 10/1). **^{1}H NMR** (300 MHz, $CDCl_3$): *δ* (ppm) = 8.12 (d, *J* = 8.1 Hz, 1H), 7.30 (d, *J* = 7.5 Hz, 1H), 7.19 (t, *J* = 7.5 Hz, 1H), 7.08 (t, *J* = 8.1 Hz, 1H), 5.26 (t, *J* = 7.1 Hz, 1H), 3.72 (s, 3H), 3.26–3.11 (m, 2H), 2.43–2.28 (m, 2H), 2.27 (s, 1H), 1.77 (s, 3H), 1.76–1.70 (m, 2H), 1.67 (s, 3H), 0.87–0.76 (m, 2H), 0.66–0.52 (m, 4H), 0.50–0.34 (m, 2H). **^{13}C NMR** (75.4 MHz, $CDCl_3$): *δ* (ppm) = 137.2, 136.8, 132.0, 126.7, 123.8, 121.7, 120.5, 118.7, 112.9, 108.9, 84.1, 72.1, 43.0, 29.7, 29.3, 25.9, 25.8, 20.9, 17.9, 3.5, 2.6. **EI-LRMS** *m/z* (%): 331 (M^+, 100), 262 (48), 234 (83). **HRMS** (ESI-TOF) *m/z*: calcd for $C_{24}H_{30}N^+$ $[M+H]^+$ 332.2373; found 332.2371.

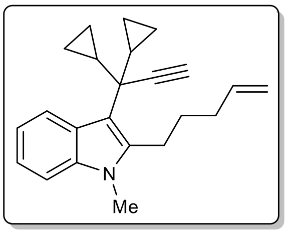

3-(1,1-Dicyclopropylprop-2-yn-1-yl)-1-methyl-2-(pent-4-en-1-yl)-1*H*-indole (8c): Following the general procedure E with **S3c** (597 mg, 3 mmol) and 1,1-dicyclopropyl-3-(trimethylsilyl)prop-2-yn-1-ol (750 mg, 3.6 mmol), the crude product was purified by column chromatography (hexane/EtOAc, 10/1) affording **8c** as a light yellow oil (599 mg, 63%). R_f = 0.42 (hexane/EtOAc, 10/1). Slightly contaminated with an unidentified product. **^{1}H NMR** (300 MHz, $CDCl_3$): *δ* (ppm) = 8.13 (ad, *J* = 8.1 Hz, 1H), 7.31 (d, *J* = 8.1 Hz, 1H), 7.20 (at, *J* = 7.3 Hz, 1H), 7.09 (at, *J* = 7.3 Hz, 1H), 6.04–5.79 (m, 1H), 5.27–4.93 (m, 2H), 3.70 (s, 3H), 3.25–3.12 (m, 2H), 2.28 (s, 1H), 2.27–2.21 (m, 2H). 1.84–1.66 (m, 4H), 0.89–0.77 (m, 2H), 0.69–0.51 (m, 4H), 0.51–0.42 (m, 2H). **^{13}C NMR** (75.4 MHz, $CDCl_3$): *δ* (ppm) = 138.3, 137.6, 136.8, 126.8, 121.7, 120.6, 118.7, 115.3, 112.9, 108.9, 84.3, 72.0, 43.1, 34.2, 30.1, 29.5, 25.4, 21.0, 3.6, 2.6. **HRMS** (ESI-TOF) *m/z*: calcd for $C_{23}H_{28}N^+$ $[M+H]^+$ 318.2216; found 318.2220.

Synthesis of 3-propargyl indole 3l

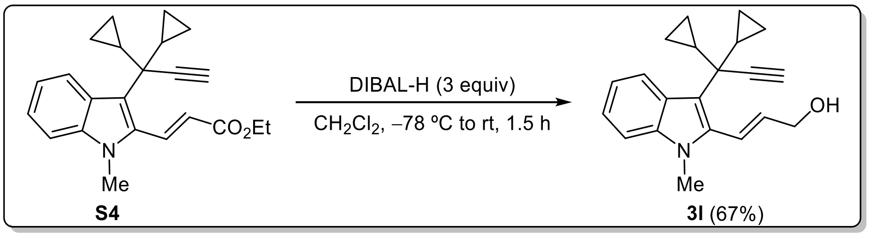

In a round-bottom flask under a nitrogen atmosphere, diisobutylaluminium hydride (DIBAL-H, 6 mmmol, 6 mL of a 1 M solution in CH_2Cl_2) was added dropwise to a solution of ethyl (*E*)-3-(3-(1,1-dicyclopropylprop-2-yn-1-yl)-1-methyl-1*H*-indol-2-yl)acrylate (**S4**) (670 mg, 2 mmol) in anhydrous dichloromethane (5 mL). Subsequently, the reaction was stirred at rt for 1.5 h. The reaction was quenched by the addition of water. The mixture was extracted with Et_2O (3 × 10 mL), and the combined organic layers were dried over anhydrous Na_2SO_4. The solvent was removed under reduced pressure, and the residue was purified by silica gel column

chromatography using a 3/1 mixture of hexane and EtOAc as eluent to afford (***E***)-**3l** as a light orange oil (445 mg, 73%). R_f = 0.30 (hexane/EtOAc, 5/1).

^{1}H NMR (300 MHz, $CDCl_3$): δ (ppm) = 8.29 (ad, *J* = 8.1 Hz, 1H), 7.41–7.30 (m, 2H), 7.29–7.15 (m, 2H), 5.99 (dt, *J* = 16.3, 5.3 Hz, 1H), 4.45 (ad, *J* = 4.2 Hz, 2H), 3.73 (s, 3H), 2.38 (s, 1H), 2.31 (br s, 1H), 1.73–1.60 (m, 2H), 0.93–0.79 (m, 2H), 0.73–0.60 (m, 4H), 0.60–0.46 (m, 2H). **^{13}C NMR** (75.4 MHz, $CDCl_3$): δ (ppm) = 137.6, 135.2, 133.7, 126.6, 122.8, 122.1, 121.5, 119.0, 115.4, 109.5, 84.1, 72.5, 63.5, 42.9, 31.0, 21.1, 3.5, 2.6. **EI-LRMS** *m/z* (%): 305 (M^+, 26), 264 (100), 234 (64). **HRMS** (ESI-TOF) *m/z*: calcd for $C_{21}H_{24}NO^+$ $[M+H]^+$ 306.1852; found 306.1856.

Intermolecular cyclopropanation reactions: Synthesis and characterization data of 3-(1-cyclopropylvinyl)-1*H*-indole derivatives 2

Optimization studies

As already mentioned in the main manuscript, a study was conducted to optimize the reaction conditions for the tandem 1,2-indole migration–cyclopropanation sequence. Herein, we present the tables summarizing the detailed optimization process for the intermolecular cyclopropanation of 3-(1,1-dicyclopropylprop-2-yn-1-yl)-1-methyl-1*H*-indole (**1a**) with styrene as a model olefin. The study focused primarily on evaluating the role of the gold(I) catalyst, as well as the presence of silver salts, on the efficiency and selectivity of the reaction (Table S1). All the tested gold catalysts and silver salts are commercially available and were purchased from Sigma-Aldrich and BLDpharm:

$[(2,4\text{-}t\text{Bu}_2C_6H_3O)_3]$PAuCl: CAS 915299-24-0
IPrAuNTf$_2$: CAS 951776-24-2
JohnPhosAuNTf$_2$: CAS 1036000-94-8
JohnPhosAu(MeCN)SbF$_6$: CAS 866641-66-9
BrettPhosAuNTf$_2$: CAS 1296269-97-0
BrettPhosAuCl: CAS 1334547-75-9

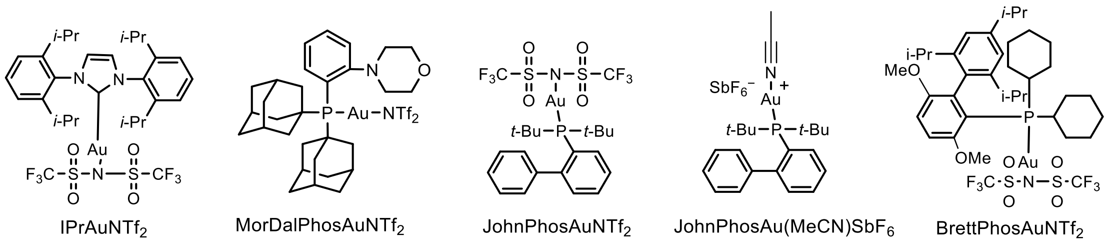

Table S1: Optimization of the reaction conditions for the gold-catalyzed cyclopropanation of **1a**.[a]

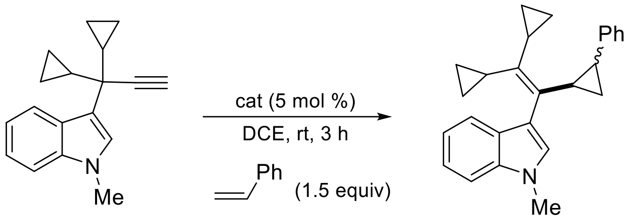

entry	catalyst	yield **2a** (%)[c]	d.r. (*cis/trans*)[d]
1[b]	$(ArO)_3PAuCl/AgNTf_2$	15	1/2
2	$IPrAuNTf_2$	71	1/1
3	$MorDalPhosAuNTf_2$	47	1/1
4	$JohnPhosAuNTf_2$	63	2.5/1
5	$JohnPhosAu(MeCN)SbF_6$	32	1/1.5
6	$BrettPhosAuNTf_2$	83	15/1
7	$BrettPhosAuCl/AgNTf_2$	75	1/11
8	BrettPhosAuCl/AgOTf	47	1/15
9	$BrettPhosAuCl/AgSbF_6$	78	1/15
10	$BrettPhosAuCl/AgBF_4$	65	1/8
11	BrettPhosAuCl/NaBARF	80	9/1
12[e]	$AgNTf_2$	–	–

[a]Reaction conditions: **1a** (0.2 mmol), catalyst (5 mol%) in dichloroethane (2 mL) at rt for 3 h. Complete conversion was observed in all the cases except otherwise established. [b]Ar = 2,4-tBu$_2$C$_6$H$_3$. [c]Determined by ^{1}H NMR analysis using 1,3,5-trimethoxybenzene as internal standard. [d]Determined by ^{1}H NMR analysis of the crude reaction mixture. [e]Almost no conversion was observed.

When a catalyst bearing a phosphite ligand was used, the desired product **2a** was obtained in only 15% yield, as a 1/2 mixture of *cis/trans* diastereoisomers (entry 1). Looking for improving the efficiency of the process, a gold complex featuring NHC ligands was tested, affording indole **2a** in good yield as a ca. 1/1 diastereomeric mixture (entry 2). A similar outcome was observed with $MorDalPhosAuNTf_2$ as catalyst (entry 3). To enhance the selectivity of the tandem 1,2-indole migration / intermolecular cyclopropanation sequence, gold catalysts bearing phosphine-type ligands were evaluated (entries 4–6). The best result was obtained with $BrettPhosAuNTf_2$, providing **2a** exclusively as the *cis* diastereoisomer in good yield (entry 6). Next, we investigated the effect of the presence of silver salts and counterions on the reaction´s diastereoselectivity. Treatment of **1a** and styrene for 3 h with a catalyst generated in situ from BrettPhosAuCl and various silver salts resulted in a switch of selectivity, yielding predominantly the *trans* diastereoisomer of **2a** in all cases (entries 7–10). The crucial role of the silver salt was further demonstrated: when a sodium salt was employed instead of silver, the cyclopropanation of **1a** afforded **2a** in 80% yield with the *cis/trans* ratio over 9/1 (entry 11). Finally, when $AgNTf_2$ alone was used as catalyst, almost no conversion of the starting material was observed (entry 12).

In view of these results, which show that the diastereoselectivity of the tandem 1,2-indole migration–intermolecular cyclopropanation could be reversed by the addition of a silver salt, we undertook

a more detailed investigation to assess the generality of this selectivity switch, by employing other styrene derivatives (Table S2).

Table S2: Study of the diastereoselectivity in the cyclopropanation reactions of **1a,b** with different olefins.[a]

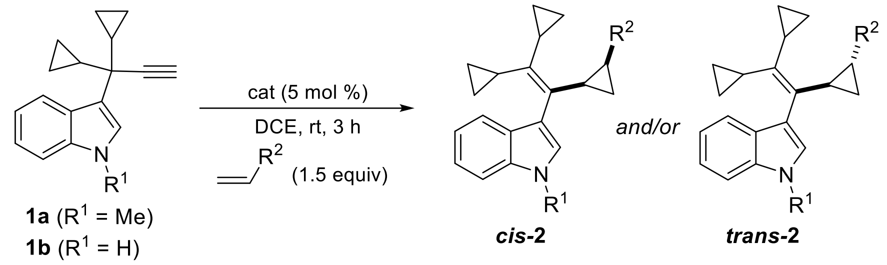

entry	1	catalyst	R^2	2	yield **2** (%)[b]	d.r. (*cis/trans*)[c]
1	**1a**	BrettPhosAuCl/$AgSbF_6$	4-MeC_6H_4	**2b**	84	1.2/1
2	**1a**	BrettPhosAuCl/$AgNTf_2$	4-MeC_6H_4	**2b**	64	1/1.6
3	**1a**	BrettPhosAuCl/$AgSbF_6$	2-MeC_6H_4	**2c**	78	1/4
4	**1a**	BrettPhosAuCl/$AgSbF_6$	4-ClC_6H_4	**2d**	74	1/3
5	**1a**	BrettPhosAuCl/$AgNTf_2$	4-ClC_6H_4	**2d**	79	2/1
6	**1b**	BrettPhosAuCl/$AgSbF_6$	Ph	**2j**	66	1/3
7	**1b**	BrettPhosAuCl/$AgSbF_6$	4-MeC_6H_4	**2k**	64	1/8

[a]Reaction conditions: **1** (0.2 mmol), catalyst (5 mol%) in dichloroethane (2 mL) at rt for 3 h. [b]Determined by ^1H NMR analysis using 1,3,5-trimethoxybenzene as internal standard. [c]Determined by ^1H NMR analysis of the crude reaction mixture.

Thus, we attempted to reproduce the reactivity of model substrate **1a** with other olefins for the diastereoselective synthesis of the *trans*-isomer of the cyclopropane product, analogous to what observed for indole **1a** with styrene (Table S1, entry 9). When **1a** was treated with 4-methylstyrene in the presence of the catalytically active gold complex generated from BrettphosAuCl and $AgSbF_6$, no inversion of diastereoselectivity toward the *trans* isomer was observed, differing significantly from the outcome with styrene. Instead, a ca. 1/1 mixture of *cis*/*trans* diastereoisomers was obtained (entry 1). This outcome indicates that while the Au(I) catalytic system plays a dominant role in controlling the diastereoselectivity, the nature of the olefin also exerts a significant influence. Poor diastereoselectivity was similarly observed when $AgNTf_2$ was used as the silver source (entry 2). When 2-methylstyrene was used, product ***trans*-2c** was obtained with a d.r. of 1/4 (*cis/trans*) (entry 3). Employing a styrene derivative bearing an electron-withdrawing substituent at the *para* position of the aromatic ring led to predominant formation of the *trans* isomer, though with considerably lower selectivity compared to the result with unsbustituted styrene (entry 4). Replacing $AgSbF_6$ with $AgNTf_2$ under otherwise identical conditions reversed the diastereoselectivity, favoring the formation of the *cis* isomer, with a *cis/trans* ratio of 2/1 (entry 5). These results collectively suggest that the presence of a silver salt significantly promotes the formation of the *trans* diastereoisomer of the final cyclopropane **2**. However, the overall diastereoselectivity is highly dependent on the nature of the catalytic system, the counterion, and the specific olefin employed. Finally, when the NH-indole derivative **1b** was reacted with the BrettPhosAuCl/$AgSbF_6$ catalytic system in the presence of two different styrenes, the corresponding 3-(1-cyclopropylvinyl)-1*H*-indole derivatives **2j** and **2k** were obtained as mixtures of diastereoisomers. In both cases, the *trans* isomer was the major product (entries 6 and 7).

Study of the silver effect on changing diastereselectivity obtained.

In order to study the selectivity observed in the presence of silver salts, we have performed additional experiments to clarify this silver effect on the diastereoselectivity. When pure *cis*-**2a** (0.2 mmol) was subjected to reaction conditions using BrettPhosAuCl/$AgNTf_2$ (5 mol%), isomerization to *trans*-**2a** was observed, achieving comparable diastereoselectivities when this catalytic system was used with the parent propargyl indole (Scheme S1).

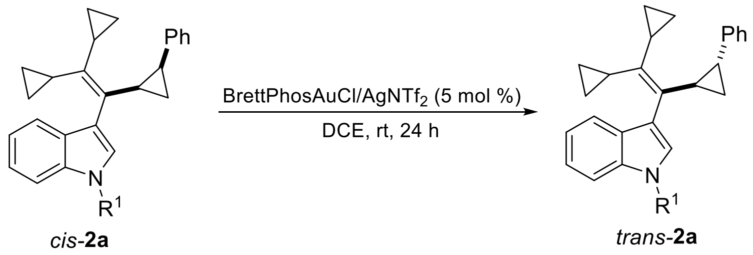

Scheme S1. Isomerization of *cis*-**2a** to *trans*-**2a**. Reaction conditions: *cis*-**2a** (0.2 mmol), catalyst (5 mol%) in dichloroethane (2 mL) at rt for 24 h. Yield and diastereomeric ratio (d.r.) were determined by ^{1}H NMR analysis from the crude reaction mixture using 1,3,5-trimethoxybenzene as internal standard.

As noted in the optimization table S1, when silver salts bearing different counteranions are used as halide scavengers, compound ***trans*-2a** is formed preferentially over ***cis*-2a**. The counteranion has a relatively small effect on the diastereoselectivity obtained. Additional control experiments were also performed. For instance, the addition of silver chloride has no effect in modifying the diastereoselectivity of the reaction when BrettPhosAuNTf_2 was used as the catalyst and ***cis*-2a** is formed as the major product (Scheme S2a), probably due very low solubility of this silver salt (AgCl is very insoluble in organic solvents as well as in water). By pre-forming the cationic gold complex with $AgSbF_6$ as a chloride scavenger and completely removing the silver chloride by filtration over a celite pad, ***cis*-2a** was obtained with similar diastereoselectivities when cationic BrettPhosAuNTf_2 was used as the catalyst (Scheme S2b). The silver counteranion does not have a crucial role in the isomerization. These results demonstrated that the Ag(I) is essential in achieving reversed diastereoselectivities by favoring the isomerization of ***cis*-2a** to ***trans*-2a**.

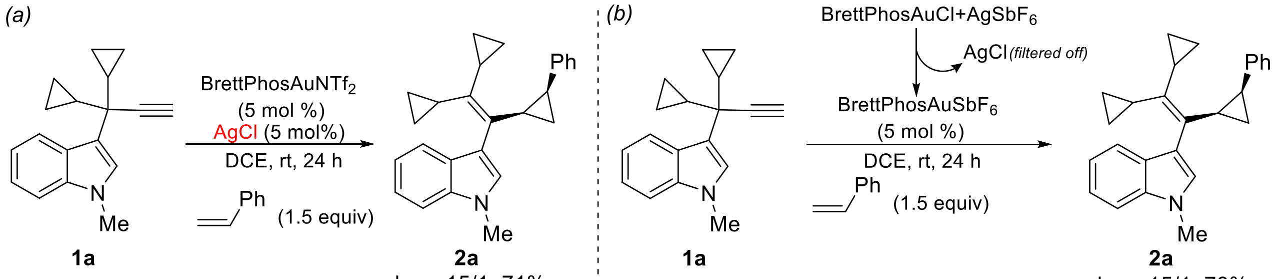

Scheme S2. Control experiments. Reaction conditions: **1a** (0.2 mmol), styrene (0.3 mmol), catalyst (5 mol%) in dichloroethane (2 mL) at rt for 24 h. Yield and diastereomeric ratio (d.r.) were determined by ^{1}H NMR analysis from the crude reaction mixture using 1,3,5-trimethoxybenzene as internal standard.

General procedures F for the synthesis of 3-(1-cyclopropylvinyl)-1H-indole derivatives 2

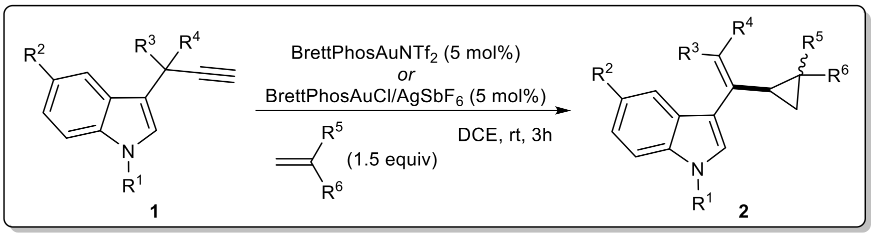

General procedure F1: $BrettPhosAuNTf_2$ (21.6 mg, 0.025 mmol, 0.05 equiv) was dissolved in DCE (0.5 mL) and the resulting solution was stirred at rt for 5 min. A solution of the olefin (0.75 mmol, 1.5 equiv) in DCE (0.5 mL) and a solution of the corresponding 3-propargylindole **1** (0.5 mmol, 1 equiv) in DCE (1 mL, 0.25 M) were then added sequentially. The reaction mixture was stirred at rt for 3h (until complete consumption of the starting material as determined by GC–MS or TLC). The crude mixture was filtered through a short pad of silica gel and celite using a 5/1 mixture of hexane/EtOAc. The solvents were removed under reduced pressure, and the residue was purified by flash column chromatography using mixtures of hexane/EtOAc as eluents to afford the corresponding indole derivatives **2**.

General procedure F2: BrettPhosAuCl (19 mg, 0.025 mmol, 0.05 equiv) and $AgSbF_6$ (8.5 mg, 0.025 mmol, 0.05 equiv) were dissolved in DCE (0.5 mL), and the resulting solution was stirred at rt for 5 min. A solution of the olefin (0.75 mmol, 1.5 equiv) in DCE (0.5 mL) and a solution of the corresponding 3-propargylindole **1** (0.5 mmol, 1 equiv) in DCE (1 mL, 0.25 M) were then added sequentially. The reaction mixture was stirred at rt for 3h (until complete consumption of the starting material as determined by GC–MS or TLC). The crude mixture was filtered through a short pad of silica gel and celite using a 5/1 mixture of hexane/EtOAc. The solvents were removed under reduced pressure, and the residue was purified by flash column chromatography using mixtures of hexane/EtOAc as eluents to afford the corresponding indole derivatives **2**.

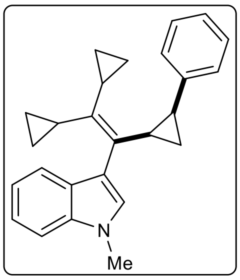

3-(2,2-Dicyclopropyl-1-((1*R,2*S**)-2-phenylcyclopropyl)vinyl)-1-methyl-1*H*-indole (*cis*-2a)**: Following the general procedure F1 with **1a** (124 mg, 0.5 mmol) and styrene (0.75 mmol, 78 mg), the crude product was purified by column chromatography (hexane/EtOAc, 7/1) affording pure **2a** as a yellow oil (127 mg, 72%). R_f = 0.20 (hexane/EtOAc, 5/1). Obtained and isolated as a ca. 16/1 mixture of *cis/trans* diastereoisomers. Data for major isomer: **^{1}H NMR** (300 MHz, $CDCl_3$): δ (ppm) = 7.53 (ad, J = 8.0 Hz, 1H), 7.33 (d, J = 8.0 Hz, 1H), 7.30–7.25 (m, 1H), 7.25–7.18 (m, 3H), 7.16–7.10 (m, 1H), 7.08–7.01 (m, 2H), 6.28 (s, 1H), 3.74 (s, 3H), 2.64–2.51 (m, 1H), 2.46–2.34 (m, 1H), 1.71–1.59 (m, 1H), 1.41–1.32 (m, 1H), 1.24–1.11 (m, 1H), 1.09–1.03 (m, 1H), 0.95–0.78 (m, 2H), 0.77–0.64 (m, 2H), 0.51–0.37 (m, 1H), 0.36–0.22 (m, 1H), 0.18–0.06 (m, 2H). **^{13}C NMR** (75.4 MHz, $CDCl_3$): δ (ppm) = 140.6, 140.5, 136.3, 128.6, 128.5, 128.4, 127.33, 127.30, 125.1, 121.1, 120.9, 118.6, 116.8, 108.8, 32.7, 25.8, 25.0, 14.5, 14.3, 13.9, 7.6, 6.6, 6.0, 5.7. **EI-LRMS** *m/z* (%): 353 (M^+, 21), 207 (100), 249 (77). **HRMS** (ESI-TOF) *m/z*: calcd for $C_{26}H_{28}N^+$ $[M+H]^+$ 354.2216; found 354.2209.

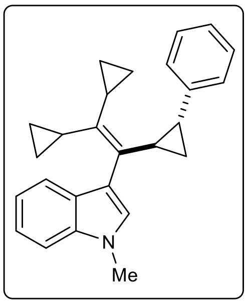

3-(2,2-Dicyclopropyl-1-((1*R,2*R**)-2-phenylcyclopropyl)vinyl)-1-methyl-1*H*-indole (*trans*-2a)**: Following the general procedure F2 with **1a** (124 mg, 0.5 mmol) and styrene (0.75 mmol, 78 mg), the crude product was purified by column chromatography (hexane/EtOAc, 10/1) affording pure **2a** as a orange oil (141 mg, 80%). R_f = 0.30 (hexane/EtOAc, 10/1). Obtained and isolated as a ca. 1/15 mixture of *cis/trans* diastereoisomers. Data for major isomer: **^{1}H NMR** (500 MHz, $CDCl_3$): δ (ppm) = 7.57 (ad, J = 7.8 Hz, 1H), 7.37 (ad, J = 8.1 Hz, 1H), 7.33–7.26 (m, 3H), 7.21–7.15 (m, 2H), 7.14–7.11 (m, 2H), 6.86 (s, 1H), 3.85 (s, 3H), 2.71–2.59 (m, 1H), 1.88–1.78 (m, 1H), 1.61–1.43 (m, 1H), 1.35–1.24 (m, 1H), 1.22–1.11 (m, 1H), 1.08–0.92 (m, 1H), 0.82–0.63 (m, 4H), 0.58–0.37 (m, 2H), 0.37–0.22 (m, 2H). **^{13}C NMR** (75.4 MHz, $CDCl_3$): δ (ppm) = 143.9, 138.7, 136.6, 131.4, 129.0, 128.3, 127.8, 125.7, 125.2, 121.4, 120.6, 119.0, 113.5, 109.0, 32.9, 28.4, 24.1, 16.4, 15.8, 12.2, 6.4, 6.2, 6.1, 6.0. **EI-LRMS** *m/z* (%): 353 (M^+, 30), 249 (100), 234 (63). **HRMS** (ESI-TOF) *m/z*: calcd for $C_{26}H_{28}N^+$ $[M+H]^+$ 354.2216; found 354.2213.

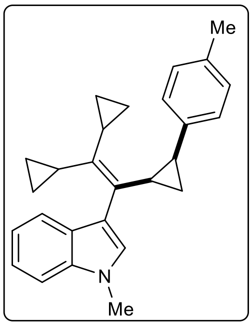

3-(2,2-Dicyclopropyl-1-((1*R,2*S**)-2-(*p*-tolyl)cyclopropyl)vinyl)-1-methyl-1*H*-indole (*cis*-2b)**: Following the general procedure F1 with indole **1a** (124 mg, 0.5 mmol) and 1-methyl-4-vinylbenzene (0.75 mmol, 88 mg), the crude product was purified by column chromatography (hexane/EtOAc, 7/1) affording pure **2b** as an yellow oil (150 mg, 82%). R_f = 0.42 (hexane/EtOAc, 5/1). Obtained as a ca. 18/1 mixture and isolated as a ca. 10/1 mixture of *cis/trans* diastereoisomers. Data for the major diastereoisomer: **^{1}H NMR** (300 MHz, $CDCl_3$): δ (ppm) = 7.51 (ad, J = 8.0 Hz, 1H), 7.33 (ad, J = 8.0 Hz, 1H), 7.26 (at, J = 7.3 Hz, 1H), 7.13 (at, J = 7.3 Hz, 1H), 7.02 (ad, J = 8.1 Hz, 2H), 6.94 (ad, J = 8.1 Hz, 2H), 6.30 (s, 1H), 3.74 (s, 3H), 2.60–2.50 (m, 1H), 2.40 (s, 3H), 2.43–2.31 (m, 1H), 1.72–1.56 (m, 1H), 1.43–1.23 (m, 1H), 1.24–1.13 (m, 1H), 1.10–1.01 (m, 1H), 0.98–0.91 (m, 1H), 0.90–0.80 (m, 1H), 0.79–0.66 (m, 2H), 0.51–0.33 (m, 1H), 0.36–0.28 (m, 1H), 0.21–0.07 (m, 2H). **^{13}C NMR** (75.4 MHz, $CDCl_3$): δ (ppm) = 140.3 (C), 137.4 (C), 136.3 (C), 134.4 (C), 128.6 (CH), 128.5 (C), 128.4 (2 × CH), 128.1 (2 × CH), 127.6 (C), 121.1 (CH), 120.9 (CH), 118.5 (CH), 116.8 (C), 108.8 (CH), 32.7 (CH_3), 25.6 (CH), 24.5 (CH), 21.1 (CH_3), 14.7 (CH_2), 14.2 (CH), 13.9 (CH), 7.5 (CH_2), 6.6 (CH_2), 6.0 (CH_2), 5.7 (CH_2). **EI-LRMS** *m/z* (%): 367 (M^+, 25), 249 (95), 234 (100). **HRMS** (ESI-TOF) *m/z*: calcd for $C_{27}H_{30}N^+$ $[M+H]^+$ 368.2373; found 368.2378.

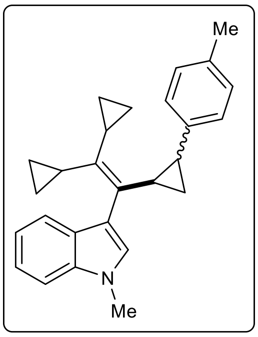

3-(2,2-Dicyclopropyl-1-(2-(*p*-tolyl)cyclopropyl)vinyl)-1-methyl-1*H*-indole (2b): Following the general procedure F2 with **1a** (124 mg, 0.5 mmol) and 1-methyl-4-vinylbenzene (0.75 mmol, 88 mg), the crude product was purified by column chromatography (hexane/EtOAc, 10/1) affording pure **2b** as an orange oil (145 mg, 79%). R_f = 0.33 (hexane/EtOAc, 10/1). Obtained as a 1.4/1 mixture and isolated as a ca. 1/1.5 mixture of *cis/trans* diastereoisomers. Data for both diastereoisomers: **^{1}H NMR** (300 MHz, $CDCl_3$): δ (ppm) = 7.61 (ad, J = 7.8 Hz, 1H), 7.52 (ad, J = 7.9 Hz, 1H), 7.40 (ad, J = 8.1 Hz, 1H), 7.36–7.21 (m, 4H), 7.22–7.11 (m, 3H), 7.11–7.00 (m, 4H), 6.95 (ad, J = 8.1 Hz, 2H), 6.89 (s, 1H), 6.31 (s, 1H), 3.87 (s, 3H), 3.75 (s, 3H), 2.71–2.60 (m, 1H), 2.60–2.51 (m, 1H), 2.41 (s, 6H), 2.43–2.40 (m, 1H), 1.87–1.79 (m, 1H), 1.75–1.61 (m, 1H), 1.59–1.50 (m, 2H), 1.43–1.27 (m, 2H), 1.28–1.10 (m, 2H), 1.10–0.91 (m, 3H), 0.93–0.67 (m, 7H), 0.59–0.39 (m, 3H), 0.39–0.28 (m, 2H), 0.23–0.10 (m, 2H). **^{13}C NMR** (75.4 MHz, $CDCl_3$): δ (ppm) = 140.8, 140.3, 138.5, 137.4, 136.6, 136.3, 134.6, 134.4, 131.5, 129.0, 128.5, 128.4, 128.1, 127.8, 127.6, 125.6, 121.4, 121.1, 120.9, 120.6, 119.0, 118.5, 116.8, 113.6,

109.0, 108.8, 32.8, 32.7, 28.0, 25.6, 24.5, 23.7, 21.1, 21.0, 16.2, 15.8, 14.7, 14.2, 13.9, 12.2, 7.5, 6.6, 6.4, 6.2, 6.1, 6.0, 5.9, 5.7. **EI-LRMS** *m/z* (%): 367 (M^+, 25), 249 (100), 248 (46). **HRMS** (ESI-TOF) *m/z*: calcd for $C_{27}H_{30}N^+$ $[M+H]^+$ 368.2373; found 368.2365.

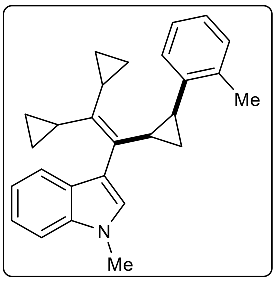

3-(2,2-Dicyclopropyl-1-((1*R,2*S**)-2-(*o*-tolyl)cyclopropyl)vinyl)-1-methyl-1*H*-indole (*cis*-2c)**: Following the general procedure F1 with **1a** (124 mg, 0.5 mmol) and 1-methyl-2-vinylbenzene (0.75 mmol, 88 mg), the crude product was purified by column chromatography (hexane/EtOAc, 7/1) affording pure **2c** as a brown oil (132 mg , 72%). R_f = 0.42 (hexane/EtOAc, 5/1). Obtained and isolated as a ca. 8/1 mixture of *cis/trans* diastereoisomers. Data for the major diastereoisomer: **^{1}H NMR** (300 MHz, $CDCl_3$): δ (ppm) = 7.55 (ad, *J* = 7.9 Hz, 1H), 7.35–7.25 (m, 2H), 7.21–7.10 (m, 3H), 7.01–6.91 (m, 1H), 6.74 (ad, *J* = 7.7 Hz, 1H), 5.88 (s, 1H), 3.65 (s, 3H), 2.90–2.78 (m, 1H), 2.46 (s, 3H), 2.49–2.34 (m, 1H), 1.83–1.73 (m, 1H), 1.42–1.29 (m, 1H), 1.29–1.14 (m, 2H), 1.08–0.97 (m, 1H), 0.97–0.91 (m, 1H), 0.91–0.75 (m, 2H), 0.50–0.33 (m, 2H), 0.28–0.09 (m, 2H). **^{13}C NMR** (75.4 MHz, $CDCl_3$): δ (ppm) = 140.1, 138.4, 138.0, 136.2, 129.1, 128.9, 128.6, 128.3, 127.7, 125.0, 124.6, 120.7, 120.6, 118.5, 115.6, 108.7, 32.4, 24.5, 21.9, 20.0, 14.6, 13.6, 12.8, 7.4, 6.9, 6.1, 5.7. **EI-LRMS** *m/z* (%): 367 (M^+, 21), 249 (100), 234 (69). **HRMS** (ESI-TOF) *m/z*: calcd for $C_{27}H_{30}N^+$ $[M+H]^+$ 368.2373; found 368.2375.

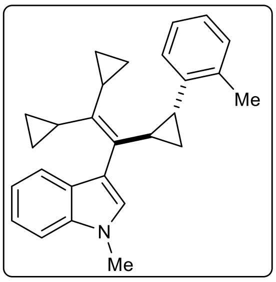

3-(2,2-Dicyclopropyl-1-((1*R,2*R**)-2-(*o*-tolyl)cyclopropyl)vinyl)-1-methyl-1*H*-indole (*trans*-2c):** Following the general procedure F2 with **1a** (124 mg, 0.5 mmol) and 1-methyl-2-vinylbenzene (0.75 mmol, 88 mg), the crude product was purified by column chromatography (hexane/EtOAc, 10/1) affording pure **2c** as a yellow oil (139 mg, 76%). R_f = 0.35 (hexane/EtOAc, 10/1). Obtained as a ca. 1/4 mixture and isolated as a ca. 1/6.5 mixture of *cis/trans* diastereoisomers. Data for the major diastereoisomer: **^{1}H NMR** (500 MHz, $CDCl_3$): δ (ppm) = 7.58 (ad, *J* = 7.9 Hz, 1H), 7.38 (ad, *J* = 8.2 Hz, 1H), 7.32–7.27 (m, 1H), 7.22–7.16 (m, 2H), 7.16–7.11 (m, 2H), 7.11–7.04 (m, 1H), 6.87 (s, 1H), 3.86 (s, 3H), 2.71–2.63 (m, 1H), 2.38 (s, 3H), 1.94–1.81 (m, 1H), 1.59–1.48 (m, 1H), 1.32–1.25 (m, 1H), 1.17–1.10 (m, 1H), 1.06–0.98 (m, 1H), 0.87–0.65 (m, 4H), 0.60–0.47 (m, 2H), 0.43–0.31 (m, 1H), 0.31–0.21 (m, 1H). **^{13}C NMR** (75.4 MHz, $CDCl_3$): δ (ppm) = 141.6, 138.3, 137.4, 136.7, 131.8, 129.7, 129.1, 127.5, 125.9, 125.4, 125.0, 121.4, 120.6, 119.0, 113.4, 109.1, 32.9, 26.1, 21.3, 20.1, 15.9, 14.4, 12.1, 6.4, 6.3, 6.2, 6.0. **HRMS** (ESI-TOF) *m/z*: calcd for $C_{27}H_{30}N^+$ $[M+H]^+$ 368.2373; found 368.2375.

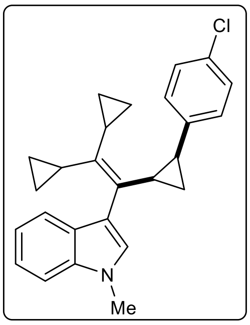

3-(1-((1*R,2*S**)-2-(4-Chlorophenyl)cyclopropyl)-2,2-dicyclopropylvinyl)-1-methyl-1*H*-indole (*cis*-2d):** Following the general procedure F1 with **1a** (104 mg, 0.5 mmol) and 1-chloro-4-vinylbenzene (0.75 mmol, 104 mg), the crude product was purified by column chromatography (hexane/EtOAc, 10/1) affording pure **2d** as an orange oil (157 mg, 81%). R_f = 0.30 (hexane/EtOAc, 10/1). Obtained and isolated as a ca. 17/1 mixture of *cis/trans* diastereoisomers. Data for the major diastereoisomer: **^{1}H NMR** (300 MHz, $CDCl_3$): δ (ppm) = 7.42 (ad, *J* = 7.9 Hz, 1H), 7.30 (ad, *J* = 7.3 Hz, 1H), 7.22 (at, *J* = 7.1 Hz, 1H), 7.10 (ad, *J* = 8.3 Hz, 3H), 6.88 (ad, *J* = 8.3 Hz, 2H), 6.33 (s, 1H), 3.73 (s, 3H), 2.61–2.45 (m, 1H), 2.38–2.28 (m, 1H), 1.68–1.52 (m, 1H), 1.41–1.25 (m, 1H), 1.22–1.05 (m, 1H), 1.04–0.92 (m, 1H), 0.89–0.75 (m, 2H), 0.73–0.61 (m, 2H), 0.47–0.36 (m, 1H), 0.30–0.16 (m, 1H), 0.15–0.00 (m, 2H).

^{13}C NMR (75.4 MHz, $CDCl_3$): δ (ppm) = 140.8, 139.1, 136.3, 130.7, 129.8, 128.6, 128.4, 127.4, 127.0, 121.10, 121.05, 118.7, 116.6, 108.9, 32.8, 25.8, 24.5, 14.4, 14.3, 14.0, 7.8, 6.7, 6.0, 5.8.**EI-LRMS** *m/z* (%): 389 (M^+, 37), 387 (100), 358 (44). **HRMS** (ESI-TOF) *m/z*: could not be recorded.

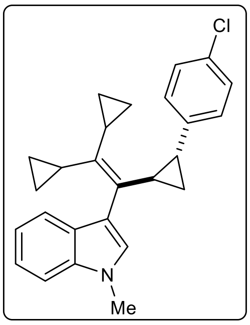

3-(1-((1*R,2*R**)-2-(4-Chlorophenyl)cyclopropyl)-2,2-dicyclopropylvinyl)-1-methyl-1*H*-indole (*trans*-2d):** Following the general procedure F2 with **1a** (124 mg, 0.5 mmol) and 1-chloro-4-vinylbenzene (0.75 mmol, 103 mg), the crude product was purified by column chromatography (hexane/EtOAc, 10/1) affording pure **2d** as a yellow oil (142 mg, 73%). R_f = 0.32 (hexane/EtOAc, 10/1). Obtained as a ca. 1/3.5 mixture and isolated as a ca. 1/4 mixture of *cis/trans* diastereoisomers. Data for the major diastereoisomer: **^{1}H NMR** (300 MHz, $CDCl_3$): δ (ppm) = 7.50–7.47 (m, 1H), 7.33 (ad, *J* = 8.2 Hz, 1H), 7.27–7.24 (m, 1H), 7.24–7.20 (m, 2H), 7.15–7.10 (m, 1H), 7.00–6.96 (m, 2H), 6.81 (s, 1H), 3.82 (s, 3H), 2.58–2.49 (m, 1H), 1.76–1.65 (m, 1H), 1.48–1.41 (m, 1H), 1.35–1.24 (m, 1H), 1.25–1.17 (m, 1H), 1.13–1.03 (m, 1H), 1.02–0.93 (m, 1H), 0.94–0.84 (m, 1H), 0.73–0.68 (m, 1H), 0.68–0.57 (m, 3H), 0.48–0.39 (m, 1H), 0.34–0.16 (m, 1H).**^{13}C NMR** (75.4 MHz, $CDCl_3$): δ (ppm) = 142.5, 139.0, 136.6, 131.0, 130.7, 129.8, 128.3, 126.9, 121.4, 120.5, 119.0, 113.3, 109.0, 32.9, 28.6, 23.5, 16.4, 15.8, 12.1, 6.4, 6.2, 6.0, 5.9.

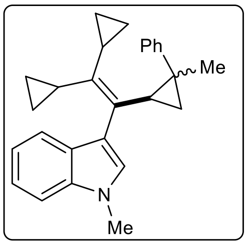

3-(2,2-Dicyclopropyl-1-(2-methyl-2-phenylcyclopropyl)vinyl)-1-methyl-1*H*-indole (2e): Following the general procedure F1 with **1a** (124 mg, 0.5 mmol) and α-methystyrene (0.75 mmol, 88 mg), the crude product was purified by column chromatography (hexane/EtOAc, 7/1) affording pure **2e** as a yellow oil (134 mg, 73%). R_f = 0.24 (hexane/EtOAc, 3/1). Obtained as a ca. 1/1.2 mixture and isolated as a ca. 1.4/1 mixture of *cis/trans* diastereoisomers. Data for both diastereoisomers: **^{1}H NMR** (300 MHz, $CDCl_3$): δ (ppm) = 7.68 (ad, *J* = 7.9 Hz, 1H), 7.46–7.31 (m, 6H), 7.29–7.20 (m, 7H), 7.20–7.09 (m, 4H), 6.94 (as, 1H), 5.49 (as, 1H), 3.88 (s, 3H), 3.58 (s, 3H), 2.57–2.50 (m, 1H), 2.49–2.41 (m, 1H), 1.85–1.69 (m, 2H), 1.62 (s, 3H), 1.53–1.46 (m, 1H), 1.41 (s, 3H), 1.34–1.26 (m, 1H), 1.19–1.09 (m, 2H), 1.05–0.86 (m, 6H), 0.85–0.74 (m, 2H), 0.71–0.52 (m, 3H), 0.42–0.28 (m, 3H), 0.26–0.01 (m, 4H). **^{13}C NMR** (75.4 MHz, $CDCl_3$): δ (ppm) = 148.0, 144.1, 140.9, 139.5, 136.5, 136.2, 129.8, 129.4, 128.71, 128.69, 128.65, 128.4, 128.3, 128.2, 127.5, 125.3, 124.8, 124.7, 121.2, 120.7, 120.6, 118.8, 118.5, 116.9, 115.1, 109.0, 108.6, 34.5, 32.8, 32.4, 31.2, 28.9, 27.7, 25.8, 23.7, 19.8, 18.8, 14.8, 14.4, 13.7, 13.7, 8.4, 7.3, 6.2, 6.11, 6.05, 6.0, 5.8, 5.4. One CH was not observed due to overlapping. **EI-LRMS** *m/z* (%): 367 (M^+, 59), 272 (63), 144 (100). **HRMS** (ESI-TOF) *m/z*: calcd for $C_{27}H_{30}N^+$ $[M+H]^+$ 368.2373; found 368.2376.

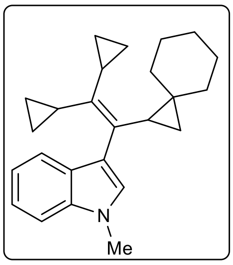

3-(2,2-Dicyclopropyl-1-(spiro[2.5]octan-1-yl)vinyl)-1-methyl-1*H*-indole (2f): Following the general procedure F1 with **1a** (124 mg, 0.5 mmol) and methylenecyclohexane (0.75 mmol, 72 mg), the crude product was purified by column chromatography (hexane/EtOAc, 7/1) affording pure **2f** as a colourless oil (138 mg, 80%). R_f = 0.52 (hexane/EtOAc, 5/1). **^{1}H NMR** (300 MHz, $CDCl_3$): δ (ppm) = 7.54 (ad, *J* = 7.4 Hz, 1H), 7.31 (ad, *J* = 7.4 Hz, 1H), 7.22 (at, *J* = 7.4 Hz, 1H), 7.09 (at, *J* = 7.4 Hz, 1H), 6.79 (s, 1H), 3.81 (s, 3H), 1.91–1.73 (m, 3H), 1.71–1.38 (m, 5H), 1.34–1.14 (m, 3H), 1.12–1.03 (m, 1H), 1.03–0.89 (m, 2H), 0.82–0.69 (m, 3H), 0.60–0.52 (m, 1H), 0.51–0.41 (m, 1H), 0.31–0.18 (m, 2H), 0.10–0.07 (m, 2H). **^{13}C NMR** (75.4

MHz, $CDCl_3$): δ (ppm) = 138.8, 136.5, 129.4, 128.51, 128.47, 121.3, 120.9, 118.6, 117.5, 108.8, 38.0, 32.8, 31.0, 29.4, 27.1, 26.8, 25.9, 25.8, 19.4, 14.6, 13.7, 8.1, 6.1, 6.0. **EI-LRMS** *m/z* (%): 345 (M^+, 65), 250 (100), 144 (61). **HRMS** (ESI-TOF) *m/z*: calcd for $C_{25}H_{32}N^+$ $[M+H]^+$ 346.2529; found 346.2536.

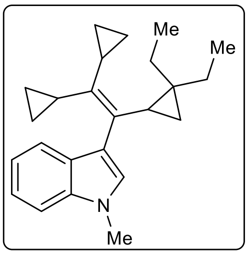

3-(2,2-Dicyclopropyl-1-(2,2-diethylcyclopropyl)vinyl)-1-methyl-1*H*-indole (2g): Following the general procedure F1 with **1a** (124 mg, 0.5 mmol) and 3-methylenepentane (0.75 mmol, 91 mg), the crude product was purified by column chromatography (hexane/EtOAc, 7/1) affording pure **2g** as a yellow oil (139 mg, 84%). R_f = 0.35 (hexane/EtOAc, 5/1). **^{1}H NMR** (300 MHz, $CDCl_3$): δ (ppm) = 7.62 (ad, *J* = 7.6 Hz, 1H), 7.37 (ad, *J* = 7.6 Hz, 1H), 7.28 (at, *J* = 7.6 Hz, 1H), 7.16 (at, *J* = 7.6 Hz, 1H), 6.85 (s, 1H), 3.84 (s, 3H), 2.10–1.85 (m, 2H), 1.82–1.63 (m, 1H), 1.50–1.36 (m, 1H), 1.35–1.18 (m, 3H), 1.14–1.08 (m, 1H), 1.04–0.92 (m, 6H), 0.89–0.76 (m, 3H), 0.70–0.62 (m, 1H), 0.55–0.46 (m, 1H), 0.38–0.27 (m, 2H), 0.19–0.02 (m, 2H). **^{13}C NMR** (75.4 MHz, $CDCl_3$): δ (ppm) = 138.6, 136.5, 129.5, 128.6, 128.4, 121.2, 121.0, 118.6, 117.4, 108.8, 32.7, 29.5, 29.3, 28.7, 24.1, 18.2, 14.7, 13.8, 10.9, 10.3, 7.9, 6.3, 5.6, 5.2. **EI-LRMS** *m/z* (%): 333 (M^+, 54), 238 (100), 144 (87). **HRMS** (ESI-TOF) *m/z*: calcd for $C_{24}H_{32}N^+$ $[M+H]^+$ 334.2529; found 334.2526.

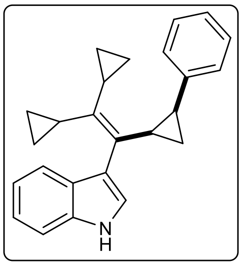

3-(2,2-Dicyclopropyl-1-((1*R,2*S**)-2-phenylcyclopropyl)vinyl)-1*H*-indole (*cis*-2h)**: Following the general procedure F1 with **1b** (118 mg, 0.5 mmol) and styrene (0.75 mmol, 78 mg), the crude product was purified by column chromatography (hexane/EtOAc, 7/1) affording pure **2h** as a yellow oil (113 mg, 67%). R_f = 0.30 (hexane/EtOAc, 10/1). Obtained as a ca. 8/1 mixture and isolated as a ca. 7/1 mixture of *cis/trans* diastereoisomers. Data for the major diastereoisomer: **^{1}H NMR** (300 MHz, $CDCl_3$): δ (ppm) = 7.87 (br s, 1H), 7.54 (ad, *J* = 7.8 Hz, 1H), 7.40–7.31 (m, 1H), 7.28–7.11 (m, 5H), 7.09–7.04 (m, 2H), 6.45 (ad, *J* = 1.2 Hz, 1H), 2.64–2.52 (m, 1H), 2.49–2.38 (m, 1H), 1.79–1.65 (m, 1H), 1.45–1.31 (m, 1H), 1.21–1.10 (m, 1H), 1.11–1.02 (m, 1H), 0.96–0.84 (m, 2H), 0.78–0.69 (m, 2H), 0.51–0.37 (m, 1H), 0.32–0.22 (m, 1H), 0.17–0.03 (m, 2H). **^{13}C NMR** (75.4 MHz, $CDCl_3$): δ (ppm) = 140.9, 140.5, 135.5, 128.5, 128.0, 127.4, 127.3, 125.2, 123.8, 121.4, 121.0, 119.2, 118.5, 110.8, 25.7, 24.9, 14.30, 14.25, 14.2, 7.6, 6.7, 6.0, 5.7. **EI-LRMS** *m/z* (%): 339 (M^+, 38), 235 (100), 220 (50). **HRMS** (ESI-TOF) *m/z*: calcd for $C_{25}H_{26}N^+$ $[M+H]^+$ 340.2060; found 340.2056.

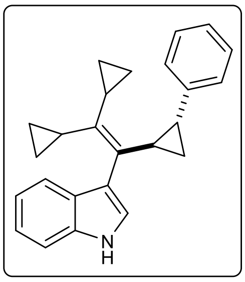

3-(2,2-Dicyclopropyl-1-((1*R,2*R**)-2-phenylcyclopropyl)vinyl)-1*H*-indole (*trans*-2h)**: Following the general procedure F2 with **1b** (117 mg, 0.5 mmol) and styrene (0.75 mmol, 78 mg), the crude product was purified by column chromatography (hexane/EtOAc, 10/1) affording pure **2h** as a yellow oil (115 mg, 68%). R_f = 0.30 (hexane/EtOAc, 10/1). Obtained as a ca. 1/3 mixture and isolated as a ca. 1/6 mixture of *cis/trans* diastereoisomers. Data for the major diastereoisomer: **^{1}H NMR** (300 MHz, $CDCl_3$): δ (ppm) = 8.01 (br s, 1H), 7.64 (ad, *J* = 7.7 Hz, 1H), 7.43 (ad, *J* = 7.7 Hz, 1H), 7.40–7.30 (m, 3H), 7.29–7.12 (m, 4H), 6.99 (d, *J* = 2.3 Hz, 1H), 2.75–2.61 (m, 1H), 1.93–1.77 (m, 1H), 1.66–1.51 (m, 1H), 1.37–1.25 (m, 1H), 1.25–1.15 (m, 1H), 1.12–1.01 (m, 1H), 0.87–0.66 (m, 4H), 0.56–0.42 (m, 2H), 0.41–0.26 (m, 2H). **^{13}C NMR** (75.4 MHz, $CDCl_3$): δ (ppm) = 143.8, 139.0, 135.7, 131.3, 128.5, 128.3, 125.7, 125.2, 123.0, 121.9, 120.4, 119.6, 115.0, 111.0, 28.3, 24.0, 16.3, 15.7, 12.3, 6.4, 6.2, 6.1, 5.9. **EI-LRMS** *m/z*

(%): 339 (M$^+$, 25), 235 (93), 206 (100). **HRMS** (ESI-TOF) *m/z*: calcd for $C_{25}H_{26}N^+$ [M+H]$^+$ 340.2060; found 340.2047.

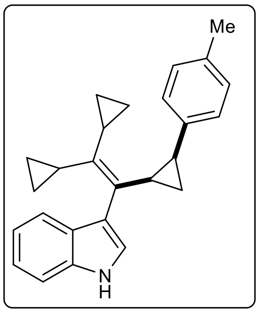

3-(2,2-Dicyclopropyl-1-((1*R,2*S**)-2-(*p*-tolyl)cyclopropyl)vinyl)-1*H*-indole (*cis*-2i)**: Following the general procedure F1 with **1b** (117 mg, 0.5 mmol) and 1-methyl-4-vinylbenzene (0.75 mmol, 88 mg), the crude product was purified by column chromatography (hexane/EtOAc, 7/1) affording pure **2i** as a yellow oil (135 mg, 77%). R_f = 0.46 (hexane/EtOAc, 5/1). Obtained and isolated as a ca. 9/1 mixture of *cis/trans* diastereoisomers. Data for the major diastereoisomer: **^{1}H NMR** (300 MHz, $CDCl_3$): δ (ppm) = 7.83 (br s, 1H), 7.56 (ad, *J* = 7.8 Hz, 1H), 7.37 (ad, *J* = 7.8 Hz, 1H), 7.27 (at, *J* = 7.4 Hz, 1H), 7.19 (at, *J* = 7.4 Hz, 1H), 7.07 (ad, *J* = 8.1 Hz, 2H), 7.00 (ad, *J* = 8.1 Hz, 2H), 6.50 (d, *J* = 2.3 Hz, 1H), 2.68–2.53 (m, 1H), 2.50–2.36 (m, 1H), 2.43 (s, 3H), 1.83–1.72 (m, 1H), 1.45 1.28 (m, 1H), 1.25–1.16 (m, 1H), 1.13–1.05 (m, 1H), 1.05–0.96 (m, 1H), 0.9–0.85 (m, 1H), 0.84–0.70 (m, 2H), 0.53–0.40 (m, 1H), 0.38–0.22 (m, 1H), 0.22–0.11 (m, 2H). **^{13}C NMR** (75.4 MHz, $CDCl_3$): δ (ppm) = 140.6, 137.3, 135.4, 134.4, 129.0, 128.3, 128.1, 127.5, 123.7, 121.3, 121.0, 119.0, 118.4, 110.7, 25.5, 24.5, 21.1, 14.4, 14.1, 7.5, 6.6, 5.9, 5.6. **EI-LRMS** *m/z* (%): 353 (M$^+$, 25), 235 (100), 206 (63). **HRMS** (ESI-TOF) *m/z*: calcd for $C_{26}H_{28}N^+$ [M+H]$^+$ 354.2216; found 354.2216.

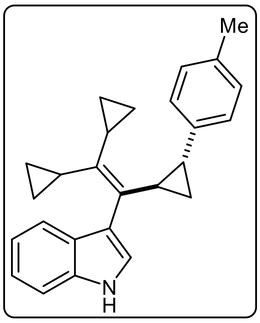

3-(2,2-Dicyclopropyl-1-((1*R,2*R**)-2-(*p*-tolyl)cyclopropyl)vinyl)-1*H*-indole (*trans*-2i):** Following the general procedure F1 with **1b** (117 mg, 0.5 mmol) and 1-methyl-4-vinylbenzene (0.75 mmol, 88 mg), the crude product was purified by column chromatography (hexane/EtOAc, 7/1) affording pure **2i** as a yellow oil (112 mg, 64%). R_f = 0.46 (hexane/EtOAc, 5/1). Obtained as a ca. 1/8 mixture of *cis/trans* diastereoisomers and isolated as a ca. 1/20. Data for the major diastereoisomer: **^{1}H NMR** (300 MHz, $CDCl_3$): δ (ppm) = 8.02 (br s, 1H), 7.63 (ad, *J* = 7.8 Hz, 1H), 7.43 (ad, *J* = 7.8 Hz, 1H), 7.30 (t, *J* = 6.9 Hz, 1H), 7.27–7.13 (m, 3H), 7.07 (ad, *J* = 8.1 Hz, 2H), 6.99 (ad, *J* = 2.3 Hz, 1H), 2.72–2.61 (m, 1H), 2.41 (s, 3H), 1.90–1.74 (m, 1H), 1.70–1.53 (m, 1H), 1.38–1.24 (m, 1H), 1.22–1.10 (m, 1H), 1.07–0.95 (m, 1H), 0.89–0.65 (m, 4H), 0.56–0.44 (m, 2H), 0.42–0.24 (m, 2H). **^{13}C NMR** (75.4 MHz, $CDCl_3$): δ (ppm) = 140.7, 138.8, 135.7, 134.7, 131.4, 129.0, 128.5, 125.6, 123.0, 121.8, 120.5, 119.5, 115.1, 111.0, 27.9, 23.7, 21.1, 16.2, 15.7, 12.3, 6.4, 6.2, 6.1, 5.9. **EI-LRMS** *m/z* (%): 353 (M$^+$, 25), 235 (100), 220 (98). **HRMS** (ESI-TOF) *m/z*: calcd for $C_{26}H_{28}N^+$ [M+H]$^+$ 354.2216; found 354.2207.

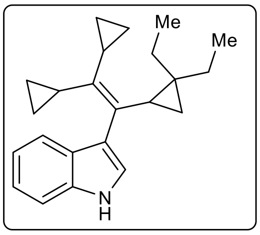

3-(2,2-Dicyclopropyl-1-(2,2-diethylcyclopropyl)vinyl)-1*H*-indole (2j): Following the general procedure F1 with **1b** (118 mg, 0.5 mmol) and 3-methylenepentane (0.75 mmol, 91 mg), the crude product was purified by column chromatography (hexane/EtOAc, 7/1) affording pure **2j** as a yellow solid (102 mg, 64%). R_f = 0.38 (hexane/EtOAc, 5/1); m.p. = 92–94 °C. **^{1}H NMR** (300 MHz, $CDCl_3$): δ (ppm) = 7.98 (br s, 1H), 7.58 (ad, *J* = 7.8 Hz, 1H), 7.37 (ad, *J* = 7.8 Hz, 1H), 7.21 (at, *J* = 7.3 Hz, 1H), 7.13 (at, *J* = 7.3 Hz, 1H), 6.92 (as, 1H), 2.03–1.86 (m, 2H), 1.74–1.56 (m, 1H), 1.48–1.31 (m, 1H), 1.29–1.03 (m, 4H), 0.96–0.83 (m, 6H), 0.82–0.73 (m, 3H), 0.64–0.59 (m, 1H), 0.50–0.40 (m, 1H), 0.36–0.15 (m, 2H), 0.13–0.03 (m, 1H), –0.04– –0.09 (m, 1H). **^{13}C NMR** (75.4 MHz, $CDCl_3$): δ (ppm) = 139.1, 135.7, 129.5, 128.2, 123.6, 121.5,

121.1, 119.1, 119.0, 110.8, 29.5, 29.2, 28.7, 24.0, 18.2, 14.8, 13.6, 10.9, 10.4, 7.8, 6.3, 5.6, 5.2. **EI-LRMS** *m/z* (%): 319 (M^+, 36), 224 (100), 130 (89). **HRMS** (ESI-TOF): calcd for $C_{23}H_{30}N^+$ $[M+H]^+$ 320.2373; found 320.2370.

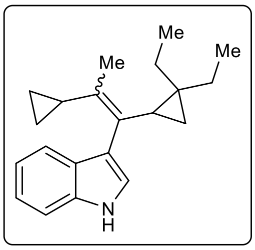

3-(2-Cyclopropyl-1-(2,2-diethylcyclopropyl)prop-1-en-1-yl)-1*H*-indole (2k): Following the general procedure F1 with **1c** (104 mg, 0.5 mmol) and 3-methylenepentane (0.75 mmol, 91 mg), the crude product was purified by column chromatography (hexane/EtOAc, 7/1) affording pure **2k** as an orange oil (70 mg, 48%). R_f = 0.41 (hexane/EtOAc, 5/1). Obtained and isolated as a ca. 2/1 mixture of *E*/*Z* diastereoisomers. Data for both diastereoisomers: **^{1}H NMR** (300 MHz, $CDCl_3$): δ (ppm) = 7.99 (br s, 1H, *E* diast), 7.96 (s, 1H, *Z* diast), 7.68 (ad, *J* = 7.8 Hz, 1H, *E* diast), 7.57 (ad, *J* = 7.8 Hz, 1H, *Z* diast), 7.39 (ad, *J* = 7.8 Hz, 2H, both diast), 7.30–7.12 (m, 4H, both diast), 7.02 (ad, *J* = 2.3 Hz, 1H, *E* diast), 6.90 (ad, *J* = 2.3 Hz, 1H, *Z* diast), 2.39–2.24 (m, 1H, *Z* diast), 2.03–1.92 (m, 1H, *Z* diast), 1.87–1.73 (m, 2H, *E* diast), 1.68 (s, 3H, *E* diast), 1.64–1.53 (m, 2H, both diast), 1.40 (s, 3H, *Z* diast), 1.49–1.31 (m, 3H, both diast), 1.31–1.17 (m, 3H, both diast), 1.01–0.91 (m, 8H, both diast), 0.92–0.73 (m, 7H, both diast), 0.69–0.48 (m, 6H, both diast), 0.45–0.30 (m, 1H, *E* diast), 0.17–0.06 (m, 2H, both diast). **^{13}C NMR** (75.4 MHz, $CDCl_3$): δ (ppm) = 136.2, 136.1, 135.8, 135.7, 128.1, 127.8, 126.1, 125.9, 123.5, 123.1, 121.60, 121.57, 121.2, 121.0, 119.6, 119.3, 119.2, 118.7, 111.0, 110.9, 29.6, 29.4, 28.7, 28.6, 24.2, 24.1, 18.7, 18.6, 16.1, 14.9, 13.3, 10.71, 10.67, 10.5, 10.4, 4.9, 4.8, 4.52, 4.48. **EI-LRMS** *m/z* (%): 293 (M^+, 25), 222 (100), 209 (91). **HRMS** (ESI-TOF) *m/z*: calcd for $C_{21}H_{28}N^+$ $[M+H]^+$ 294.2216; found 294.2214.

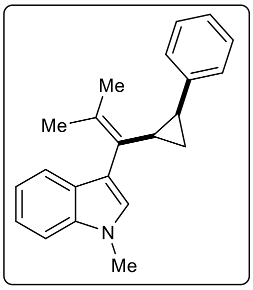

1-Methyl-3-(2-methyl-1-((1*R,2*S**)-2-phenylcyclopropyl)prop-1-en-1-yl)-1*H*-indole (*cis*-2l):** Following the general procedure F1 with **1d** (104 mg, 0.5 mmol) and styrene (0.75 mmol, 78 mg), the crude product was purified by column chromatography (hexane/EtOAc, 7/1) affording pure **2l** as an orange oil (117 mg, 78%). R_f = 0.31 (hexane/EtOAc, 5/1). Obtained and isolated as a ca. 8/1 mixture of *cis/trans* diastereoisomers. Data for the major diastereoisomer: **^{1}H NMR** (300 MHz, $CDCl_3$): δ (ppm) = 7.58 (ad, *J* = 7.9 Hz, 1H), 7.46–7.36 (m, 2H), 7.36–7.21 (m, 4H), 7.16–7.08 (m, 2H), 6.45 (s, 1H), 3.82 (s, 3H), 2.55–2.45 (m, 2H), 1.99 (s, 3H), 1.75 (s, 3H), 1.41–1.30 (m, 1H), 0.95–0.83 (m, 1H). **^{13}C NMR** (75.4 MHz, $CDCl_3$): δ (ppm) = 140.4, 136.5, 134.2, 128.5, 128.2, 128.1, 127.3, 125.1, 123.2, 121.2, 121.1, 118.7, 116.8, 109.0, 32.6, 25.0, 24.7, 23.0, 21.3, 13.6. **EI-LRMS** *m/z* (%): 301 (M^+, 100), 220 (92), 210 (57). **HRMS** (ESI-TOF) *m/z*: calcd for $C_{22}H_{24}N^+$ $[M+H]^+$ 302.1903; found 302.1902.

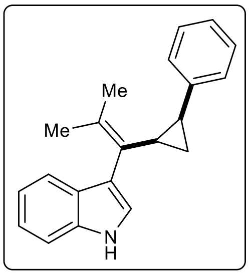

3-(2-Methyl-1-((1*R,2*S**)-2-phenylcyclopropyl)prop-1-en-1-yl)-1*H*-indole (*cis*-2m)**: Following the general procedure F1 with **1e** (118 mg, 0.5 mmol) and styrene (0.75 mmol, 78 mg), but carrying out at 80°C for 2 h, the crude product was purified by column chromatography (hexane/EtOAc, 7/1) affording pure **2m** as a yellow oil (93 mg, 65%). R_f = 0.38 (hexane/EtOAc, 5/1). Obtained and isoalted as a ca. 5/1 mixture of *cis/trans* diastereoisomers. Data for the major diastereoisomer: **^{1}H NMR** (300 MHz, $CDCl_3$): δ (ppm) = 7.90 (br s, 1H), 7.50 (ad, *J* = 8.0 Hz, 2H), 7.38 (ad, *J* = 8.0 Hz, 2H), 7.30–7.11 (m, 5H), 7.06–6.99 (m, 2H), 6.48 (d, *J* = 2.1 Hz, 1H), 2.51–2.36 (m, 2H), 1.89 (s, 3H), 1.63 (s, 3H), 1.31–1.20 (m, 1H), 0.83–0.73 (m, 1H). **^{13}C NMR** (75.4 MHz, $CDCl_3$): δ (ppm) = 140.3, 135.8, 134.7, 128.6, 127.4, 125.9, 125.2, 123.4,

123.2, 121.6, 121.1, 119.3, 118.5, 110.9, 24.9, 24.5, 22.9, 21.3, 13.5. **EI-LRMS** *m/z* (%): 287 (M^+, 87), 196 (97), 155 (100). **HRMS** (ESI-TOF) *m/z*: calcd for $C_{21}H_{22}N^+$ $[M+H]^+$ 288.1747; found 288.1746.

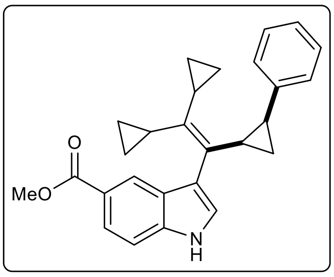

Methyl 3-(2,2-dicyclopropyl-1-((1*R,2*S**)-2-phenylcyclopropyl)vinyl)-1*H*-indole-5-carboxylate (*cis*-2n):** Following the general procedure F1 with **1f** (147 mg, 0.5 mmol) and styrene (0.75 mmol, 78 mg), the crude product was purified by column chromatography (hexane/EtOAc, 7/1) affording pure **2n** as a yellow oil (143 mg, 72%). R_f = 0.25 (hexane/EtOAc, 5/1). Obtained as a ca. 12/1 mixture of *cis/trans* diastereoisomers and isolated as pure ***cis*-2n**. Data for the *cis* isomer: **^{1}H NMR** (300 MHz, $CDCl_3$): δ (ppm) = 8.27 (br s, 1H), 8.20 (s, 1H), 7.88 (add, *J* = 8.6, 1.5 Hz, 1H), 7.32 (ad, *J* = 8.6 Hz, 1H), 7.17–7.07 (m, 3H), 7.00–6.95 (m, 2H), 6.41 (ad, *J* = 1.5 Hz, 1H), 3.97 (s, 3H), 2.63–2.48 (m, 1H), 2.44–2.29 (m, 1H), 1.77–1.64 (m, 1H), 1.36–1.23 (m, 1H), 1.11–0.77 (m, 5H), 0.73–0.65 (m, 1H), 0.41–0.28 (m, 1H), 0.26–0.12 (m, 1H), 0.05– −0.07 (m, 2H). **^{13}C NMR** (75.4 MHz, $CDCl_3$): δ (ppm) = 168.7, 141.6, 140.2, 138.1, 128.6, 127.6, 127.4, 126.7, 125.3, 124.9, 124.0, 122.9, 121.3, 119.7, 110.6, 51.9, 25.2, 24.7, 14.3, 14.1, 13.8, 7.6, 6.7, 5.9, 5.7. **EI-LRMS** *m/z* (%): 397 (M+, 25), 293 (50), 207 (100). **HRMS** (ESI-TOF) *m/z*: calcd for $C_{27}H_{28}NO_2^+$ $[M+H]^+$ 398.2115; found 398.2123.

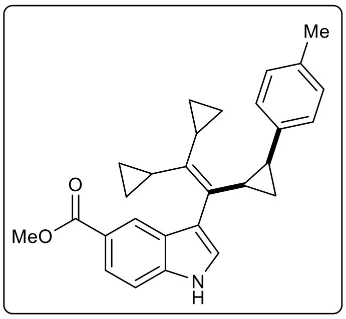

Methyl 3-(2,2-dicyclopropyl-1-((1*R,2*S**)-2-(*p*-tolyl)cyclopropyl)vinyl)-1*H*-indole-5-carboxylate (*cis*-2o):** Following the general procedure F1 with **1f** (147 mg, 0.5 mmol) and 1-methyl-4-vinylbenzene (0.75 mmol, 88 mg), the crude product was purified by column chromatography (hexane/EtOAc, 7/1) affording pure **2o** as a yellow oil (144 mg, 70%). R_f = 0.26 (hexane/EtOAc, 5/1). Obtained as a ca. 15/1 mixture and isolated as a ca. 10/1 mixture of *cis/trans* diastereoisomers. Data for the major diastereoisomer: **^{1}H NMR** (300 MHz, $CDCl_3$): δ (ppm) = 8.51 (br s, 1H), 8.17 (as, 1H), 7.90 (dd, *J* = 8.6, 1.6 Hz, 1H), 7.32 (ad, *J* = 8.6 Hz, 1H), 6.97–6.83 (m, 4H), 6.51 (d, *J* = 2.2 Hz, 1H), 3.99 (s, 3H), 2.62–2.48 (m, 1H), 2.42–2.32 (m, 1H), 2.28 (s, 3H), 1.83–1.66 (m, 1H), 1.38–1.18 (m, 1H), 1.13–1.03 (m, 1H), 1.02–0.90 (m, 2H), 0.90–0.78 (m, 1H), 0.77–0.61 (m, 2H), 0.43–0.30 (m, 1H), 0.29–0.12 (m, 1H), 0.07– −0.09 (m, 2H). **^{13}C NMR** (75.4 MHz, $CDCl_3$): δ (ppm) = 168.8, 141.2, 138.1, 136.8, 134.6, 128.3, 128.1, 127.5, 126.9, 124.9, 124.0, 122.7, 120.9, 119.5, 110.5, 51.9, 25.1, 24.2, 21.0, 14.3, 14.2, 13.5, 7.5, 6.6, 5.9, 5.6. **EI-LRMS** *m/z* (%): 411 (M^+, 11), 281 (100), 207 (51). **HRMS** (ESI-TOF) *m/z*: calcd for $C_{28}H_{30}NO_2^+$ $[M+H]^+$ 412.2271; found 412.2279.

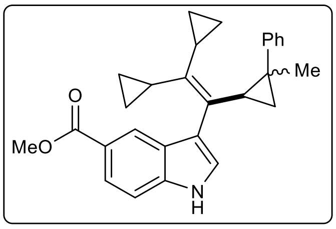

Methyl 3-(2,2-dicyclopropyl-1-(2-methyl-2-phenylcyclopropyl)vinyl)-1*H*-indole-5-carboxylate (2p): Following the general procedure F1 with **1f** (147 mg, 0.5 mmol) and α-methylstyrene (0.75 mmol, 88 mg), the crude product was purified by column chromatography (hexane/EtOAc, 7/1) affording pure **2p** as a brown oil (145 mg, 71%). R_f = 0.23 (hexane/EtOAc, 5/1). Obtained and isolated as a ca. 1.2/1 mixture of *cis/trans* diastereoisomers. Data for both diastereoisomers: **^{1}H NMR** (300 MHz, $CDCl_3$): δ (ppm) = 8.67 (br s, 1H), 8.41 (s, 1H), 8.18 (br s, 1H), 8.11 (s, 1H), 7.95 (dd, *J* = 8.6, 1.6 Hz, 1H), 7.86 (dd, *J* = 8.6, 1.6 Hz, 1H), 7.40 (ad, *J* = 8.6 Hz, 1H), 7.35–7.23 (m, 5H), 7.22–7.04 (m, 7H), 5.75 (as, 1H), 3.98 (s, 3H), 3.98 (s, 3H), 2.53–2.34 (m, 2H), 1.93–1.79 (m, 1H), 1.77–1.66 (m, 1H), 1.55 (s, 3H), 1.47–1.40 (m, 1H), 1.35 (s, 3H), 1.38–1.28 (m, 1H), 1.26–1.10 (m, 2H), 1.06–0.87 (m, 8H), 0.82–0.68 (m, 2H), 0.67–0.55 (m,

1H), 0.55–0.46 (m, 1H), 0.38–0.27 (m, 1H), 0.27–0.15 (m, 2H), 0.17–0.06 (m, 1H), 0.02– –0.12 (m, 2H). **^{13}C NMR** (75.4 MHz, $CDCl_3$): *δ* (ppm) = 168.75, 168.74, 147.8, 143.8, 142.1, 140.4, 138.5, 138.0, 129.2, 129.0, 128.2, 127.9, 127.8, 127.6, 127.5, 125.5, 125.3, 125.0, 124.8, 124.6, 124.0, 123.5, 123.1, 122.7, 121.4, 121.1, 119.8, 118.1, 110.8, 110.4, 52.0, 51.9, 34.0, 30.8, 28.9, 27.8, 25.9, 23.5, 19.6, 18.9, 15.1, 14.2, 14.0, 13.3, 8.3, 7.3, 6.2, 6.02, 5.97, 5.7, 5.3. One CH_2 were not observed due to overlapping. **EI-LRMS** *m/z* (%): 411 (M^+, 94), 316 (100), 207 (75). **HRMS** (ESI-TOF) *m/z*: calcd for $C_{28}H_{30}NO_2^+$ $[M+H]^+$ 412.2271; found 412.2274.

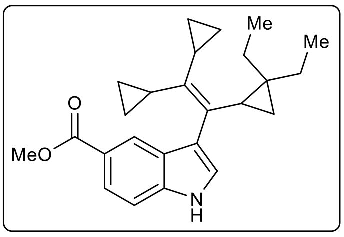

Methyl 3-(2,2-dicyclopropyl-1-(2,2-diethylcyclopropyl)vinyl)-1*H*-indole-5-carboxylate (2q): Following the general procedure F1 with **1f** (147 mg, 0.5 mmol) and 3-methylenepentane (0.75 mmol, 91 mg), the crude product was purified by column chromatography (hexane/EtOAc, 7/1) affording pure **2q** as a yellow solid (120 mg, 64%). R_f = 0.19 (hexane/EtOAc, 5/1); m.p. = 105–107 °C. **^{1}H NMR** (300 MHz, $CDCl_3$): *δ* (ppm) = 8.61 (br s, 1H), 8.34 (as, 1H), 7.90 (ad, *J* = 8.6 Hz, 1H), 7.35 (ad, *J* = 8.6 Hz, 1H), 6.96 (ad, *J* = 2.1 Hz, 1H), 3.95 (s, 3H), 2.06–1.84 (m, 2H), 1.67–1.51 (m, 1H), 1.45–1.03 (m, 6H), 0.95–0.81 (m, 6H), 0.81–0.66 (m, 2H), 0.62–0.50 (m, 1H), 0.47–0.27 (m, 1H), 0.21–0.13 (m, 2H), 0.06– –0.08 (m, 1H), –0.09– –0.32 (m, 1H). **^{13}C NMR** (75.4 MHz, $CDCl_3$): *δ* (ppm) = 168.9, 140.0, 138.4, 128.8, 127.8, 124.9, 124.1, 122.9, 121.2, 120.4, 110.7, 51.9, 29.5, 29.0, 28.8, 24.0, 18.3, 15.0, 13.4, 10.8, 10.3, 7.9, 6.4, 5.6, 5.2. **HRMS** (ESI-TOF) *m/z*: calcd for $C_{25}H_{32}NO_2^+$ $[M+H]^+$ 378.2428; found 378.2432.

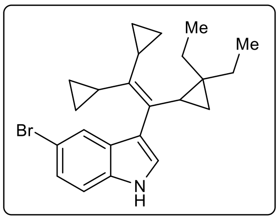

5-Bromo-3-(2,2-dicyclopropyl-1-(2,2-diethylcyclopropyl)vinyl)-1*H*-indole (2r): Following the general procedure F1 with **1g** (157 mg, 0.5 mmol) and 3-methylenepentane (0.75 mmol, 91 mg), the crude product was purified by column chromatography (hexane/EtOAc, 7/1) affording pure **2r** as a white oil (159 mg, 80%). R_f = 0.42 (hexane/EtOAc, 7/1). **^{1}H NMR** (300 MHz, $CDCl_3$): *δ* (ppm) = 8.03 (br s, 1H), 7.67 (as, 1H), 7.33–7.13 (m, 2H), 6.91 (ad, *J* = 2.2 Hz, 1H), 1.96–1.83 (m, 2H), 1.68–1.50 (m, 1H), 1.44–1.28 (m, 1H), 1.27–1.15 (m, 2H), 1.14–1.00 (m, 2H), 0.96–0.83 (m, 7H), 0.81–0.68 (m, 2H), 0.61–0.56 (m, 1H), 0.52–0.38 (m, 1H), 0.26–0.16 (m, 2H), 0.09– –0.08 (m, 1H), –0.10– –0.16 (m, 1H). **^{13}C NMR** (75.4 MHz, $CDCl_3$): *δ* (ppm) = 139.8, 134.3, 129.9, 128.8, 124.8, 124.4, 123.5, 118.9, 112.6, 112.3, 29.5, 29.0, 28.8, 24.0, 18.3, 15.0, 13.4, 10.8, 10.4, 7.9, 6.4, 5.6, 5.3. **EI-LRMS** *m/z* (%): 399 (M^++2, 35), 397 (M^+, 22), 304 (41), 95 (100). **HRMS** (APCI) *m/z*: calcd for $C_{23}H_{29}BrN^+$ $[M+H]^+$ 398.1477; found 398.1481.

Limitations on the scope of the intermolecular reaction

Once the reactivity of various terminal 3-propargylindoles with different olefins had been studied, our next objective was to extend this methodology to a related type of terminal substrates **S5a,b**, bearing an aromatic substituent at the propargylic position (R^2 = H). As shown in Table S3, when these 3-propargylindoles **S5a,b** were subjected to the previously optimized reaction conditions in the presence of styrene, the only products obtained were the corresponding 3-inden-2-yl indoles **S6**, arising from a tandem 1,2-indole migration / iso-

Nazarov cyclization (entries 1 and 2). This reactivity is consistent with our previous report for analogous substrates in the absence of external olefin.[15]

Table S3: Limitations of substrate scope.[a]

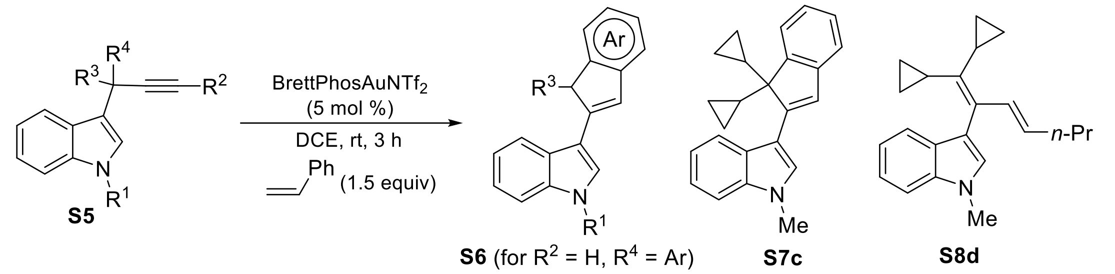

Entry	S5	R^1	R^2	R^3	R^4	Product	Yield (%)[b]
1	**S5a**	Me	H	H	2,4-$(MeO)_2C_6H_3$	**S6a**	32
2	**S5b**	H	H	*c*-C_3H_5	Ph	**S6b**	73
3[c]	**S5c**	Me	Ph	*c*-C_3H_5	*c*-C_3H_5	**S7c**	71
4[c]	**S5d**	Me	*n*-Bu	*c*-C_3H_5	*c*-C_3H_5	**S5d**	58
5[d]	**S5d**	Me	*n*-Bu	*c*-C_3H_5	*c*-C_3H_5	**S8d**	69

[a]Reaction conditions: **S5** (0.3 mmol), catalyst (5 mol%) in dichloroethane (2 mL) at rt for 3 h. [b]Isolated yield after column chromatography referred to the corresponding starting material **S5**. [c]Caried out at reflux for 16 h. [d]Carried out with $IPrAuNTf_2$.

We then explored the reactivity of 3-propargylindoles bearing a non-terminal triple bond ($R^2 \neq$ H). In this case, when **S5c**, bearing a phenyl substituent on the alkyne (R^2 = Ph), was used under the previously optimized conditions, but under reflux, indenylindole **S7c** was selectively obtained via a tandem 1,2-indole migration / Nazarov cyclization (entry 3). Finally, when **S5d**, possessing an alkyl substituent on the alkyne (R^2 = *n*-Bu) and no aromatic groups at the propargylic positions, was employed, the reaction does not proceed even under reflux (entry 4). However, using a more active catalyst, such as IPrAuNTf, led to 3-dienylindole **S8d** derived from a tandem 1,2-indole migration / 1,2-H migration. These results demonstrate that the tandem 1,2-indole migration / cyclopropanation sequence requires a terminal alkyne and the absence of aromatic groups at the propargylic position to be successful.

<u>*Characterization data:*</u>

Starting 3-propargylindoles **S5a-d**, 3-indenylindole **S7c**, and 3-dienylindole **S8d** have been reported in our previous works.[1,15]

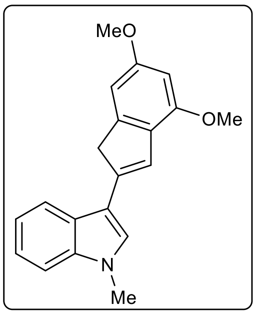

3-(4,6-Dimethoxy-1*H*-inden-2-yl)-1-methyl-1*H*-indole (S6a): Following the general procedure F1 with **S5a** (92 mg, 0.3 mmol), the crude product was purified by column chromatography (hexane/EtOAc, 7/1) affording pure **S6a** as a white solid (29 mg, 32%). R_f = 0.17 (hexane/EtOAc, 5/1); m.p. = 141–143 °C. **^{1}H NMR** (300 MHz, $CDCl_3$): *δ* (ppm) = 8.07 (ad, *J* = 7.2 Hz, 1H), 7.39–7.26 (m, 4H), 7.13 (as, 1H), 6.66 (ad, *J* = 1.9 Hz, 1H), 6.36 (ad, *J* = 1.9 Hz, 1H), 3.94 (s, 3H), 3.90 (s, 3H), 3.82 (s, 3H), 3.81 (ad, *J* = 0.9 Hz, 2H). **^{13}C NMR** (75.4 MHz, $CDCl_3$): *δ* (ppm) = 160.9, 155.4, 148.8, 142.8, 137.8,

[15] Sanz, R.; Miguel, D.; Gohain, M.; García-García, P.; Fernández-Rodríguez, M. A.; González-Pérez, A.; Nieto-Faza, O.; De Lera, A. R. *Chem. Eur. J.* **2010**, *16*, 9818–9828.

127.8, 126.1, 122.9, 122.3, 121.0, 120.40, 120.35, 113.0, 109.7, 98.0, 94.9, 55.8, 55.4, 37.3, 33.0. **EI-LRMS** *m/z* (%): 305 (M^+, 100), 290 (69), 247 (14). **HRMS** (ESI-TOF) *m/z*: calcd for $C_{20}H_{20}NO_2^+$ $[M+H]^+$ 306.1489; found, 306.1483.

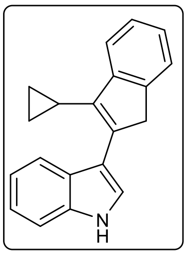

3-(3-Cyclopropyl-1*H*-inden-2-yl)-1*H*-indole (S6b): Following the general procedure F1 with **S5b** (81 mg, 0.3 mmol), the crude product was purified by flash column chromatography (hexane/EtOAc, 7/1) affording pure **S6b** as a colorless solid (58 mg, 73%). R_f = 0.24 (hexane/EtOAc, 5/1); m.p. = 137–139 °C. **^{1}H NMR** (300 MHz, $CDCl_3$): δ (ppm) = 8.18 (br s, 1H), 8.06 (ad, *J* = 7.5 Hz, 1H), 7.70 (ad, *J* = 7.5 Hz, 1H), 7.63–7.54 (m, 2H), 7.50–7.41 (m, 2H), 7.38–7.24 (m, 3H), 4.01 (s, 1H), 4.00 (s, 1H), 2.05–1.91 (m, 1H), 1.06–0.91 (m, 2H), 0.77–0.66 (m, 2H). **^{13}C NMR** (75.4 MHz, $CDCl_3$): δ (ppm) = 147.4, 142.6, 137.0, 136.7, 136.1, 126.7, 126.3, 124.3, 123.9, 123.4, 122.2, 121.1, 120.1, 119.6, 113.8, 111.4, 41.9, 9.1, 6.9. **EI-LRMS** *m/z* (%): 271 (M^+, 100), 270 (65), 256 (32). **HRMS** (ESI-TOF) *m/z*: calcd for $C_{20}H_{18}N^+$ $[M+H]^+$ 272.1434; found 272.1421.

Intramolecular cyclopropanation reactions of 2-alkenyl-3-propargylindoles 3: Synthesis and characterization data of cyclopenta[1,2-*b*]indole derivatives 4

General procedure G for the synthesis of tetrahydro-1H-cyclopropa[4,5]cyclopenta[1,2-b]indole derivatives 4

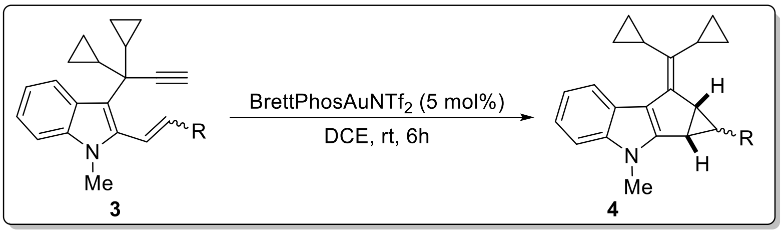

BrettPhosAuNTf$_2$ (21.6 mg, 0.025 mmol, 0.0 5 equiv) was dissolved in DCE (1 mL), and the resulting solution was stirred at rt for 5 min. A solution of the corresponding 3-propargyl indole **3** (0.5 mmol, 1 equiv) in DCE (19 mL, 0.025 M) was then added. The reaction mixture was stirred at rt for 6h (until complete consumption of the starting material, as determined by GC–MS or TLC). The mixture was filtered through a short pad of silica gel and celite using a hexane/EtOAc (10/1) mixture as eluent. The solvents were removed under reduced pressure, and the crude product was purified by flash column chromatography using mixtures of hexane/EtOAc as eluents, affording the corresponding cyclopenta[1,2-*b*]indole derivatives **4**.

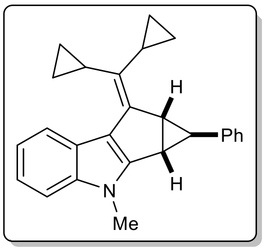

(1*R,1a*S**,7a*R**)-7-(Dicyclopropylmethylene)-2-methyl-1-phenyl-1a,2,7,7a-tetrahydro-1*H*-cyclopropa[4,5]cyclopenta[1,2-*b*]indole (*cis*-4a)**: Following the general procedure G with (*E*)-3-(1,1-dicyclopropylprop-2-yn-1-yl)-1-methyl-2-styryl-1*H*-indole ((***E***)-**3a**) (175 mg, 0.5 mmol), the crude product was purified by column chromatography (hexane/EtOAc, 10/1) affording pure ***cis*-4a** as a light yellow oil (123 mg, 70%). R_f = 0.30 (hexane/EtOAc, 10/1). **^{1}H NMR** (300 MHz, $CDCl_3$): δ (ppm) = 7.93 (ad, *J* = 7.9 Hz, 1H), 7.39–7.31 (m, 2H), 7.31–7.19 (m, 3H), 7.19–7.09 (m, 3H), 3.80 (s, 3H), 3.34–3.09 (m, 1H), 2.93–2.75 (m, 1H), 2.32–2.18 (m, 1H), 1.96–1.86 (m, 1H), 1.50–1.38 (m, 1H), 1.17–1.02 (m, 1H), 1.01–

0.86 (m, 1H), 0.87–0.75 (m, 3H), 0.68–0.47 (m, 2H), 0.42–0.25 (m, 1H). **^{13}C NMR** (75.4 MHz, $CDCl_3$): *δ* (ppm) = 152.5, 141.7, 141.3, 136.3, 128.5, 127.5, 125.9, 125.5, 123.6, 121.2, 120.7, 119.7, 116.5, 109.7, 39.6, 36.4, 31.0, 25.6, 16.8, 13.0, 6.9, 6.5, 5.5, 4.7. **EI-LRMS** *m/z* (%): 351 (M^+, 100), 207 (95), 144 (46). **HRMS** (ESI-TOF) *m/z*: calcd for $C_{26}H_{26}N^+$ $[M+H]^+$ 352.2060; found 352.2037.

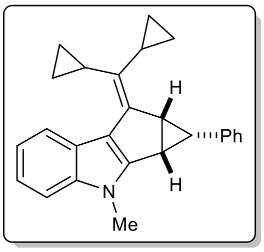

(1*S,1*aS**,7*aR**)-7-(Dicyclopropylmethylene)-2-methyl-1-phenyl-1*a*,2,7,7*a*-tetrahydro-1*H*-cyclopropa[4,5]cyclopenta[1,2-*b*]indole (*trans*-4a)**: Following the general procedure G with (*Z*)-3-(1,1-dicyclopropylprop-2-yn-1-yl)-1-methyl-2-styryl-1*H*-indole ((***Z***)-**3a**) (175 mg, 0.5 mmol), the crude product was purified by column chromatography (hexane/EtOAc, 10/1) affording pure ***trans*-4a** as a light yellow oil (117 mg, 67%). R_f = 0.25 (hexane/EtOAc, 10/1). **^{1}H NMR** (300 MHz, $CDCl_3$): *δ* (ppm) = 7.71 (ad, *J* = 8.0 Hz, 1H), 7.13 (ad, *J* = 7.7 Hz, 1H), 7.06 (at, *J* = 7.7 Hz, 1H), 6.99–6.88 (m, 6H), 3.80 (s, 3H), 3.44–3.28 (m, 1H), 3.02–2.87 (m, 1H), 2.76–2.71 (m, 1H), 2.12–1.96 (m, 1H), 1.67–1.56 (m, 1H), 1.12–0.98 (m, 1H), 0.96–0.69 (m, 5H), 0.58–0.48 (m, 2H). **^{13}C NMR** (125 MHz, $CDCl_3$): *δ* (ppm) = 149.3, 141.0, 136.4, 133.9, 129.6, 129.2, 127.5, 125.9, 123.2, 121.2, 120.2, 119.2, 118.0, 109.1, 33.9, 32.2, 30.9, 21.2, 16.2, 13.8, 7.6, 6.6, 6.0, 5.6. **EI-LRMS** *m/z* (%): 351 (M^+, 100), 350 (23), 349 (37). **HRMS** (ESI-TOF) *m/z*: calcd for $C_{26}H_{26}N^+$ $[M+H]^+$ 352.2060; found 352.2037.

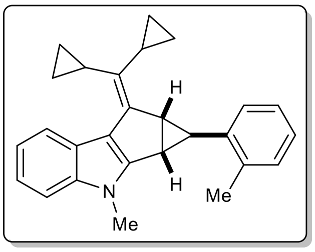

(1*R,1*aS**,7*aR**)-7-(Dicyclopropylmethylene)-2-methyl-1-(*o*-tolyl)-1*a*,2,7,7*a*-tetrahydro-1*H*-cyclopropa[4,5]cyclopenta[1,2-*b*]indole (*cis*-4b)**: Following the general procedure G with (*E*)-3-(1,1-dicyclopropylprop-2-yn-1-yl)-1-methyl-2-(2-methylstyryl)-1*H*-indole ((***E***)-**3b**) (182 mg, 0.5 mmol), but the reaction mixture was stirred at rt for 24 h. The crude product was purified by column chromatography (hexane/EtOAc, 10/1) affording pure ***cis*-4b** as a light orange oil (123 mg, 64%). R_f = 0.29 (hexane/EtOAc, 10/1). **^{1}H NMR** (300 MHz, $CDCl_3$): *δ* (ppm) = 7.98 (ad, *J* = 7.9 Hz, 1H), 7.38–6.95 (m, 7H), 3.85 (s, 3H), 3.56–3.30 (m, 1H), 2.81–2.63 (m, 1H), 2.41 (s, 3H), 2.37–2.21 (m, 1H), 2.13–1.96 (m, 1H), 1.67–1.49 (m, 1H), 1.17–1.05 (m, 1H), 1.05–0.83 (m, 4H), 0.83–0.61 (m, 2H), 0.50–0.26 (m, 1H). **^{13}C NMR** (75.4 MHz, $CDCl_3$): *δ* (ppm) = 152.8, 141.3, 139.0, 137.0, 136.5, 130.0, 127.4, 126.2, 126.1, 124.2, 123.7, 121.3, 120.7, 119.7, 116.7, 109.6, 37.3, 34.0, 31.0, 24.8, 20.0, 16.8, 13.2, 7.0, 6.8, 5.8, 4.9. **EI-LRMS** *m/z* (%): 365 (M^+, 100), 337 (45), 322 (38). **HRMS** (ESI-TOF) *m/z*: calcd for $C_{27}H_{27}NNa^+$ $[M+Na]^+$ 388.2036; found 388.2026.

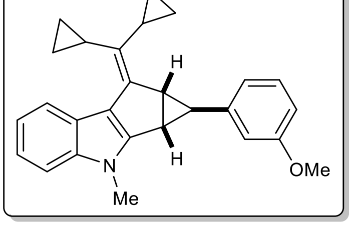

(1*R,1*aS**,7*aR**)-7-(Dicyclopropylmethylene)-1-(3-methoxyphenyl)-2-methyl-1*a*,2,7,7*a*-tetrahydro-1*H*-cyclopropa[4,5]cyclopenta[1,2-*b*]indole (*cis*-4c):** Following the general procedure G with (*E*)-3-(1,1-dicyclopropylprop-2-yn-1-yl)-2-(3-methoxystyryl)-1-methyl-1*H*-indole ((***E***)-**3c**) (190 mg, 0.5 mmol), the crude product was purified by column chromatography (hexane/EtOAc, 10/1) affording pure ***cis*-4c** as a light brown solid (126 mg, 66%). R_f = 0.18 (hexane/EtOAc, 10/1); m.p. = 157–159 °C. **^{1}H NMR** (300 MHz, $(CD_3)_2CO$): *δ* (ppm) = 7.94 (ad, *J* = 7.9 Hz, 1H), 7.33–7.25 (m, 2H), 7.25–7.17 (m, 1H), 7.13 (at, *J* = 7.3 Hz, 1H), 6.84–6.67 (m, 3H), 3.86 (s, 3H), 3.80 (s, 3H), 3.24–3.15 (m, 1H), 2.90–2.71 (m, 1H), 2.33–2.12 (m, 1H), 1.92–1.82 (m, 1H), 1.53–1.38 (m, 1H), 1.15–1.01 (m, 1H), 1.00–0.90 (m, 1H), 0.88–0.72 (m, 3H), 0.65–0.56 (m, 2H), 0.43–0.31

(m, 1H). **^{13}C NMR** (75.4 MHz, $(CD_3)_2CO$): δ (ppm) = 159.8, 152.4, 143.4, 141.3, 136.2, 129.5, 127.6, 123.6, 121.2, 120.7, 119.7, 118.0, 116.5, 111.5, 111.0, 109.7, 55.3, 39.7, 36.4, 31.0, 25.6, 16.8, 13.0, 6.9, 6.5, 5.6, 4.7. **EI-LRMS** *m/z* (%): 381 (M^+, 31), 240 (65), 165 (100). **HRMS** (ESI-TOF) *m/z*: calcd for $C_{27}H_{28}NO^+$ $[M+H]^+$ 382.2165; found 382.2147.

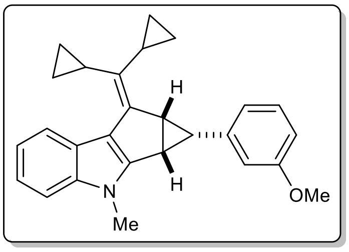

(1*S,1*aS**,7*aR**)-7-(Dicyclopropylmethylene)-1-(3-methoxyphenyl)-2-methyl-1*a*,2,7,7*a*-tetrahydro-1*H*-cyclopropa[4,5]cyclopenta[1,2-*b*]indole (*trans*-4c):** Following the general procedure G with (*Z*)-3-(1,1-dicyclopropylprop-2-yn-1-yl)-2-(3-methoxystyryl)-1-methyl-1*H*-indole ((***Z***)-**3c**) (190 mg, 0.5 mmol), the crude product was purified by column chromatography (hexane/EtOAc, 10/1) affording pure ***trans*-4c** as a light brown solid (104 mg, 55%). R_f = 0.13 (hexane/EtOAc, 10/1); m.p. = 113–115 °C. **^{1}H NMR** (300 MHz, $CDCl_3$): δ (ppm) = 7.76 (ad, *J* = 8.0 Hz, 1H), 7.14 (ad, *J* = 8.0 Hz, 1H), 7.08 (at, *J* = 7.4 Hz, 1H), 6.99 (at, *J* = 7.4 Hz, 1H), 6.90 (at, *J* = 8.0 Hz, 1H), 6.62 (ad, *J* = 7.5 Hz, 1H), 6.47 (ad, *J* = 8.0 Hz, 1H), 6.37 (as, 1H), 3.77 (s, 3H), 3.45 (s, 3H), 3.41–3.32 (m, 1H), 3.02–2.92 (m, 1H), 2.71 (t, *J* = 7.9 Hz, 1H), 2.23–2.04 (m, 1H), 1.70–1.51 (m, 1H), 1.08–0.91 (m, 2H), 0.91–0.74 (m, 4H), 0.71–0.55 (m, 1H), 0.5–0.17 (m, 1H). **^{13}C NMR** (125 MHz, $CDCl_3$): δ (ppm) = 158.9, 149.0, 141.2, 138.2, 133.8, 129.2, 128.3, 123.2, 122.3, 121.2, 120.4, 119.3, 118.2, 113.6, 112.7, 109.2, 55.0, 33.5, 32.3, 30.8, 21.7, 16.5, 13.6, 7.4, 6.5, 5.8, 5.5. **EI-LRMS** *m/z* (%): 381 (M^+, 27), 352 (30). **HRMS** (ESI-TOF) *m/z*: calcd for $C_{27}H_{27}NONa^+$ $[M+Na]^+$ 404.1985; found 404.1988.

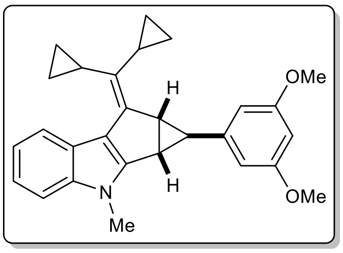

(1*R,1*aS**,7*aR**)-7-(Dicyclopropylmethylene)-1-(3,5-dimethoxyphenyl)-2-methyl-1*a*,2,7,7*a*-tetrahydro-1*H*-cyclopropa[4,5]cyclopenta[1,2-*b*]indole (*cis*-4d)**: Following the general procedure G with (*E*)-3-(1,1-dicyclopropylprop-2-yn-1-yl)-2-(3,5-dimethoxystyryl)-1-methyl-1*H*-indole ((***E***)-**3d**) (205 mg, 0.5 mmol), the crude product was purified by column chromatography (hexane/EtOAc, 10/1) affording pure ***cis*-4d** as a light yellow oil (141 mg, 69%). R_f = 0.24 (hexane/EtOAc, 10/1). **^{1}H NMR** (300 MHz, $CDCl_3$): δ (ppm) = 7.96 (ad, *J* = 7.8 Hz, 1H), 7.29 (ad, *J* = 7.8 Hz, 1H), 7.25–7.19 (m, 1H), 7.19–7.12 (m, 1H), 6.42–6.38 (m, 1H), 6.38–6.31 (m, 2H), 3.86 (s, 6H), 3.81 (s, 3H), 3.22 (dd, *J* = 6.0, 3.3 Hz, 1H), 2.86 (dd, *J* = 6.0, 3.3 Hz, 1H), 2.36–2.14 (m, 1H), 1.89 (t, *J* = 3.3 Hz, 1H), 1.55–1.43 (m, 1H), 1.20–1.05 (m, 1H), 1.04–0.92 (m, 1H), 0.92–0.78 (m, 3H), 0.71–0.61 (m, 2H), 0.45–0.33 (m, 1H). **^{13}C NMR** (75.4 MHz, $CDCl_3$): δ (ppm) = 160.9, 152.3, 144.2, 141.3, 136.2, 127.6, 123.6, 121.1, 120.7, 119.7, 116.4, 109.6, 103.8, 97.6, 55.4, 39.9, 36.3, 31.0, 25.6, 16.8, 13.0, 6.9, 6.5, 5.6, 4.7. **EI-LRMS** *m/z* (%): 411 (M^+, 100), 330 (69), 274 (32). **HRMS** (ESI-TOF) *m/z*: calcd for $C_{28}H_{29}NO_2Na^+$ $[M+Na]^+$ 434.2091; found 434.2089.

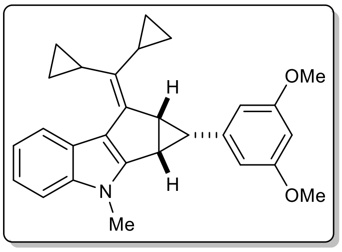

(1*S,1*aS**,7*aR**)-7-(Dicyclopropylmethylene)-1-(3,5-dimethoxyphenyl)-2-methyl-1*a*,2,7,7*a*-tetrahydro-1*H*-cyclopropa[4,5]cyclopenta[1,2-*b*]indole (*trans*-4d)**: Following the general procedure G with 3-(1,1-dicyclopropylprop-2-yn-1-yl)-2-(3,5-dimethoxystyryl)-1-methyl-1*H*-indole (**3d**) as a ca. 1/8 mixture of *E/Z* isomers (205 mg, 0.5 mmol), the crude product was purified by column chromatography (hexane/EtOAc, 10/1) affording pure ***trans*-4d** as a light yellow oil (143 mg, 70%). R_f = 0.23 (hexane/EtOAc, 10/1). Obtained as a ca. 1/7.5 mixture of *cis/trans*

diastereoisomers and isolated as pure ***trans*-4d**: **^{1}H NMR** (300 MHz, $CDCl_3$): δ (ppm) = 7.80 (ad, *J* = 8.0 Hz, 1H), 7.18–7.08 (m, 2H), 7.06–6.98 (m, 1H), 6.14–6.01 (m, 3H), 3.76 (s, 3H), 3.50 (s, 6H), 3.35 (dd, *J* = 7.7, 5.9 Hz, 1H), 2.99 (dd, *J* = 7.7, 5.9 Hz, 1H), 2.67 (t, *J* = 7.7 Hz, 1H), 2.26–2.12 (m, 1H), 1.67–1.49 (m, 1H), 1.08–0.92 (m, 2H), 0.92–0.76 (m, 4H), 0.73–0.62 (m, 1H), 0.51–0.40 (m, 1H). **^{13}C NMR** (75.4 MHz, $CDCl_3$): δ (ppm) = 159.9, 148.8, 141.2, 139.0, 133.8, 129.0, 123.2, 121.2, 120.4, 119.4, 118.3, 109.3, 107.0, 99.2, 55.0, 33.5, 32.4, 30.8, 21.9, 16.6, 13.4, 7.3, 6.4, 5.6, 5.4. **EI-LRMS** *m/z* (%): 411 (M^+, 100), 330 (56), 115 (43). **HRMS** (ESI-TOF) *m/z*: calcd for $C_{28}H_{29}NO_2Na^+$ $[M+Na]^+$ 434.2091; found 434.2095.

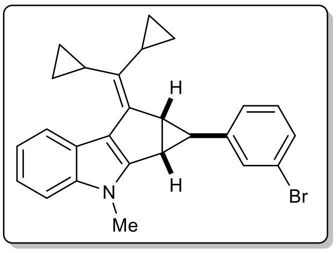

(1*R,1*aS**,7a*R**)-1-(3-Bromophenyl)-7-(dicyclopropylmethylene)-2-methyl-1*a*,2,7,7*a*-tetrahydro-1*H*-cyclopropa[4,5]cyclopenta[1,2-*b*]indole (*cis*-4e)**: Following the general procedure G with (*E*)-2-(3-bromostyryl)-3-(1,1-dicyclopropylprop-2-yn-1-yl)-1-methyl-1*H*-indole ((***E***)**-3e**) (215 mg, 0.5 mmol), the crude product was purified by column chromatography (hexane/EtOAc, 10/1) affording pure ***cis*-4e** as a light brown solid (146 mg, 68%). R_f = 0.29 (hexane/EtOAc, 10/1); m.p. = 142–144 °C. **^{1}H NMR** (300 MHz, $CDCl_3$): δ (ppm) = 7.95 (ad, *J* = 7.9 Hz, 1H), 7.37 (ad, *J* = 7.9 Hz, 1H), 7.32–7.26 (m, 2H), 7.25–7.19 (m, 2H), 7.18–7.11 (m, 1H), 7.07 (ad, *J* = 7.8 Hz, 1H), 3.80 (s, 3H), 3.31–3.05 (m, 1H), 2.90–2.76 (m, 1H), 2.36–2.20 (m, 1H), 1.93–1.83 (m, 1H), 1.54–1.41 (m, 1H), 1.18–1.03 (m, 1H), 1.03–0.90 (m, 1H), 0.88–0.72 (m, 3H), 0.72–0.55 (m, 2H), 0.42–0.31 (m, 1H). **^{13}C NMR** (75.4 MHz, $CDCl_3$): δ (ppm) = 152.0, 144.2, 141.3, 135.9, 130.0, 129.0, 128.3, 128.0, 124.2, 123.5, 122.8, 121.2, 120.9, 119.8, 116.6, 109.7, 38.9, 36.6, 31.0, 25.8, 16.9, 13.0, 7.0, 6.5, 5.6, 4.7. **EI-LRMS** *m/z* (%): 431 (M^++2, 25), 429 (M^+, 28) 281 (100), 231 (95). **HRMS** (ESI-TOF) *m/z*: calcd for $C_{26}H_{25}NBr^+$ $[M-H+H]^+$ 429.1087; found 429.1093.

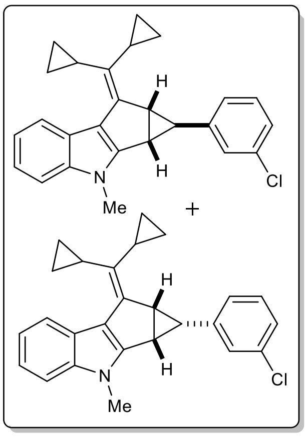

1-(3-Chlorophenyl)-7-(dicyclopropylmethylene)-2-methyl-1*a*,2,7,7*a*-tetrahydro-1*H*-cyclopropa[4,5]cyclopenta[1,2-*b*]indole (4f): Following the general procedure G with 2-(3-chlorostyryl)-3-(1,1-dicyclopropylprop-2-yn-1-yl)-1-methyl-1*H*-indole (**3f**) as a ca. 1/2 mixture of *E*/*Z* diastereoisomers (193 mg, 0.5 mmol), the crude product was obtained as a ca. 1/1.5 mixture of *cis*/*trans* diastereoisomers and was purified by column chromatography affording pure ***cis*-4f** (50 mg, 26%) and ***trans*-4f** (84 mg, 44%), which were isolated independently.

(1*R,1*aS**,7*aR**)-1-(3-Chlorophenyl)-7-(dicyclopropylmethylene)-2-methyl-1*a*,2,7,7*a*-tetrahydro-1*H*-cyclopropa[4,5]cyclopenta[1,2-*b*]indole (*cis*-4f)**: Light yellow oil. R_f = 0.24 (hexane/EtOAc, 10/1). **^{1}H NMR** (500 MHz, $CDCl_3$): δ (ppm) = 7.90 (ad, *J* = 8.0 Hz, 1H), 7.26 (ad, *J* = 8.0 Hz, 1H), 7.23 (ad, *J* = 7.7 Hz, 1H), 7.21–7.15 (jm, 2H), 7.13–7.10 (m, 1H), 7.10–7.06 (m, 1H), 7.02–6.98 (m, 1H), 3.80 (s, 3H), 3.19–3.10 (m, 1H), 2.89–2.77 (m, 1H), 2.27–2.15 (m, 1H), 1.91–1.77 (m, 1H), 1.46–1.39 (m, 1H), 1.11–1.01 (m, 1H), 0.94–0.86 (m, 1H), 0.84–0.80 (m, 1H), 0.80–0.76 (m, 1H), 0.76–0.66 (m, 1H), 0.66–0.61 (m, 1H), 0.61–0.51 (m, 1H), 0.35–0.29 (m, 1H). **^{13}C NMR** (125 MHz, $CDCl_3$): δ (ppm) = 152.0, 143.9, 141.3, 135.9, 134.5, 129.8, 128.1, 126.1, 125.4, 123.8, 123.6, 121.2, 120.9, 119.8, 116.6, 109.7, 39.0, 36.6, 31.1, 25.8, 16.9, 13.0, 7.0, 6.6, 5.6, 4.7. **EI-LRMS** *m/z* (%): 385 (M^+, 25), 218 (65), 207 (100). **HRMS** (ESI-TOF) *m/z*: calcd for $C_{26}H_{24}NClNa^+$ $[M+Na]^+$ 408.1489; found 408.1482.

(1*S,1*aS**,7*aR**)-1-(3-Chlorophenyl)-7-(dicyclopropylmethylene)-2-methyl-1*a*,2,7,7*a*-tetrahydro-1*H*-cyclopropa[4,5]cyclopenta[1,2-*b*]indole (*trans*-4f)**: Light pink oil. R_f = 0.20 (hexane/EtOAc, 10/1). **^{1}H NMR** (300 MHz, $CDCl_3$): δ (ppm) = 7.73 (ad, *J* = 8.0 Hz, 1H), 7.15 (ad, *J* = 8.0 Hz, 1H), 7.08 (at, *J* = 7.5 Hz, 1H), 7.02–6.94 (m, 2H), 6.89–6.81 (m, 2H), 6.79–6.73 (m, 1H), 3.81 (s, 3H), 3.46–3.30 (m, 1H), 3.01–2.88 (m, 1H), 2.68 (t, *J* = 7.5 Hz, 1H), 2.11–1.92 (m, 1H), 1.73–1.56 (m, 1H), 1.06–0.95 (m, 1H), 0.93–0.75 (m, 6H), 0.64–0.52 (m, 2H). **^{13}C NMR** (75.4 MHz, $CDCl_3$): δ (ppm) = 148.9, 141.1, 138.5, 133.4, 133.1, 130.1, 129.8, 128.7, 127.6, 126.1, 123.0, 121.3, 120.4, 119.3, 118.0, 109.2, 33.3, 32.2, 30.9, 21.2, 16.2, 13.9, 7.8, 6.6, 6.1, 5.8. **EI-LRMS** *m/z* (%): 385 (M^+, 21), 212 (23), 145 (100). **HRMS** (ESI-TOF) *m/z*: calcd for $C_{26}H_{24}NClNa^+$ $[M+Na]^+$ 408.1489; found 408.1491.

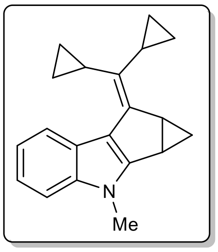

7-(Dicyclopropylmethylene)-2-methyl-1*a*,2,7,7*a*-tetrahydro-1*H*-cyclopropa[4,5]cyclopenta[1,2-*b*]indole (4g): Following the general procedure G with 3-(1,1-dicyclopropylprop-2-yn-1-yl)-1-methyl-2-vinyl-1*H*-indole (**3g**) (137 mg, 0.5 mmol), the crude product was purified by column chromatography (hexane/EtOAc, 10/1) affording pure **4g** as a light yellow solid (92 mg, 67%). R_f = 0.44 (hexane/EtOAc, 10/1); m.p. = 135–137 °C. **^{1}H NMR** (300 MHz, $(CD_3)_2CO$): δ (ppm) = 7.80 (ad, *J* = 8.0 Hz, 1H), 7.29 (ad, *J* = 8.0 Hz, 1H), 7.07 (at, *J* = 7.0 Hz, 1H), 6.99 (at, *J* = 7.0 Hz, 1H), 3.82 (s, 3H), 2.81–2.72 (m, 1H), 2.64–2.54 (m, 1H), 2.23–2.12 (m, 1H), 1.51–1.39 (m, 1H), 1.31–1.21 (m, 1H), 1.06–0.93 (m, 1H), 0.92–0.80 (m, 2H), 0.80–0.60 (m, 4H), 0.40–0.33 (m, 1H), 0.33–0.22 (m, 1H). **^{13}C NMR** (75.4 MHz, $(CD_3)_2CO$): δ (ppm) = 154.0 (C), 141.1 (C), 136.9 (C), 125.3 (C), 123.8 (C), 120.6 (CH), 120.1 (CH), 119.3 (CH), 114.8 (C), 109.6 (CH), 30.0 (CH_3), 24.0 (CH), 21.5 (CH_2), 16.5 (CH), 14.2 (CH), 12.5 (CH), 6.1 (CH_2), 5.8 (CH_2), 4.9 (CH_2), 4.5 (CH_2). **EI-LRMS** *m/z* (%): 275 (M^+, 100), 260 (80), 181 (32). **HRMS** (ESI-TOF) *m/z*: calcd for $C_{20}H_{22}N^+$ $[M+H]^+$ 276.1747; found 276.1729.

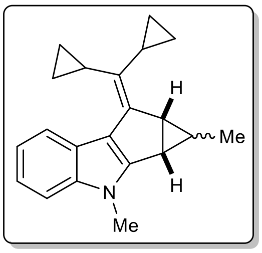

(1*aS,7*aR**)-7-(Dicyclopropylmethylene)-1,2-dimethyl-1*a*,2,7,7*a*-tetrahydro-1*H*-cyclopropa[4,5]cyclopenta[1,2-*b*]indole (4h)**: Following the general procedure G with 3-(1,1-dicyclopropylprop-2-yn-1-yl)-1-methyl-2-(prop-1-en-1-yl)-1*H*-indole (**3h**) as a ca. 1.4/1 mixtured of *E*/*Z* diastereoisomers (144 mg, 0.5 mmol), the crude product was purified by column chromatography (hexane/EtOAc, 10/1) affording pure **4h** as a light orange oil (117 mg, 81%). R_f = 0.29 (hexane/EtOAc, 10/1). Obtained and isolated as a ca. 1.2/1 mixture of *cis*/*trans* diastereoisomers. Data for both diastereoisomers: **^{1}H NMR** (300 MHz, $CDCl_3$): δ (ppm) = 7.93 (ad, *J* = 8.0 Hz, 1H), 7.89 (ad, *J* = 8.0 Hz, 1H), 7.29–7.22 (m, 2H), 7.21–7.15 (m, 2H), 7.15–7.06 (m, 2H), 3.80 (s, 3H), 3.78 (s, 3H), 2.91–2.82 (m, 1H), 2.65–2.54 (m, 2H), 2.36–2.15 (m, 3H), 1.55–1.39 (m, 3H), 1.26 (d, *J* = 6.1 Hz, 3H), 1.15–1.00 (m, 2H), 0.97–0.85 (m, 4H), 0.85–0.65 (m, 9H), 0.53 (d, *J* = 6.2 Hz, 3H), 0.43–0.28 (m, 2H). **^{13}C NMR** (75.4 MHz, $CDCl_3$): δ (ppm) = 153.5, 149.4, 141.3, 141.1, 136.8, 133.4, 129.0, 125.6, 123.7, 122.9, 121.2, 121.0, 120.34, 120.28, 119.5, 119.4, 118.5, 115.9, 109.42, 109.37, 33.8, 30.9, 30.8, 30.5, 23.6, 22.6, 19.8, 17.3, 16.9, 16.8, 12.9, 7.8, 7.1, 6.7, 6.6, 6.5, 5.8, 5.7, 5.4, 4.5. **EI-LRMS** *m/z* (%): 289 (M^+, 79), 274 (100), 208 (83). **HRMS** (ESI-TOF) *m/z*: calcd for $C_{21}H_{24}N^+$ $[M+H]^+$ 290.1903; found 290.1893.

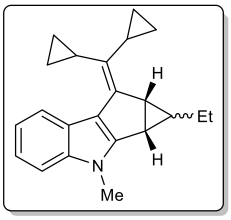

(1*aS,7*aR**)-7-(Dicyclopropylmethylene)-1-ethyl-2-methyl-1*a*,2,7,7*a*-tetrahydro-1*H*-cyclopropa[4,5]cyclopenta[1,2-*b*]indole (4i)**: Following the general procedure G with 2-(but-1-en-1-yl)-3-(1,1-dicyclopropylprop-2-yn-1-yl)-1-methyl-1*H*-indole (**3i**) as a ca. 1.7/1 mixture of *E/Z* diastereoisomers (152 mg, 0.5 mmol), the crude product was purified by column chromatography (hexane/EtOAc, 10/1) affording pure **4i** as a light orange oil (93 mg, 61%). R_f = 0.34 (hexane/EtOAc, 10/1). Obtained as a ca. 1.8/1 mixture and isolated as a ca. 2.8/1 mixture of *cis/trans* diastereoisomers. Data for the major diastereoisomer: **^{1}H NMR** (300 MHz, $CDCl_3$): *δ* (ppm) = 7.91 (ad, *J* = 7.9 Hz, 1H), 7.27–7.21 (m, 1H), 7.19–7.13 (m, 1H), 7.13–7.04 (m, 1H), 3.78 (s, 3H), 2.95–2.82 (m, 1H), 2.63–2.52 (m, 1H), 2.28–2.17 (m, 1H), 1.49–1.29 (m, 3H), 1.14–0.97 (m, 2H), 0.95–0.82 (m, 2H), 0.83–0.64 (m, 6H), 0.61–0.51 (m, 1H), 0.40–0.27 (m, 1H). **^{13}C NMR** (75.4 MHz, $CDCl_3$): *δ* (ppm) = 149.4, 141.2, 133.6, 128.6, 122.9, 121.3, 120.3, 119.4, 118.5, 109.4, 31.9, 30.8, 30.6, 19.1, 16.8, 16.0, 13.8, 13.1, 7.2, 6.6, 5.6, 5.4. **EI-LRMS** *m/z* (%): 303 (M^+, 89), 274 (100), 232 (47). **HRMS** (ESI-TOF) calcd for $C_{22}H_{26}N^+$ $[M+H]^+$, 304.2060; found, 304.2049.

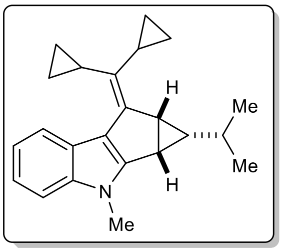

(1*S,1*aS**,7*aR**)-7-(Dicyclopropylmethylene)-1-isopropyl-2-methyl-1*a*,2,7,7*a*-tetrahydro-1*H*-cyclopropa[4,5]cyclopenta[1,2-*b*]indole (*trans*-4j)**: Following the general procedure G with 3-(1,1-dicyclopropylprop-2-yn-1-yl)-1-methyl-2-(3-methylbut-1-en-1-yl)-1*H*-indole (**3j**) as a ca. 1/8 mixture of *E/Z* diastereoisomers (158 mg, 0.5 mmol), for 48 h, the crude product was purified by column chromatography (hexane/EtOAc, 10/1) affording pure ***trans*-4j** as a light orange oil (130 mg, 82%). R_f = 0.32 (hexane/EtOAc, 10/1). Obtained as a ca. 1/10 mixture of *cis/trans* diastereoisomers and isolated as pure ***trans*-4j**: **^{1}H NMR** (300 MHz, $CDCl_3$): *δ* (ppm) = 7.91 (ad, *J* = 7.7 Hz, 1H), 7.24 (ad, *J* = 7.7 Hz, 1H), 7.20–7.12 (m, 1H), 7.12–7.04 (m, 1H), 3.78 (s, 3H), 3.03–2.93 (m, 1H), 2.66–2.46 (m, 1H), 2.27–2.02 (m, 1H), 1.53–1.42 (m, 1H), 1.20–1.05 (m, 2H), 1.05–0.91 (m, 3H), 0.91–0.85 (m, 3H), 0.85–0.78 (m, 1H), 0.78–0.61 (m, 6H), 0.46–0.39 (m, 1H). **^{13}C NMR** (75.4 MHz, $CDCl_3$): *δ* (ppm) = 149.7, 141.1, 133.4, 128.6, 123.0, 121.4, 120.3, 119.4, 118.5, 109.4, 39.0, 30.82, 30.77, 23.0, 22.4, 21.9, 19.3, 16.2, 13.6, 7.6, 6.7, 5.7, 5.3. **EI-LRMS** *m/z* (%): 317 (M^+, 30), 275 (21), 274 (100). **HRMS** (ESI-TOF) *m/z*: calcd for $C_{23}H_{28}N^+$ $[M+H]^+$ 318.2216; found 318.2219.

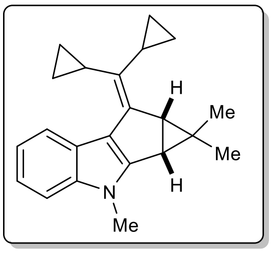

7-(Dicyclopropylmethylene)-1,1,2-trimethyl-1*a*,2,7,7*a*-tetrahydro-1*H*-cyclopropa[4,5]cyclopenta[1,2-*b*]indole (4k): Following the general procedure G with 3-(1,1-dicyclopropylprop-2-yn-1-yl)-1-methyl-2-(2-methylprop-1-en-1-yl)-1*H*-indole (**3k**) (151 mg, 0.5 mmol), the crude product was purified by column chromatography (hexane/EtOAc, 10/1) affording pure **4k** as a light yellow oil (102 mg, 67%). R_f = 0.35 (hexane/EtOAc, 10/1). **^{1}H NMR** (300 MHz, $CDCl_3$): *δ* (ppm) = 7.91 (ad, *J* = 7.9 Hz, 1H), 7.24 (ad, *J* = 7.9 Hz, 1H), 7.16 (at, *J* = 7.0 Hz, 1H), 7.08 (at, *J* = 7.0 Hz, 1H), 3.76 (s, 3H), 2.67 (d, *J* = 5.9 Hz, 1H), 2.32 (d, *J* = 5.9 Hz, 1H), 2.30–2.16 (m, 1H), 1.47–1.32 (m, 1H), 1.28 (s, 3H), 1.13–0.99 (m, 1H), 0.97–0.71 (m, 4H), 0.71–0.62 (m, 2H), 0.57 (s, 3H), 0.42–0.21 (m, 1H). **^{13}C NMR** (75.4 MHz, $CDCl_3$): *δ* (ppm) = 150.7, 141.3, 134.6, 128.3, 123.1, 121.3, 120.4, 119.5, 118.9, 109.4, 39.7, 30.8, 30.0, 27.6, 26.3, 16.9, 15.0, 12.9, 7.0, 6.7, 6.0, 4.7. **EI-LRMS** *m/z* (%): 303 (M^+, 29), 288 (100), 208 (51). **HRMS** (ESI-TOF) *m/z*: calcd for $C_{22}H_{26}N^+$ $[M+H]^+$ 304.2060; found 304.2052.

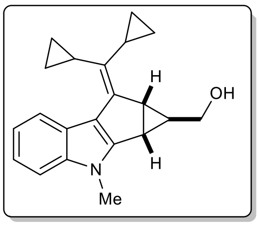

((1*R,1*aS**,7*aR**)-7-(Dicyclopropylmethylene)-2-methyl-1*a*,2,7,7*a*-tetrahydro-1*H*-cyclopropa[4,5]cyclopenta[1,2-*b*]indol-1-yl)methanol (*cis*-4l)**: Following the general procedure G with (*E*)-3-(3-(1,1-dicyclopropylprop-2-yn-1-yl)-1-methyl-1*H*-indol-2-yl)prop-2-en-1-ol ((***E***)**-3l**) (150 mg, 0.5 mmol), for 8 h, the crude product was purified by column chromatography (hexane/EtOAc, 10/1) affording pure ***cis*-4l** as a light yellow oil (52 mg, 34%). R_f = 0.29 (hexane/EtOAc, 10/1). **^{1}H NMR** (300 MHz, $CDCl_3$): δ (ppm) = 7.88 (ad, *J* = 7.5 Hz, 1H), 7.24 (ad, *J* = 7.5 Hz, 1H), 7.16 (at, *J* = 7.5 Hz, 1H), 7.09 (at, *J* = 7.5 Hz, 1H), 3.87–3.70 (m, 1H), 3.78 (s, 3H), 3.67–3.48 (m, 1H), 2.82–2.71 (m, 1H), 2.51–2.35 (m, 1H), 2.26–2.13 (m, 1H), 1.68 (br s, 1H), 1.54–1.39 (m, 1H), 1.25–1.13 (m, 1H), 1.10–0.98 (m, 1H), 0.98–0.64 (m, 6H), 0.42–0.30 (m, 1H). **^{13}C NMR** (75.4 MHz, $CDCl_3$): δ (ppm) = 152.1, 141.1, 135.8, 127.0, 123.6, 121.1, 120.6, 119.6, 116.1, 109.5, 64.4, 37.7, 30.9, 29.6, 19.6, 16.8, 13.0, 6.8, 6.6, 5.8, 4.8. **EI-LRMS** *m/z* (%): 305 (M^+, 74), 118 (75), 117 (100). **HRMS** (ESI-TOF) *m/z*: calcd for $C_{21}H_{24}NO^+$ $[M+H]^+$ 306.1852; found 306.1838.

Limitations on the scope of the intramolecular reaction

Having studied the reactivity of different terminal 3-propargylindoles **3** bearing an alkenyl group at C2, we decided to establish the usefulness of this intramolecular cyclopropanation for starting 2-vinyl-3-propargylindoles with non-terminal alkynes, **S9a,b**, and a terminal 2-vinyl-3-propargylindole with an aryl group at the propargylic position, **S9c**. As shown in Scheme S3, no reaction occurred when **S9a**, with a phenyl group as substituent of the alkyne, was subjected to the previously optimized reaction conditions ($BrettPhosAuNTf_2$ as catalyst). However, when using $IPrAuNTf_2$ was used as the catalyst, the indenylindole **S10** was obtained exclusively in a high yield via a tandem 1,2-indole migration / Nazarov cyclization. This reactivity is consistent with our previous report on analogous substrates in the absence of an alkenyl substituent at C2.[15]

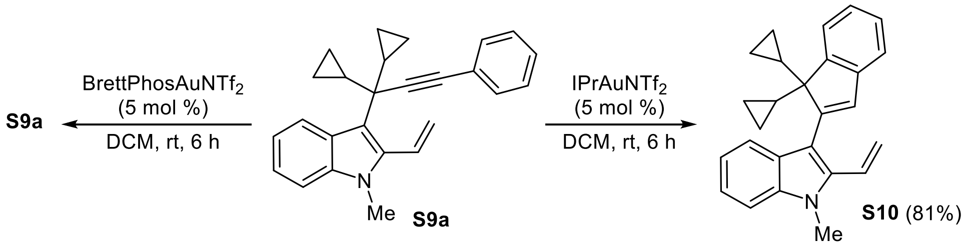

Scheme S3. Reaction of **S9a** under gold-catalysis.

We then investigated the reactivity of a related 2-vinyl-3-propargylindole **S9b** bearing a butyl group as the alkyne substituent. In this case, under the previously optimized conditions, 3-dienylindole **S11** was obtained selectively in a high yield via selective tandem 1,2-indole migration / 1,2-H migration. This compound was obtained as a 3/1 mixture of *E*/*Z* isomers (Scheme S4). Again, this reactivity is consistent with our previous report on analogous substrates in the absence of an alkenyl substituent at C2.[15]

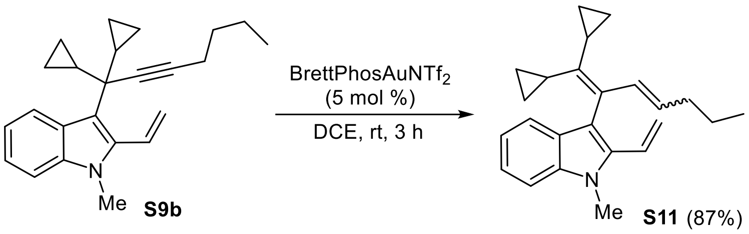

Scheme S4. Reaction of **S9b** under gold-catalysis.

Finally, when **S9c**, possessing a terminal alkyne but an aryl group at the propargylic position, was employed, the reaction does not proceed under BrettPhosAuNTf$_2$-catalysis (Scheme S5). Once more, when using more active IPrAuNTf$_2$ catalyst, the starting material underwent a tandem 1,2-indole migration / iso-Nazarov cyclization, resulting in the formation of indenylindole **S12** in good yield (Scheme S5). This indole derivative was obtained as a mixture of regioisomeric indenylindoles. These results indicate that the tandem 1,2-indole migration / intramolecular cyclopropanation sequence requires the presence of a terminal alkyne and the absence of aromatic groups at the propargylic position to be successful.

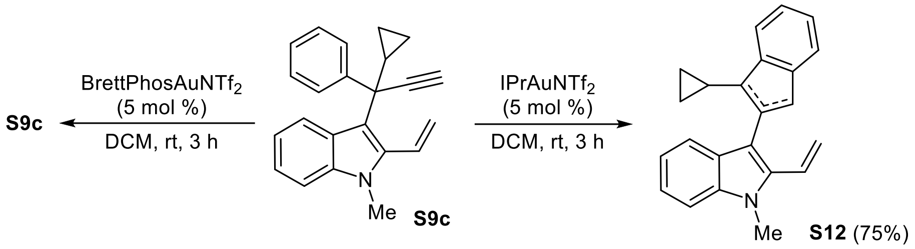

Scheme S5. Reaction of **S9c** under gold-catalysis.

Characterization data:

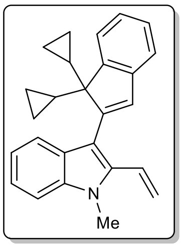

3-(1,1-Dicyclopropyl-1*H*-inden-2-yl)-1-methyl-2-vinyl-1*H*-indole (S10): Following the general procedure F1, but using IPrAuNTf$_2$ as the catalyst, with **S9a** (105 mg, 0.3 mmol), the crude product was purified by flash column chromatography (hexane/EtOAc, 10/1) affording pure **S10** as a colorless solid (86 mg, 81%). R$_f$ = 0.29 (hexane/EtOAc, 10/1); m.p. = 137–139 °C. ^{1}**H NMR** (300 MHz, CDCl$_3$): δ (ppm) = 7.71 (d, J = 7.9 Hz, 1H), 7.50–7.28 (m, 5H), 7.22 (t, J = 7.2 Hz, 1H), 7.14 (t, J = 7.4 Hz, 1H), 6.98 (dd, J = 18.1, 12.0 Hz, 1H), 6.77 (s, 1H), 5.73 (d, J = 18.1 Hz, 1H), 5.40 (d, J = 12.0 Hz, 1H), 3.96 (s, 3H), 1.03–0.89 (m, 2H), 0.70–0.54 (m, 2H), 0.53–0.38 (m, 2H), 0.38–0.25 (m, 2H), 0.17–0.07 (m, 2H).^{13}**C NMR** (75.4 MHz, CDCl$_3$): δ (ppm) = 152.5, 147.6, 144.5, 138.1, 135.6, 131.1, 128.9, 127.5, 127.1, 123.9, 123.7, 122.3, 121.2, 120.7, 119.5, 116.8, 112.6, 109.1, 58.1, 31.7, 14.7, 2.9, –0.1. **EI-LRMS** *m/z* (%): 351 (M$^+$, 53), 294 (25), 194 (100), 182 (24). **HRMS** (ESI-TOF) *m/z*: calcd for $C_{26}H_{26}N^+$ [M+H]$^+$ 352.2060; found 352.2054.

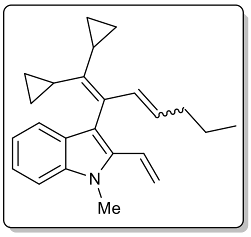

3-(1,1-Dicyclopropylhepta-1,3-dien-2-yl)-1-methyl-2-vinyl-1*H*-indole (S11): Following the general procedure F1 with **S9b** (100 mg, 0.3 mmol), the crude product was purified by flash column chromatography (hexane/EtOAc, 10/1) affording pure **S11** as a yellow oil (87 mg, 87%). R$_f$ = 0.34 (hexane/EtOAc, 10/1). Obtained and isolated as a ca. 3/1 mixture of *E/Z* diastereoisomers. Data for the major diastereoisomer: ^{1}**H NMR** (300 MHz, CDCl$_3$): δ (ppm) = 7.47 (d, J = 7.9 Hz, 1H), 7.36–

7.30 (m, 1H), 7.29–7.21 (m, 1H), 7.14–7.08 (m, 1H), 6.79 (dd, *J* = 18.0, 11.9 Hz, 1H), 6.36 (d, *J* = 11.5 Hz, 1H), 5.66 (d, *J* = 17.9 Hz, 1H), 5.48–5.35 (m, 2H), 3.85 (s, 3H), 1.87–1.63 (m, 2H), 1.54–1.43 (m, 1H), 1.39–1.29 (m, 2H), 1.27–1.15 (m, 2H), 0.94–0.83 (m, 1H), 0.82–0.67 (m, 5H), 0.56–0.44 (m, 2H), 0.42–0.24 (m, 2H). **^{13}C NMR** (75.4 MHz, $CDCl_3$): *δ* (ppm) = 141.3, 137.7, 133.8, 131.2, 129.4, 128.3, 127.9, 126.7, 122.1, 120.6, 119.2, 117.3, 116.6, 108.9, 31.0, 30.8, 22.8, 15.0, 14.0, 13.1, 6.4, 6.2, 5.9, 5.8. **EI-LRMS** *m/z* (%): 331 (M^+, 100), 302 (44), 222 (45), 194 (51). **HRMS** (ESI-TOF) *m/z*: calcd for $C_{24}H_{30}N^+$ $[M+H]^+$ 332.2373; found 332.2368.

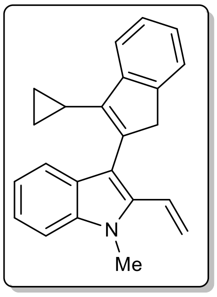

3-(3-Cyclopropyl-1*H*-inden-2-yl)-1-methyl-2-vinyl-1*H*-indole (S12): Following the general procedure F1 with **S9c** (93 mg, 0.3 mmol), the crude product was purified by flash column chromatography (hexane/EtOAc, 10/1) affording pure **S9c** as a yellow solid (90 mg, 75%). R_f = 0.28 (hexane/EtOAc, 10/1); m.p. = 140–142 °C. Obtained and isolated as a ca. 5/1 mixture of **S12** and its regioisomer 3-(1-cyclopropyl-1*H*-inden-2-yl)-1-methyl-2-vinyl-1*H*-indole. Data for the major regioisomer: **^{1}H NMR** (300 MHz, $CDCl_3$): *δ* (ppm) = 7.61 (t, *J* = 8.4 Hz, 2H), 7.53 (d, *J* = 7.3 Hz, 1H), 7.47–7.38 (m, 2H), 7.37–7.25 (m, 2H), 7.23–7.14 (m, 1H), 6.95–6.82 (m, 1H), 5.60 (d, *J* = 16.6 Hz, 1H), 5.48 (d, *J* = 11.8 Hz, 1H), 3.91 (s, 3H), 3.77 (s, 2H), 1.94–1.80 (m, 1H), 0.78–0.70 (m, 4H). **^{13}C NMR** (75.4 MHz, $CDCl_3$): *δ* (ppm) = 146.7, 143.7, 141.1, 137.7, 136.0, 134.7, 127.9, 126.6, 126.3, 124.3, 123.6, 122.4, 120.4, 119.8, 119.7, 118.4, 112.2, 109.3, 41.6, 31.0, 9.9, 5.2. **EI-LRMS** *m/z* (%): 311 (M^+, 100), 282 (41), 270 (39), 254 (41). **HRMS** (ESI-TOF) *m/z*: calcd for $C_{23}H_{22}N^+$ $[M+H]^+$ 312.1747; found 312.1730.

Intramolecular cyclopropanation reactions of 2-allyl-3-propargylindole 5

Optimization studies

Table S4: Optimization of the reaction conditions for the cyclopropanation of **5**.[a]

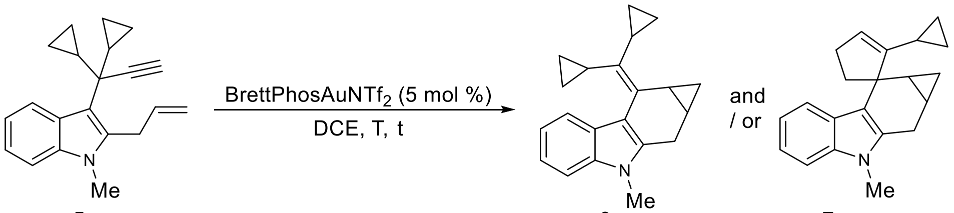

5 **6** **7**

Entry	T (°C)	t (h)	Products[b]	**6/7**[b]
1	rt	3	**6**+**7**	3/1
2	rt	24	**6**+**7**	1.4/1
3	0	3	**6**+**7**	3.3/1
4	0	1.5	**6**	1/0
5	80	6	**7**	0/1

[a]Reaction conditions: **5** (0.2 mmol), catalyst (5 mol %) in dichloroethane (8 mL). [b]Determined by ^{1}H NMR analysis of the crude reaction mixture.

Once the intramolecular cyclopropanations of 3-propargyl indoles bearing an alkenyl moiety at the C2 position of the indole had been explored, we turned our attention to investigating this reactivity using 2-allyl-

3-propargyl indole **5**. Initially, the reaction carried out in the presence of BrettPhosAuNTf$_2$, at rt for 3 h, led to an approximate 3/1 mixture of two products: the expected indole derivative **6**, resulting from a tandem 1,2-indole migration / intramolecular cyclopropanation, and compound **7**, likely arising from a ring expansion of one of the cyclopropane rings onto the exocyclic double bond of compound **6** (entry 1). As the reaction time increased, a higher proportion of **7** was observed, supporting its formation via subsequent transformation of indole **6** (entry 2). In search of conditions that would allow for selective formation of one product over the other, we lowered the reaction temperature to suppress the formation of **7**, which led to the exclusive formation of indole derivative **6** at 0 °C after 1.5 h (entry 4). Conversely, indole **7** was selectively obtained by heating the reaction mixture at 80 °C for 6 h (entry 5).

Au(I)-catalyzed intramolecular cyclopropanation of 2-allyl-3-propargyl indole 5

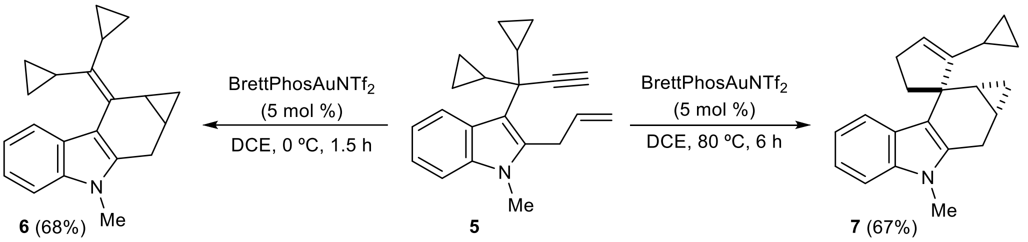

6 (68%) **5** **7** (67%)

Synthesis of hexahydrocyclopropa[b]carbazole (6): BrettPhosAuNTf$_2$ (21.6 mg, 0.025 mmol) was dissolved in DCE (1 mL) and the resulting solution was stirred at rt for 5 min. A solution of indole **6** (144 mg, 0.5 mmol) in DCE (19 mL) was then added. The reaction mixture was stirred at 0 °C for 1.5 h (until complete consumption of the starting material, as determined by GC–MS or TLC). The mixture was filtered through a short pad of silica gel and celite using a 10/1 mixture of hexane/EtOAc. The filtrate was concentrated under reduced pressure, and the crude product was purified by flash column chromatography using a 10/1 mixture of hexane/EtOAc as eluent, to afford hexahydrocyclopropa[*b*]carbazole **6**.

Synthesis of spiro[cyclopentane-1,8'-cyclopropa[b]carbazole]-2-ene (7): The same procedure was followed, except that the reaction mixture was heated to 80 °C and stirred for 6 h. After completion of the reaction, the crude mixture was purified by flash column chromatography using a 10/1 mixture of hexane/EtOAc as eluent, affording tetrahydro-1'*H*-spiro[cyclopentane-1,8'-cyclopropa[*b*]carbazol]-2-ene **7**.

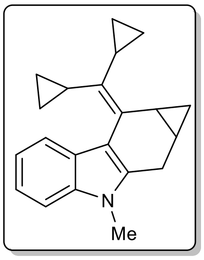

8-(Dicyclopropylmethylene)-3-methyl-1,1*a*,2,3,8,8*a*-hexahydrocyclopropa[*b*]carbazole (6): White solid (98 mg, 68%). R$_f$ = 0.35 (hexane/EtOAc, 10/1); m.p. = 157–159 °C. **^{1}H NMR** (300 MHz, CDCl$_3$): *δ* (ppm) = 7.80 (ad, *J* = 7.8 Hz, 1H), 7.31–7.24 (m, 1H), 7.17 (at, *J* = 7.5 Hz, 1H), 7.09 (at, *J* = 7.5 Hz, 1H), 3.68 (s, 3H), 3.35–3.03 (m, 2H), 2.65–2.52 (m, 1H), 1.95–1.78 (m, 1H), 1.55–1.48 (m, 1H), 1.48–1.36 (m, 1H), 0.92–0.63 (m, 7H), 0.53–0.43 (m, 1H), 0.43–0.30 (m, 1H), 0.18–0.05 (m, 1H). **^{13}C NMR** (75.4 MHz, CDCl$_3$): *δ* (ppm) = 137.3, 134.8, 130.2, 129.6, 126.7, 121.6, 120.4, 118.7, 108.8, 108.5, 29.4, 22.4, 18.2, 18.1, 11.8, 11.4, 8.7, 6.8, 6.3, 6.2, 6.1. **EI-LRMS** *m/z* (%): 289 (M$^+$, 36), 195 (20), 194 (100). **HRMS** (ESI-TOF) *m/z*: calcd for $C_{21}H_{24}N^+$ [M+H]$^+$ 290.1903; found 290.1898.

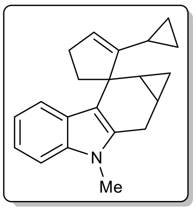

2-Cyclopropyl-3'-methyl-1*a*',2',3',8*a*'-tetrahydro-1'*H*-spiro[cyclopentane-1,8'-cyclopropa[*b*]carbazol]-2-ene (7): White solid (97 mg, 67%). R_f = 0.32 (hexane/EtOAc, 10/1); m.p. = 154–156 °C. **^{1}H NMR** (300 MHz, $CDCl_3$ / MeOD): δ (ppm) = 7.63 (ad, J = 7.8 Hz, 1H), 7.25 (ad, J = 8.1 Hz, 1H), 7.13 (at, J = 7.5 Hz, 1H), 7.02 (at, J = 7.5 Hz, 1H), 5.34 (s, 1H), 3.62 (s, 3H), 3.22–2.92 (m, 2H), 2.44–2.32 (m, 2H), 2.25–2.04 (m, 2H), 1.44–1.27 (m, 1H), 1.26–1.12 (m, 1H), 1.09–0.95 (m, 1H), 0.77–0.68 (m, 1H), 0.68–0.48 (m, 4H), 0.48–0.31 (m, 1H). **^{13}C NMR** (75.4 MHz, $CDCl_3$ / MeOD): δ (ppm) = 153.6, 137.3, 132.5, 126.2, 120.2, 119.8, 119.0, 118.5, 111.4, 108.4, 50.5, 42.3, 29.4, 29.0, 21.7, 21.0, 11.1, 9.9, 8.6, 6.32, 6.26. **EI-LRMS** *m/z* (%): 289 (M^+, 100), 209 (72), 194 (49). **HRMS** (ESI-TOF) *m/z*: calcd for $C_{21}H_{24}N^+$ $[M+H]^+$ 290.1903; found 290.1902.

Intramolecular cyclopropanation reactions of 2-(bis)homoallyl-3-propargylindoles 8: Synthesis and characterization data of 7- and 8-membered polycyclic indole derivatives 9

General procedure H for the synthesis cyclohepta and cycloocta[1,2-b]indoles 9

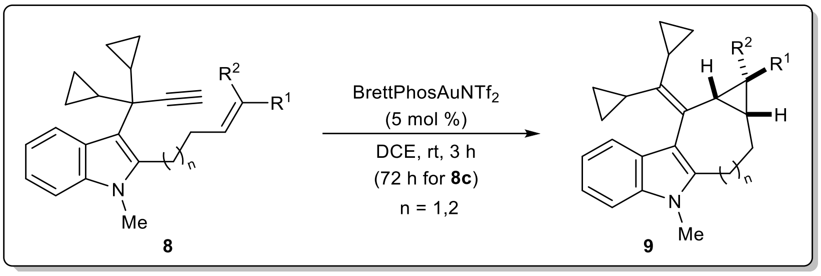

$BrettPhosAuNTf_2$ (21.6 mg, 0.025 mmol, 0.05 equiv) was dissolved in DCE (1 mL), and the resulting solution was stirred at rt for 5 min. A solution of the corresponding 3-propargyl indole **8** (0.5 mmol, 1 equiv) in DCE (19 mL, 0.025 M) was then added. The reaction mixture was stirred at rt for 3 h (until complete consumption of the starting material as determined by GC–MS or TLC). The mixture was filtered through a short pad of silica gel and celite using a 10/1 mixture of hexane/EtOAc, and the solvents were removed under reduced pressure. The crude residue was purified by flash column chromatography using mixtures of hexane/EtOAc as eluents to afford the corresponding indole derivatives **9**.

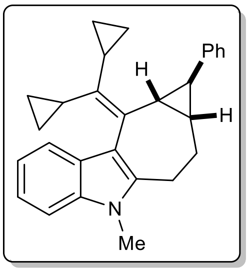

(1*S,1a*S**,9a*S**)-9-(Dicyclopropylmethylene)-4-methyl-1-phenyl-1*a*,2,3,4,9,9*a*-hexahydro-1*H*-cyclopropa[4,5]cyclohepta[1,2-*b*]indole (9a)**: Following the general procedure H with **8a** (190 mg, 0.5 mmol), the crude product was purified by column chromatography (hexane/EtOAc, 10/1) affording pure **9a** as a light orange oil (104 mg, 55%). R_f = 0.29 (hexane/EtOAc, 10/1). **^{1}H NMR** (300 MHz, $CDCl_3$): δ (ppm) = 7.49 (ad, J = 7.7 Hz, 1H), 7.34–7.27 (m, 3H), 7.24–7.16 (m, 2H), 7.15–7.07 (m, 3H), 3.72 (s, 3H), 3.20–2.92 (m, 2H), 2.57–2.48 (m, 1H), 2.39–2.29 (m, 1H), 2.14 (t, J = 4.6 Hz, 1H), 1.96–1.72 (m, 1H), 1.65–1.53 (m, 1H), 1.52–1.36 (m, 2H), 0.98–0.87 (m, 1H), 0.81–0.69 (m, 2H), 0.62–0.50 (m, 3H), 0.49–0.37 (m, 1H), 0.18–0.04 (m, 1H). **^{13}C NMR** (75.4 MHz, $CDCl_3$): δ (ppm) = 143.9, 139.7, 136.4, 136.3, 128.3, 127.9, 127.4, 125.5, 125.2, 120.5, 120.3, 118.8, 115.1, 108.3, 37.0, 33.6, 29.5, 29.3, 27.9, 26.7, 15.5, 14.0, 7.3, 7.1, 5.6, 4.7. **EI-LRMS** *m/z* (%): 379 (M^+, 10), 262 (66), 197 (100). **HRMS** (ESI-TOF) *m/z*: calcd for $C_{28}H_{30}N^+$ $[M+H]^+$ 380.2373; found 380.2364.

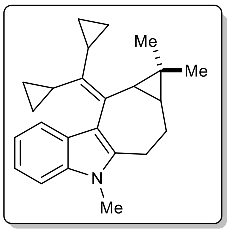

9-(Dicyclopropylmethylene)-1,1,4-trimethyl-1*a*,2,3,4,9,9*a*-hexahydro-1*H*-cyclopropa[4,5]cyclohepta[1,2-*b*]indole (9b): Following the general procedure H with **8b** (165 mg, 0.5 mmol), the crude product was purified by column chromatography (hexane/EtOAc, 10/1) affording pure **9b** as a white solid (104 mg, 63%). R_f = 0.40 (hexane/EtOAc, 10/1); m.p. = 111–113 °C. **^{1}H NMR** (300 MHz, $CDCl_3$): δ (ppm) = 7.42 (ad, *J* = 7.8 Hz, 1H), 7.26 (ad, *J* = 7.8 Hz, 1H), 7.15 (at, *J* = 7.5 Hz, 1H), 7.07 (at, *J* = 7.5 Hz, 1H), 3.68 (s, 3H), 3.15–2.84 (m, 2H), 2.23–2.06 (m, 1H), 1.78–1.63 (m, 2H), 1.60–1.48 (m, 1H), 1.47–1.36 (m, 1H), 1.26 (s, 3H), 1.15 (s, 3H), 1.03–0.92 (m, 1H), 0.83–0.71 (m, 3H), 0.69–0.49 (m, 3H), 0.48–0.31 (m, 1H), 0.17–0.00 (m, 1H). **^{13}C NMR** (75.4 MHz, $CDCl_3$): δ (ppm) = 140.0, 136.9, 136.2, 127.8, 125.6, 120.4, 120.3, 118.6, 115.8, 108.2, 36.9, 29.4, 28.5, 27.8, 26.0, 22.2, 21.5, 17.1, 16.1, 14.4, 8.3, 7.7, 5.9, 5.0. **EI-LRMS** *m/z* (%): 331 (M^+, 30), 262 (100), 207 (91). **HRMS** (ESI-TOF) *m/z*: calcd for $C_{48}H_{59}N_2^+$ $[2M+H]^+$ 663.4673; found 663.4666.

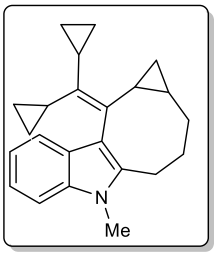

10-(Dicyclopropylmethylene)-5-methyl-1,1*a*,2,3,4,5,10,10*a*-octahydrocyclopropa[4,5]cycloocta[1,2-*b*]indole (9c): Following the general procedure H with **8c** (159 mg, 0.5 mmol), for 72 h, the crude product was purified by column chromatography (hexane/EtOAc, 10/1) affording pure **9c** as a yellow oil (98 mg, 62%). R_f = 0.42 (hexane/EtOAc, 10/1). **^{1}H NMR** (300 MHz, $CDCl_3$): δ (ppm) = 7.37 (ad, *J* = 7.8 Hz, 1H), 7.27 (ad, *J* = 7.8 Hz, 1H), 7.14 (at, *J* = 6.9 Hz, 1H), 7.06 (at, *J* = 6.9 Hz, 1H), 3.69 (s, 3H), 3.32–3.17 (m, 1H), 2.86–2.73 (m, 1H), 2.39–2.25 (m, 1H), 1.91–1.78 (m, 1H), 1.74–1.59 (m, 1H), 1.58–1.47 (m, 1H), 1.36–1.20 (m, 1H), 1.12–0.98 (m, 2H), 0.98–0.86 (m, 1H), 0.81–0.70 (m, 2H), 0.58–0.44 (m, 3H), 0.44–0.29 (m, 3H), 0.07– –0.05 (m, 1H), –0.09– –0.18 (m, 1H). **^{13}C NMR** (75.4 MHz, $CDCl_3$): δ (ppm) = 137.5, 136.8, 136.3, 131.5, 128.3, 120.1, 120.0, 118.6, 113.4, 108.5, 29.3, 26.8, 26.6, 23.0, 22.3, 19.2, 16.2, 12.83, 12.75, 6.7, 6.0, 5.8, 5.1. **EI-LRMS** *m/z* (%): 317 (M^+, 100), 197 (85), 147 (81). **HRMS** (ESI-TOF) *m/z*: calcd for $C_{23}H_{28}N^+$ $[M+H]^+$ 318.2216; found 318.2206.

Synthetic procedure for the preparation of *cis*-2a (1 mmol scale):

BrettPhosAuNTf$_2$ (43 mg, 0.05 mmol) was dissolved in DCE (1 mL) and the resulting solution was stirred at rt for 5 min. A solution of the styrene (156 mg, 1.5 mmol) in DCE (1 mL) and a solution of 3-propargylindole **1a** (249 mg, 1 mmol) in DCE (2 mL) were then added sequentially. The reaction mixture was stirred at rt for 3h (complete consumption of the starting material determined by GC–MS). The crude mixture was filtered through a short pad of silica gel and celite using a 5/1 mixture of hexane/EtOAc. The solvents were removed under reduced pressure, and the residue was purified by flash column chromatography (hexane/EtOAc, 7/1) affording pure **2a** as a yellow oil (237 mg, 67%). R_f = 0.20 (hexane/EtOAc, 5/1). Obtained and isolated as a ca. 15/1 mixture of *cis/trans* diastereoisomers.

X-Ray Crystallographic Data for 6

A single crystal of CCDC 2446430 (**6**) suitable for X-Ray crystallography was obtained by crystallization from a hexane/CH_2Cl_2 solution. The crystal was kept at 283.0 K during data collection on Bruker D8 VENTURE PhotonIII area-detector diffractometer. The structure was solved with the ShelXT[16a] structure solution program using Intrinsic Phasing and refined with the ShelXL[16b] refinement package using Least Squares minimization within the OLEX2 suite (Figure 1).[17]

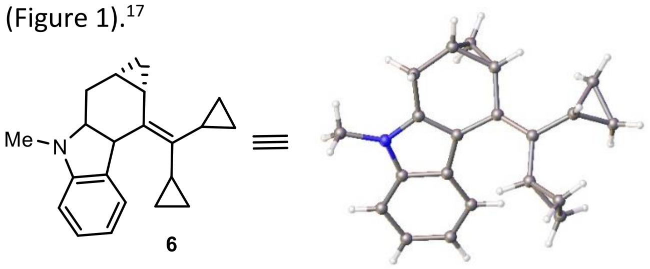

Figure 1. Compound **6** (thermal ellipsoids drawn at the 50% probablility level)

Table S4: Crystal data and structure refinement for 6 (CCDC2446430)

Identification code	**6**
Empirical formula	$C_{21}H_{23}N$
Formula weight	289.40
Temperature/K	300.0
Crystal system	monoclinic
Space group	$P2_1/c$
a/Å	8.1954(13)
b/Å	23.332(4)
c/Å	8.6709(13)
α/°	90
β/°	105.655(6)
γ/°	90
Volume/Å^3	1596.5(4)
Z	4
ρ_{calc}g/cm^3	1.204
μ/mm^{-1}	0.069
F(000)	624.0
Crystal size/mm^3	0.1 × 0.1 × 0.08
Radiation	MoKα (λ = 0.71073)
2Θ range for data collection/°	5.182 to 55.11
Index ranges	-10 ≤ h ≤ 10, -30 ≤ k ≤ 30, -11 ≤ l ≤ 11
Reflections collected	47184
Independent reflections	3686 [R_{int} = 0.0993, R_{sigma} = 0.0390]
Data/restraints/parameters	3686/0/201
Goodness-of-fit on F^2	1.164
Final R indexes [I>=2σ (I)]	R_1 = 0.0566, wR_2 = 0.1416
Final R indexes [all data]	R_1 = 0.1248, wR_2 = 0.2037
Largest diff. peak/hole / e Å^{-3}	0.20/-0.18

[16] (a) Sheldrick, G. M. *Acta Cryst*. **2015**, *A71*, 3-8. (b) Sheldrick, G. M.; *Acta Cryst*. **2015**, *C71*, 3-8.
[17] Dolomanov, O. V.; Bourhis, L. J.; Gildea, R. J.; Howard, J. A. K.; Puschmann, H. *J. Appl. Crystallogr*. **2009**, *42*, 339–341.

X-Ray Crystallographic Data for 7

A single crystal of CCDC 2446429 (**7**) suitable for X-Ray crystallography was obtained by crystallization from a hexane/CH_2Cl_2 solution. The crystal was kept at 283.0 K during data collection on a Bruker APEX-II CCD diffractometer. The structure was solved with the ShelXT[16a] structure solution program using Intrinsic Phasing and refined with the ShelXL[16b] refinement package using Least Squares minimization within the OLEX2 suite.[17]

Figure 2. Compound **7** (thermal ellipsoids drawn at the 50% probablility level)

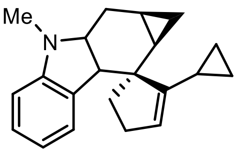

7

≡

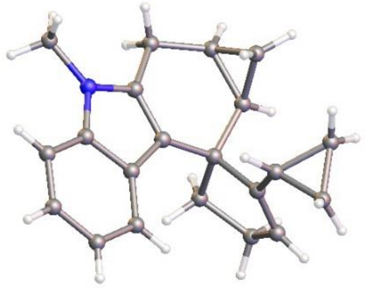

**Table S5: Crystal data and structure refinement for 7 (CCDC2446429)**	
Identification code	**7**
Empirical formula	$C_{21}H_{23}N$
Formula weight	289.40
Temperature/K	283.0
Crystal system	monoclinic
Space group	$P2_1/n$
a/Å	9.4042(6)
b/Å	12.1491(7)
c/Å	14.2194(8)
α/°	90
β/°	96.444(3)
γ/°	90
Volume/Å^3	1614.34(17)
Z	4
ρ_{calc}g/cm^3	1.191
μ/mm^{-1}	0.514
F(000)	624.0
Crystal size/mm^3	0.15 × 0.06 × 0.05
Radiation	CuKα (λ = 1.54178)
2Θ range for data collection/°	12.528 to 147.044
Index ranges	-11 ≤ h ≤ 11, -12 ≤ k ≤ 14, -17 ≤ l ≤ 17
Reflections collected	22518
Independent reflections	3200 [R_{int} = 0.0530, R_{sigma} = 0.0296]
Data/restraints/parameters	3200/0/200
Goodness-of-fit on F^2	1.037
Final R indexes [I>=2σ (I)]	R_1 = 0.0477, wR_2 = 0.1040
Final R indexes [all data]	R_1 = 0.0675, wR_2 = 0.1247
Largest diff. peak/hole / e Å^{-3}	0.23/-0.16

^{1}H and ^{13}C NMR Spectra

^{1}H NMR ($CDCl_3$, 300 MHz)

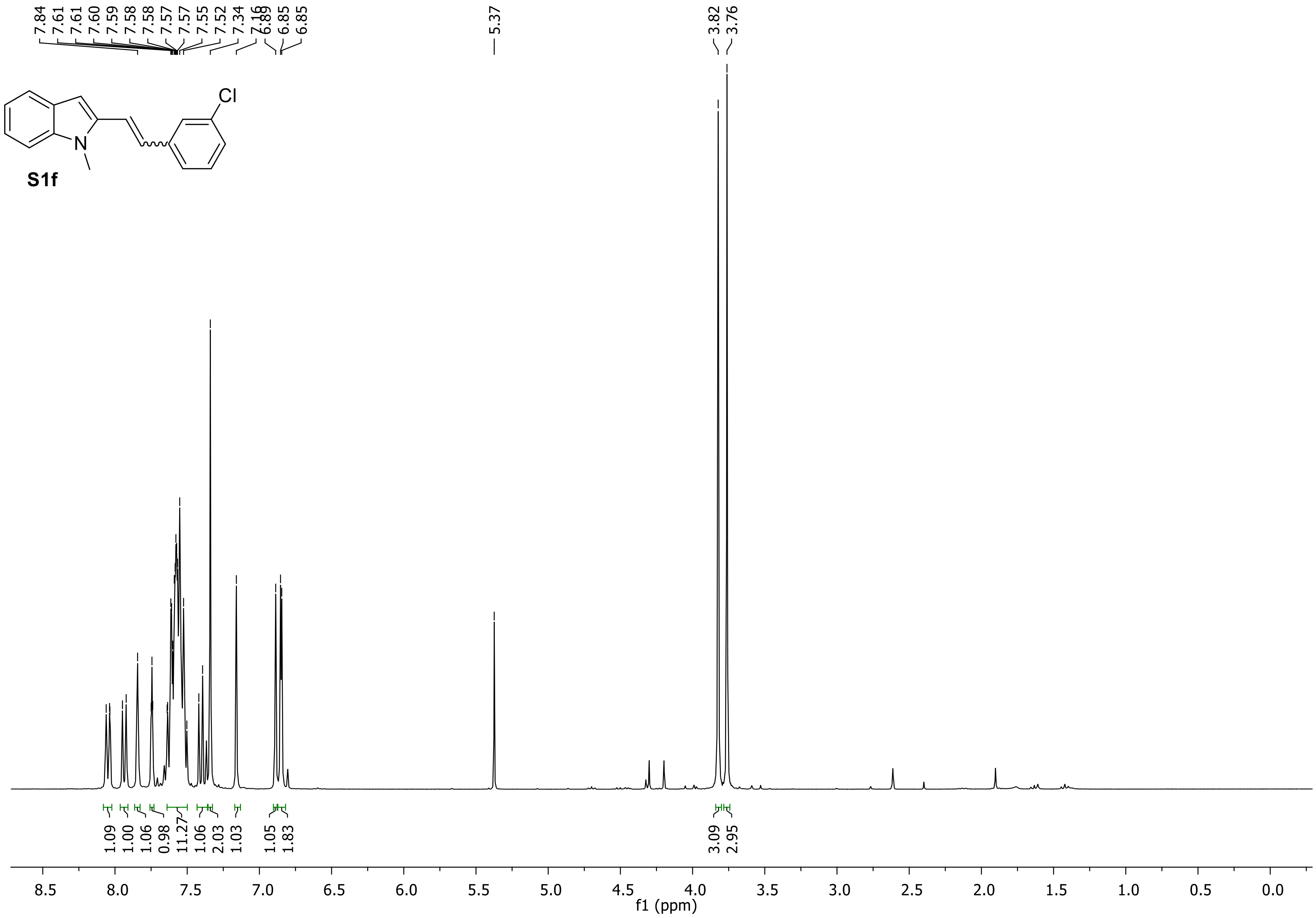

^{13}C NMR ($CDCl_3$, 75.4 MHz)

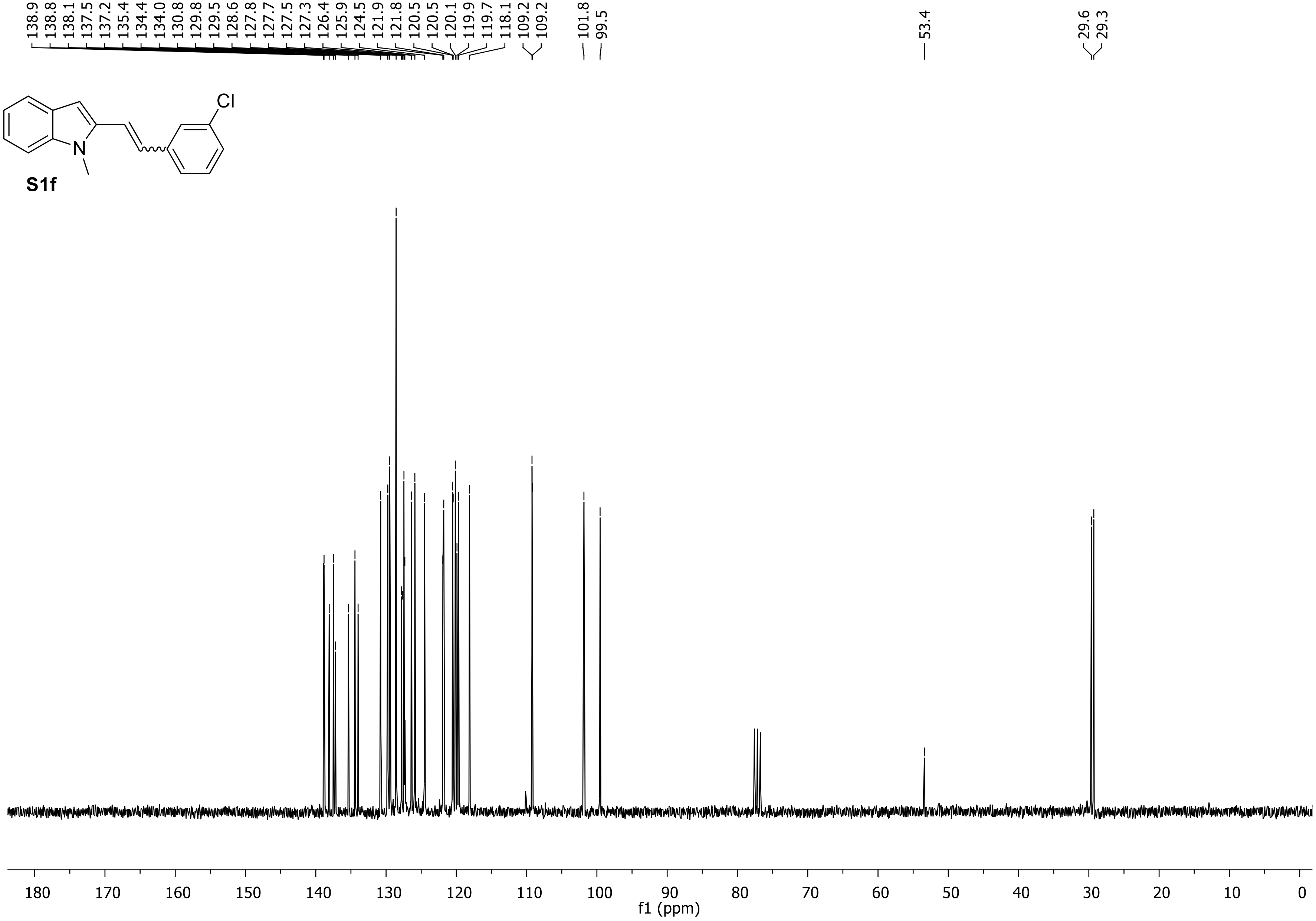

S1f

^{1}H NMR ($CDCl_3$, 300 MHz)

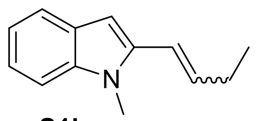

S1i

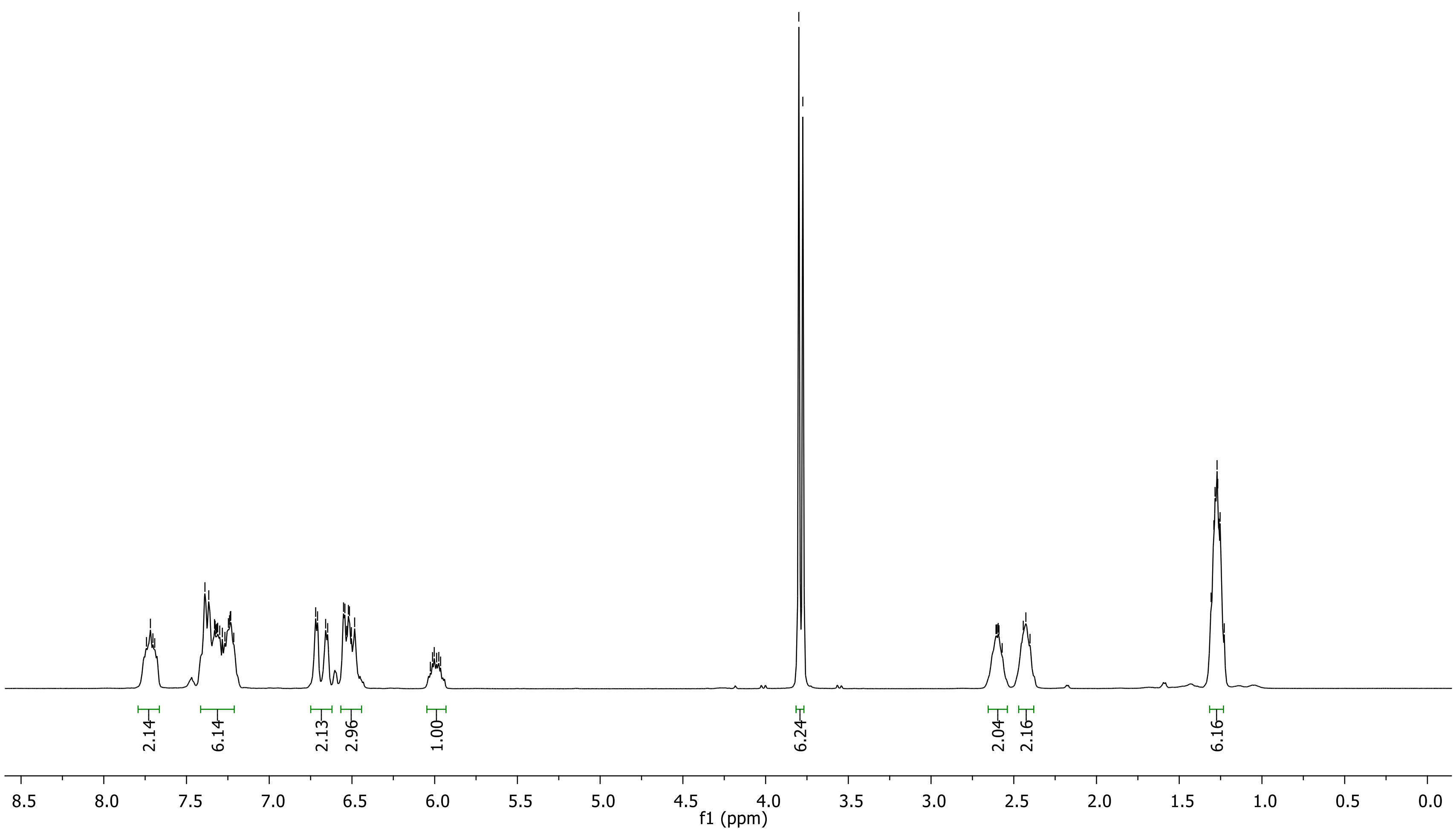

^{13}C NMR ($CDCl_3$, 75.4 MHz)

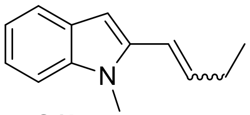

S1i

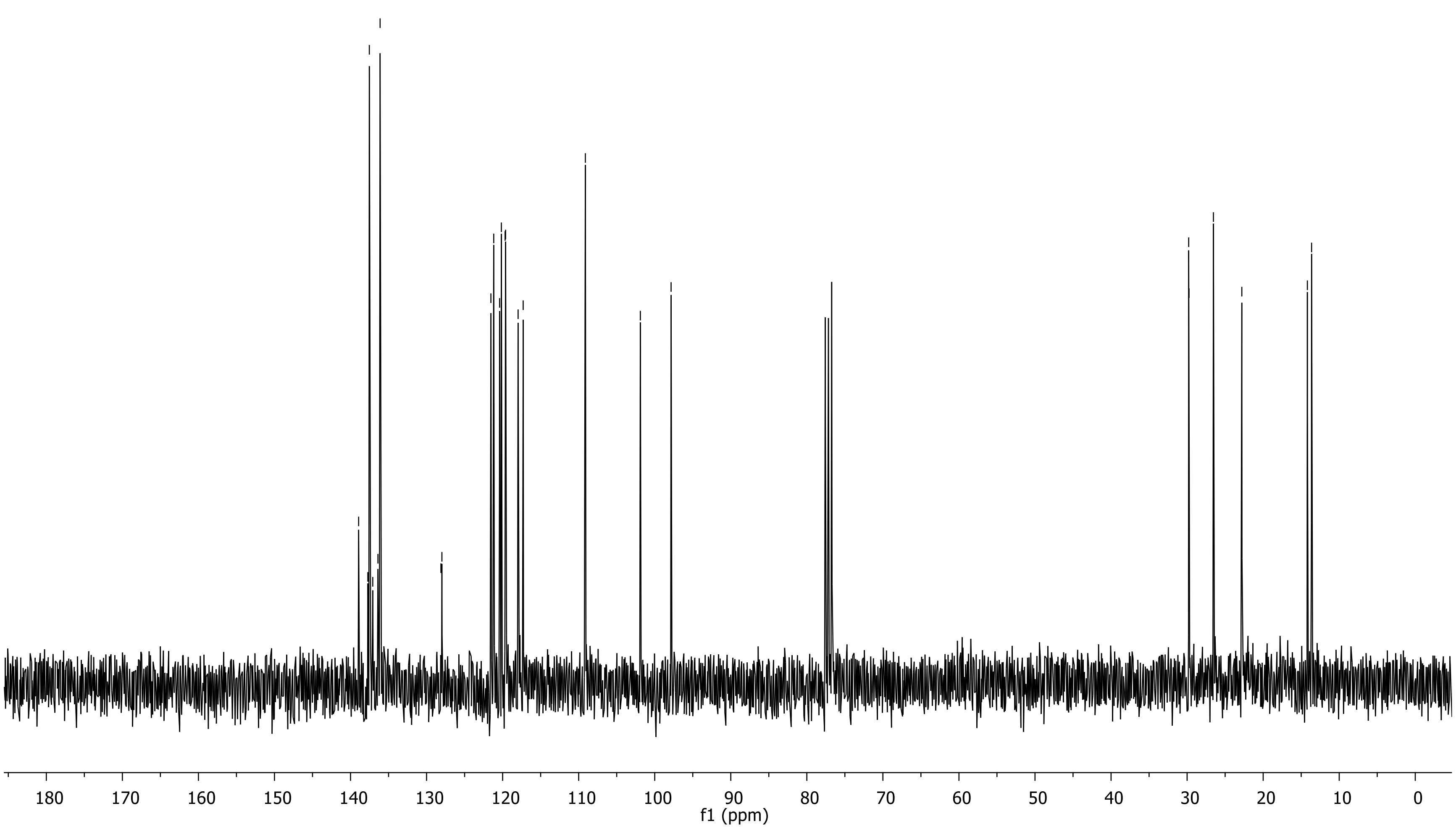

^{1}H NMR (CDCl$_3$, 300 MHz)

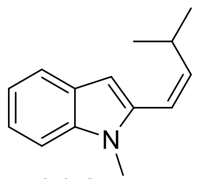

(*Z*)-S1j

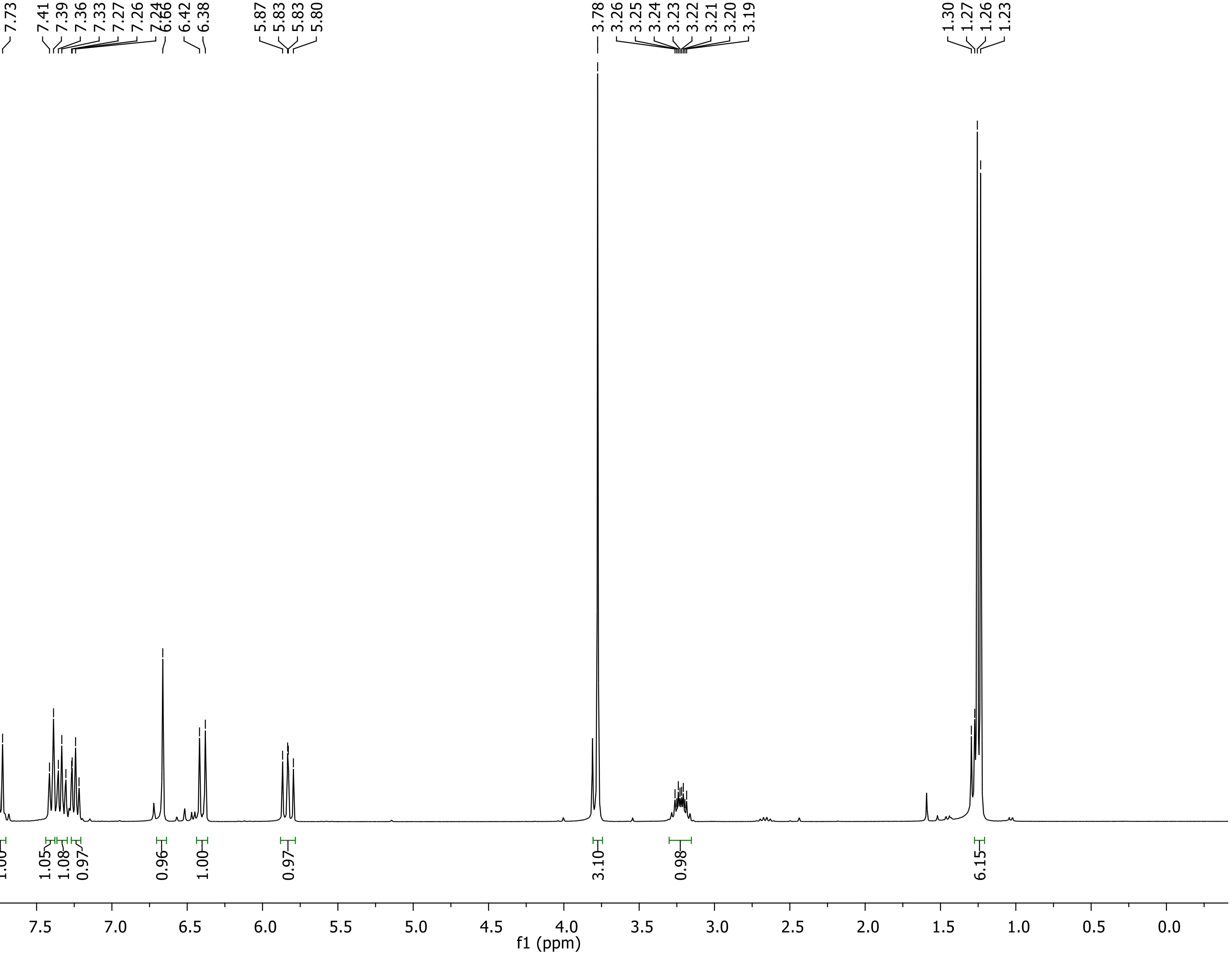

^{13}C NMR ($CDCl_3$, 75.4 MHz)

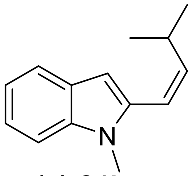

(*Z*)-S1j

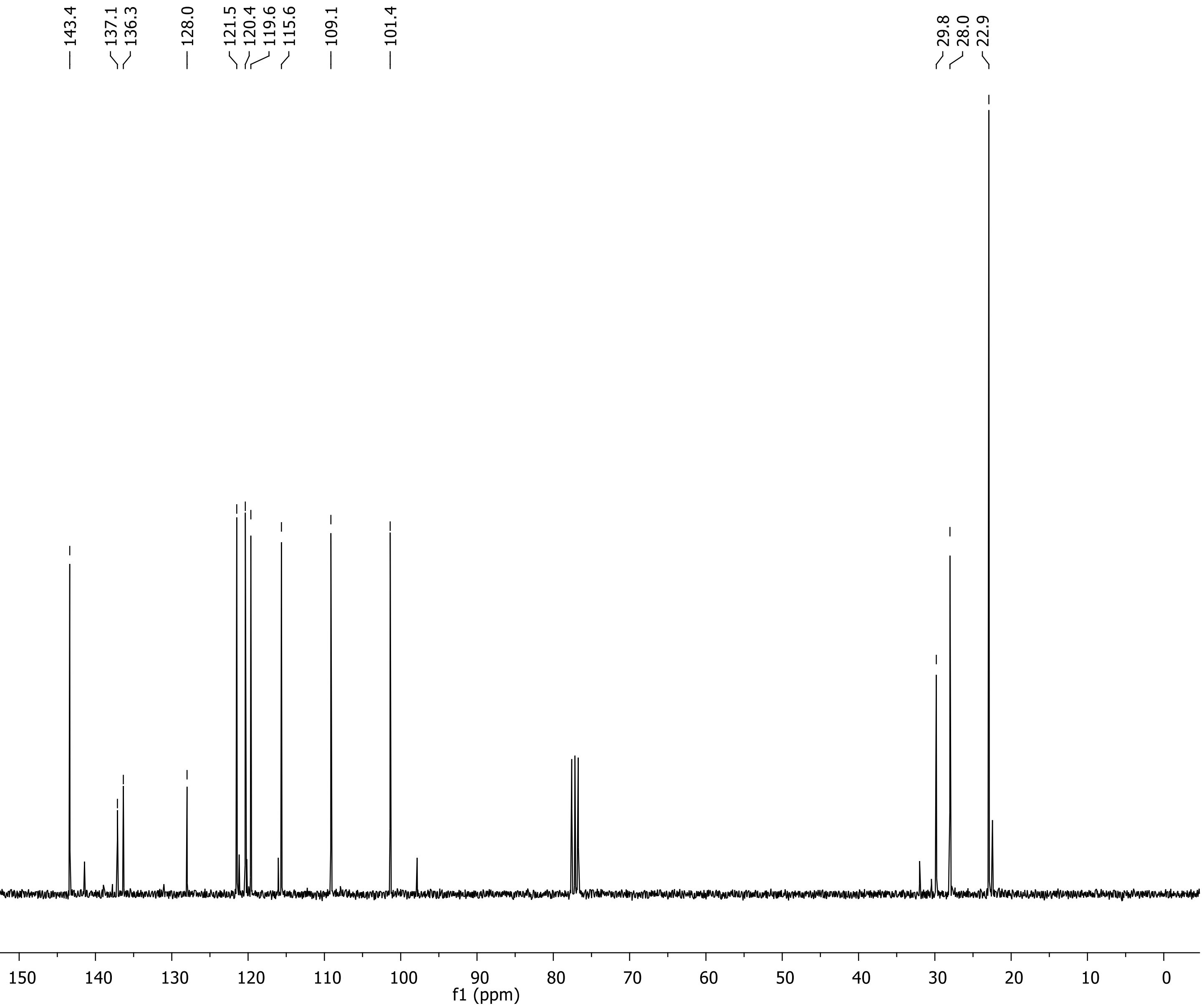

^{1}H NMR ($CDCl_3$, 500 MHz)

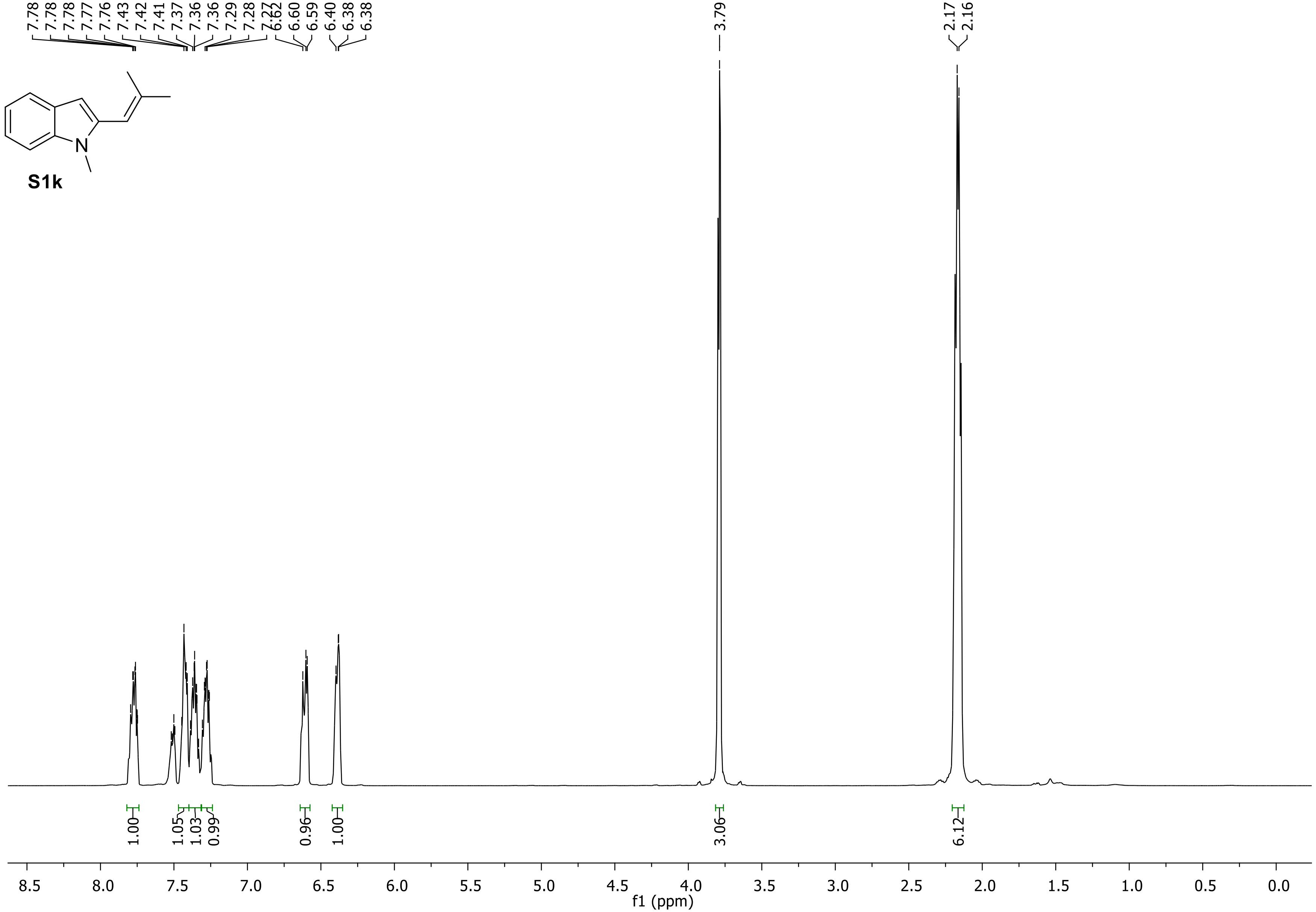

S1k

^{13}C NMR ($CDCl_3$, 125 MHz)

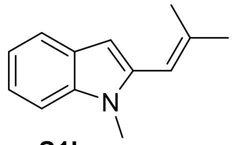

S1k

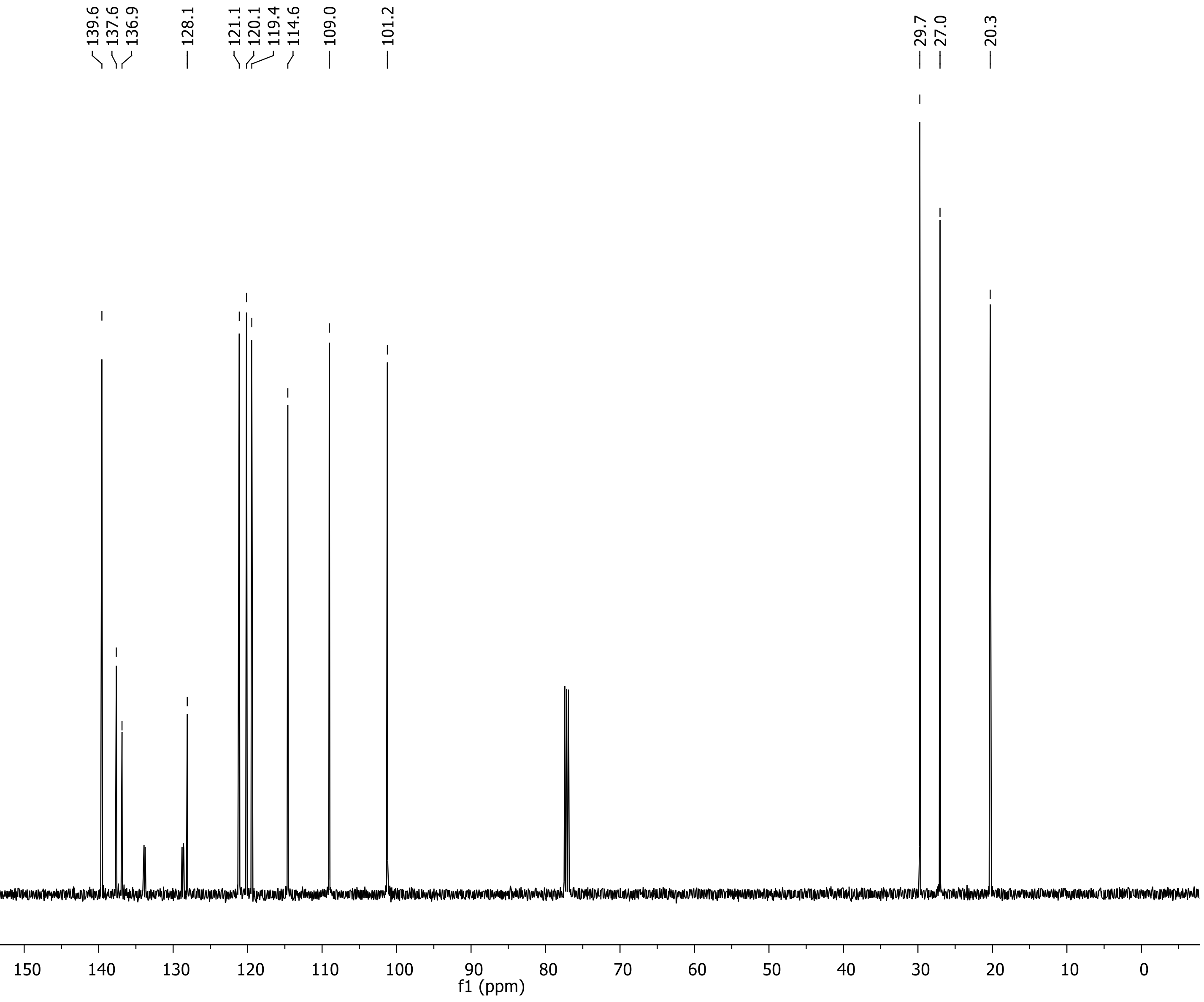

^{1}H NMR ($CDCl_3$, 300 MHz)

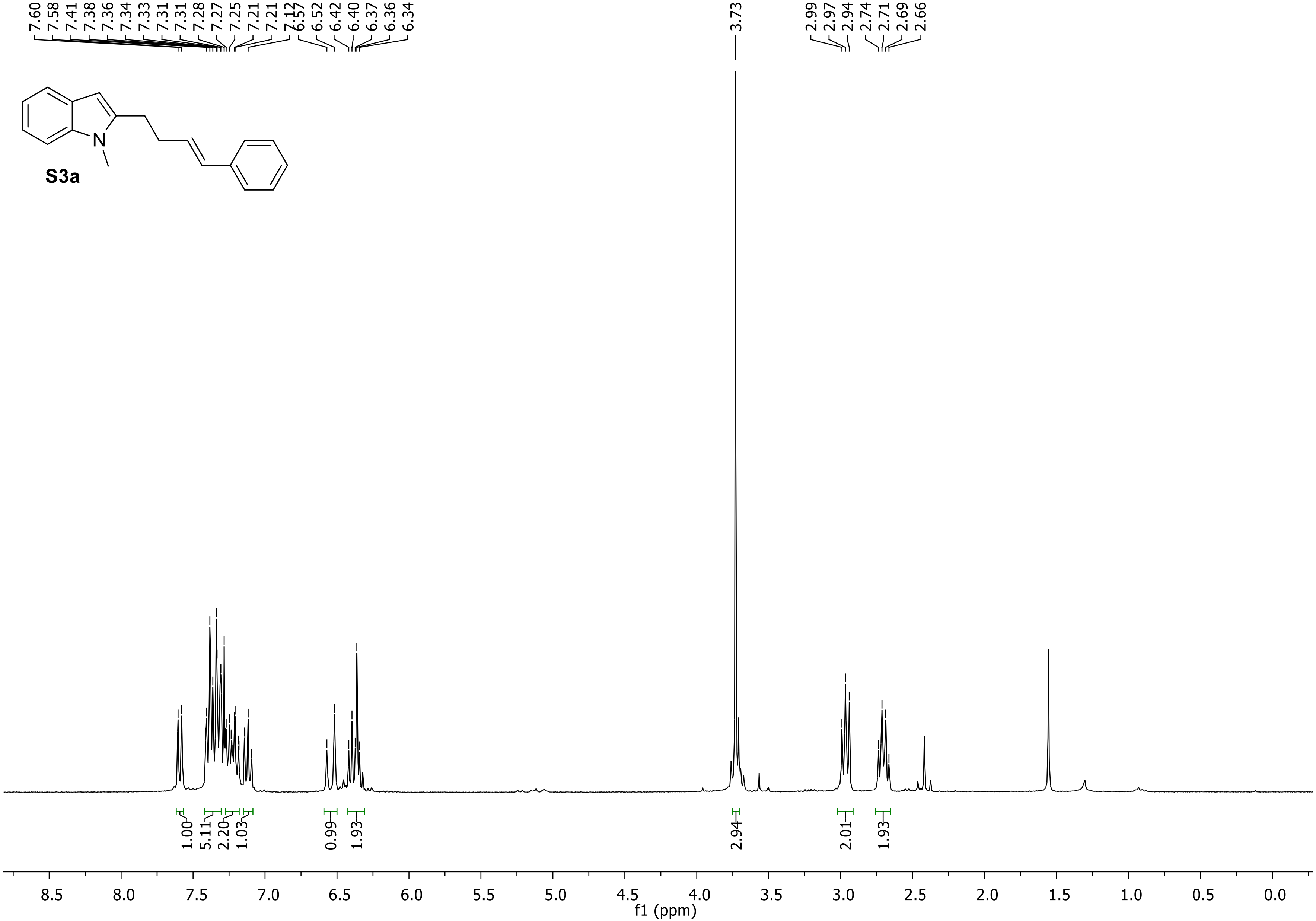

^{13}C NMR ($CDCl_3$, 75.4 MHz)

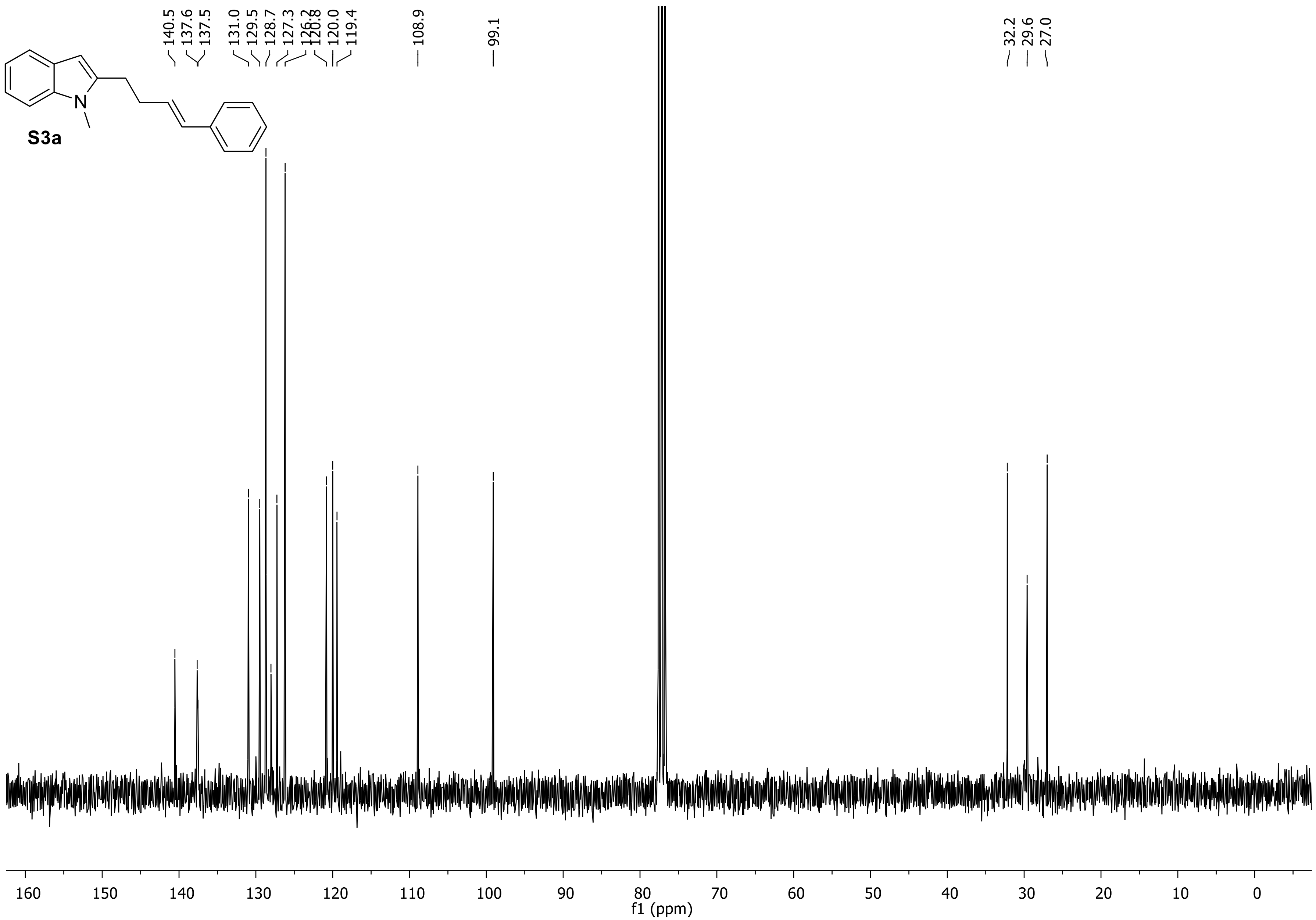

^{1}H NMR (CDCl$_3$, 500 MHz)

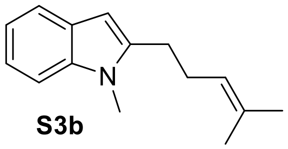

S3b

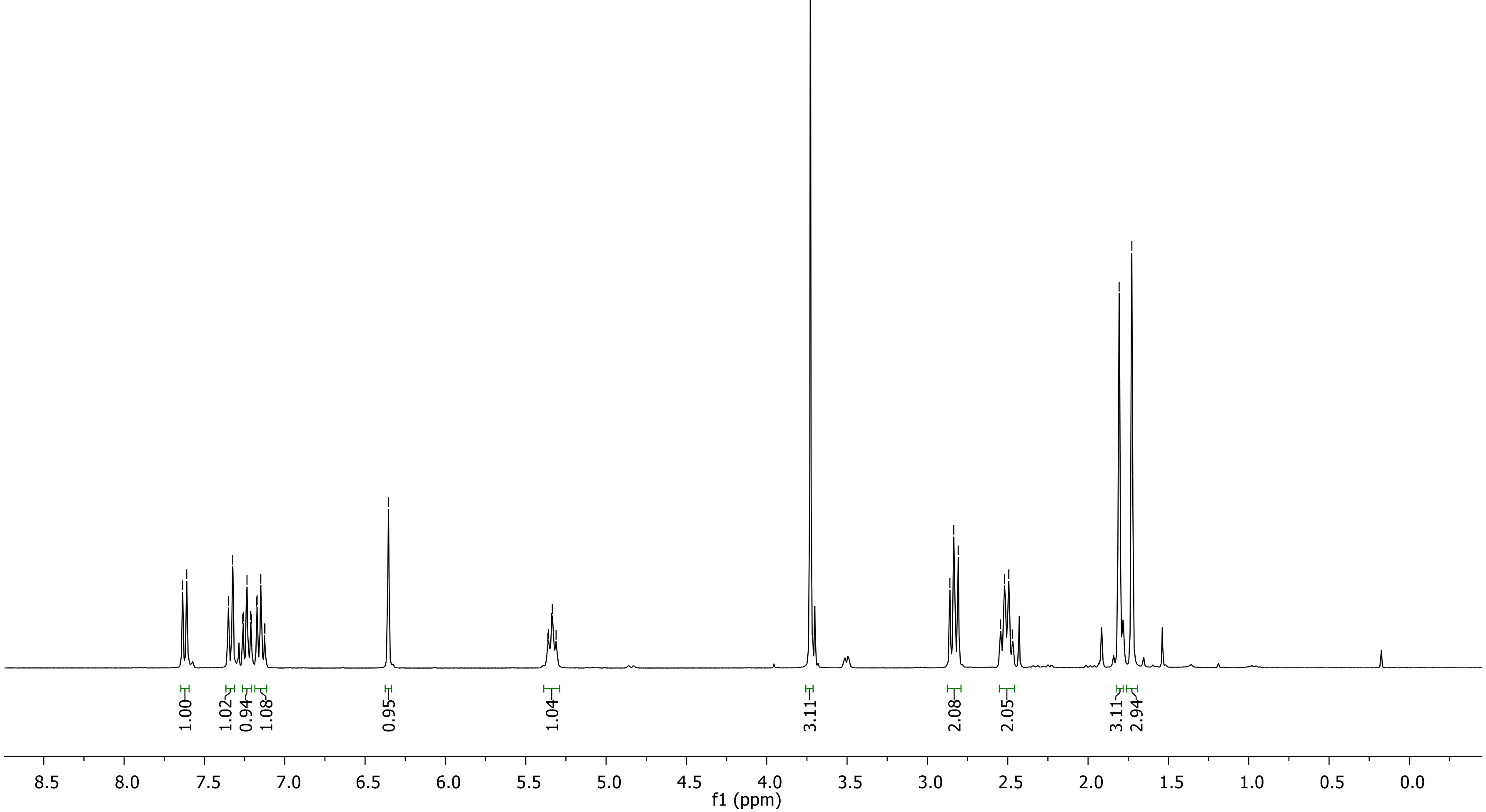

^{13}C NMR ($CDCl_3$, 125 MHz)

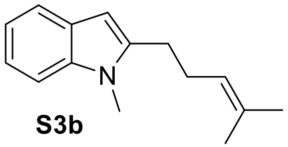

S3b

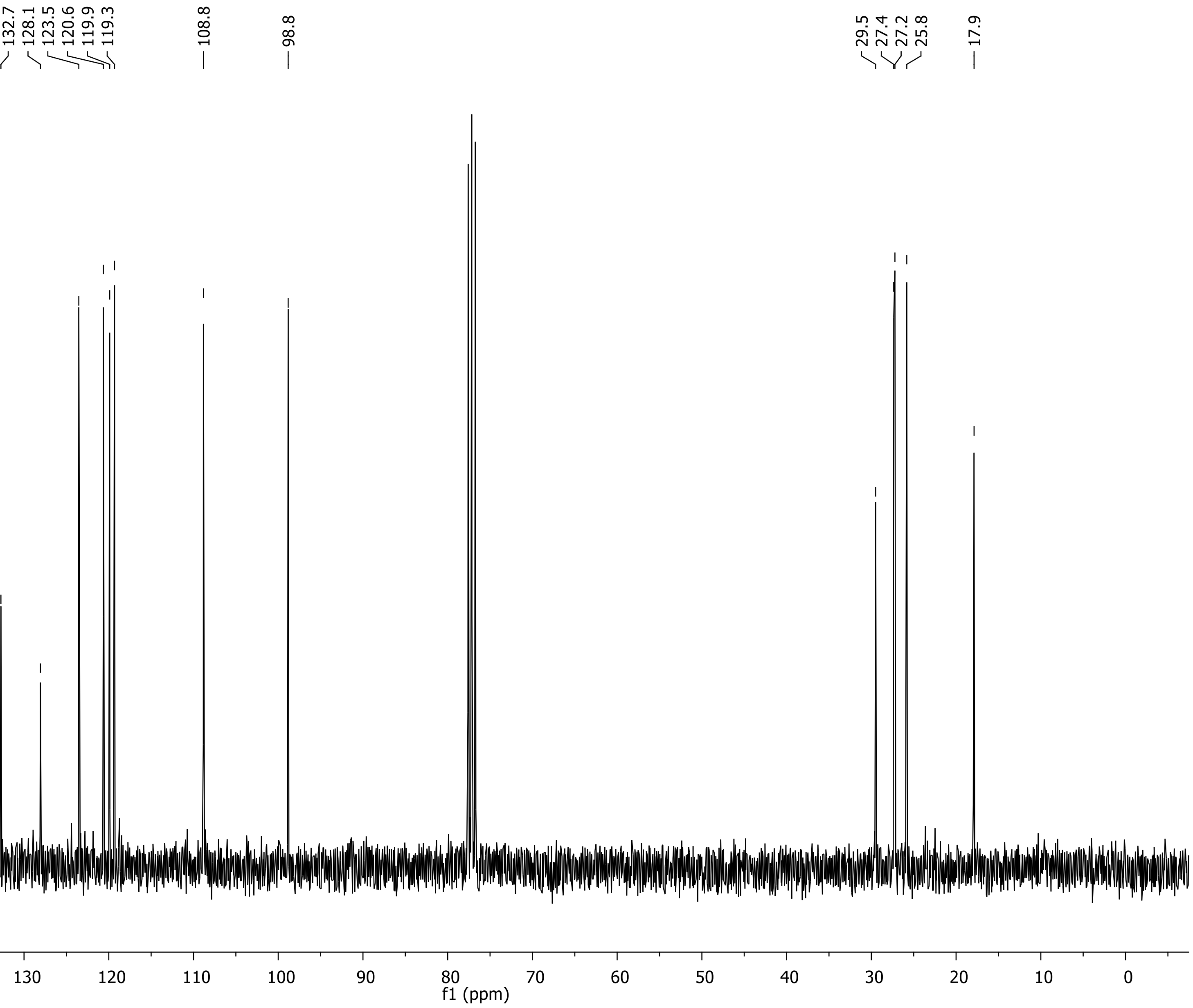

^{1}H NMR ($CDCl_3$, 300 MHz)

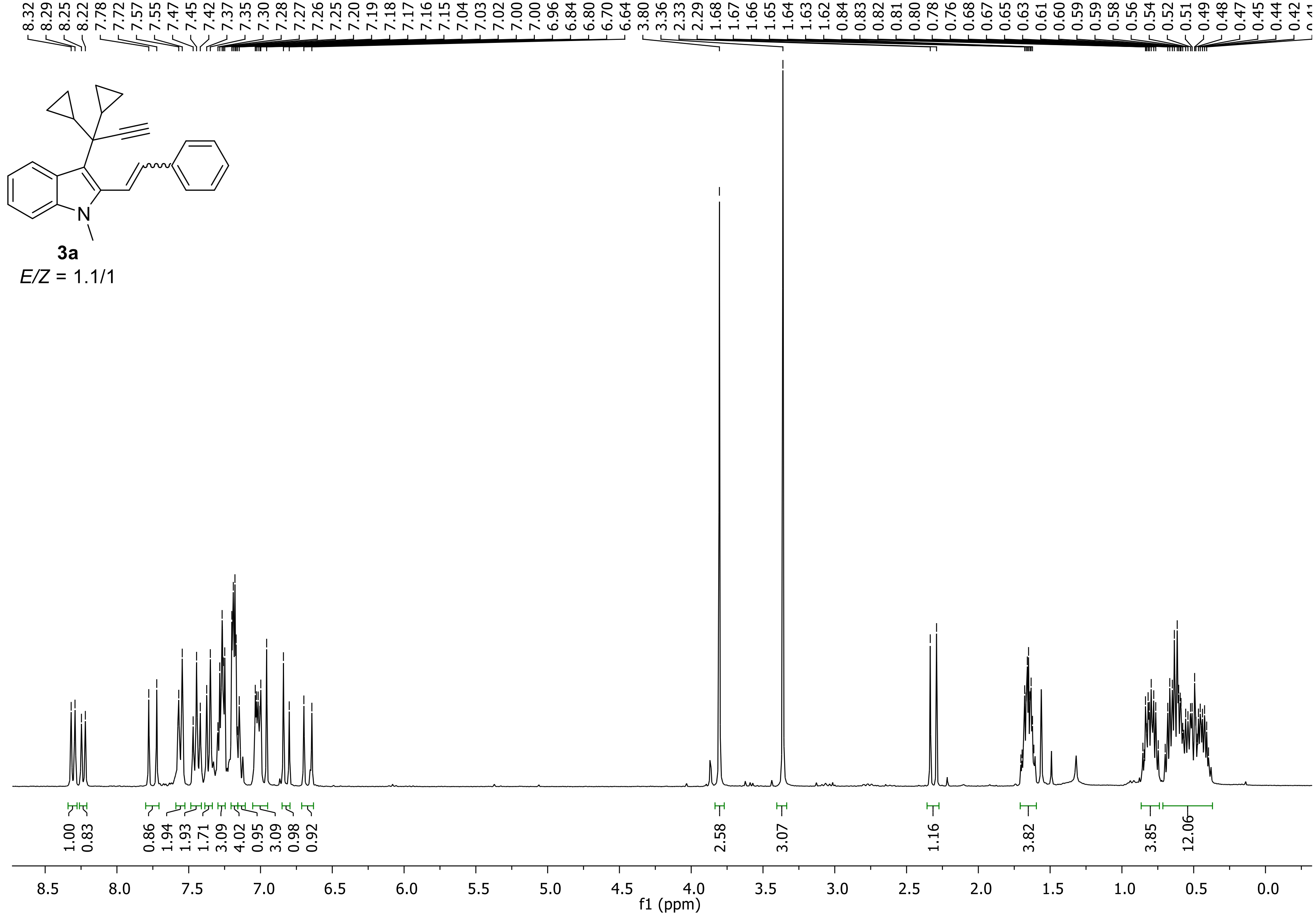

3a

E/Z = 1.1/1

^{13}C NMR ($CDCl_3$, 75.4 MHz)

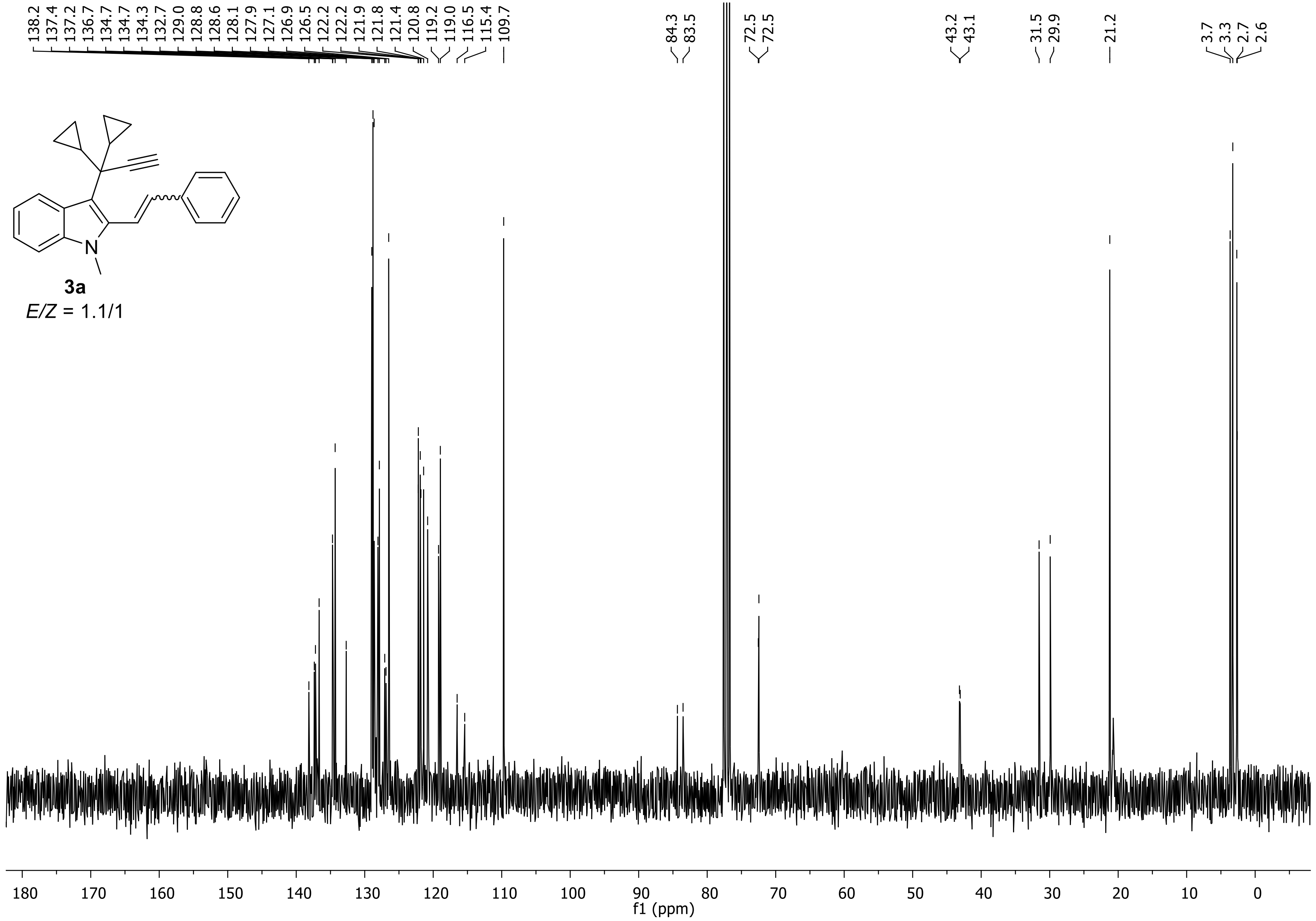

3a

E/*Z* = 1.1/1

^{1}H NMR ($CDCl_3$, 300 MHz)

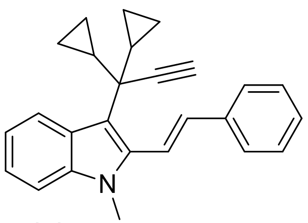

(*E*)-3a

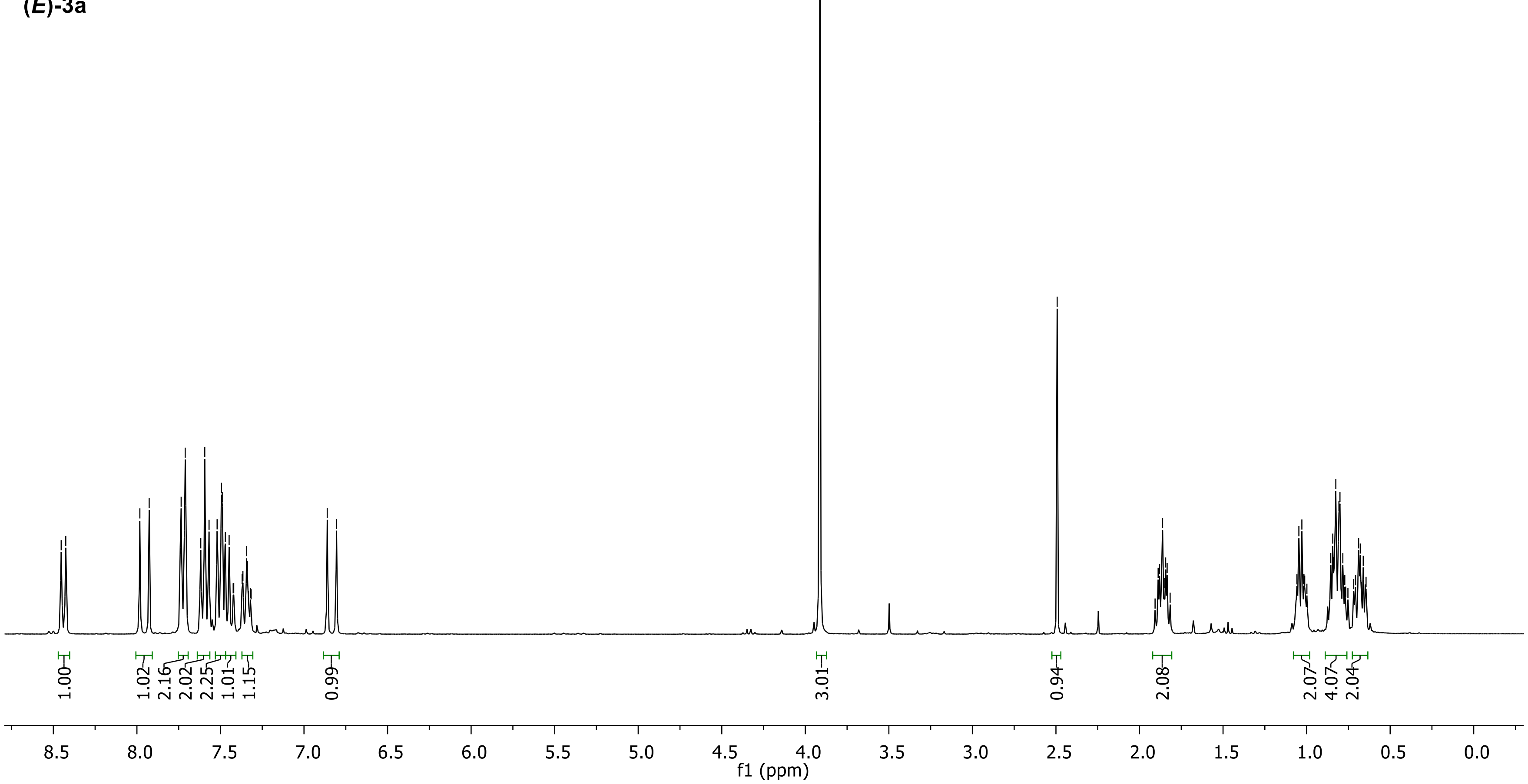

^{13}C NMR ($CDCl_3$, 75.4 MHz)

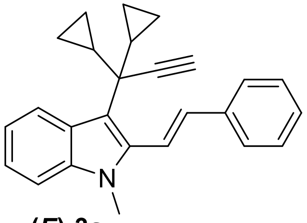

(*E*)-3a

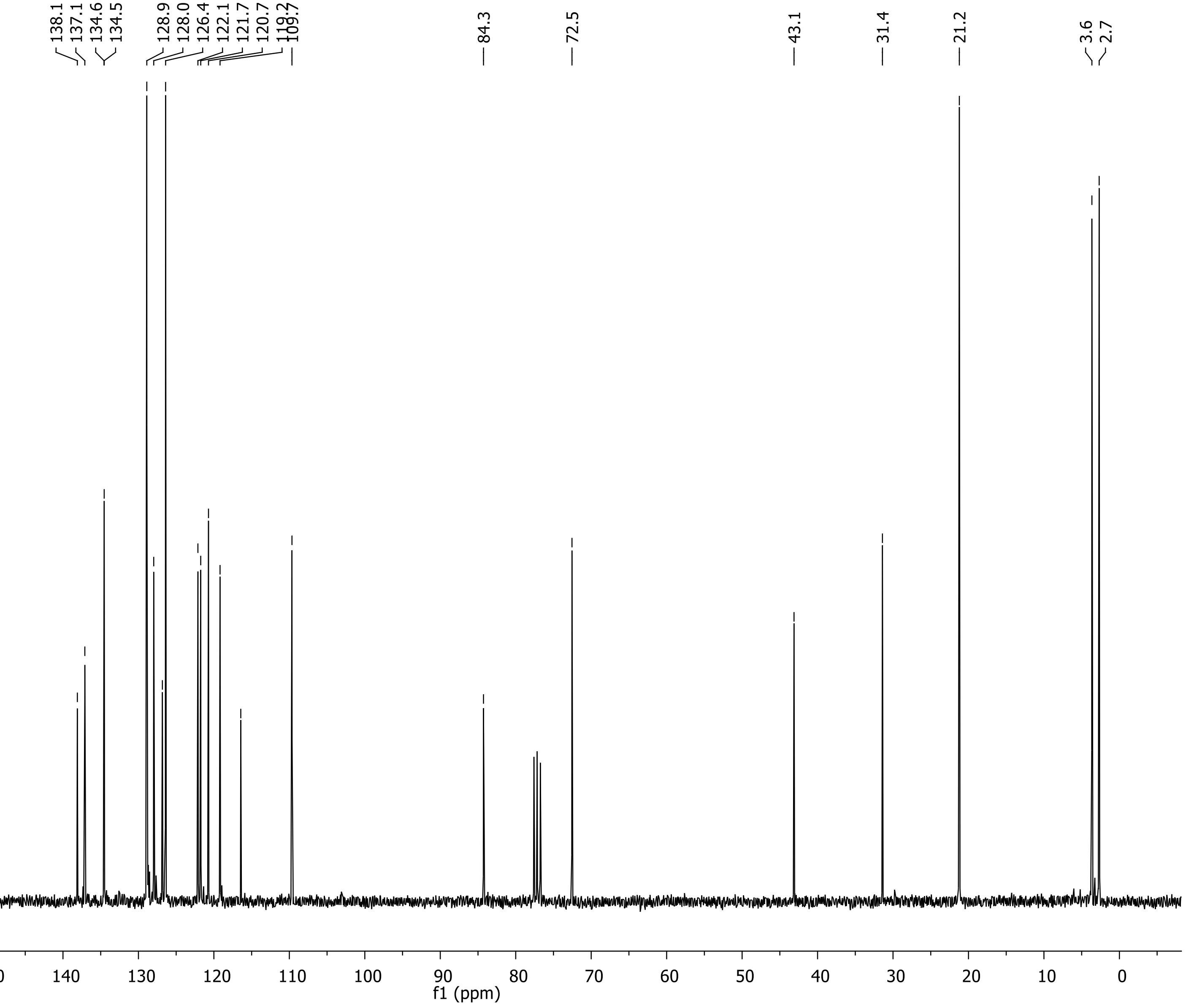

^{1}H NMR ($CDCl_3$, 300 MHz)

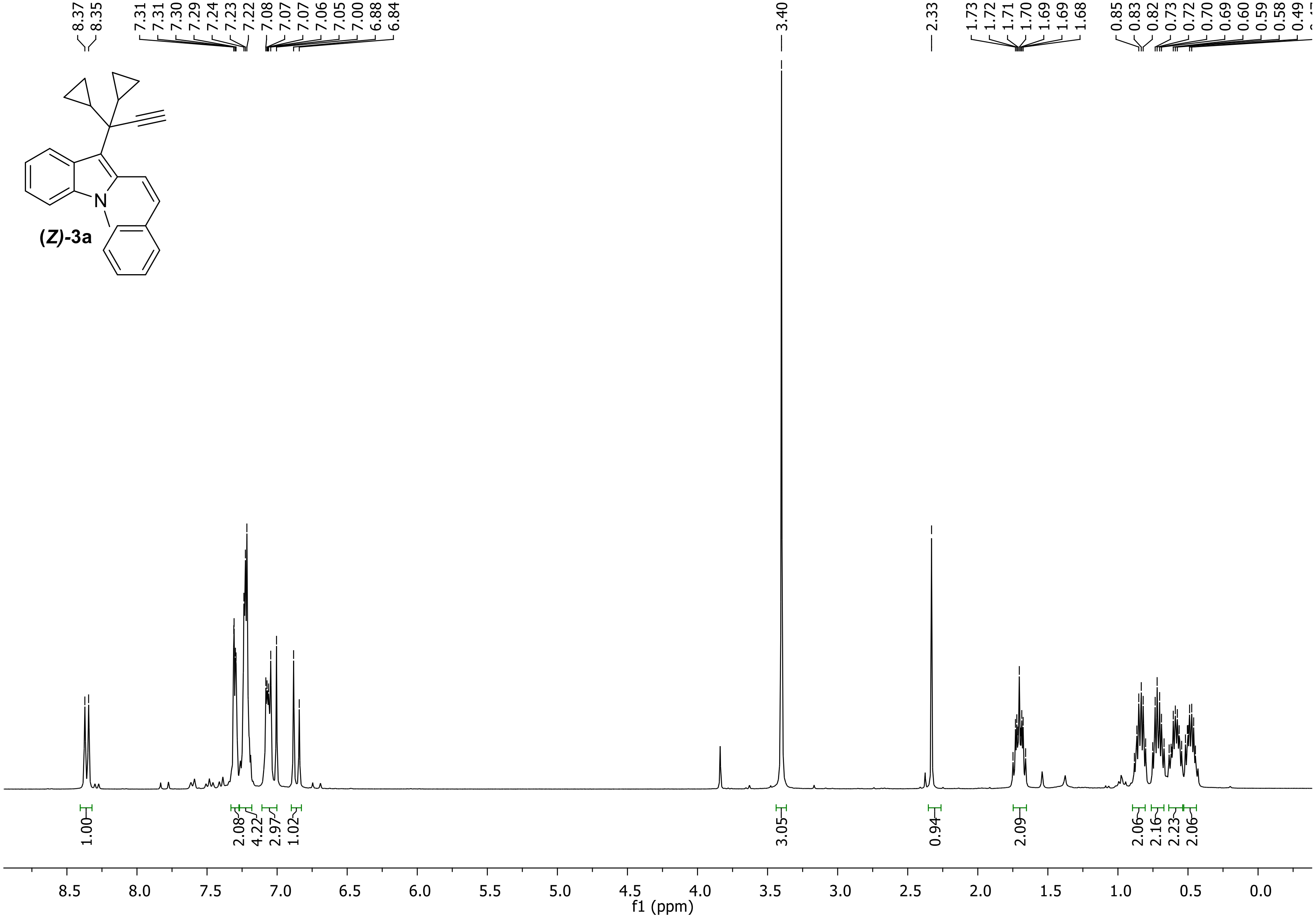

^{13}C NMR (CDCl$_3$, 75.4 MHz)

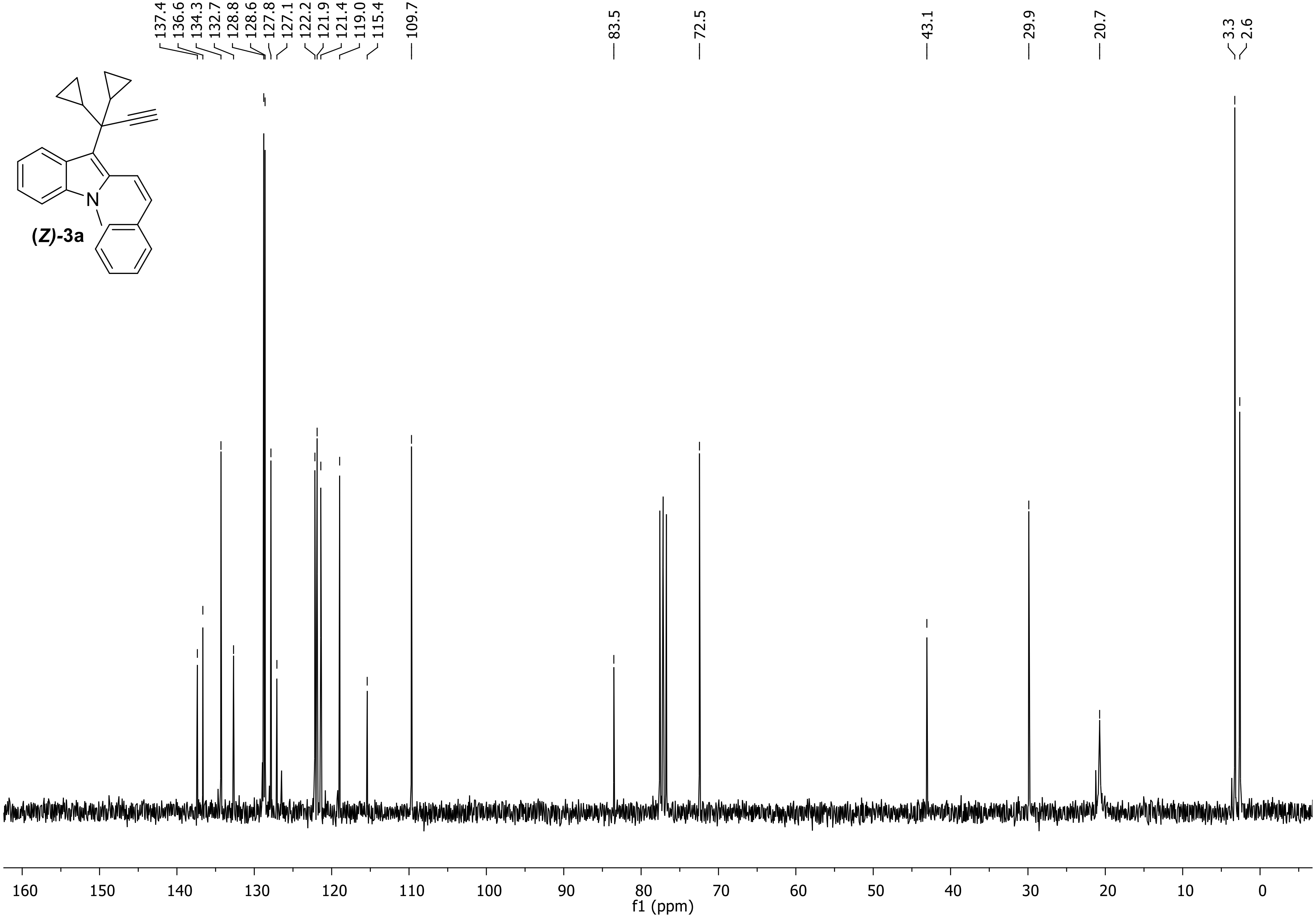

^{1}H NMR ($CDCl_3$, 300 MHz)

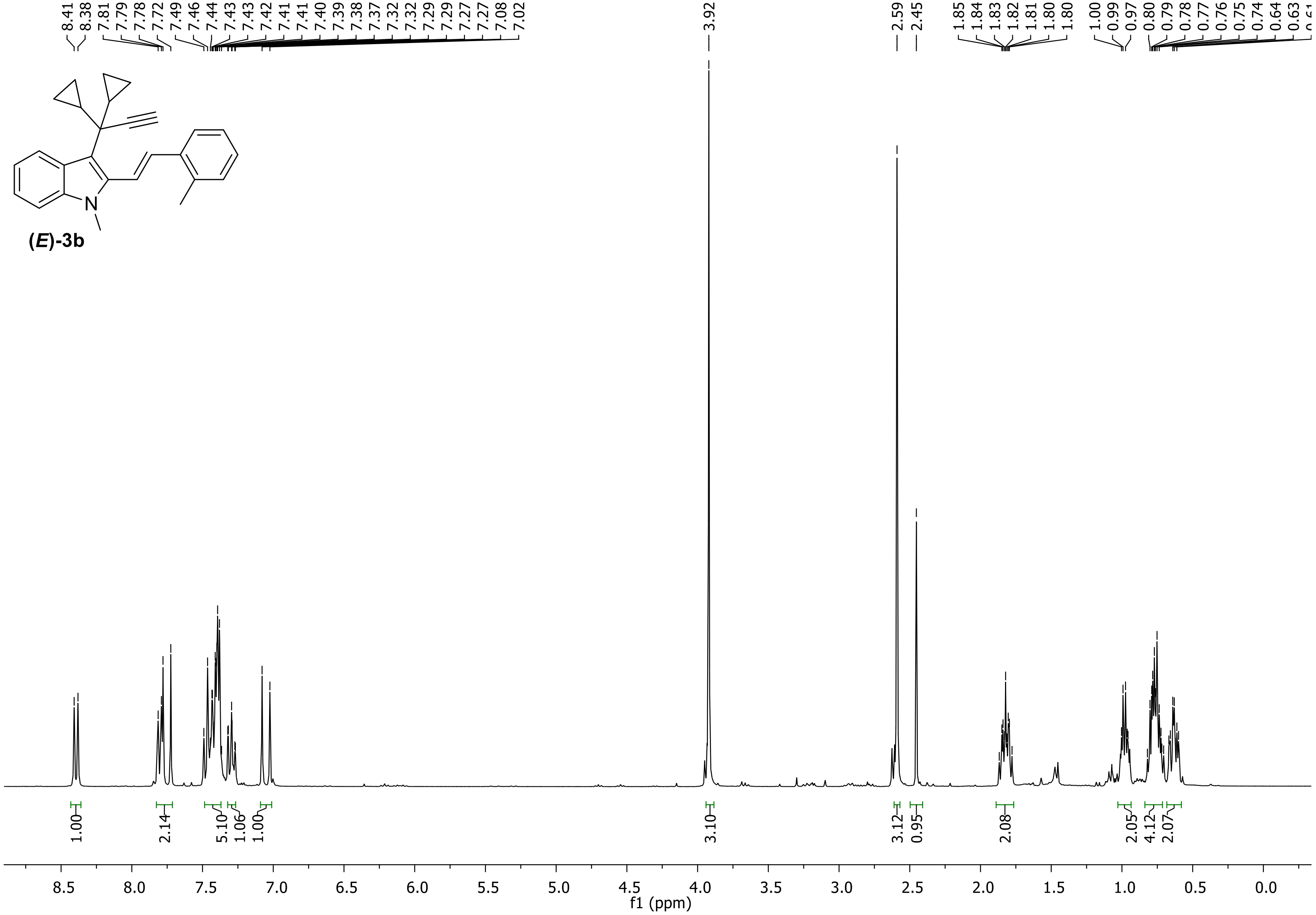

^{13}C NMR ($CDCl_3$, 75.4 MHz)

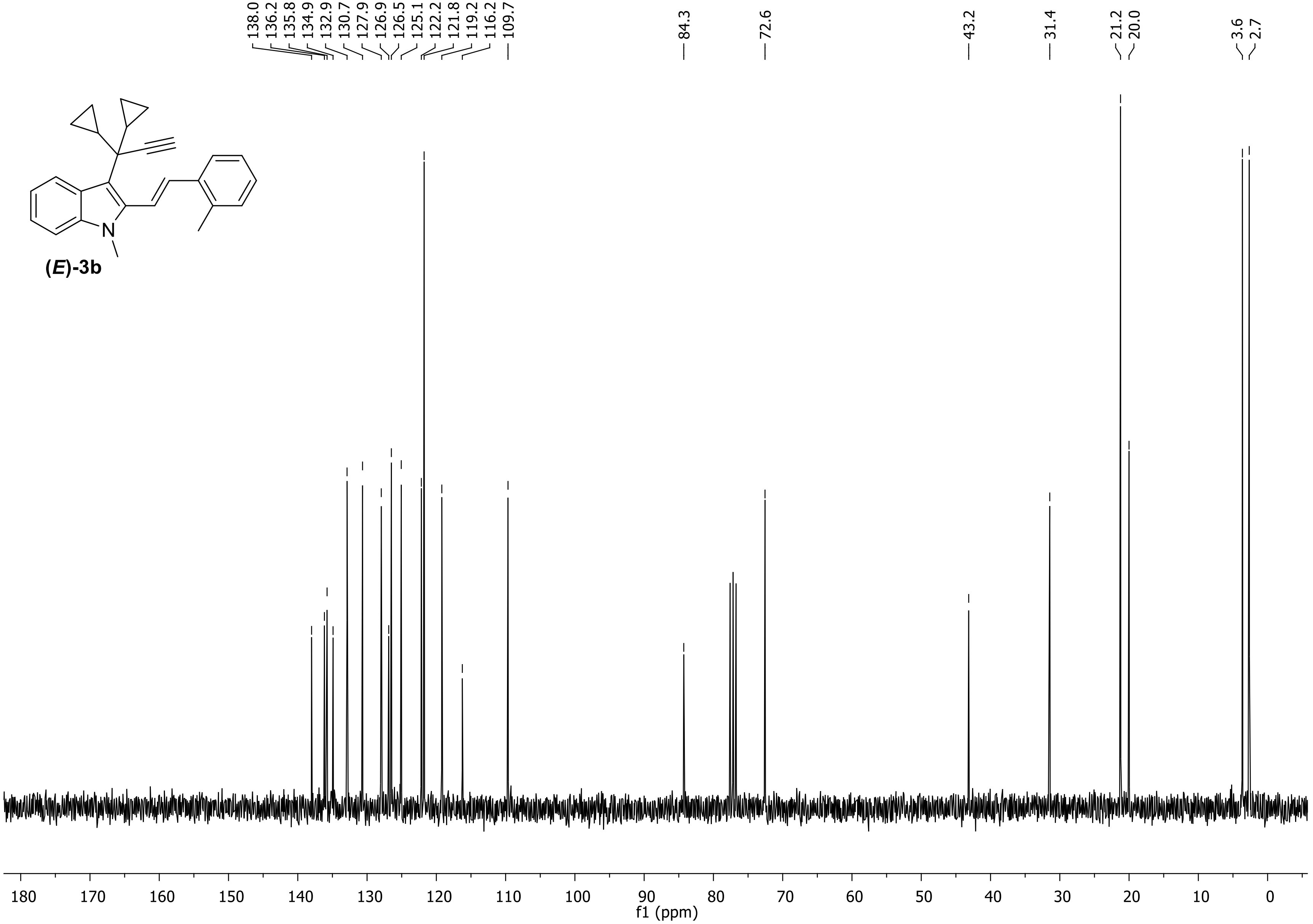

(*E*)-3b

^{1}H NMR ($CDCl_3$, 300 MHz)

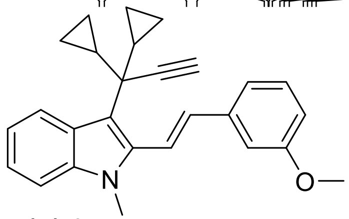

(*E*)-3c

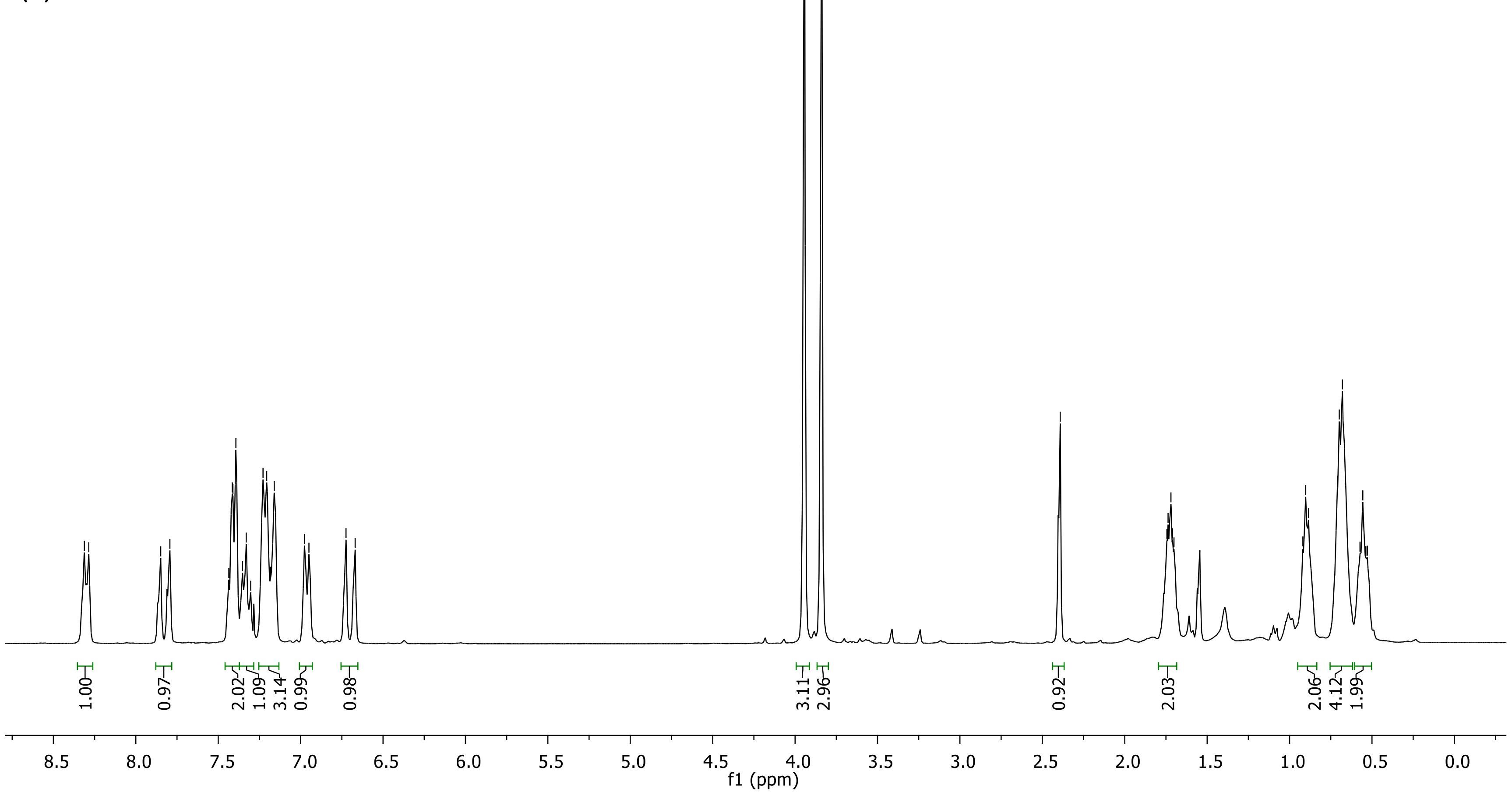

^{13}C NMR ($CDCl_3$, 75.4 MHz)

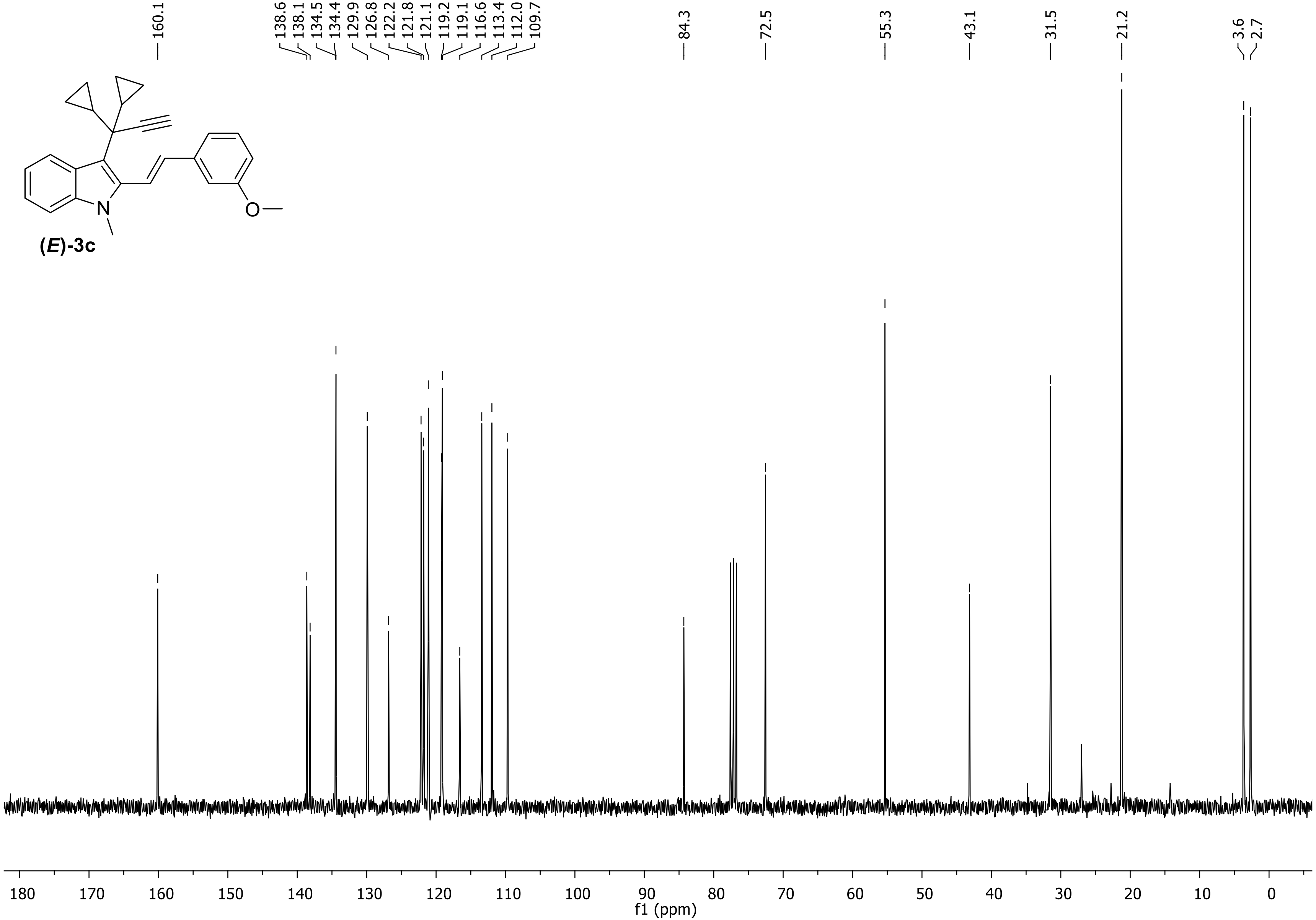

^{1}H NMR (CDCl$_3$, 300 MHz)

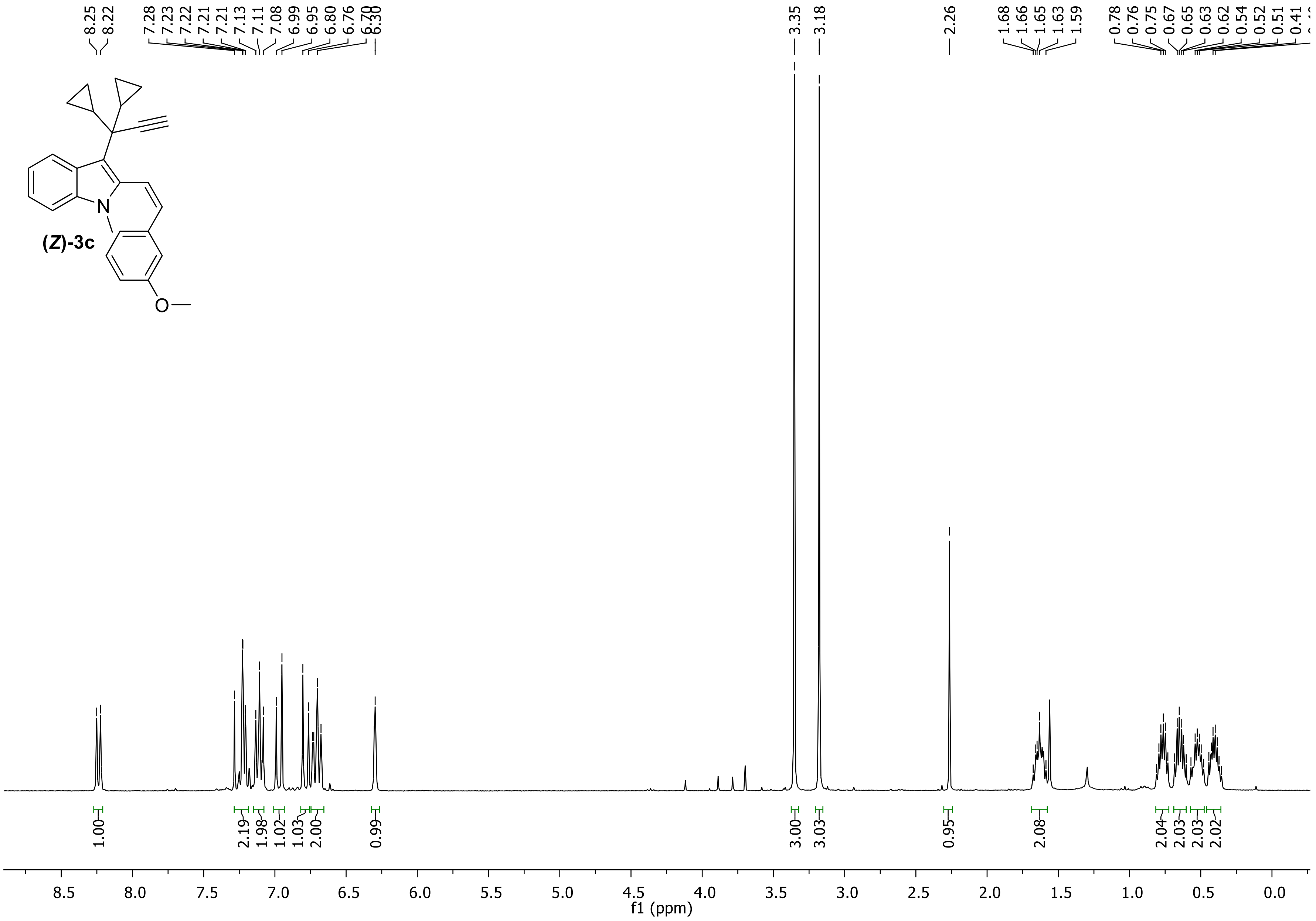

^{13}C NMR ($CDCl_3$, 75.4 MHz)

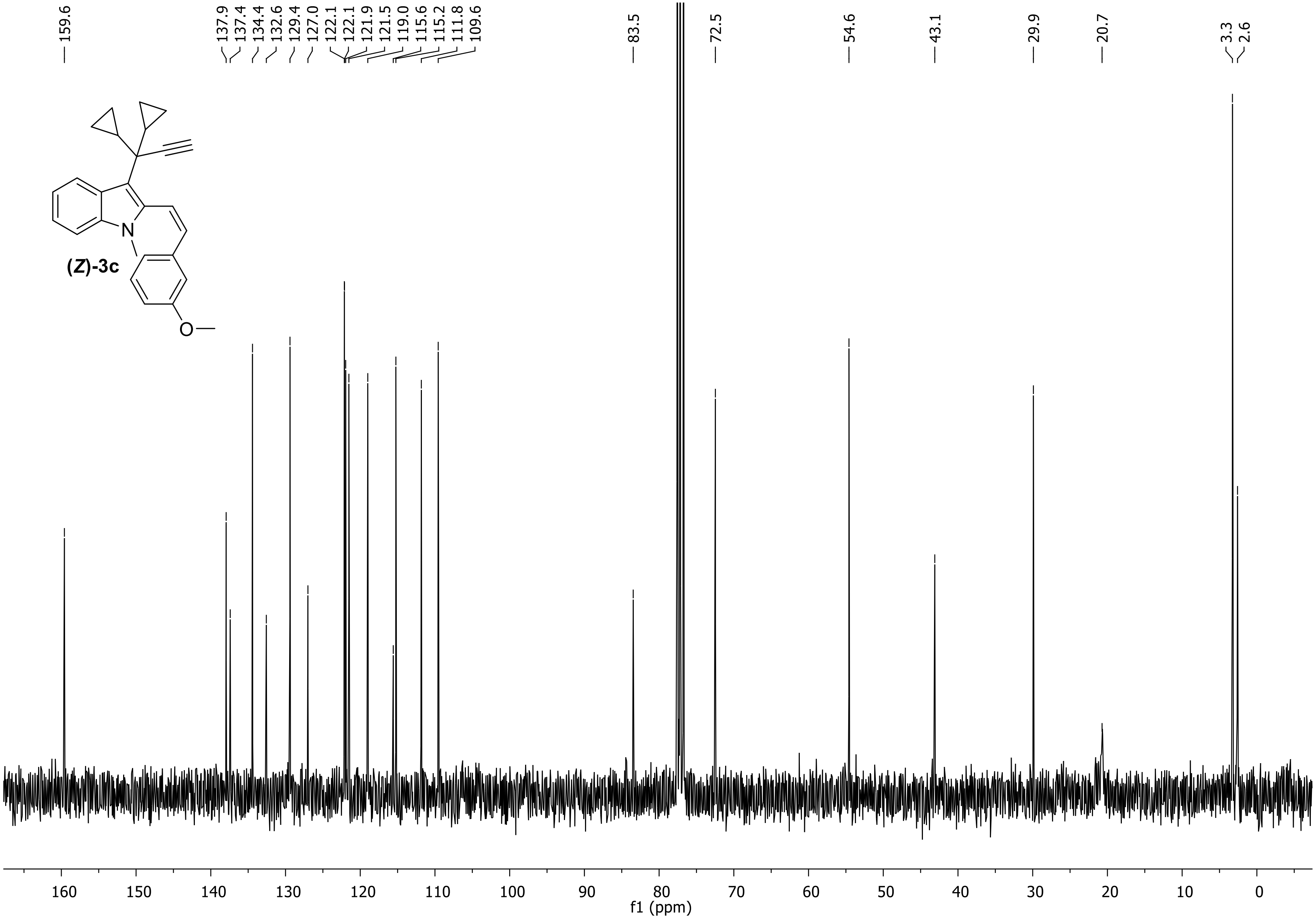

^{1}H NMR ($CDCl_3$, 300 MHz)

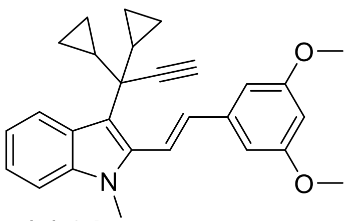

(*E*)-3d

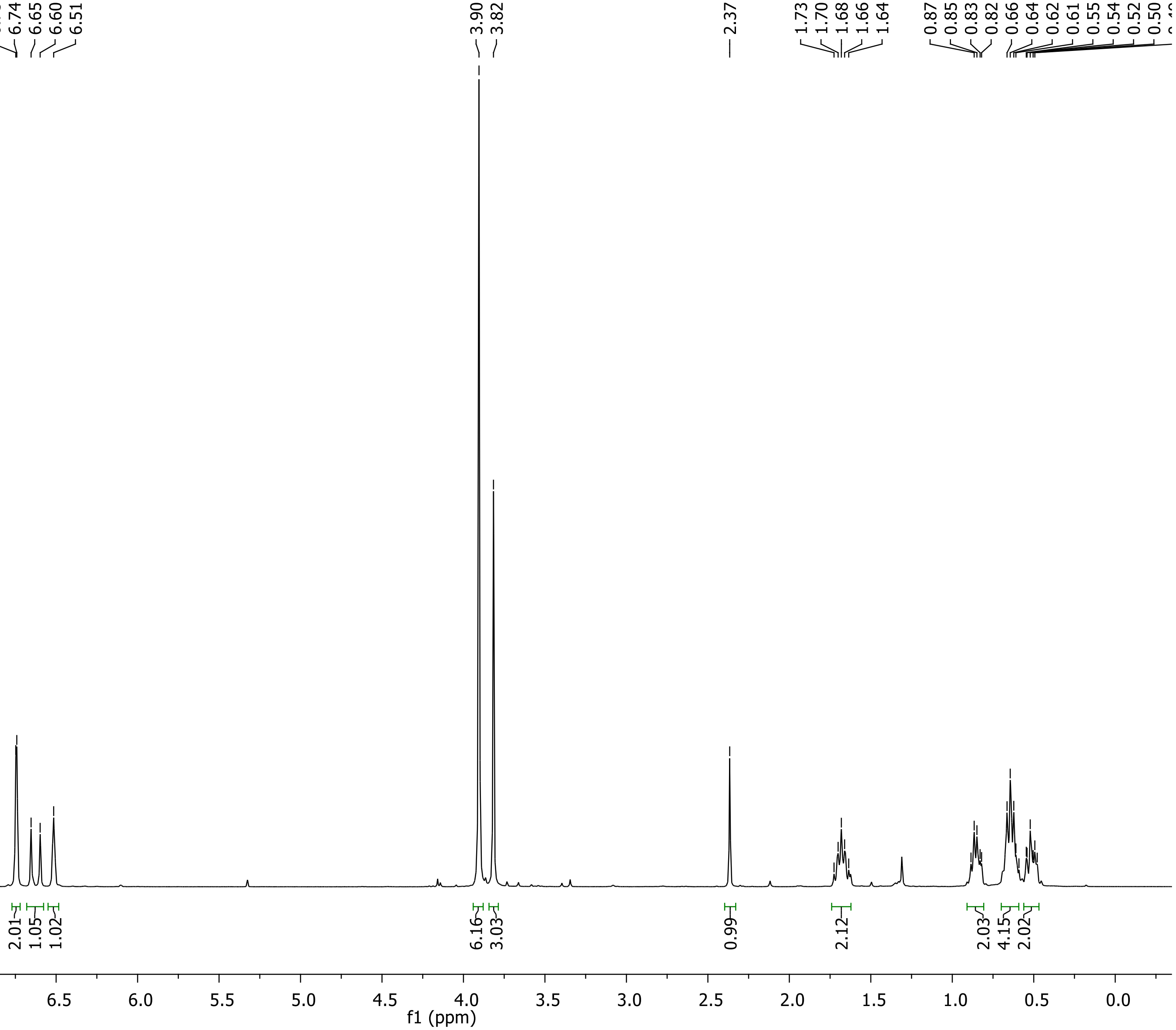

^{13}C NMR ($CDCl_3$, 75.4 MHz)

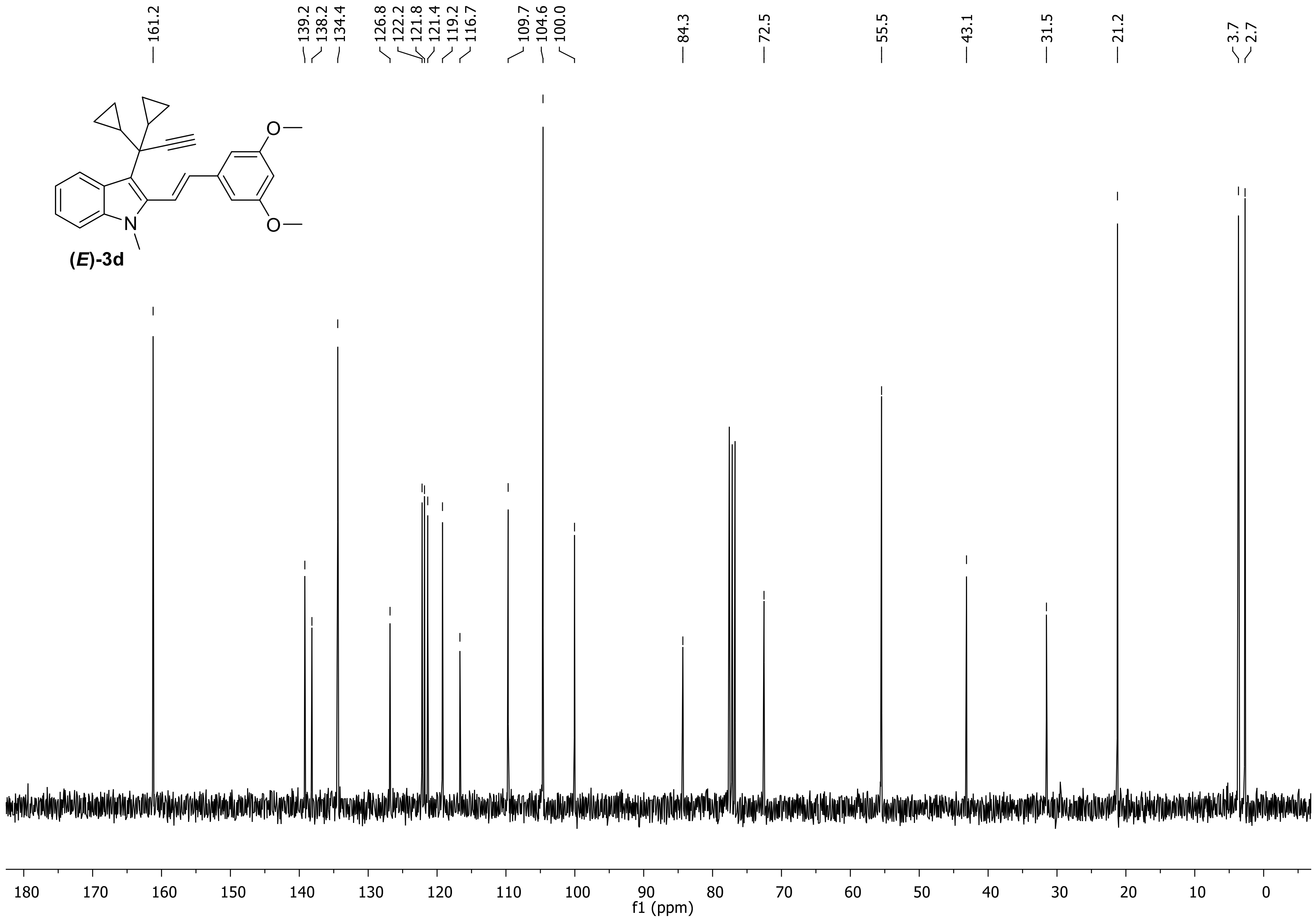

^{1}H NMR ($CDCl_3$, 300 MHz)

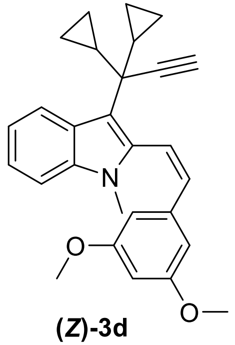

(*Z*)-3d
E/Z = 1/8

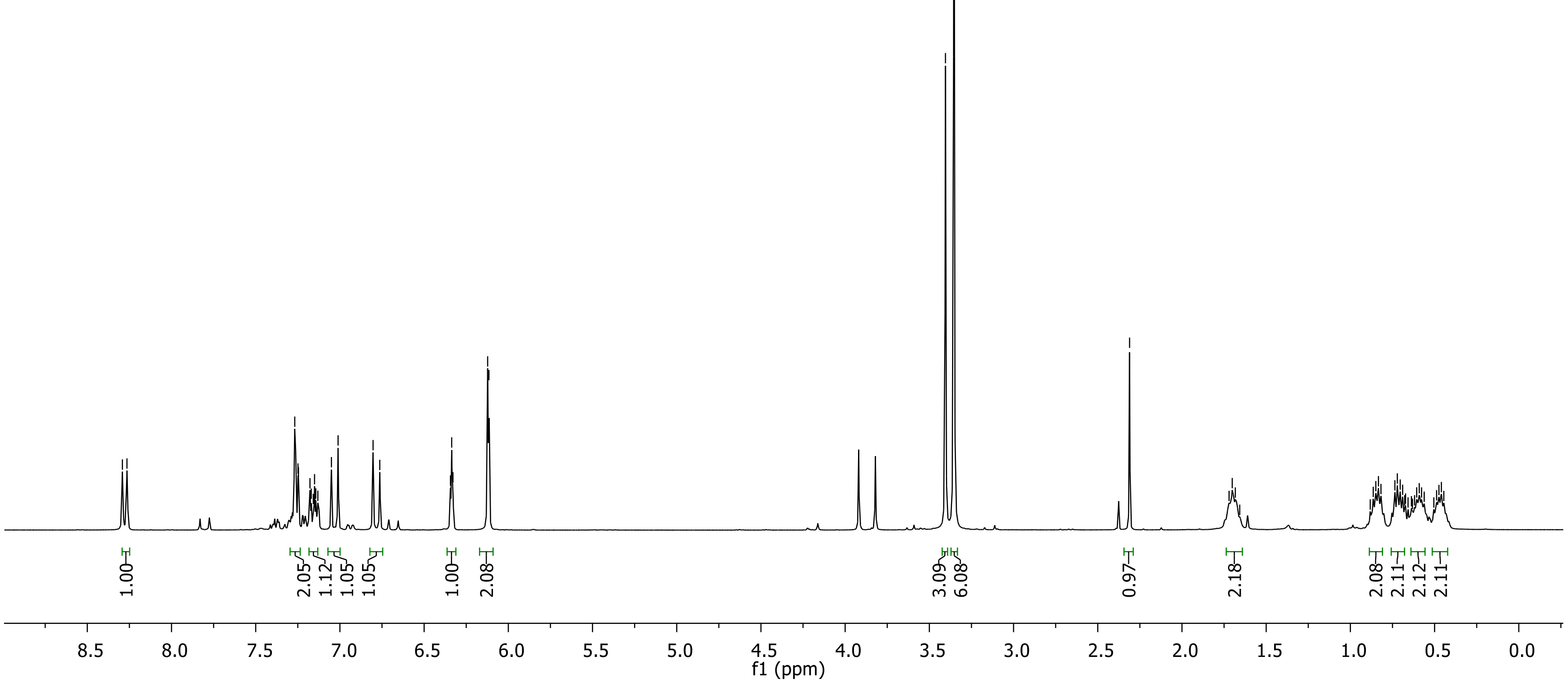

^{13}C NMR ($CDCl_3$, 75.4 MHz)

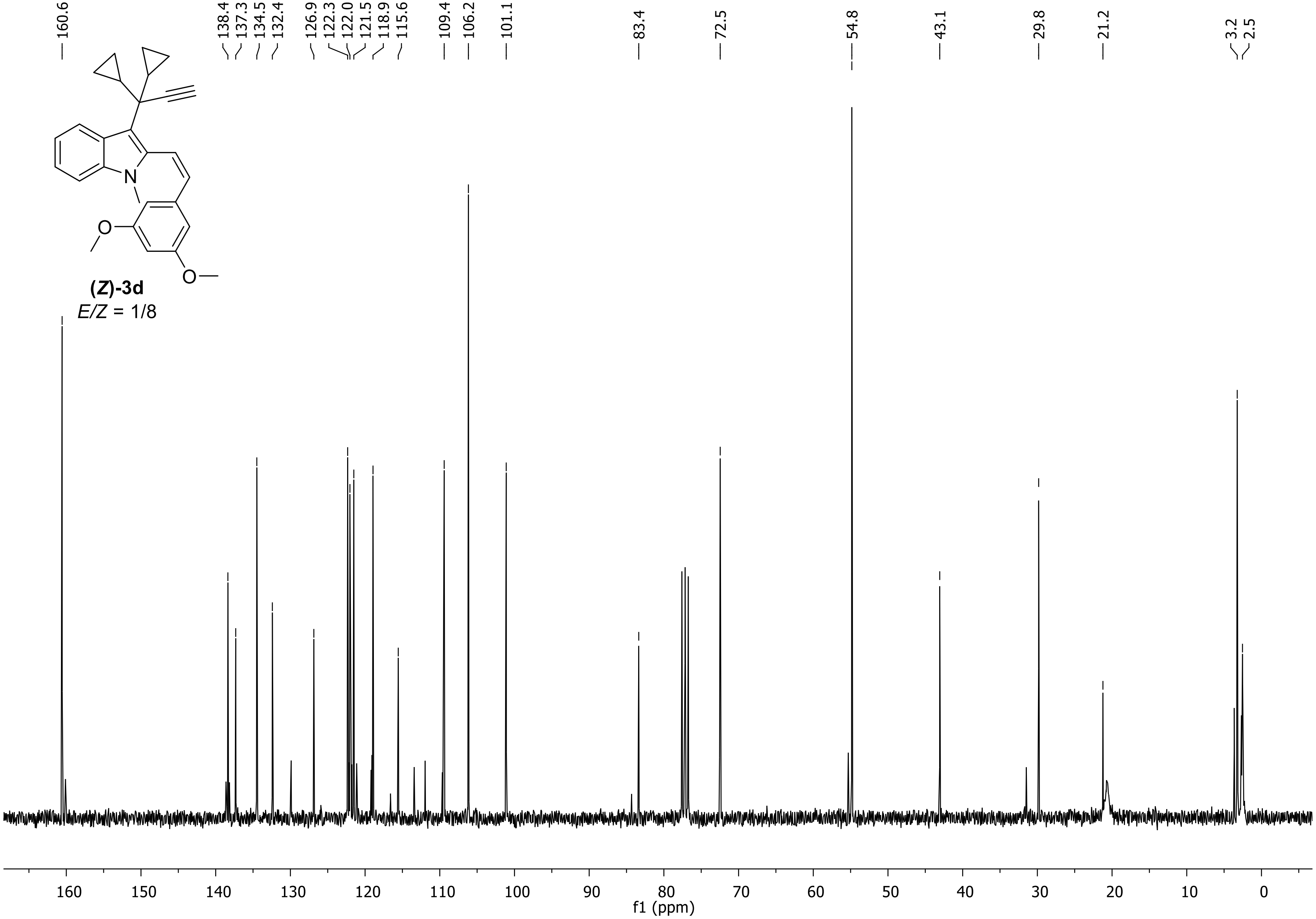

^{1}H NMR ($CDCl_3$, 300 MHz)

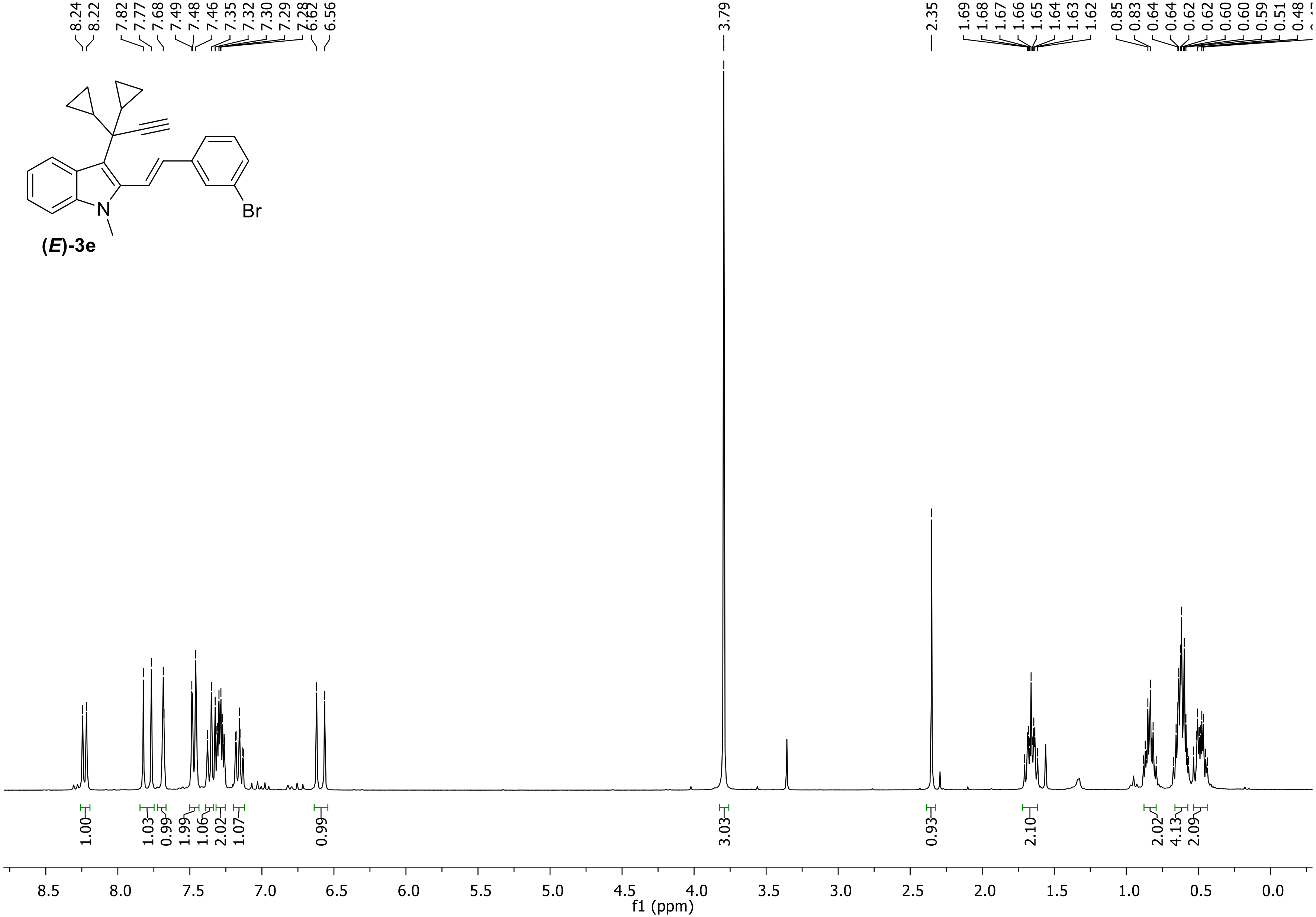

^{13}C NMR ($CDCl_3$, 75.4 MHz)

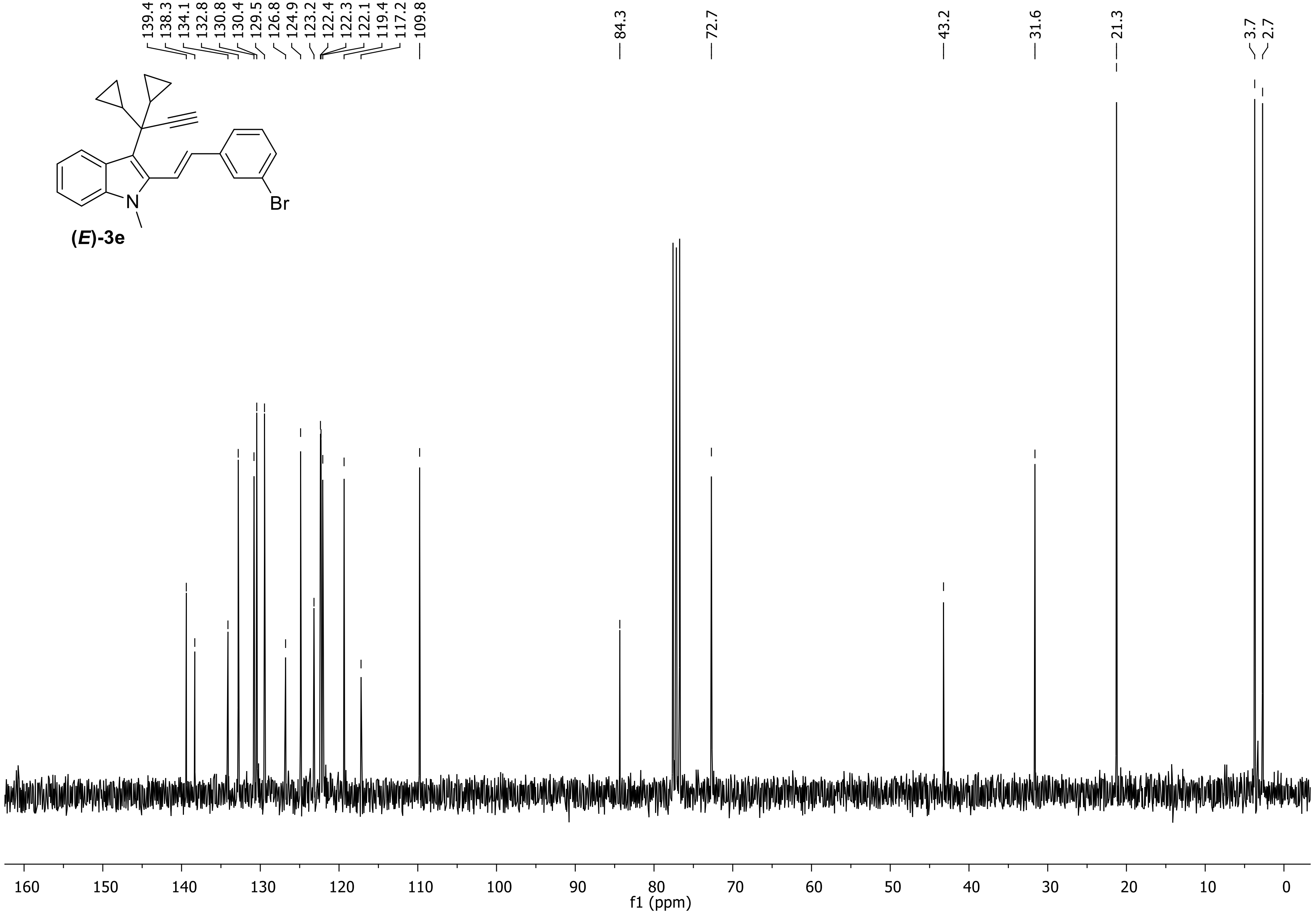

(*E*)-3e

^{1}H NMR ($CDCl_3$, 300 MHz)

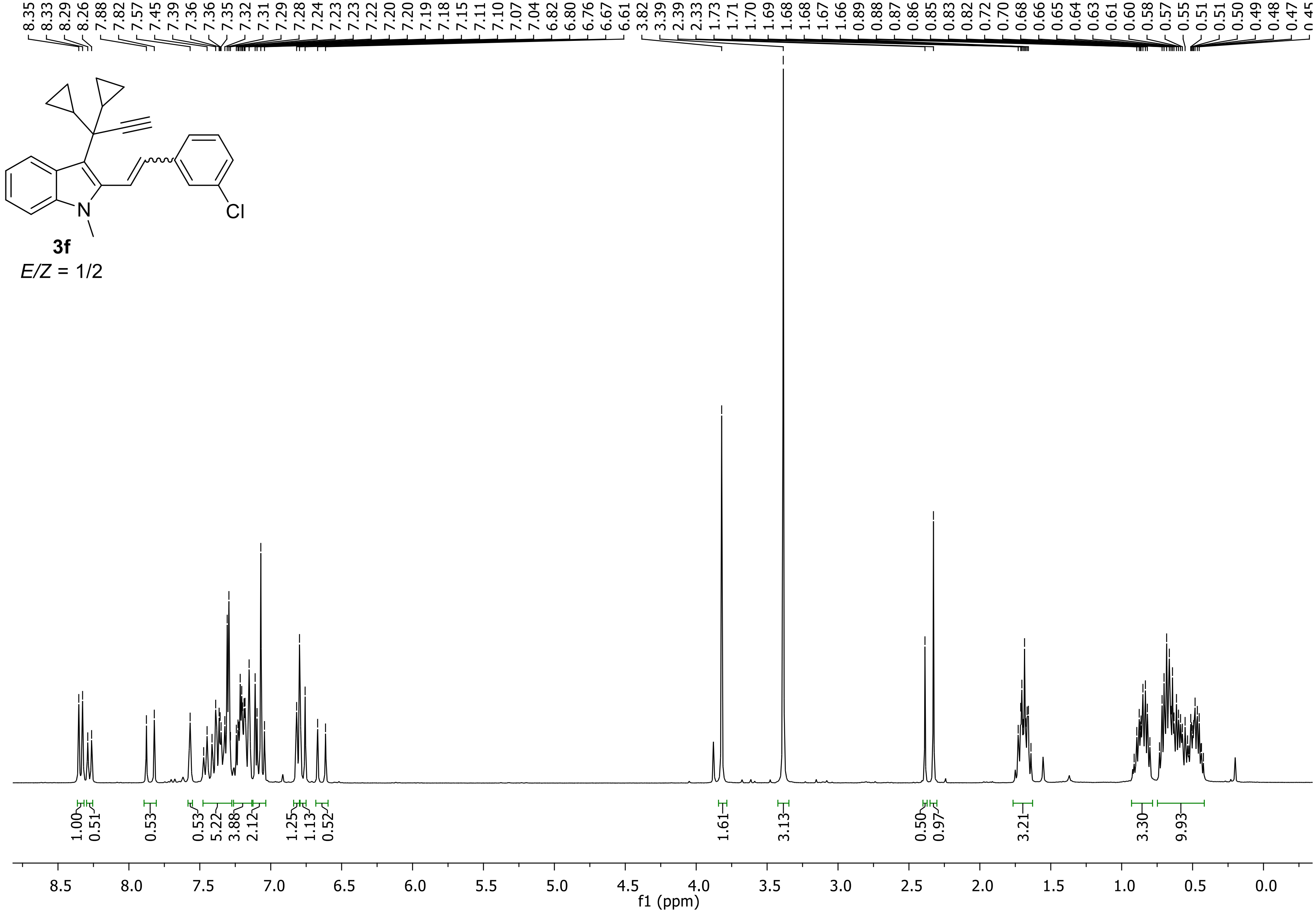

3f

E/Z = 1/2

^{13}C NMR ($CDCl_3$, 75.4 MHz)

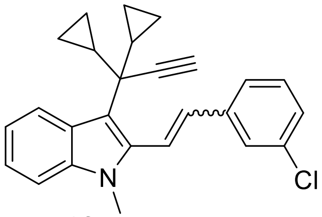

3f

E/Z = 1/2

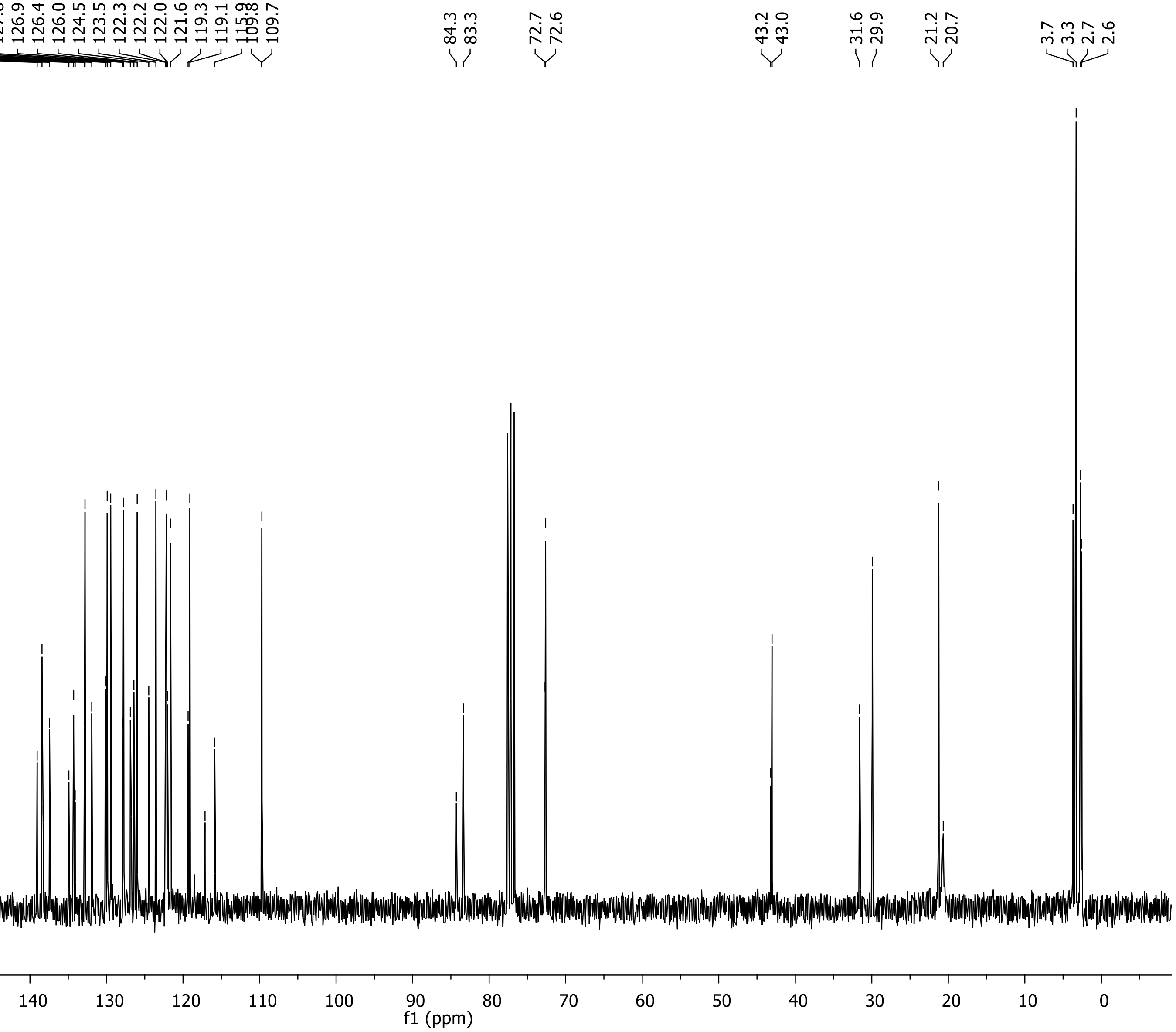

^1H NMR ($CDCl_3$, 300 MHz)

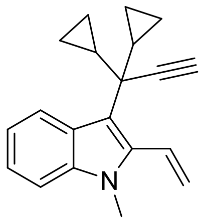

3g

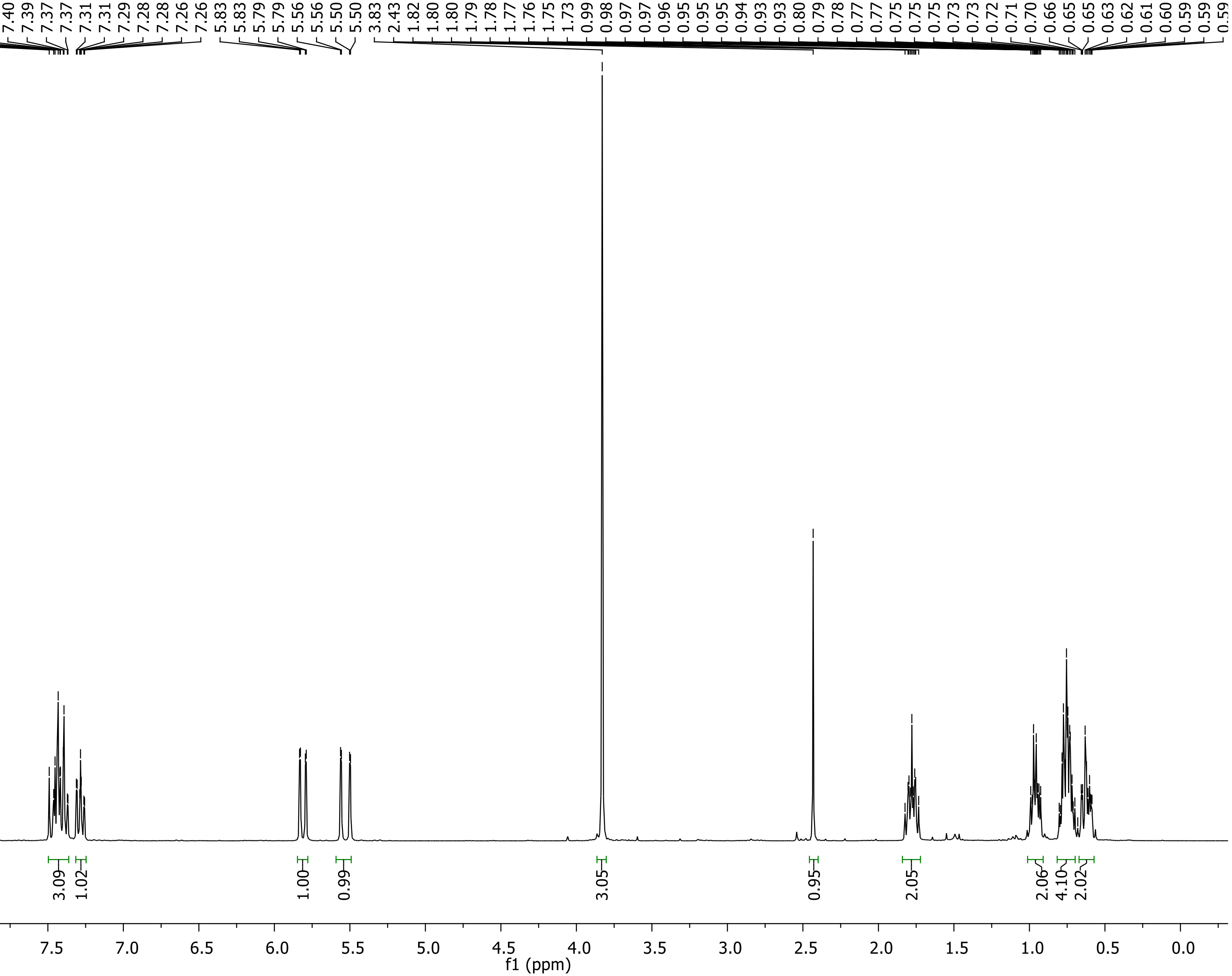

^{13}C NMR ($CDCl_3$, 75.4 MHz)

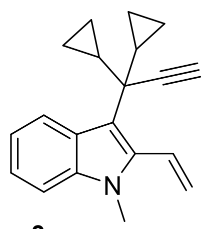

3g

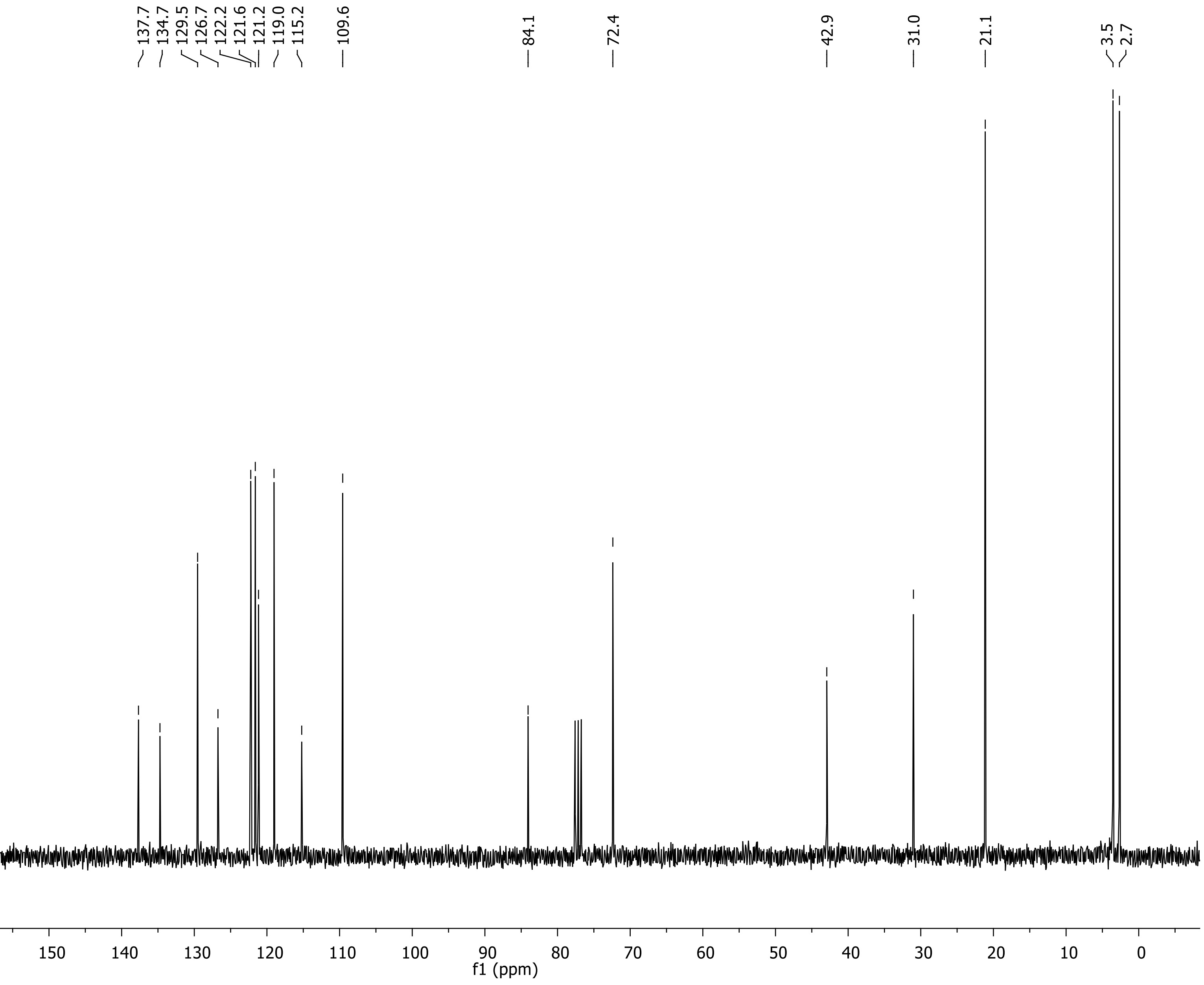

^{1}H NMR ($CDCl_3$, 300 MHz)

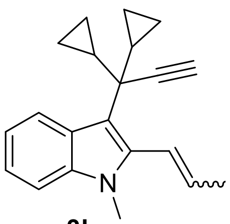

3h

E/Z = 1.4/1

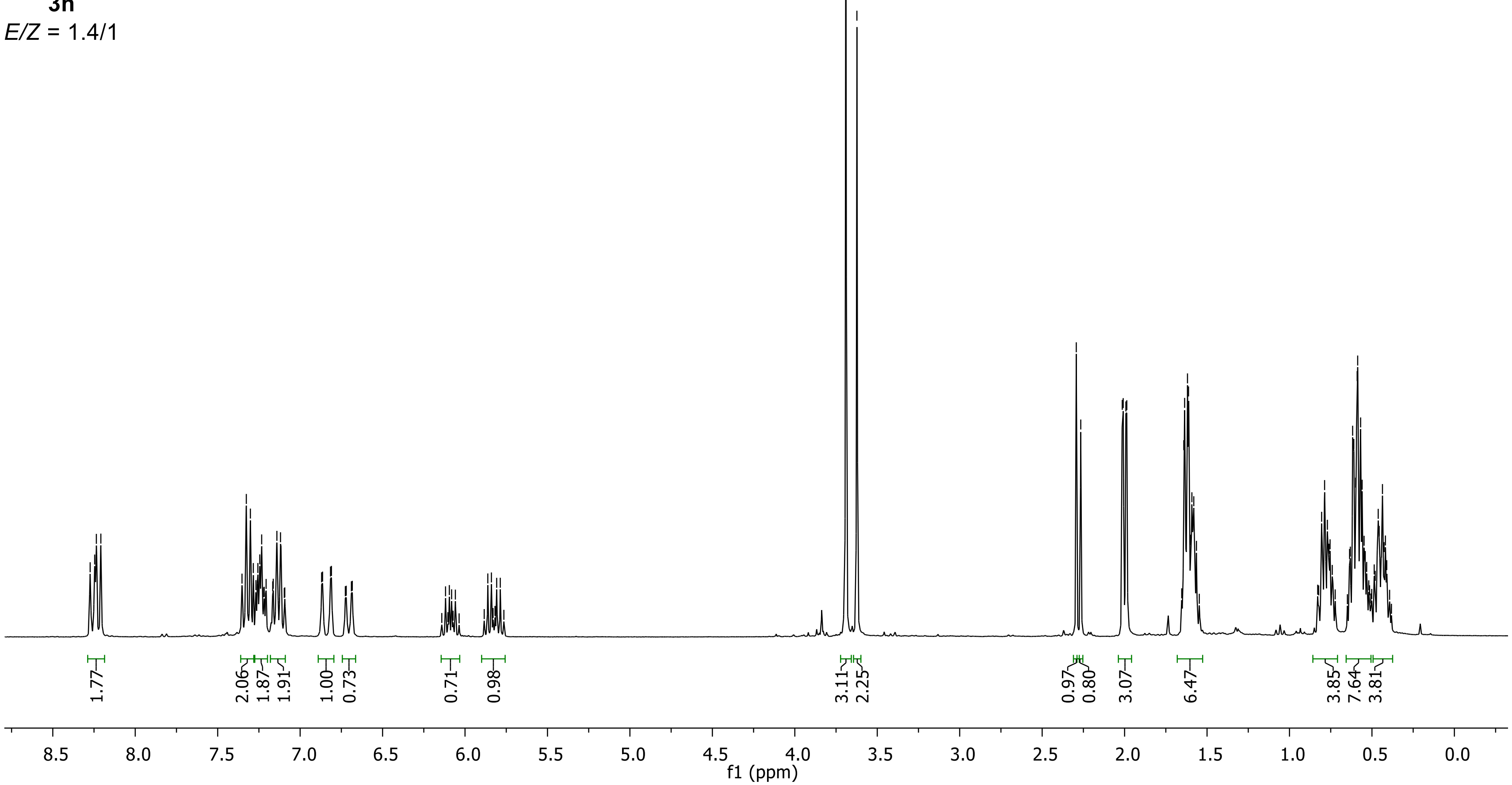

^{13}C NMR ($CDCl_3$, 75.4 MHz)

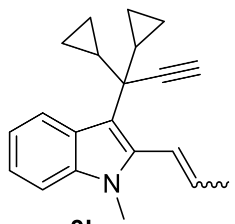

3h

E/Z = 1.4/1

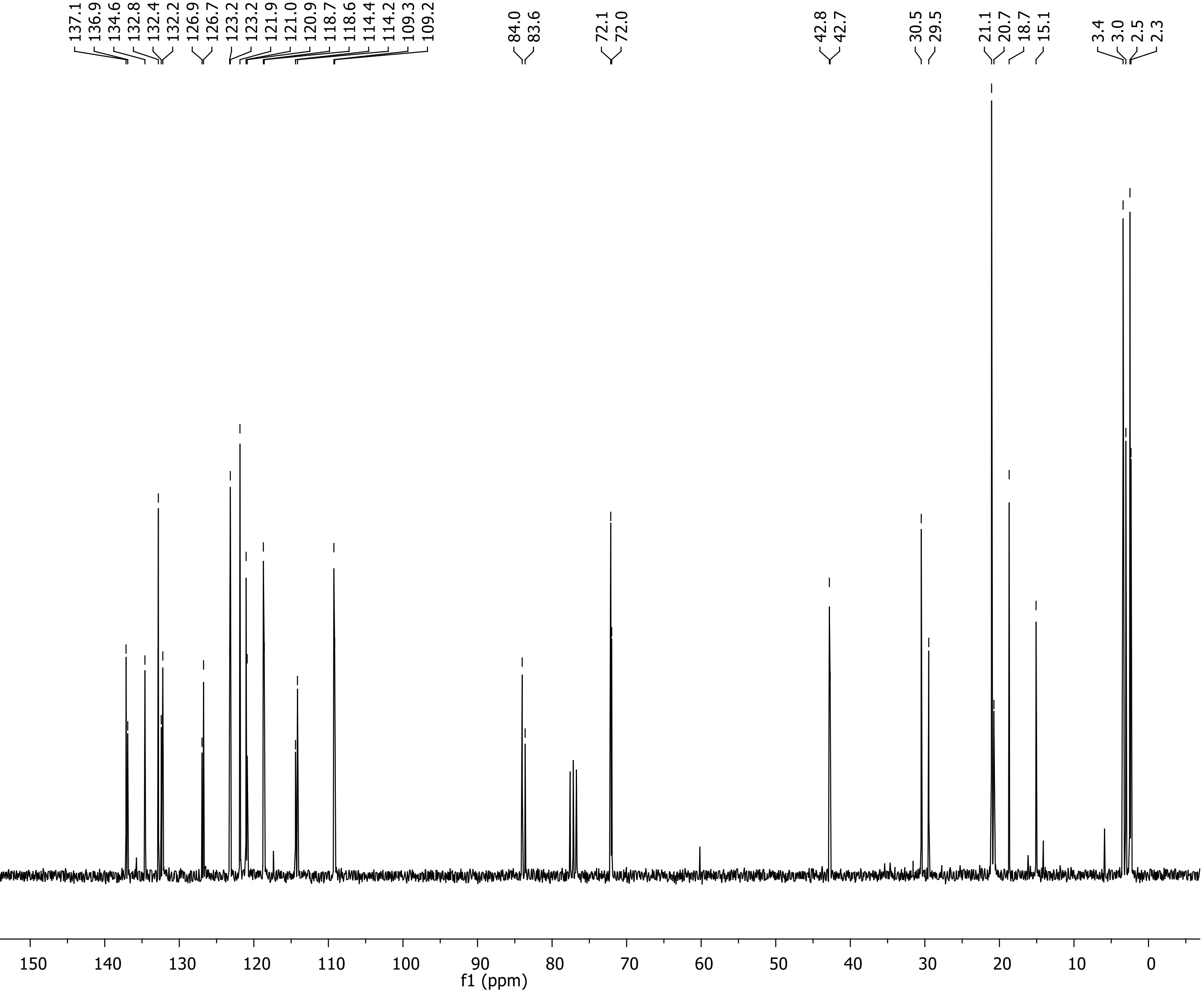

^{1}H NMR ($CDCl_3$, 300 MHz)

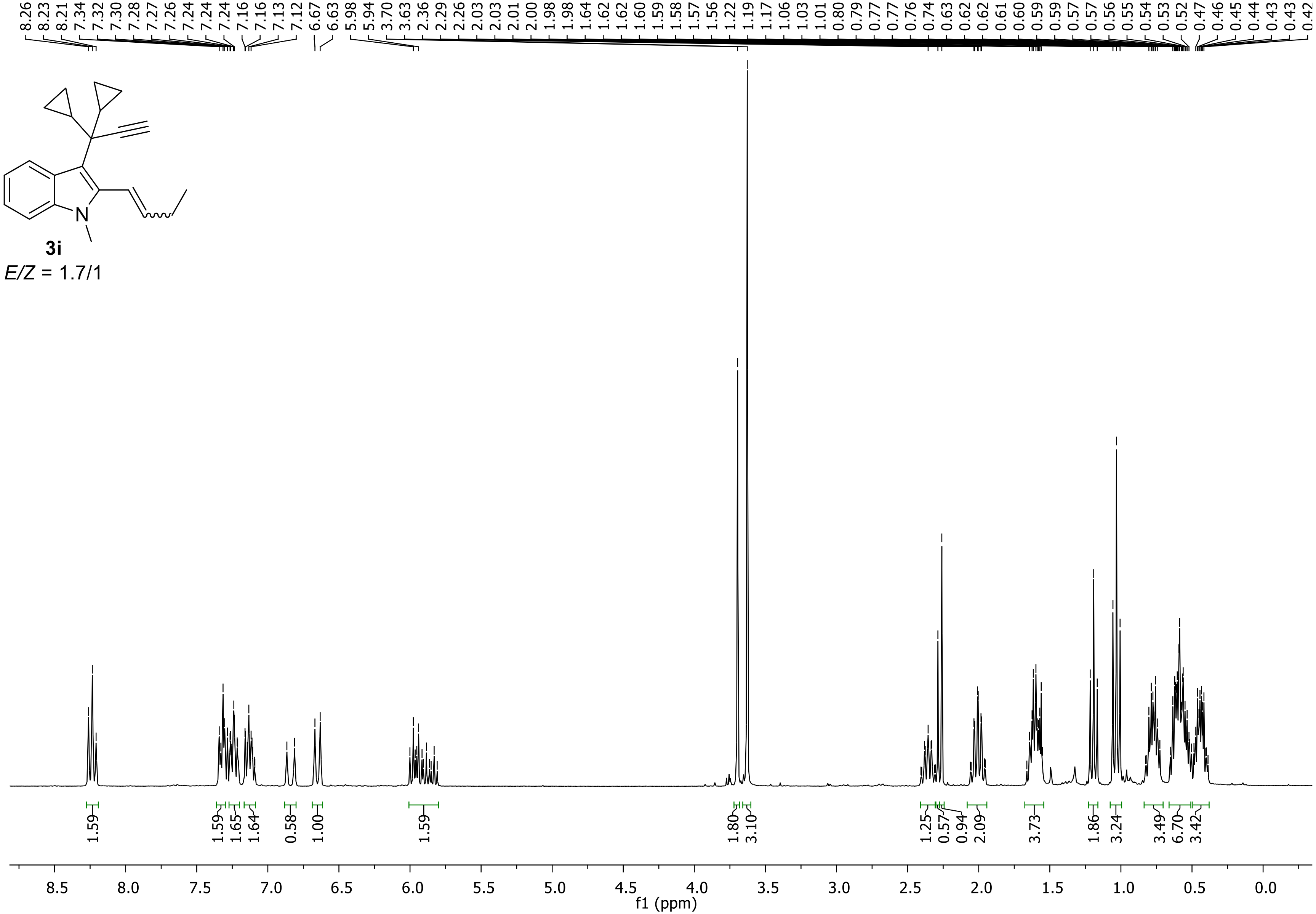

^{13}C NMR ($CDCl_3$, 75.4 MHz)

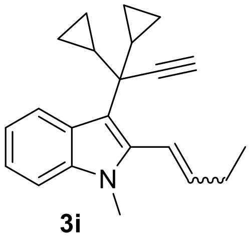

3i

E/*Z* = 1.7/1

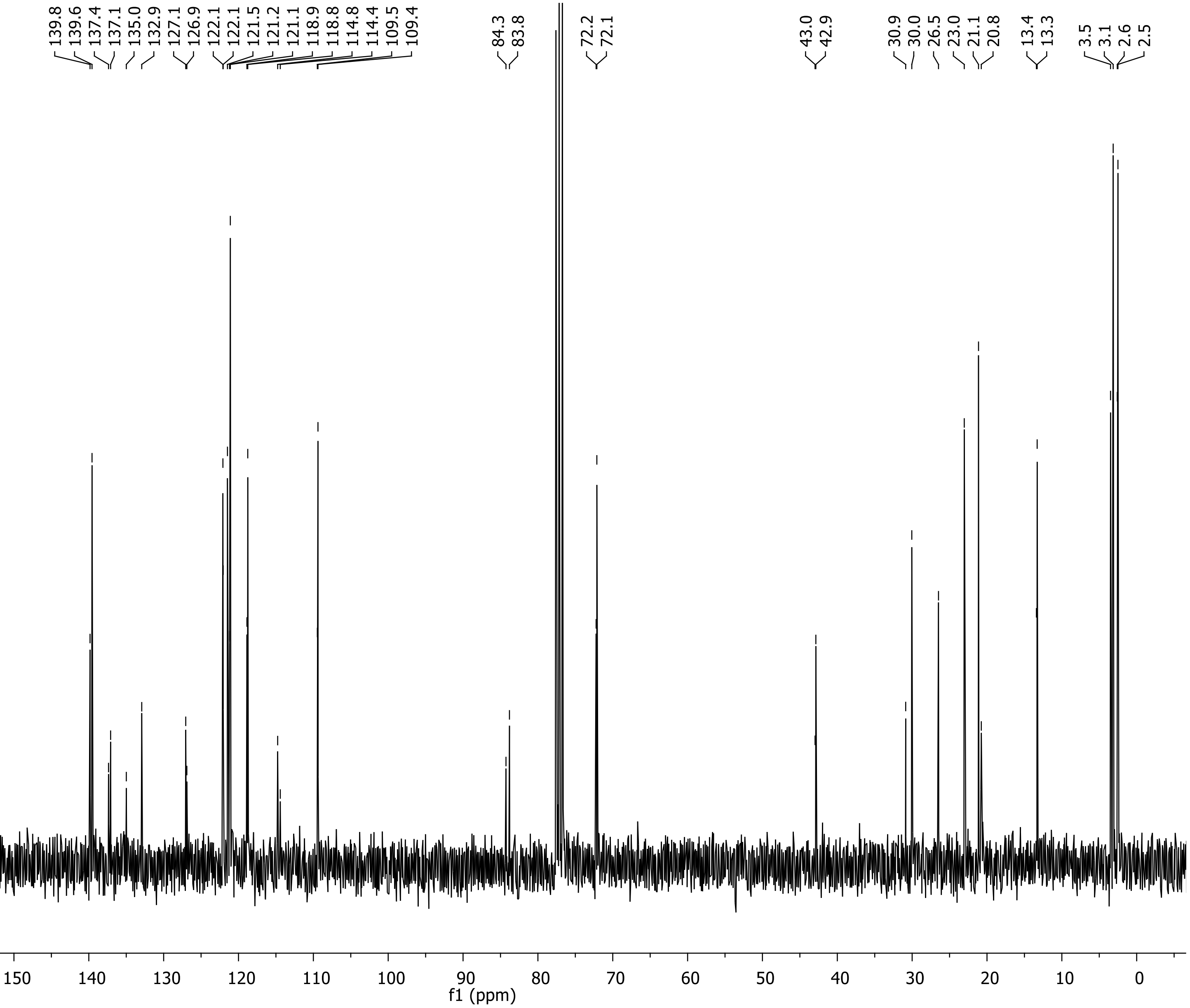

^1H NMR ($CDCl_3$, 300 MHz)

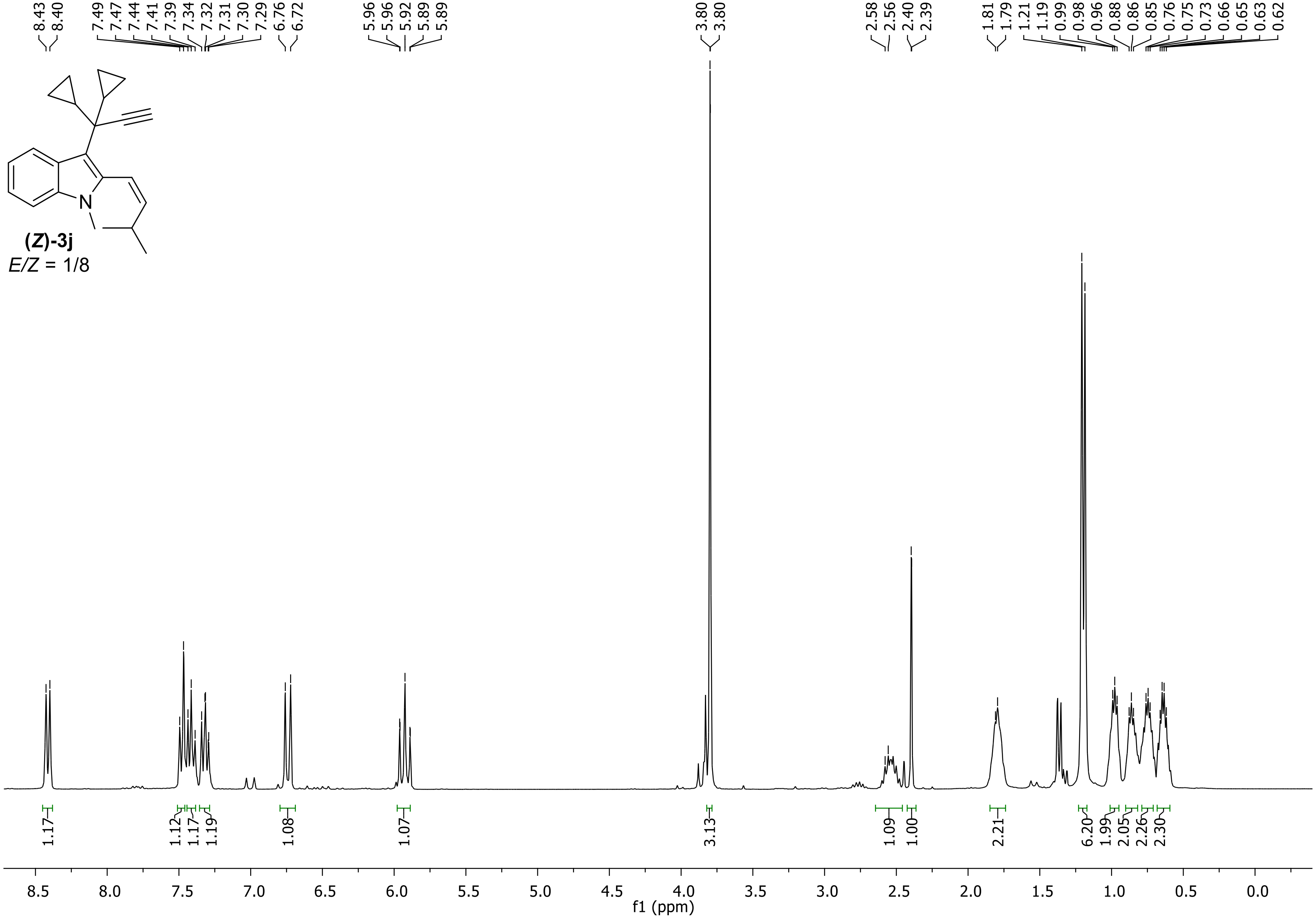

(*Z*)-3j

E/Z = 1/8

^{13}C NMR ($CDCl_3$, 75.4 MHz)

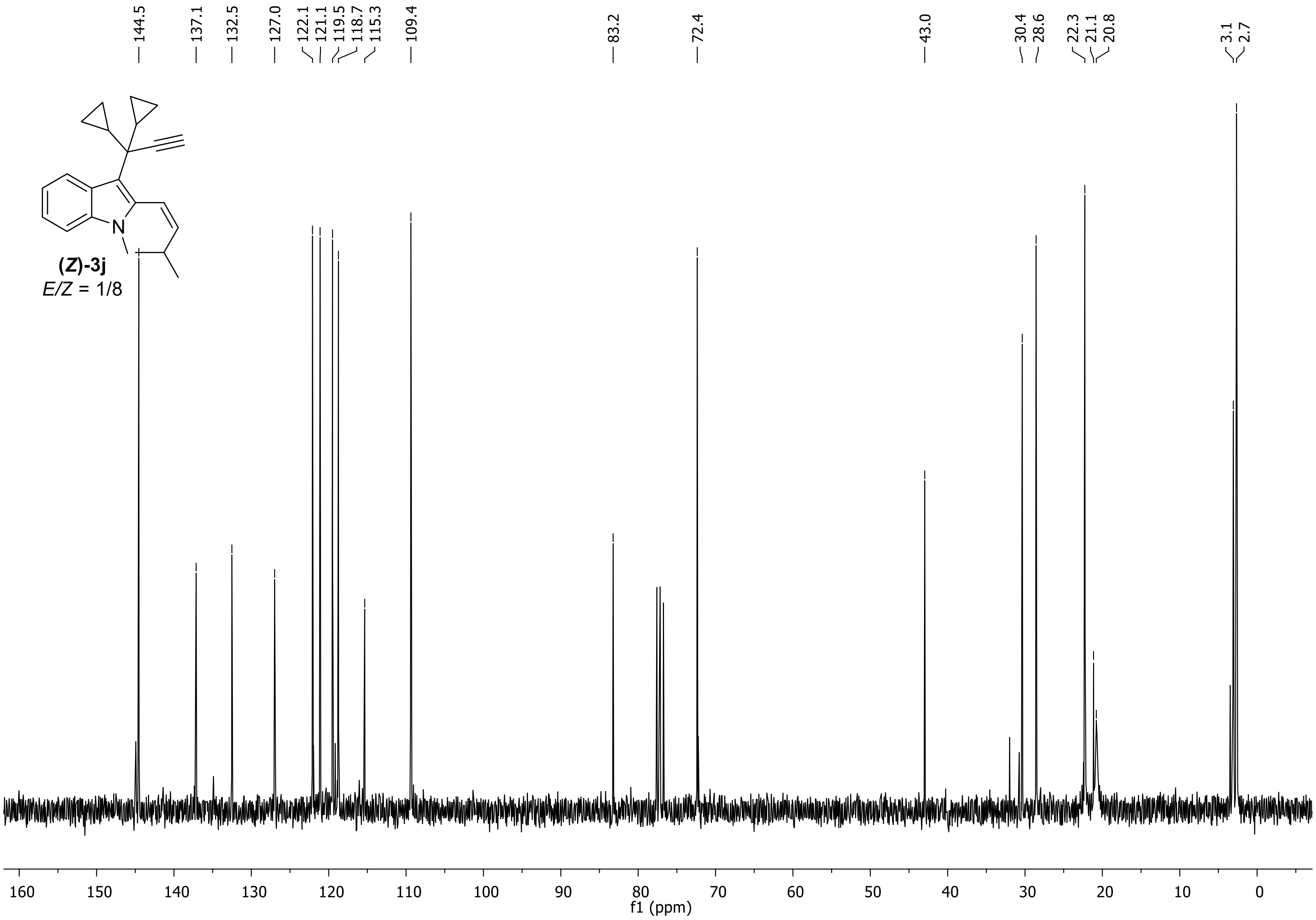

¹H NMR (CDCl$_3$, 300 MHz)

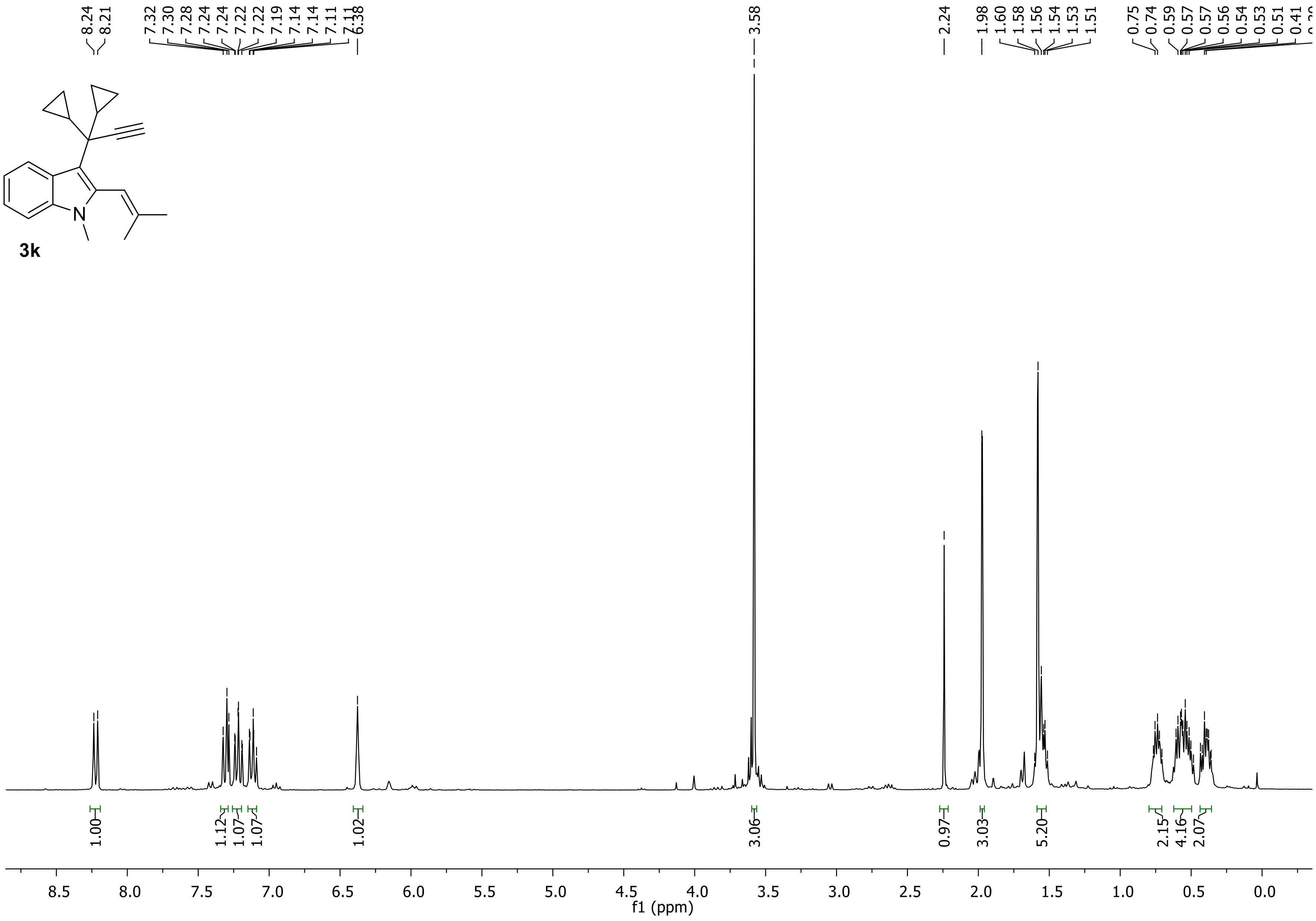

3k

^{13}C NMR (CDCl$_3$, 75.4 MHz)

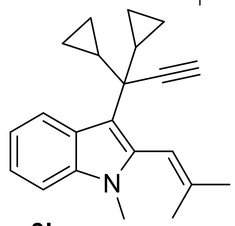

3k

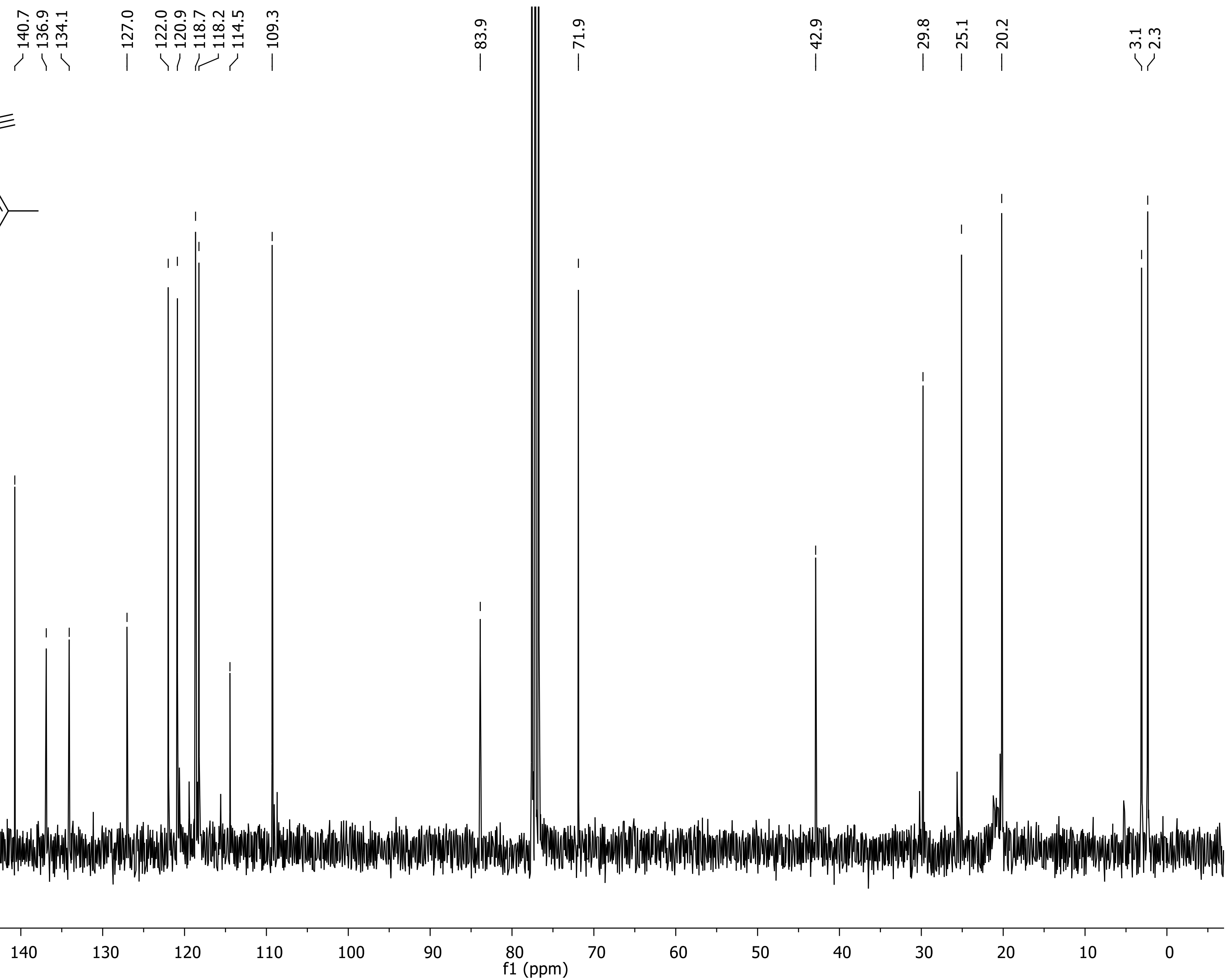

^{1}H NMR ($CDCl_3$, 300 MHz)

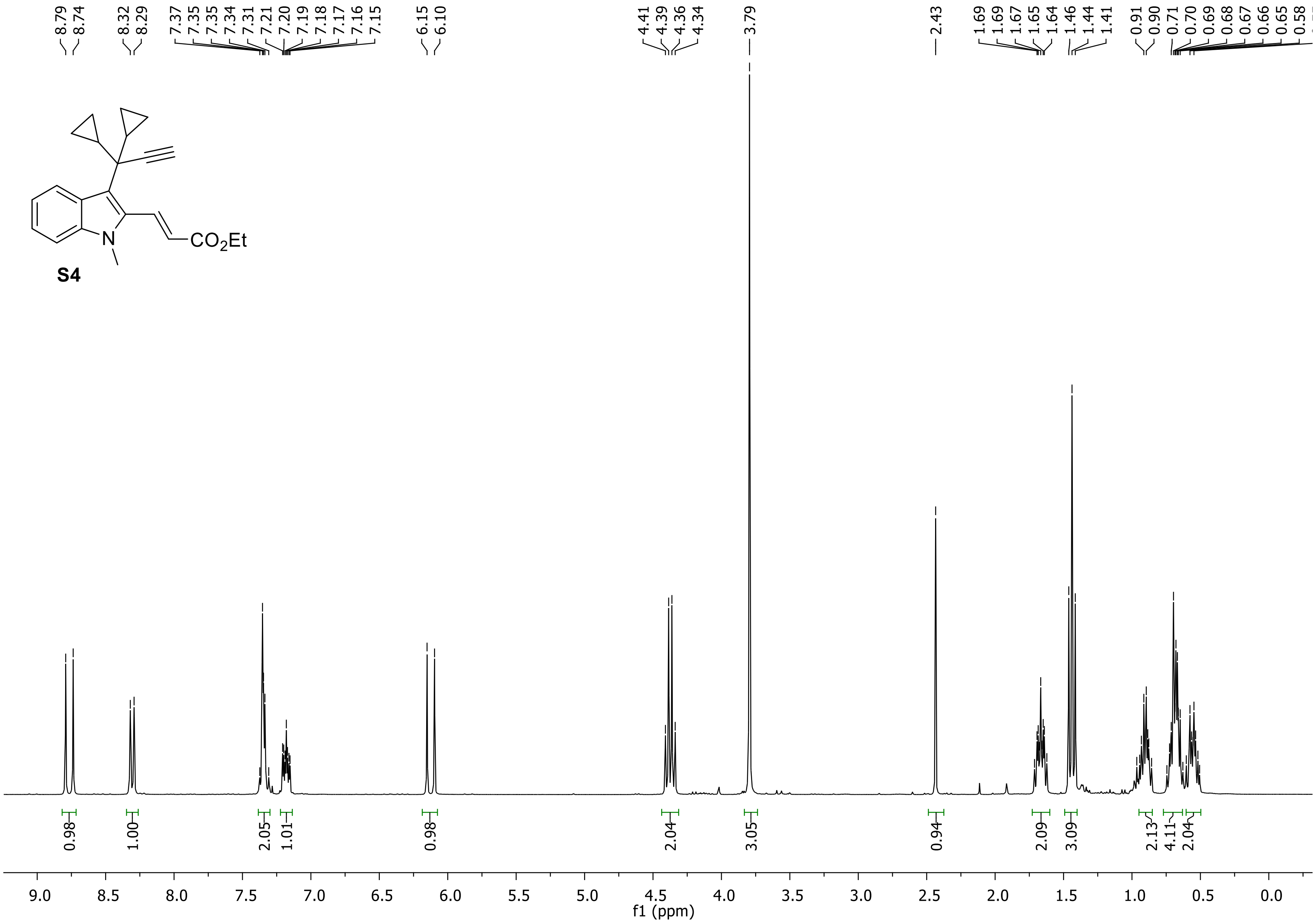

^{13}C NMR ($CDCl_3$, 75.4 MHz)

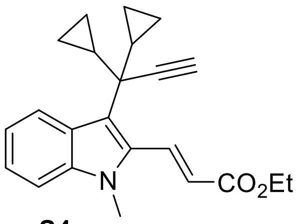

S4

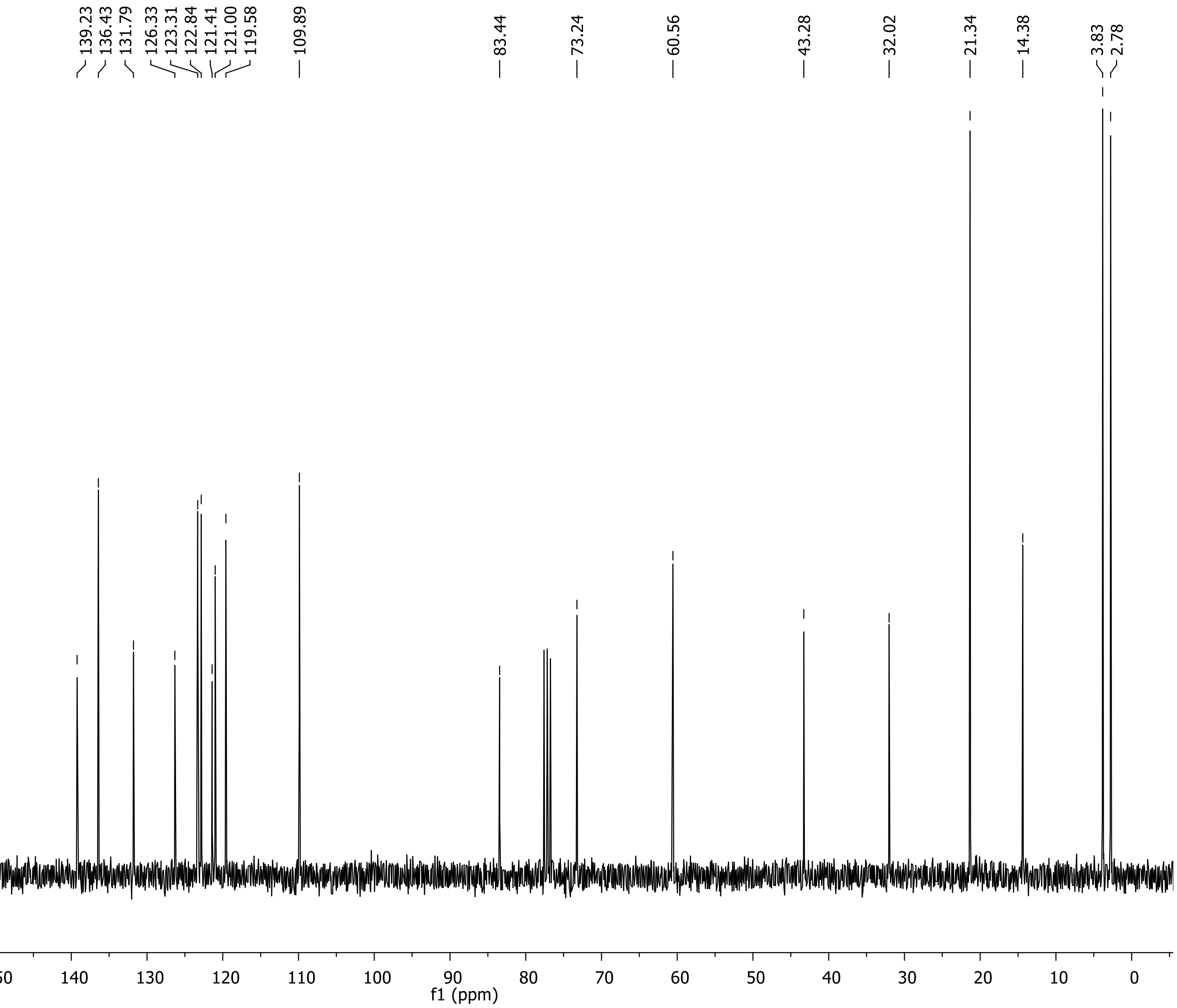

^{1}H NMR ($CDCl_3$, 300 MHz)

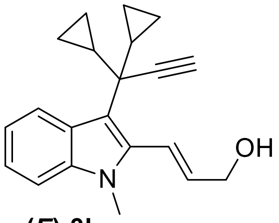

(*E*)-3l

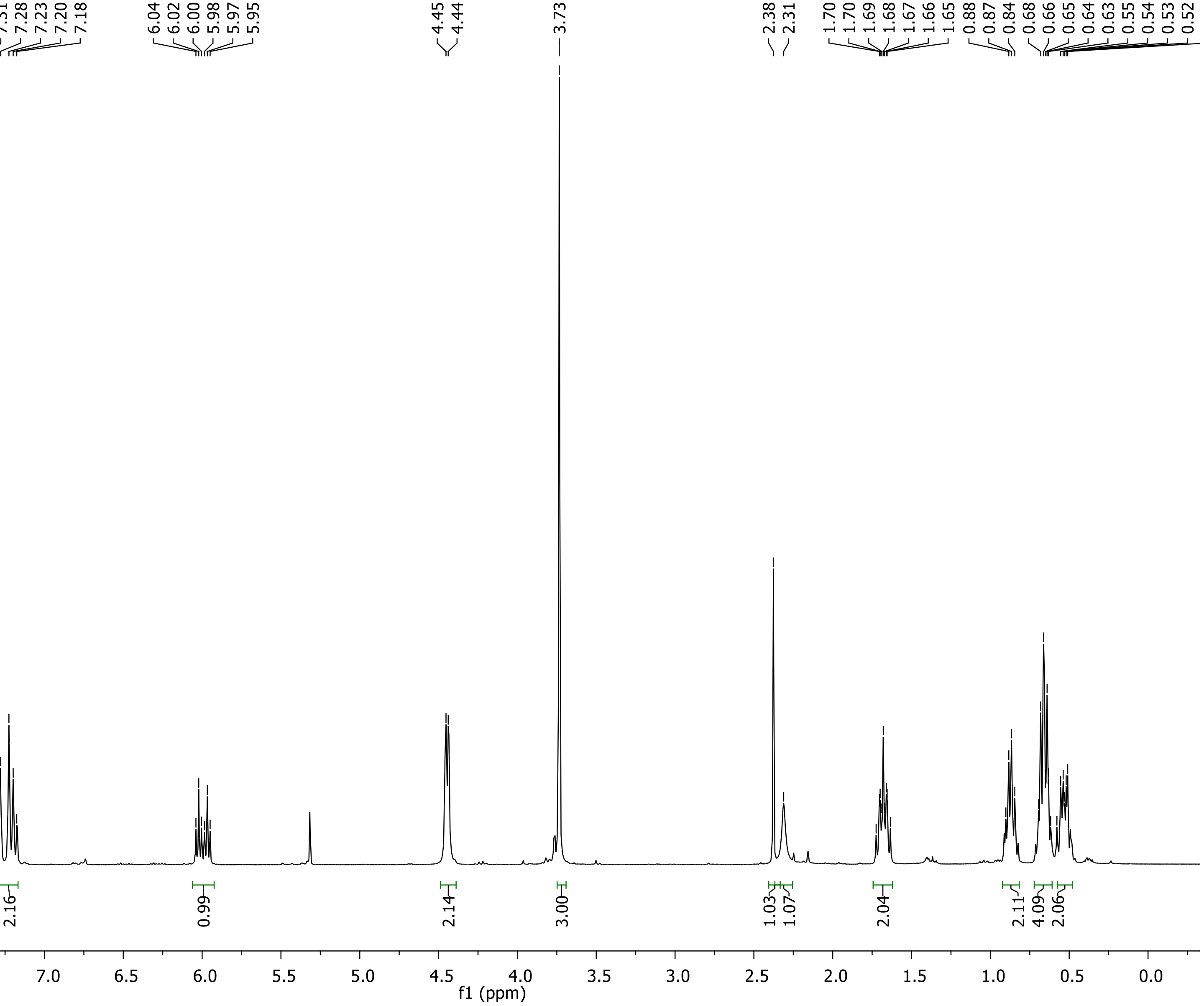

^{13}C NMR ($CDCl_3$, 75.4 MHz)

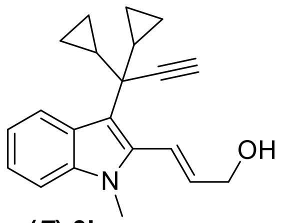

(*E*)-3l

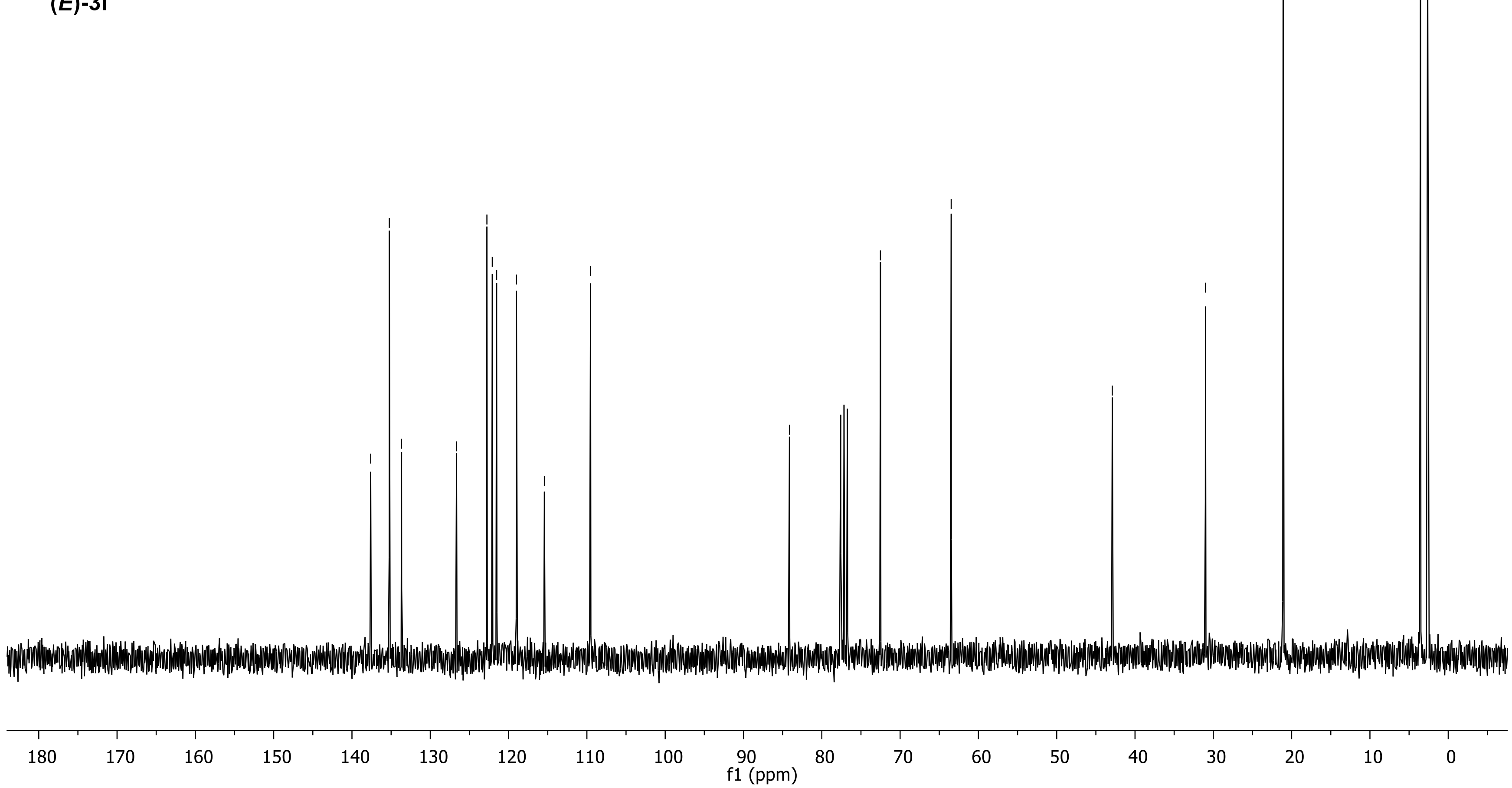

^{1}H NMR ($CDCl_3$, 300 MHz)

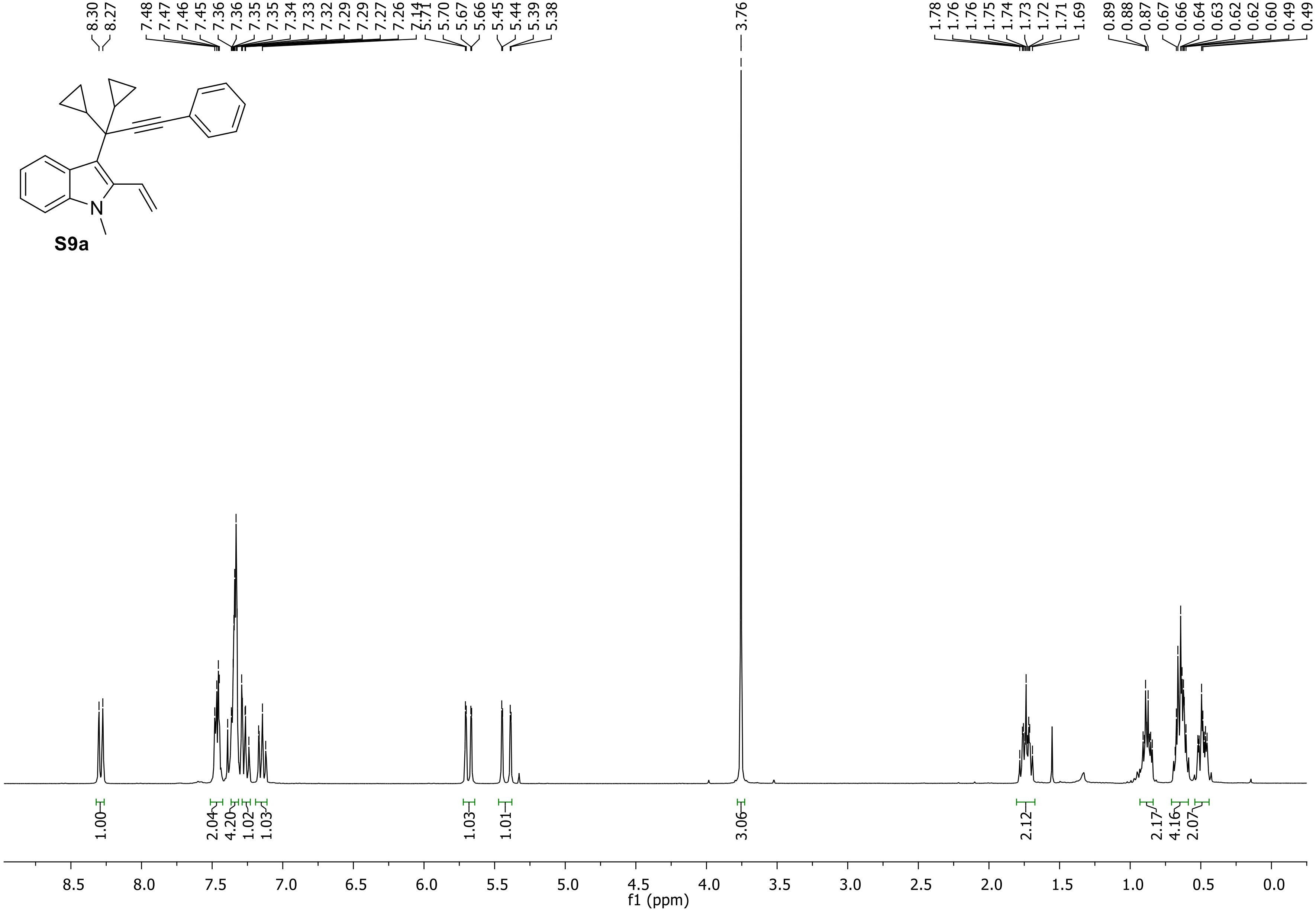

^{13}C NMR ($CDCl_3$, 75.4 MHz)

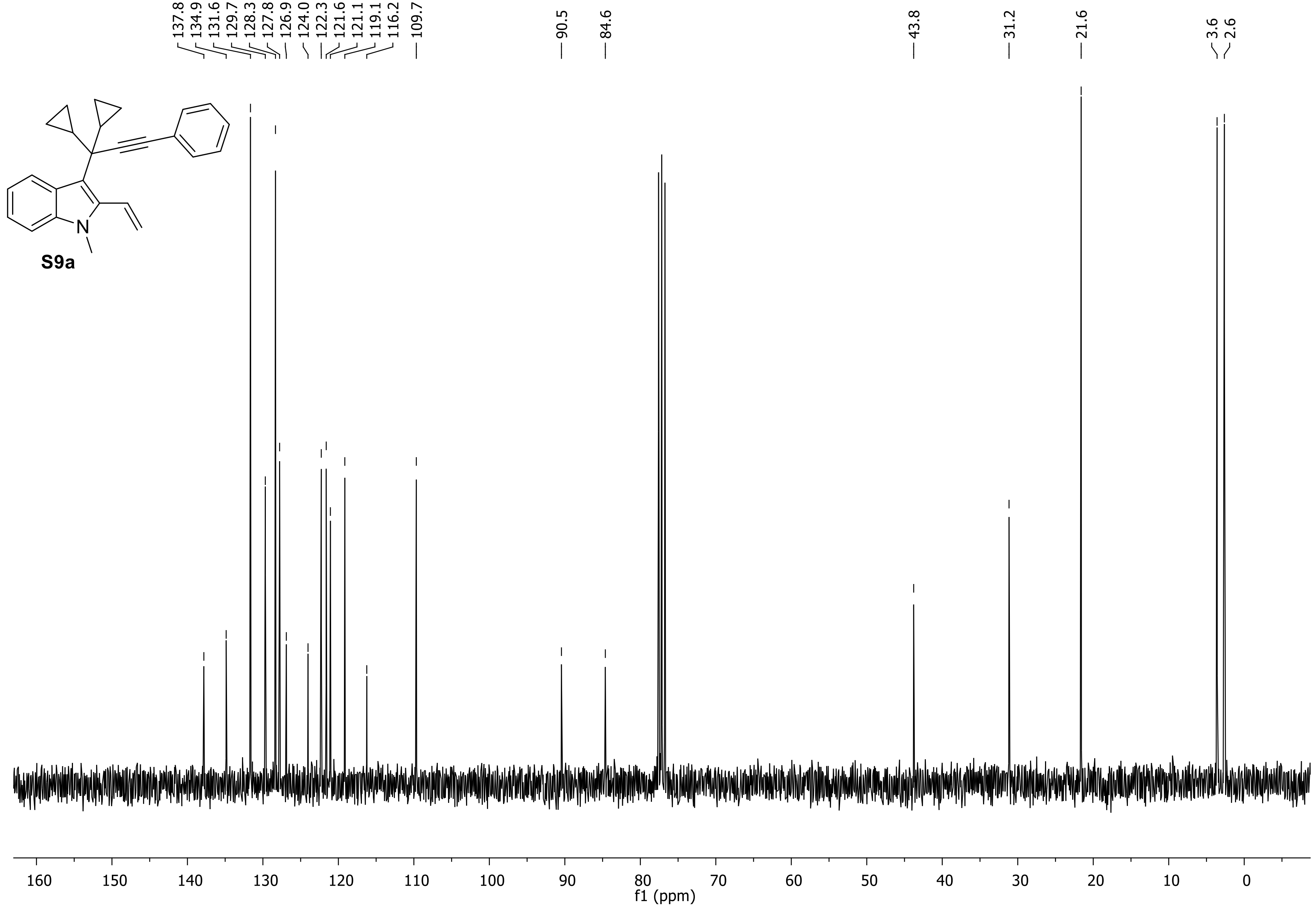

^{1}H NMR ($CDCl_3$, 300 MHz)

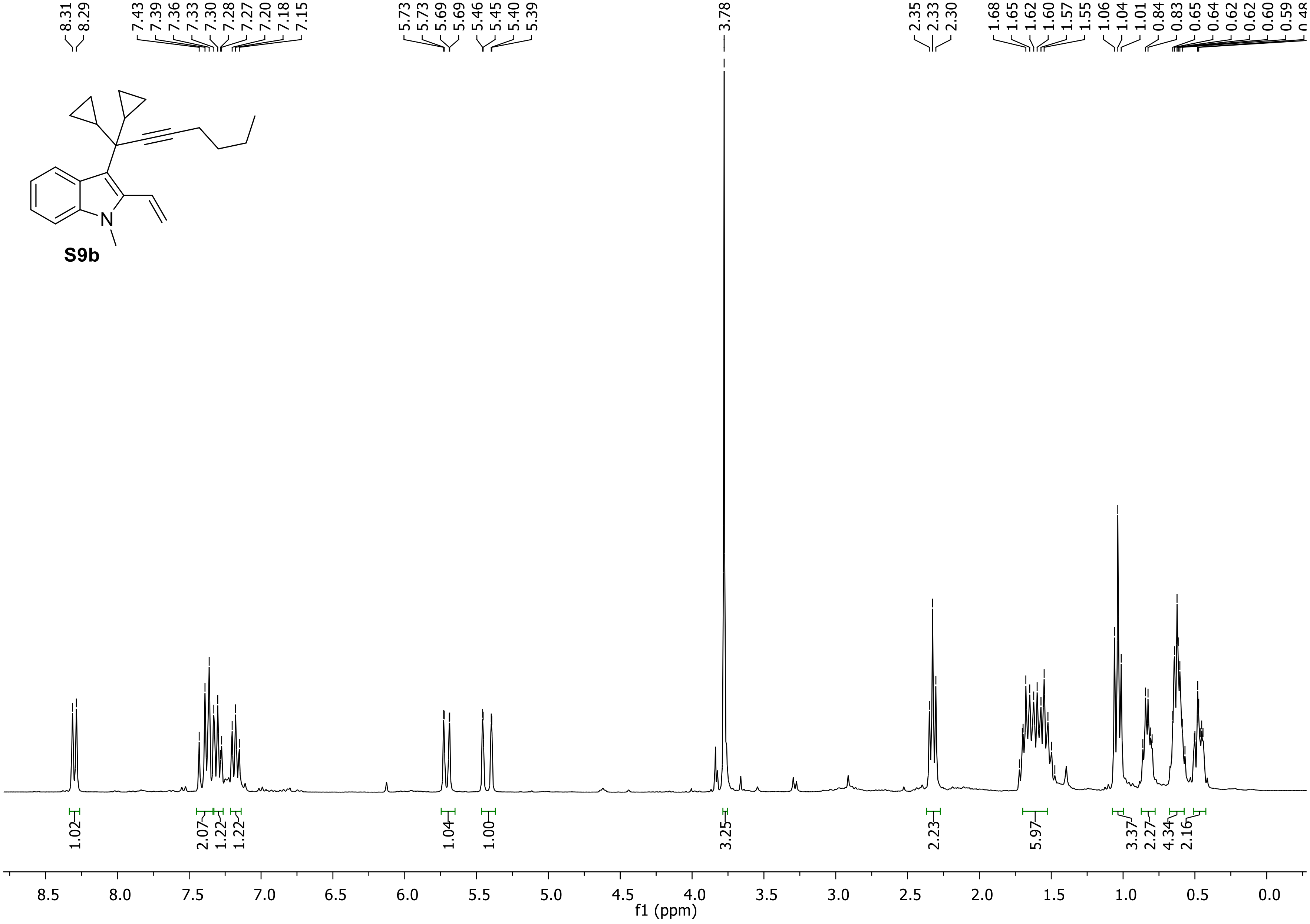

S9b

^{13}C NMR ($CDCl_3$, 75.4 MHz)

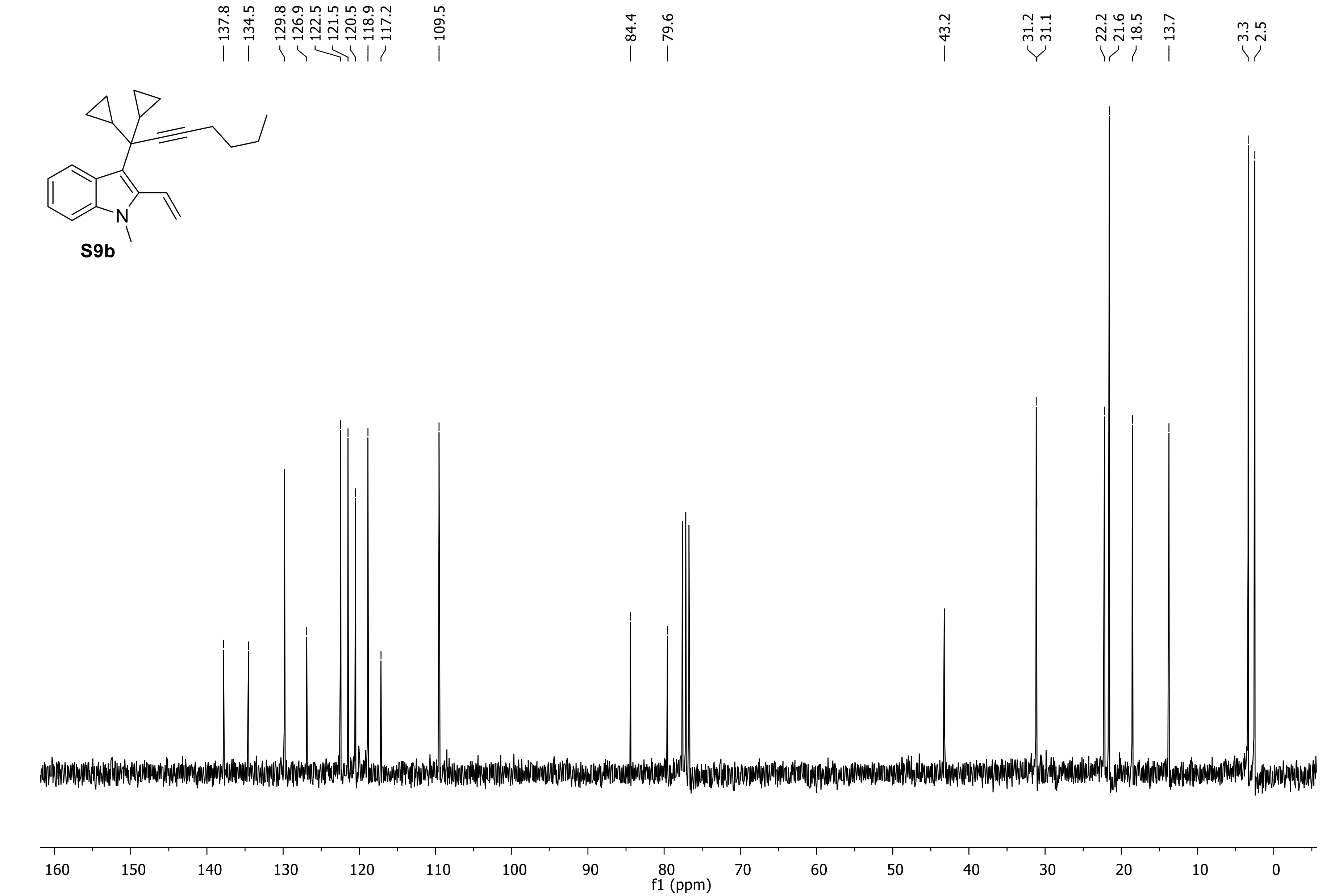

^{1}H NMR ($CDCl_3$, 300 MHz)

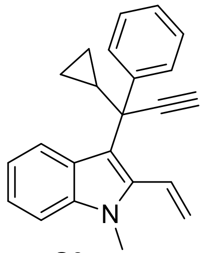

S9c

*Unidentified impurity

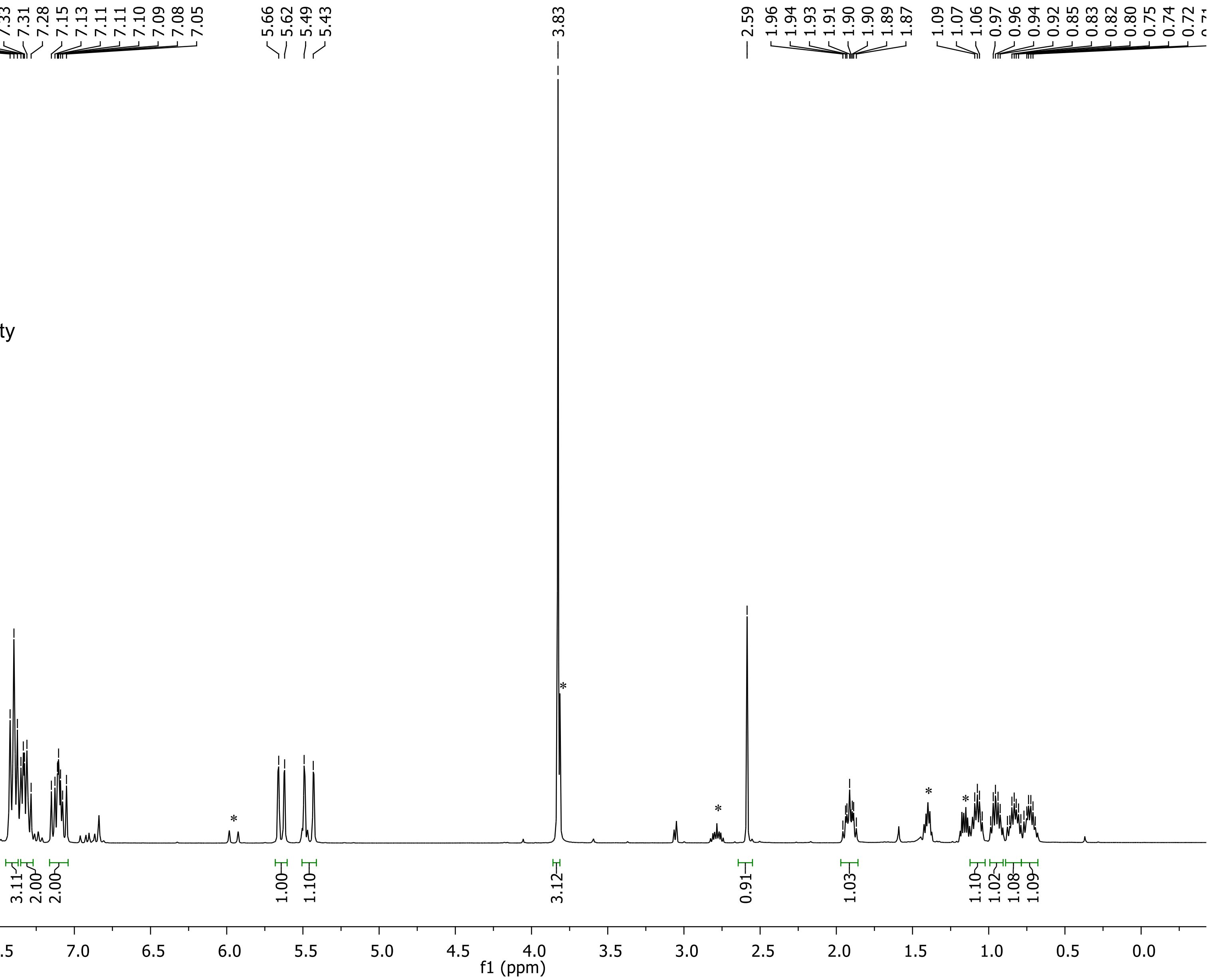

^{13}C NMR ($CDCl_3$, 75.4 MHz)

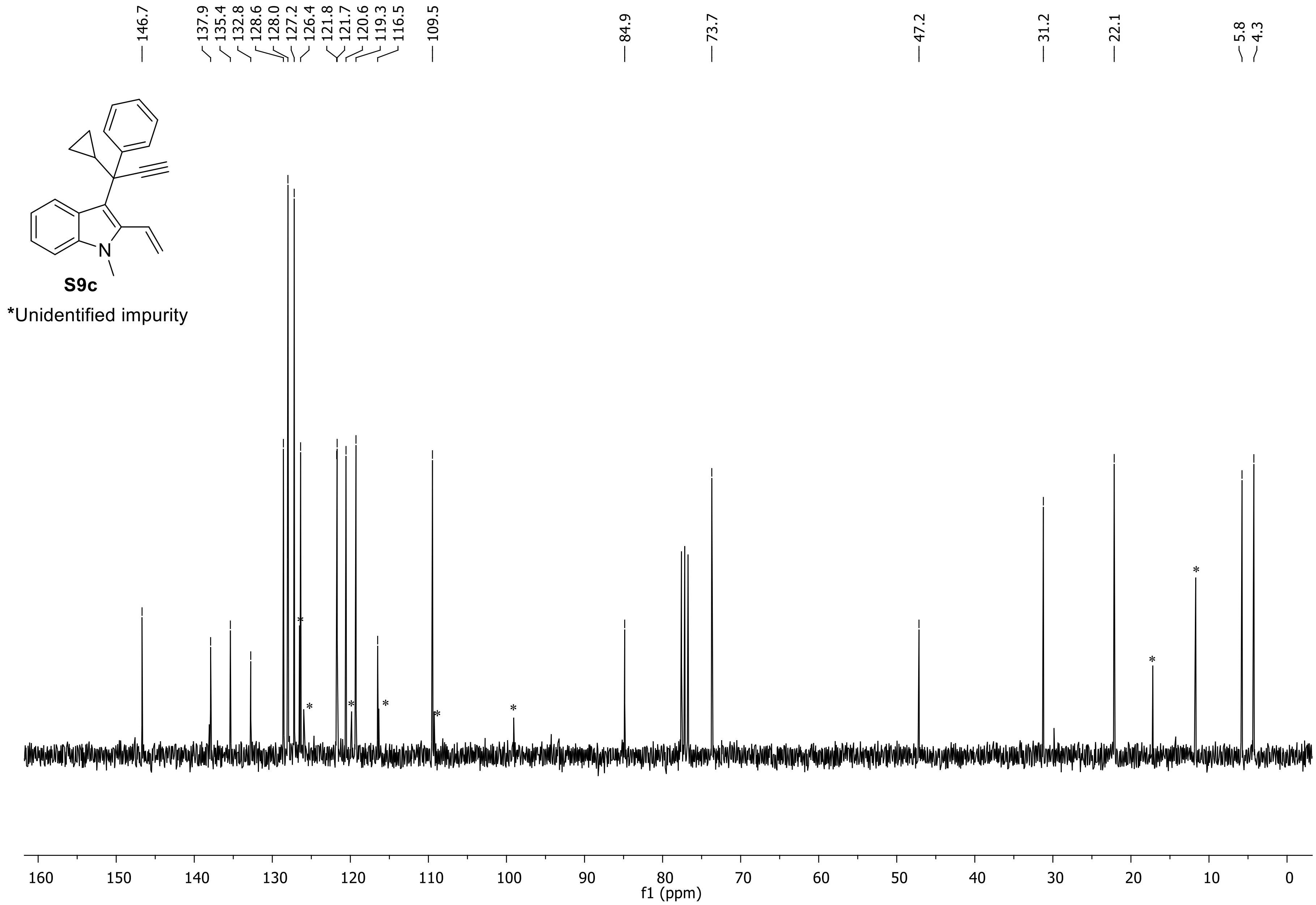

^{1}H NMR ($CDCl_3$, 300 MHz)

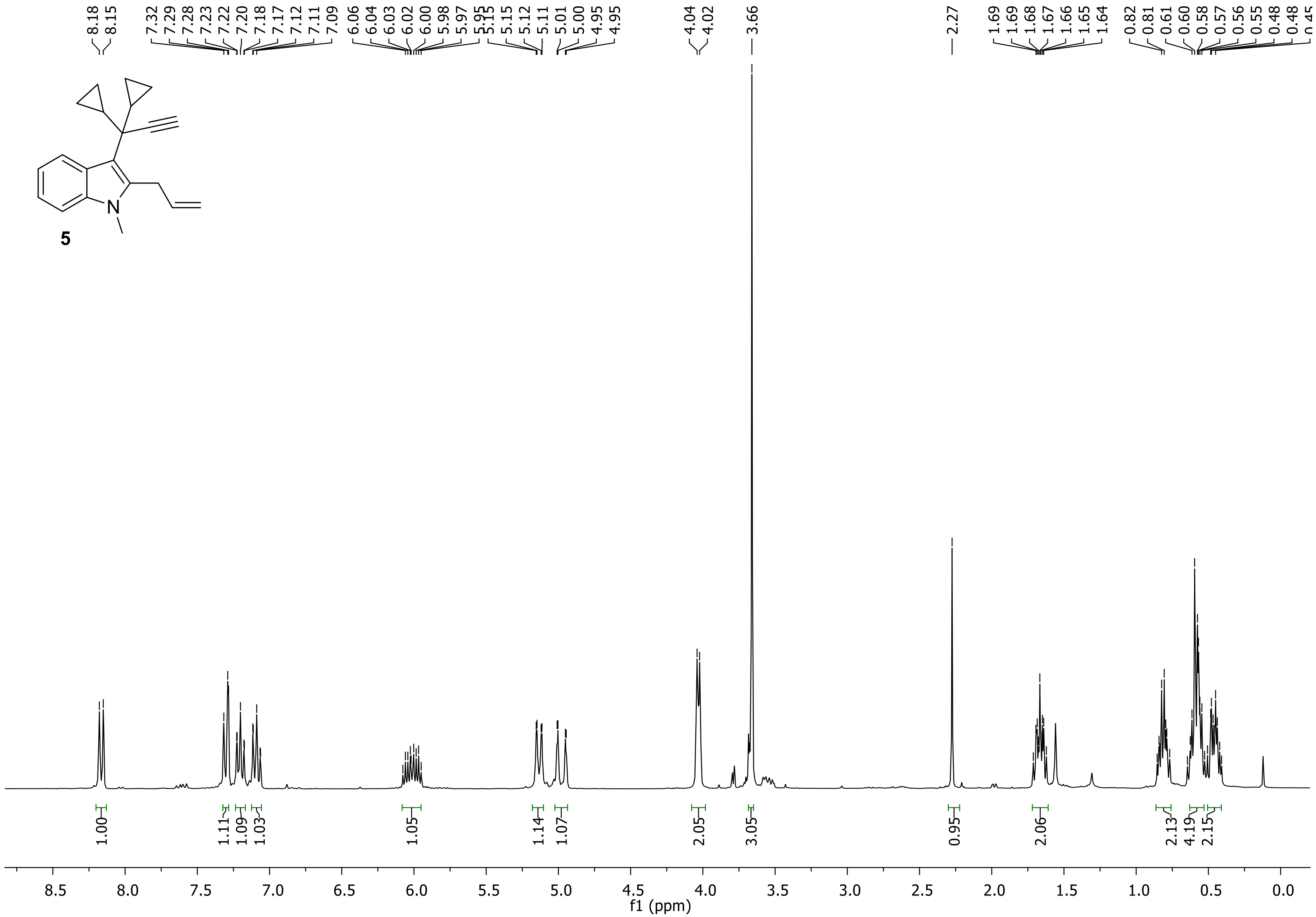

^{13}C NMR ($CDCl_3$, 75.4 MHz)

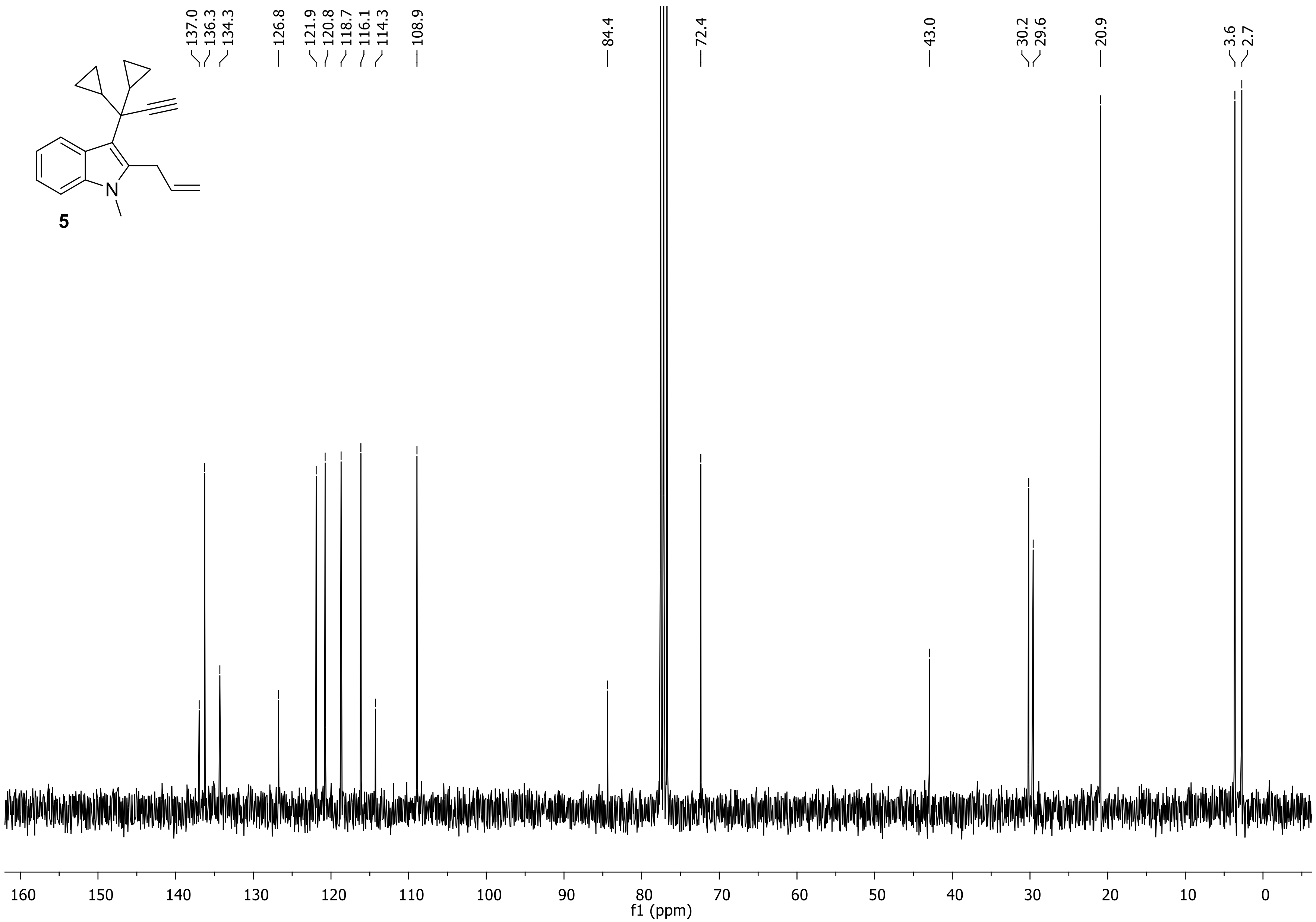

^{1}H NMR ($CDCl_3$, 300 MHz)

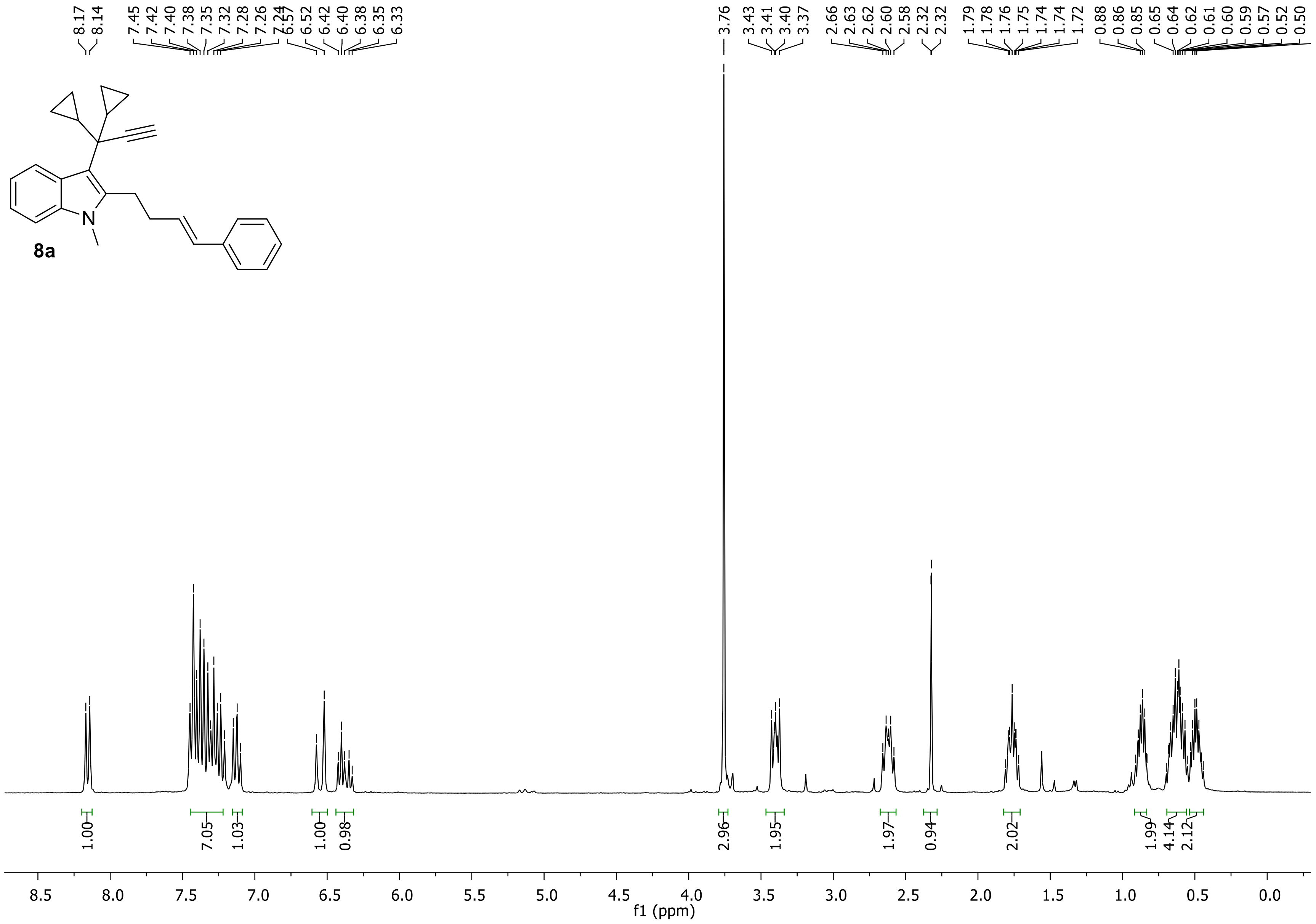

^{13}C NMR ($CDCl_3$, 75.4 MHz)

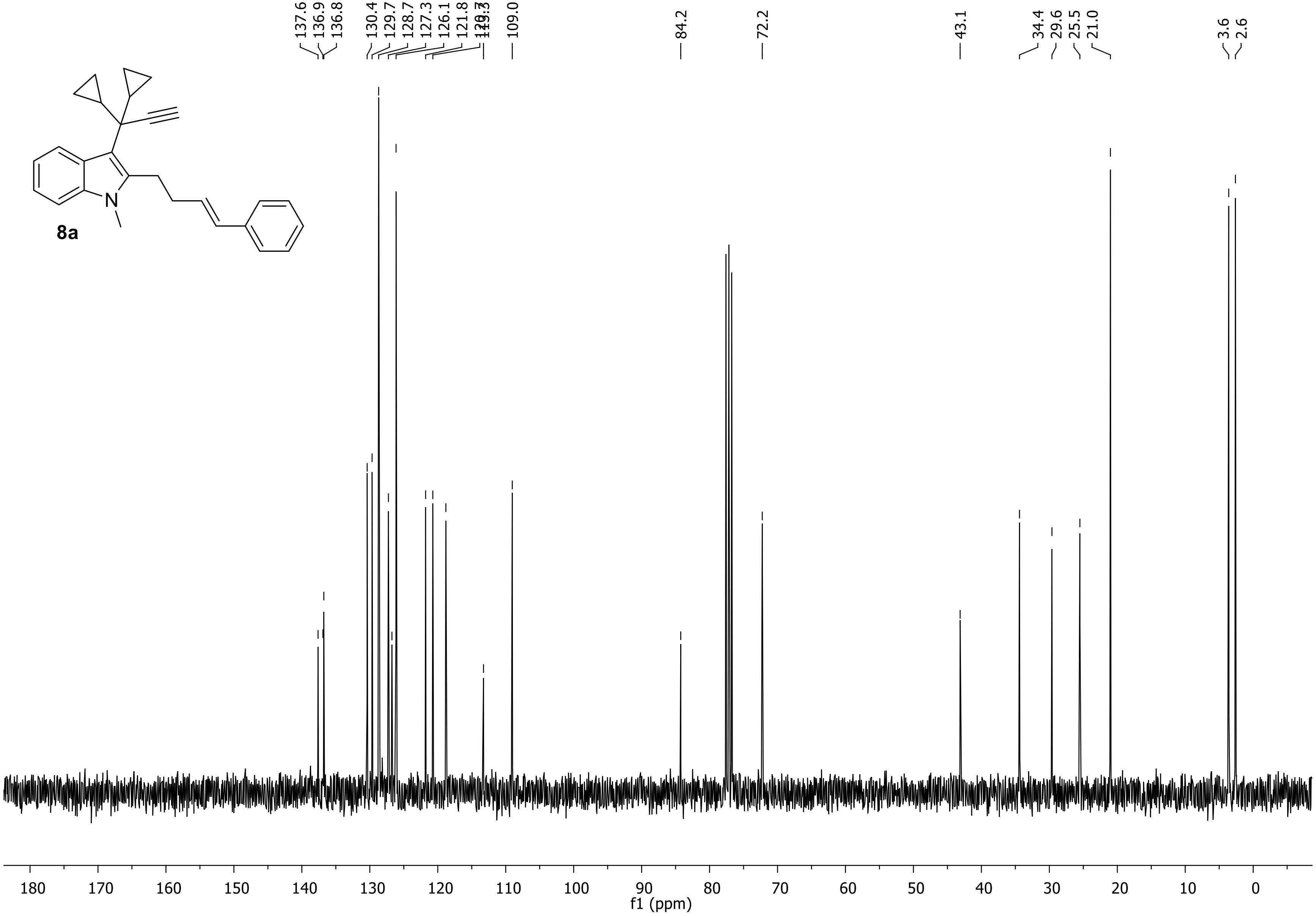

^{1}H NMR (CDCl$_3$, 300 MHz)

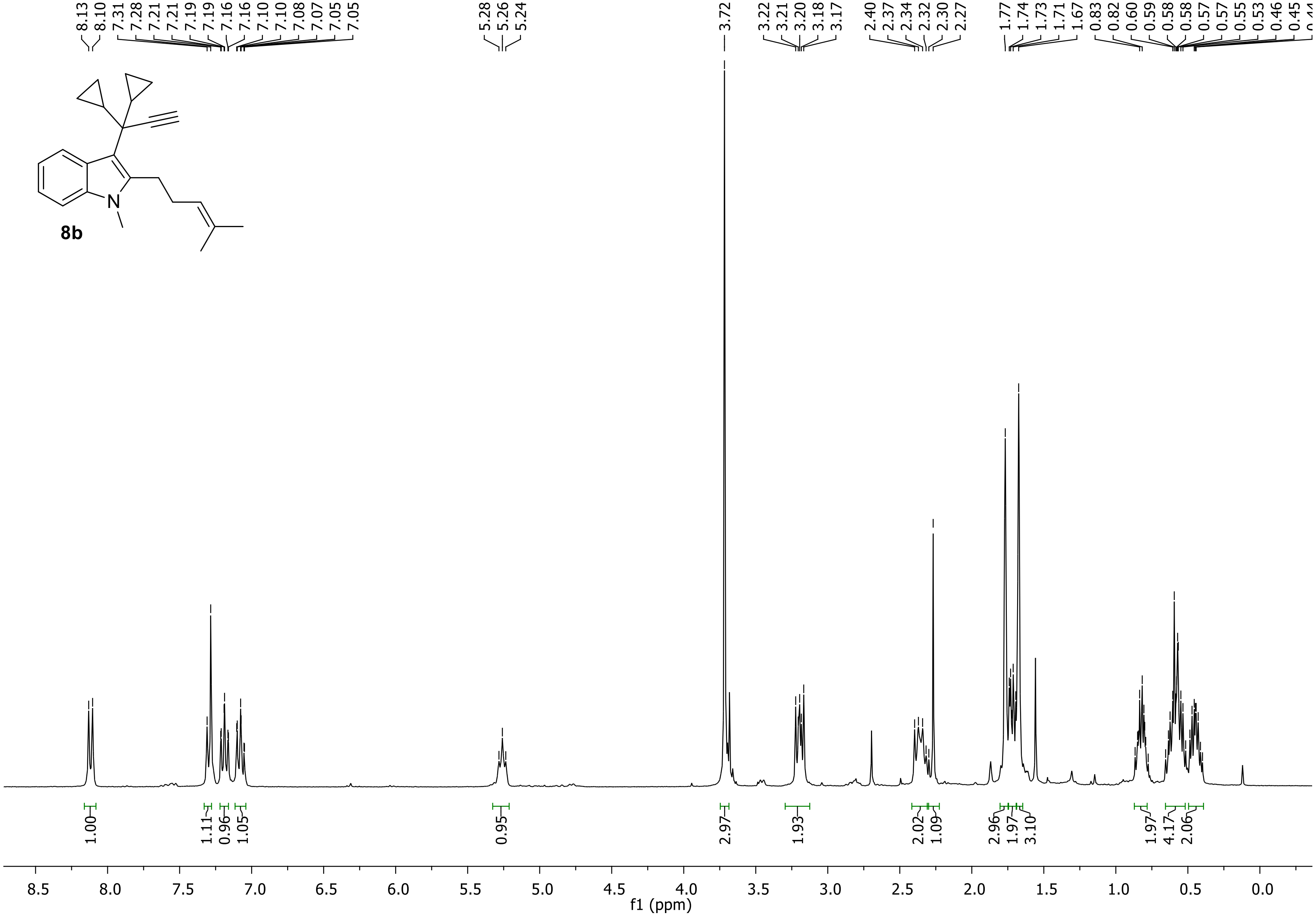

^{13}C NMR ($CDCl_3$, 75.4 MHz)

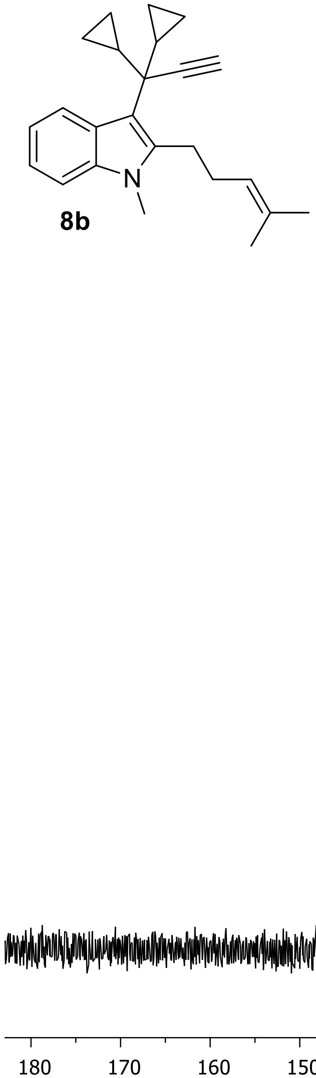

8b

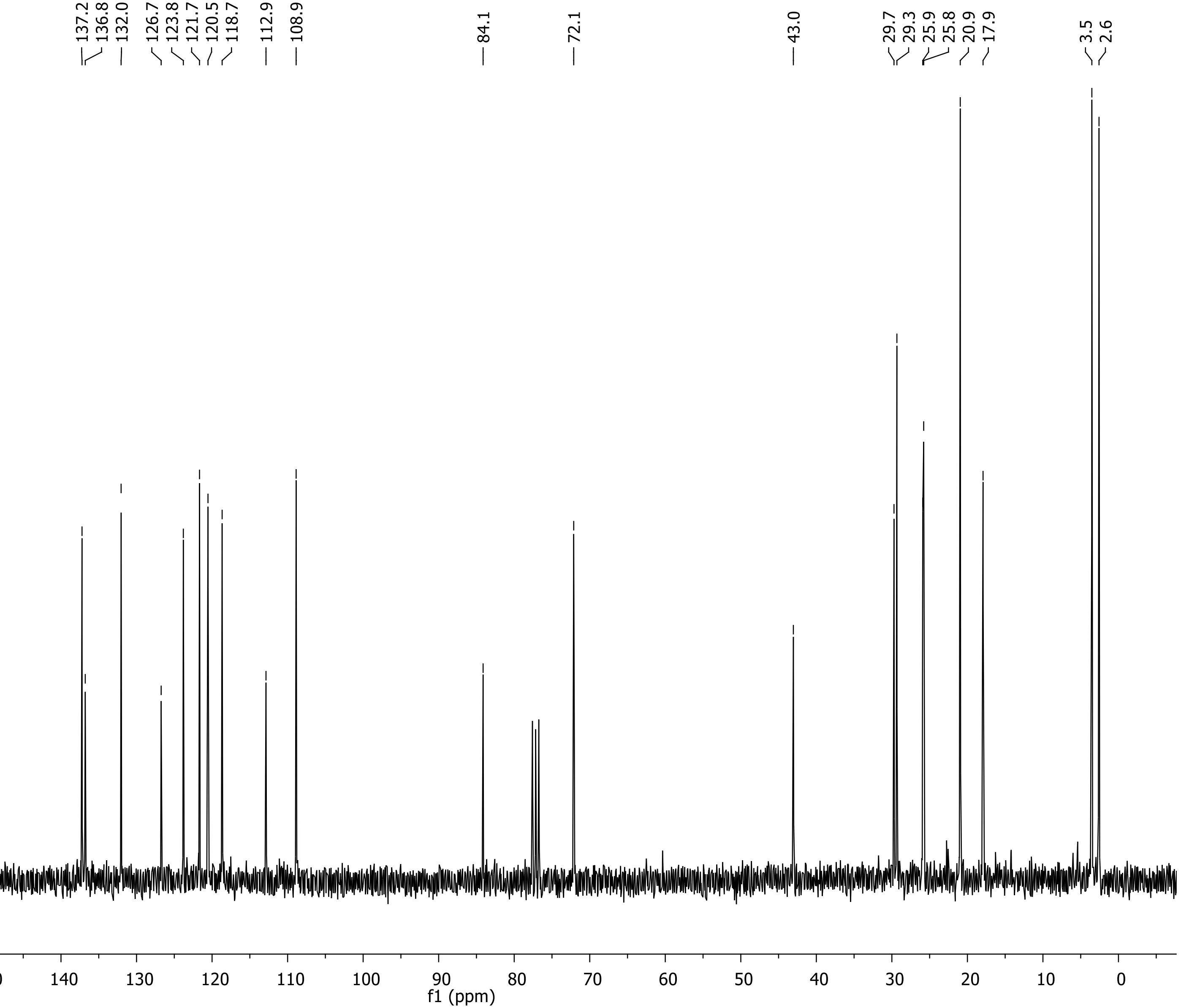

^{1}H NMR ($CDCl_3$, 300 MHz)

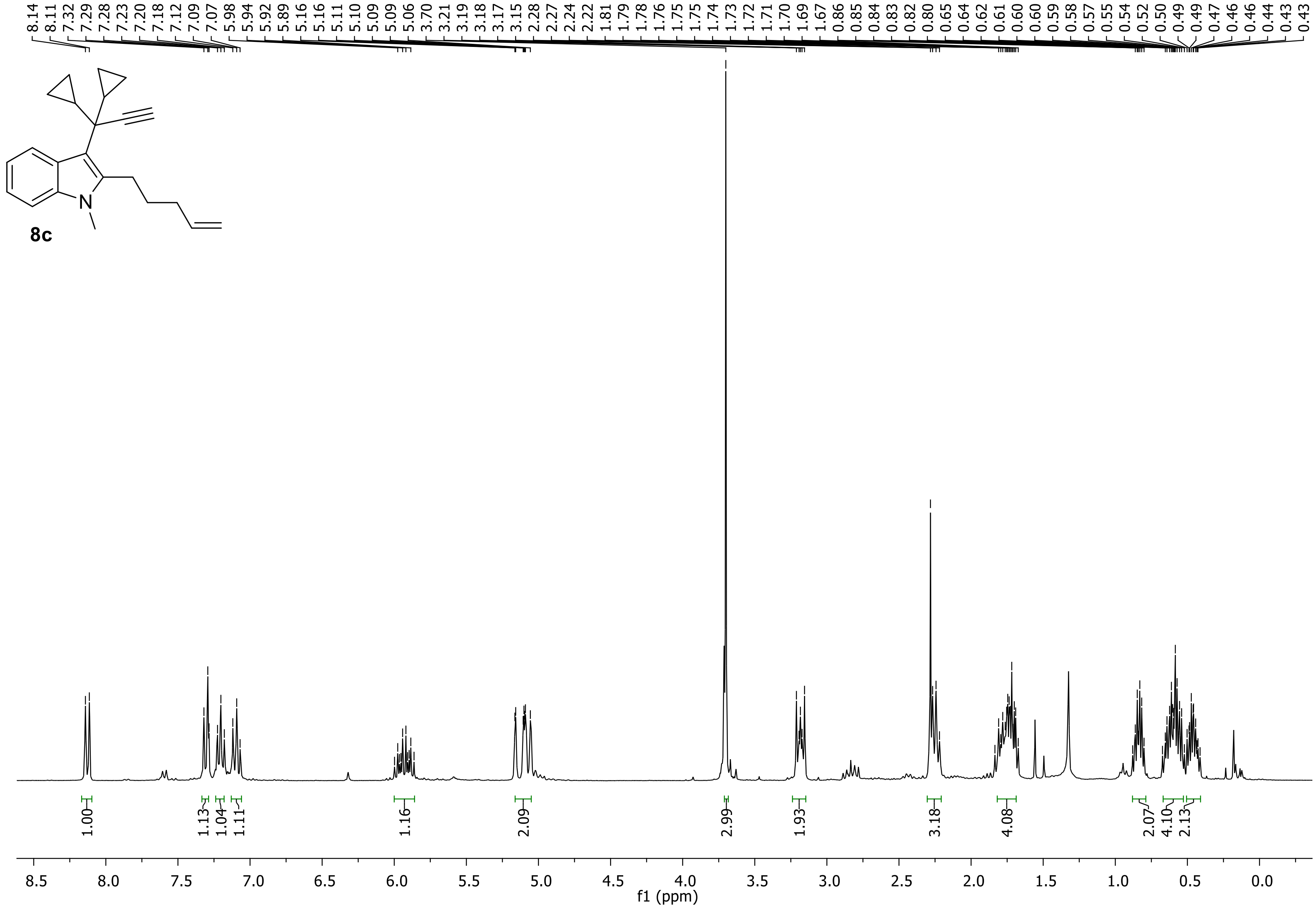

^{13}C NMR ($CDCl_3$, 75.4 MHz)

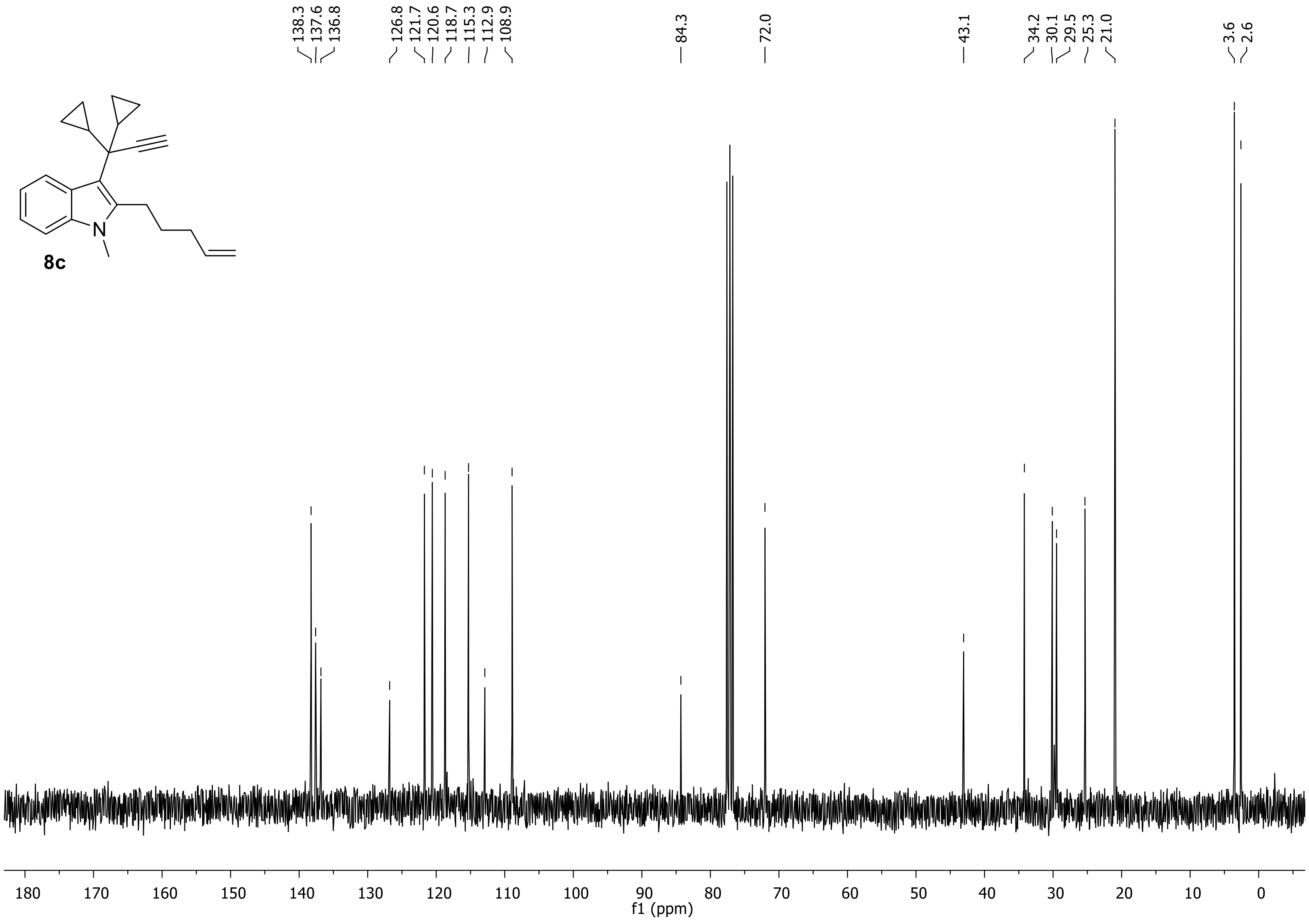

^1H NMR ($CDCl_3$, 300 MHz)

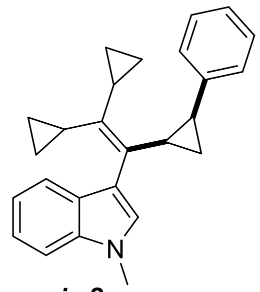

***cis*-2a**
d.r. 16/1

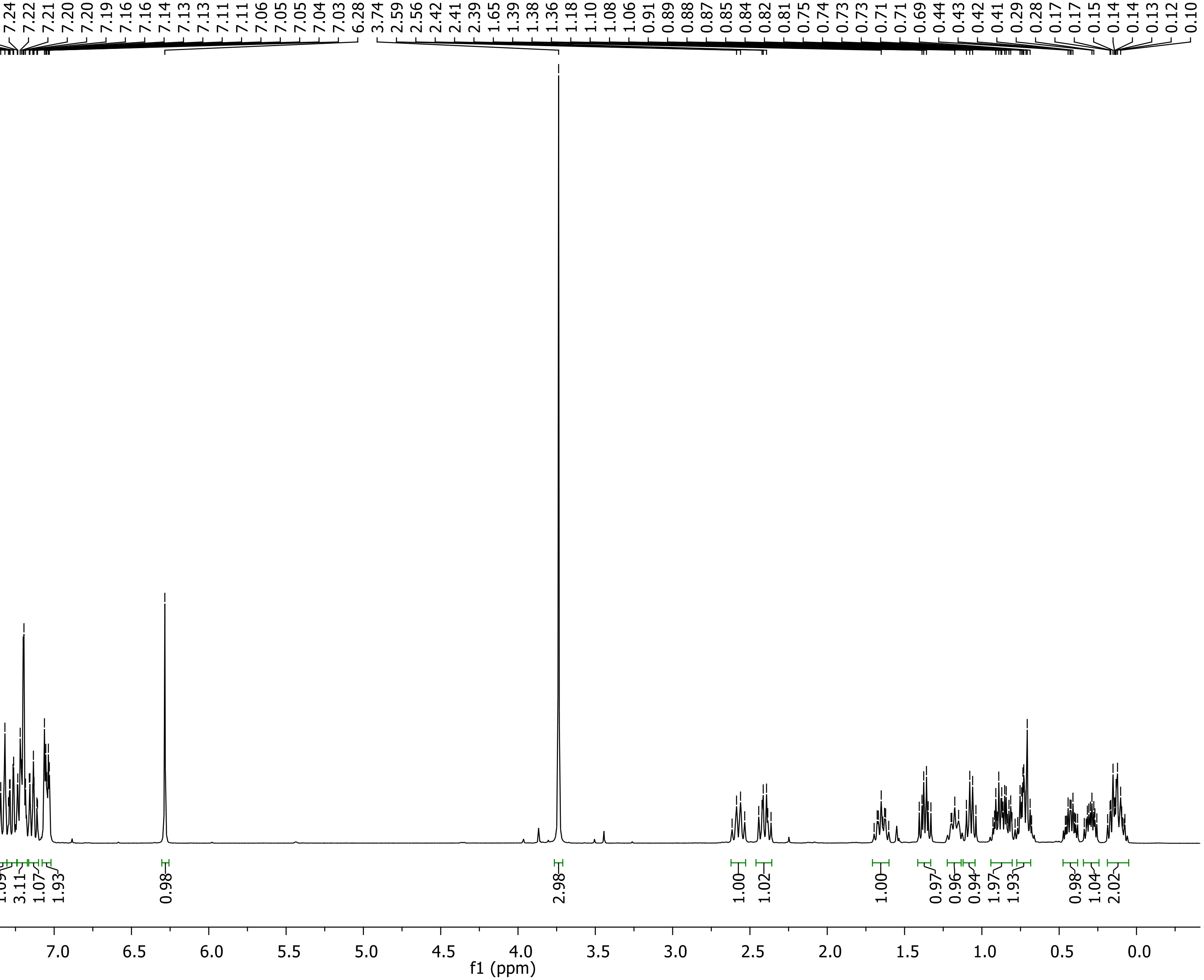

^{13}C NMR ($CDCl_3$, 75.4 MHz)

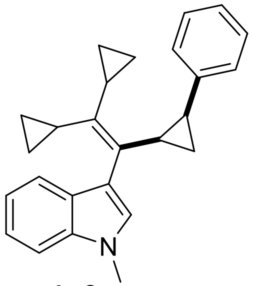

***cis*-2a**
d.r. 16/1

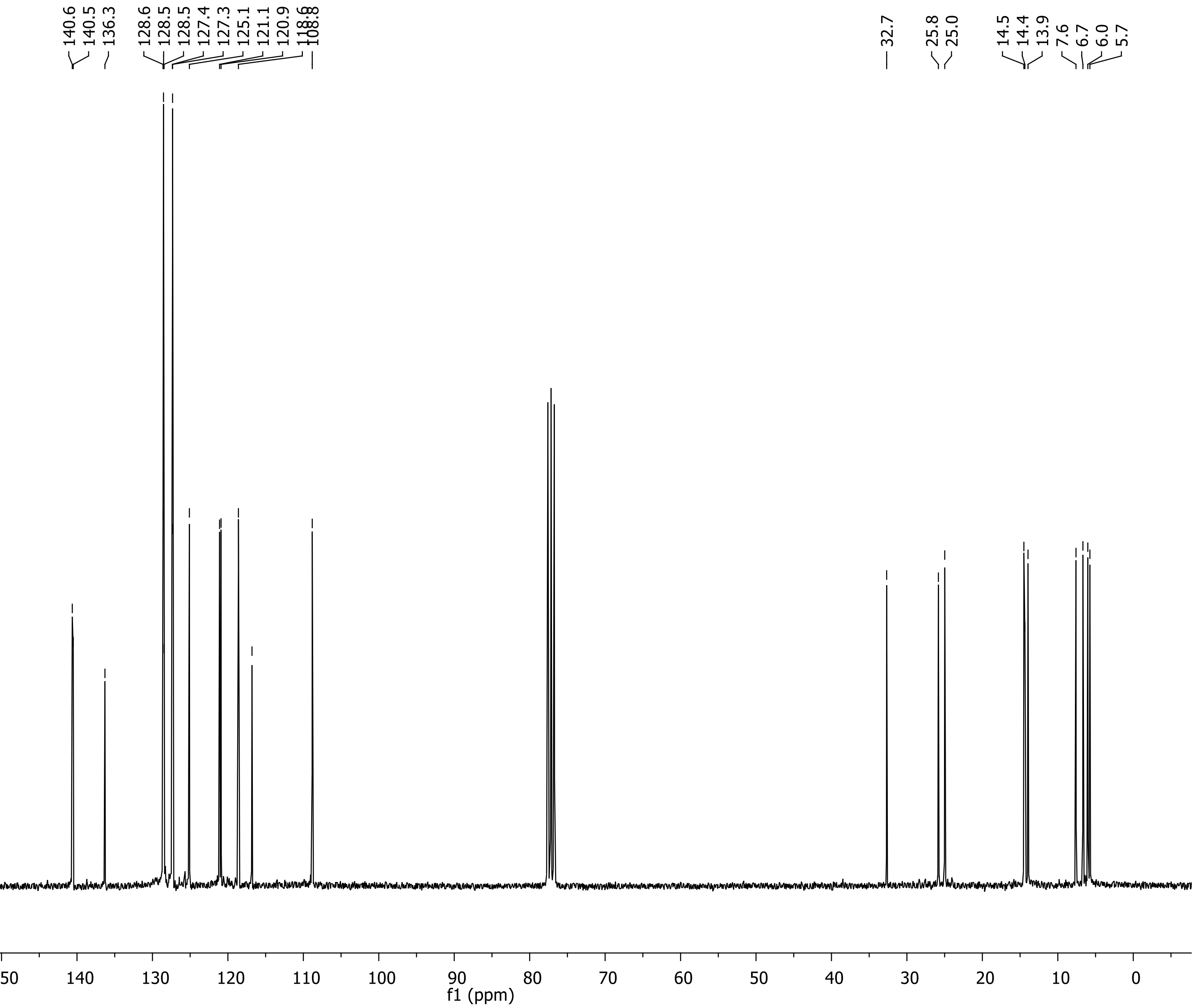

NOESY ($CDCl_3$, 500 MHz)

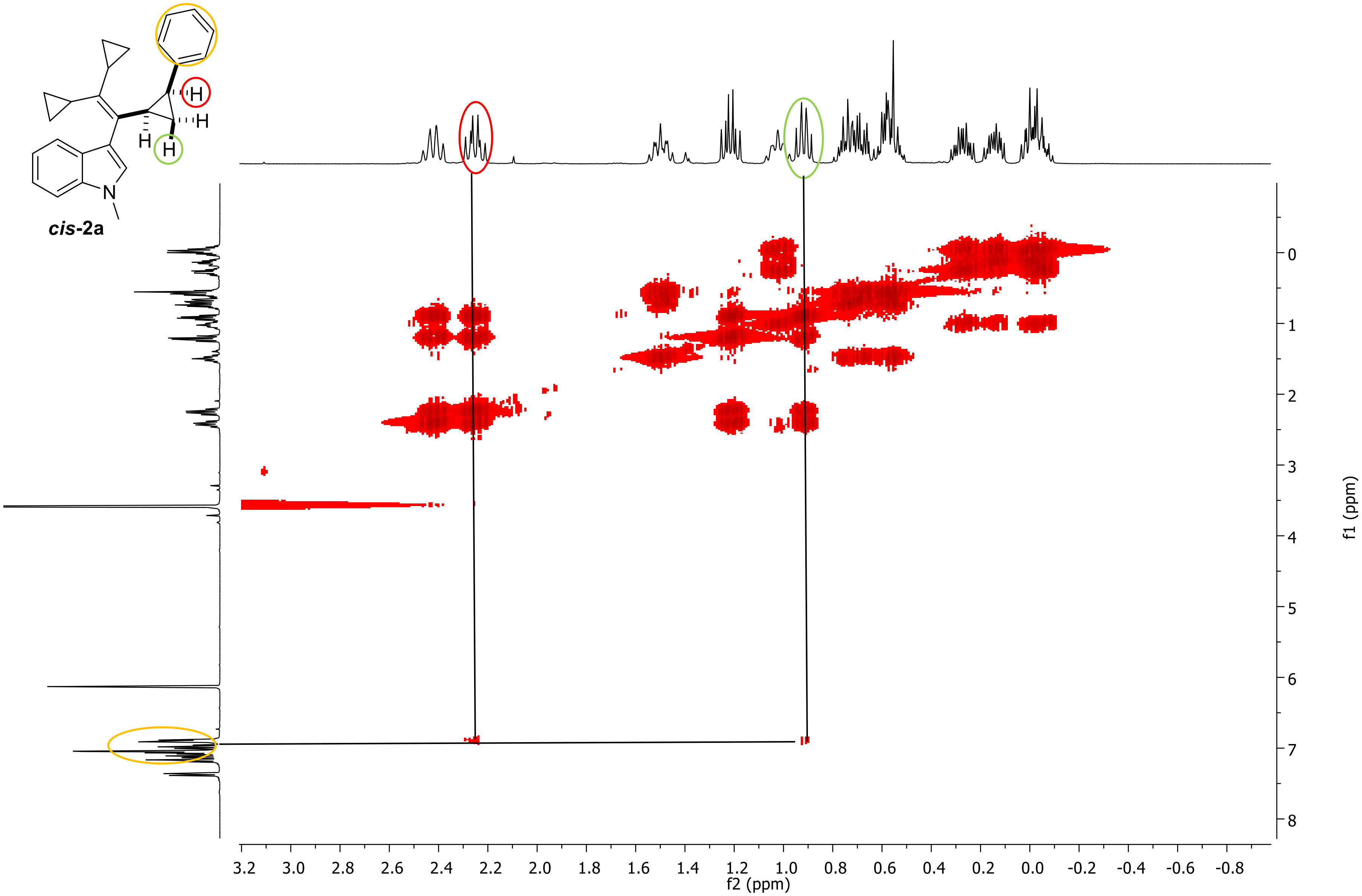

^1H NMR ($CDCl_3$, 500 MHz)

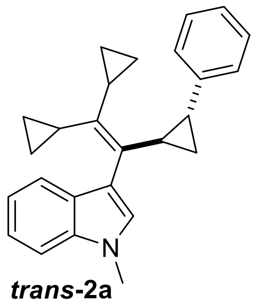

***trans*-2a**
d.r. 15/1

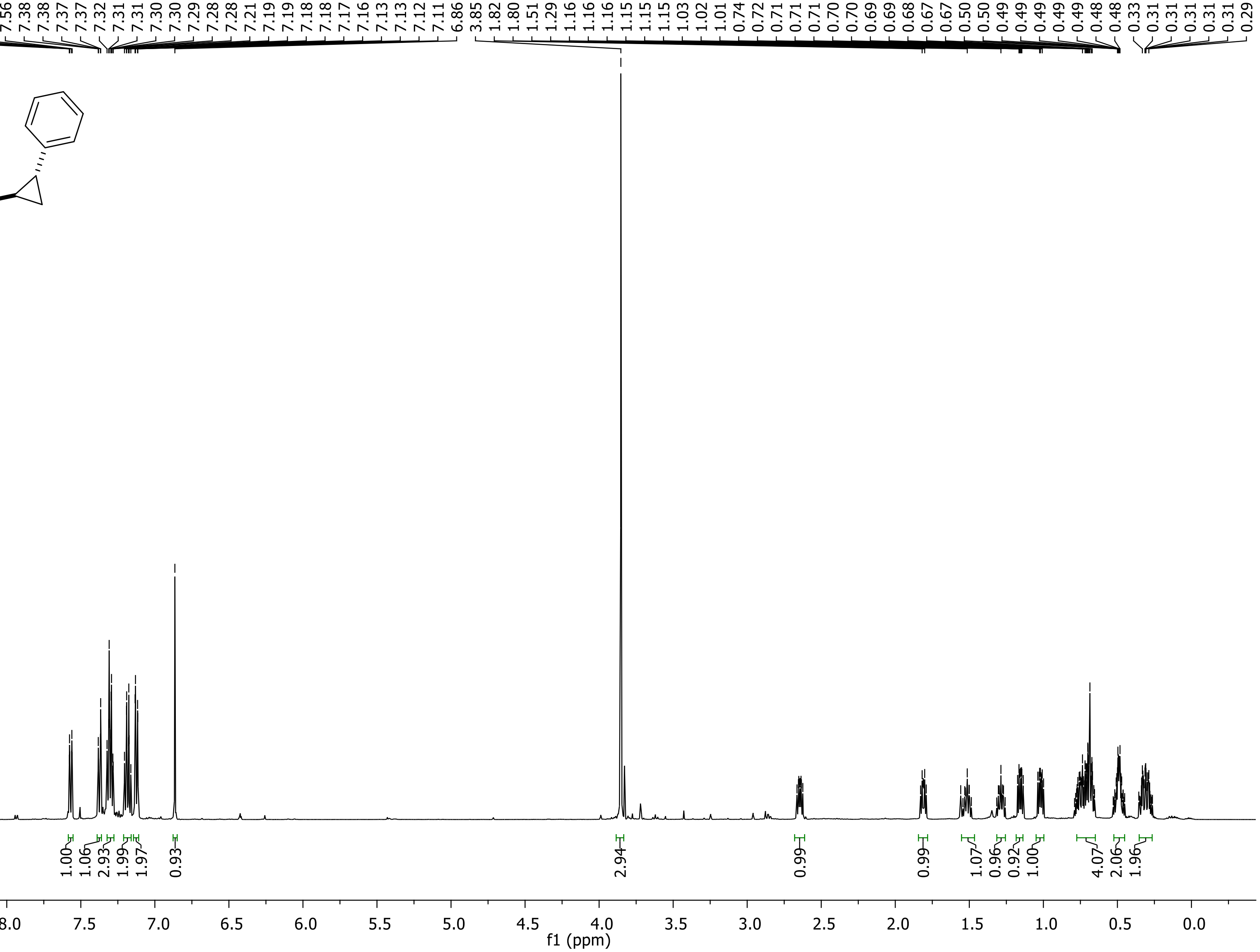

^{13}C NMR ($CDCl_3$, 75.4 MHz)

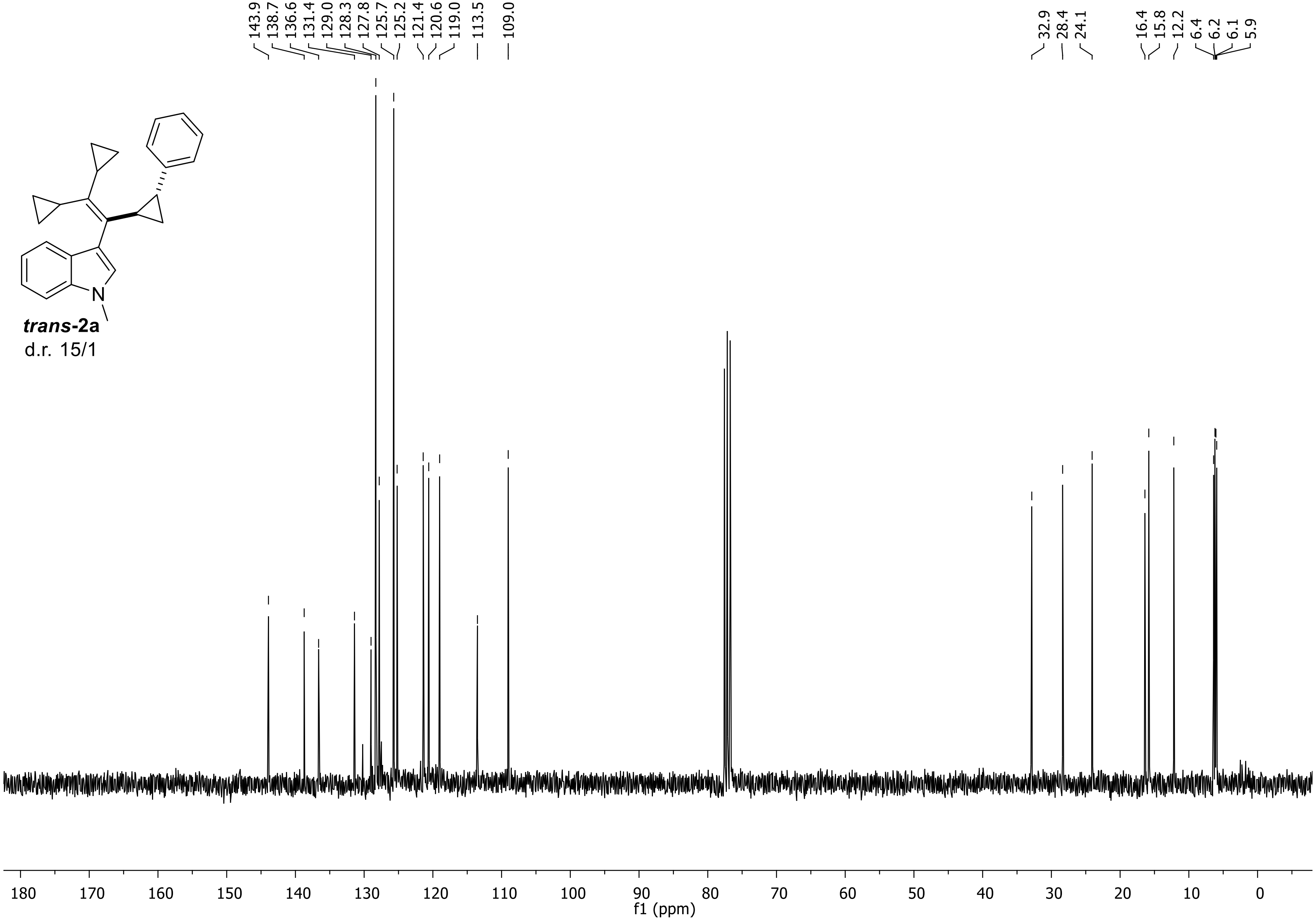

NOESY ($CDCl_3$, 500 MHz)

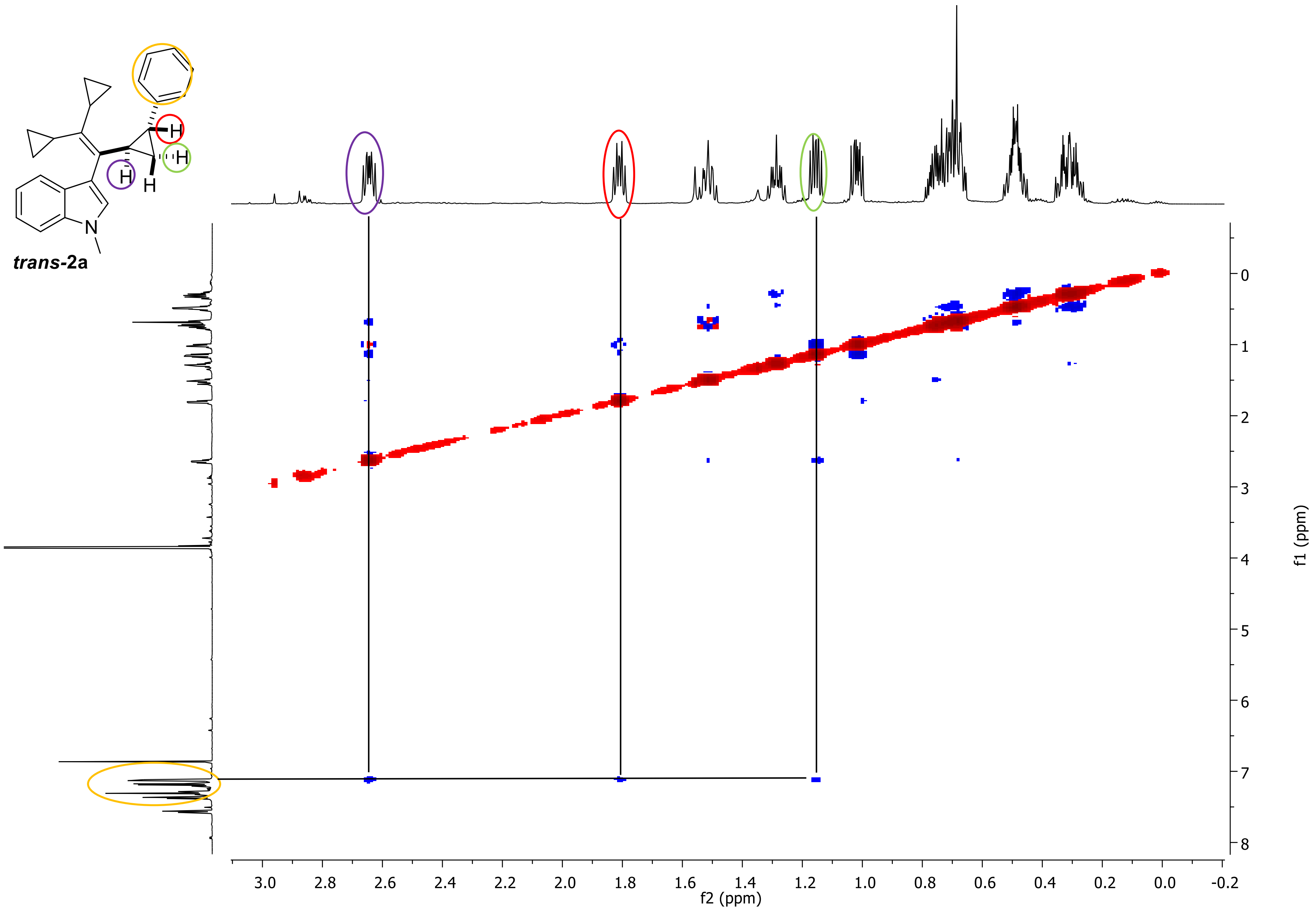

^{1}H NMR ($CDCl_3$, 300 MHz)

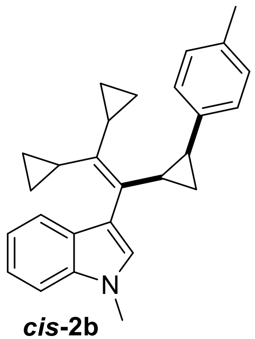

***cis*-2b**

d.r. 10/1

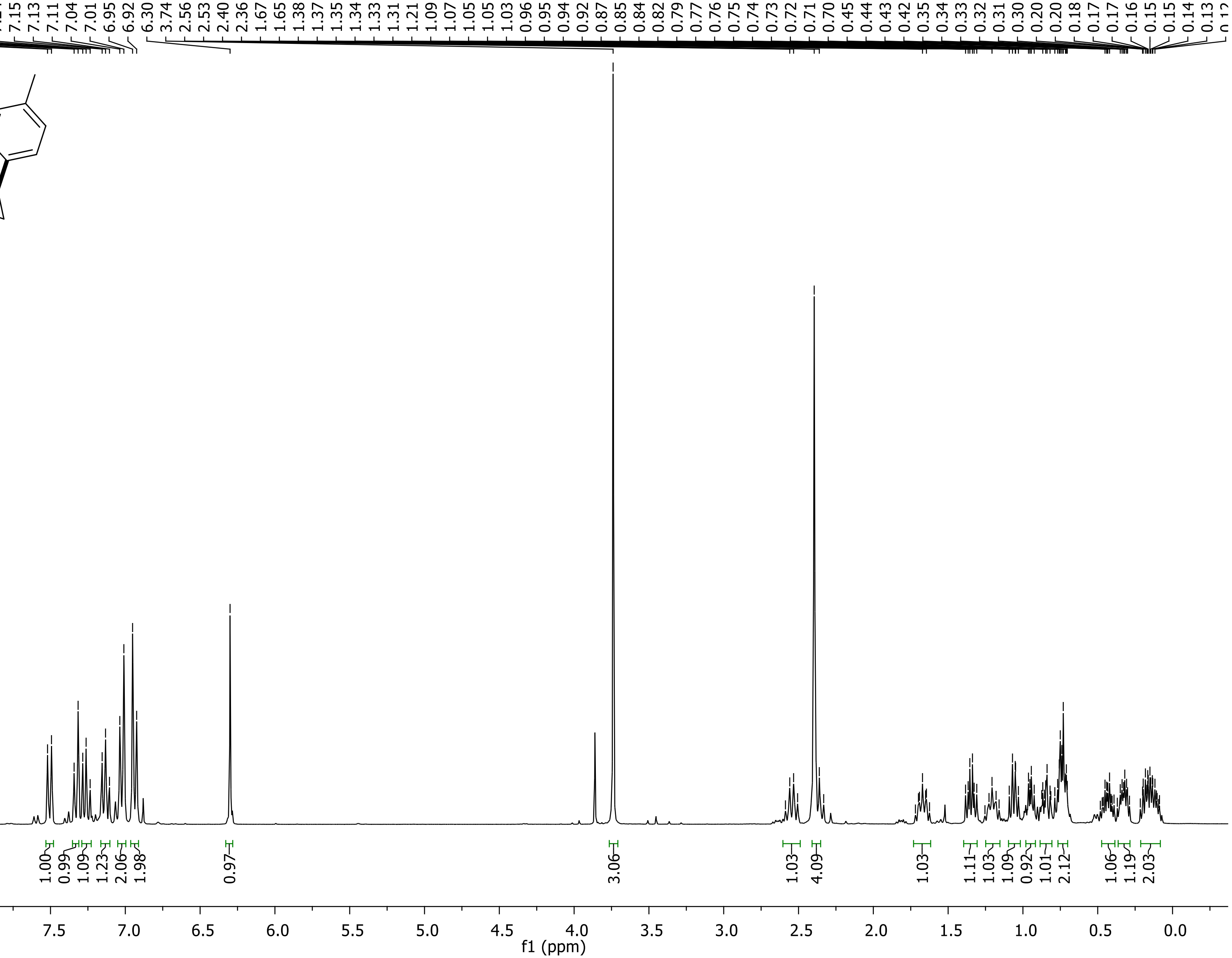

^{13}C NMR ($CDCl_3$, 75.4 MHz)

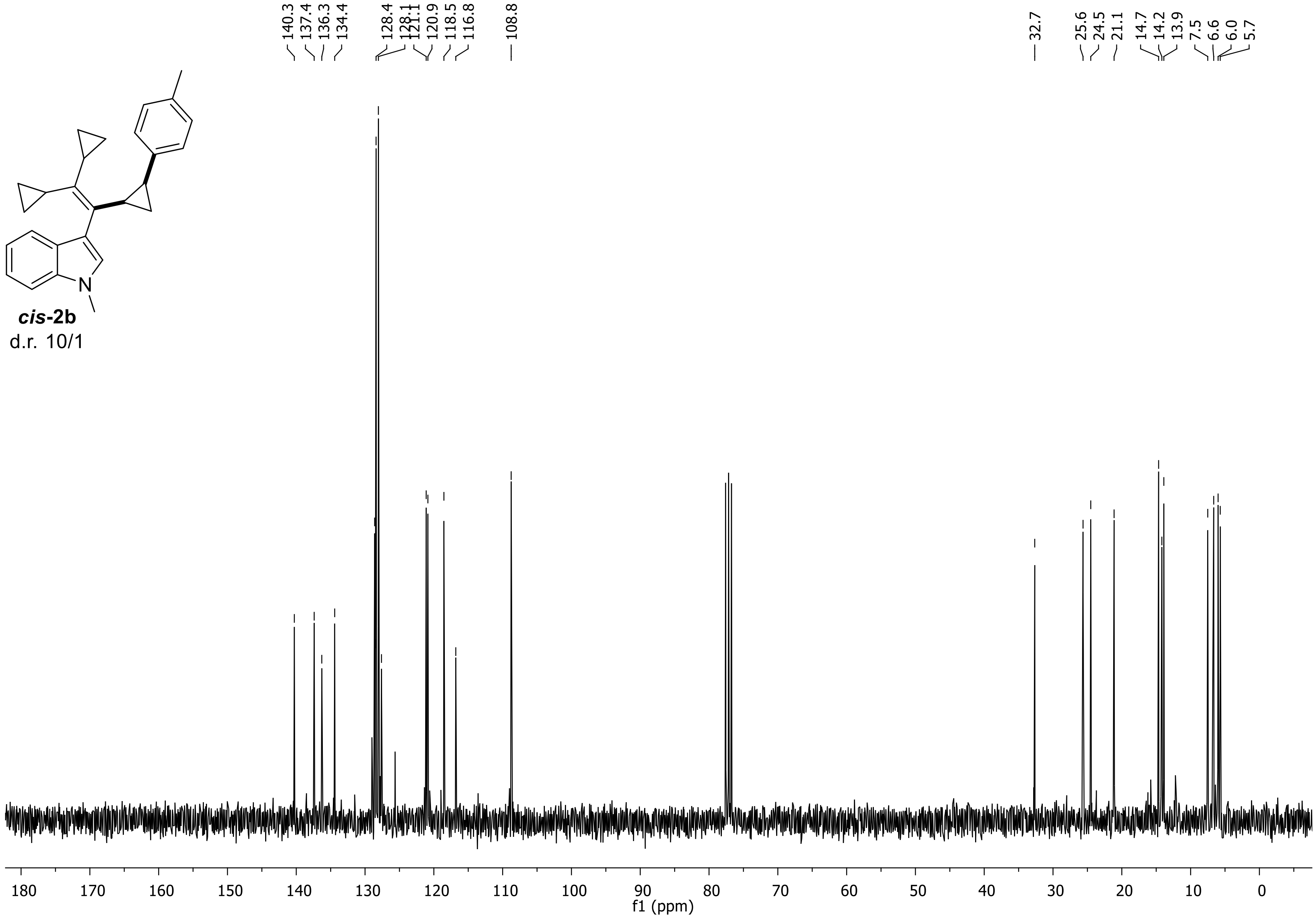

***cis*-2b**

d.r. 10/1

NOESY ($CDCl_3$, 500 MHz)

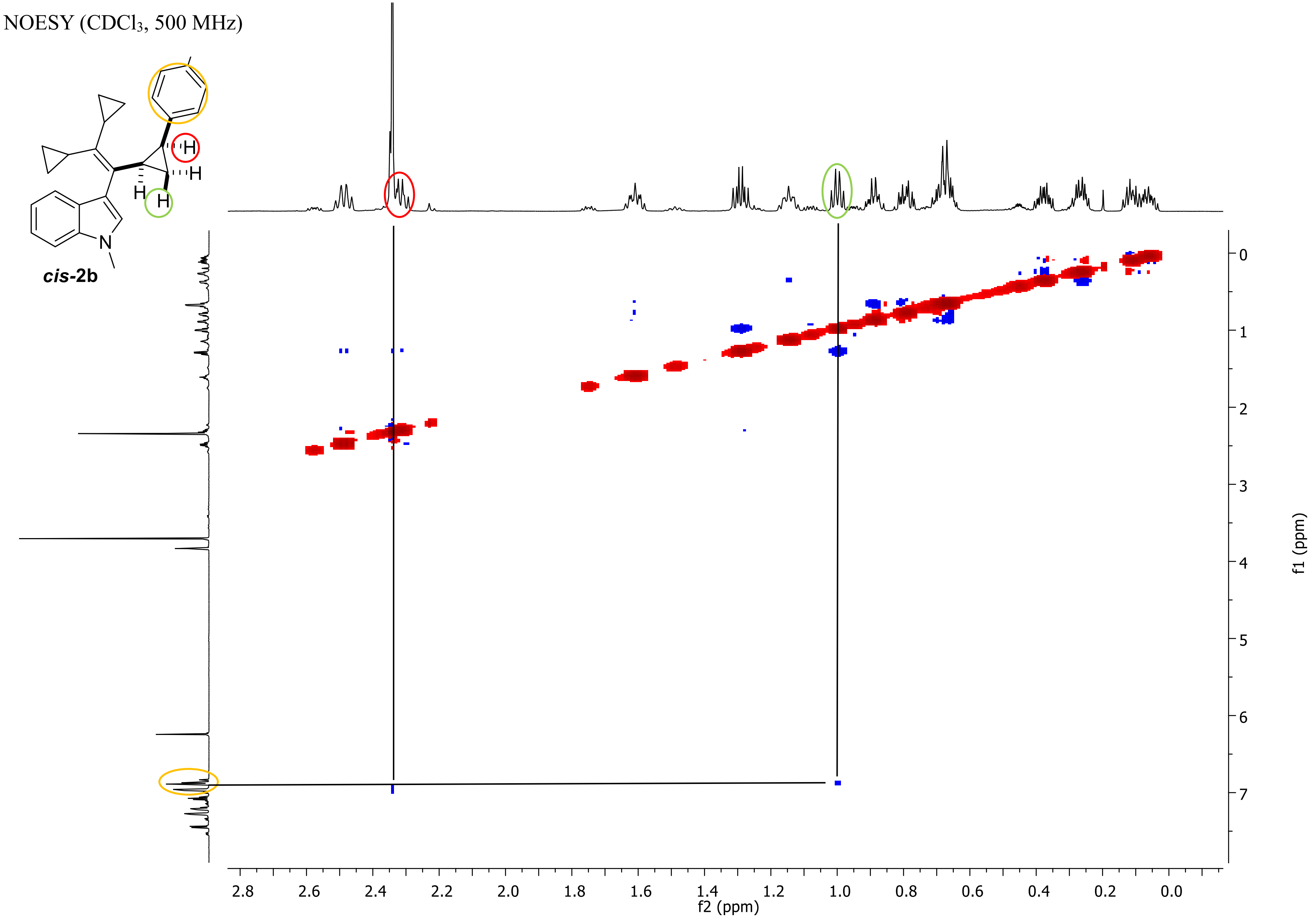

^{1}H NMR (CDCl$_3$, 300 MHz)

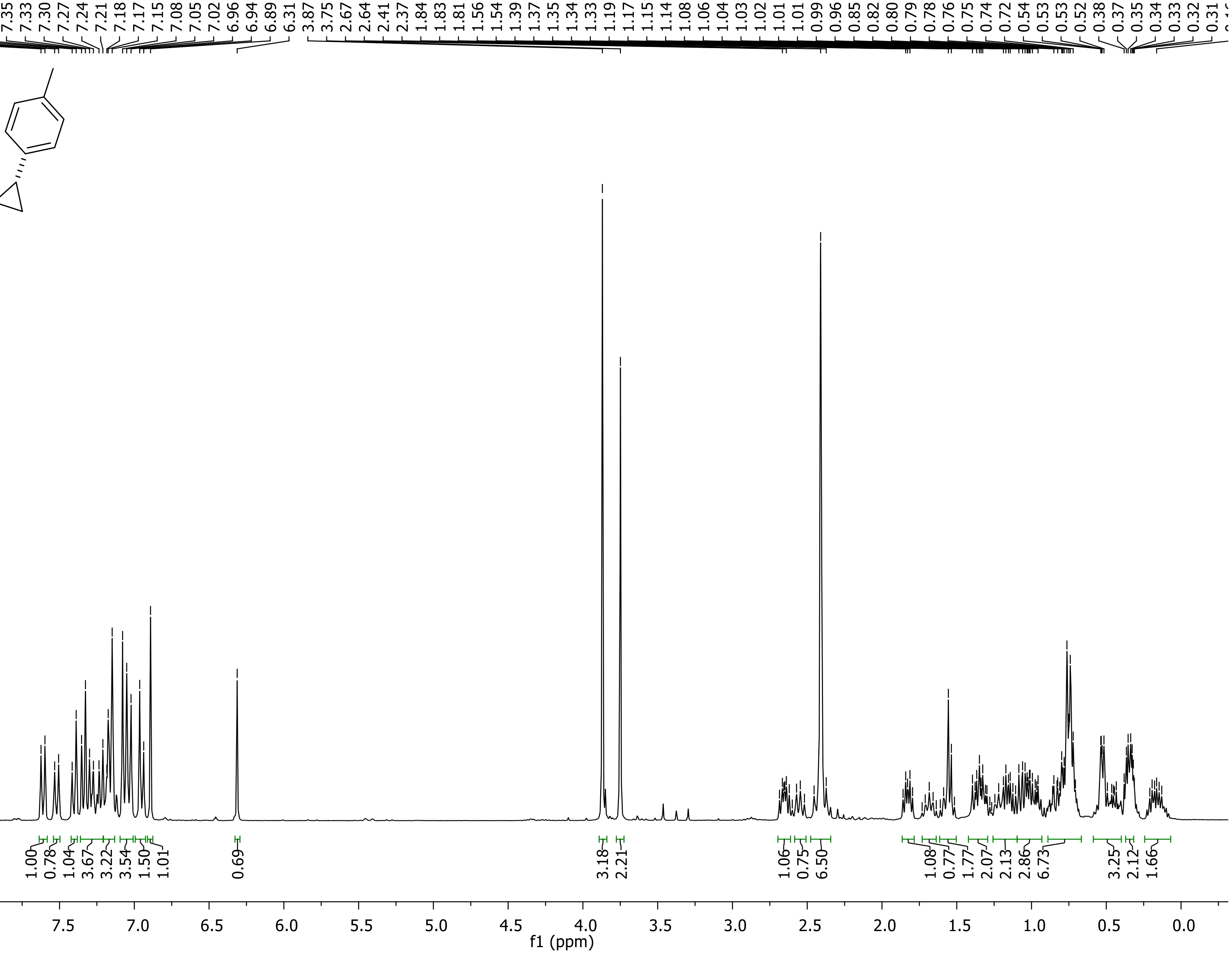

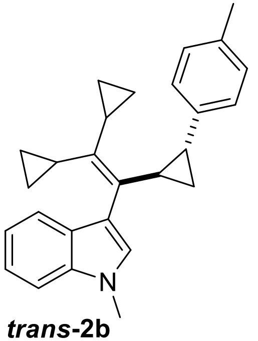

***trans*-2b**

d.r. 1.5/1

^{13}C NMR ($CDCl_3$, 75.4 MHz)

***trans*-2b**

d.r. 1.5/1

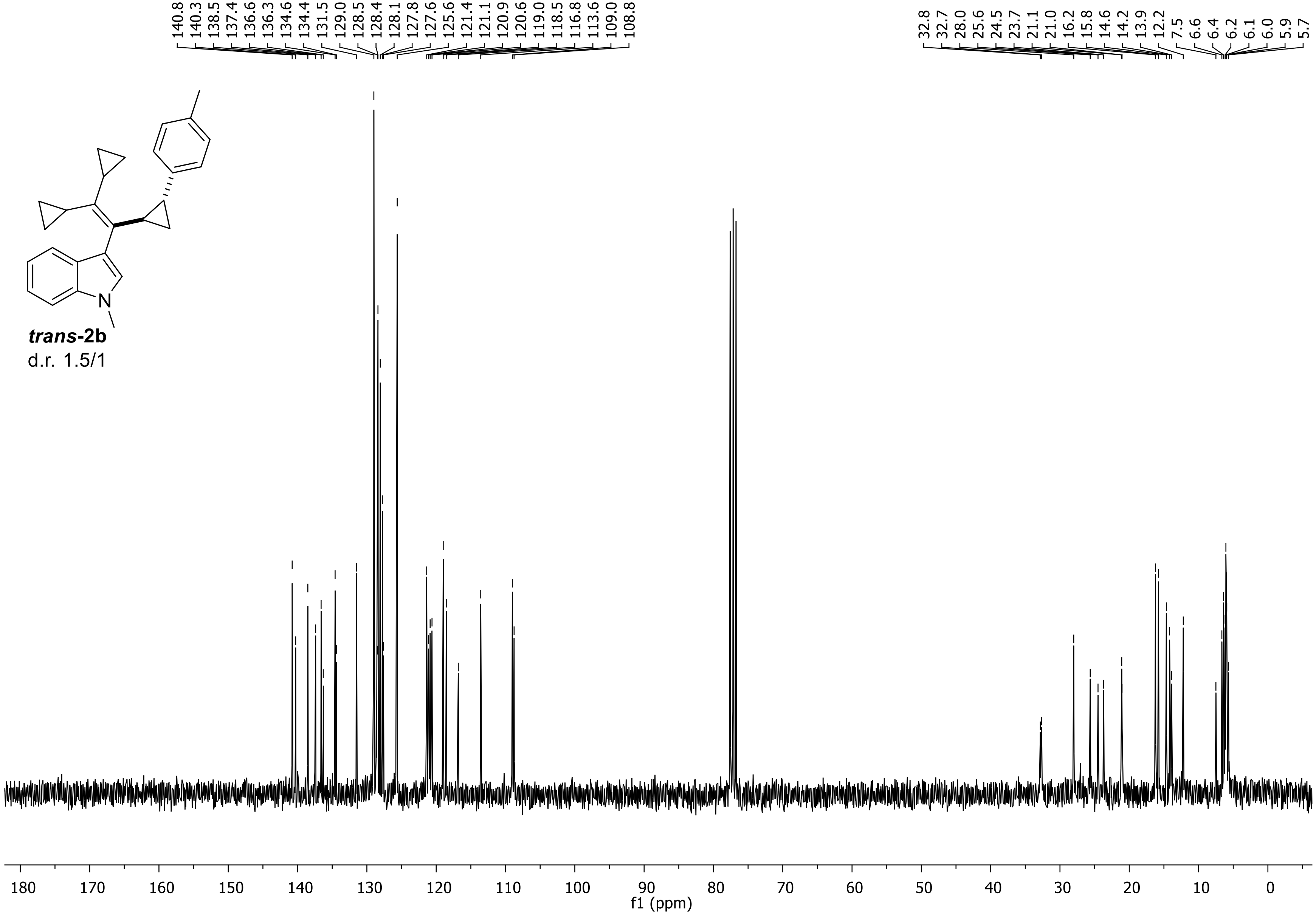

^{1}H NMR ($CDCl_3$, 300 MHz)

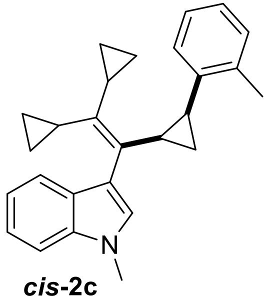

***cis*-2c**
d.r. 8/1

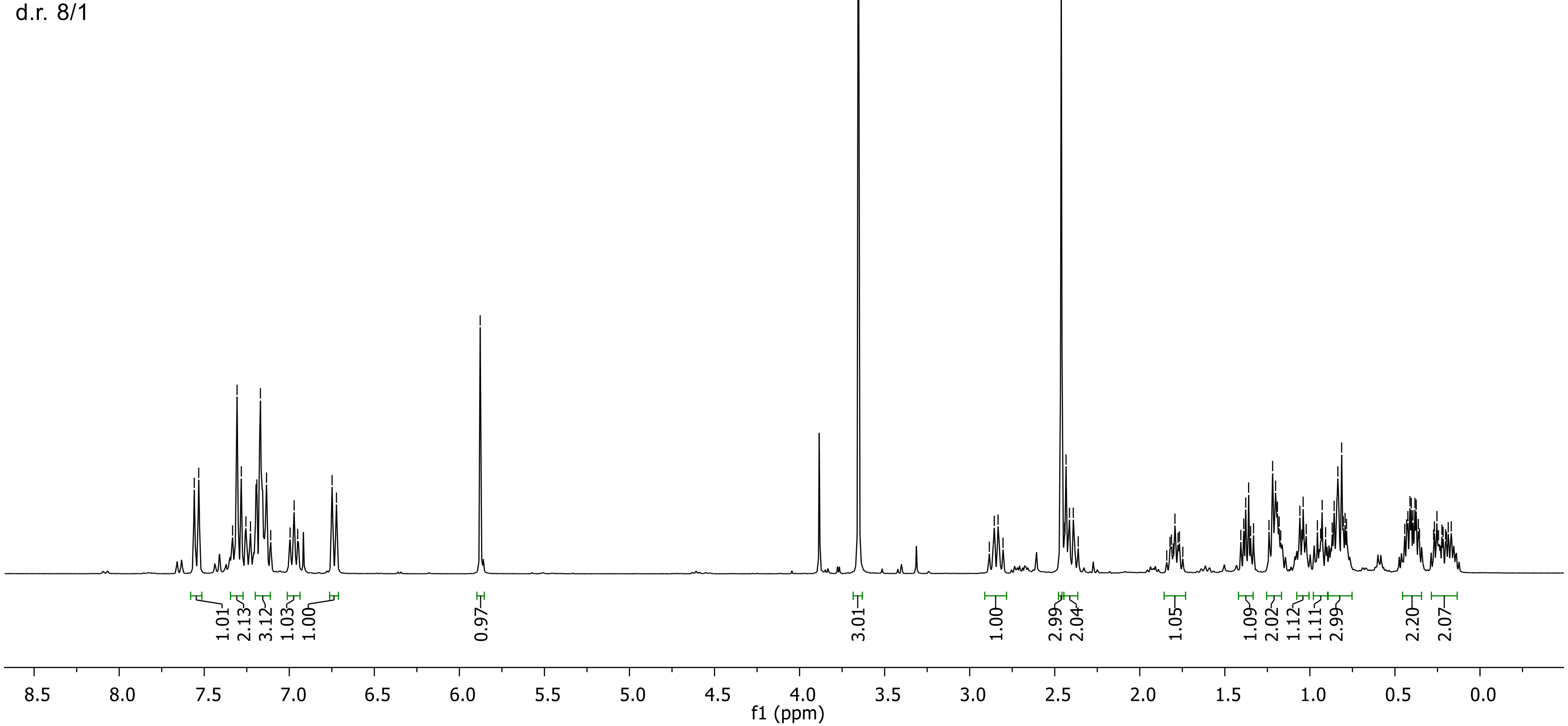

^{13}C NMR ($CDCl_3$, 75.4 MHz)

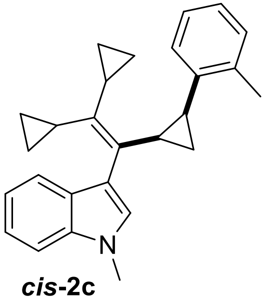

***cis*-2c**
d.r. 8/1

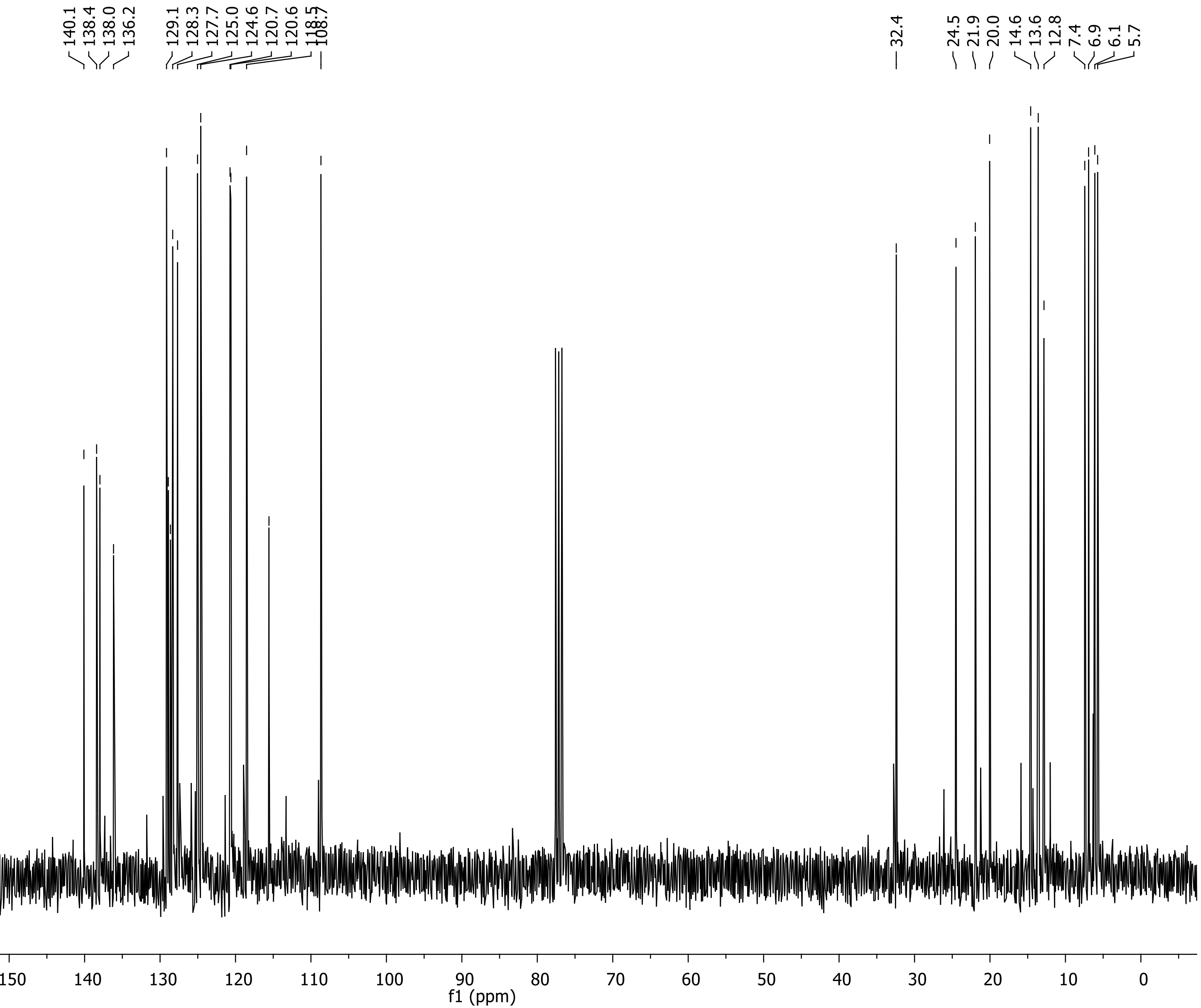

^{1}H NMR ($CDCl_3$, 500 MHz)

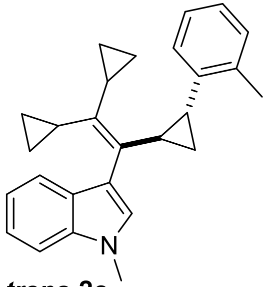

***trans*-2c**
d.r. 6.5/1

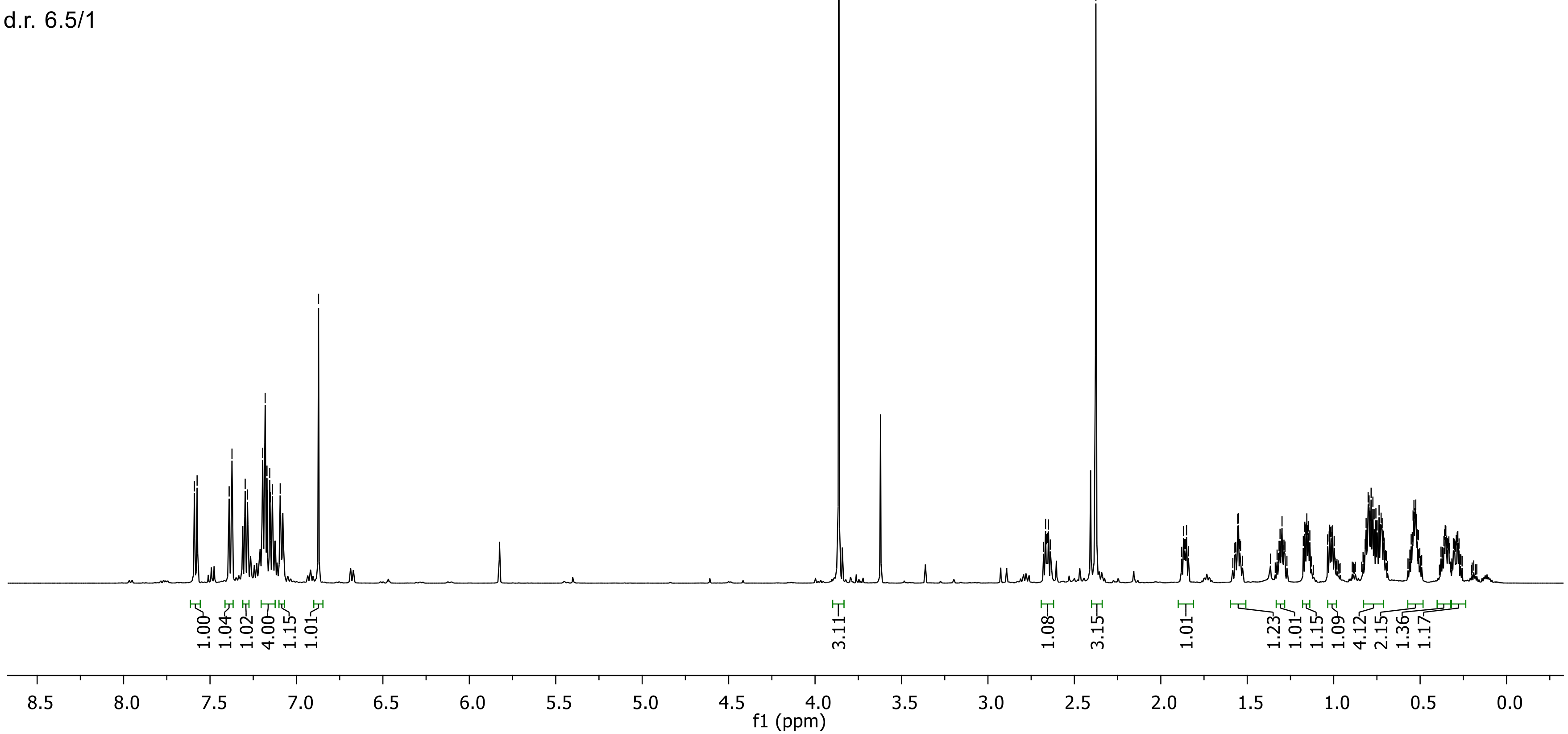

^{13}C NMR ($CDCl_3$, 75.4 MHz)

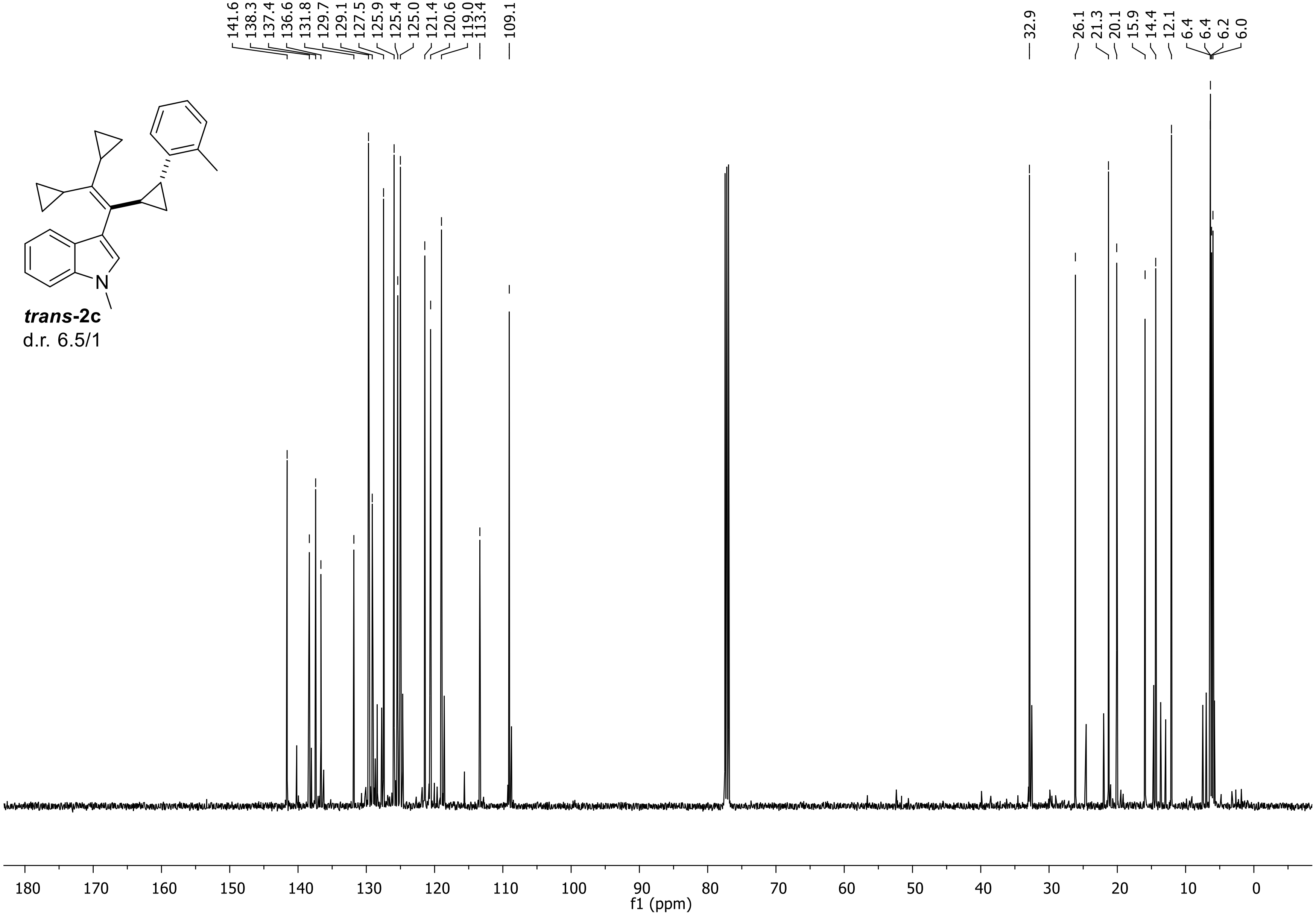

^{1}H NMR ($CDCl_3$, 300 MHz)

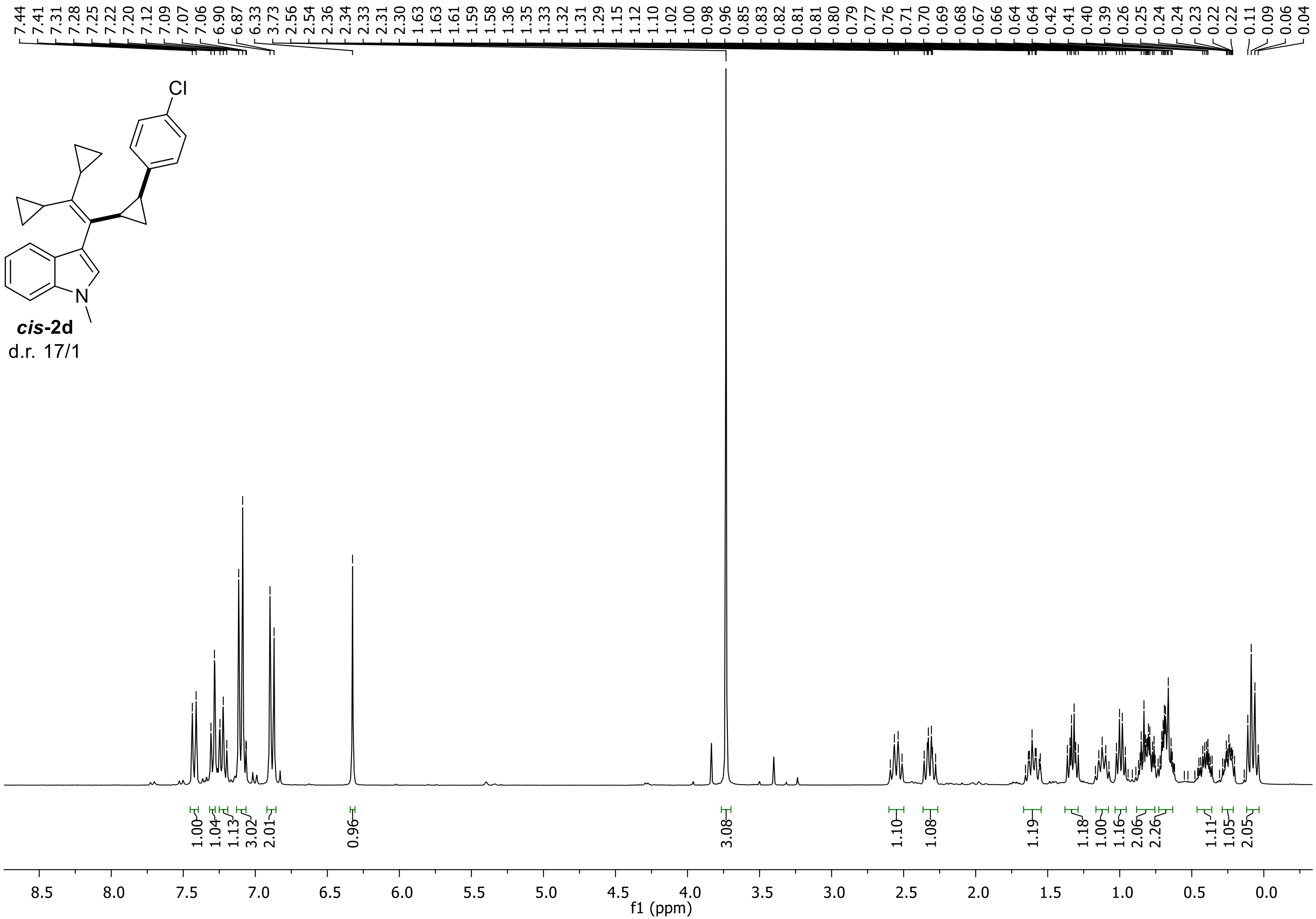

^{13}C NMR ($CDCl_3$, 75.4 MHz)

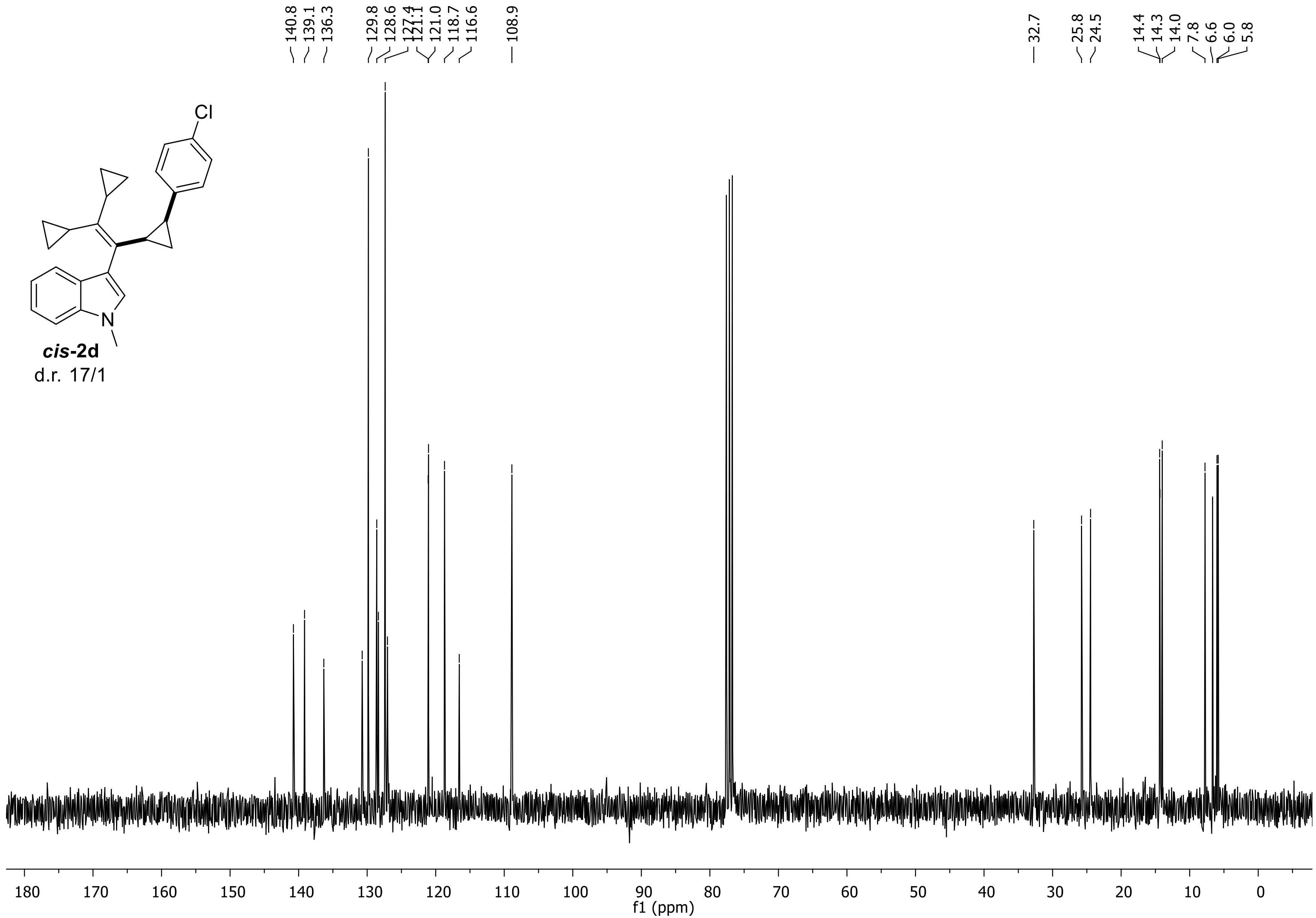

^{1}H NMR ($CDCl_3$, 300 MHz)

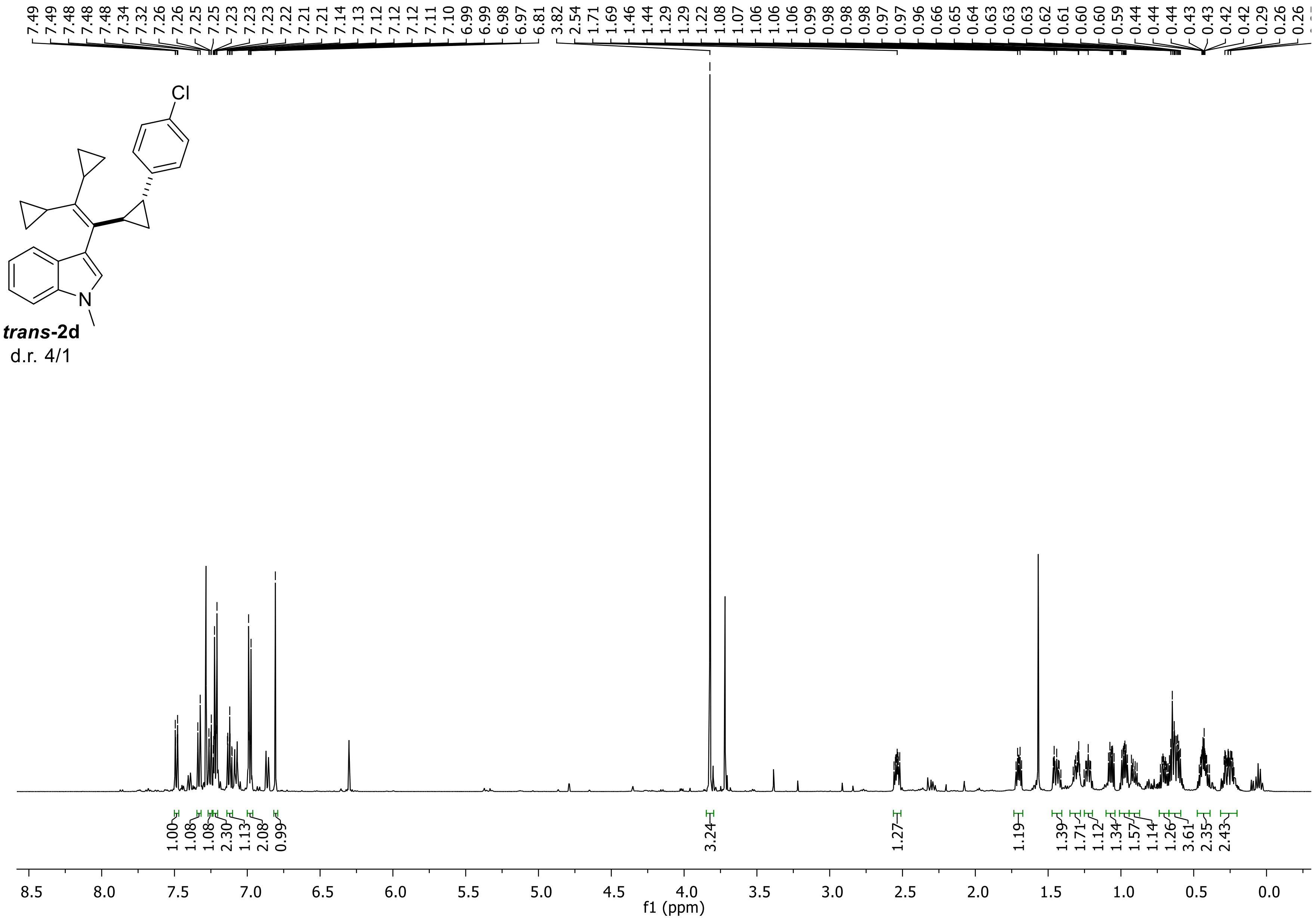

^{13}C NMR ($CDCl_3$, 75.4 MHz)

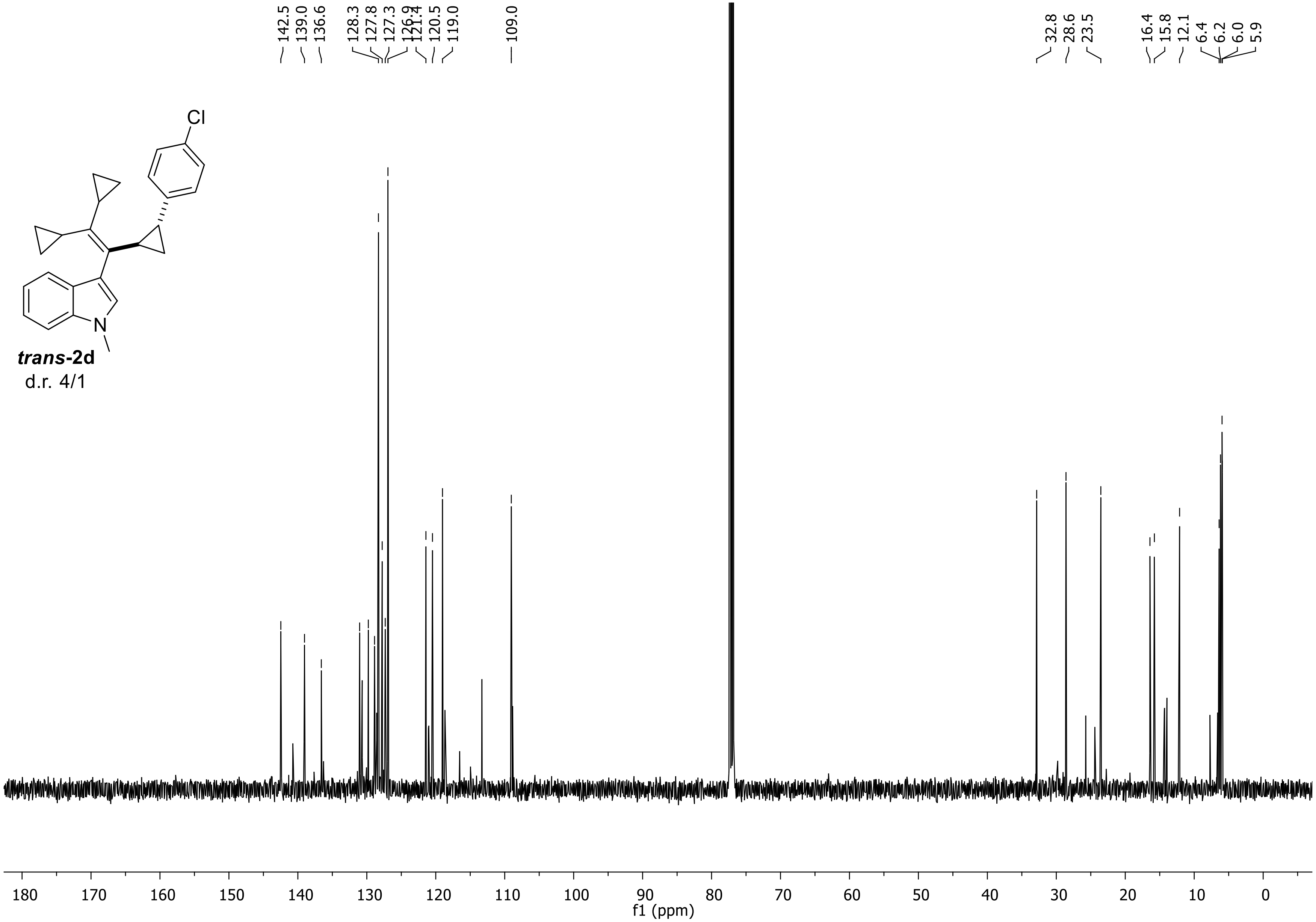

^{1}H NMR ($CDCl_3$, 300 MHz)

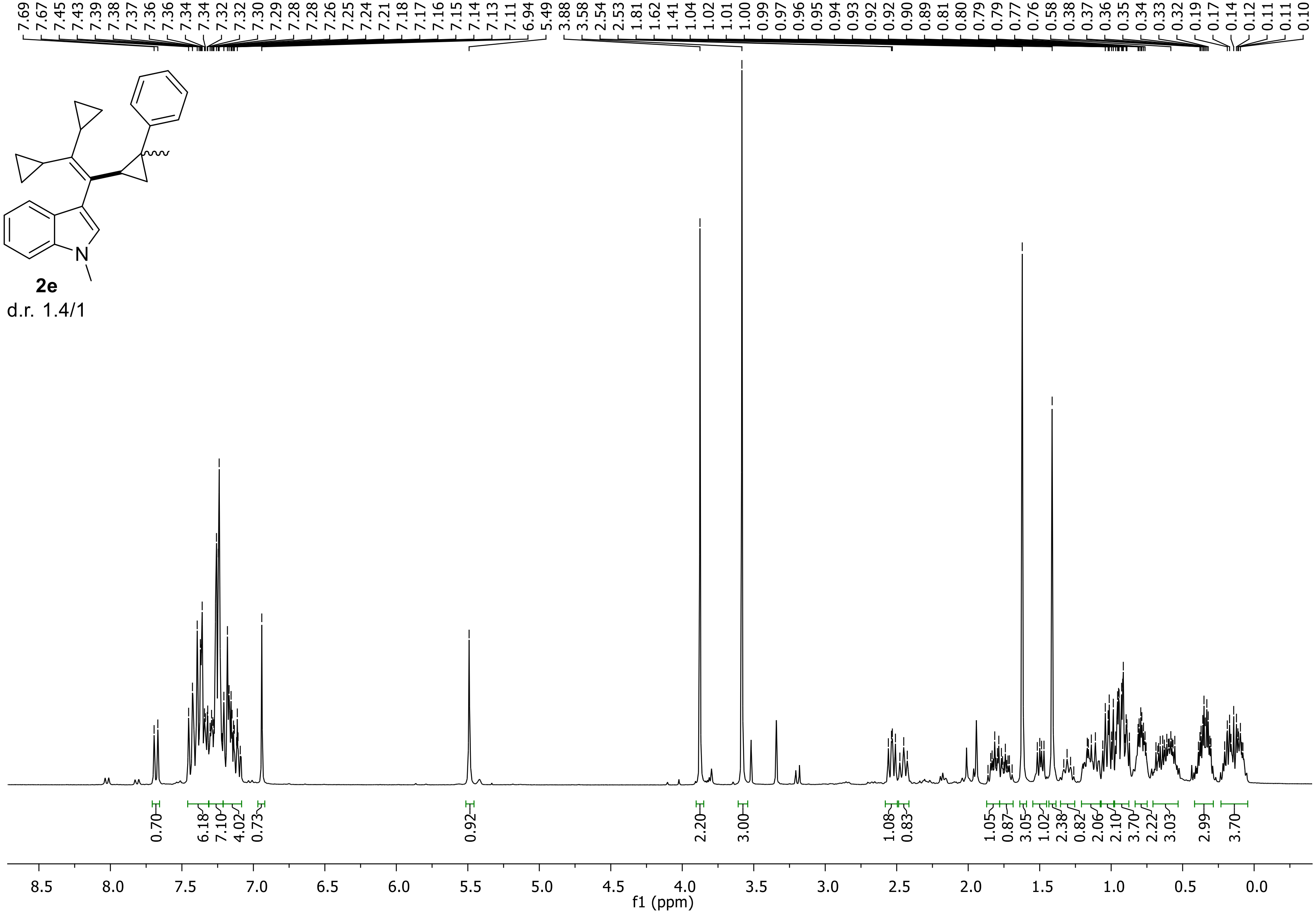

^{13}C NMR ($CDCl_3$, 75.4 MHz)

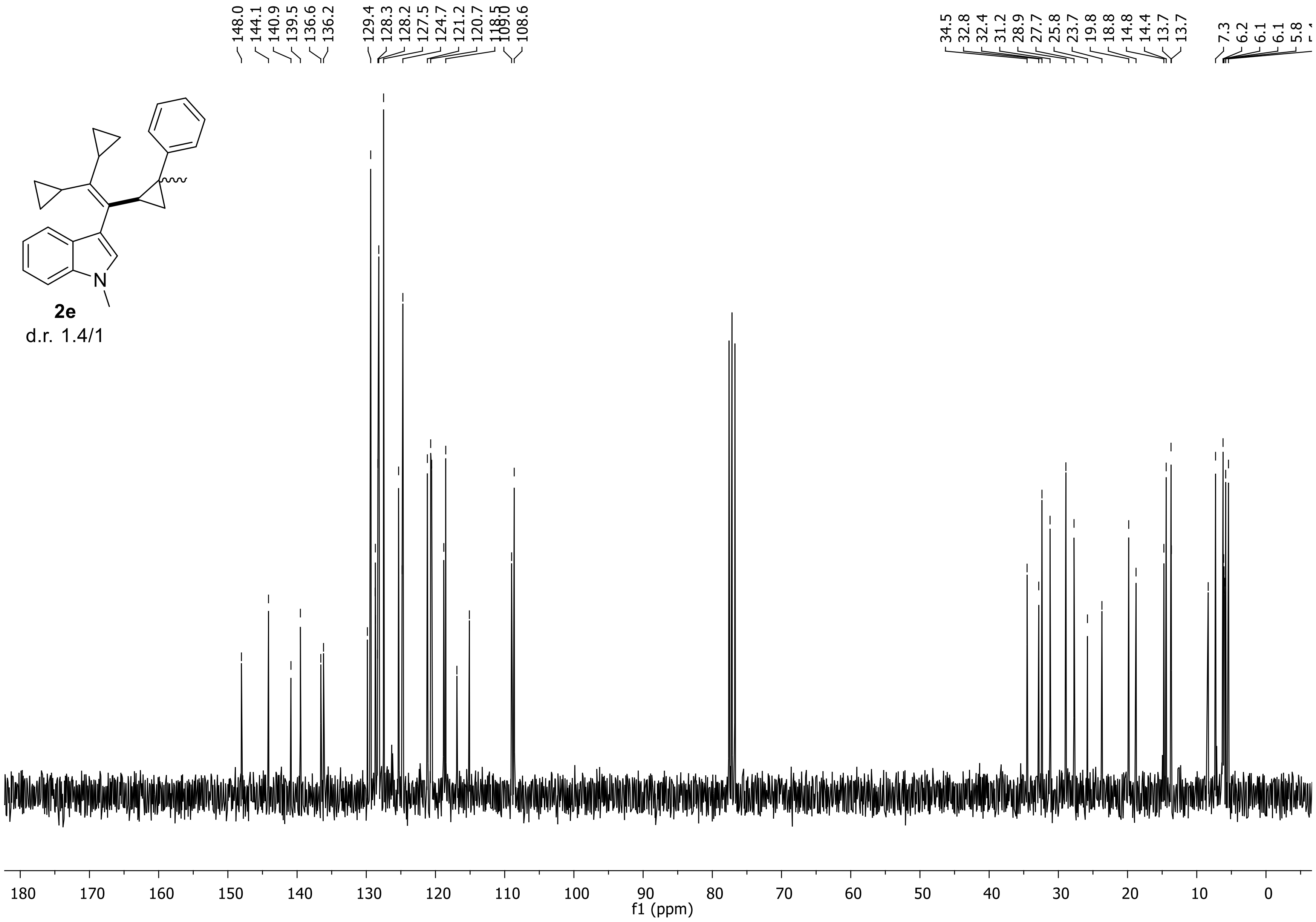

2e
d.r. 1.4/1

^{1}H NMR ($CDCl_3$, 300 MHz)

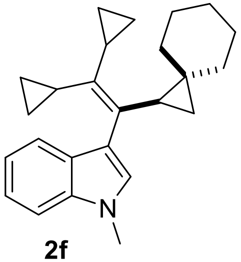

2f

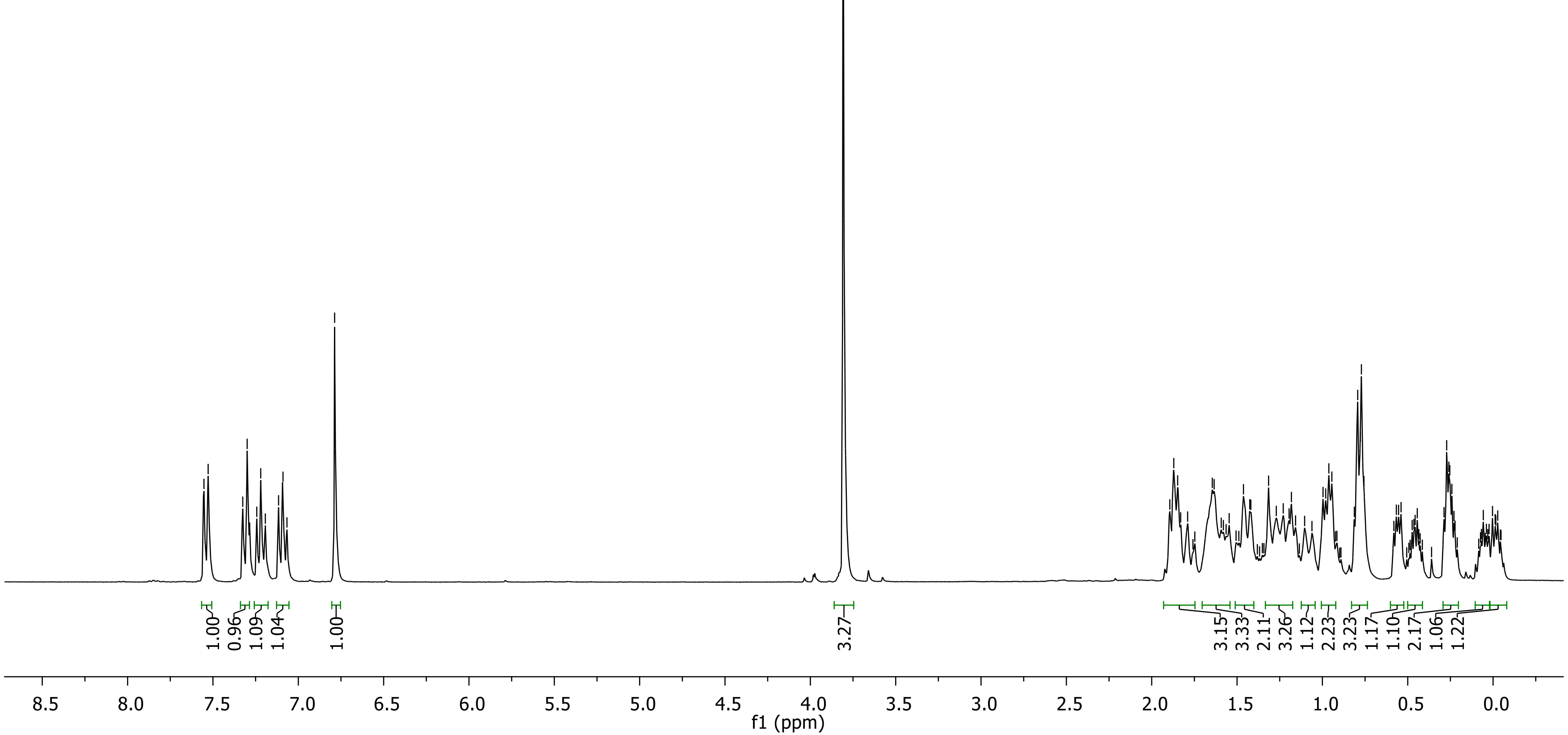

^{13}C NMR ($CDCl_3$, 75.4 MHz)

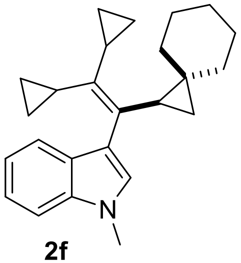

2f

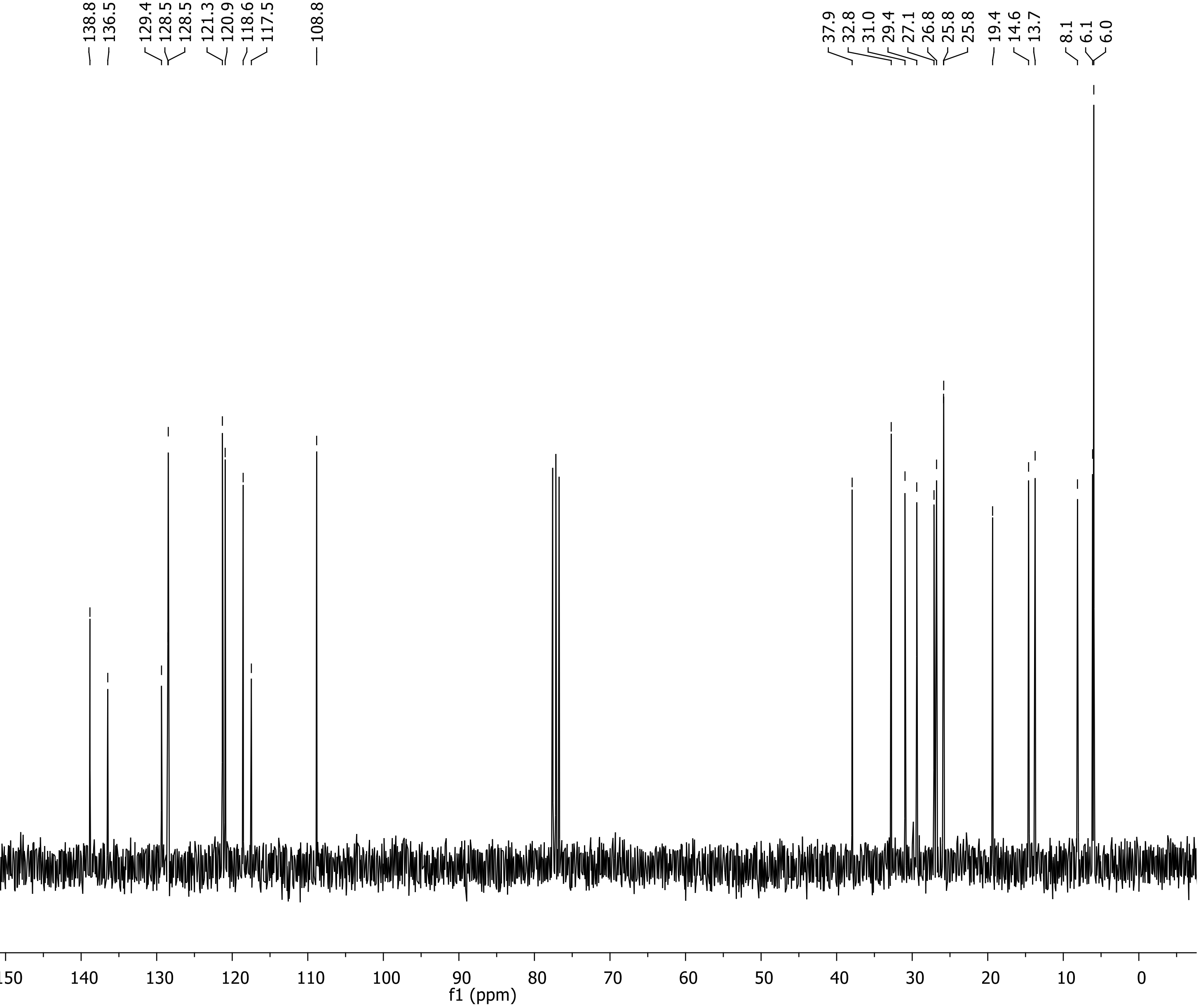

^{1}H NMR ($CDCl_3$, 300 MHz)

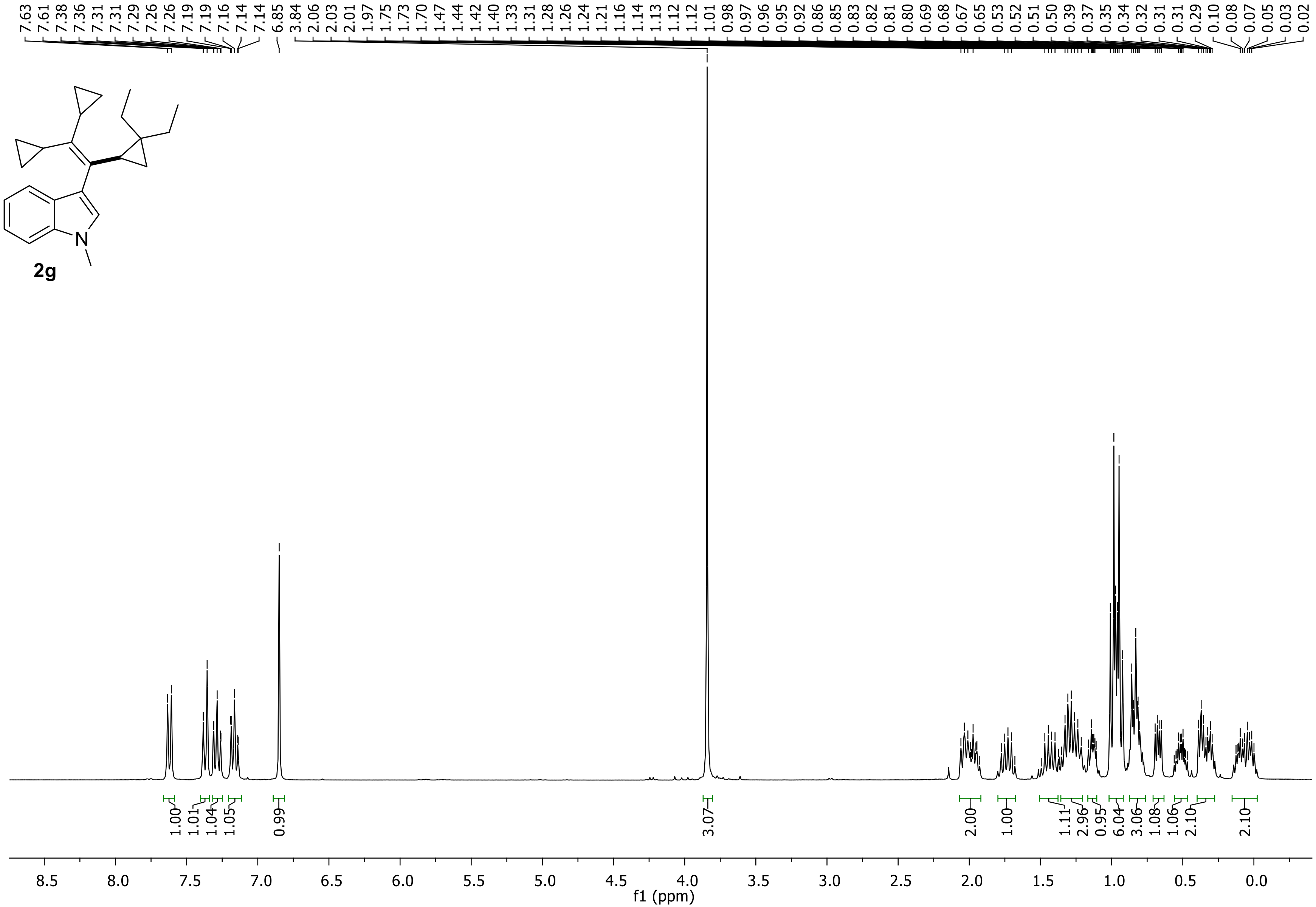

2g

^{13}C NMR ($CDCl_3$, 75.4 MHz)

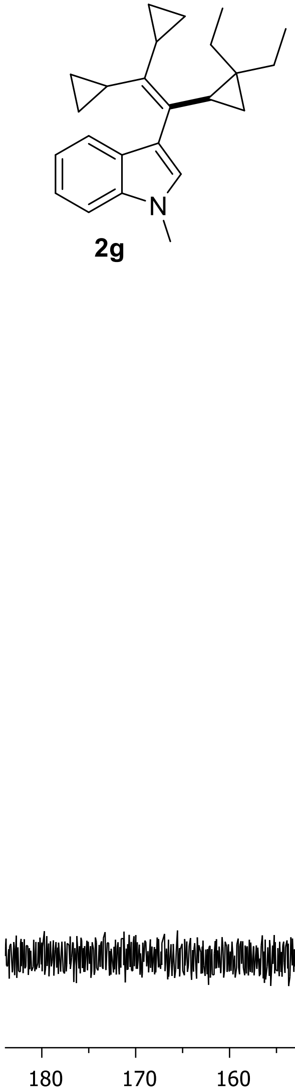

2g

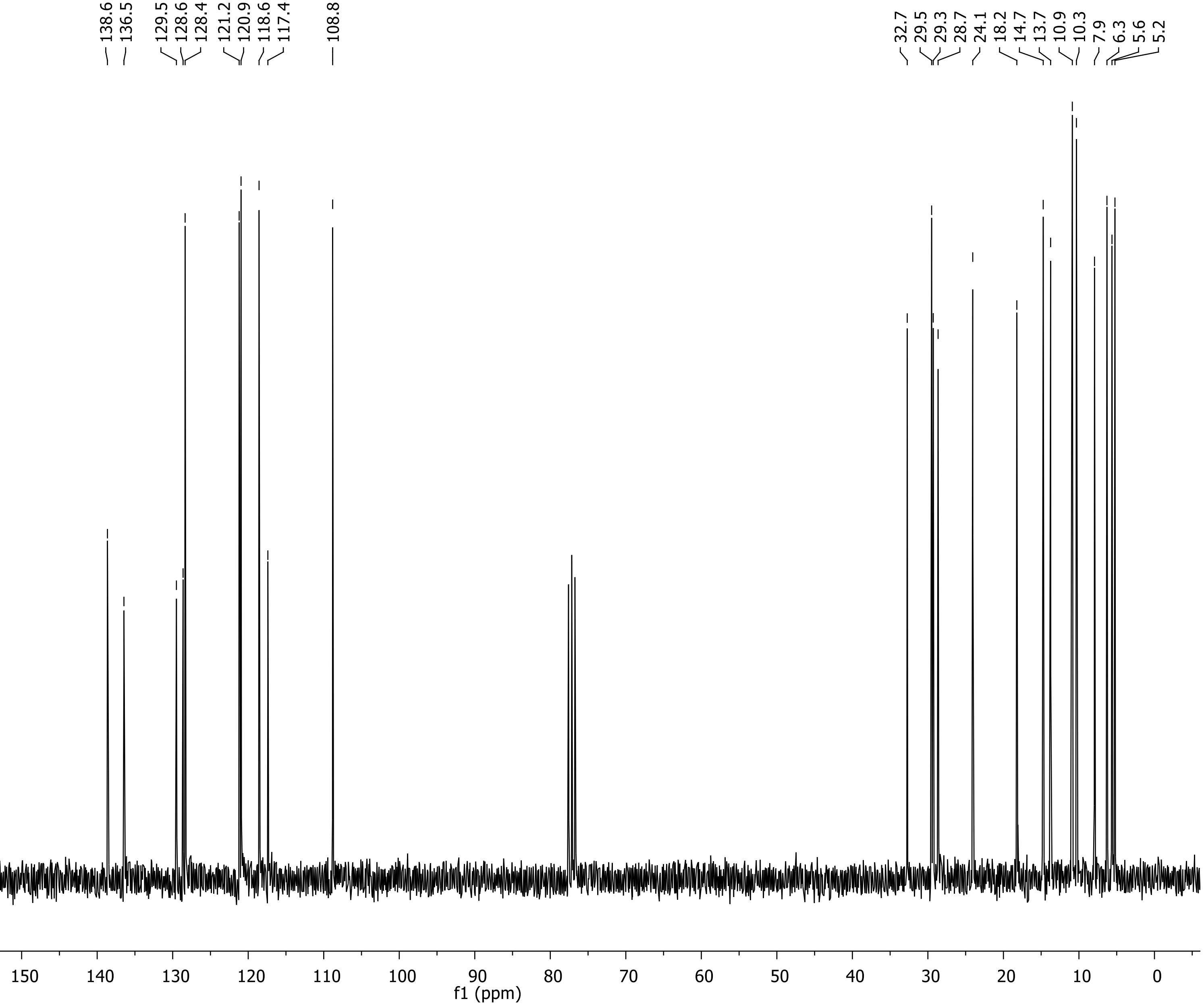

^{1}H NMR ($CDCl_3$, 300 MHz)

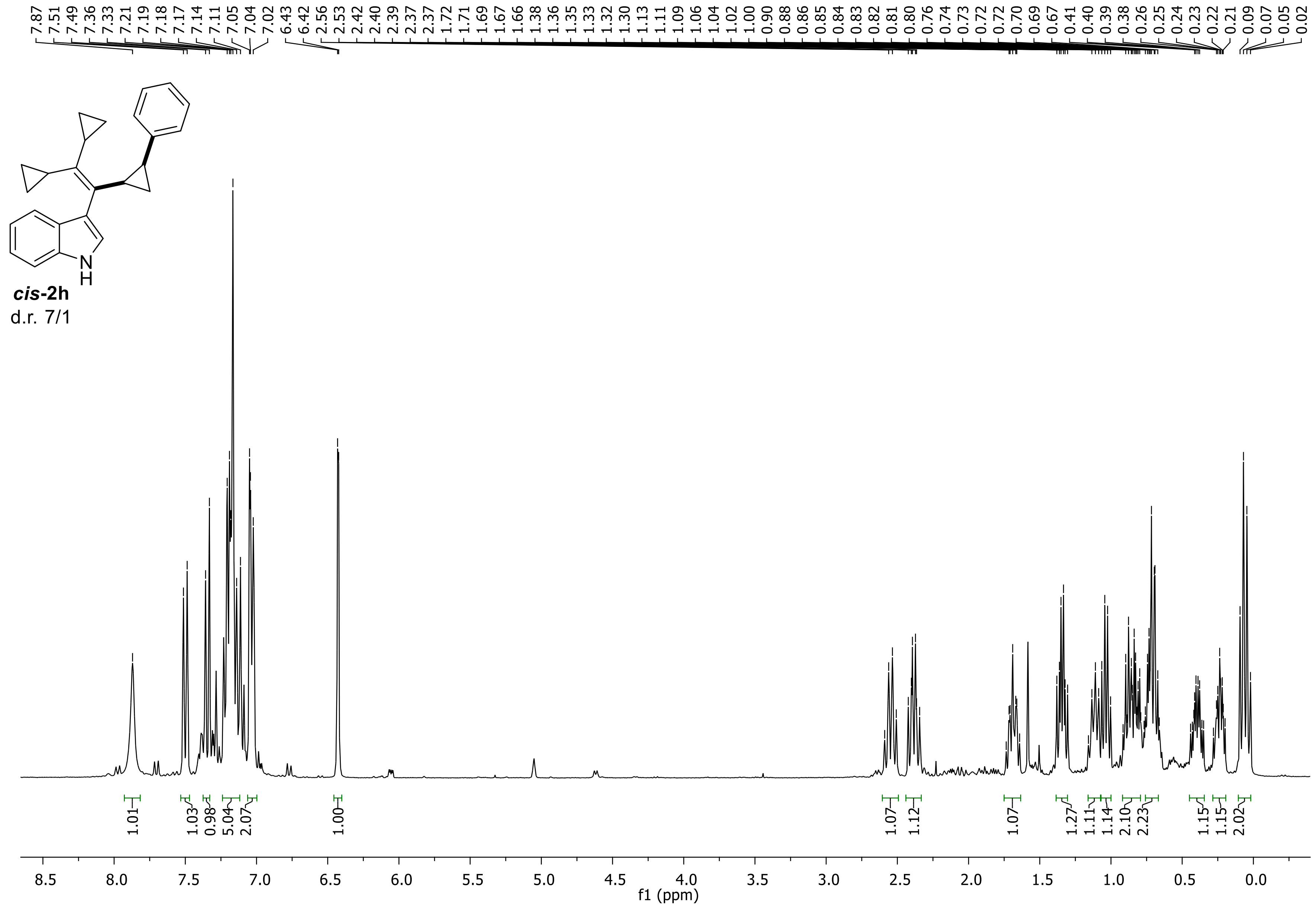

^{13}C NMR ($CDCl_3$, 75.4 MHz)

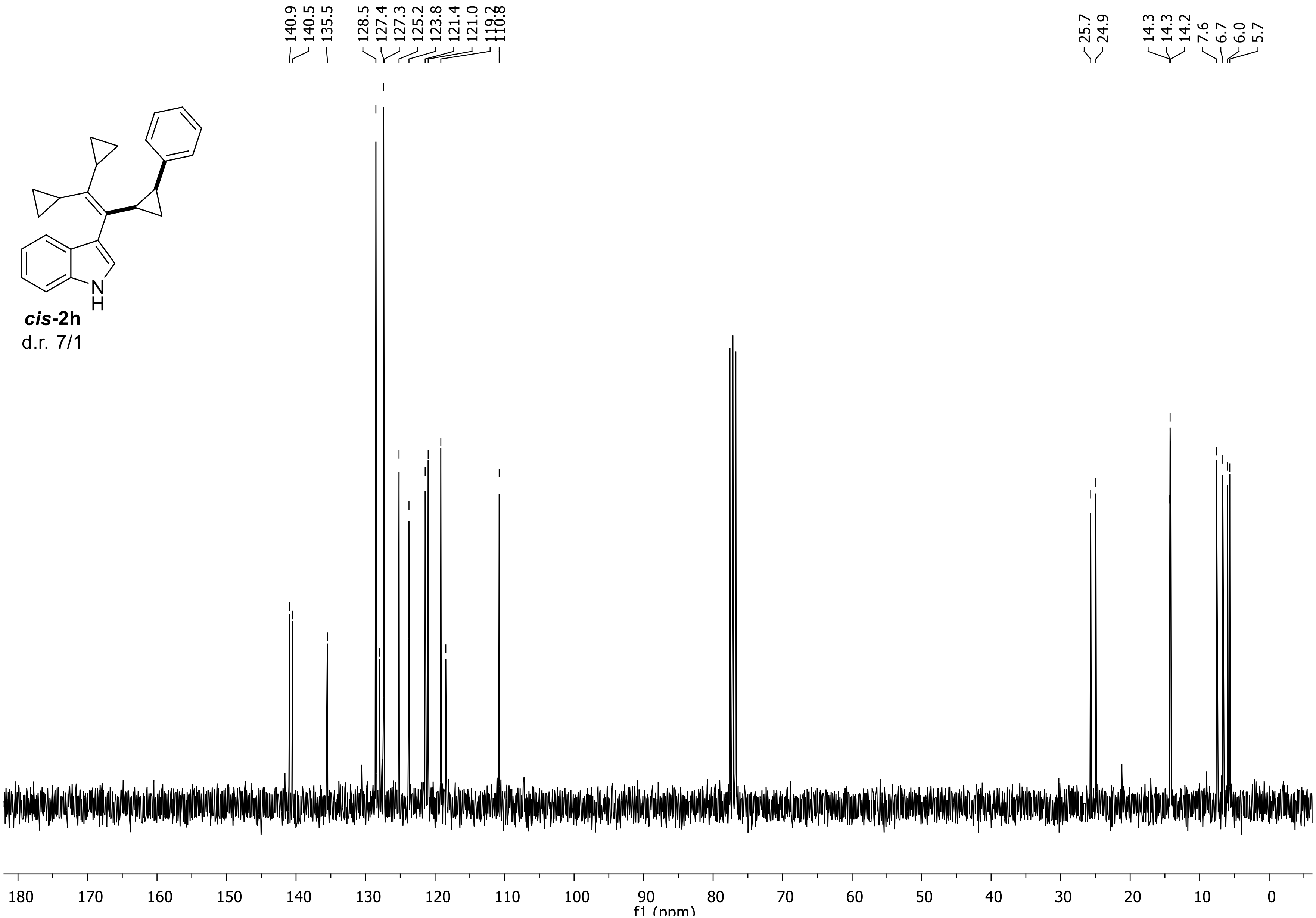

^{1}H NMR ($CDCl_3$, 300 MHz)

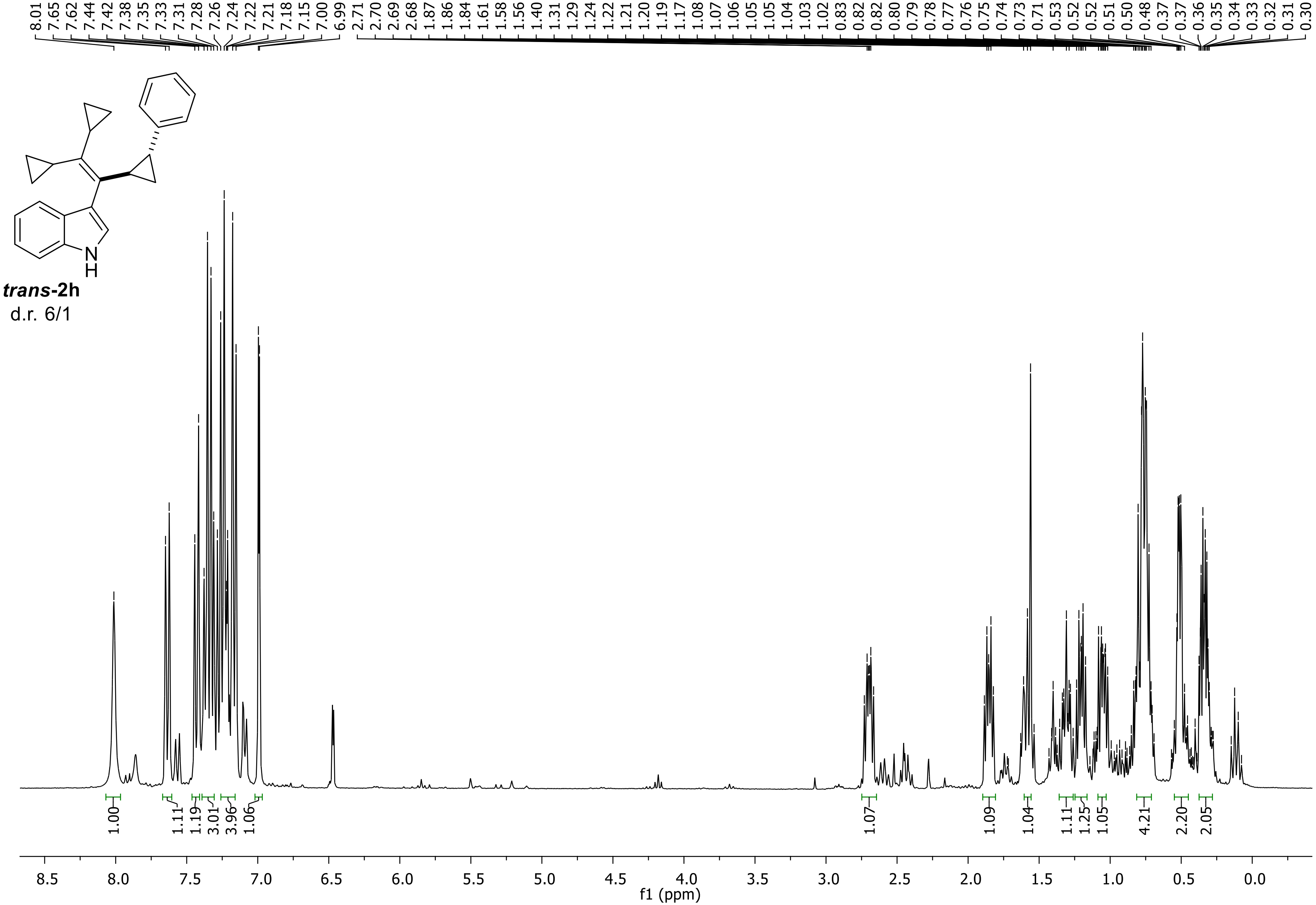

^{13}C NMR ($CDCl_3$, 75.4 MHz)

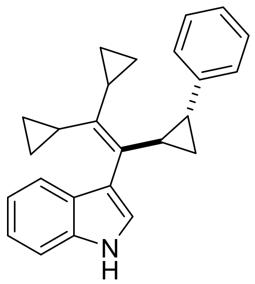

***trans*-2h**
d.r. 6/1

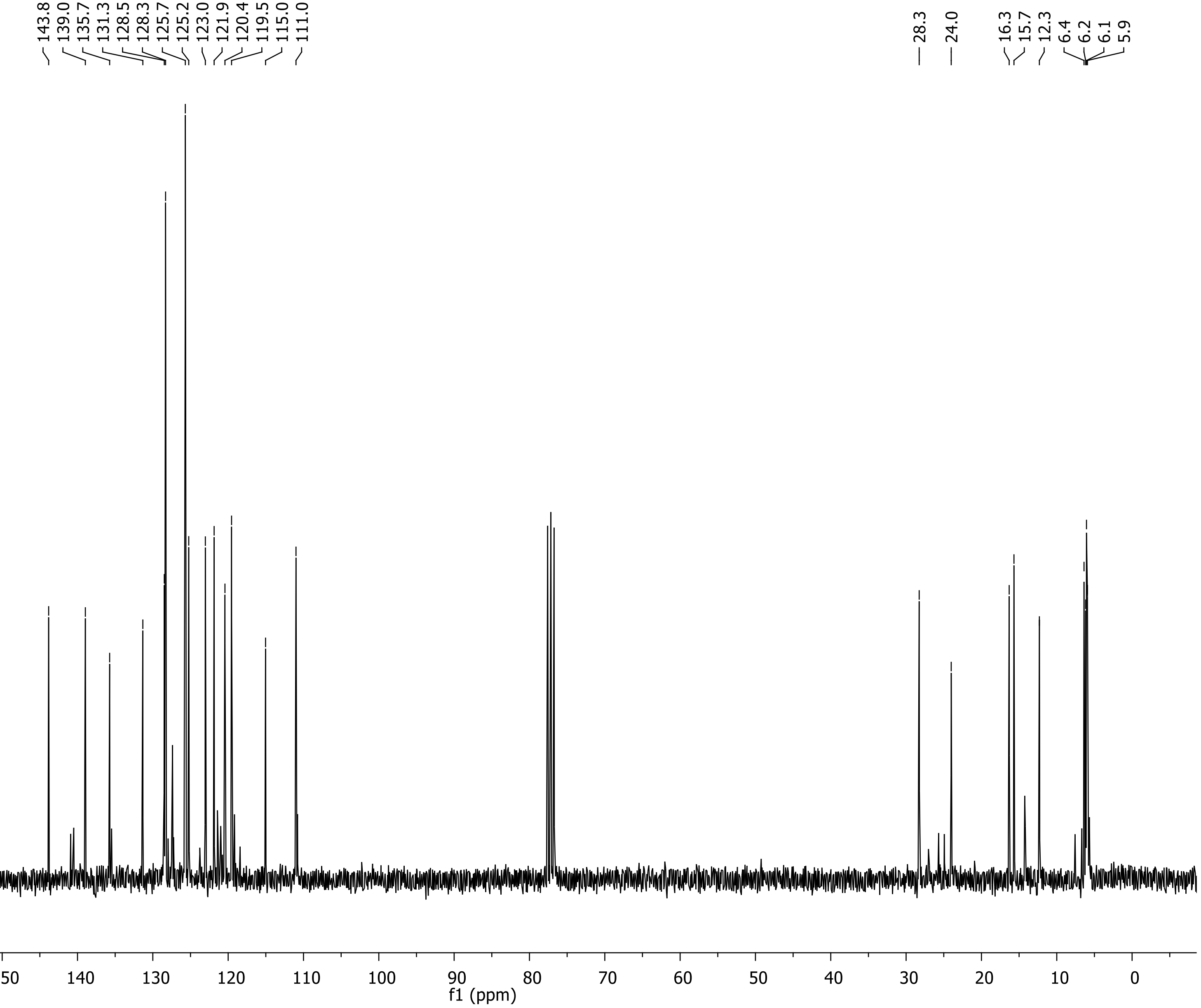

^{1}H NMR ($CDCl_3$, 300 MHz)

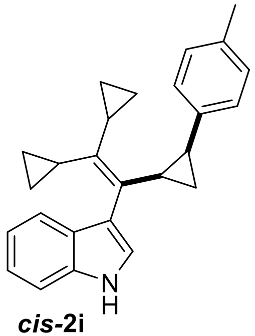

***cis*-2i**
d.r. 9/1

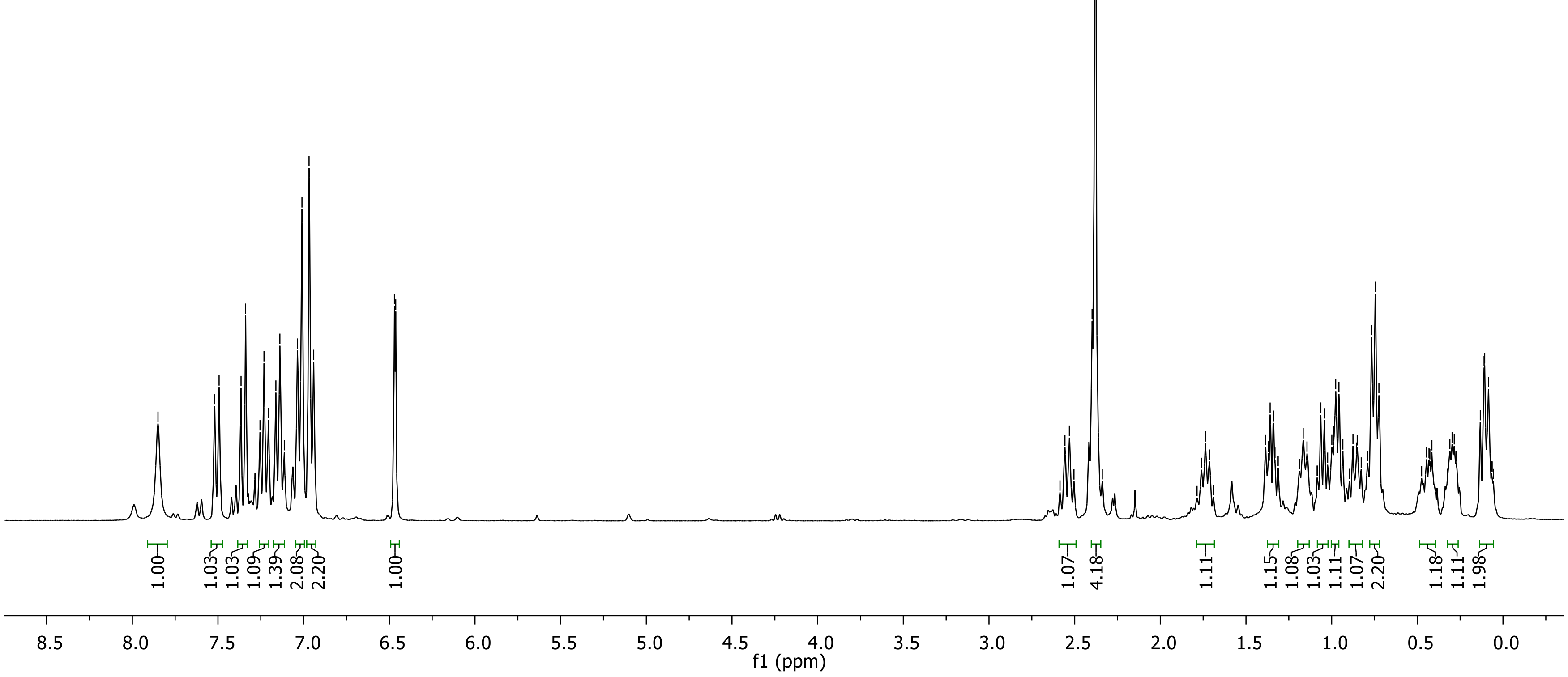

^{13}C NMR ($CDCl_3$, 75.4 MHz)

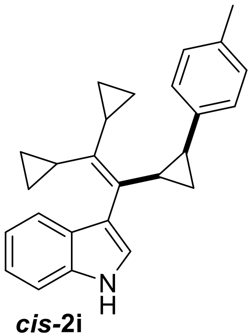

cis-**2i**
d.r. 9/1

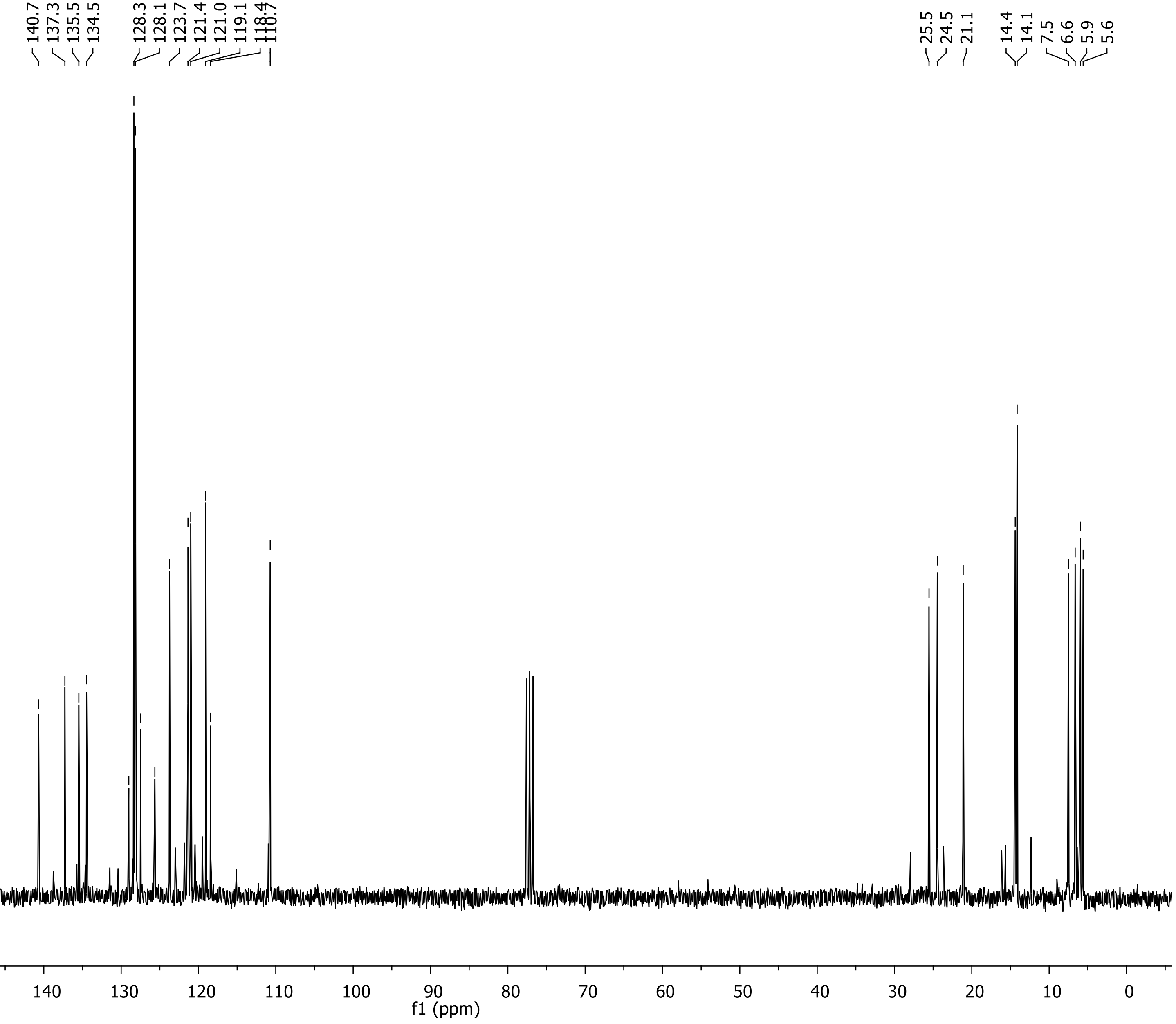

^{1}H NMR ($CDCl_3$, 500 MHz)

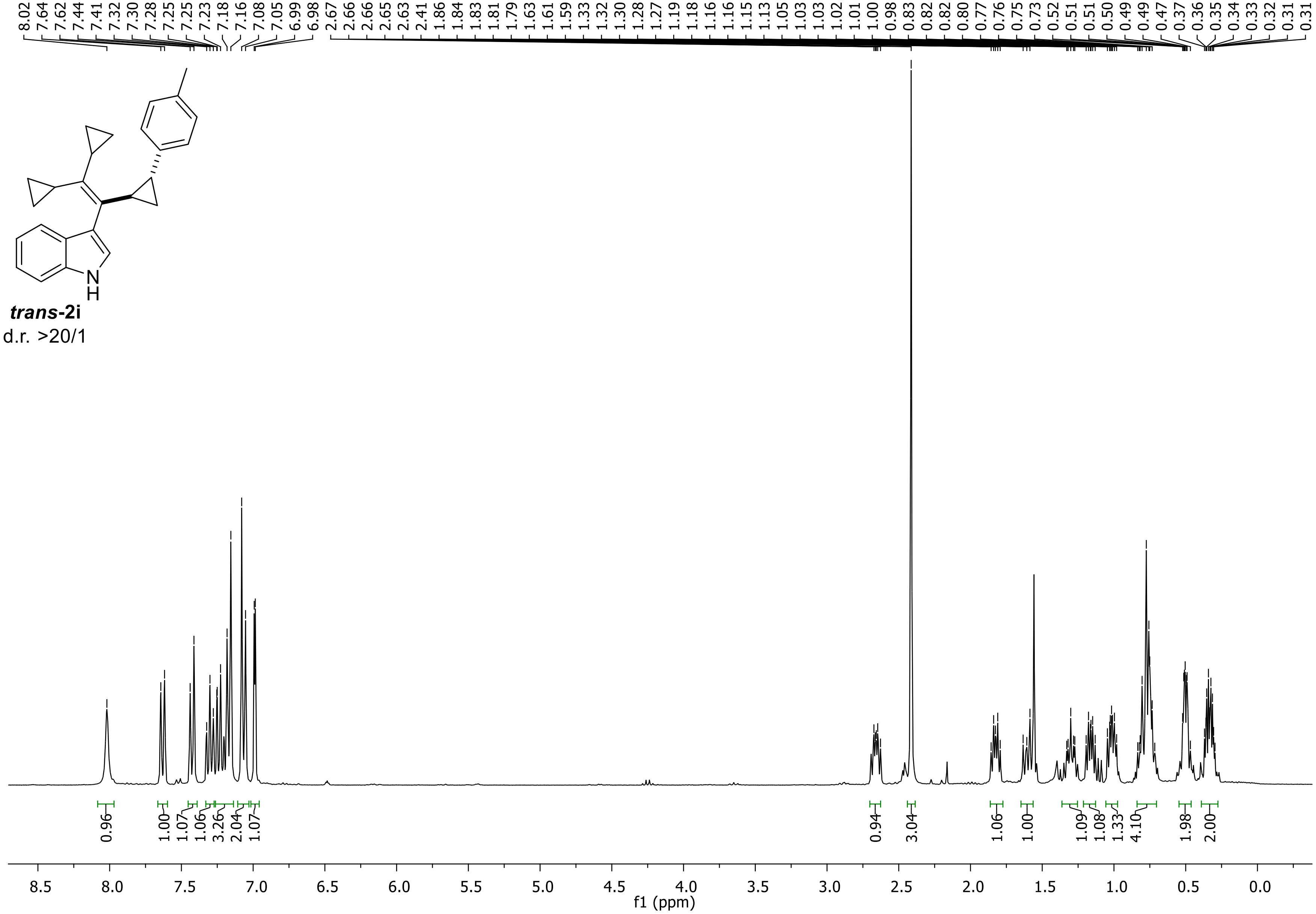

***trans*-2i**
d.r. >20/1

^{13}C NMR ($CDCl_3$, 75.4 MHz)

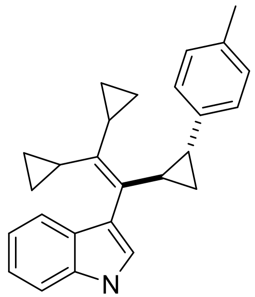

***trans*-2i**
d.r. >20/1

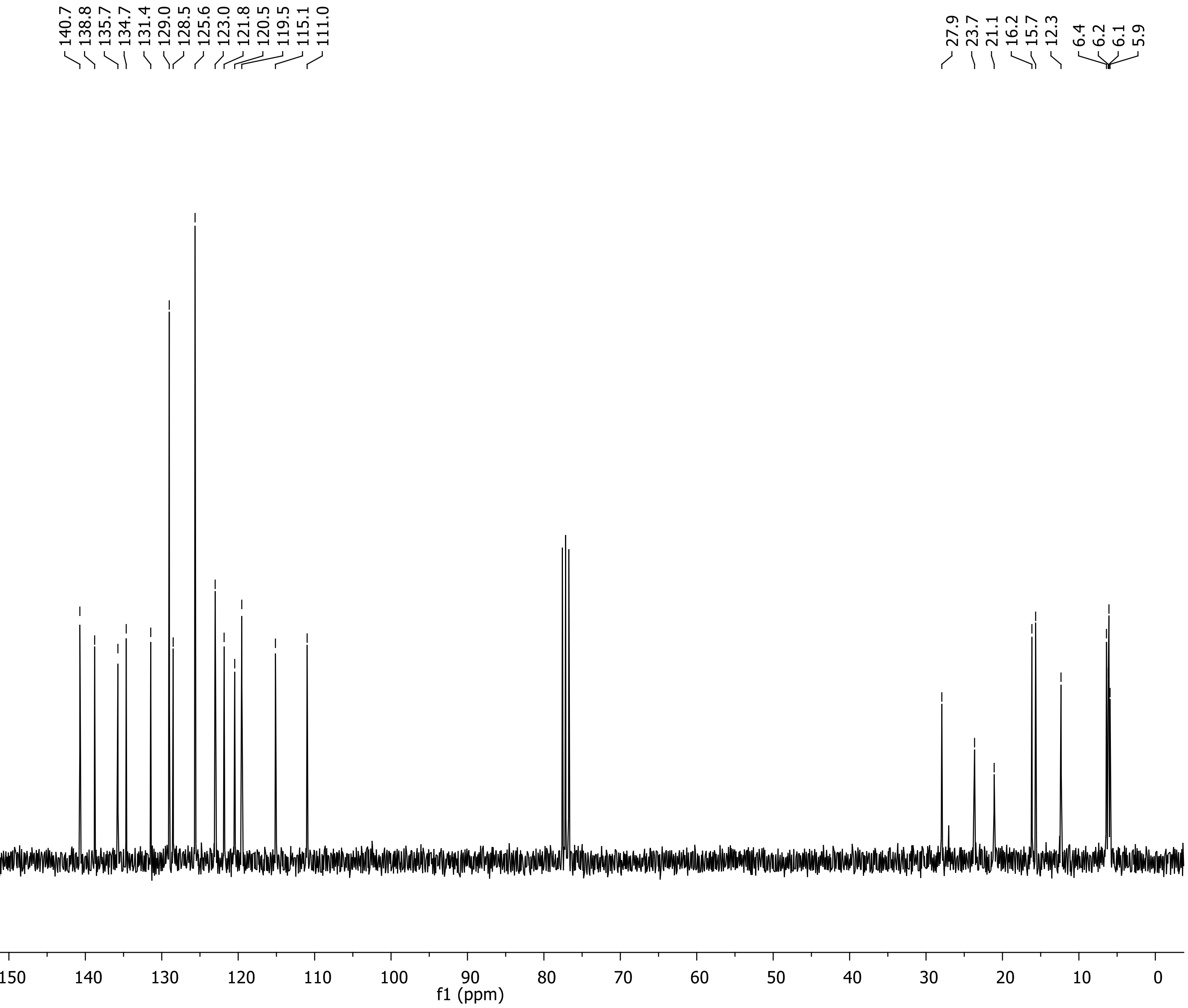

^{1}H NMR ($CDCl_3$, 300 MHz)

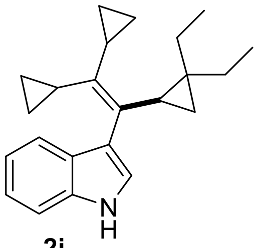
2j

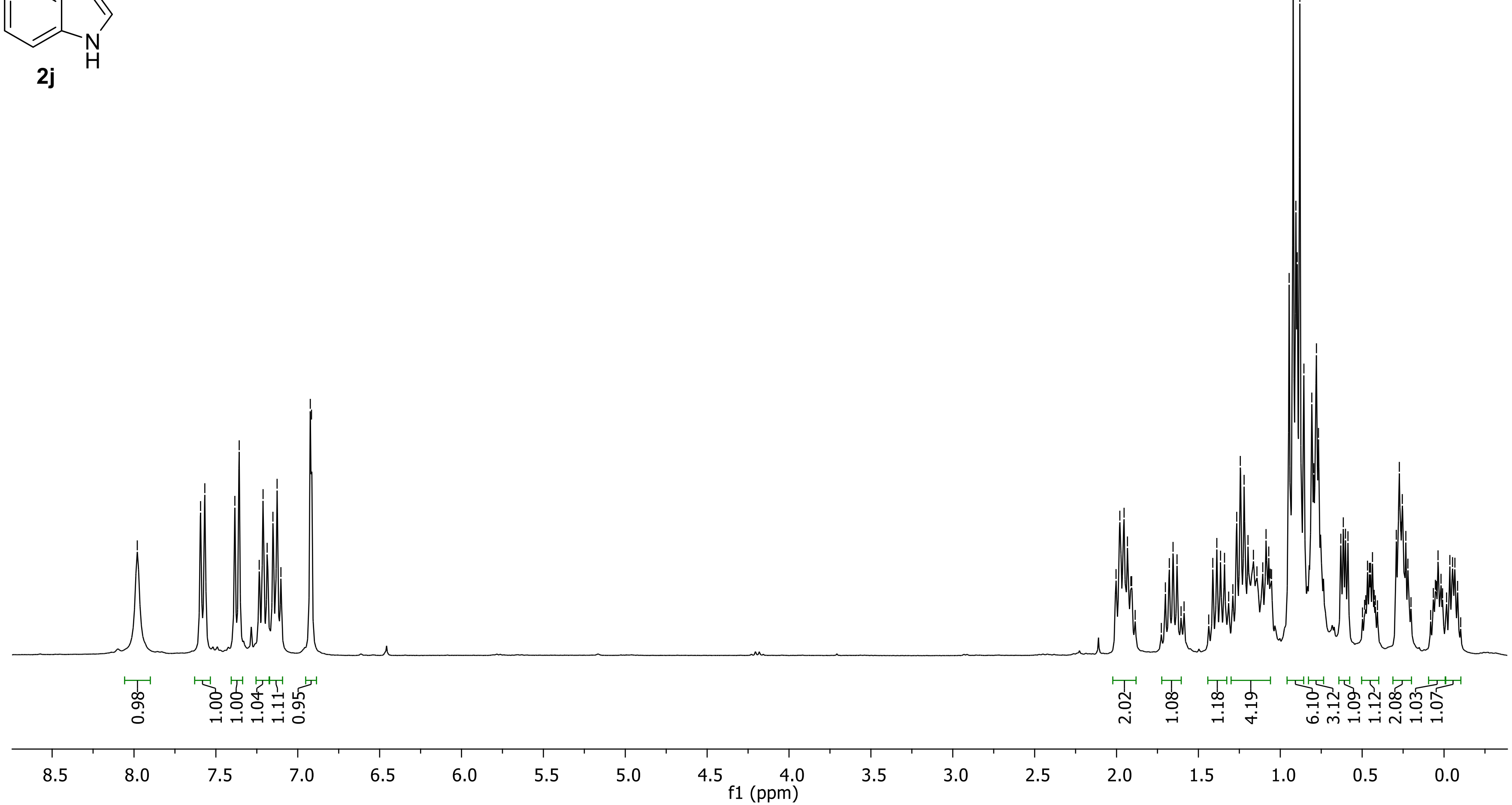

^{13}C NMR ($CDCl_3$, 75.4 MHz)

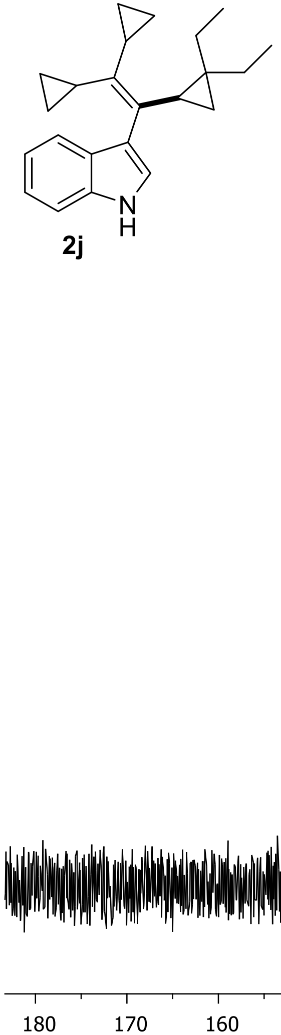

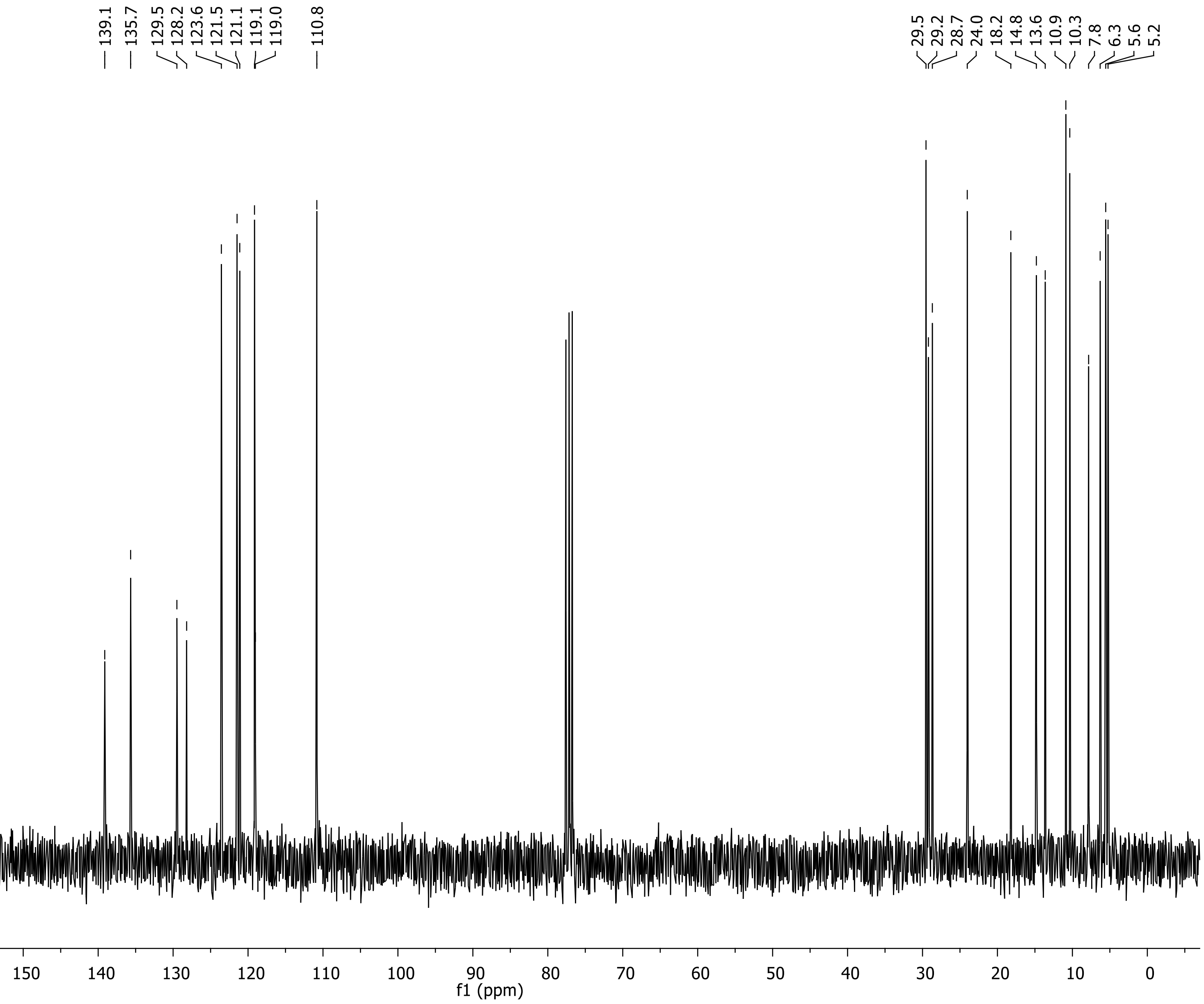

^{1}H NMR ($CDCl_3$, 300 MHz)

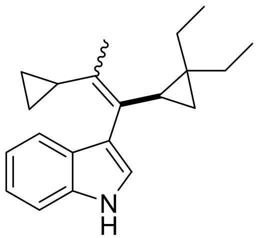

2k

E/Z = 2/1

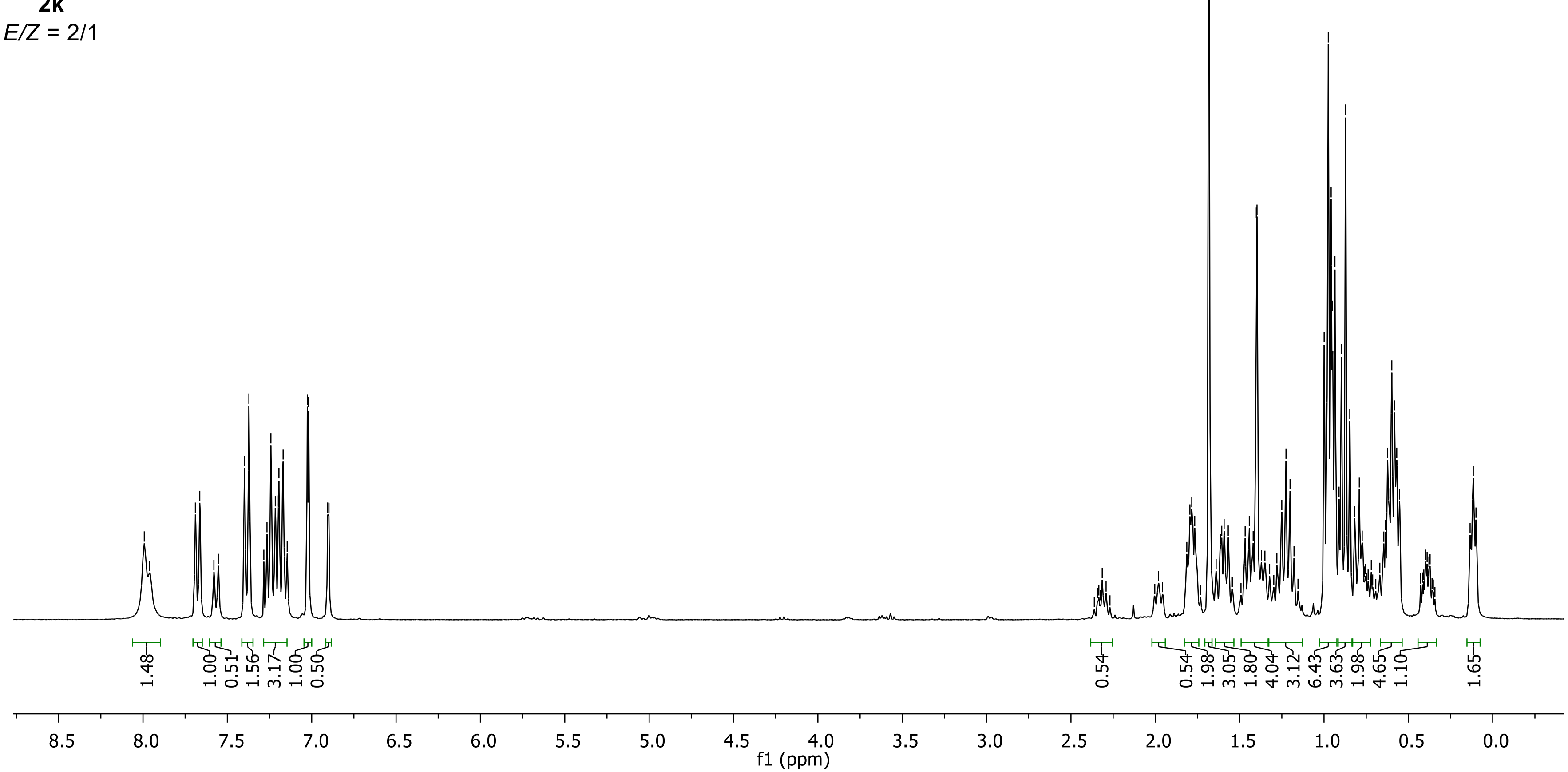

^{13}C NMR ($CDCl_3$, 75.4 MHz)

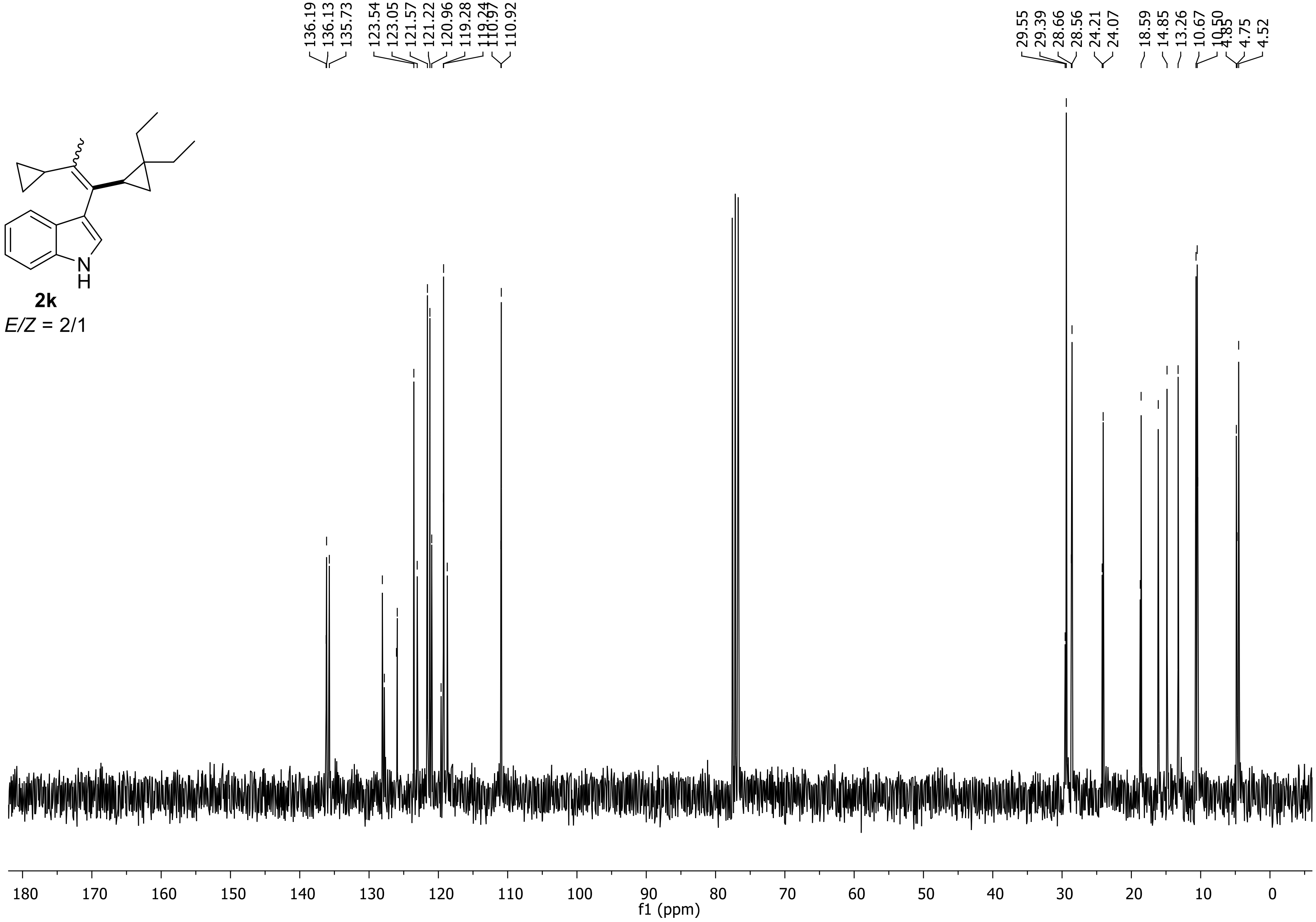

2k

E/Z = 2/1

^1H NMR ($CDCl_3$, 300 MHz)

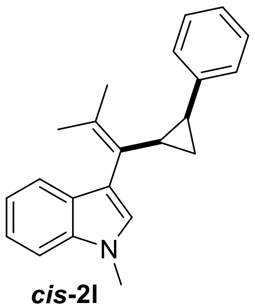

***cis*-2l**
d.r. 8/1

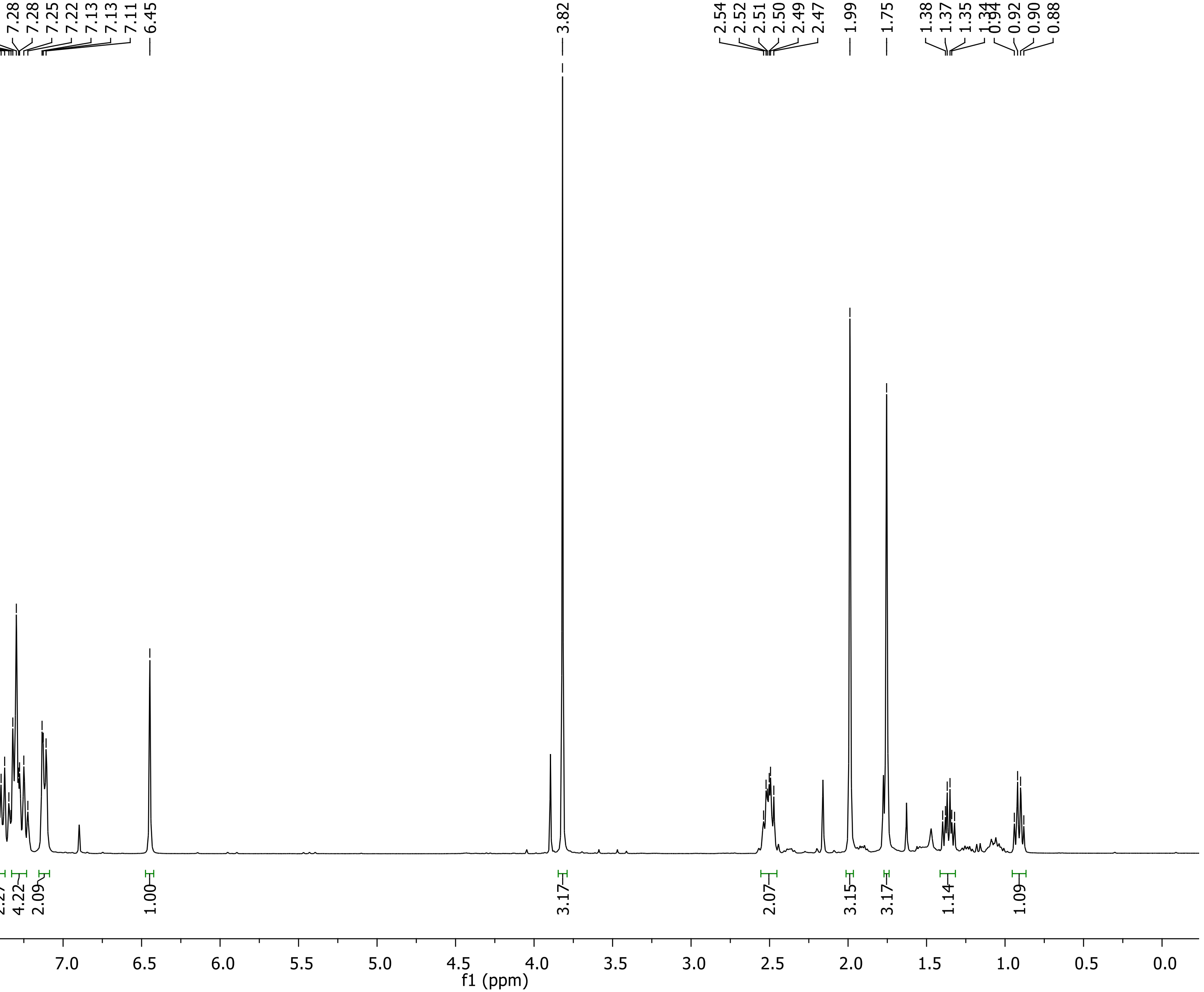

^{13}C NMR ($CDCl_3$, 75.4 MHz)

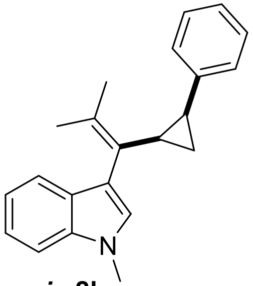

***cis*-2l**
d.r. 8/1

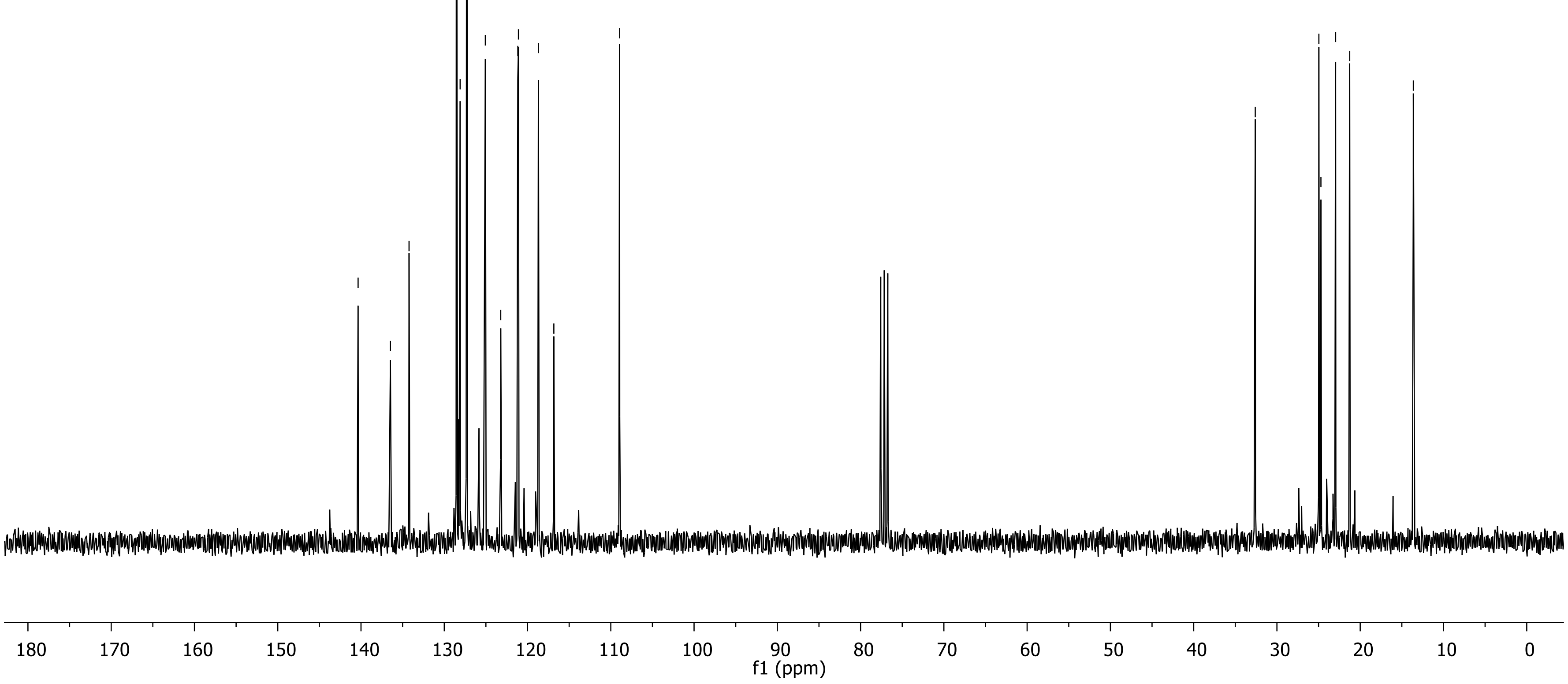

NOESY (C_6D_6, 500 MHz)

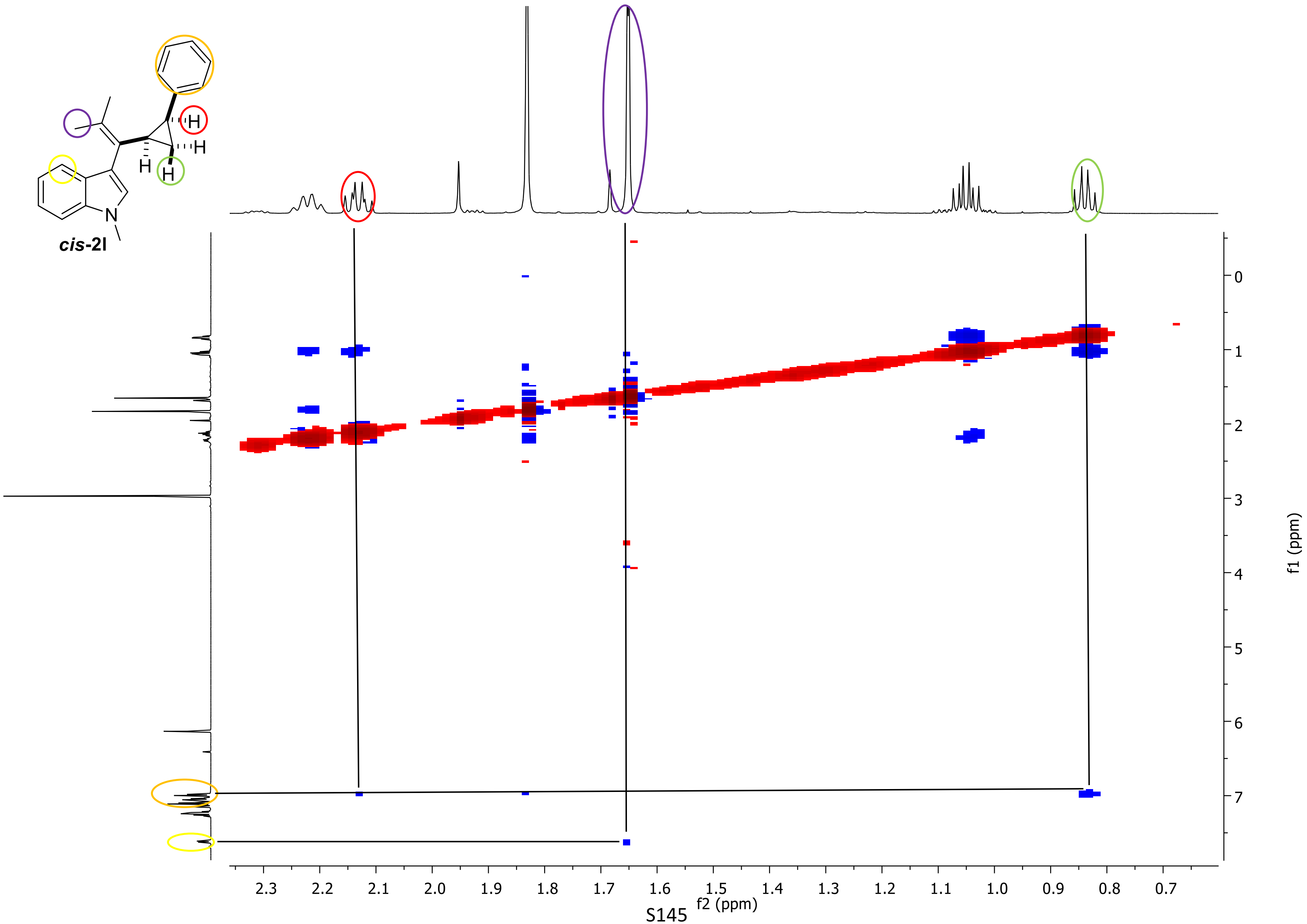

^{1}H NMR ($CDCl_3$, 300 MHz)

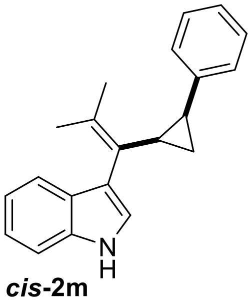

***cis*-2m**
d.r. 5/1

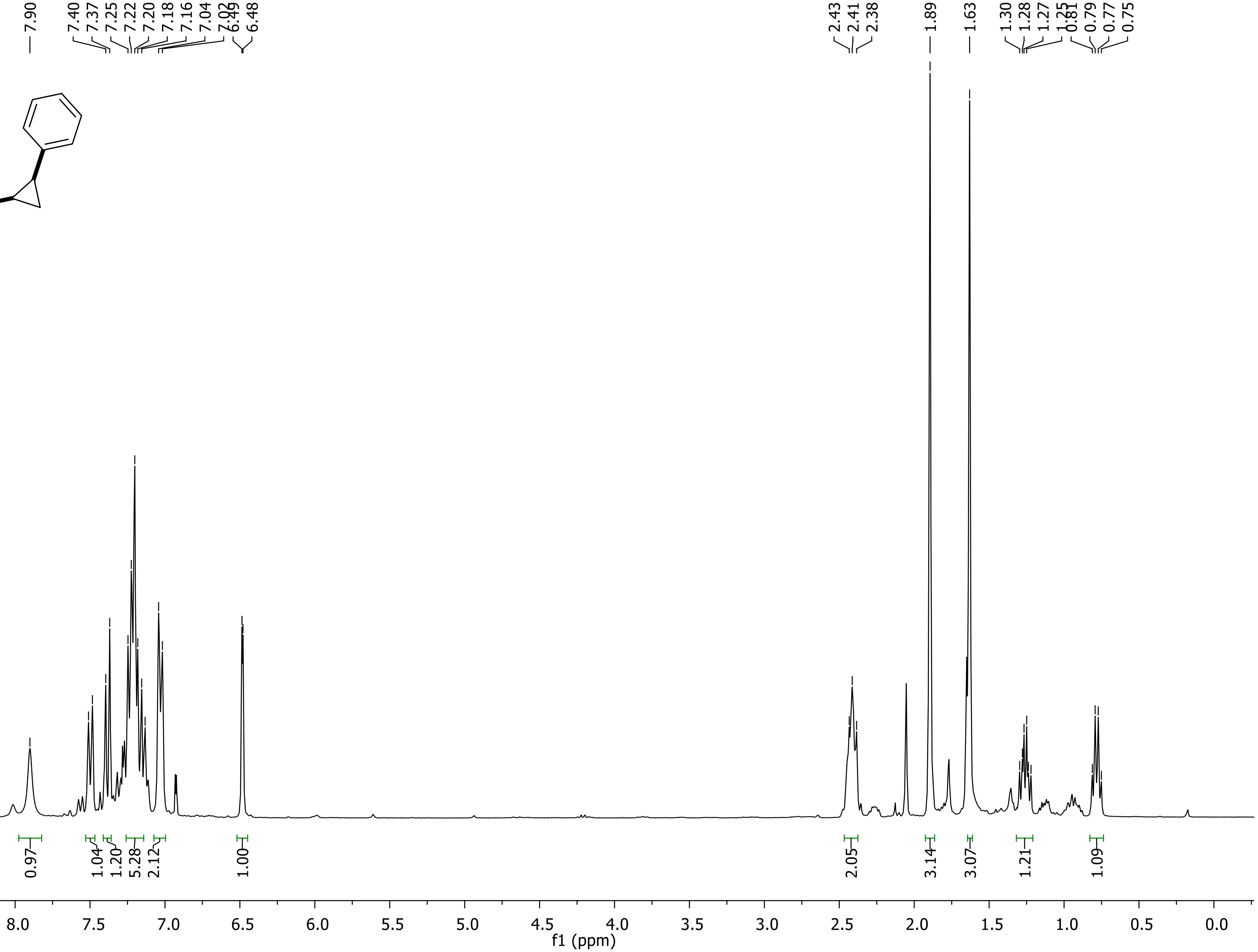

^{13}C NMR ($CDCl_3$, 75.4 MHz)

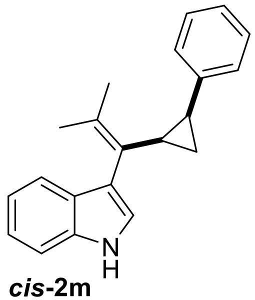

cis-**2m**
d.r. 5/1

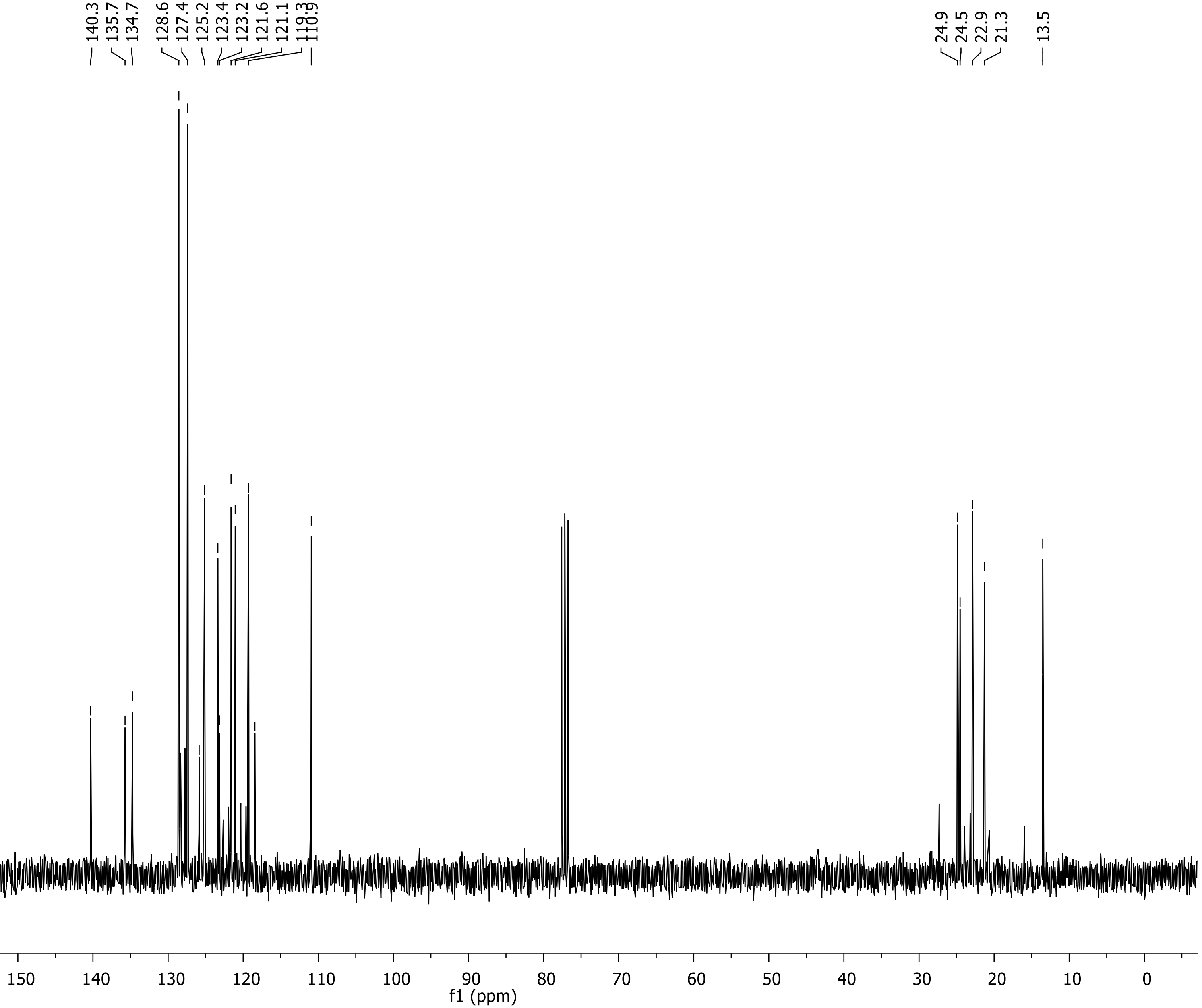

^{1}H NMR ($CDCl_3$, 300 MHz)

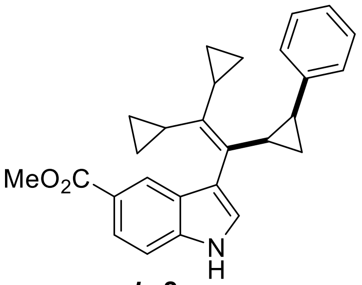

cis-**2n**

d.r. >20/1

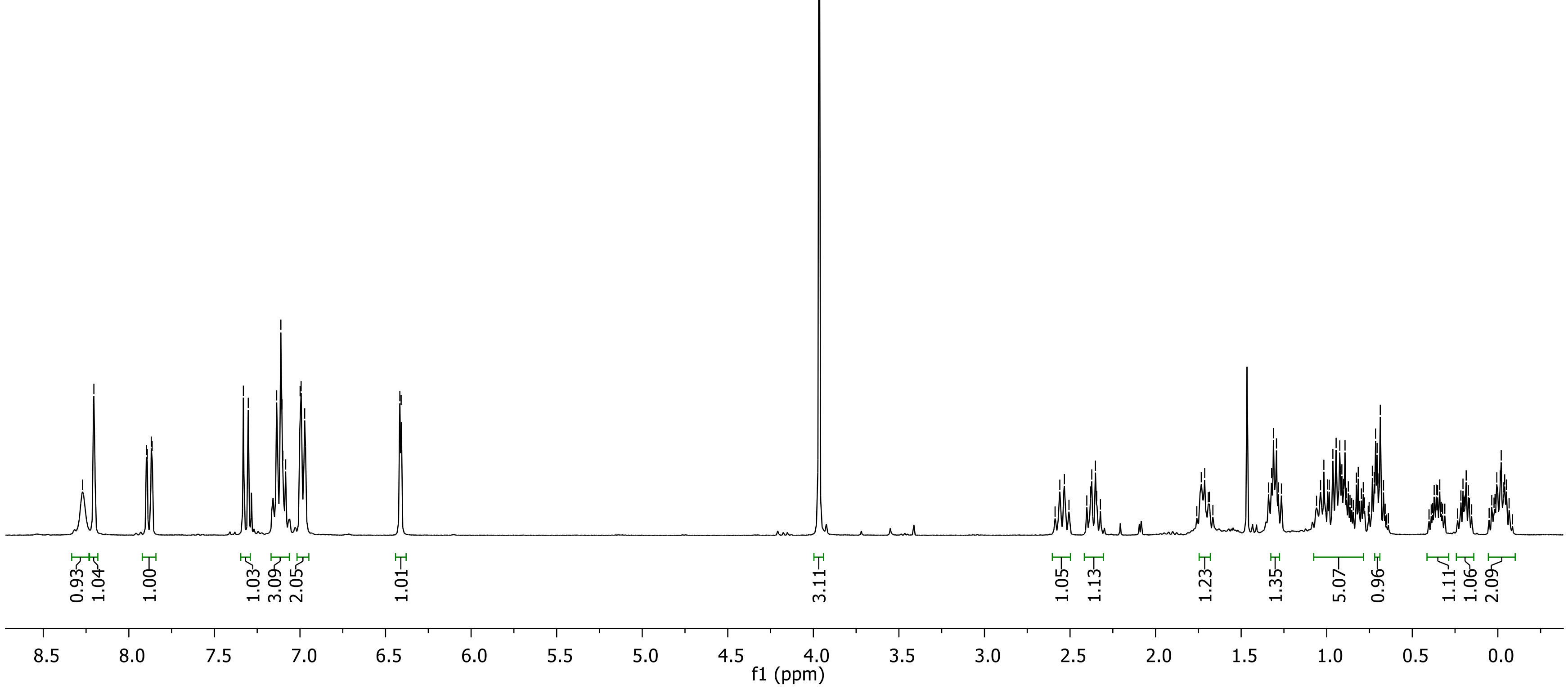

^{13}C NMR ($CDCl_3$, 75.4 MHz)

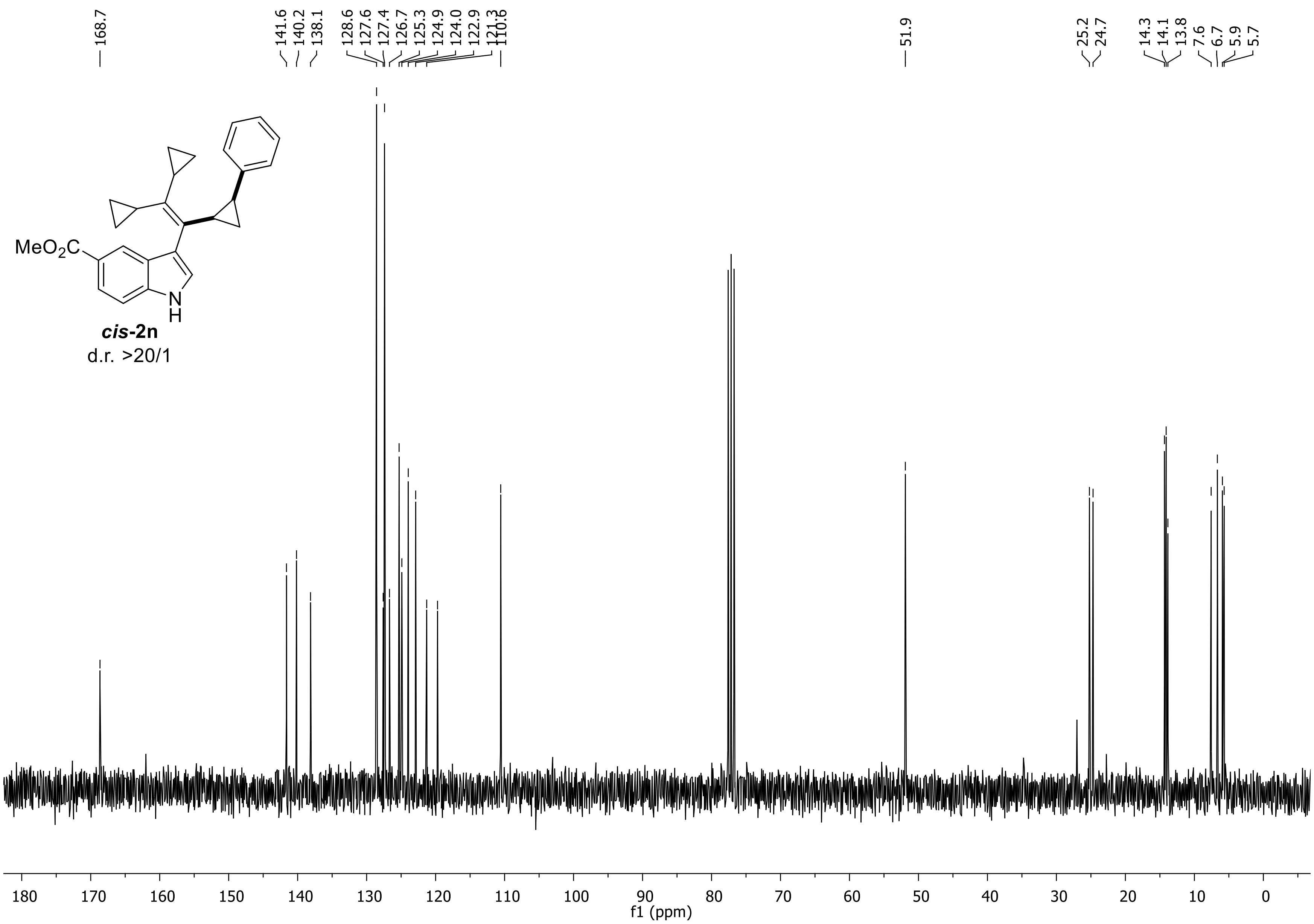

^{1}H NMR ($CDCl_3$, 300 MHz)

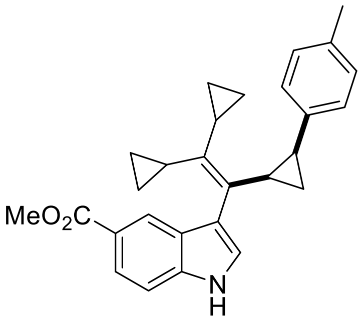

***cis*-2o**
d.r. 10/1

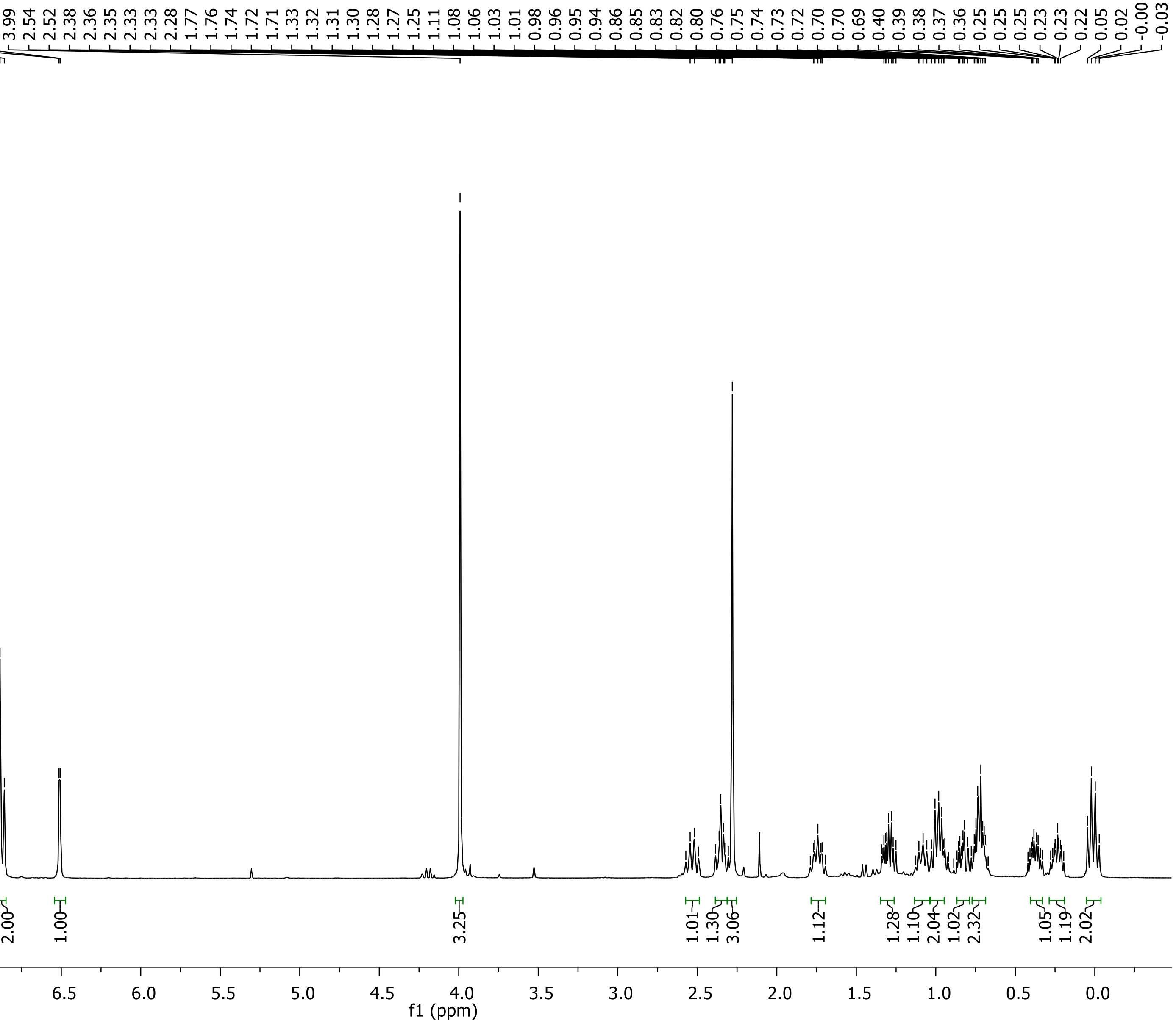

^{13}C NMR ($CDCl_3$, 75.4 MHz)

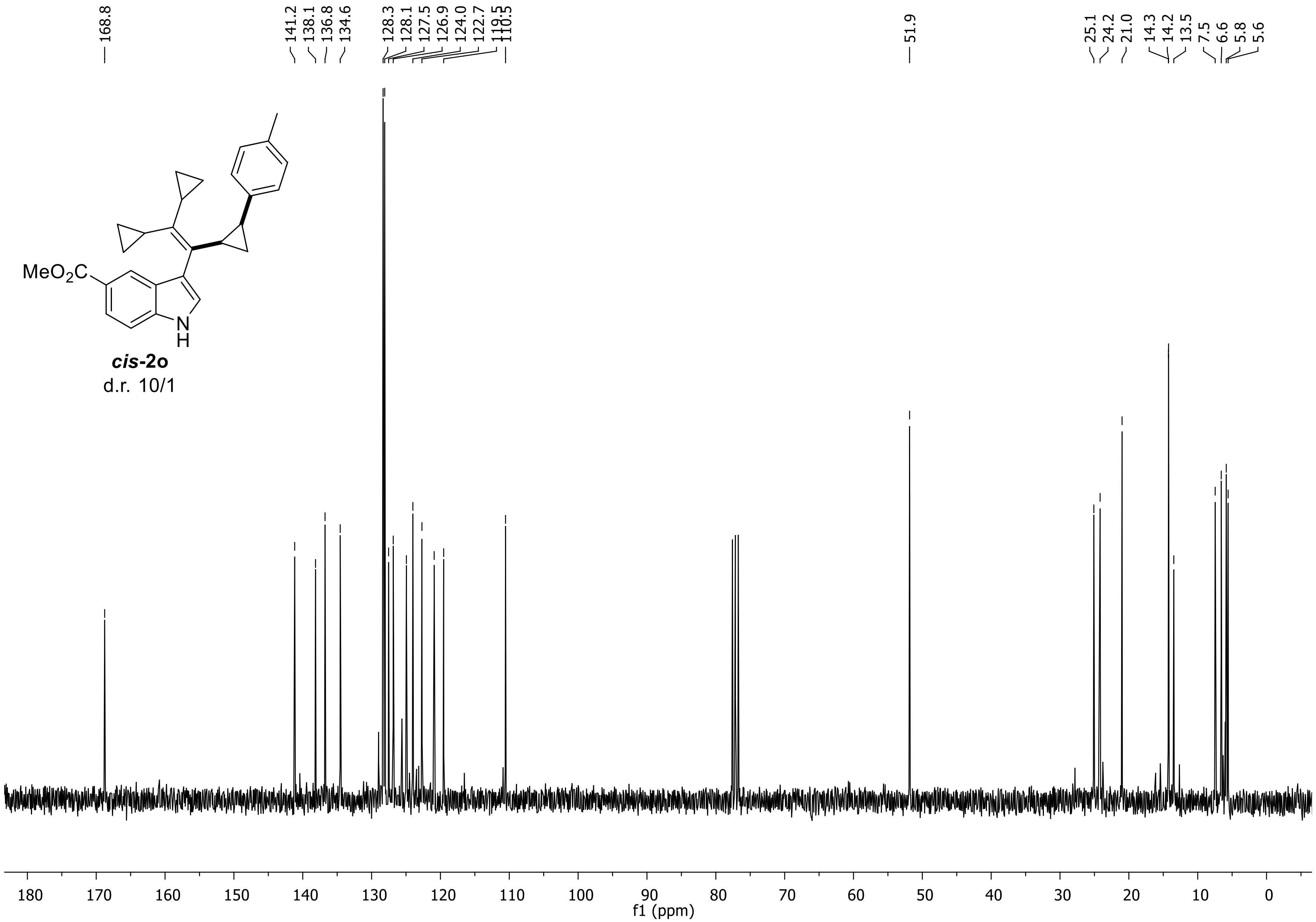

^{1}H NMR ($CDCl_3$, 300 MHz)

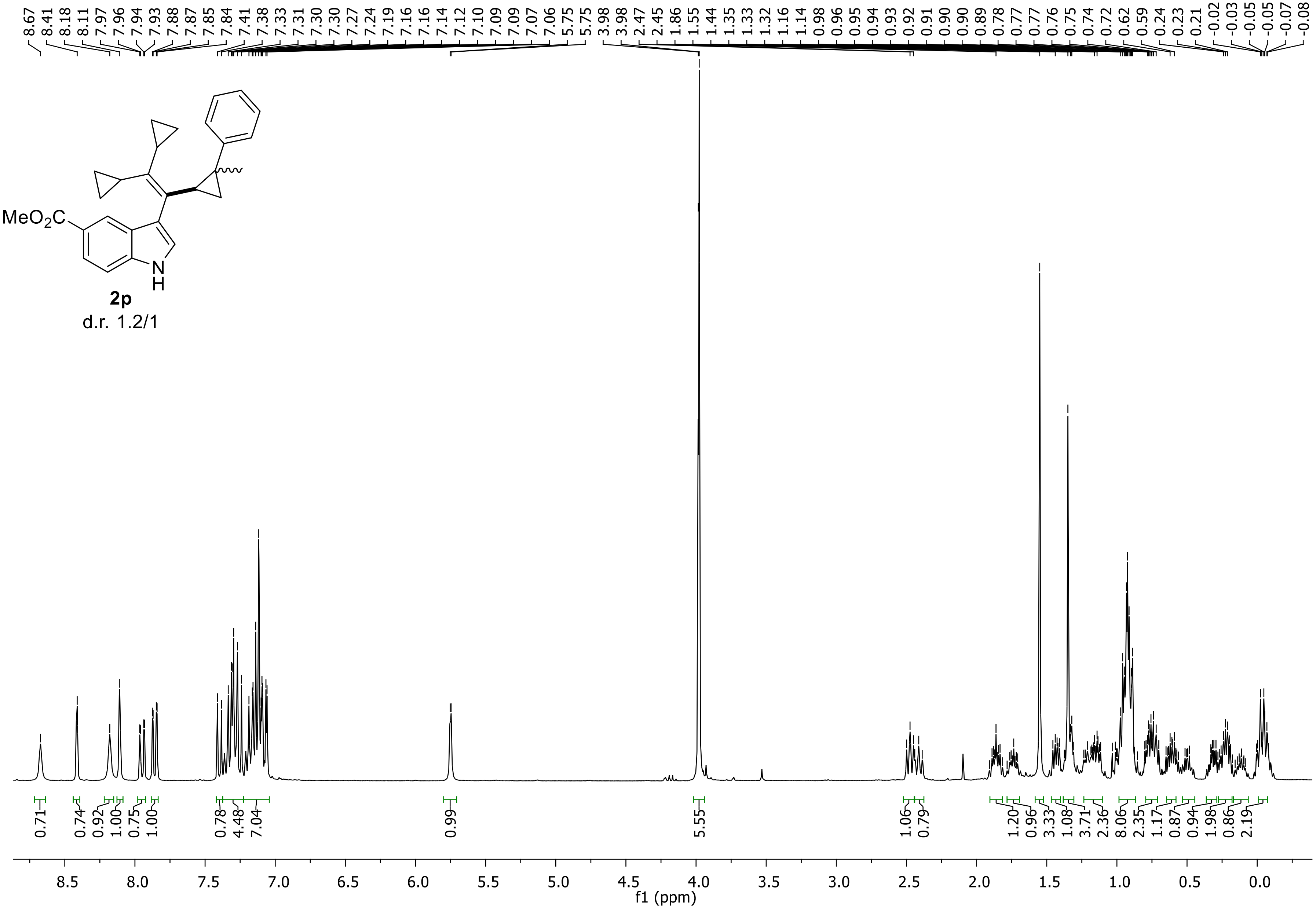

^{13}C NMR ($CDCl_3$, 75.4 MHz)

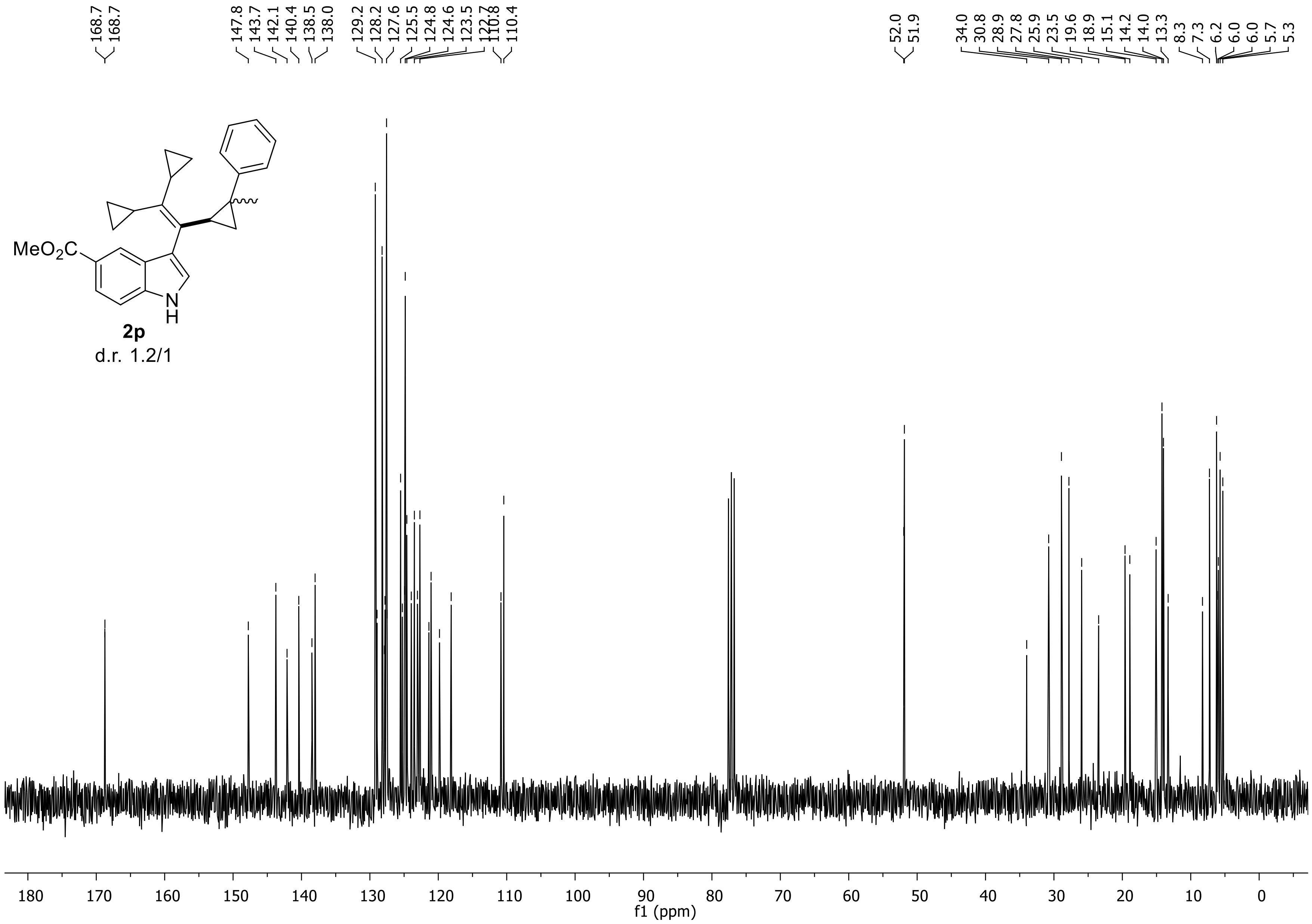

^{1}H NMR ($CDCl_3$, 300 MHz)

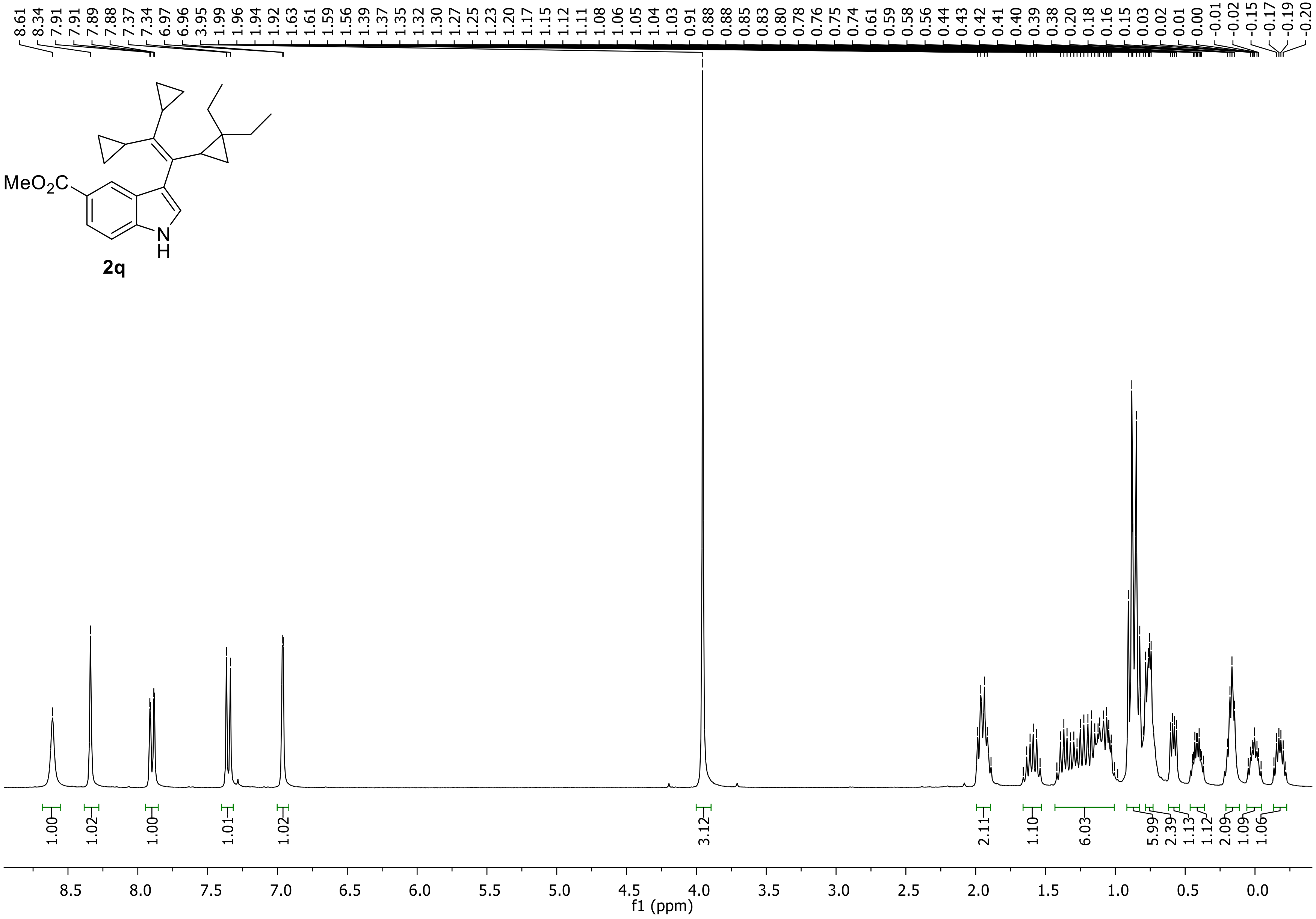

2q

^{13}C NMR ($CDCl_3$, 75.4 MHz)

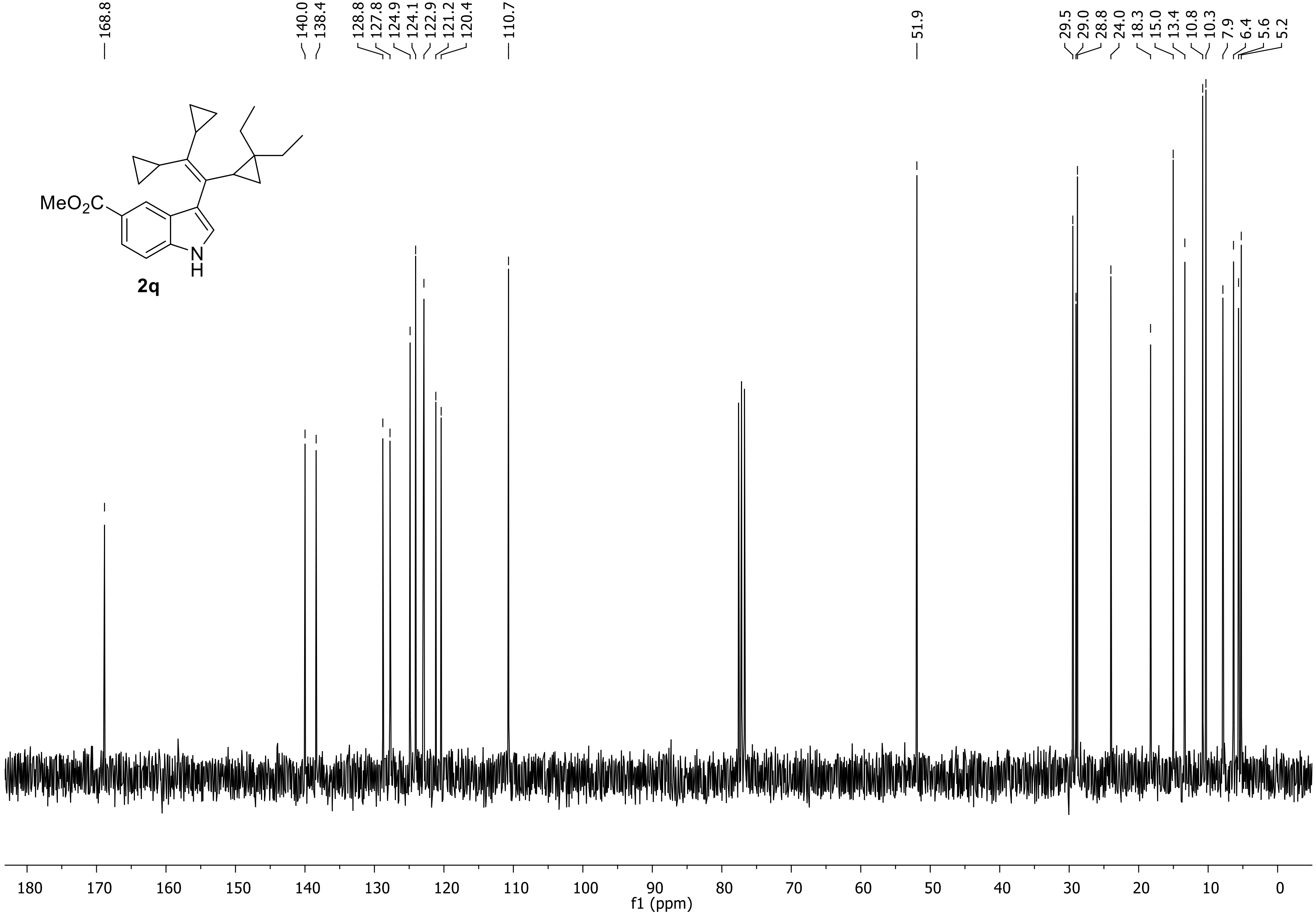

^{1}H NMR ($CDCl_3$, 300 MHz)

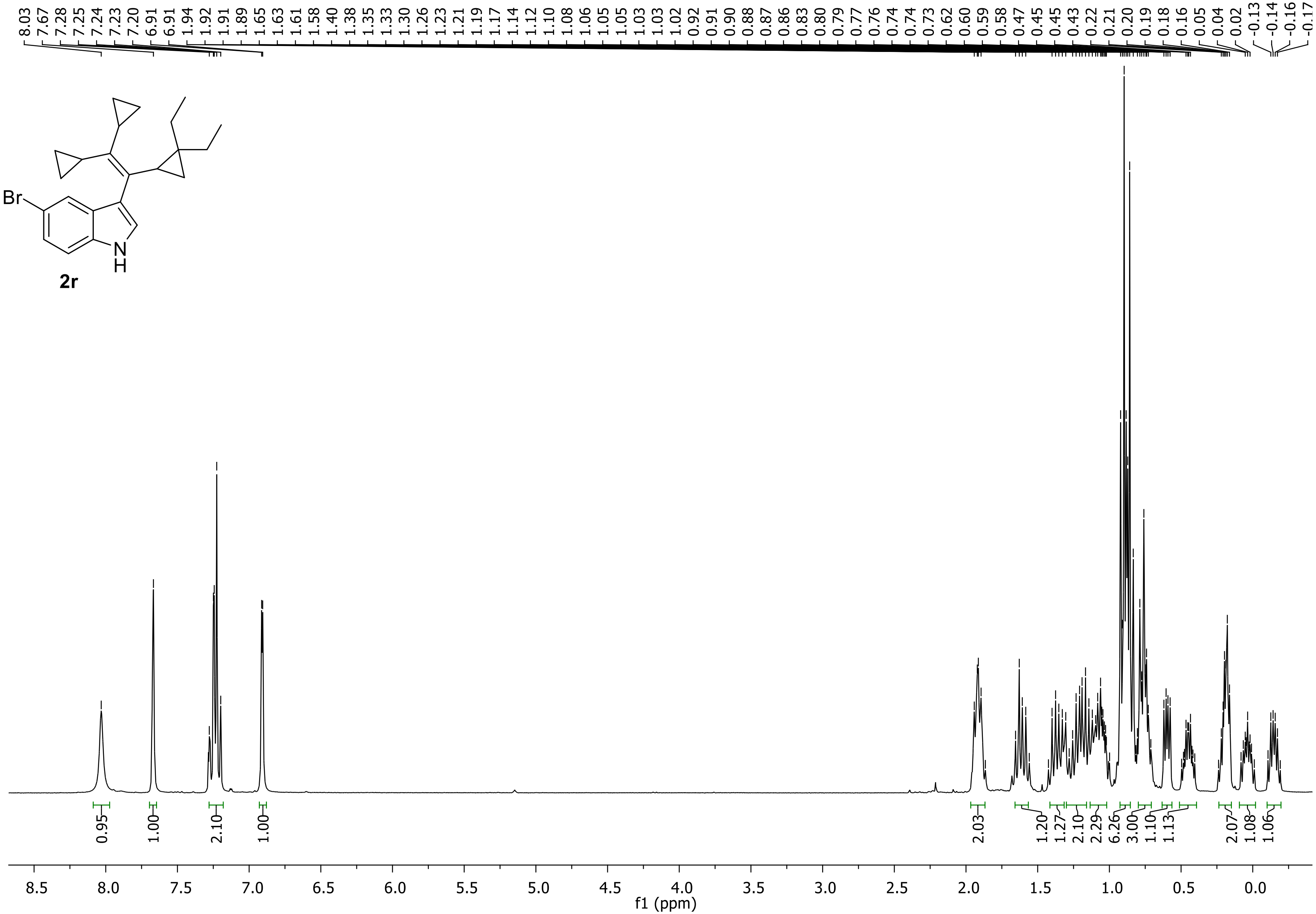

^{13}C NMR ($CDCl_3$, 75.4 MHz)

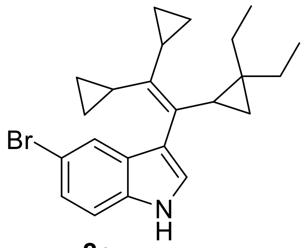

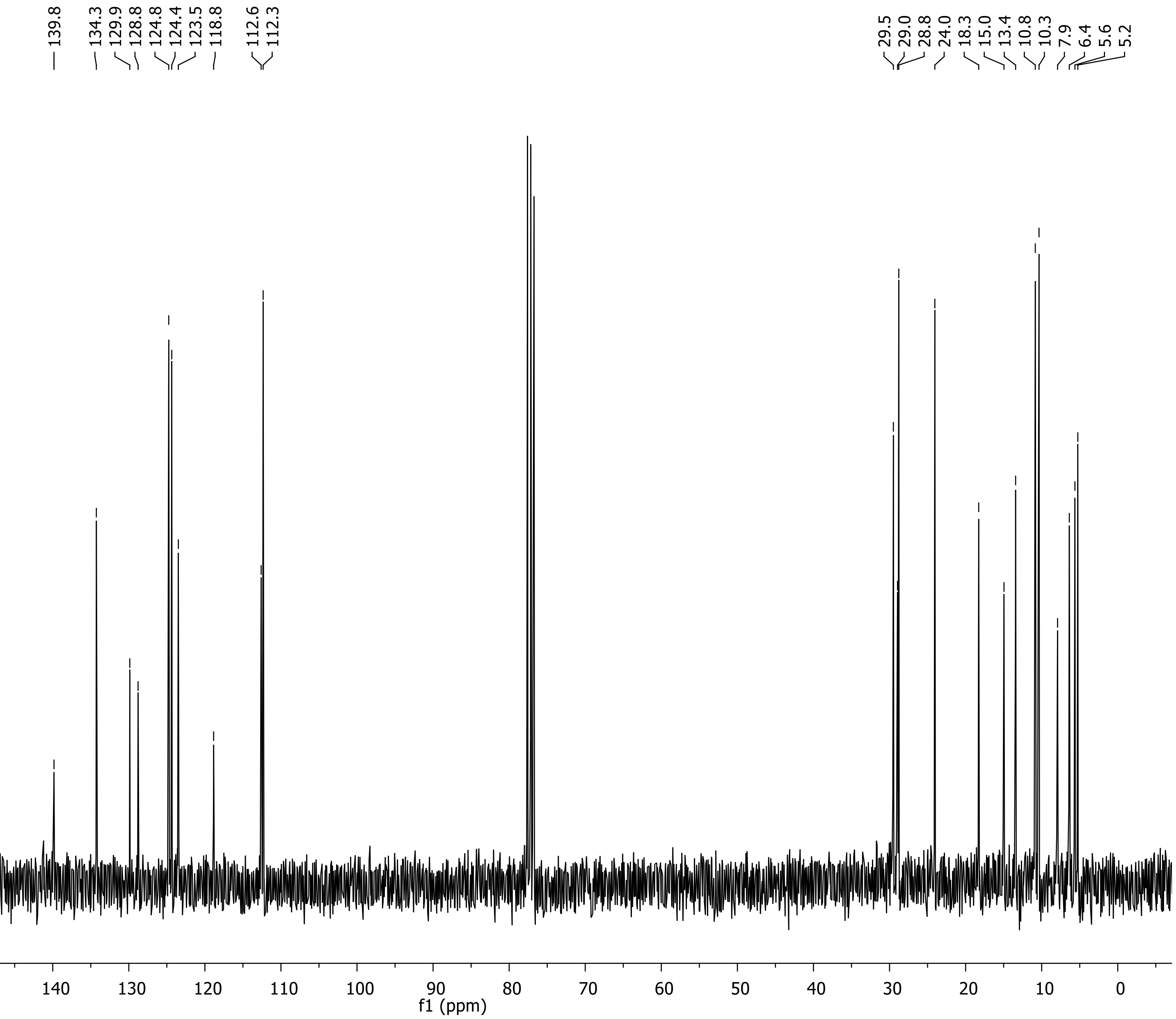

^{1}H NMR ($CDCl_3$, 300 MHz)

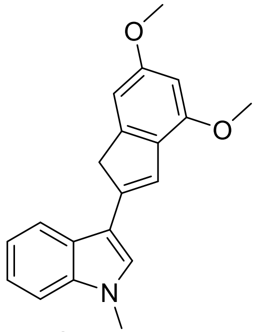

S6a

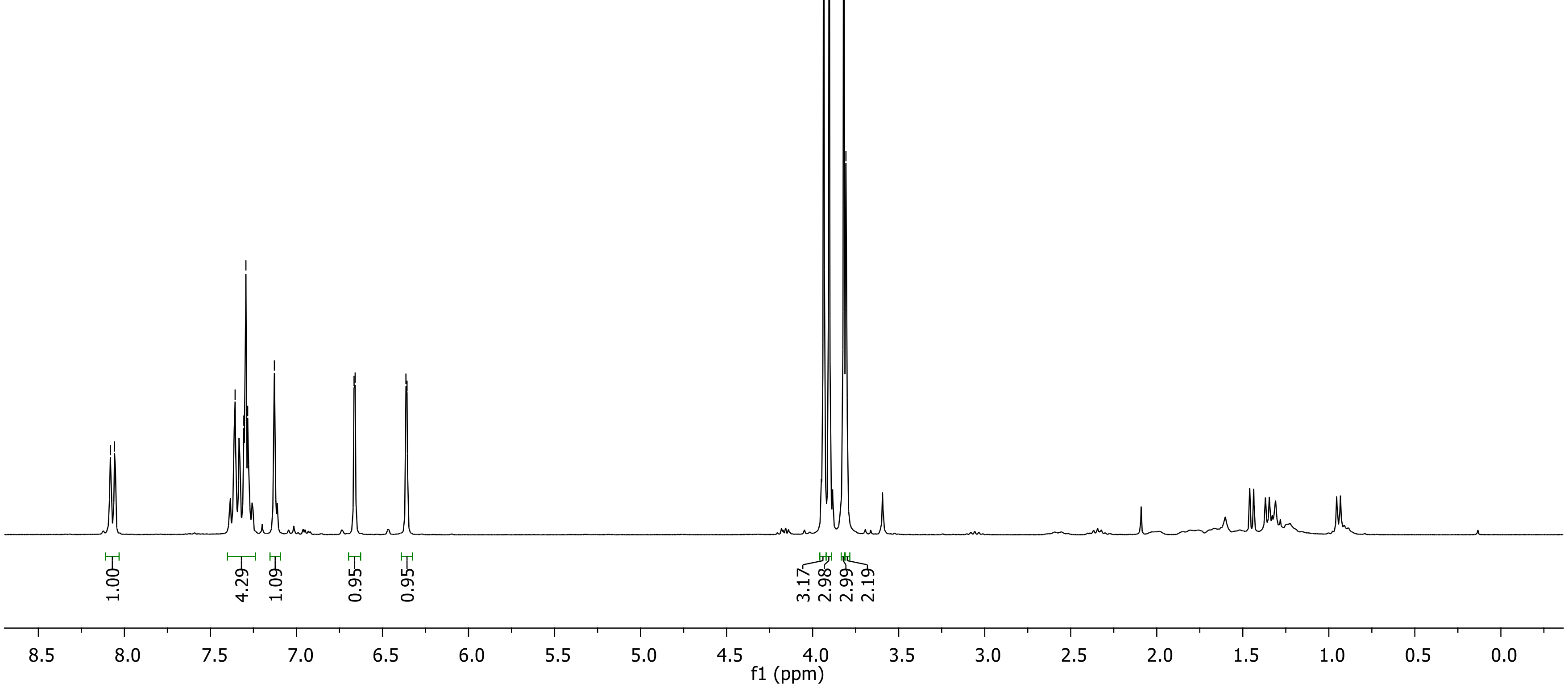

^{13}C NMR ($CDCl_3$, 75.4 MHz)

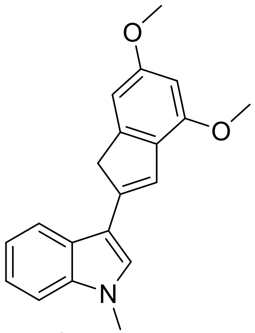

S6a

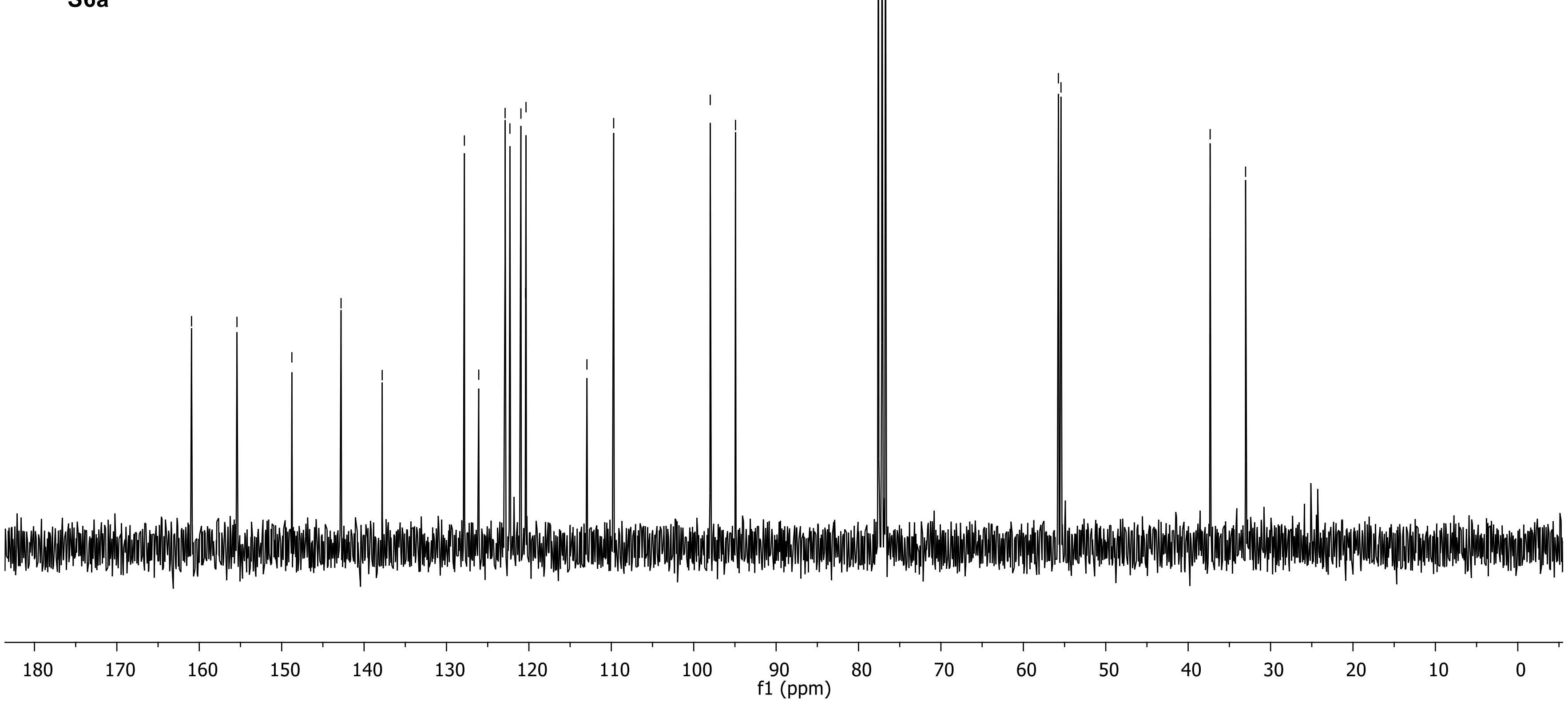

^{1}H NMR ($CDCl_3$, 300 MHz)

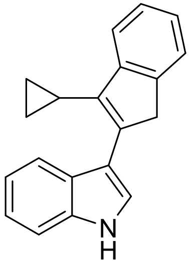

S6b

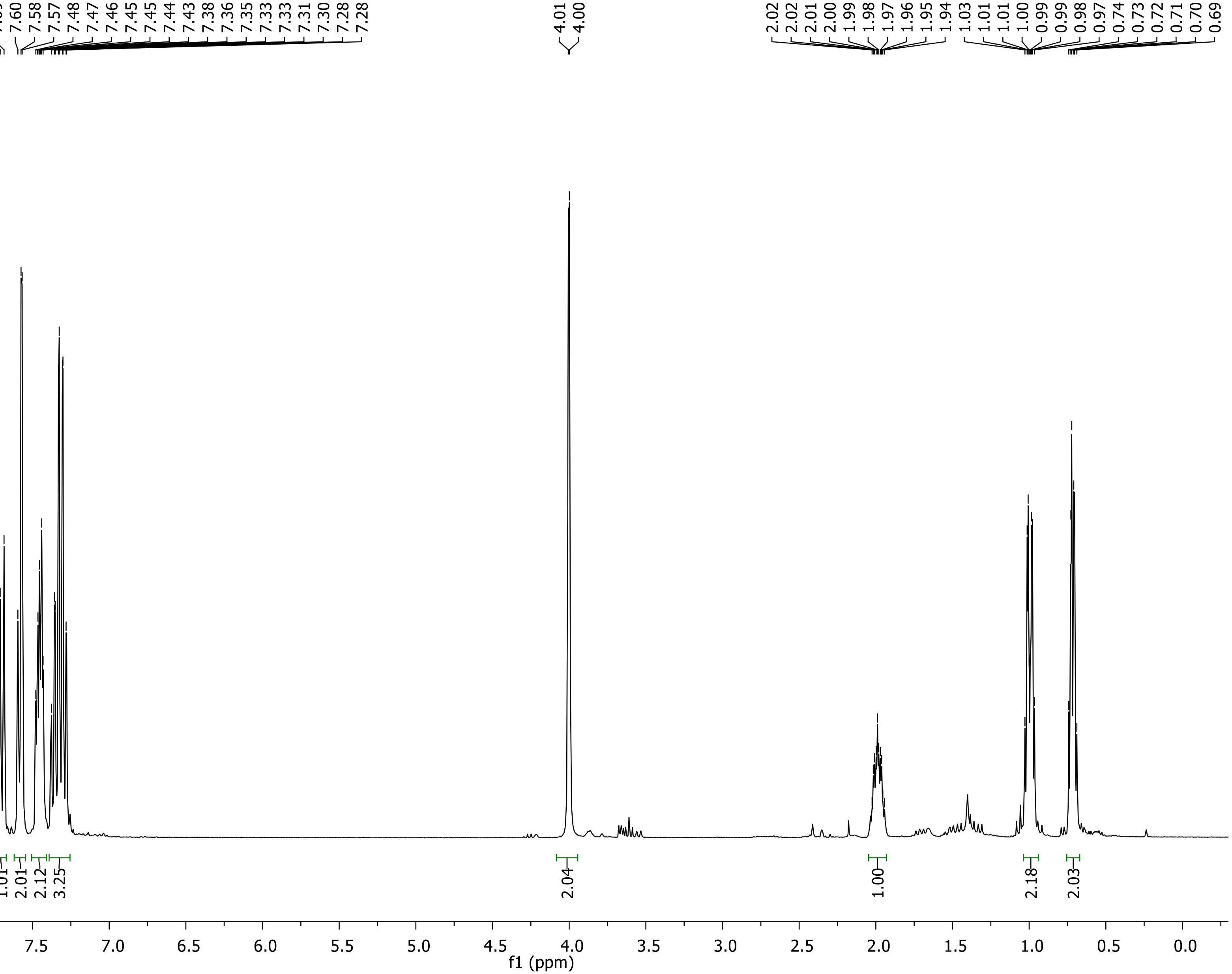

^{13}C NMR ($CDCl_3$, 75.4 MHz)

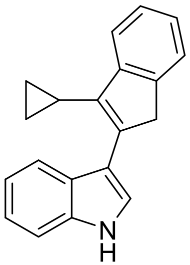

S6b

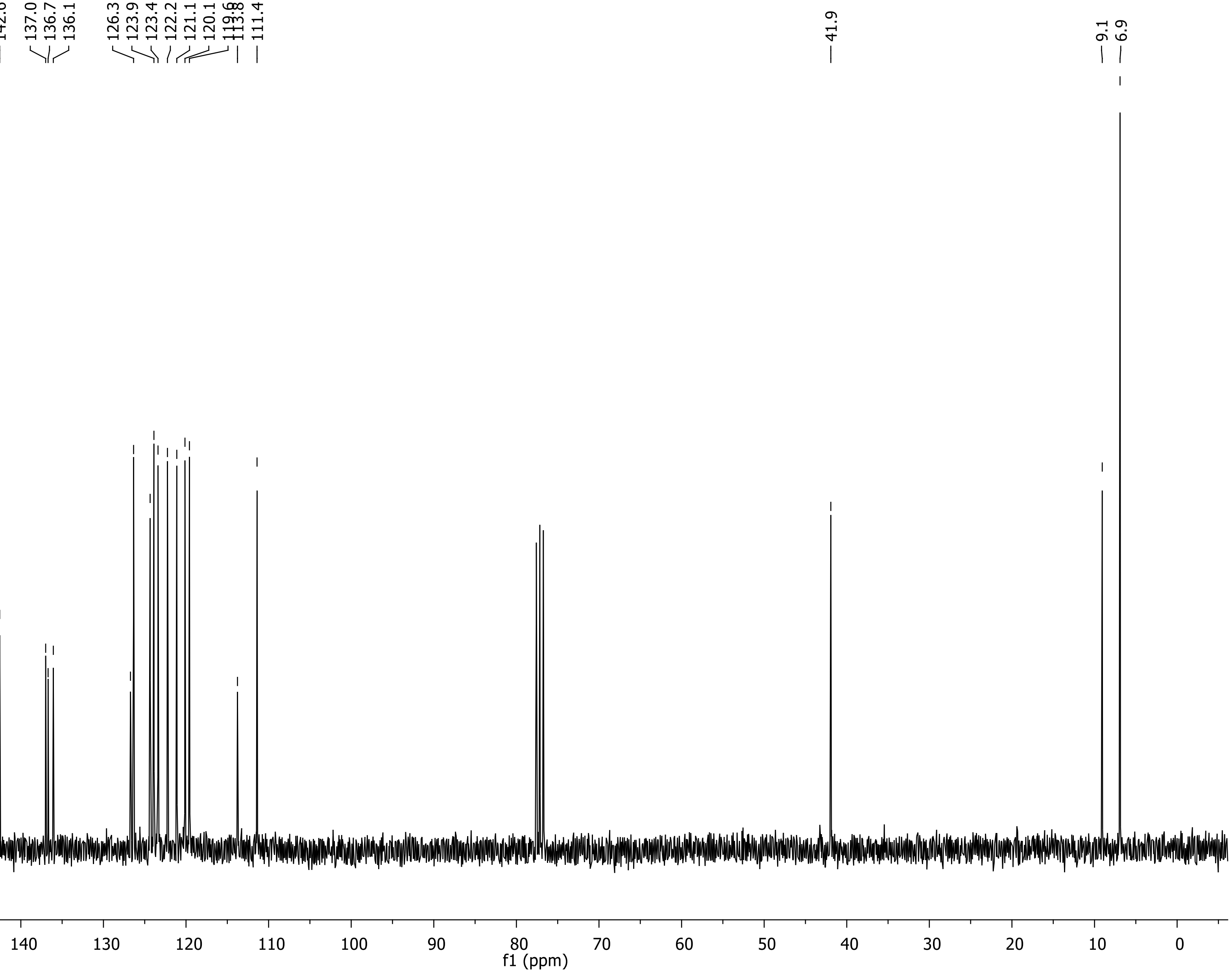

^{1}H NMR ($CDCl_3$, 300 MHz)

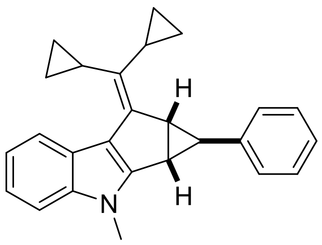

***cis*-4a**

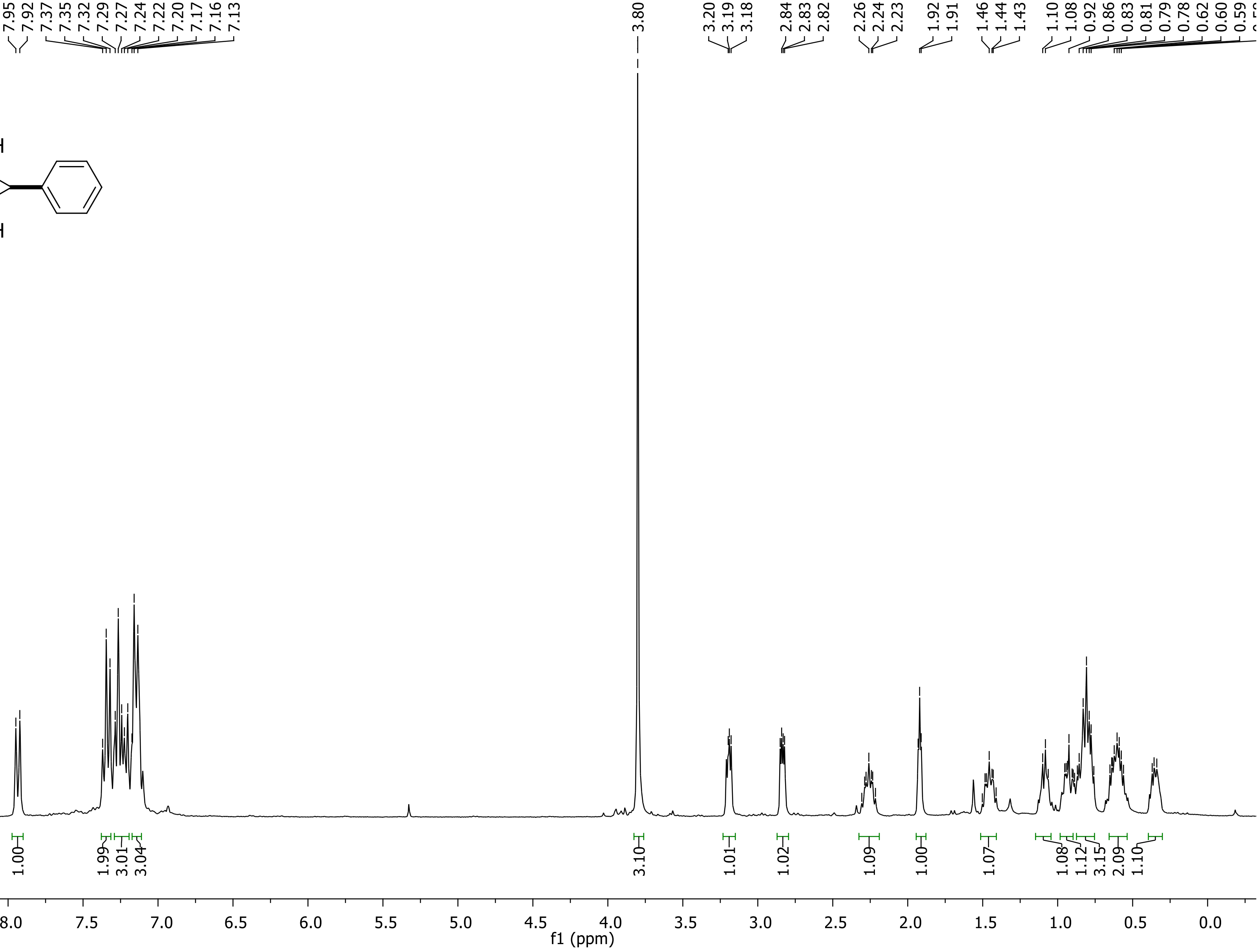

^{13}C NMR ($CDCl_3$, 75.4 MHz)

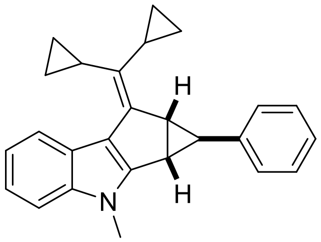

cis-**4a**

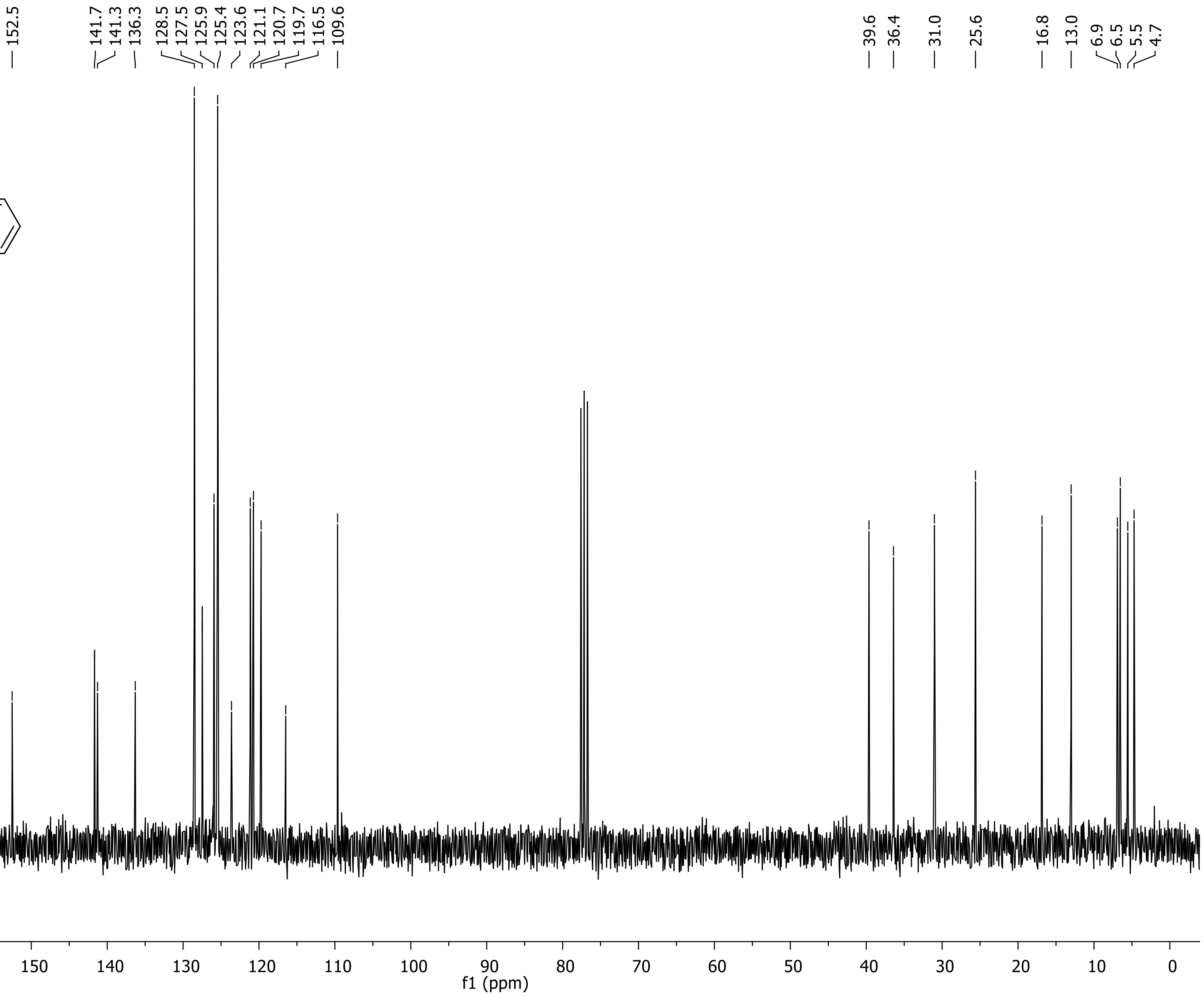

NOESY ($CDCl_3$, 500 MHz)

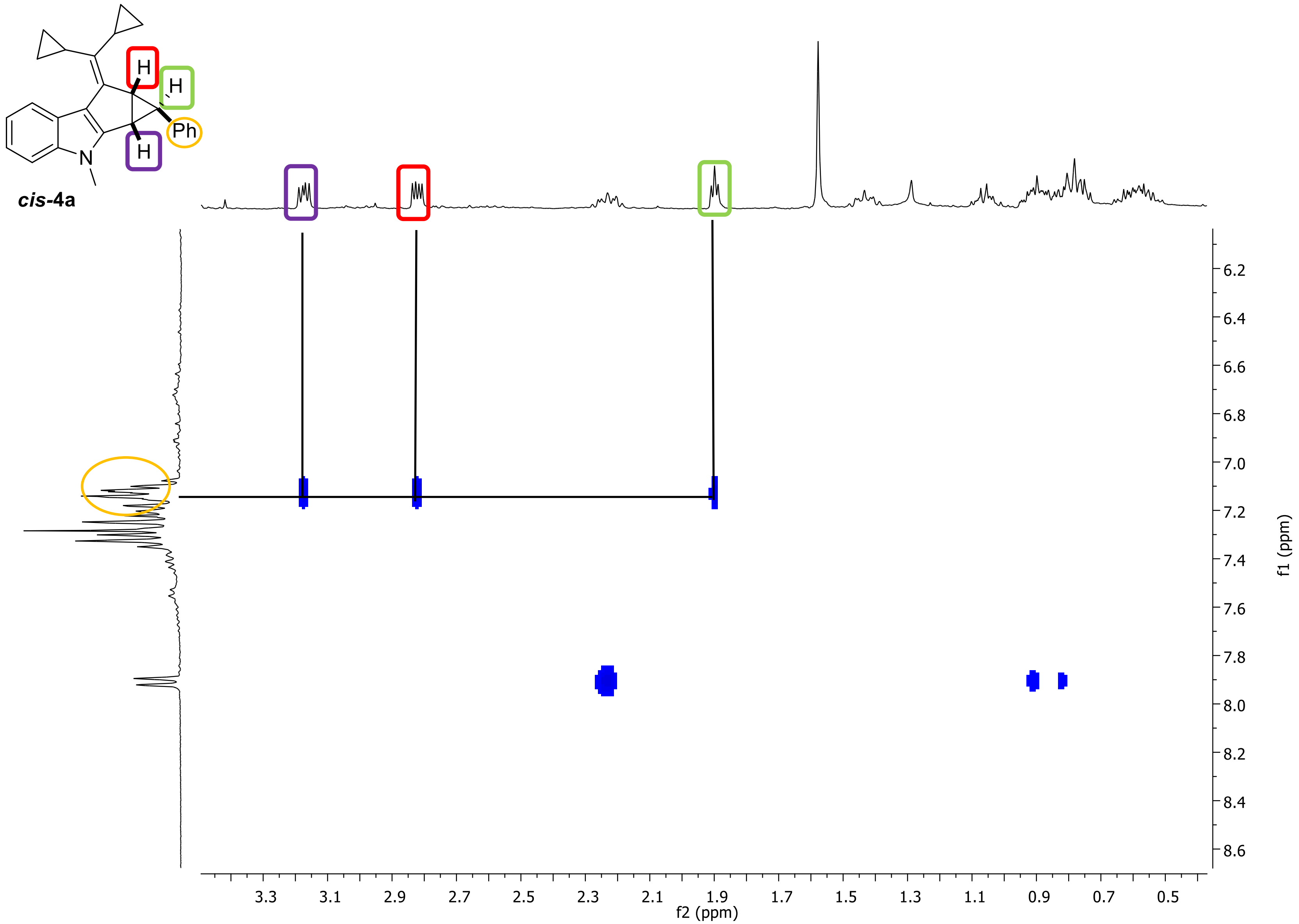

^{1}H NMR ($CDCl_3$, 300 MHz)

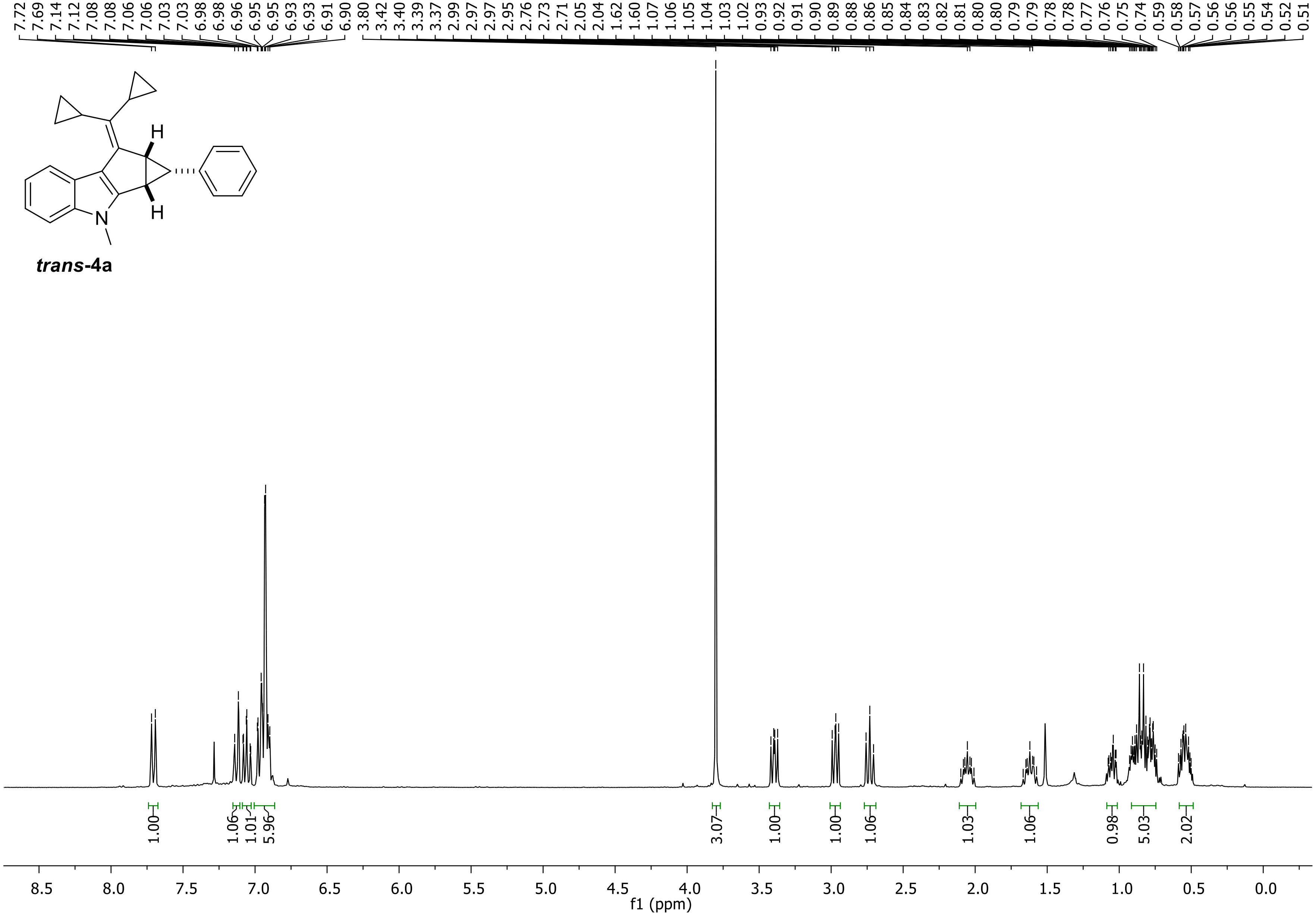

^{13}C NMR ($CDCl_3$, 125 MHz)

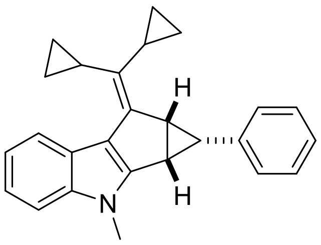

***trans*-4a**

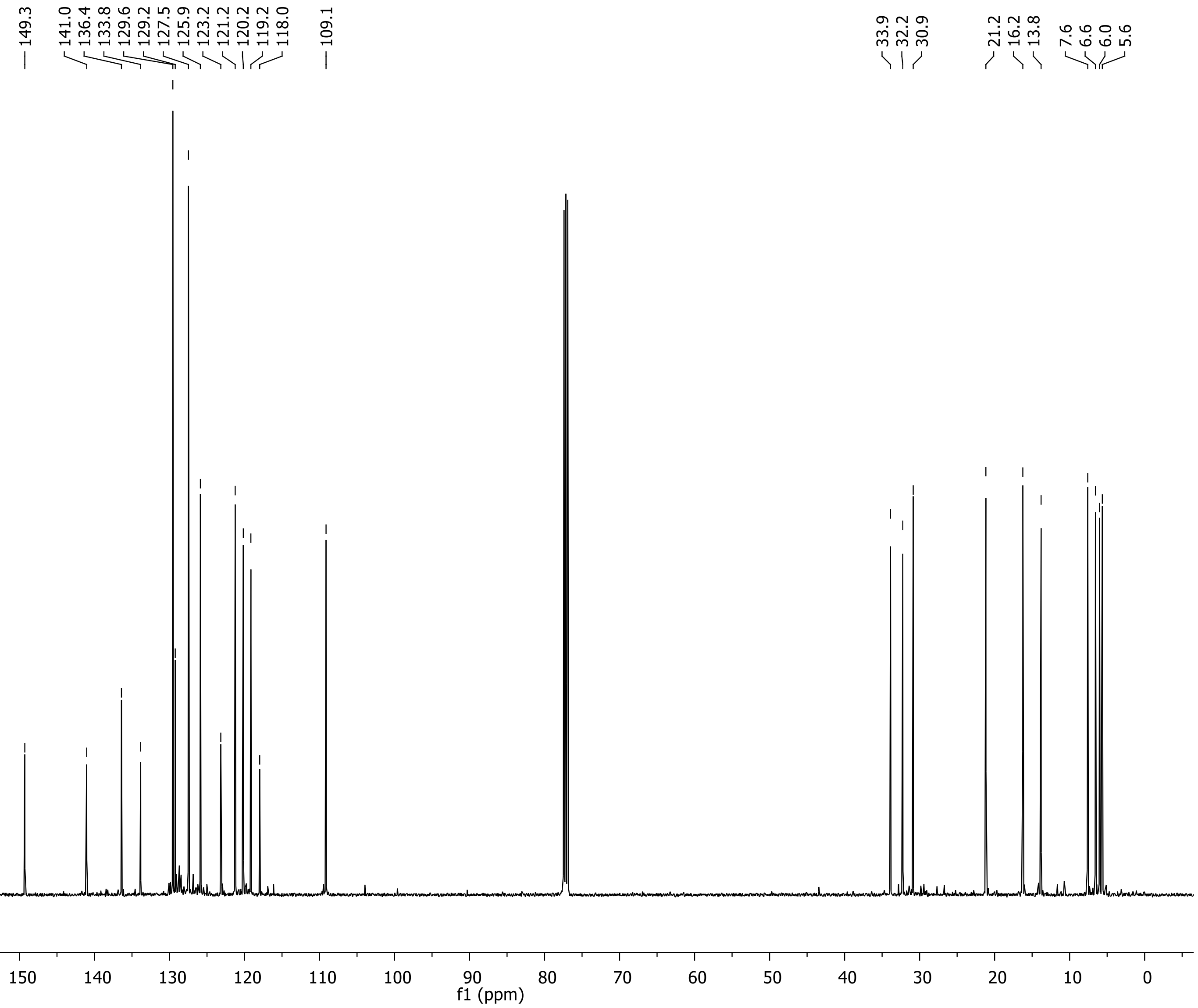

NOESY ($CDCl_3$, 500 MHz)

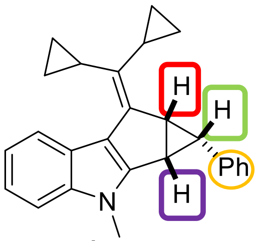

***trans*-4a**

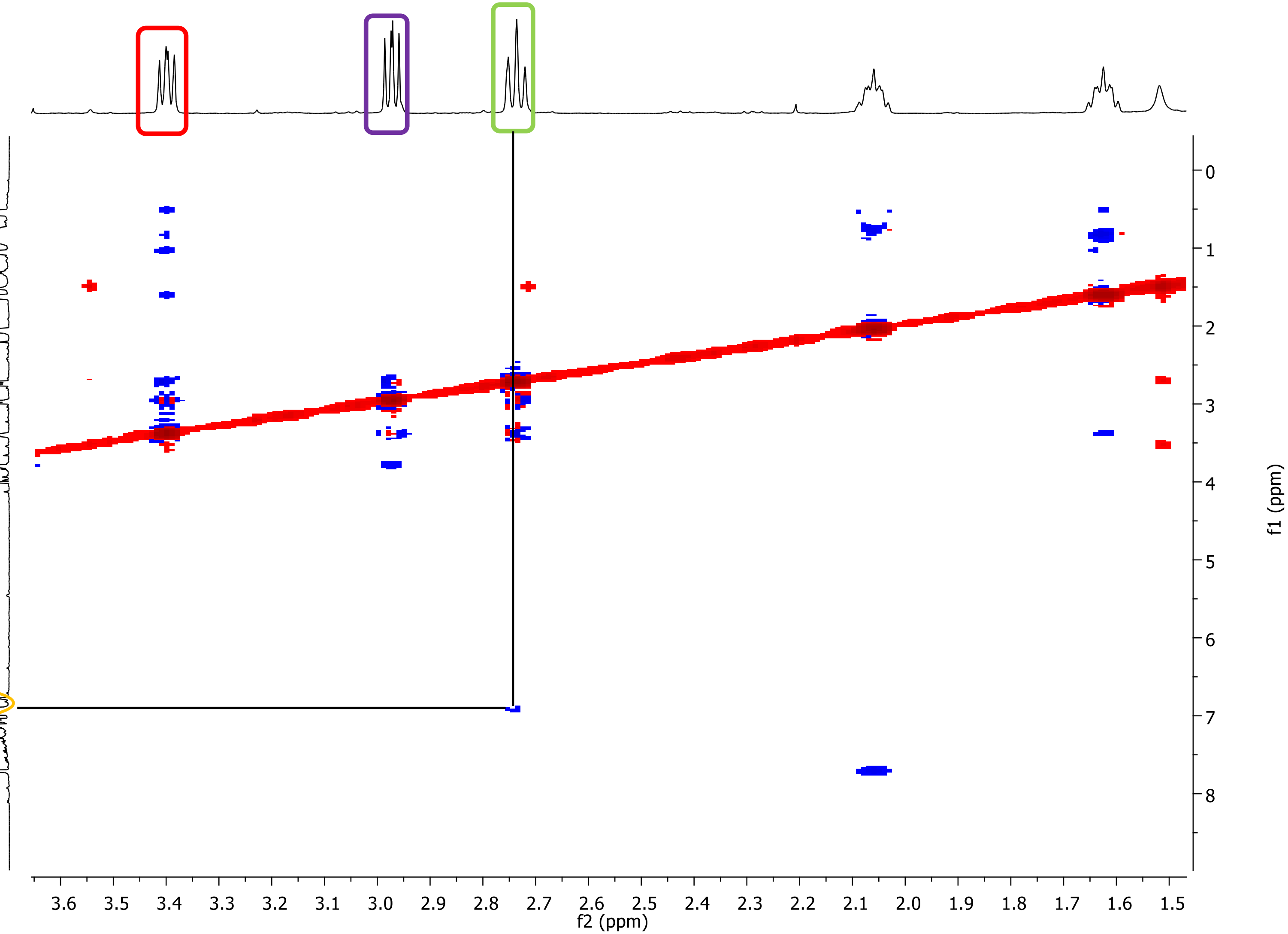

^{1}H NMR ($CDCl_3$, 300 MHz)

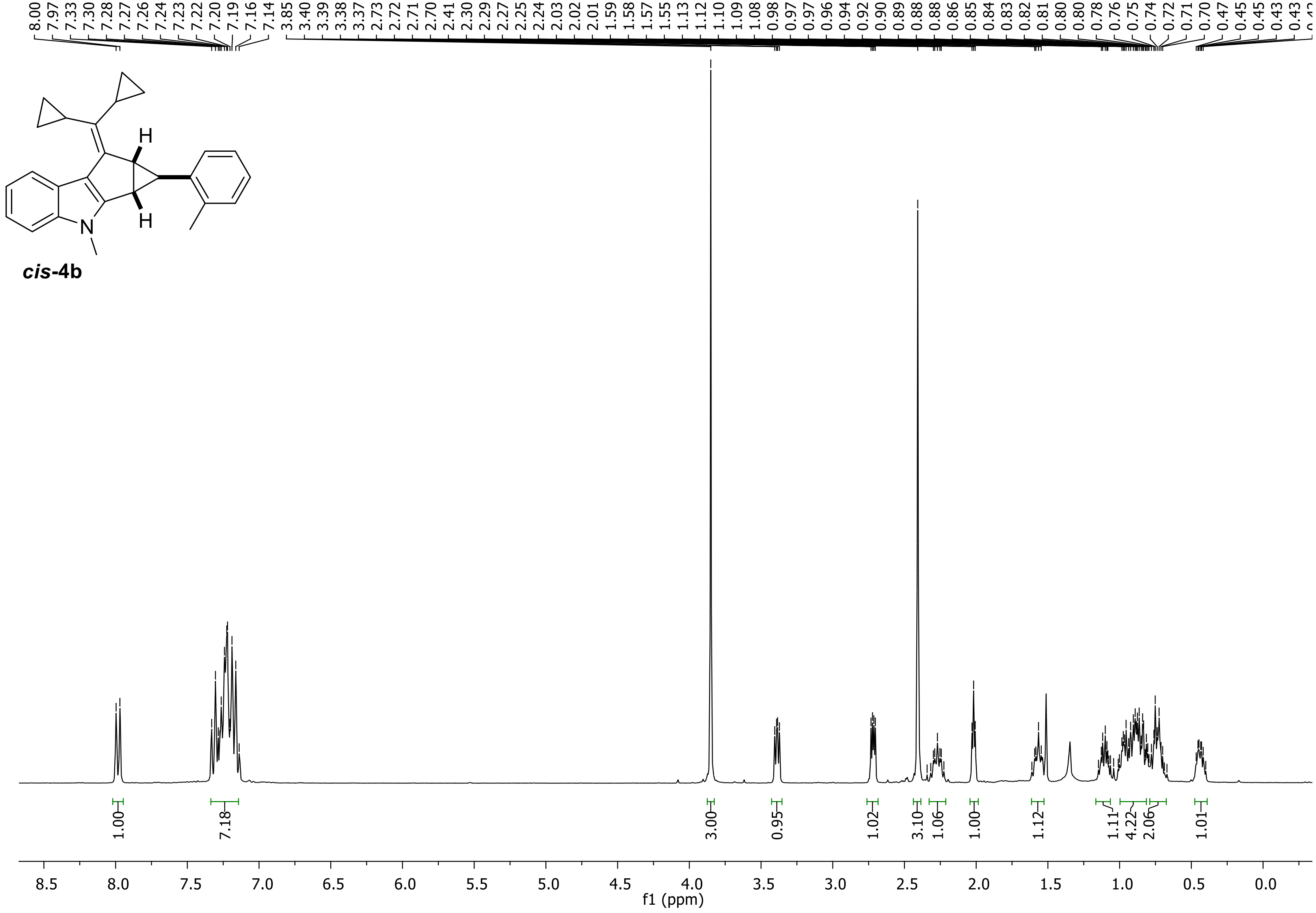

***cis*-4b**

^{13}C NMR ($CDCl_3$, 75.4 MHz)

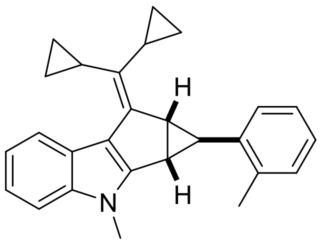

***cis*-4b**

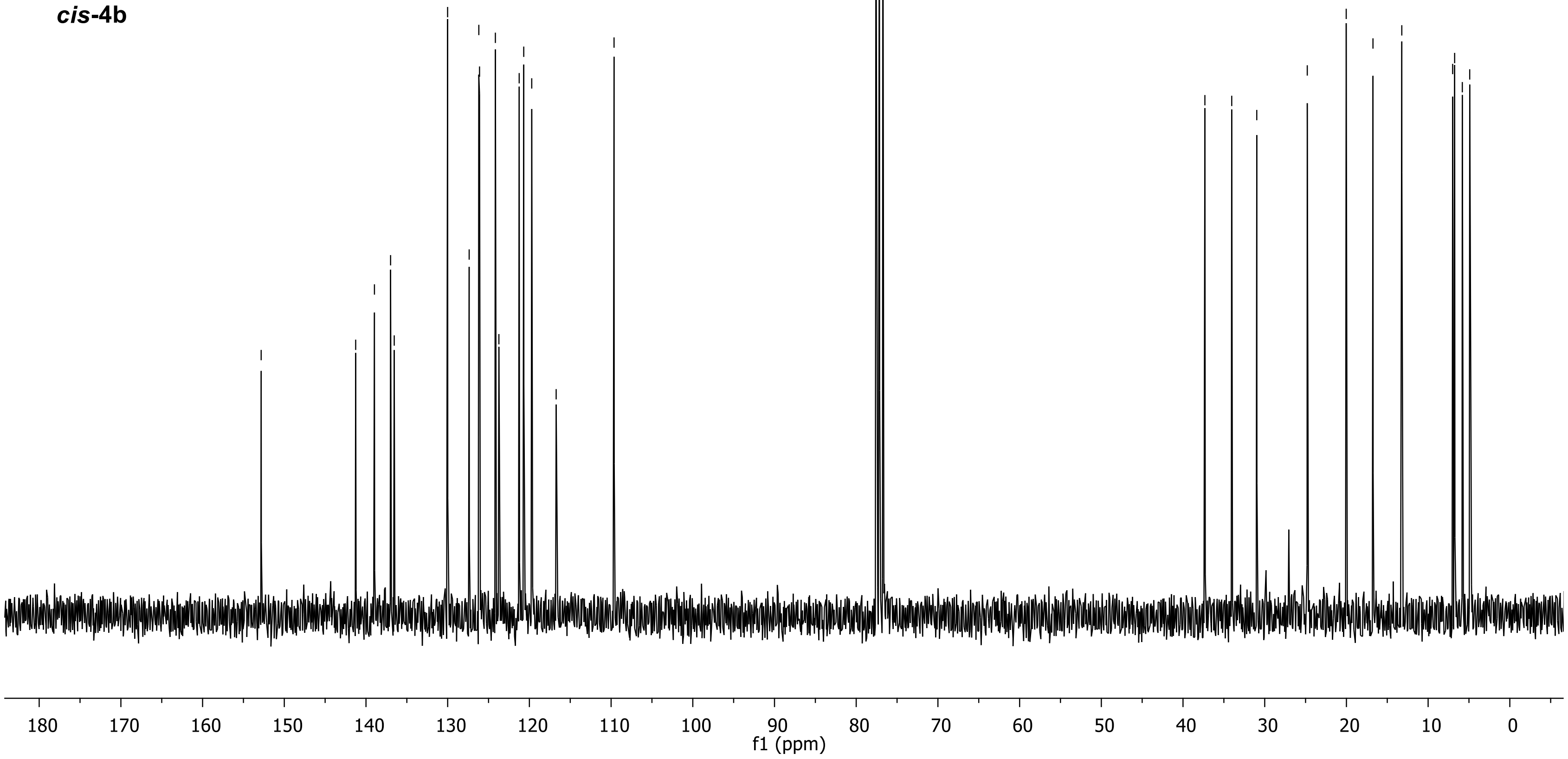

^{1}H NMR (CDCl$_3$, 300 MHz)

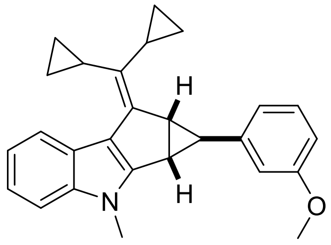

cis-**4c**

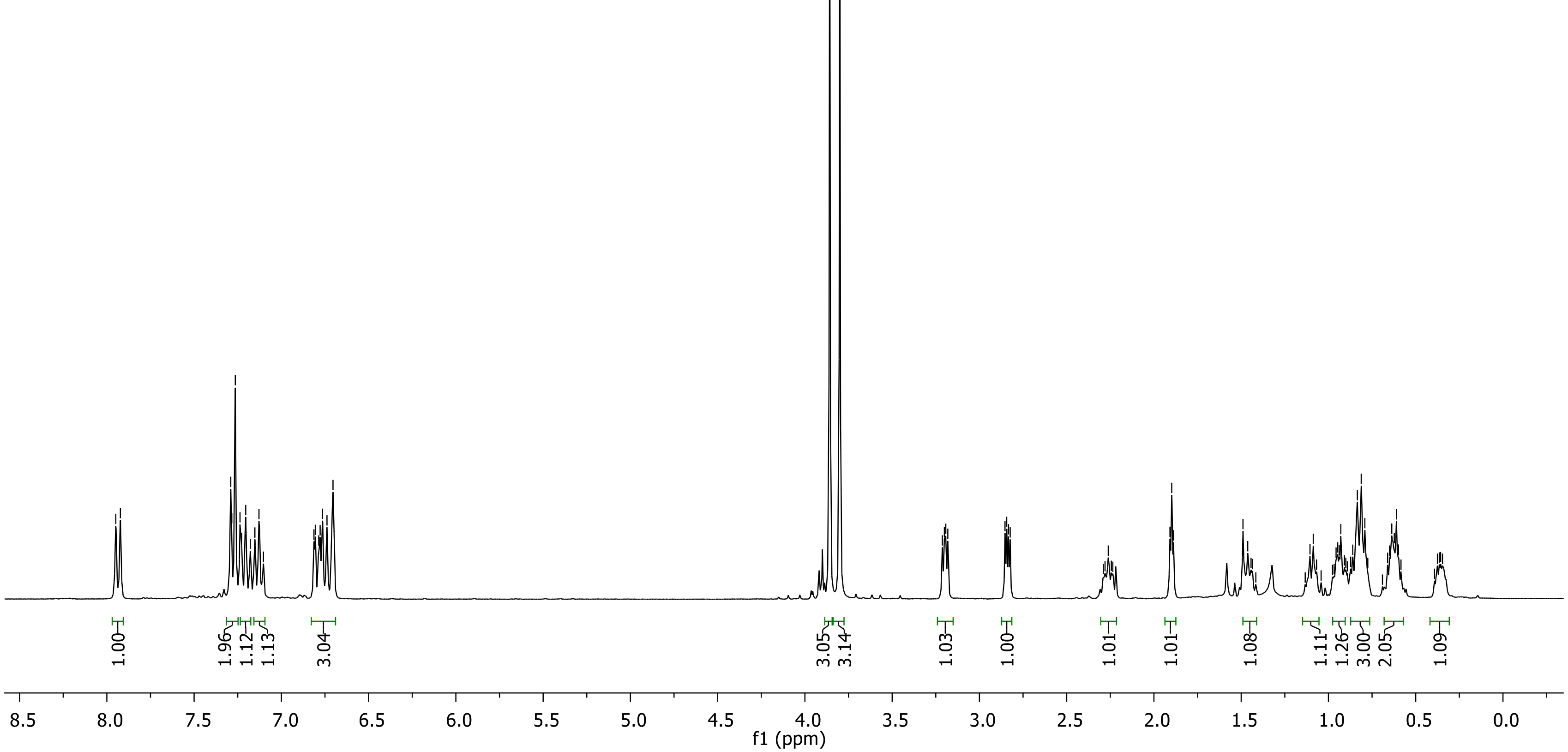

^{13}C NMR ($CDCl_3$, 75.4 MHz)

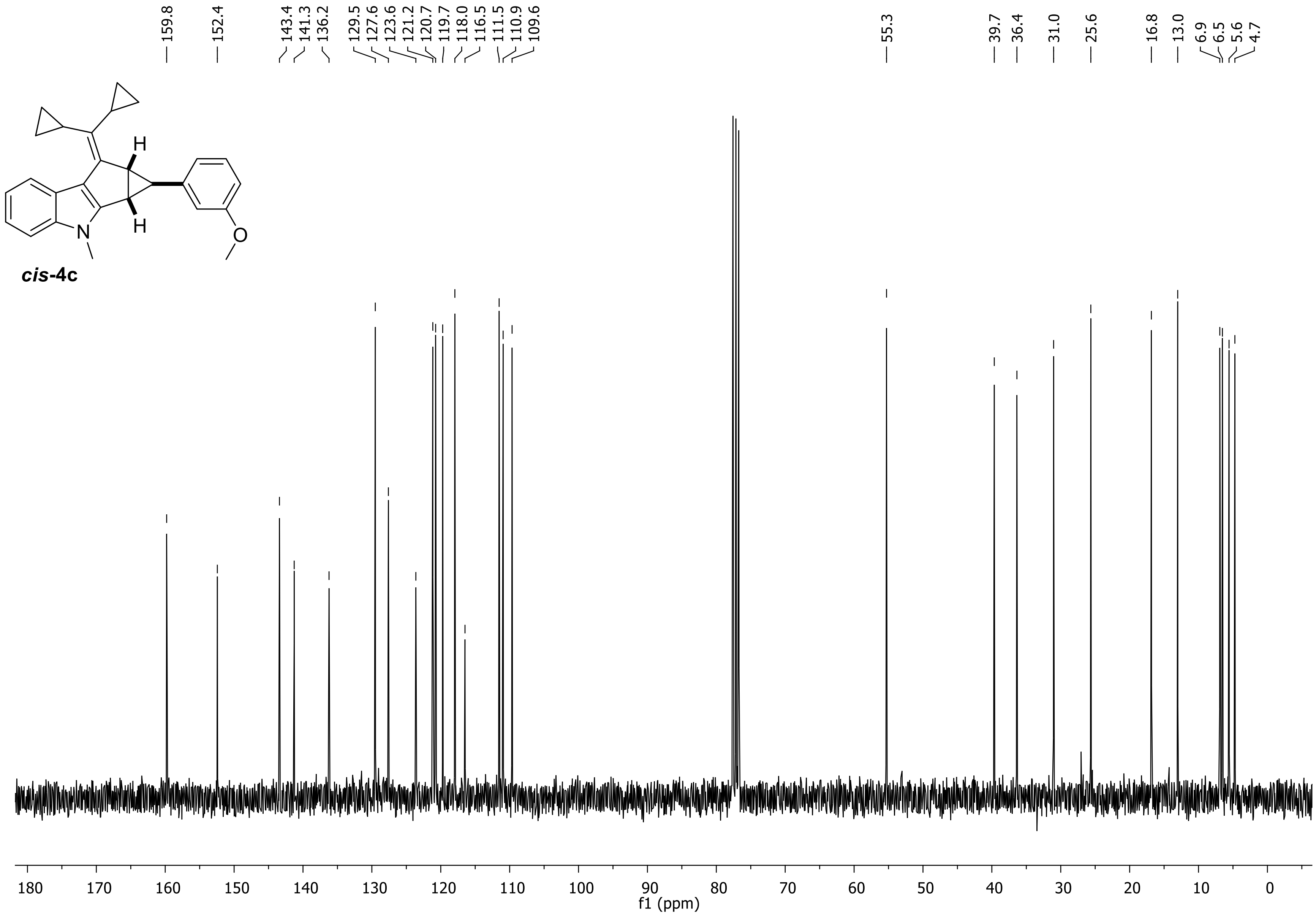

^{1}H NMR ($CDCl_3$, 300 MHz)

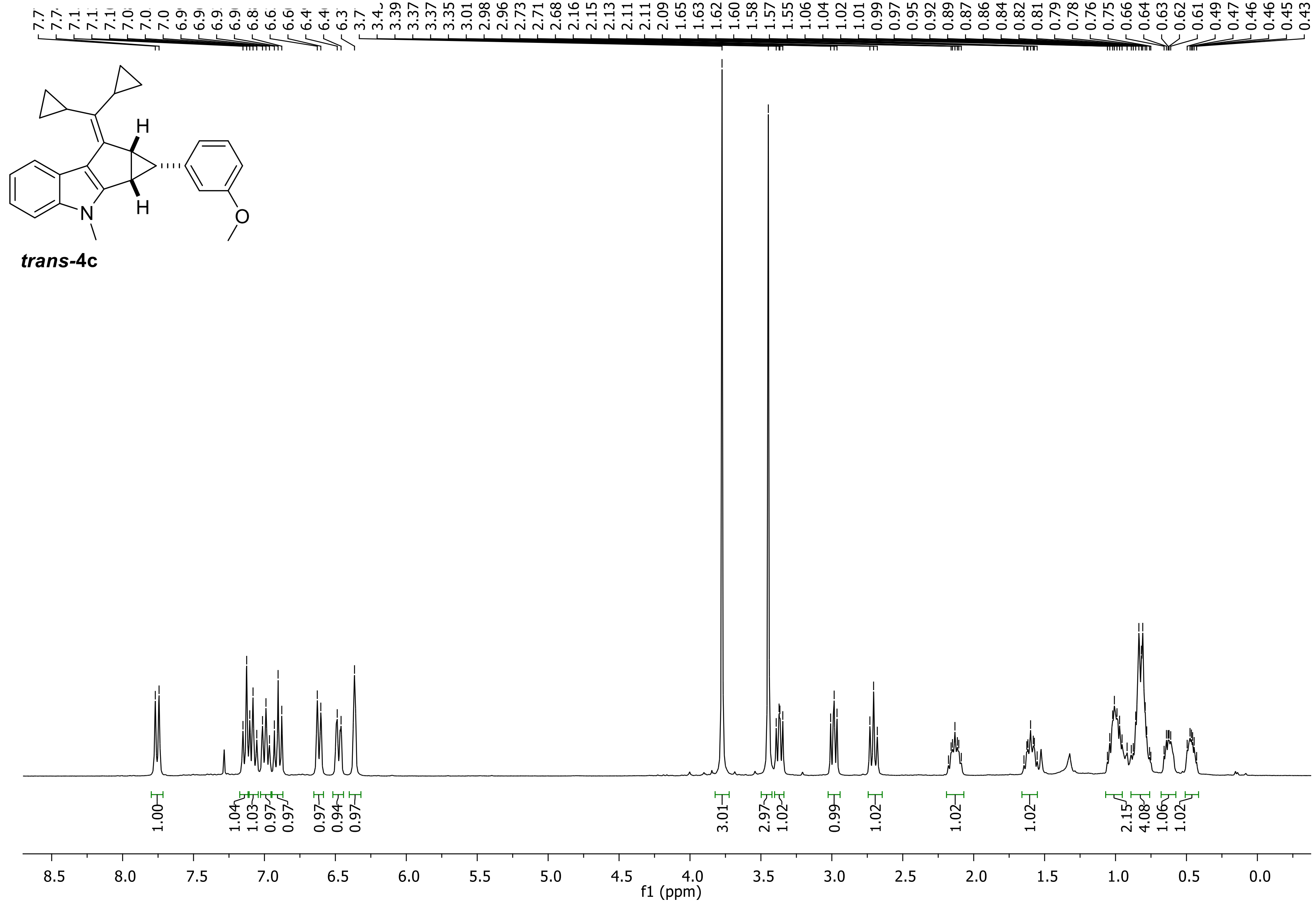

^{13}C NMR ($CDCl_3$, 125 MHz)

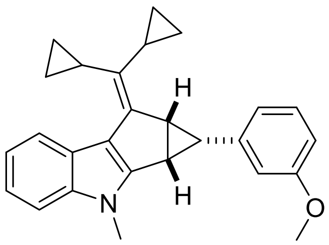

***trans*-4c**

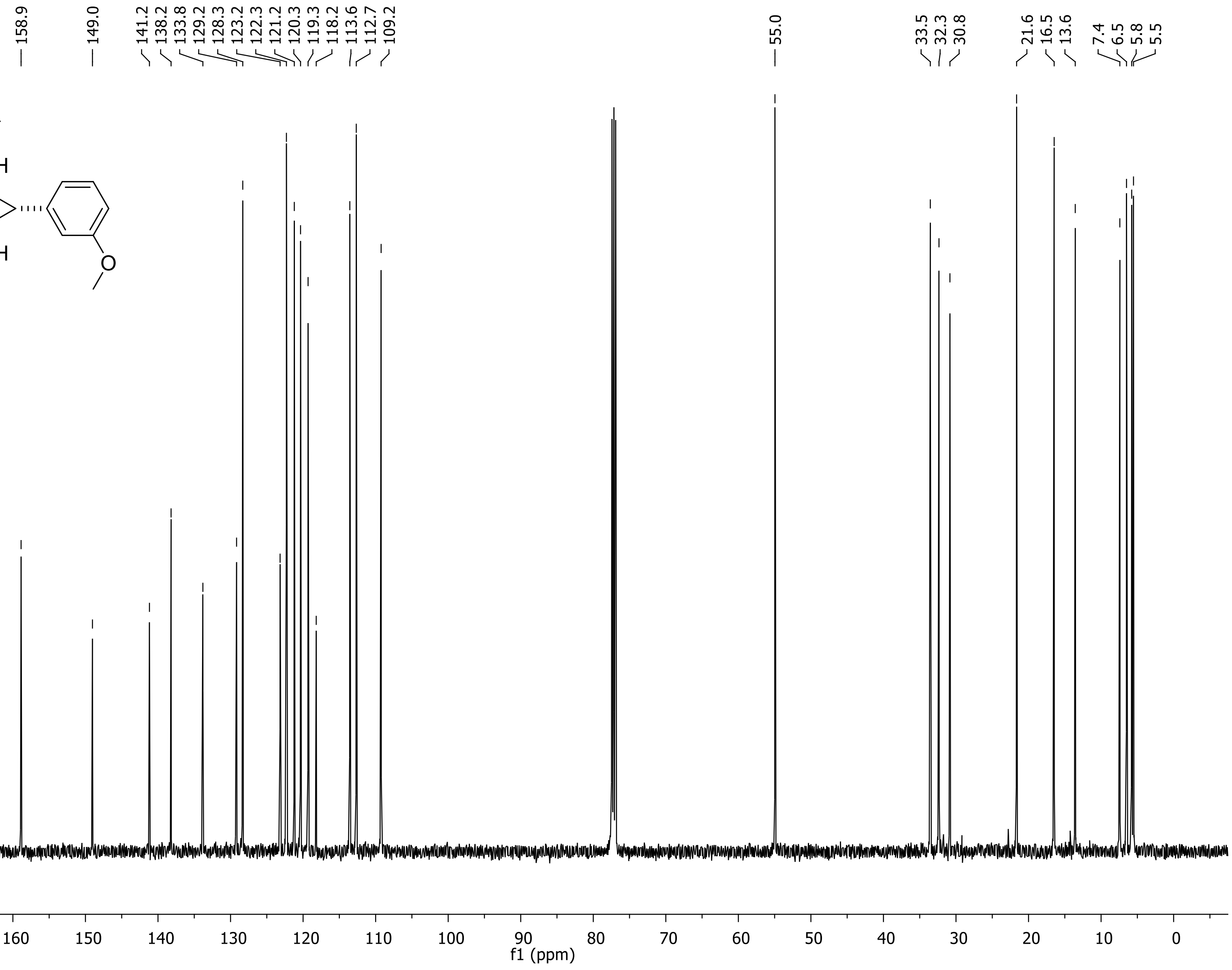

NOESY ($CDCl_3$, 500 MHz)

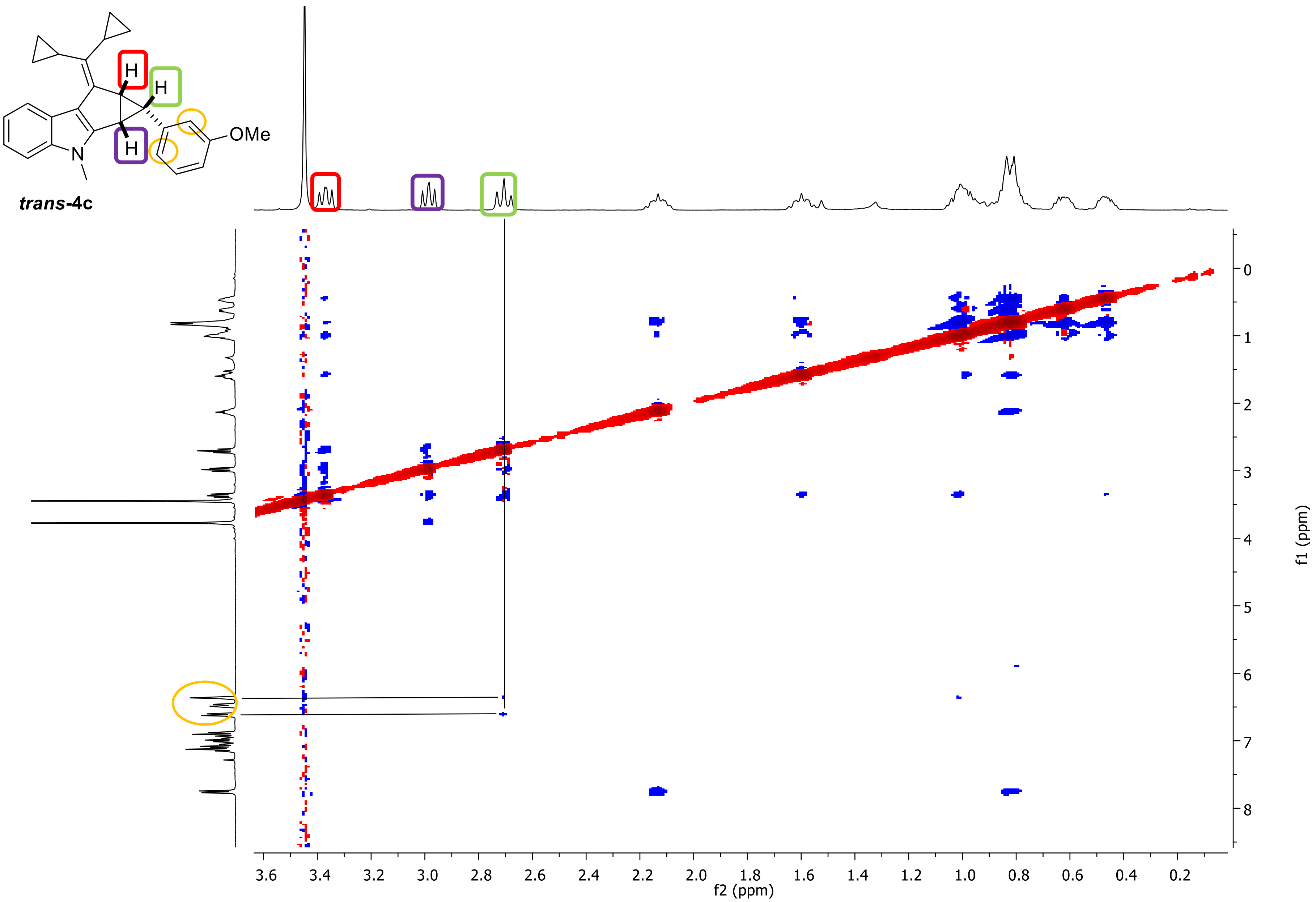

^1H NMR ($CDCl_3$, 300 MHz)

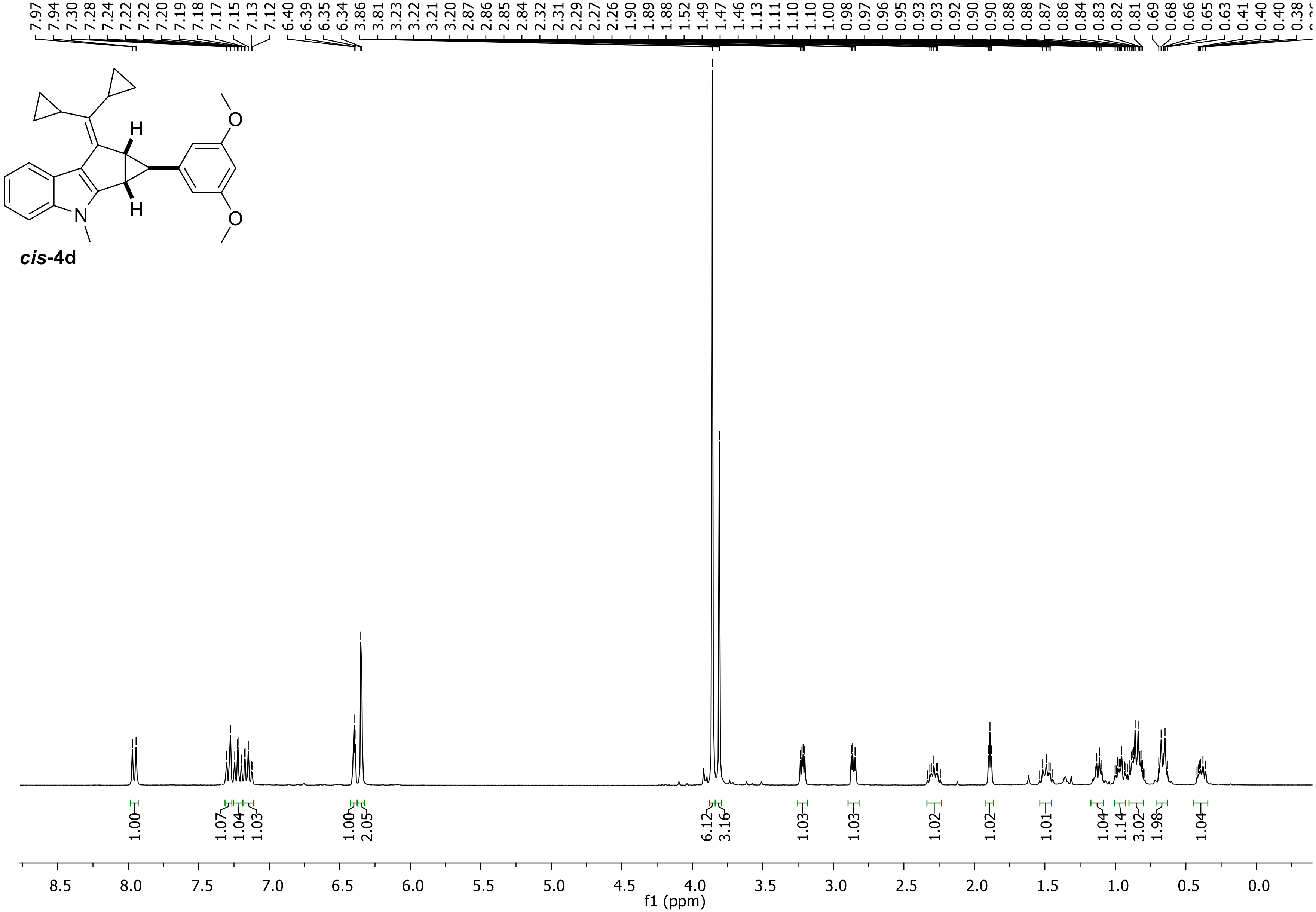

cis-**4d**

^{13}C NMR ($CDCl_3$, 75.4 MHz)

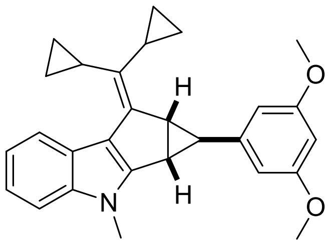

***cis*-4d**

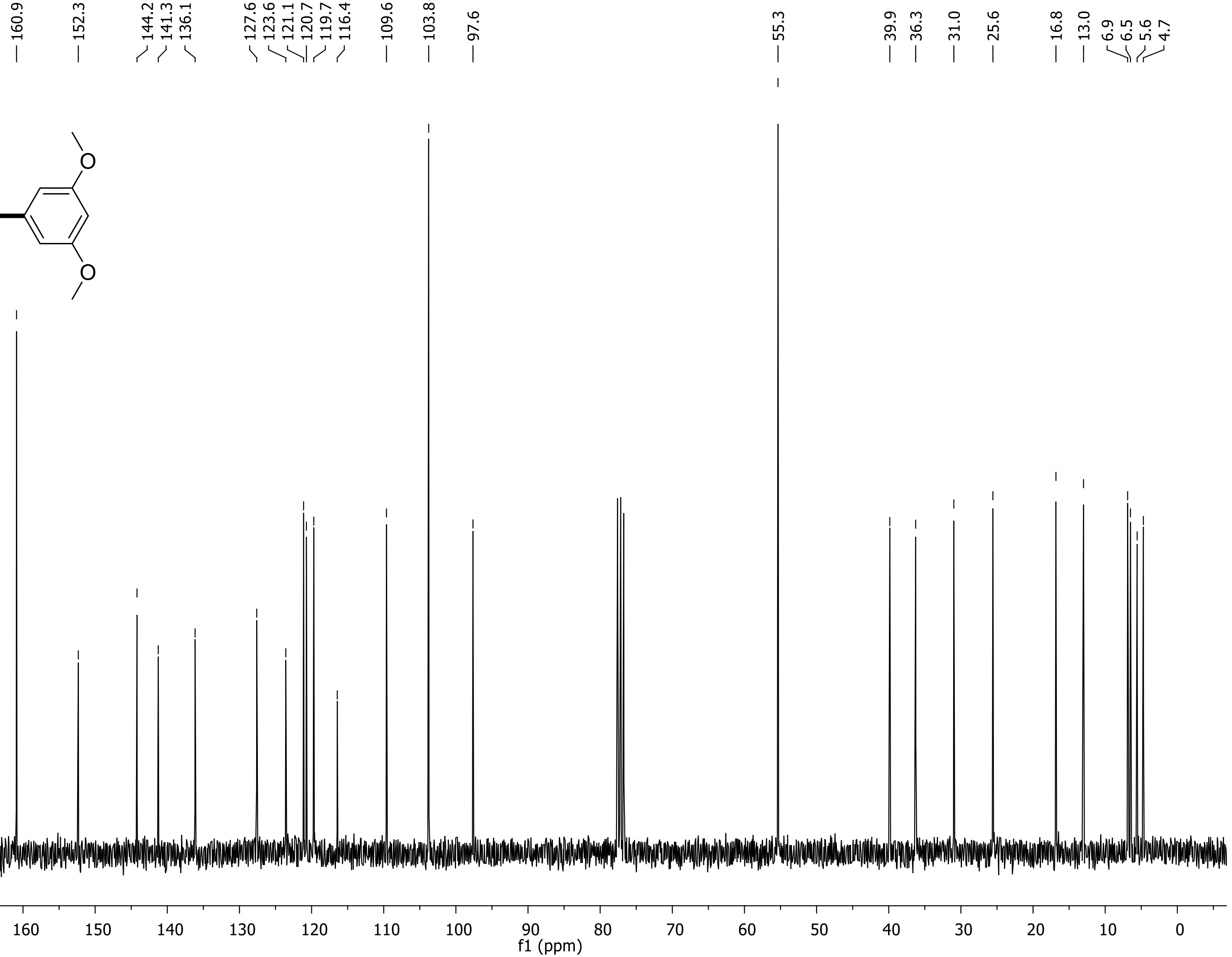

^{1}H NMR ($CDCl_3$, 300 MHz)

***trans*-4d**

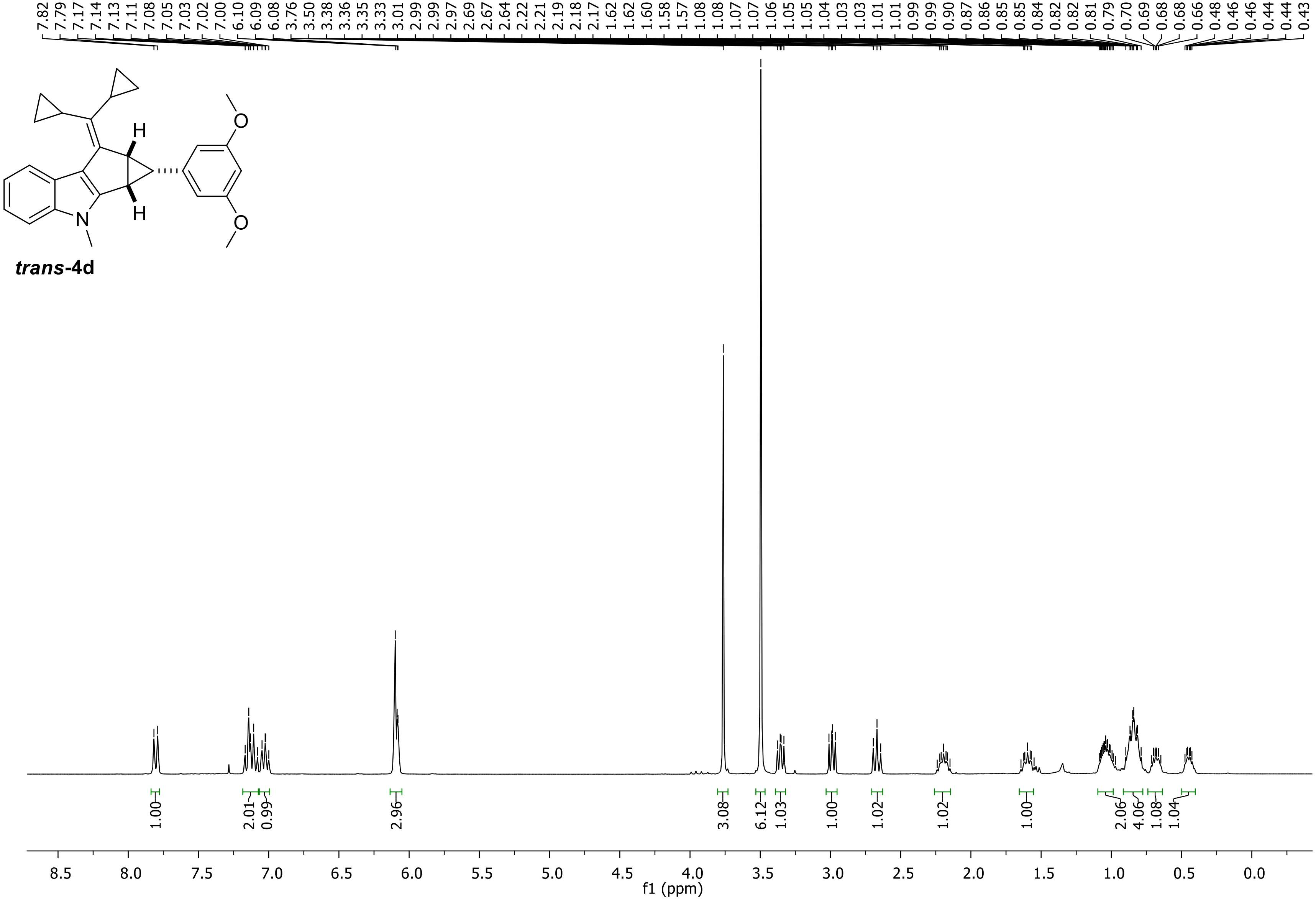

^{13}C NMR ($CDCl_3$, 75.4 MHz)

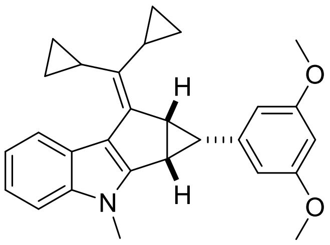

trans-**4d**

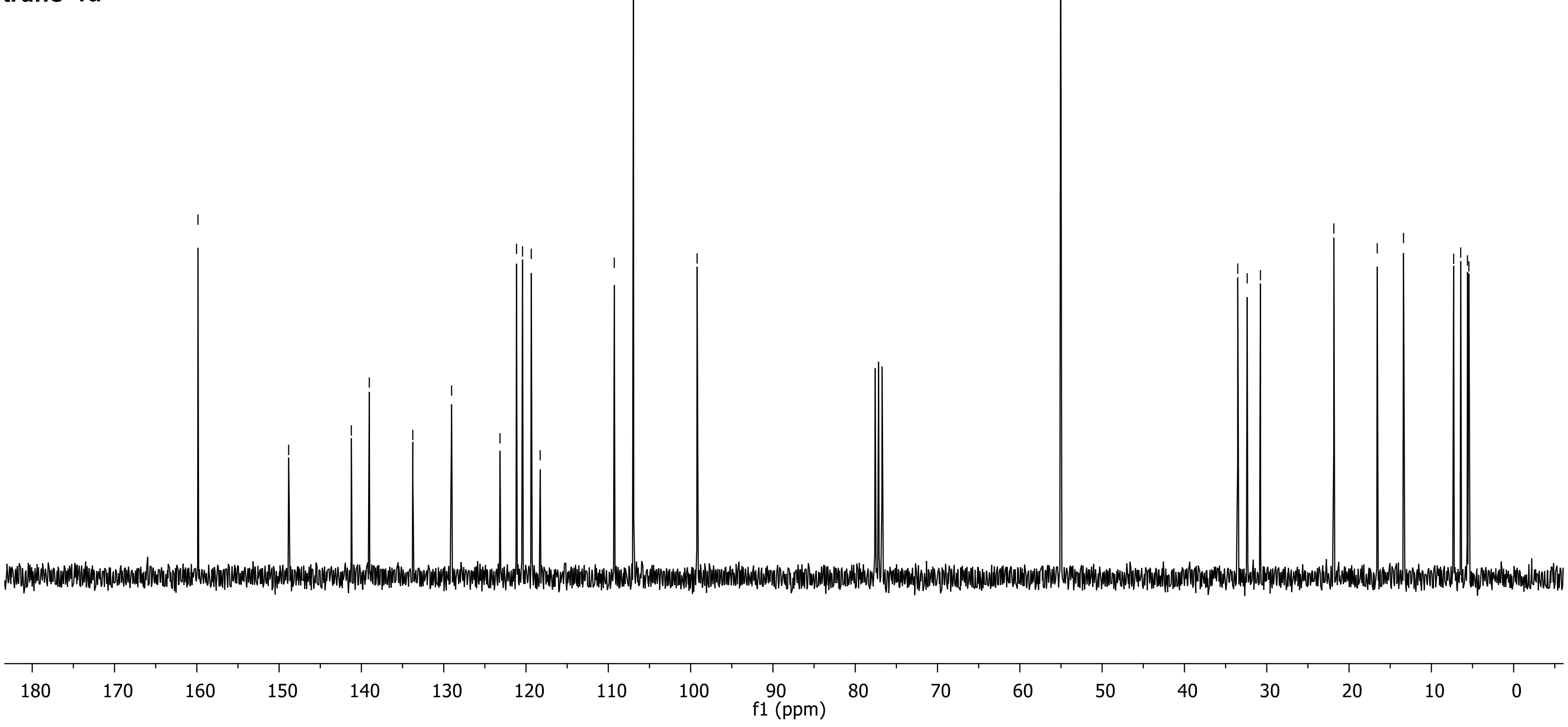

^{1}H NMR ($CDCl_3$, 300 MHz)

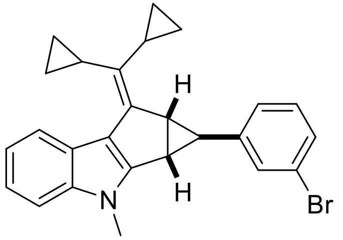

***cis*-4e**

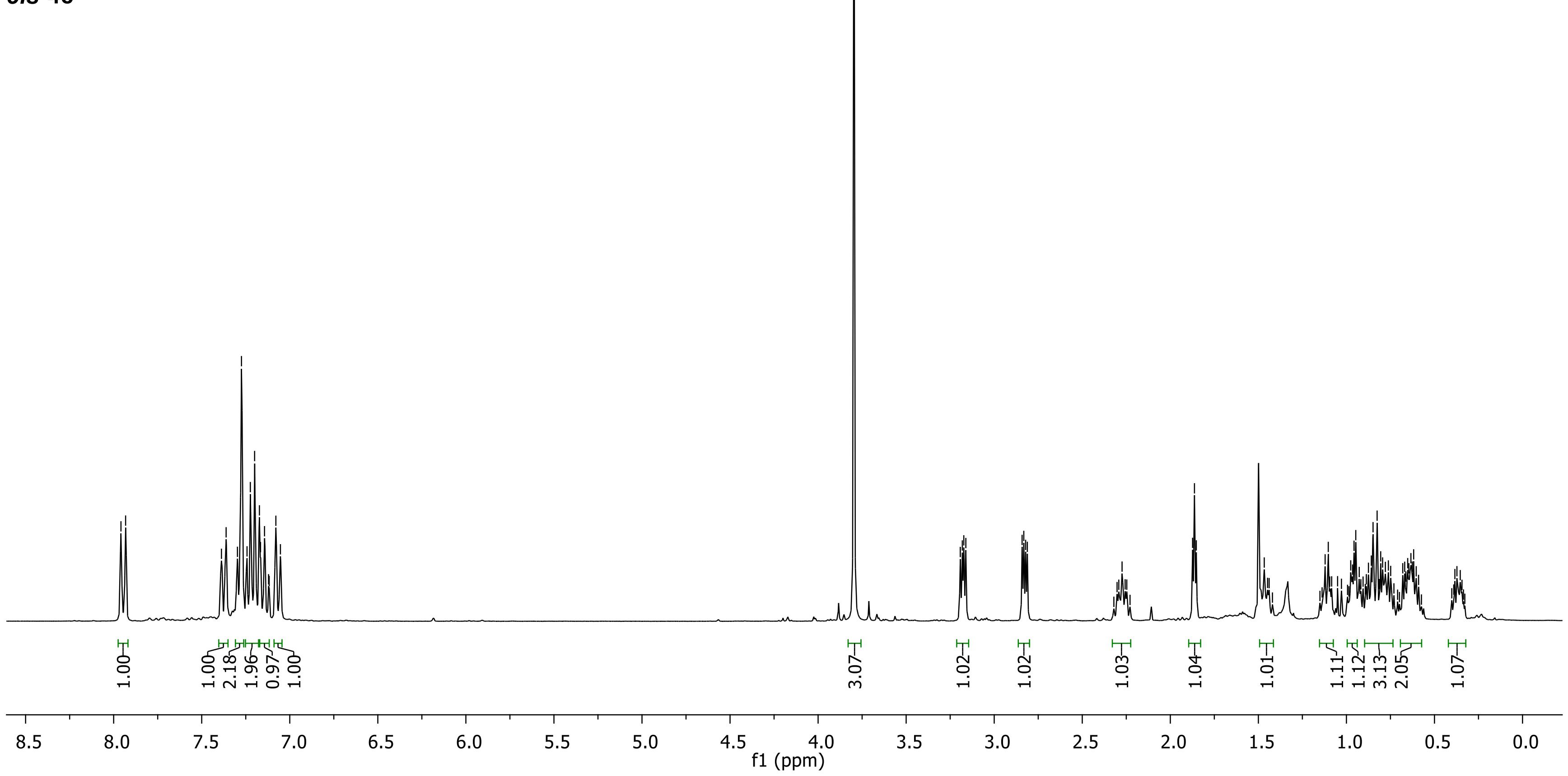

^{13}C NMR ($CDCl_3$, 75.4 MHz)

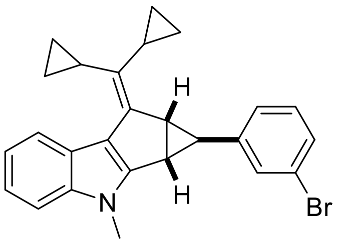

cis-**4e**

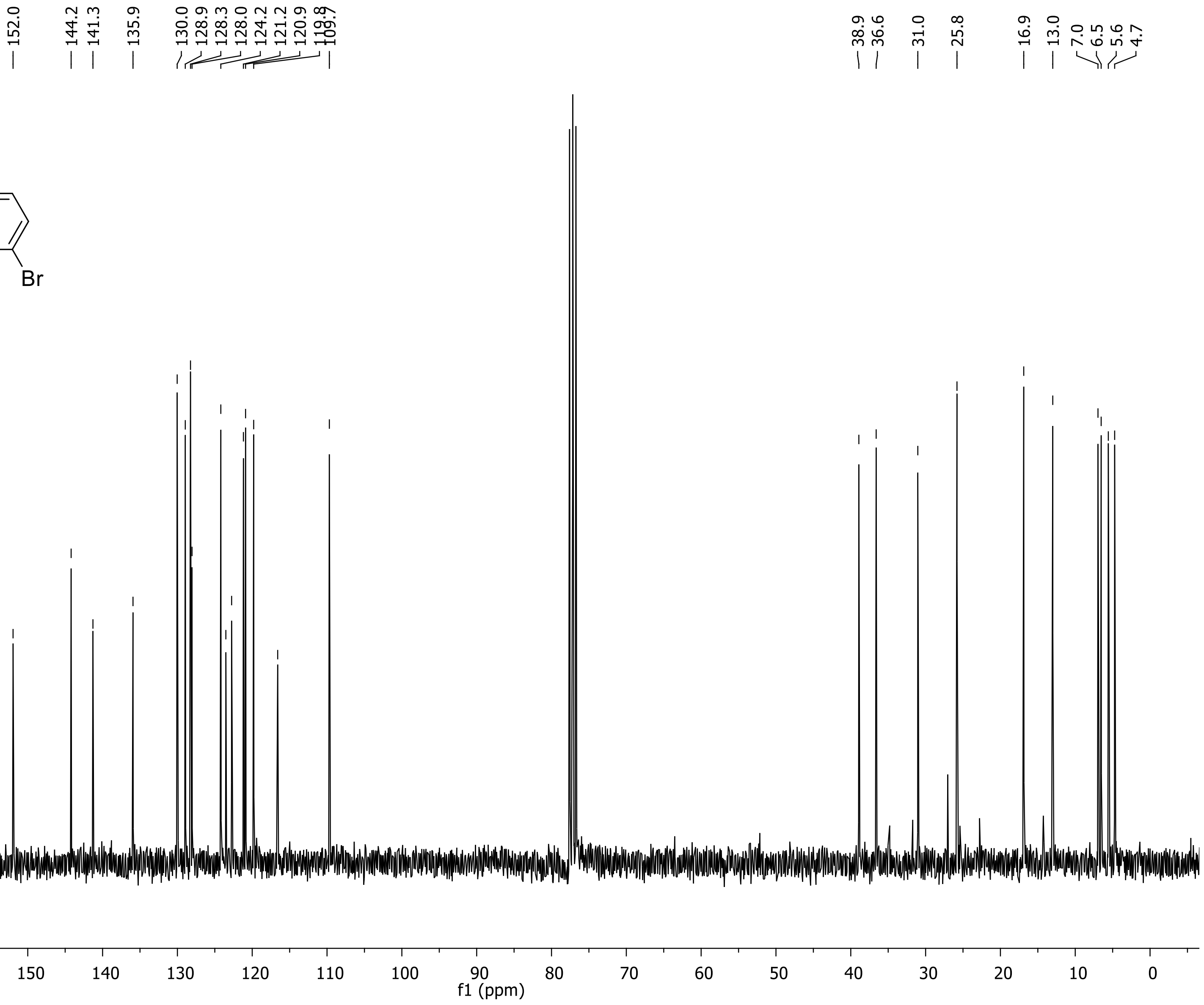

^{1}H NMR ($CDCl_3$, 500 MHz)

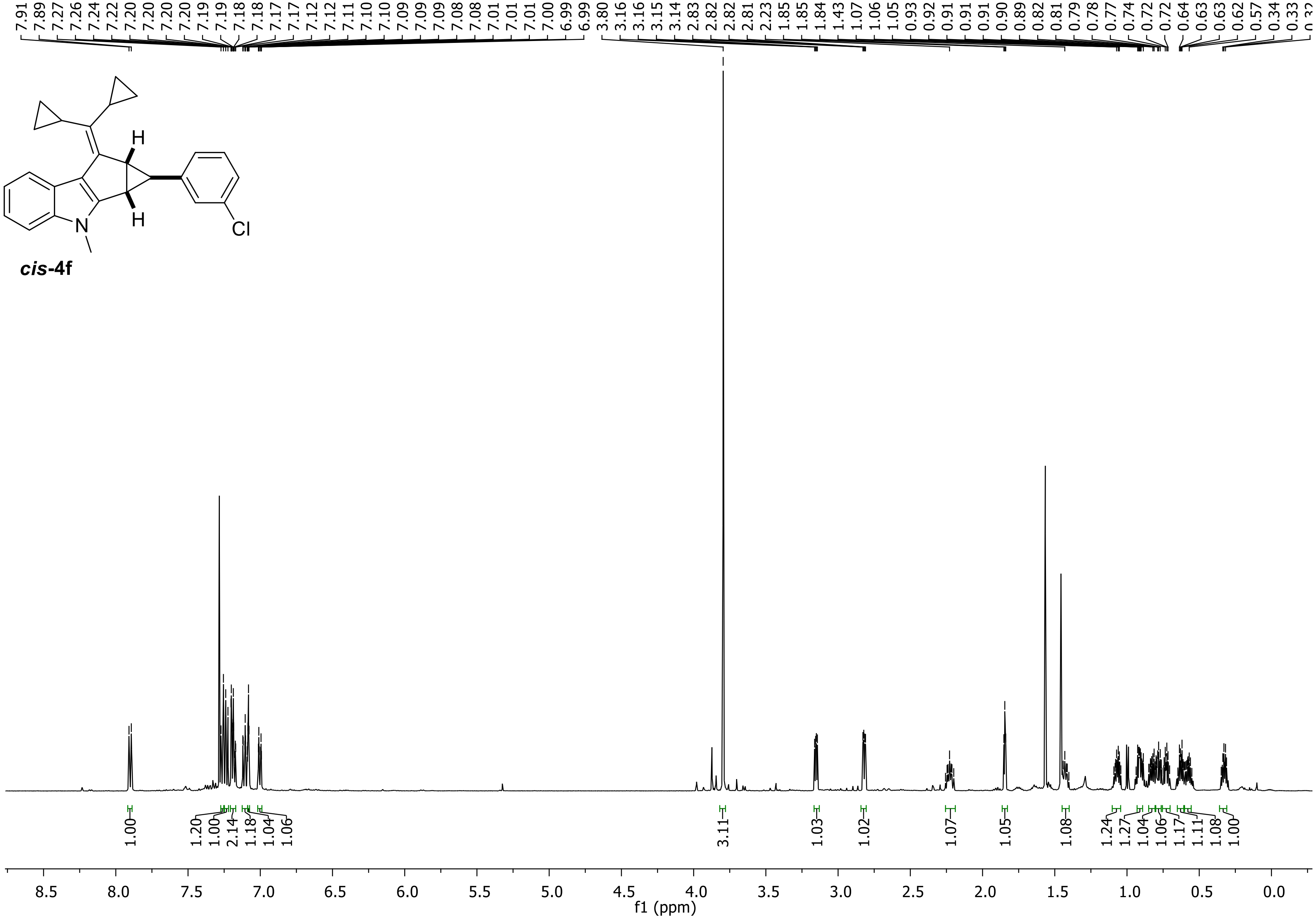

^{13}C NMR ($CDCl_3$, 75.4 MHz)

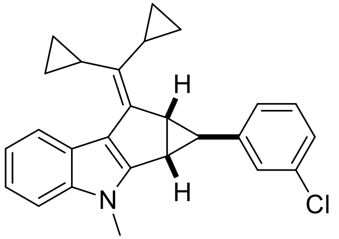

***cis*-4f**

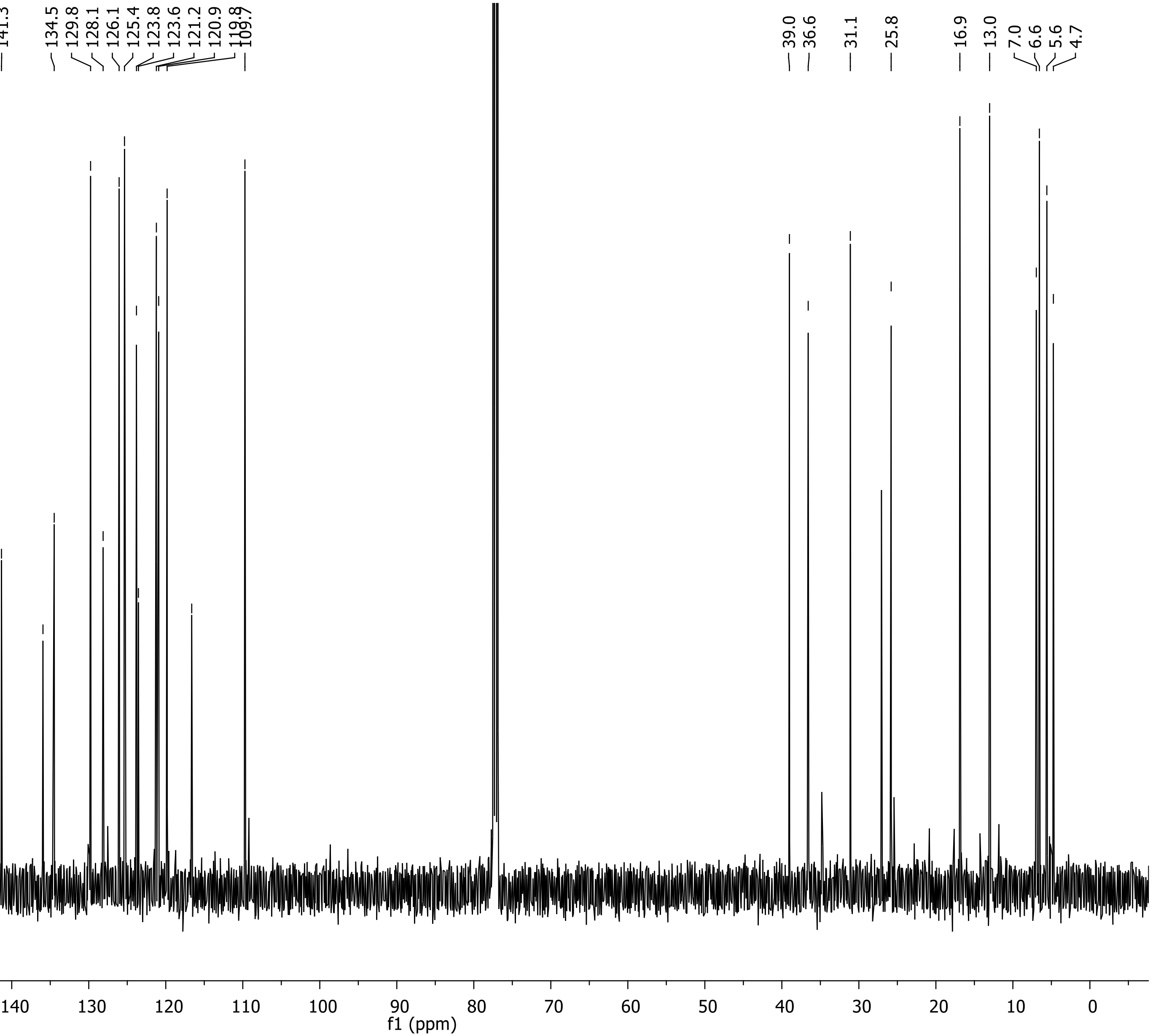

NOESY ($CDCl_3$, 500 MHz)

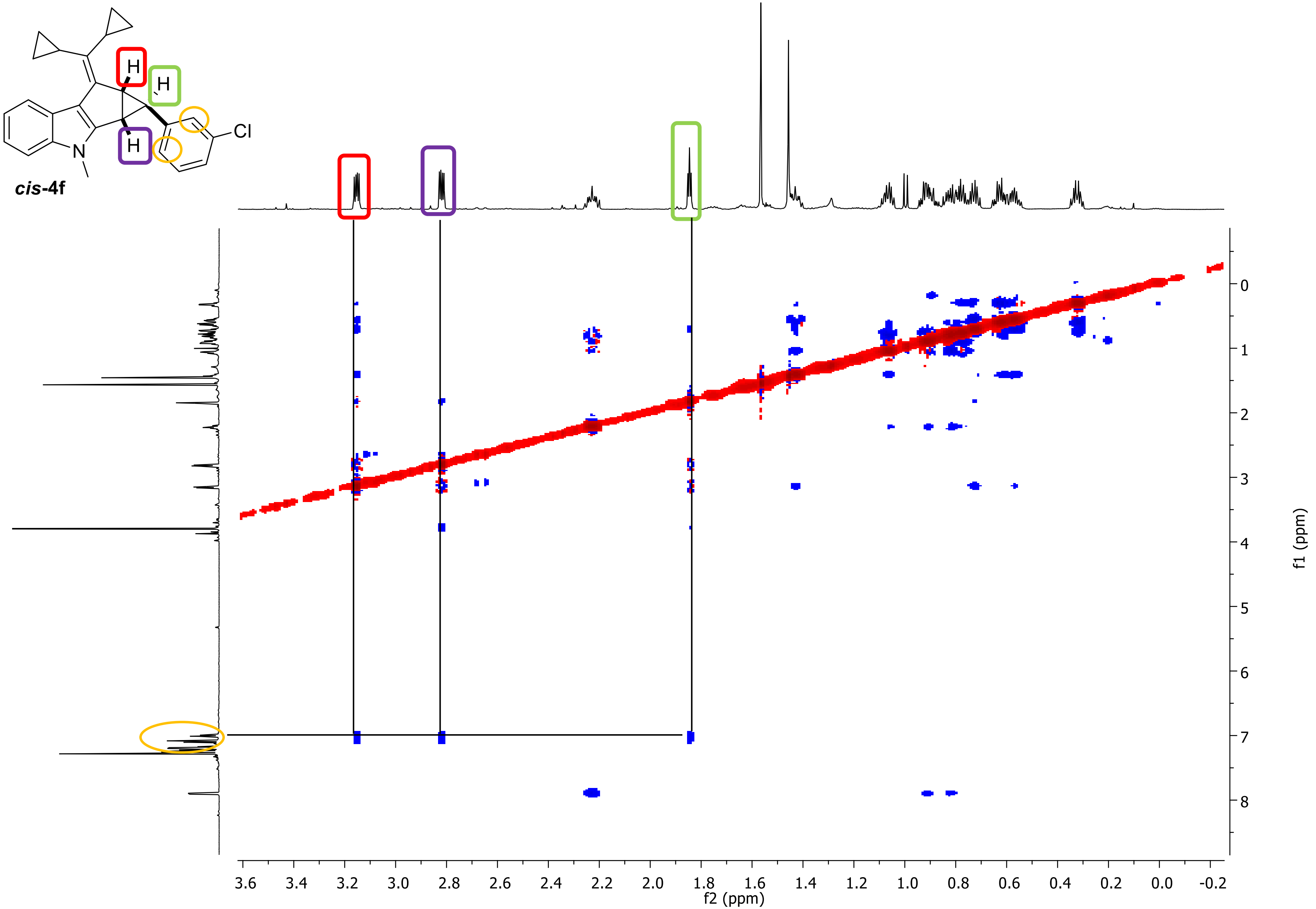

^{1}H NMR ($CDCl_3$, 300 MHz)

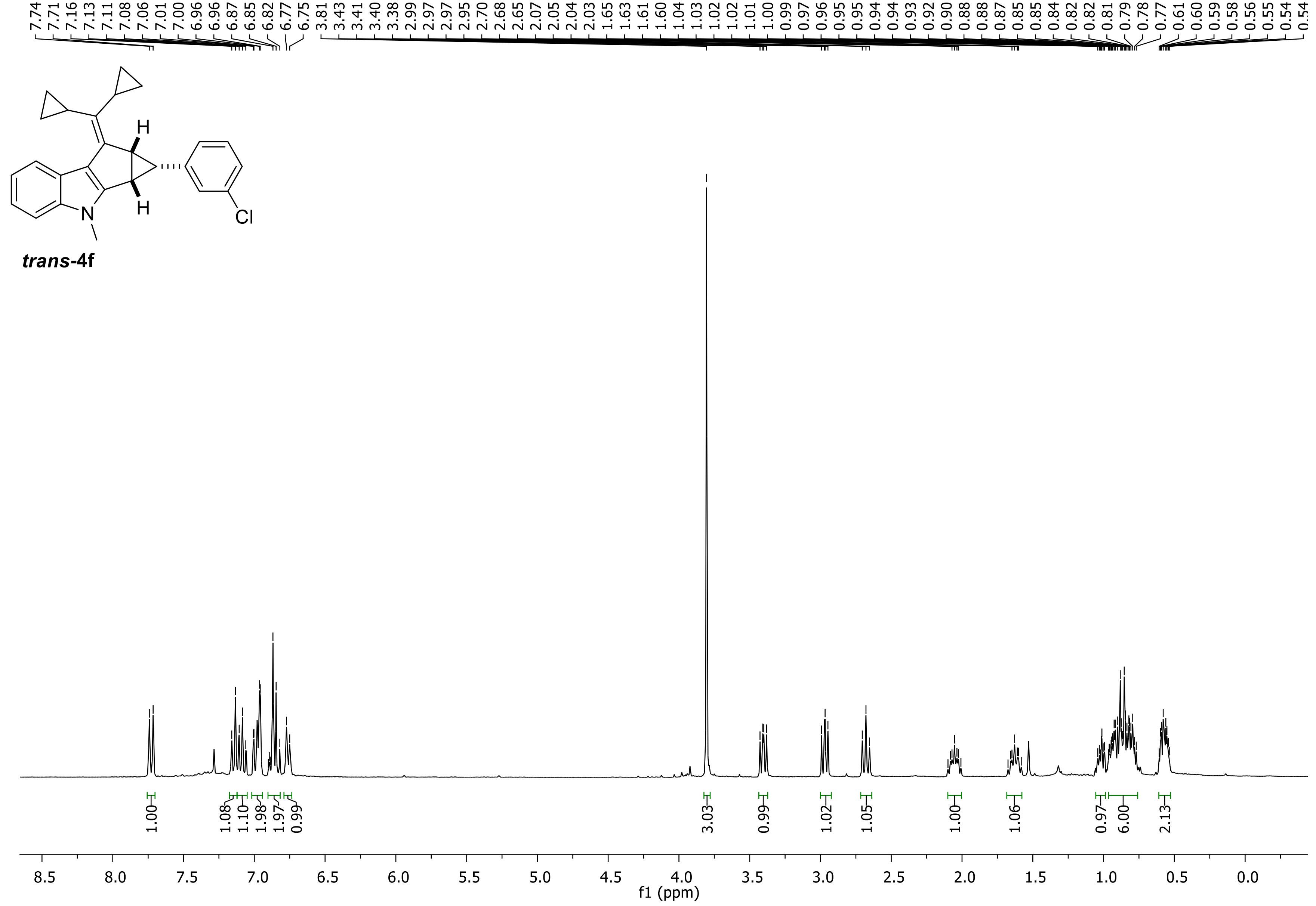

^{13}C NMR ($CDCl_3$, 75.4 MHz)

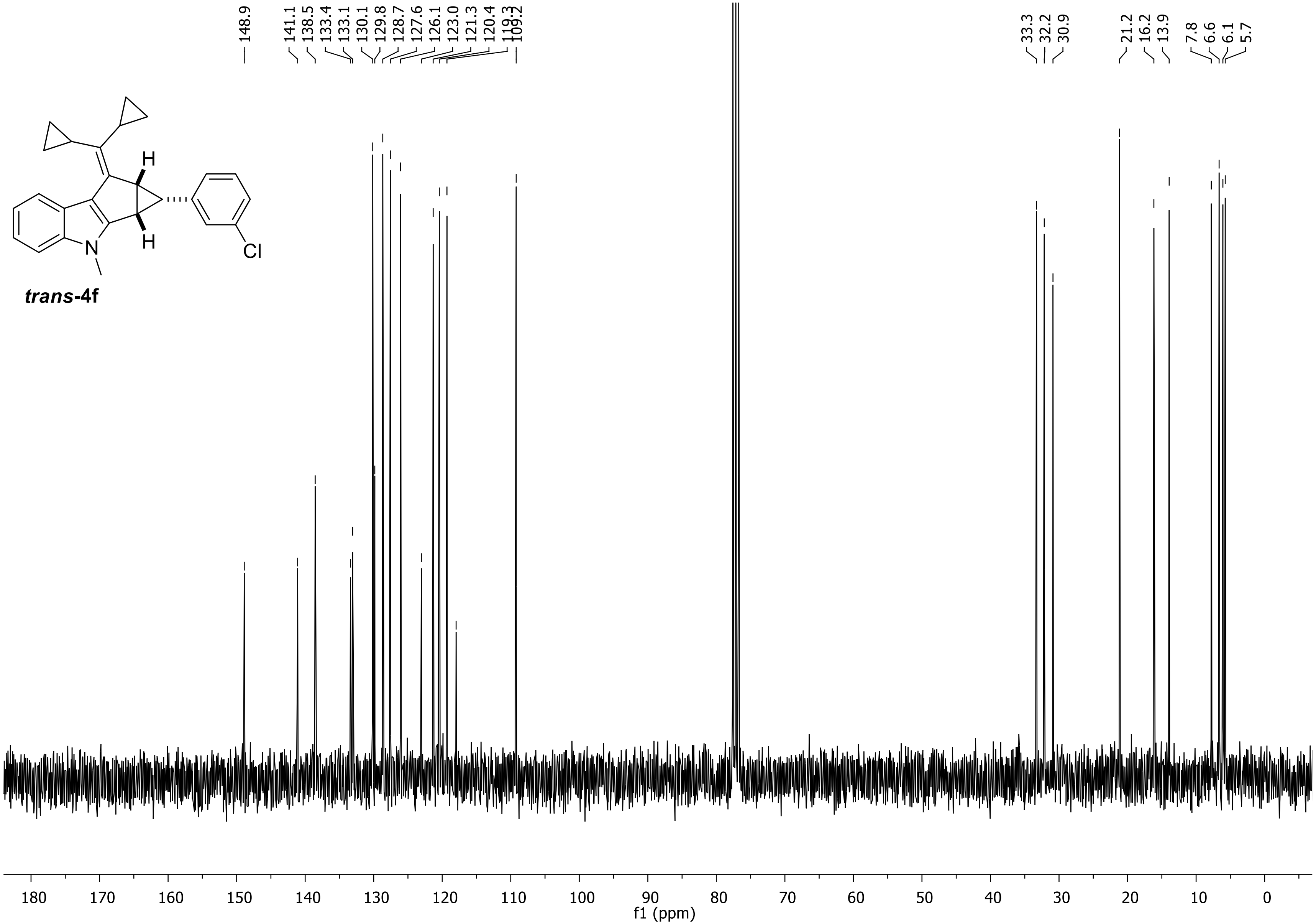

***trans*-4f**

NOESY ($CDCl_3$, 500 MHz)

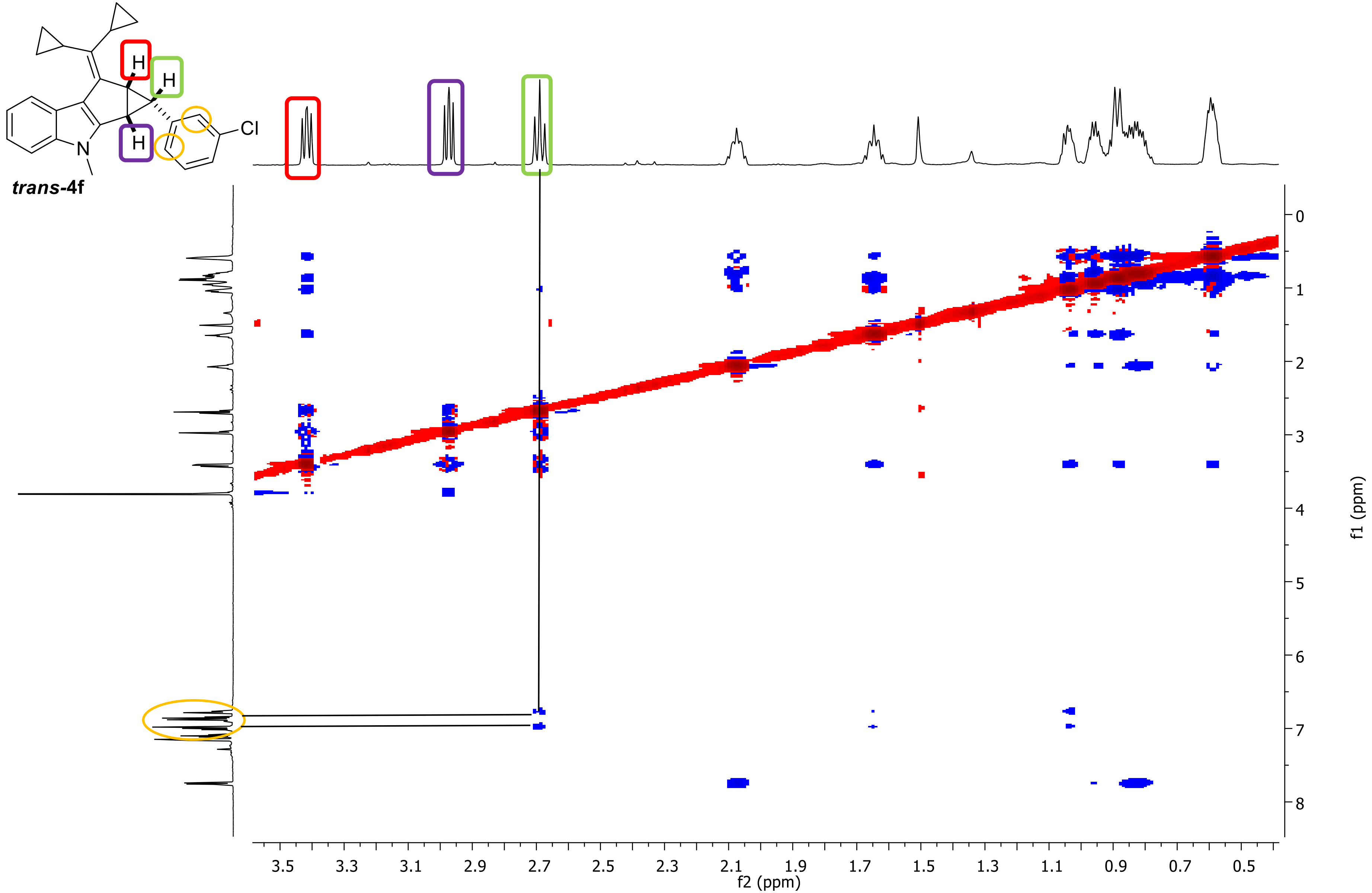

^{1}H NMR (CD_3COCD_3, 300 MHz)

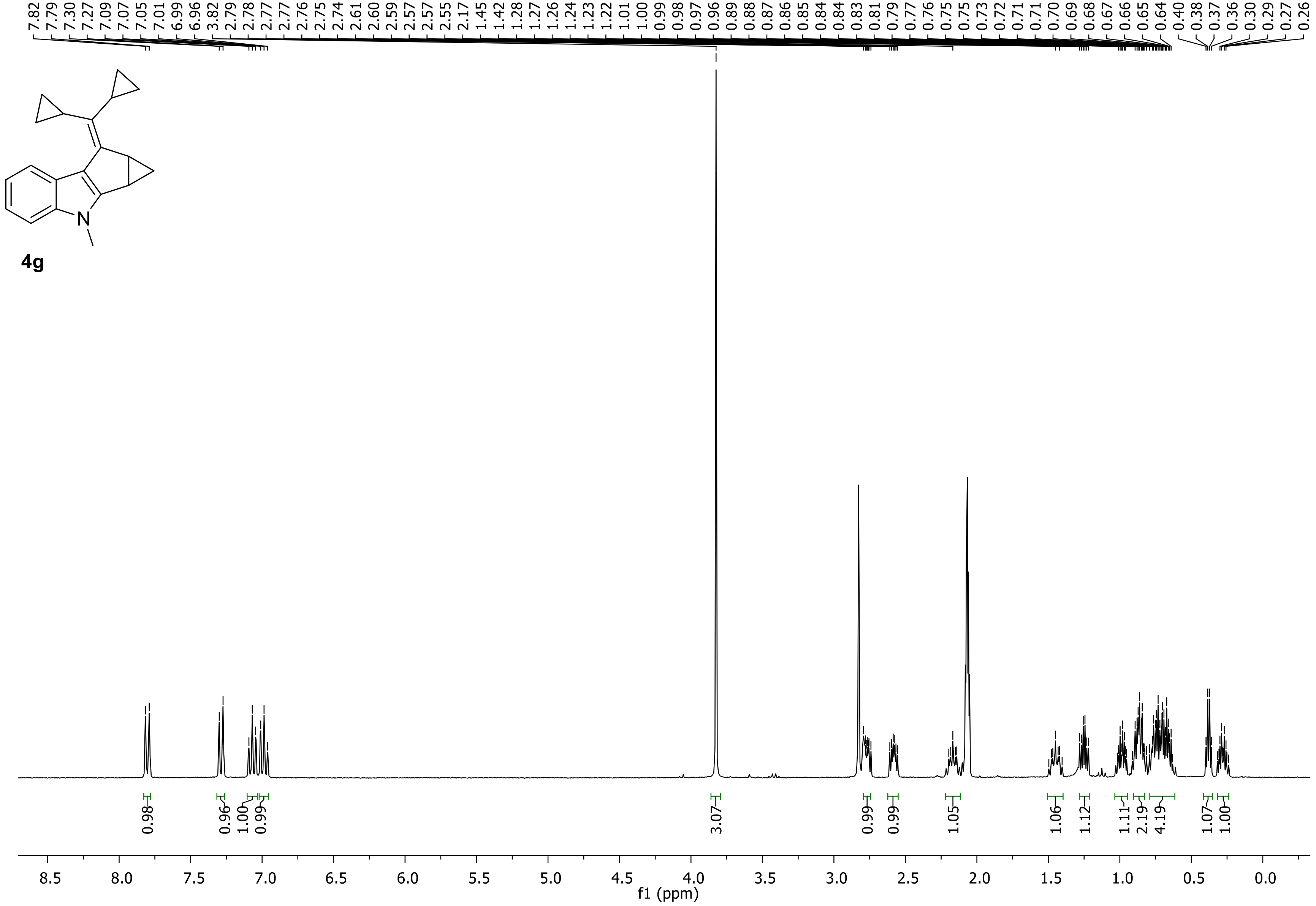

^{13}C NMR (CD_3COCD_3, 75.4 MHz)

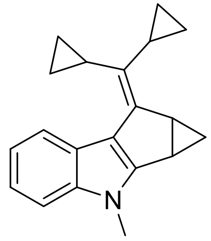

4g

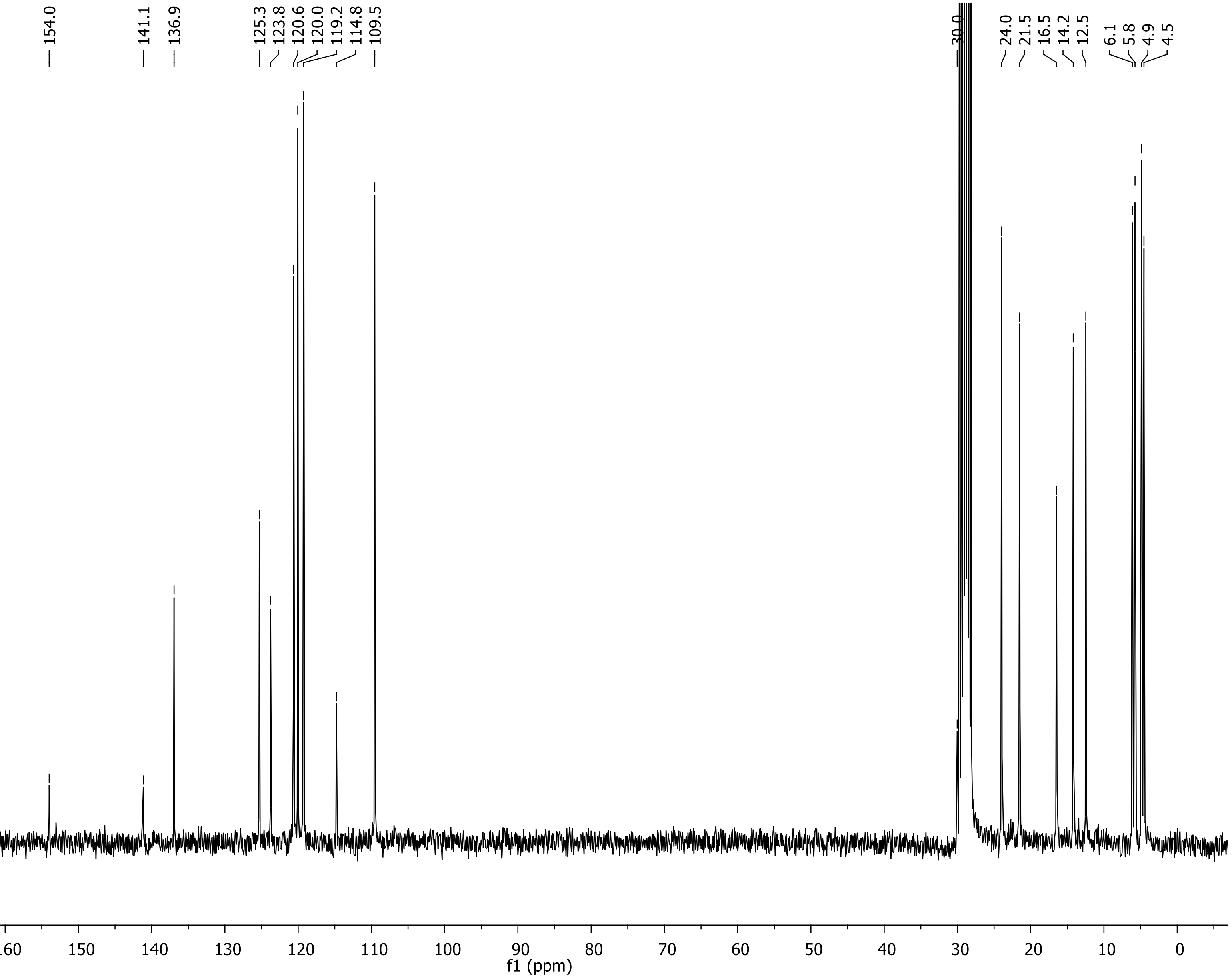

^{1}H NMR ($CDCl_3$, 300 MHz)

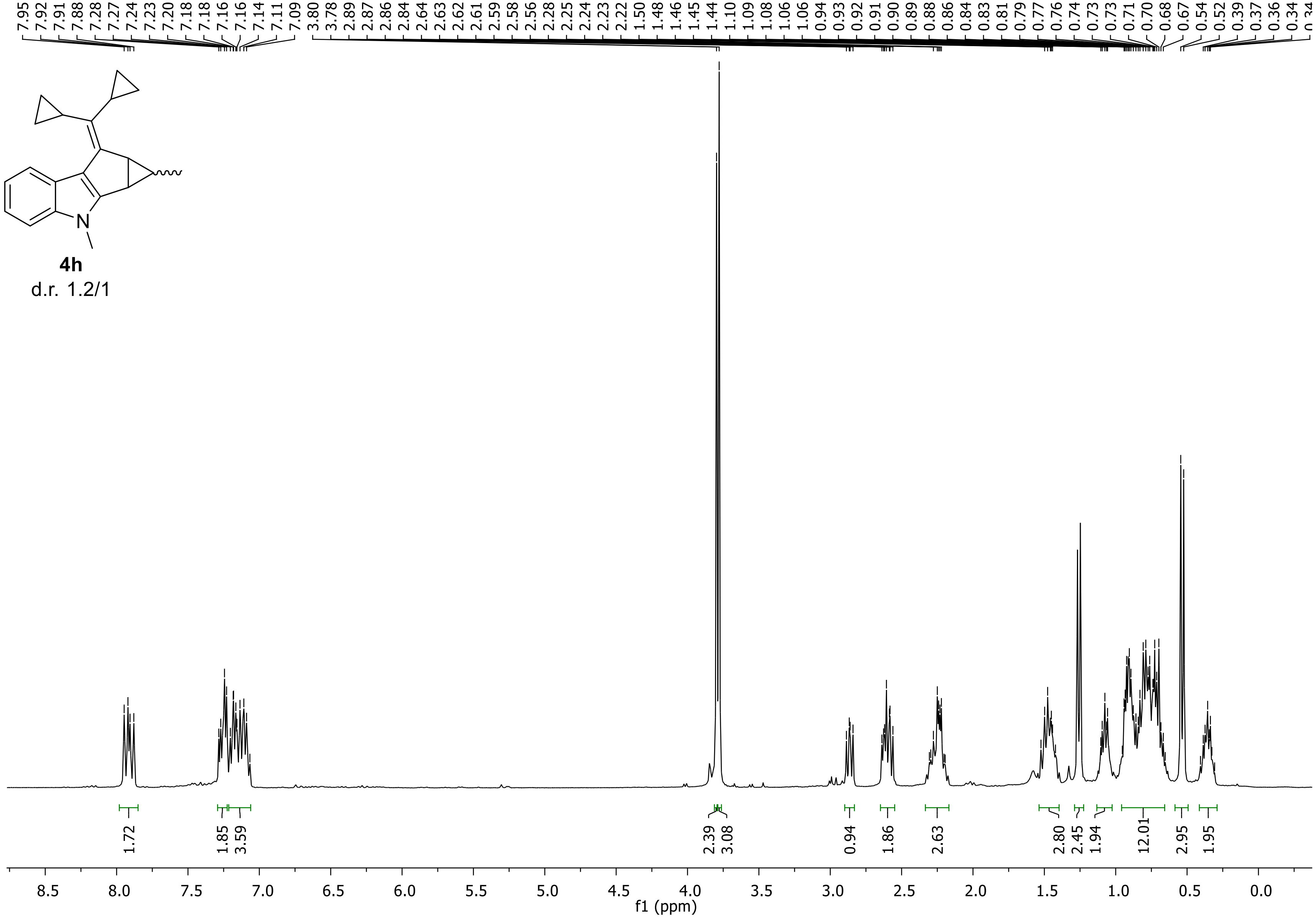

4h

d.r. 1.2/1

^{13}C NMR ($CDCl_3$, 75.4 MHz)

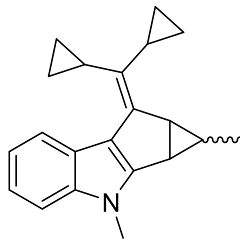

4h
d.r. 1.2/1

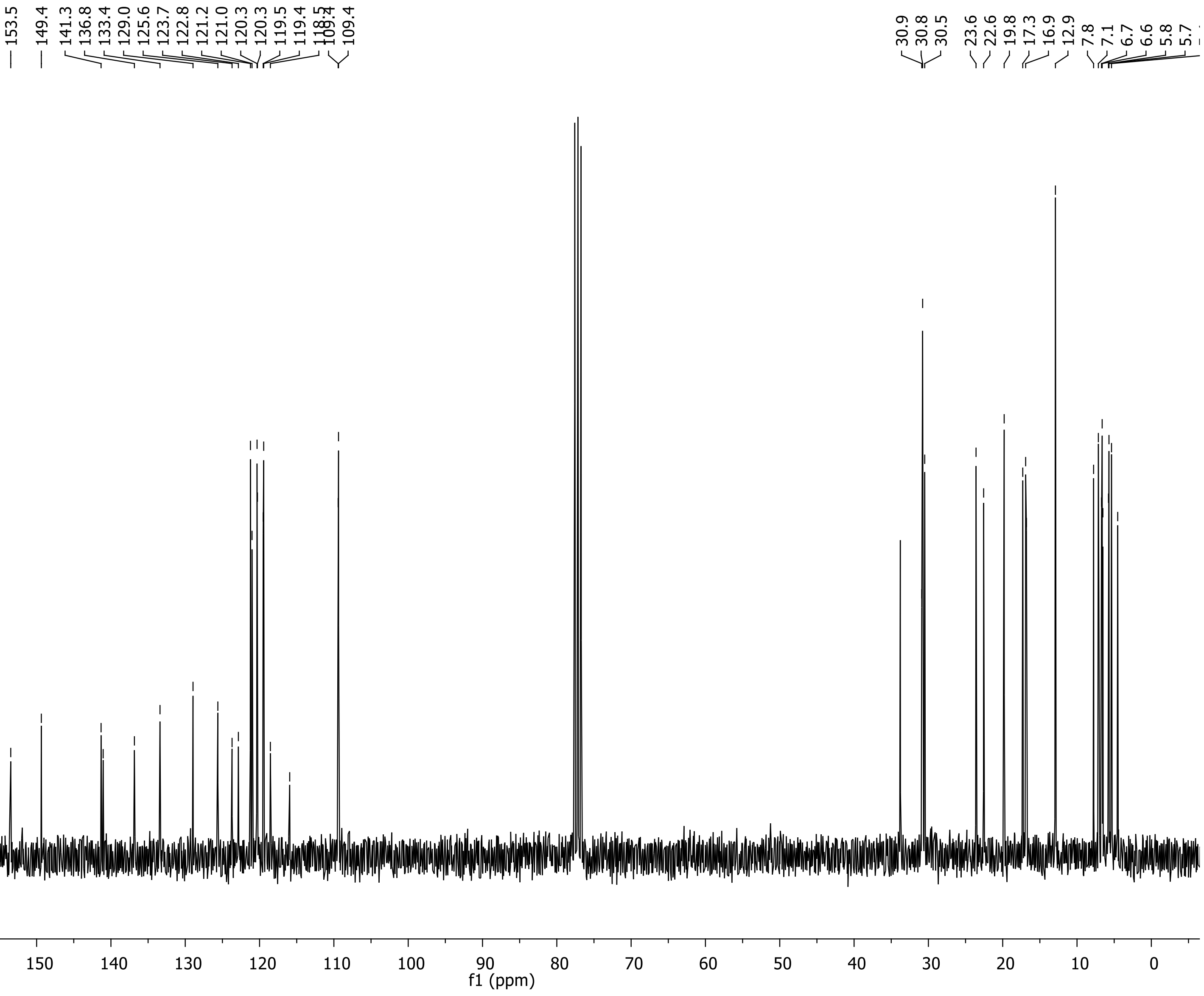

^{1}H NMR ($CDCl_3$, 300 MHz)

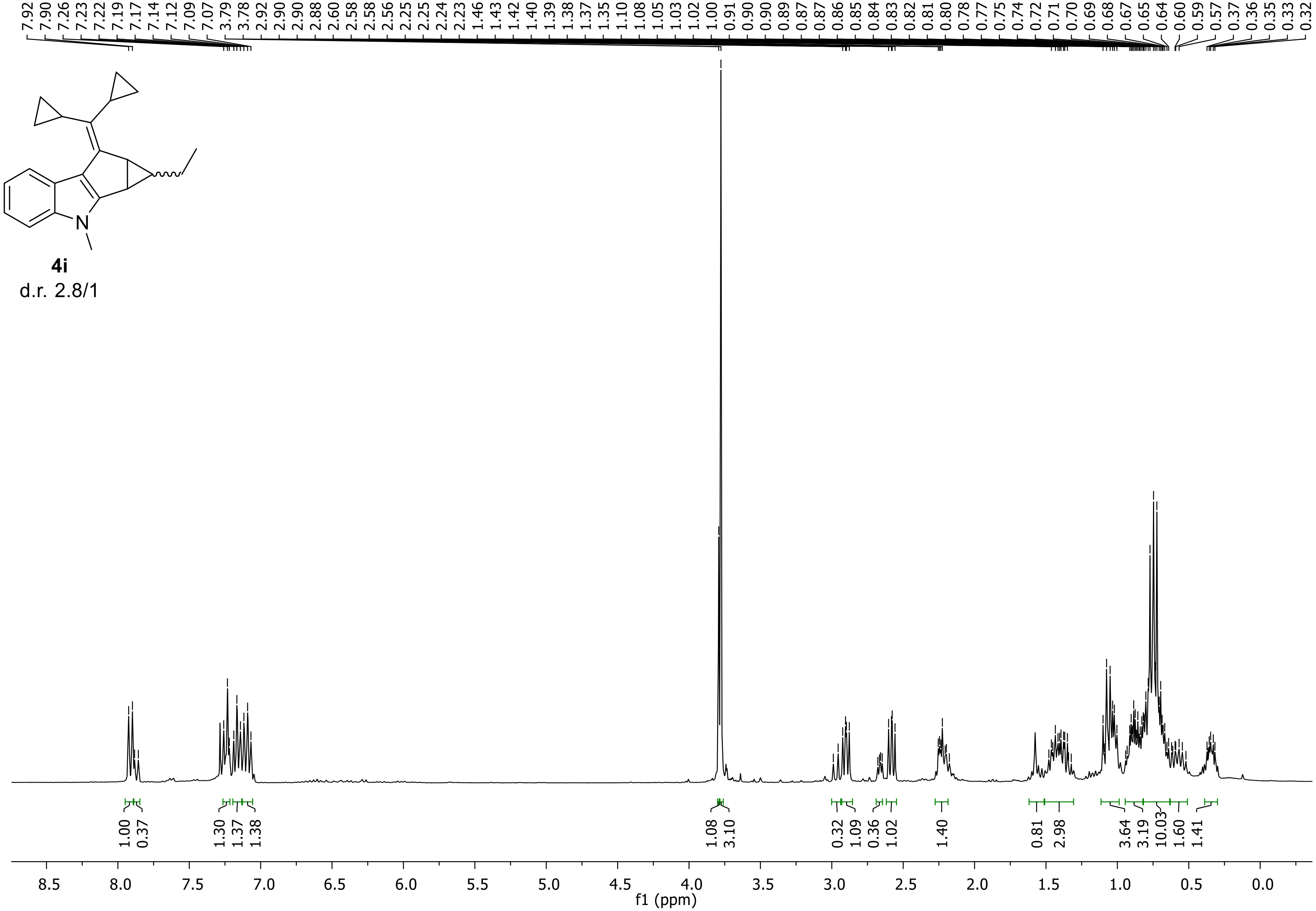

4i
d.r. 2.8/1

^{13}C NMR ($CDCl_3$, 75.4 MHz)

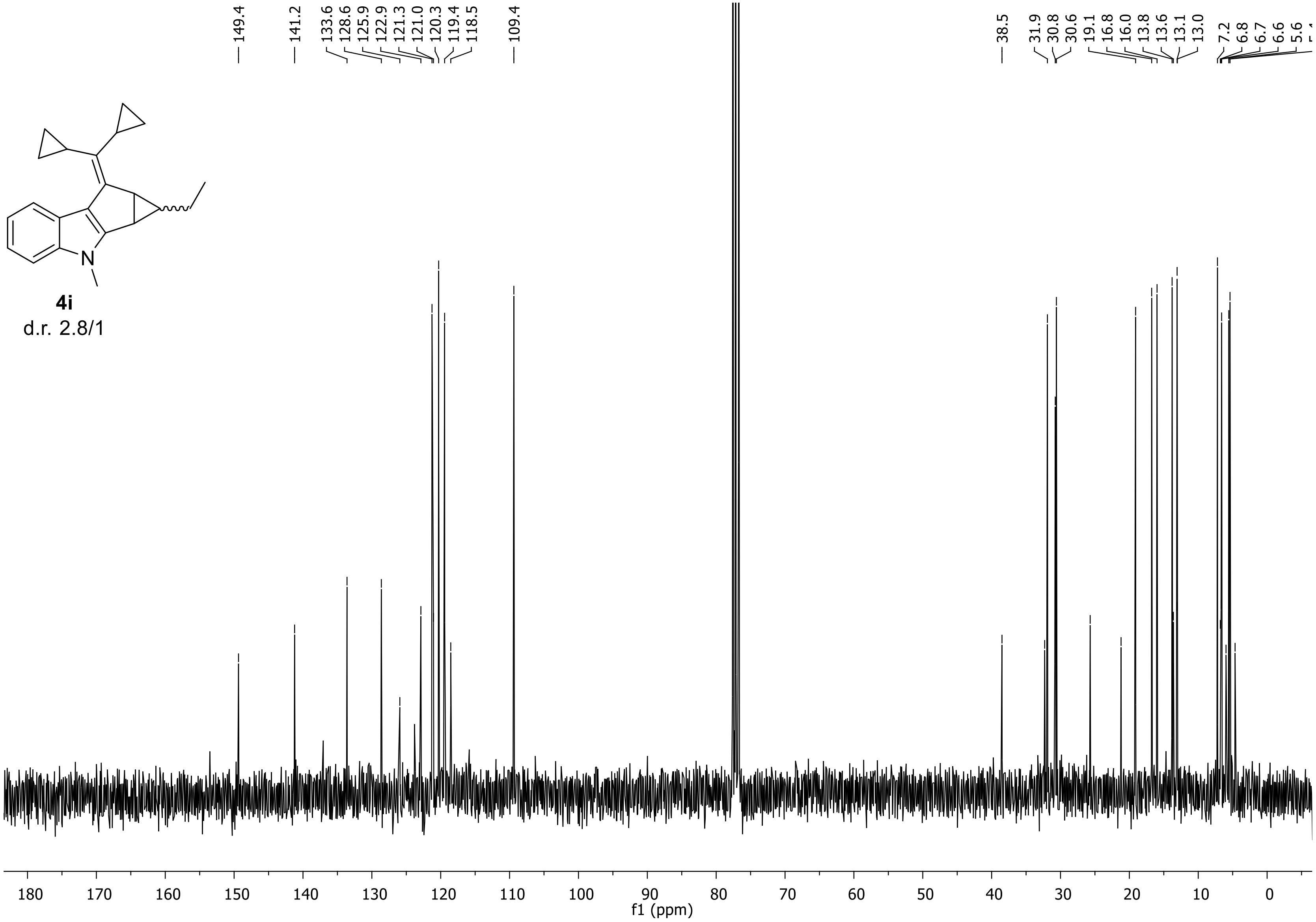

^{1}H NMR ($CDCl_3$, 300 MHz)

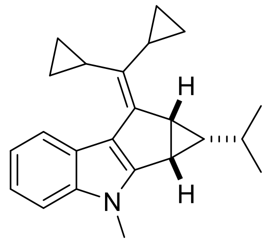

***trans*-4j**

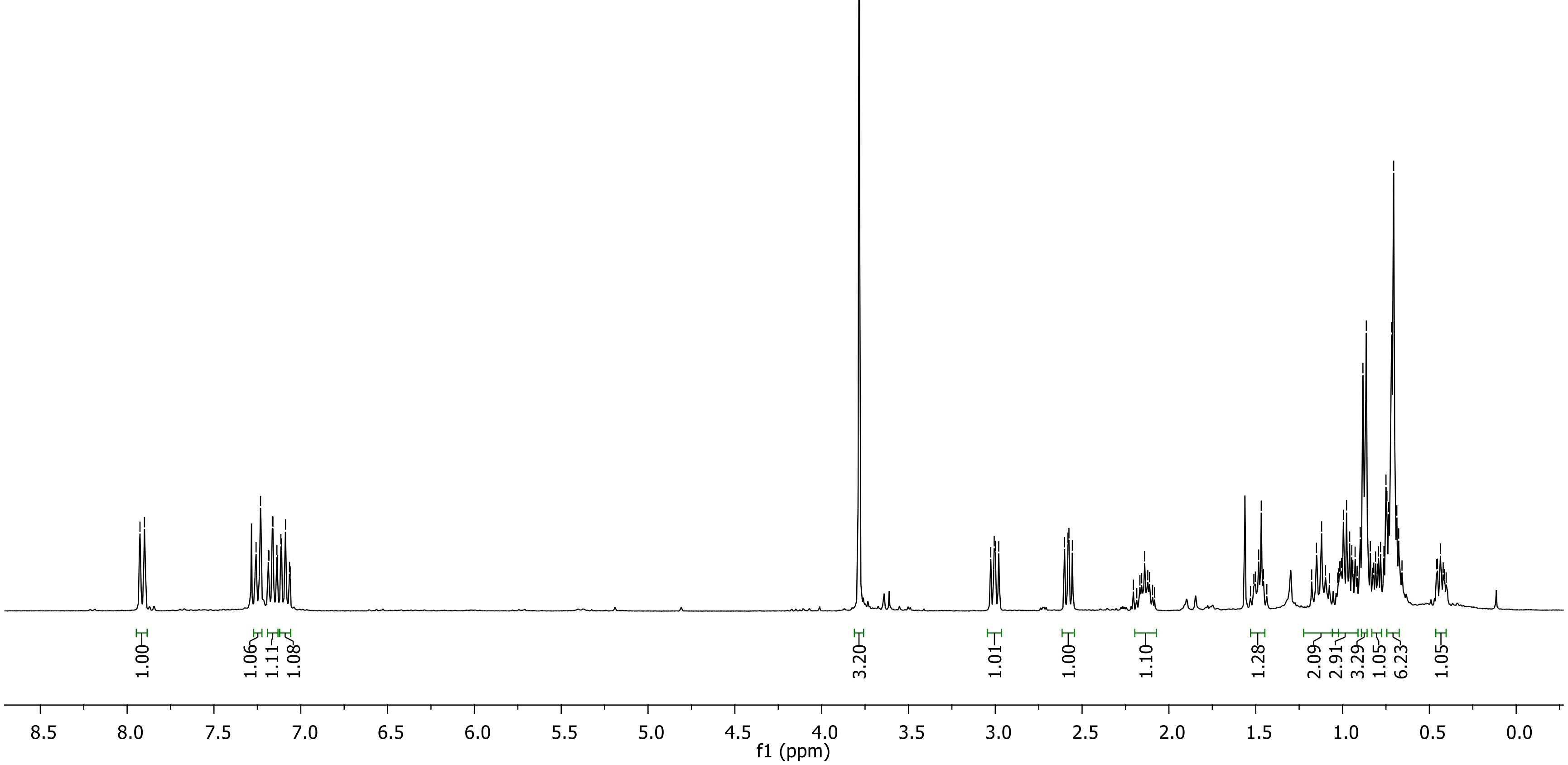

^{13}C NMR ($CDCl_3$, 75.4 MHz)

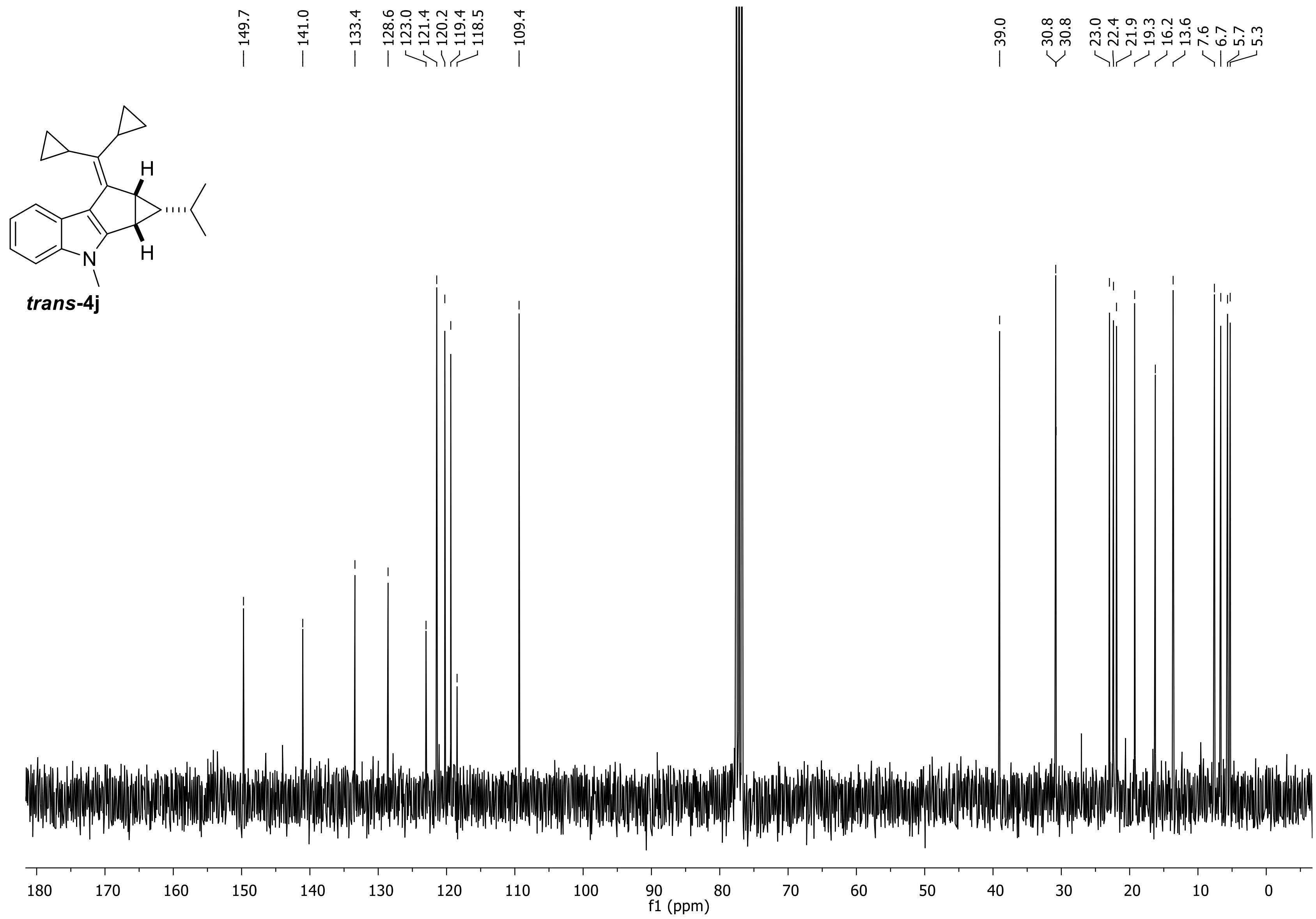

***trans*-4j**

^{1}H NMR ($CDCl_3$, 300 MHz)

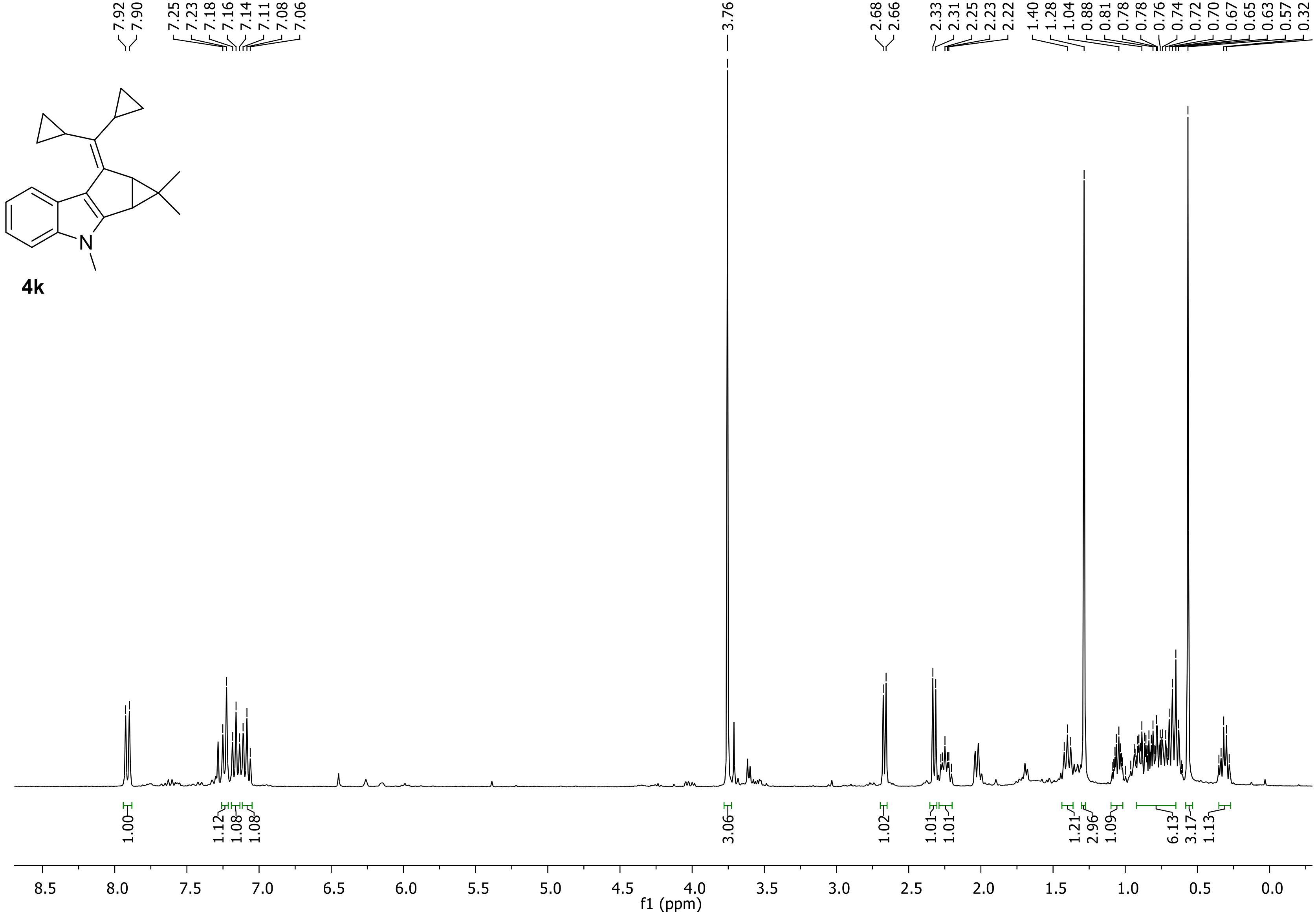

^{13}C NMR ($CDCl_3$, 75.4 MHz)

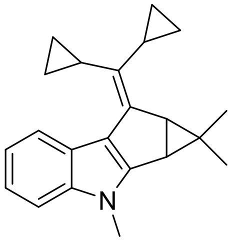

4k

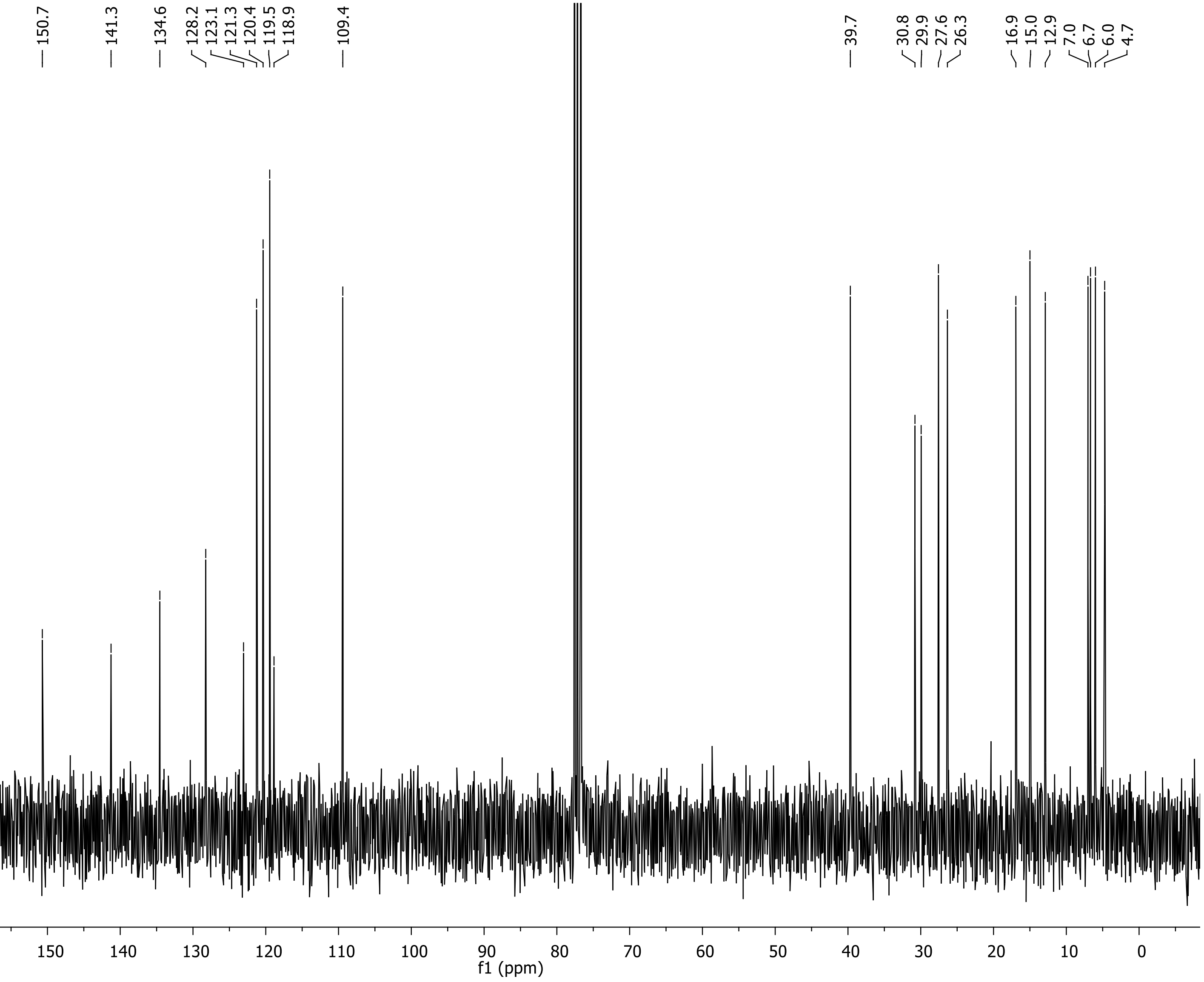

^{1}H NMR ($CDCl_3$, 300 MHz)

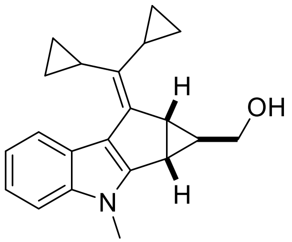

***cis*-4l**

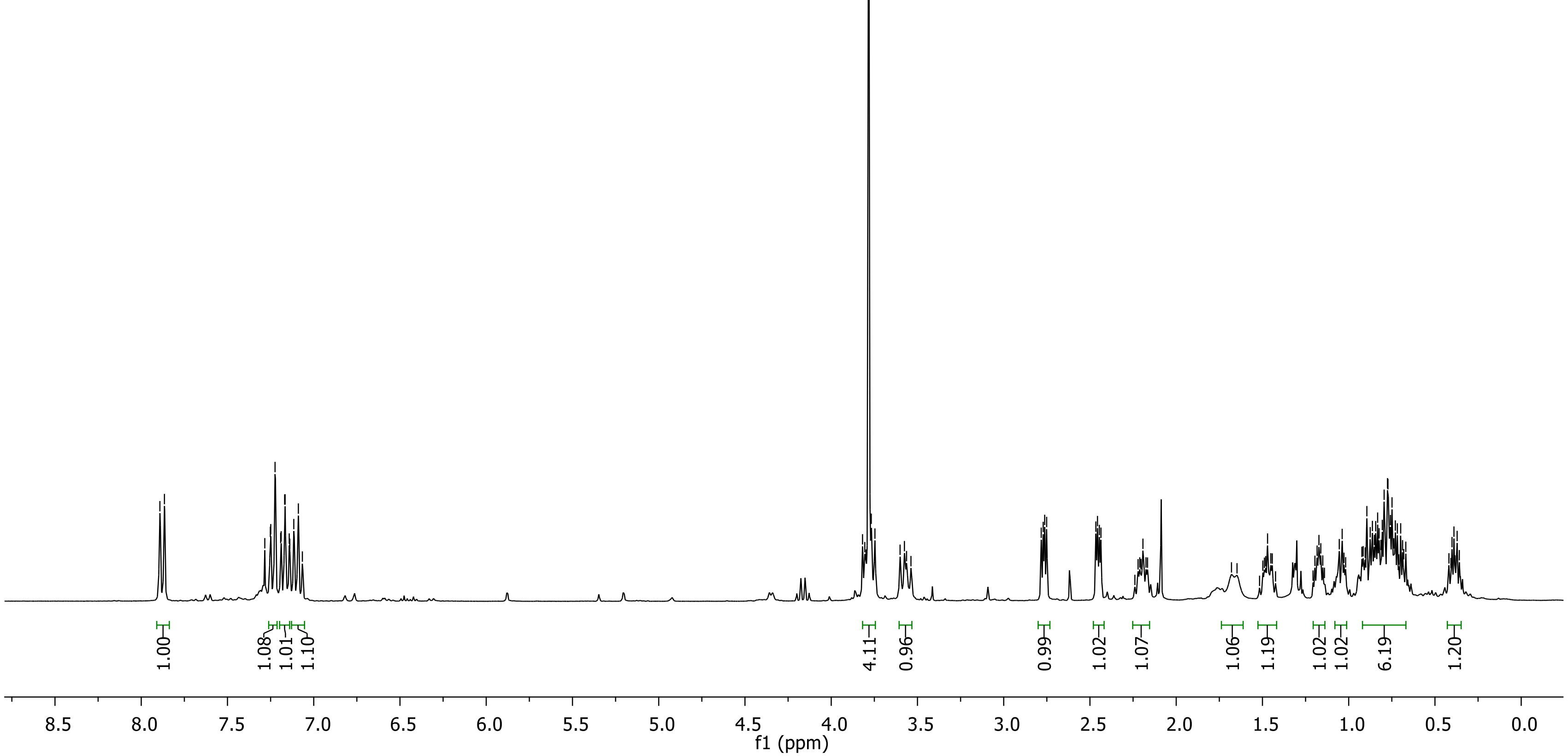

^{13}C NMR ($CDCl_3$, 75.4 MHz)

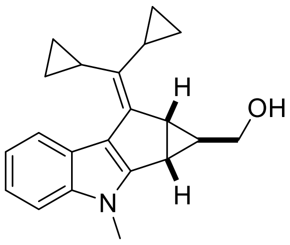

***cis*-4l**

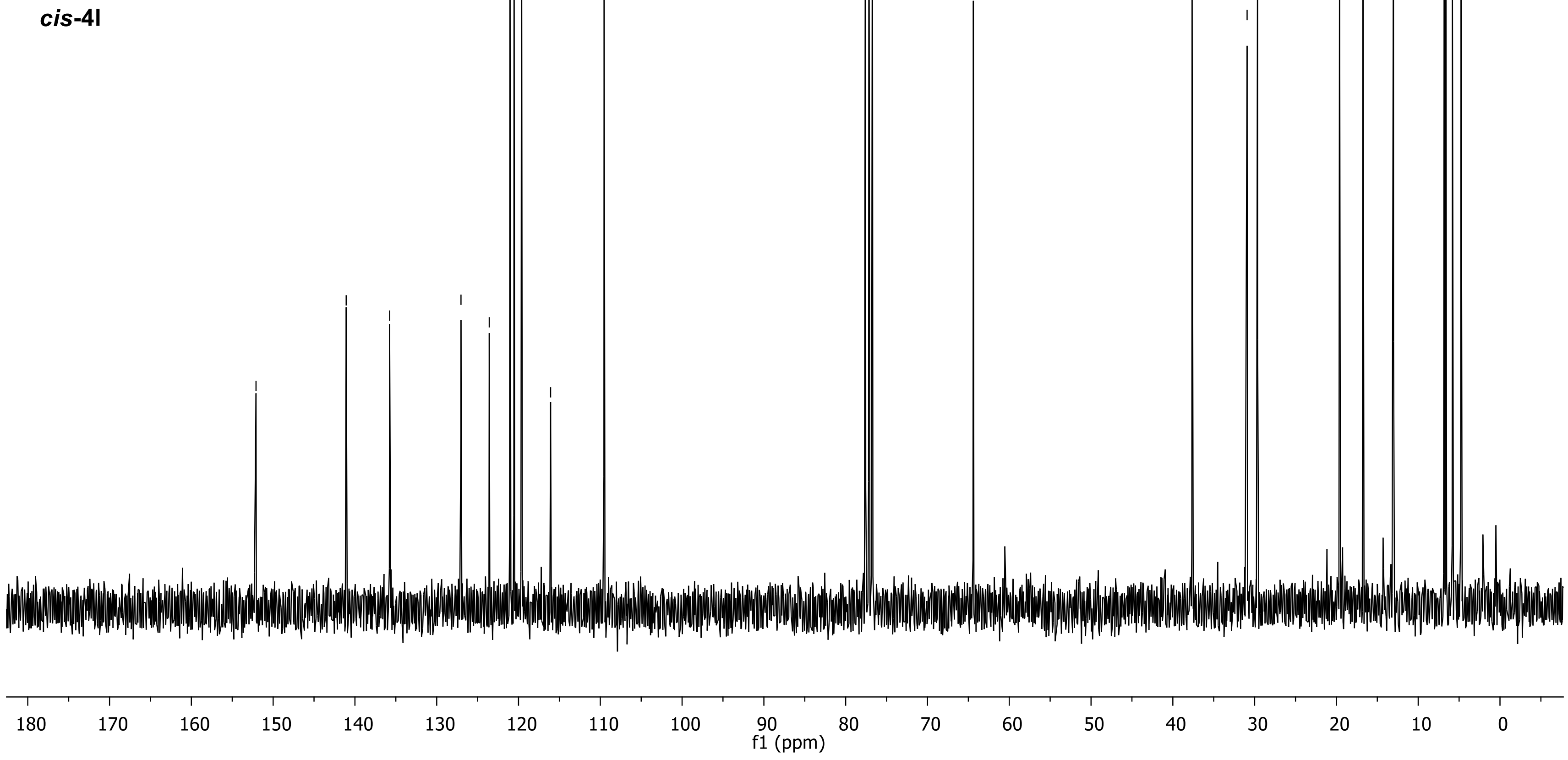

^{1}H NMR ($CDCl_3$, 300 MHz)

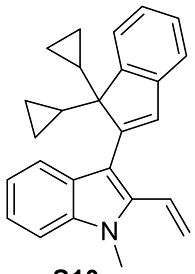

S10

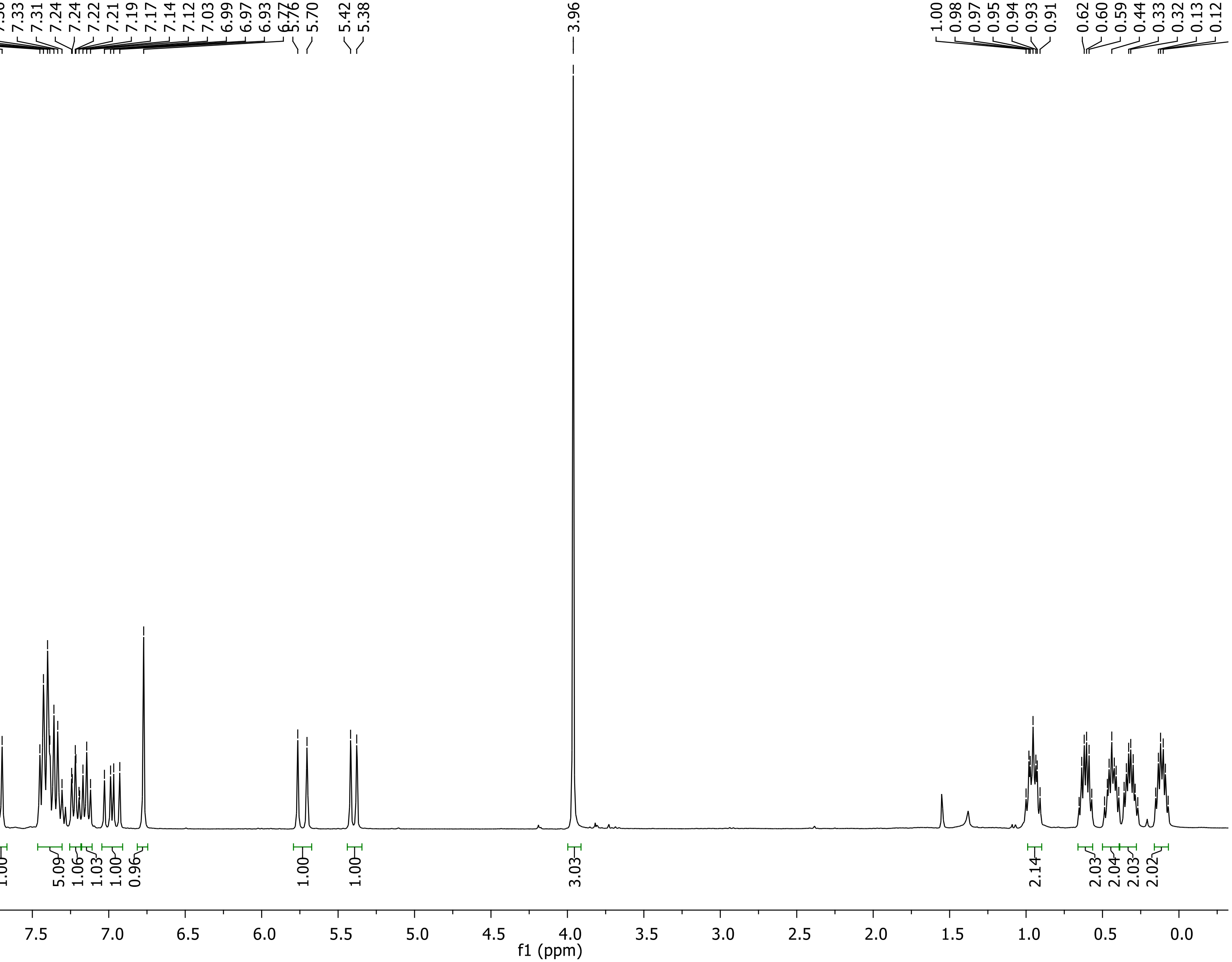

^{13}C NMR ($CDCl_3$, 75.4 MHz)

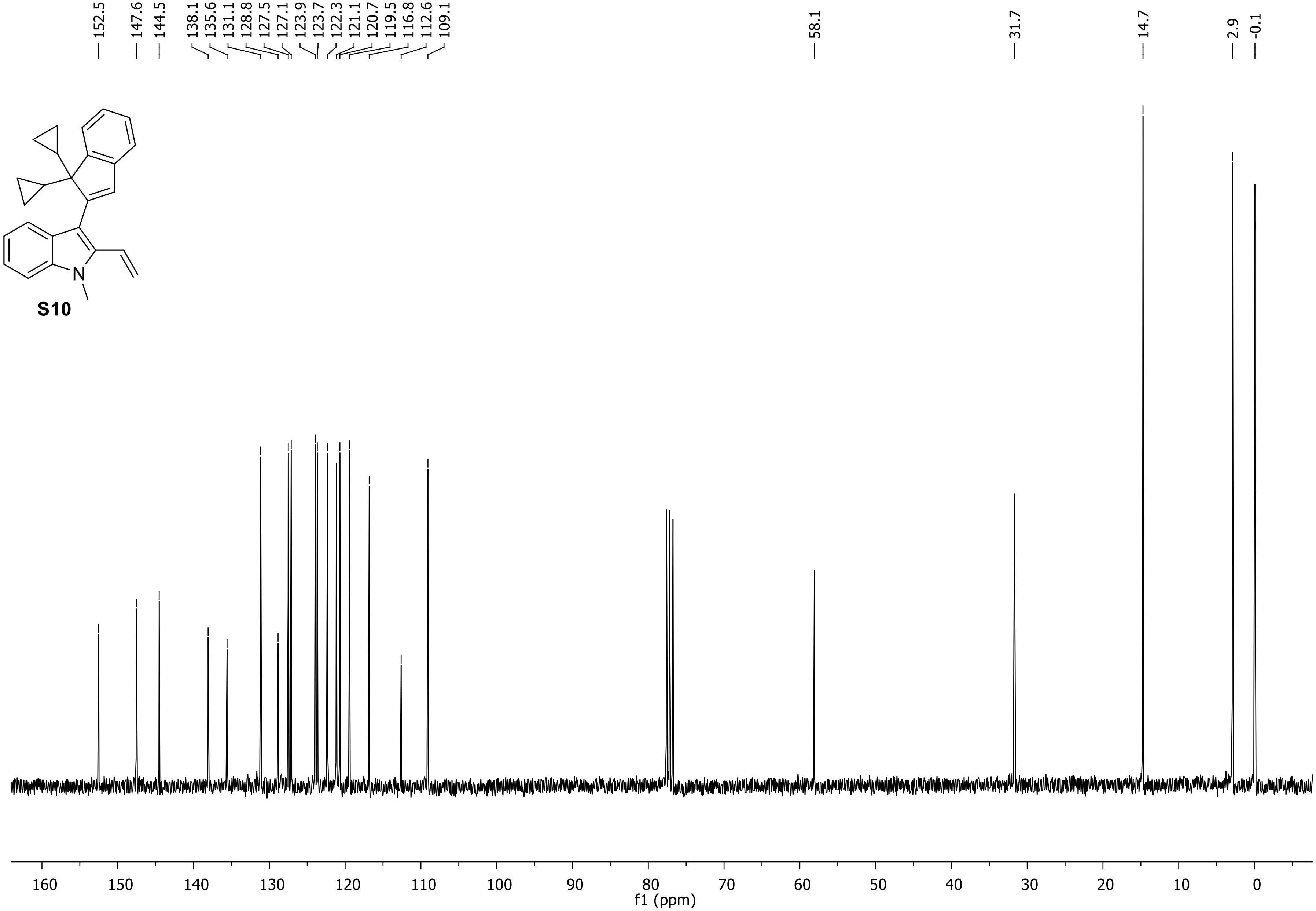

S10

^{1}H NMR ($CDCl_3$, 300 MHz)

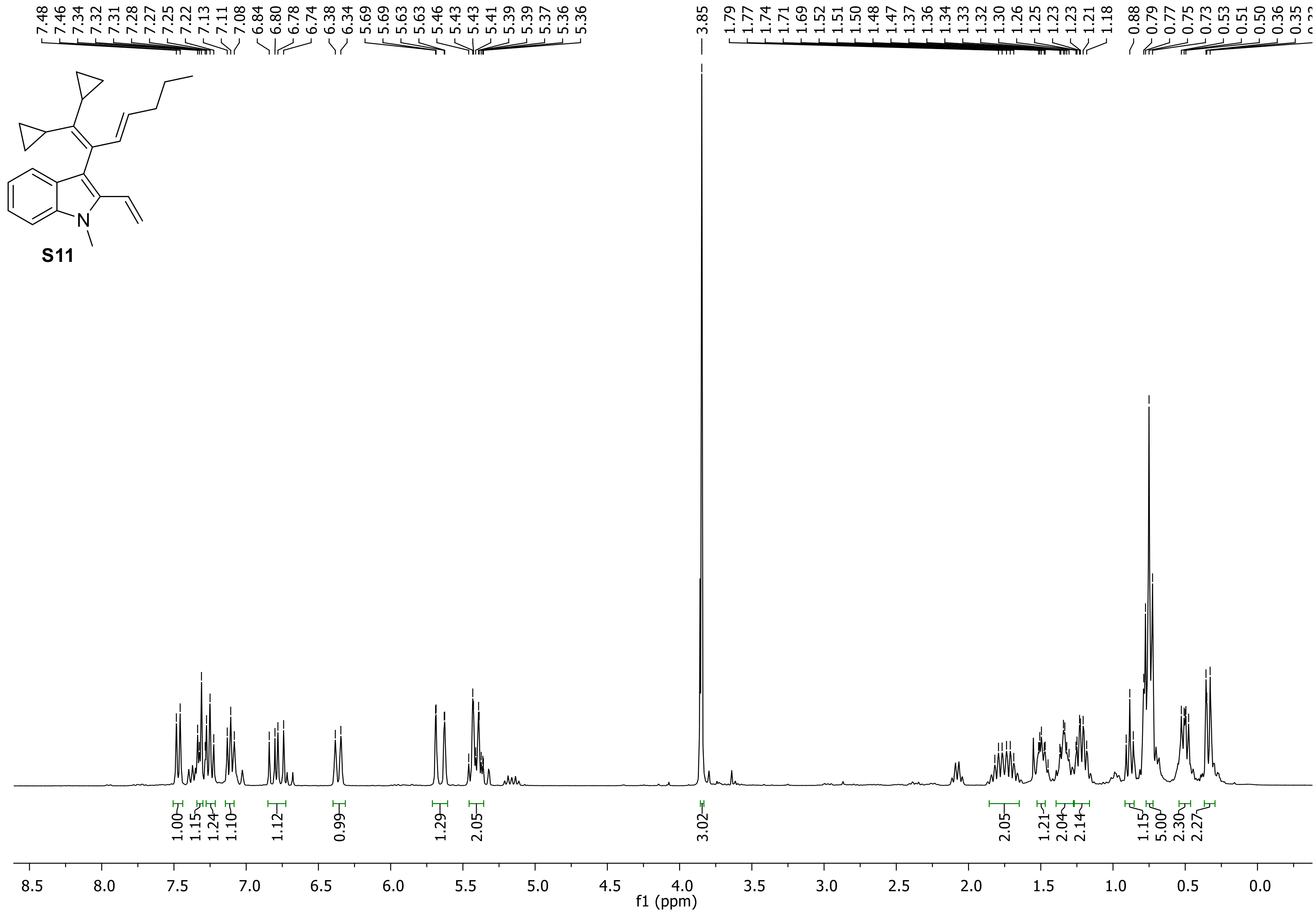

^{13}C NMR ($CDCl_3$, 75.4 MHz)

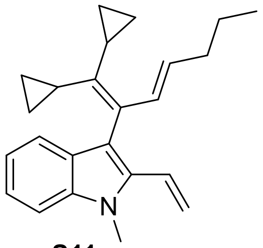

S11

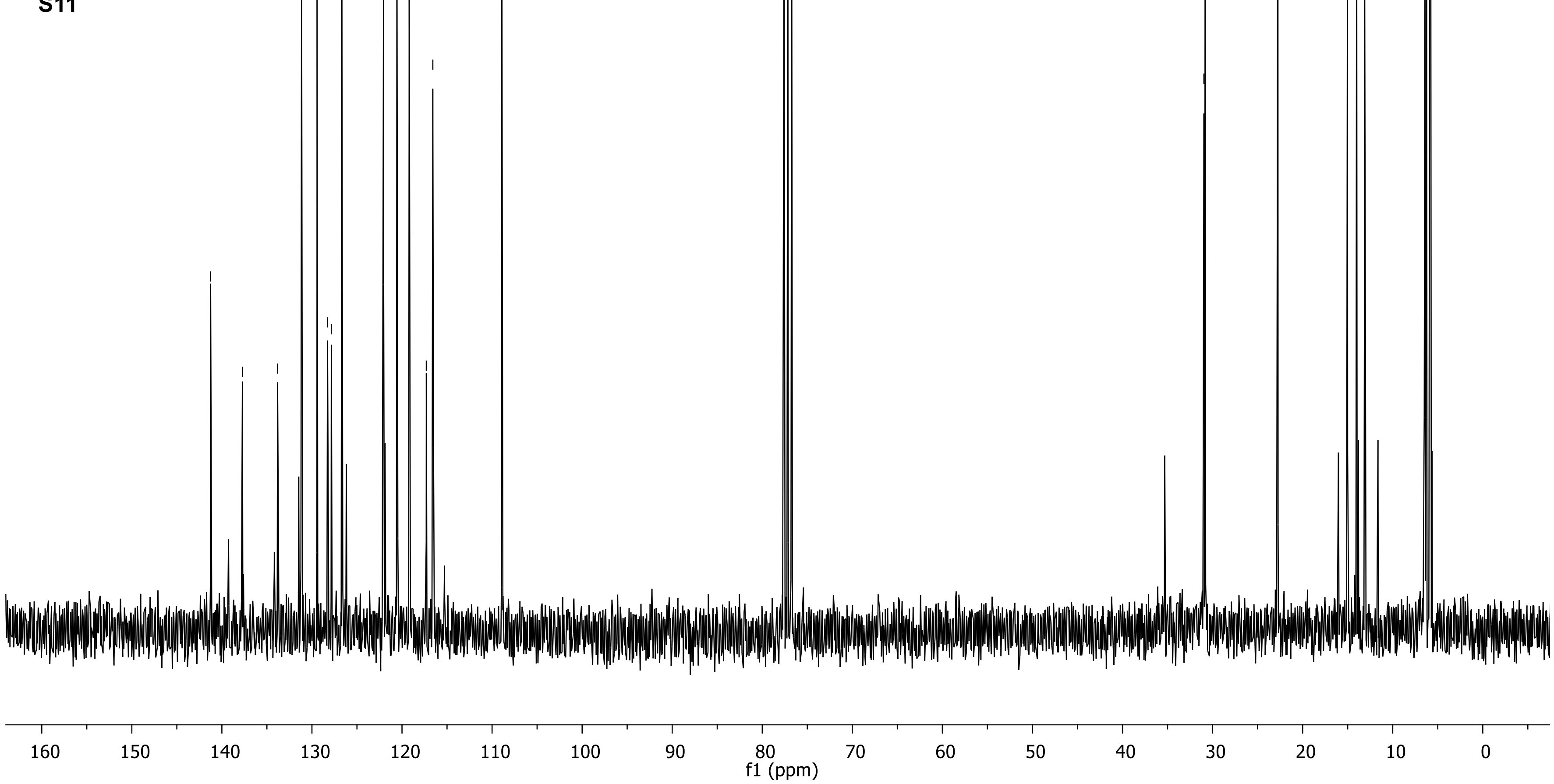

^{1}H NMR ($CDCl_3$, 300 MHz)

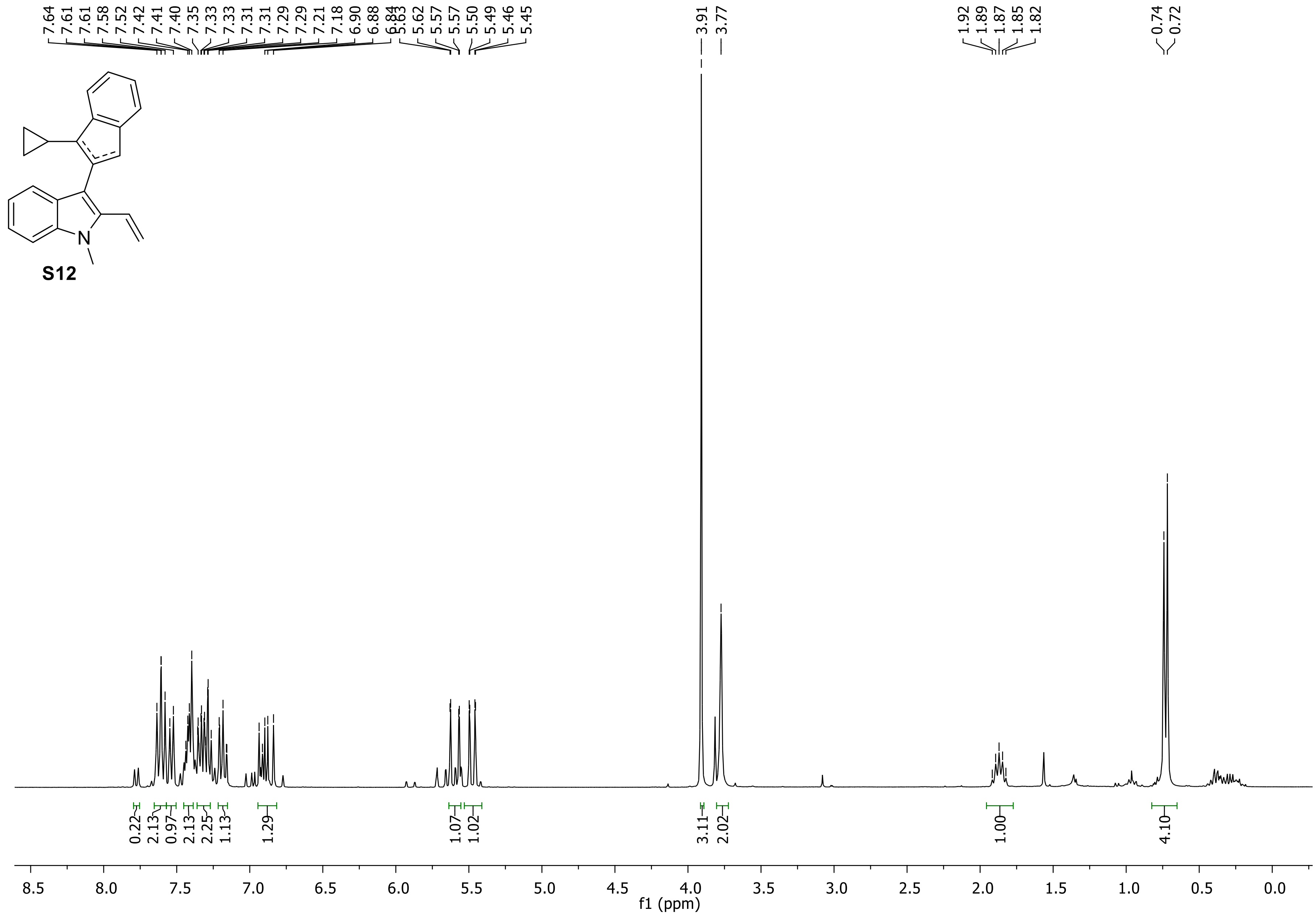

^{13}C NMR ($CDCl_3$, 75.4 MHz)

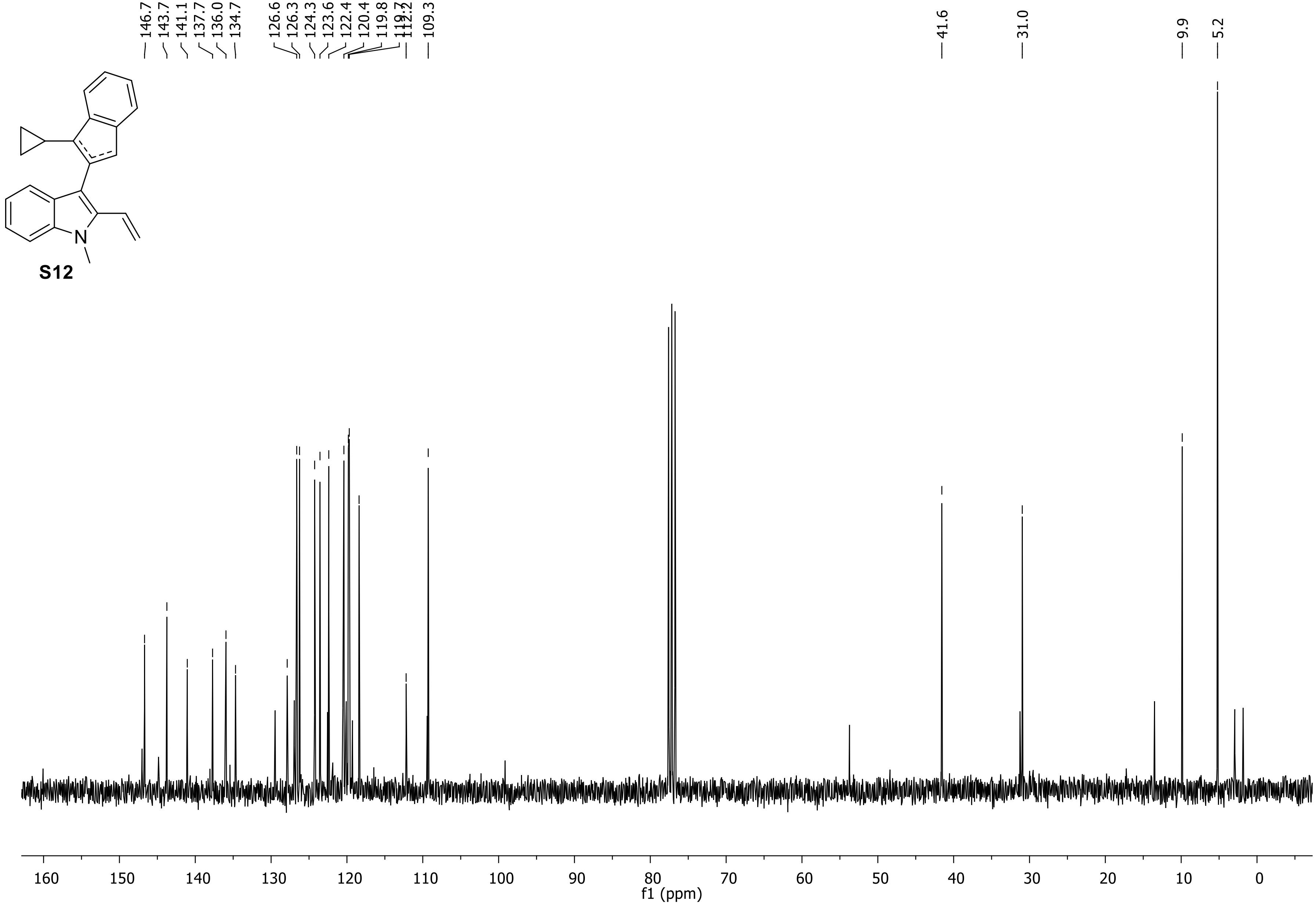

S12

^{1}H NMR ($CDCl_3$, 300 MHz)

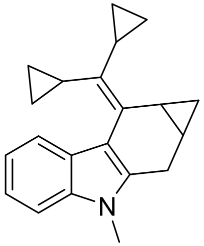

6

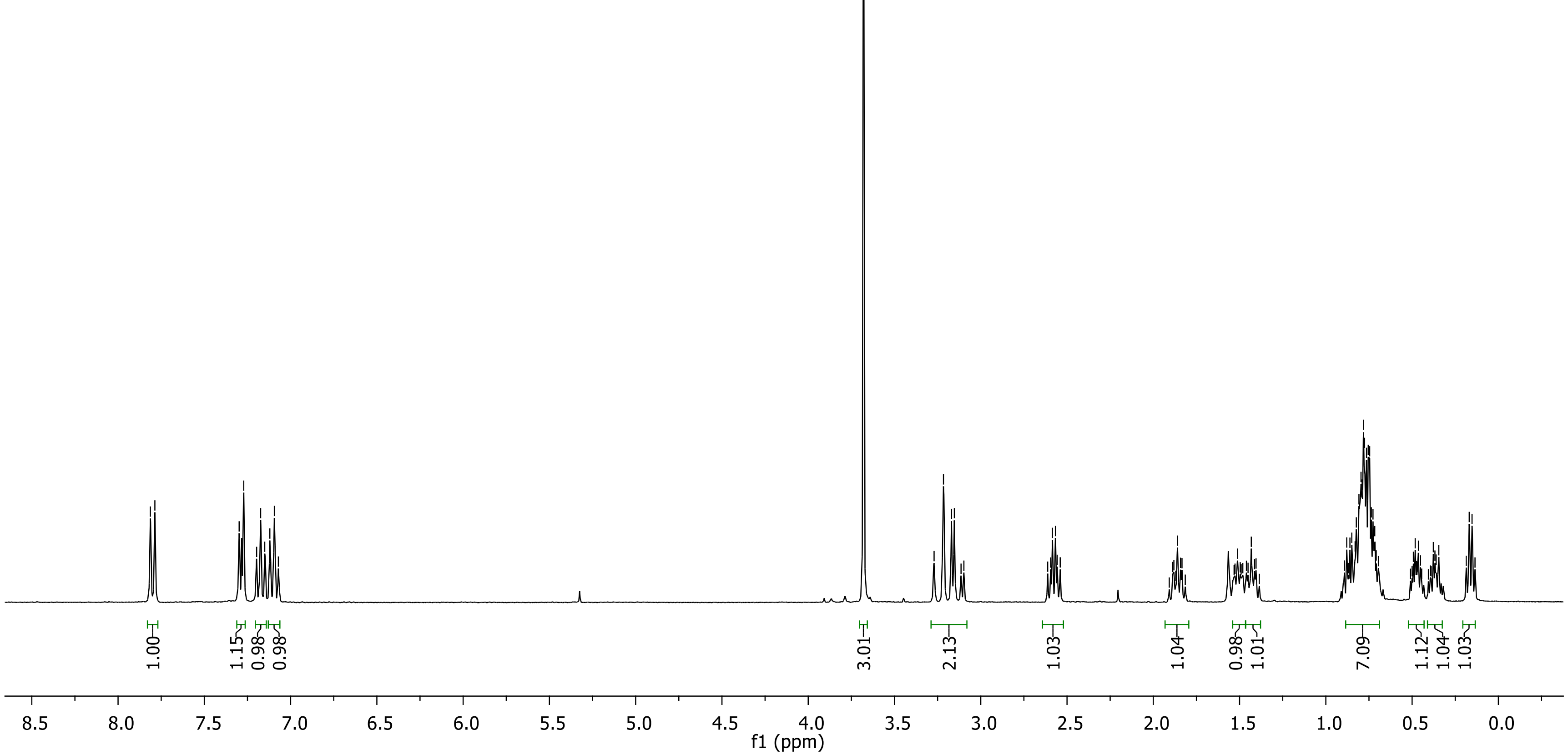

^{13}C NMR ($CDCl_3$, 75.4 MHz)

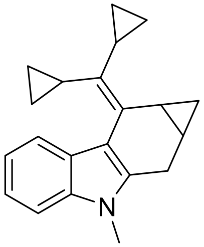

6

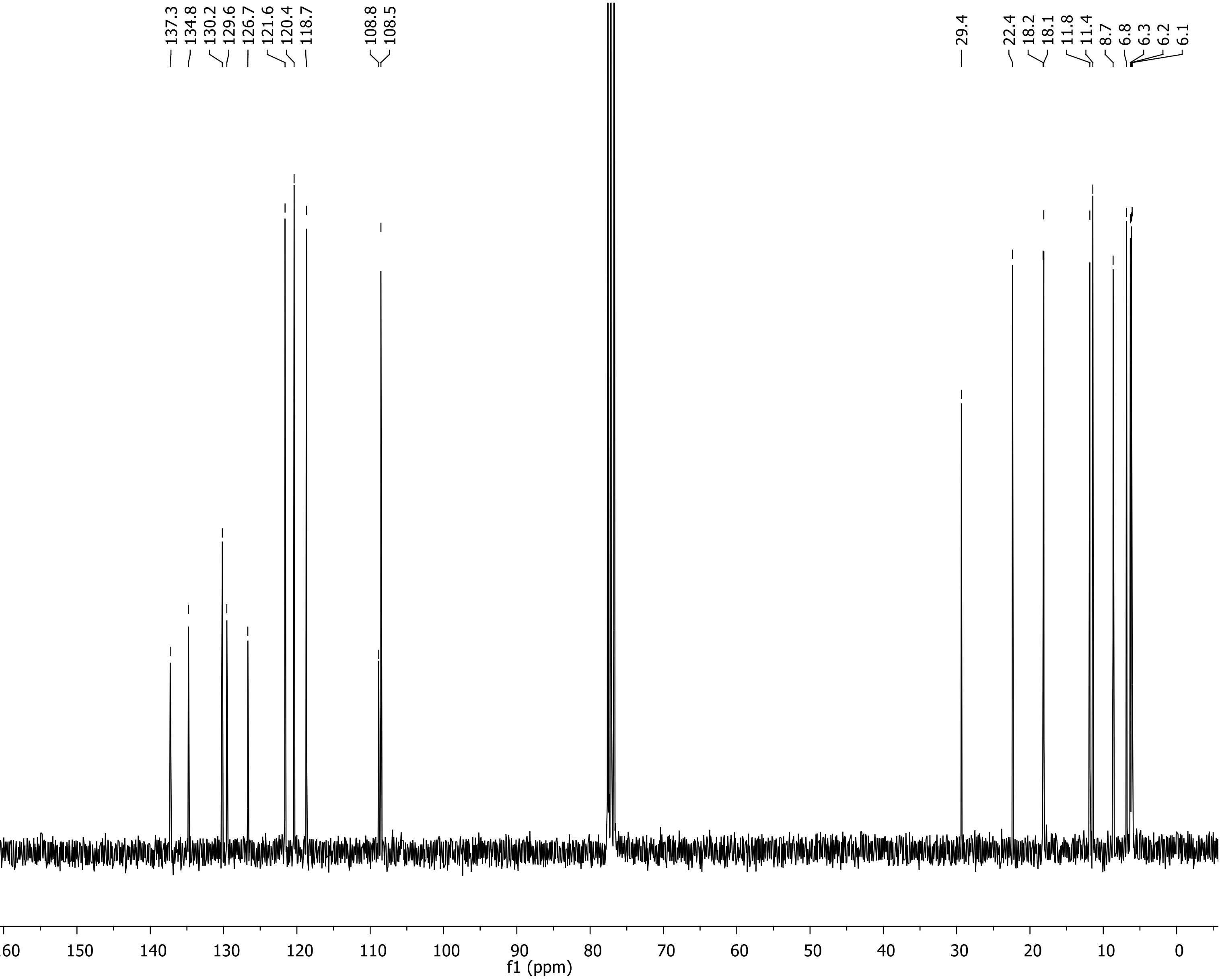

^{1}H NMR ($CDCl_3$, 300 MHz)

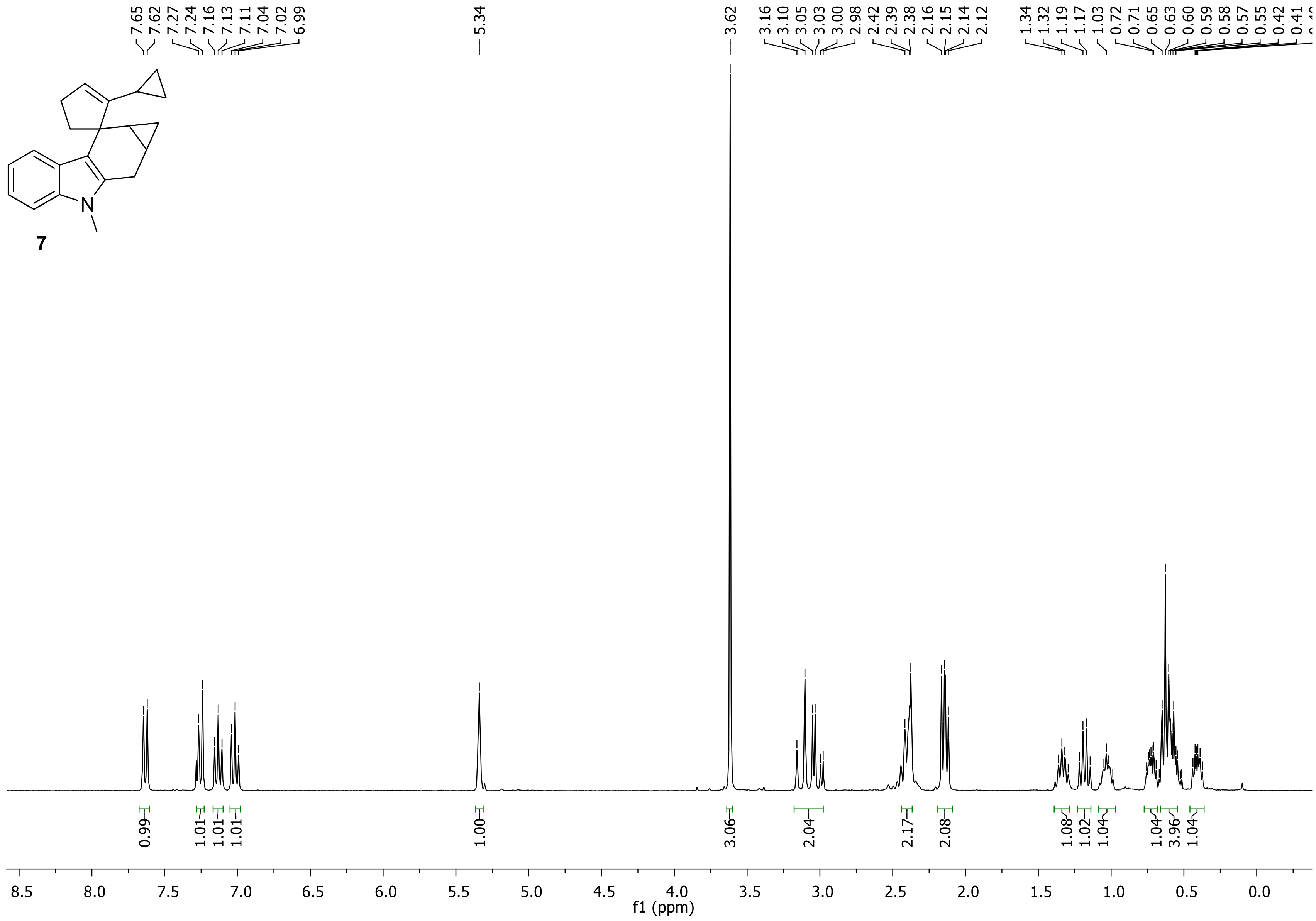

7

^{13}C NMR (CDCl$_3$, 75.4 MHz)

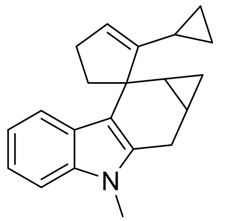

7

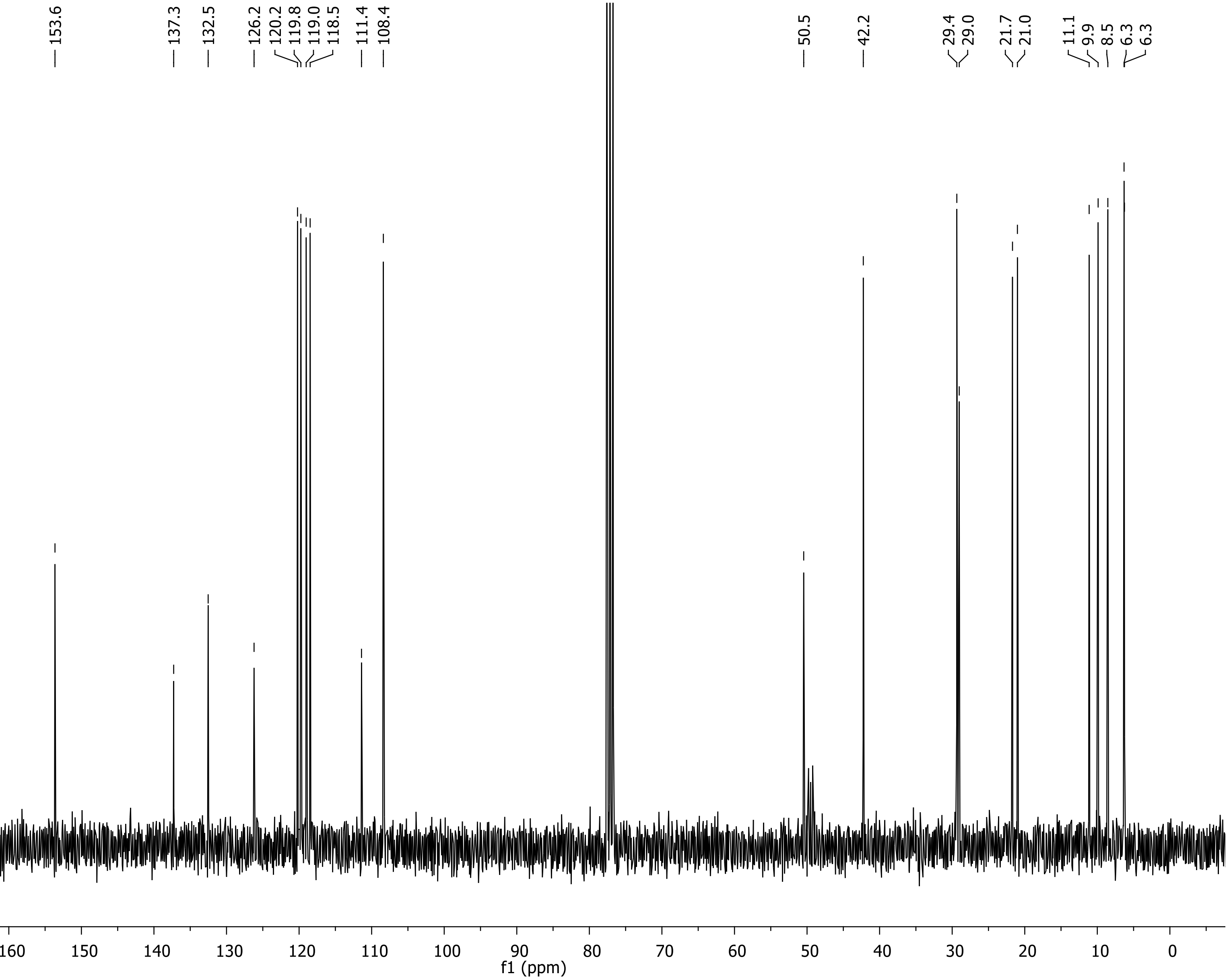

^{1}H NMR ($CDCl_3$, 300 MHz)

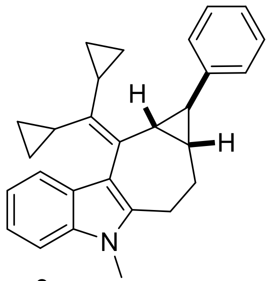

9a

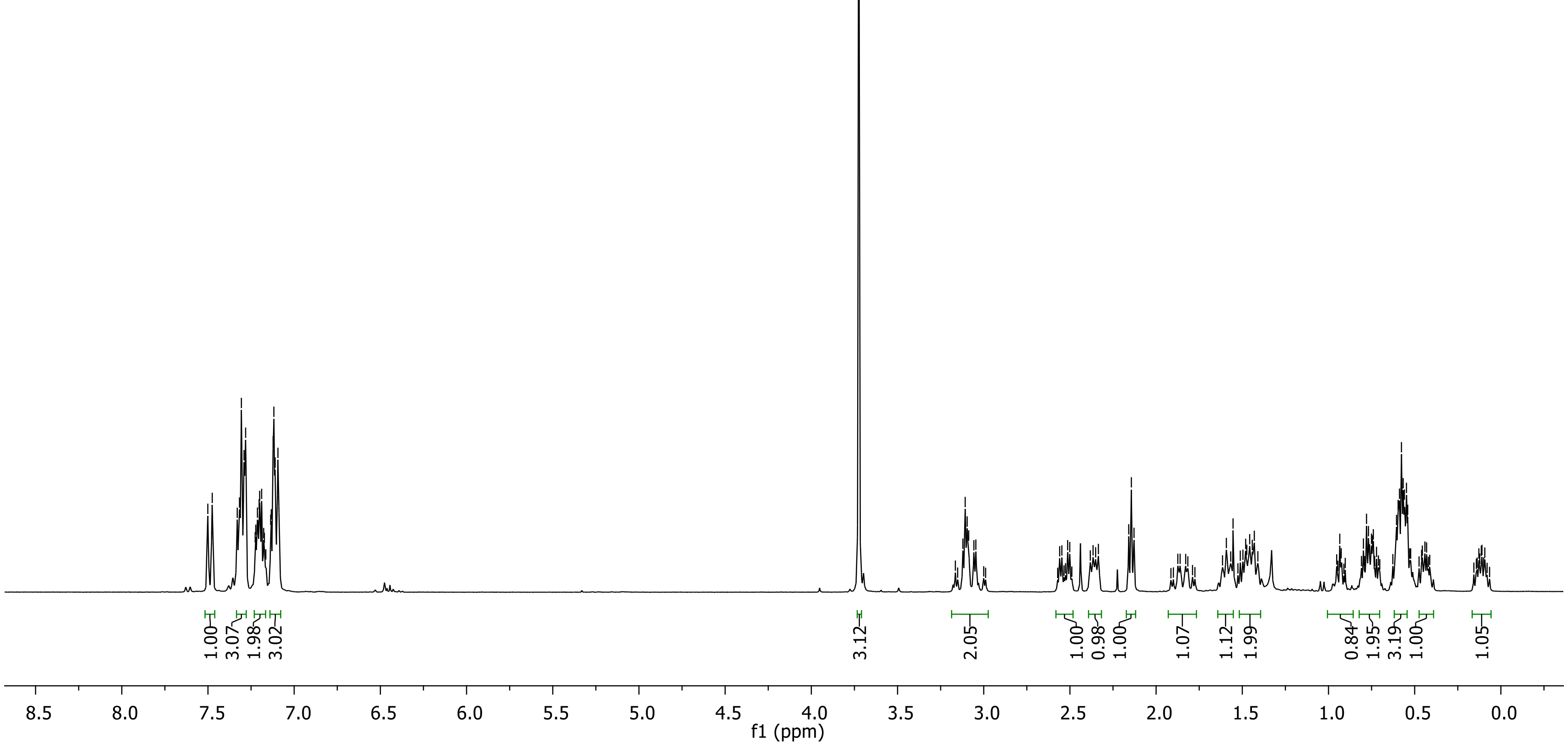

^{13}C NMR ($CDCl_3$, 75.4 MHz)

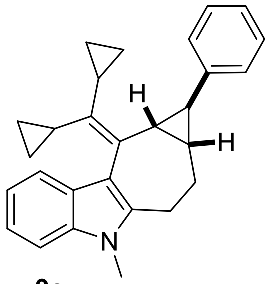

9a

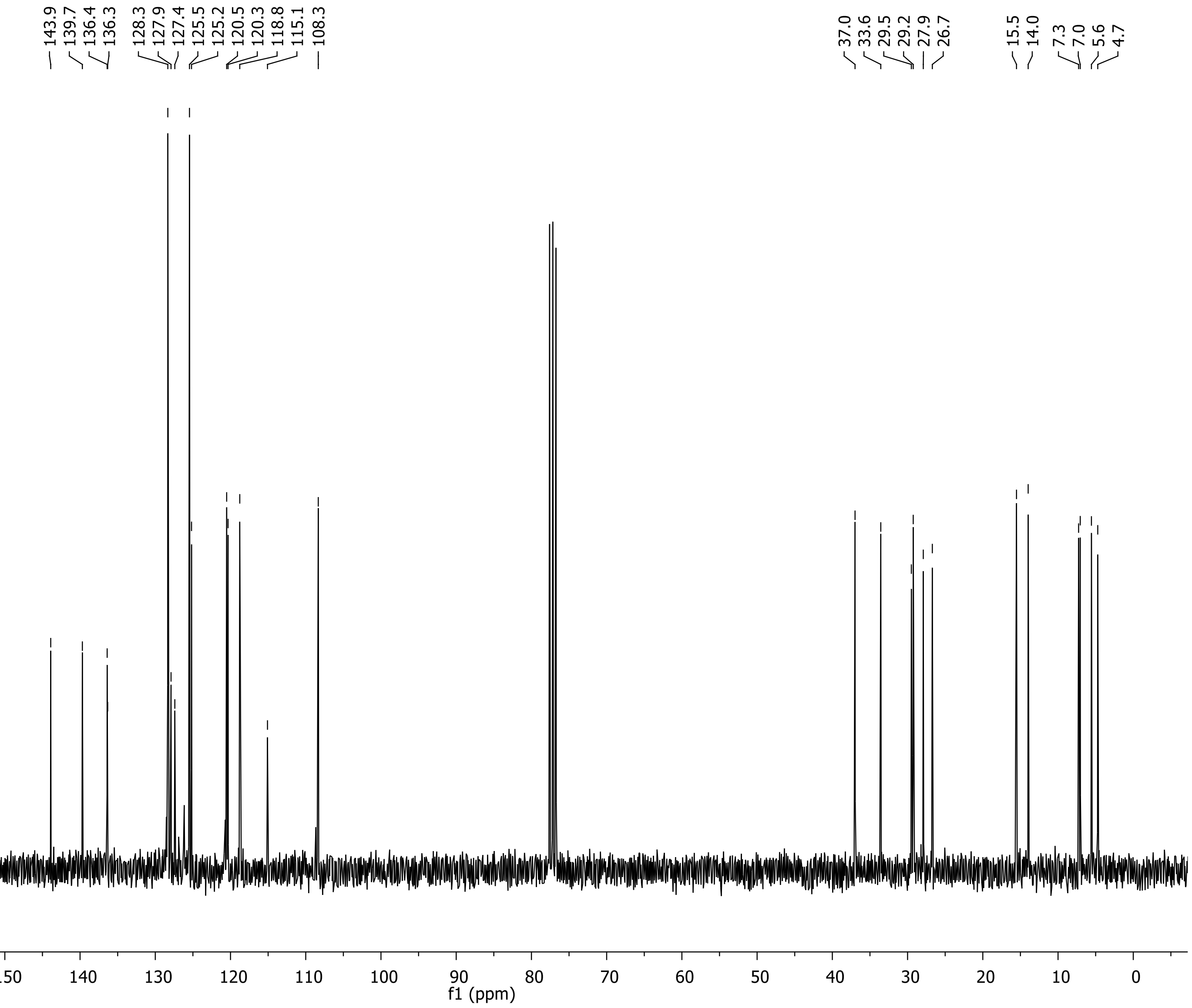

^{1}H NMR ($CDCl_3$, 300 MHz)

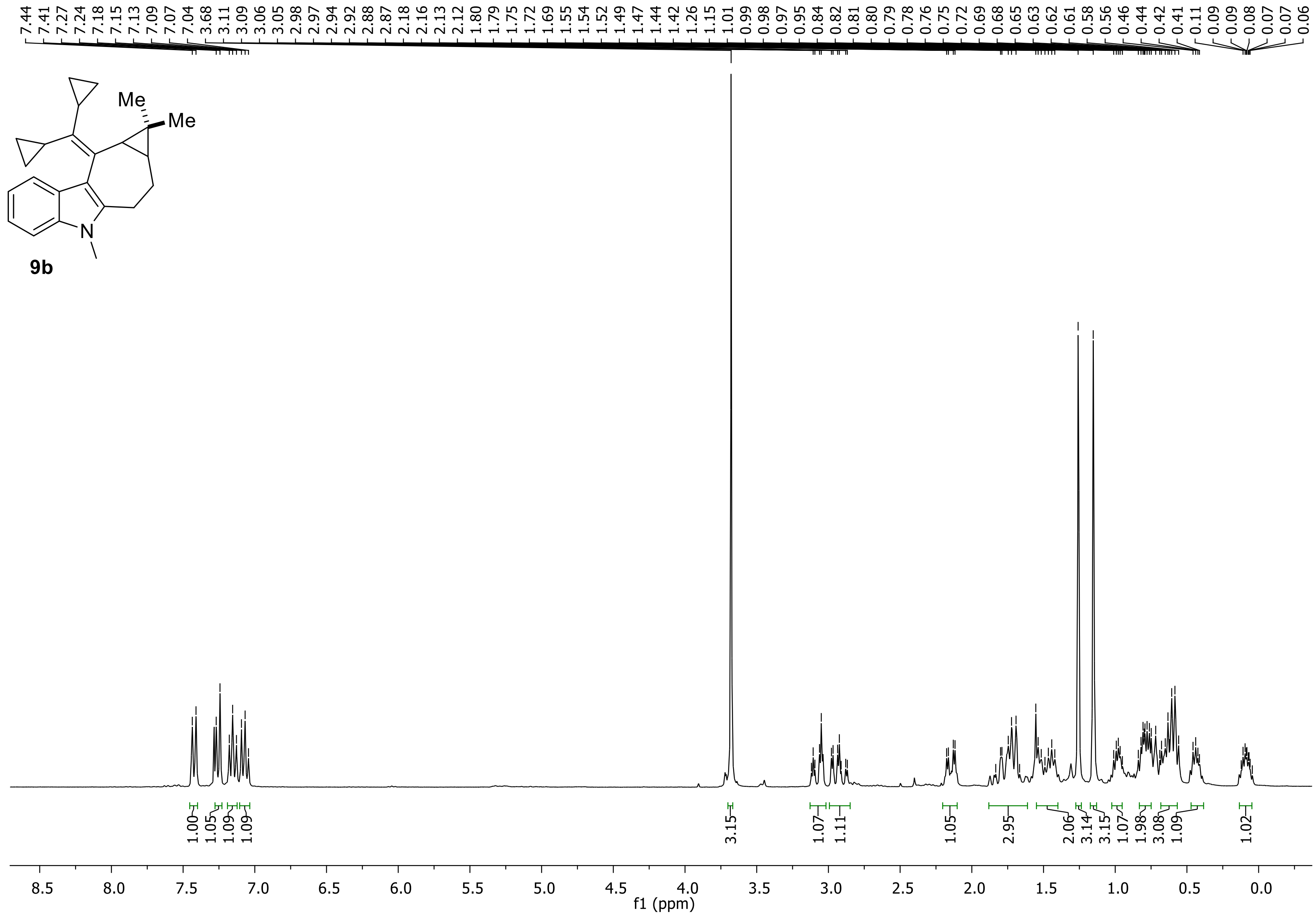

9b

^{13}C NMR ($CDCl_3$, 75.4 MHz)

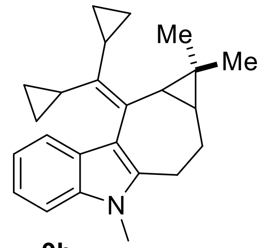

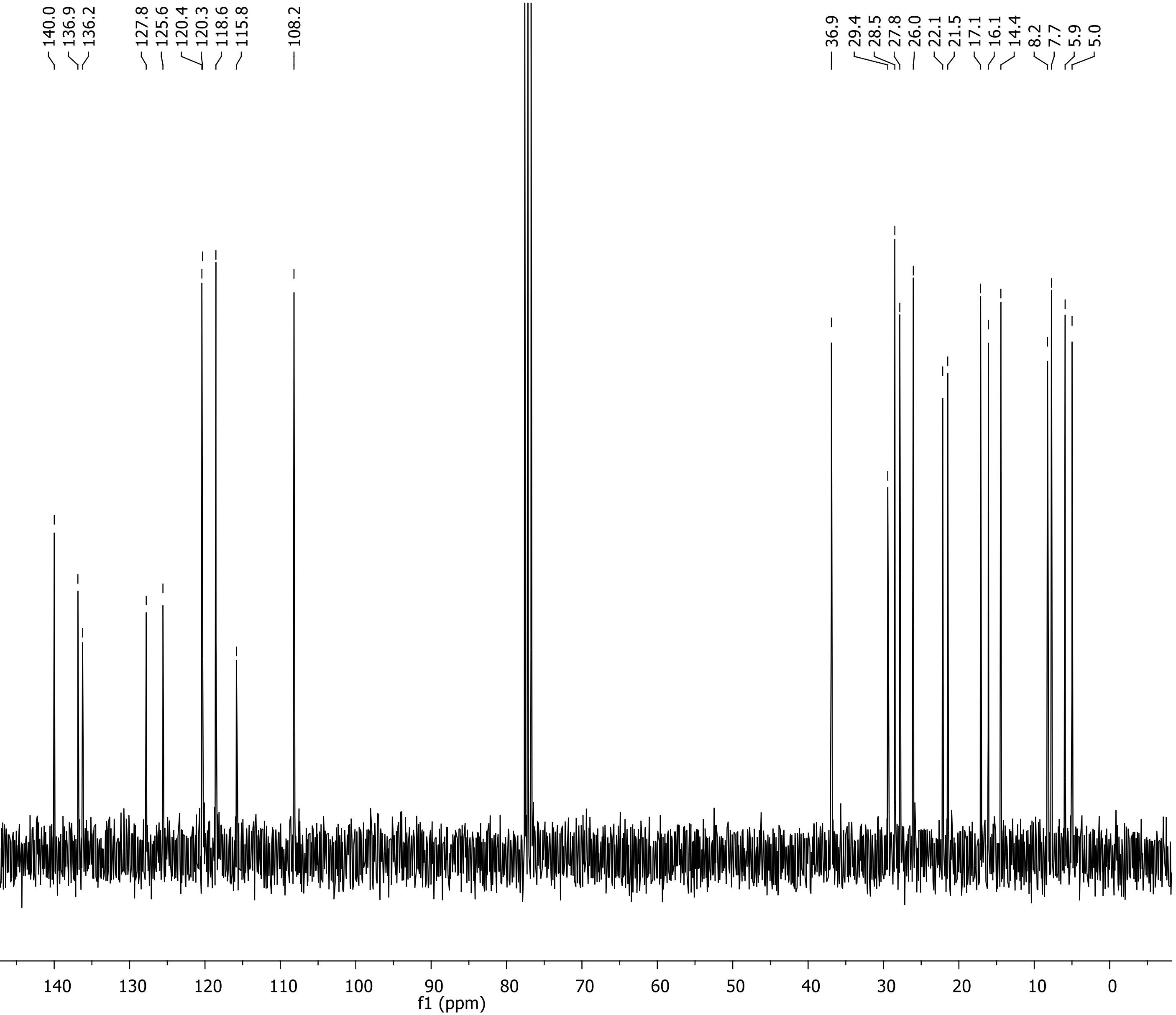

^{1}H NMR ($CDCl_3$, 300 MHz)

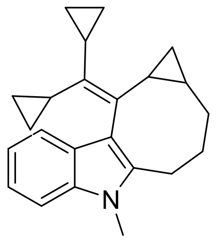

9c

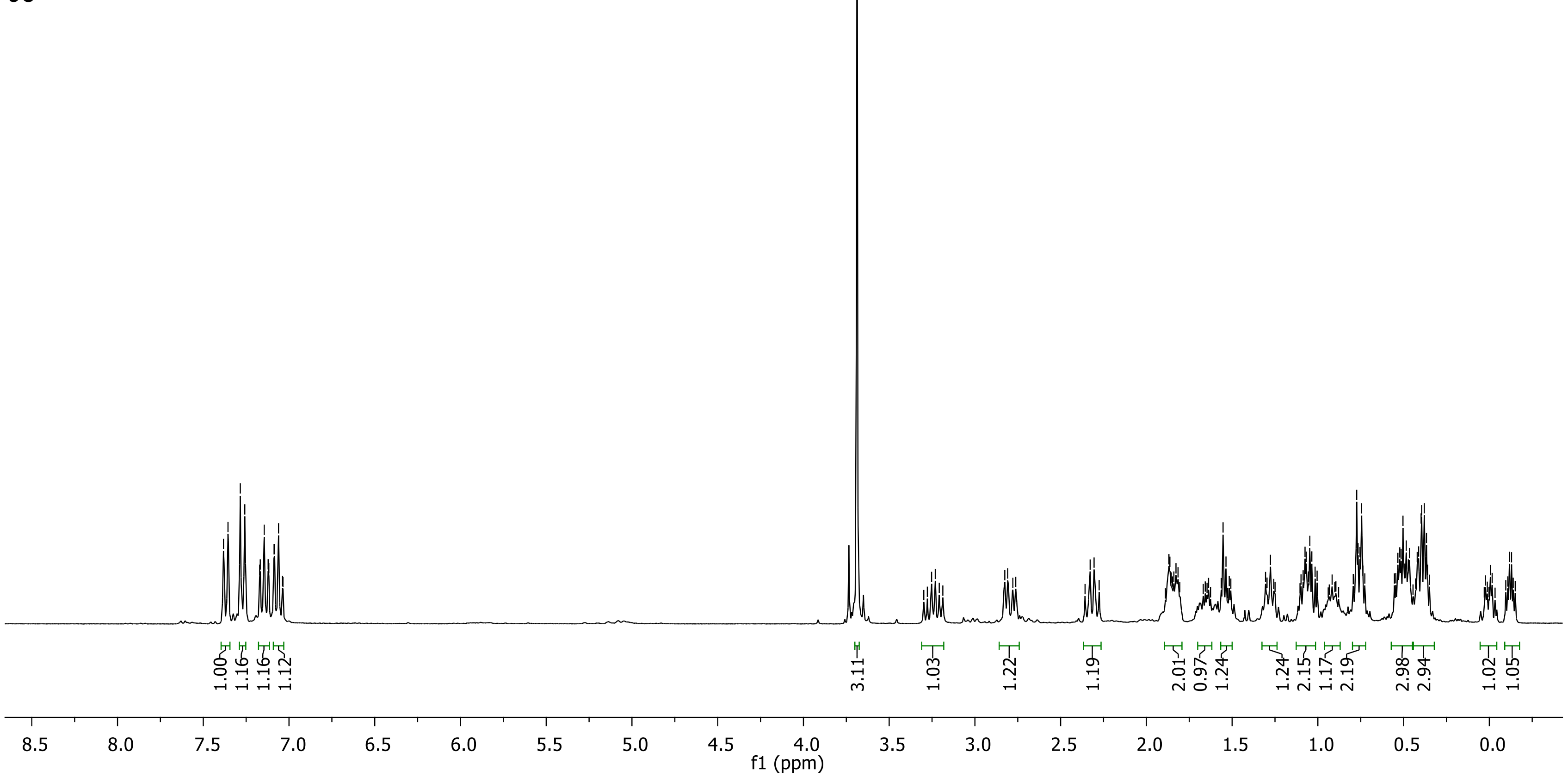

^{13}C NMR ($CDCl_3$, 75.4 MHz)

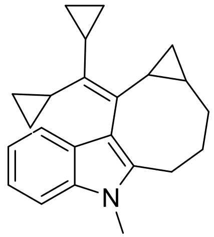

9c

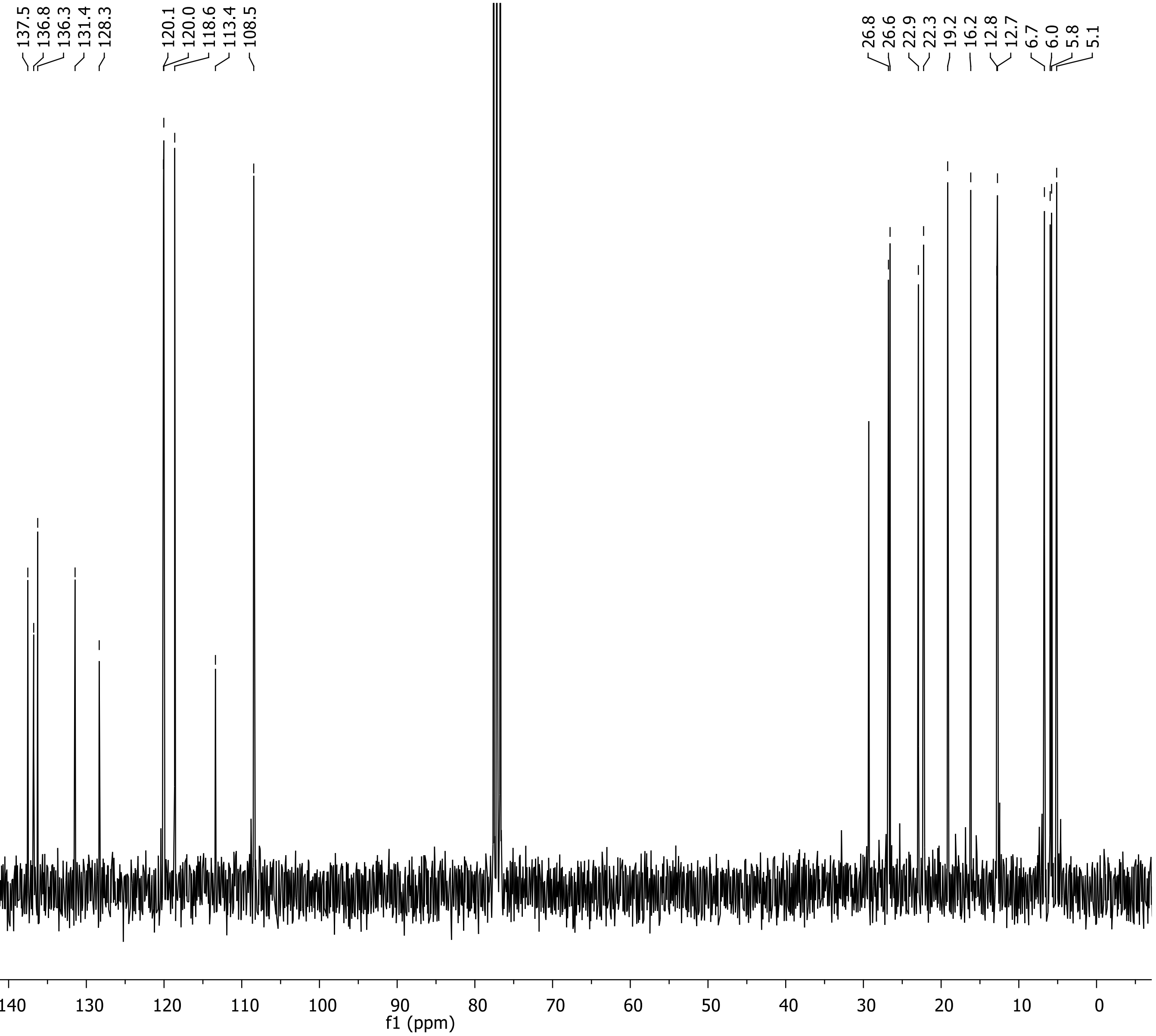

Supplement: Supplementary file 2 [file ol5c02596_si_002.pdf]
