# Supplementary material for: Gold(I)-Catalyzed Tandem 1,2-Indole Migration–Cyclopropanation Reactions of 3‑Propargylindoles with Olefins
Source: Org Lett. 2025 Jul 28;27(31):8662–7. doi: 10.1021/acs.orglett.5c02596 (PMC12340974; doi:10.1021/acs.orglett.5c02596)
Supplement: Supplementary file 1 [file ol5c02596_si_001.zip › trans-2a/HNMR/pdata/1/email_LR-766-F2_10_1.pdf]

PCT de Universidad de Burgos
H1
PCT-D01-PROTON CDCl3 {D:\PCT} PCT 7

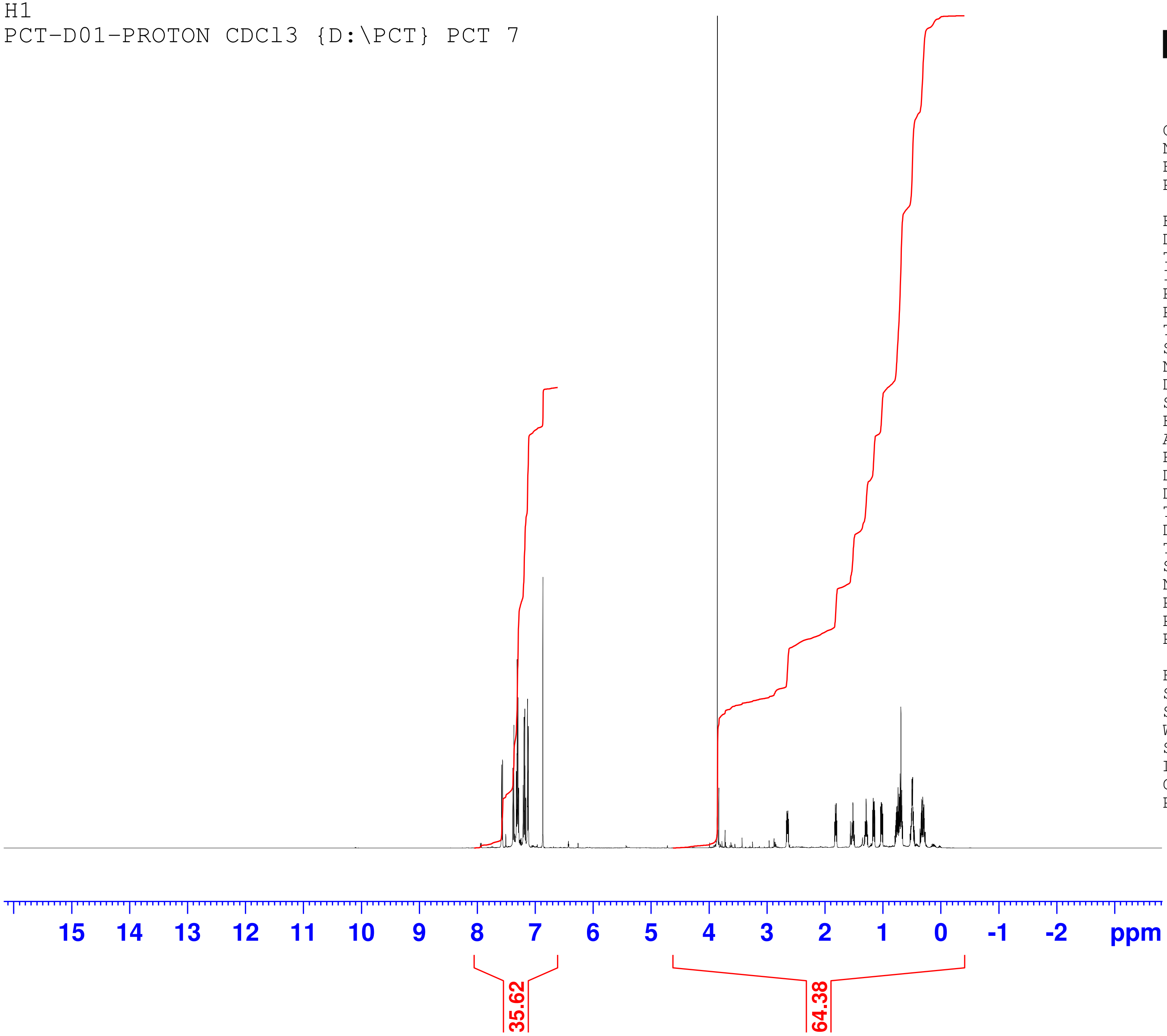

Current Data Parameters

NAME	LR-766-F2
EXPNO	10
PROCNO	1

F2 - Acquisition Parameters

Date_	20240611
Time	8.56 h
INSTRUM	AV NEO
PROBHD	Z151574_0146 (
PULPROG	zg30
TD	65536
SOLVENT	CDCl3
NS	16
DS	2
SWH	10000.000 Hz
FIDRES	0.305176 Hz
AQ	3.2767999 sec
RG	40.3
DW	50.000 usec
DE	12.14 usec
TE	298.0 K
D1	1.00000000 sec
TD0	1
SFO1	500.1630885 MHz
NUC1	1H
P0	2.67 usec
P1	8.00 usec
PLW1	25.70299911 W

F2 - Processing parameters

SI	65536
SF	500.1600000 MHz
WDW	EM
SSB	0
LB	0.30 Hz
GB	0
PC	1.00
