# Supplementary material for: Gold(I)-Catalyzed Tandem 1,2-Indole Migration–Cyclopropanation Reactions of 3‑Propargylindoles with Olefins
Source: Org Lett. 2025 Jul 28;27(31):8662–7. doi: 10.1021/acs.orglett.5c02596 (PMC12340974; doi:10.1021/acs.orglett.5c02596)
Supplement: Supplementary file 1 [file ol5c02596_si_001.zip › trans-2c/CNMR/pdata/1/email_LR-831-F1_20_1.pdf]

PCT de Universidad de Burgos
C13
PCT-D02-C13 CDCl3 {D:\PCT} PCT 3

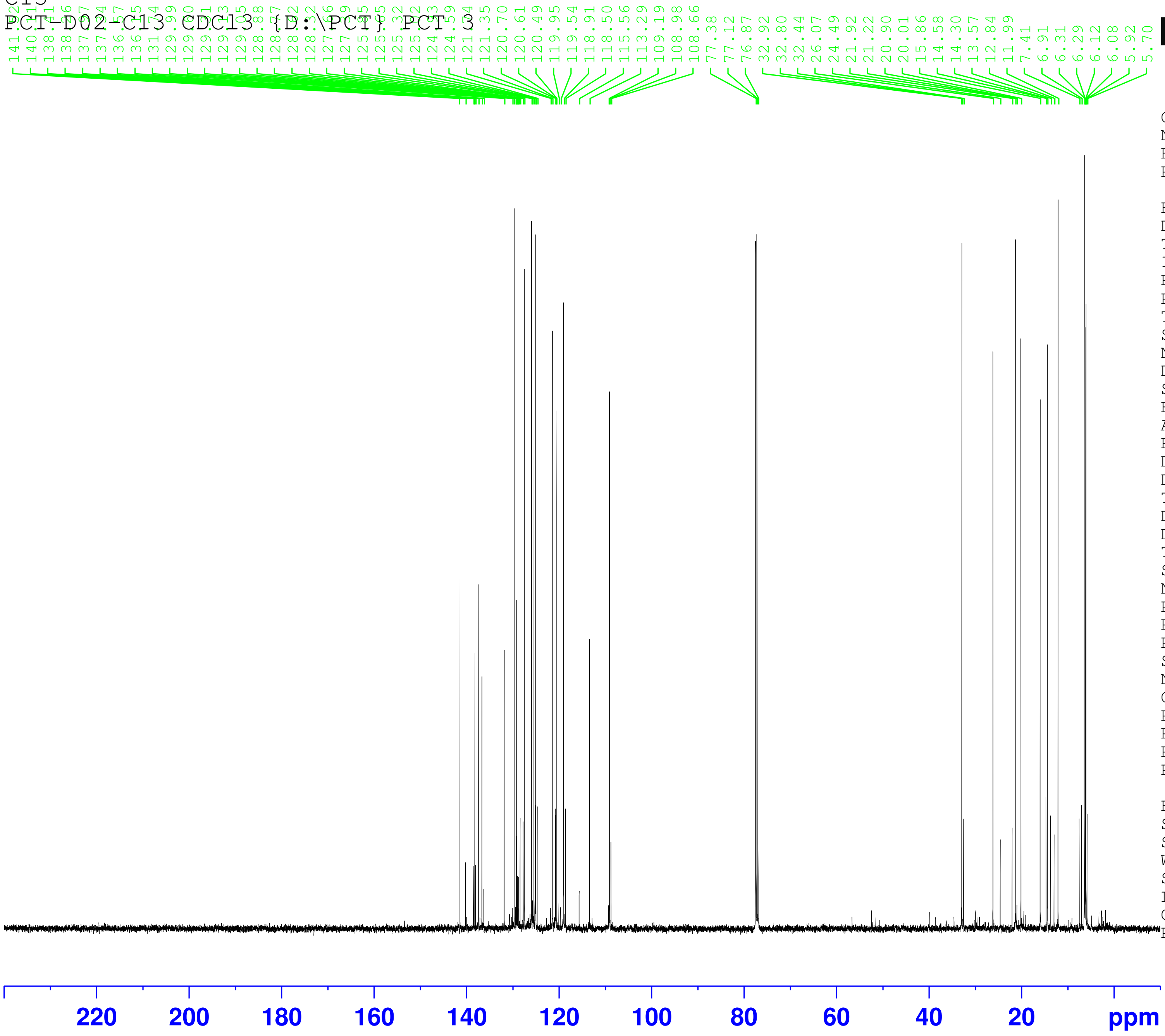


BRUKER

Current Data Parameters

| | | |
|---|---|---|
| NAME | LR-831-F1 | |
| EXPNO | 20 | |
| PROCNO | 1 | |

F2 - Acquisition Parameters

| | | |
|---|---|---|
| Date_ | 20240924 | |
| Time | 16.25 | h |
| INSTRUM | AV NEO | |
| PROBHD | Z151574_0146 ( | |
| PULPROG | zgpg30 | |
| TD | 65536 | |
| SOLVENT | CDCl3 | |
| NS | 512 | |
| DS | 4 | |
| SWH | 32679.739 | Hz |
| FIDRES | 0.997306 | Hz |
| AQ | 1.0027008 | sec |
| RG | 101 | |
| DW | 15.300 | usec |
| DE | 6.50 | usec |
| TE | 298.0 | K |
| D1 | 2.00000000 | sec |
| D11 | 0.03000000 | sec |
| TD0 | 1 | |
| SFO1 | 125.7791662 | MHz |
| NUC1 | 13C | |
| P0 | 3.00 | usec |
| P1 | 9.00 | usec |
| PLW1 | 103.68000031 | W |
| SFO2 | 500.1620006 | MHz |
| NUC2 | 1H | |
| CPDPRG2 | A000 | |
| PCPD2 | 80.00 | usec |
| PLW2 | 25.70299911 | W |
| PLW12 | 0.25703001 | W |
| PLW13 | 0.12929000 | W |

F2 - Processing parameters

| | | |
|---|---|---|
| SI | 32768 | |
| SF | 125.7653220 | MHz |
| WDW | EM | |
| SSB | 0 | |
| LB | 1.00 | Hz |
| GB | 0 | |
| PC | 1.40 | |
