# Supplementary material for: Gold(I)-Catalyzed Tandem 1,2-Indole Migration–Cyclopropanation Reactions of 3‑Propargylindoles with Olefins
Source: Org Lett. 2025 Jul 28;27(31):8662–7. doi: 10.1021/acs.orglett.5c02596 (PMC12340974; doi:10.1021/acs.orglett.5c02596)
Supplement: Supplementary file 1 [file ol5c02596_si_001.zip › trans-2d/CNMR/pdata/1/email_LR-830-F1_20_1.pdf]

PCT de Universidad de Burgos
C13
PCT-D02-C13 CDCl3 {D:\PCT} PCT 2

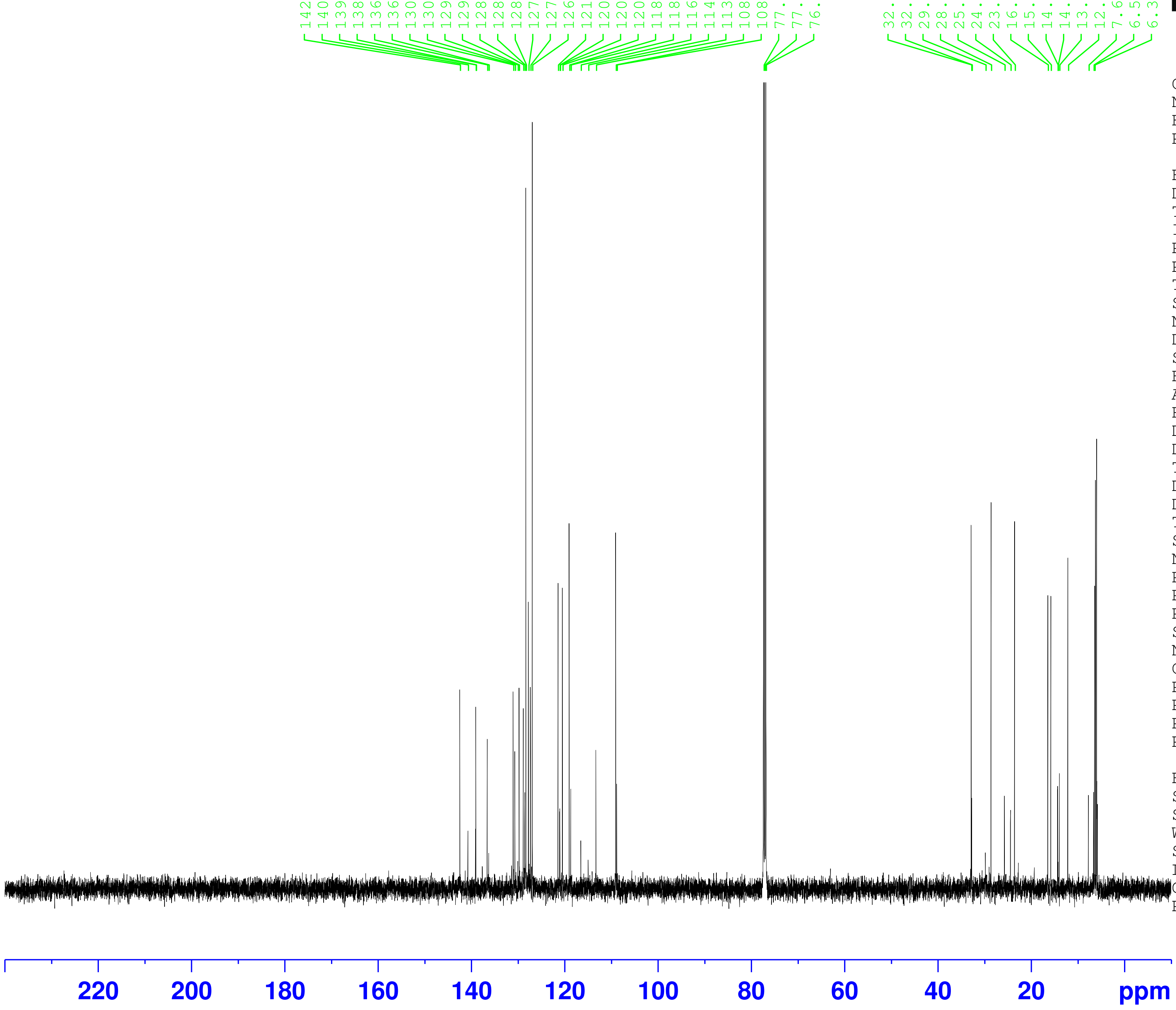

BRUKER

Current Data Parameters
NAME LR-830-F1
EXPNO 20
PROCNO 1

F2 - Acquisition Parameters
Date_ 20240924
Time 15.49 h
INSTRUM AV NEO
PROBHD Z151574_0146 (
PULPROG zgpg30
TD 65536
SOLVENT CDCl3
NS 512
DS 4
SWH 32679.739 Hz
FIDRES 0.997306 Hz
AQ 1.0027008 sec
RG 101
DW 15.300 usec
DE 6.50 usec
TE 298.0 K
D1 2.00000000 sec
D11 0.03000000 sec
TD0 1
SFO1 125.7791662 MHz
NUC1 13C
P0 3.00 usec
P1 9.00 usec
PLW1 103.68000031 W
SFO2 500.1620006 MHz
NUC2 1H
CPDPRG2 A000
PCPD2 80.00 usec
PLW2 25.70299911 W
PLW12 0.25703001 W
PLW13 0.12929000 W

F2 - Processing parameters
SI 32768
SF 125.7653220 MHz
WDW EM
SSB 0
LB 1.00 Hz
GB 0
PC 1.40
