# Supplementary material for: Gold(I)-Catalyzed Tandem 1,2-Indole Migration–Cyclopropanation Reactions of 3‑Propargylindoles with Olefins
Source: Org Lett. 2025 Jul 28;27(31):8662–7. doi: 10.1021/acs.orglett.5c02596 (PMC12340974; doi:10.1021/acs.orglett.5c02596)
Supplement: Supplementary file 1 [file ol5c02596_si_001.zip › cis-4f/CNMR/pdata/1/email_Lr-664-F1_20_1.pdf]

PCT de Universidad de Burgos
C13
PCT-D02-C13 CDCl3 {D:\PCT} PCT 4

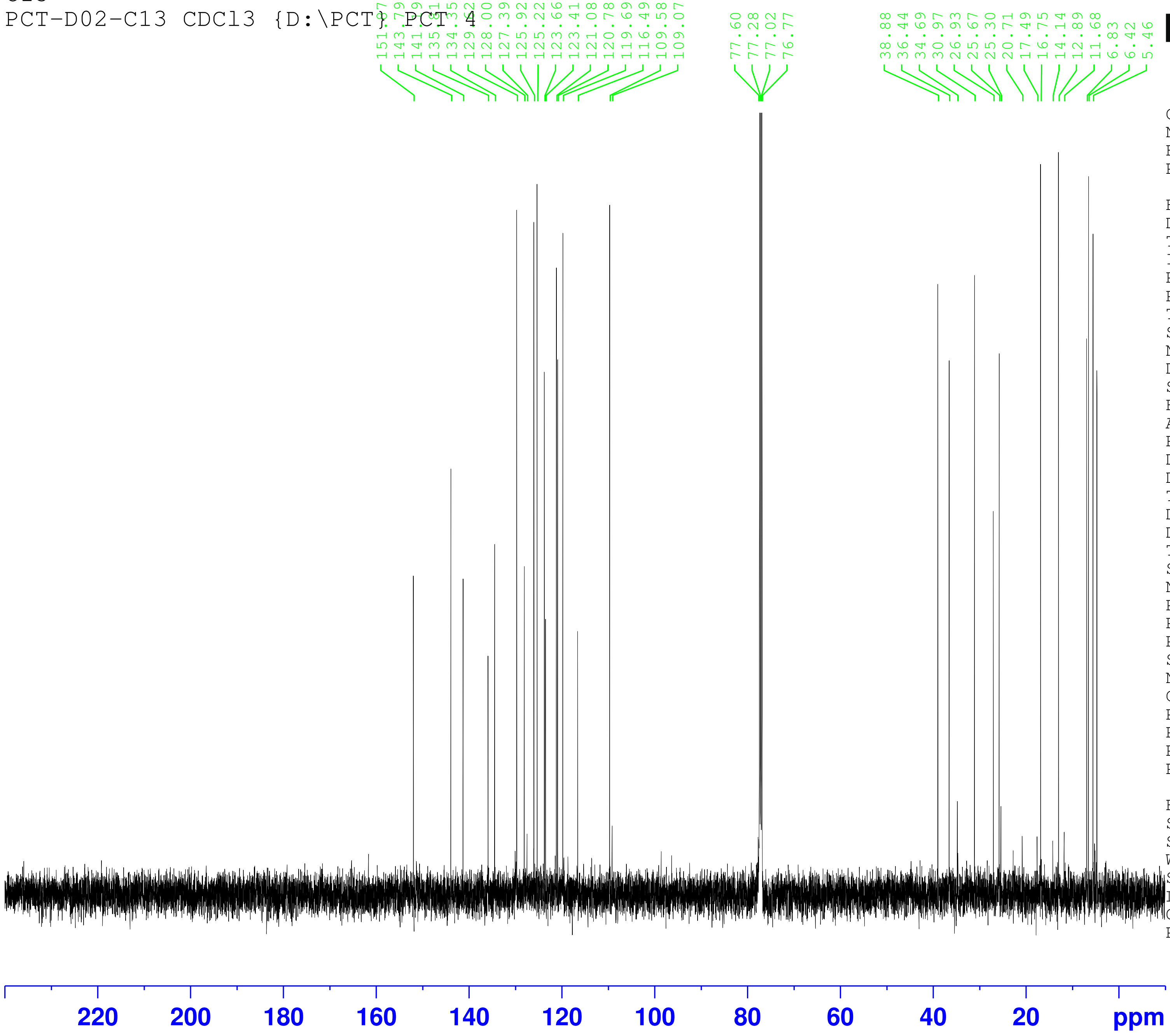

Current Data Parameters

NAME	Lr-664-F1	
EXPNO	20	
PROCNO	1	

F2 - Acquisition Parameters

Date_	20240404	
Time	18.45	h
INSTRUM	AV NEO	
PROBHD	Z124627_0025 (	
PULPROG	zgpg30	
TD	65536	
SOLVENT	CDCl3	
NS	1024	
DS	4	
SWH	32679.739	Hz
FIDRES	0.997306	Hz
AQ	1.0027008	sec
RG	101	
DW	15.300	usec
DE	6.50	usec
TE	298.0	K
D1	2.00000000	sec
D11	0.03000000	sec
TD0	1	
SFO1	125.7791662	MHz
NUC1	13C	
P0	3.33	usec
P1	10.00	usec
PLW1	41.99200058	W
SFO2	500.1620006	MHz
NUC2	1H	
CPDPRG[2	waltz65	
PCPD2	80.00	usec
PLW2	10.19999981	W
PLW12	0.30351001	W
PLW13	0.15267000	W

F2 - Processing parameters

SI	32768	
SF	125.7653220	MHz
WDW	EM	
SSB	0	
LB	1.00	Hz
GB	0	
PC	1.40	
