# Supplementary material for: Gold(I)-Catalyzed Tandem 1,2-Indole Migration–Cyclopropanation Reactions of 3‑Propargylindoles with Olefins
Source: Org Lett. 2025 Jul 28;27(31):8662–7. doi: 10.1021/acs.orglett.5c02596 (PMC12340974; doi:10.1021/acs.orglett.5c02596)
Supplement: Supplementary file 1 [file ol5c02596_si_001.zip › cis-4f/HNMR/pdata/1/email_Lr-664-F1_10_1.pdf]

PCT de Universidad de Burgos
H1
PCT-D01-PROTON CDCl3 {D:\PCT} PCT 4

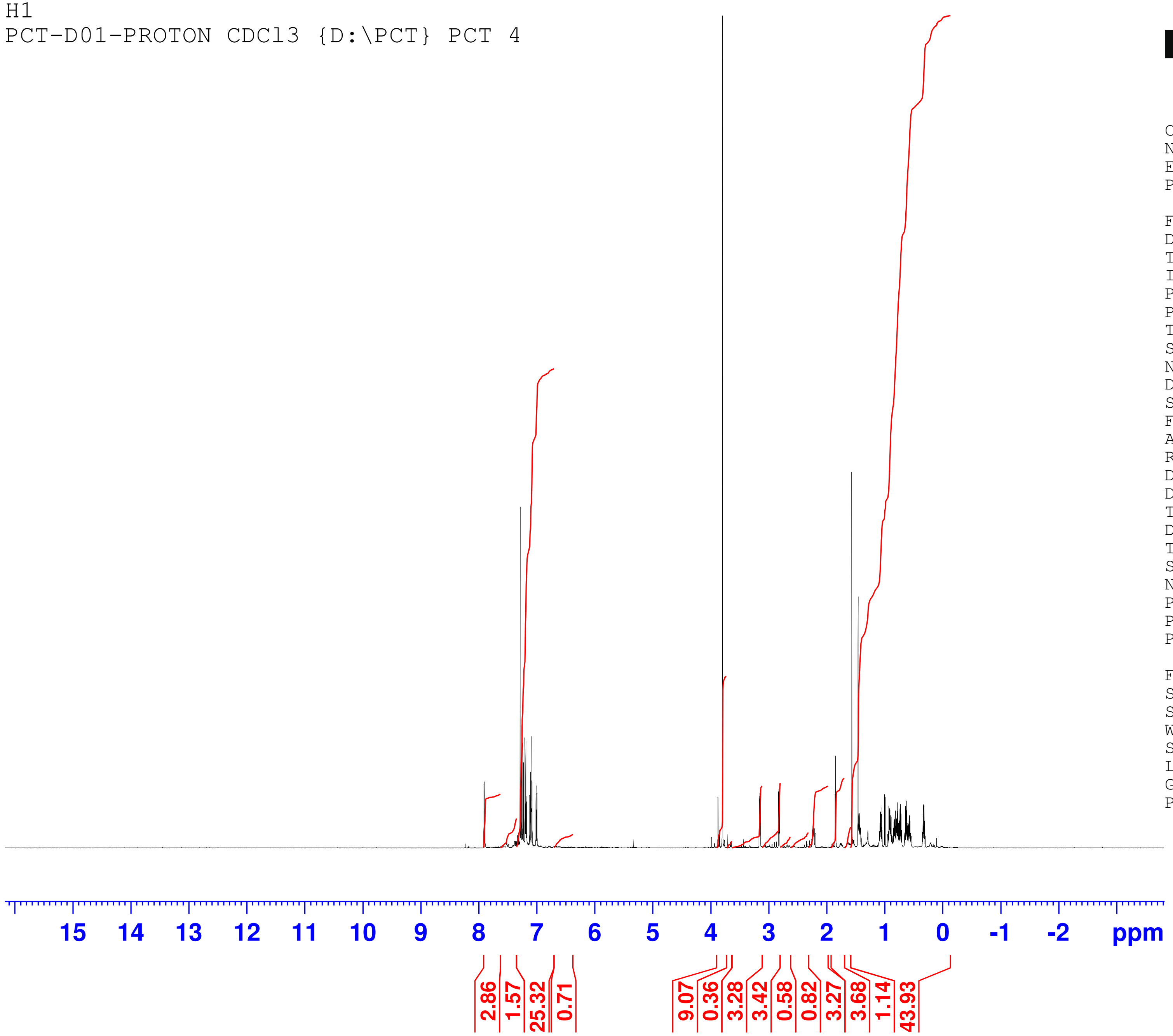


Current Data Parameters

| | |
|---|---|
| NAME | Lr-664-F1 |
| EXPNO | 10 |
| PROCNO | 1 |

F2 - Acquisition Parameters

| | |
|---|---|
| Date_ | 20240404 |
| Time | 17.51 h |
| INSTRUM | AV NEO |
| PROBHD | Z124627_0025 ( |
| PULPROG | zg30 |
| TD | 65536 |
| SOLVENT | CDCl3 |
| NS | 16 |
| DS | 2 |
| SWH | 10000.000 Hz |
| FIDRES | 0.305176 Hz |
| AQ | 3.2767999 sec |
| RG | 101 |
| DW | 50.000 usec |
| DE | 10.15 usec |
| TE | 298.0 K |
| D1 | 1.00000000 sec |
| TD0 | 1 |
| SFO1 | 500.1630885 MHz |
| NUC1 | 1H |
| P0 | 4.60 usec |
| P1 | 13.80 usec |
| PLW1 | 10.19999981 W |

F2 - Processing parameters

| | |
|---|---|
| SI | 65536 |
| SF | 500.1600000 MHz |
| WDW | EM |
| SSB | 0 |
| LB | 0.30 Hz |
| GB | 0 |
| PC | 1.00 |
