# Supplementary material for: Gold(I)-Catalyzed Tandem 1,2-Indole Migration–Cyclopropanation Reactions of 3‑Propargylindoles with Olefins
Source: Org Lett. 2025 Jul 28;27(31):8662–7. doi: 10.1021/acs.orglett.5c02596 (PMC12340974; doi:10.1021/acs.orglett.5c02596)
Supplement: Supplementary file 1 [file ol5c02596_si_001.zip › trans-4c/CNMR/pdata/1/email_LR-493-F1_20_1.pdf]

PCT de Universidad de Burgos
C13
PCT-D02-C13 CDCl3 {D:\PCT} PCT 2

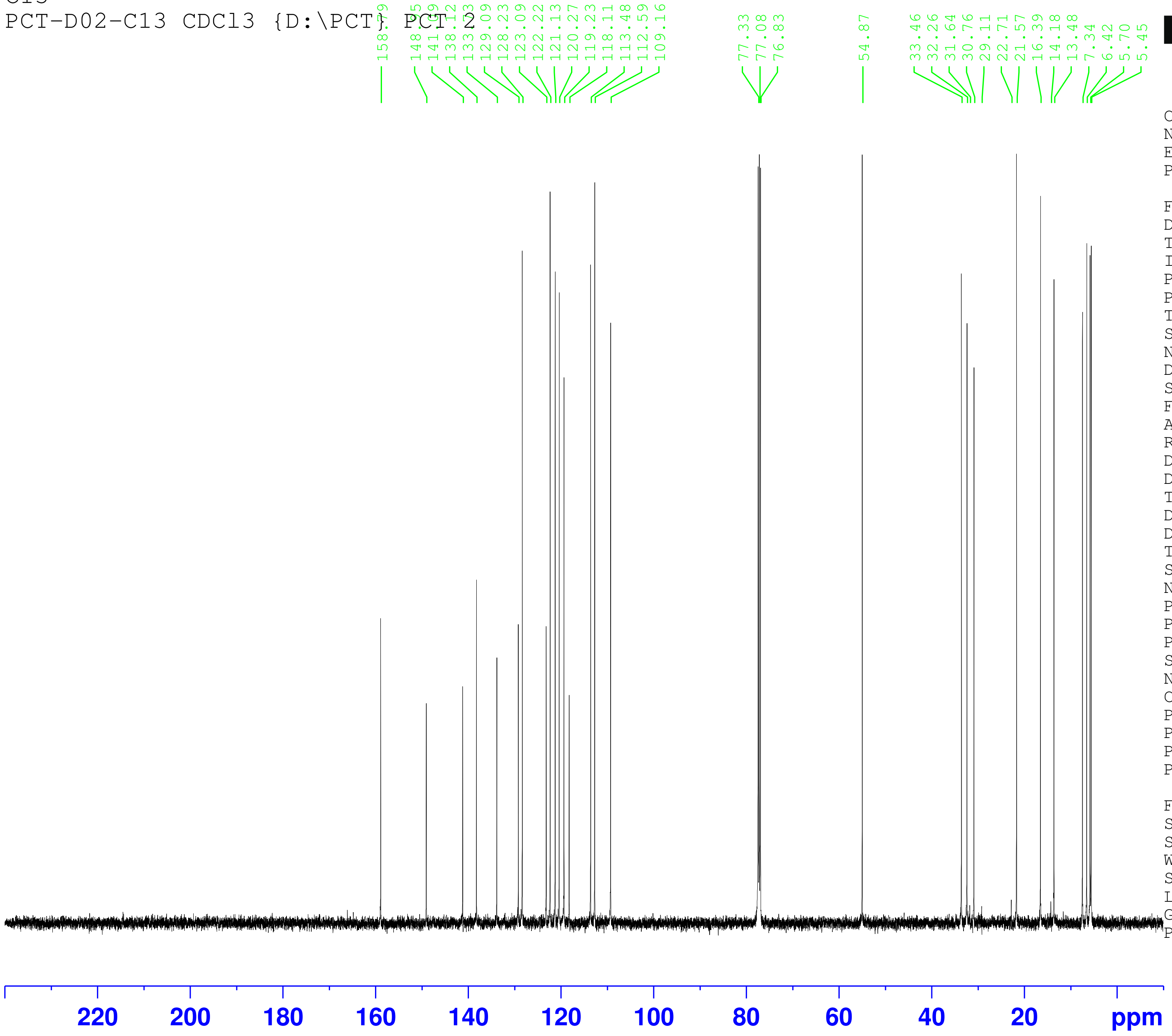

Current Data Parameters

NAME	LR-493-F1
EXPNO	20
PROCNO	1

F2 - Acquisition Parameters

Date_	20231017
Time	15.10 h
INSTRUM	AV NEO
PROBHD	Z151574_0146 (
PULPROG	zgpg30
TD	65536
SOLVENT	CDCl3
NS	512
DS	4
SWH	32679.739 Hz
FIDRES	0.997306 Hz
AQ	1.0027008 sec
RG	101
DW	15.300 usec
DE	6.50 usec
TE	298.0 K
D1	2.00000000 sec
D11	0.03000000 sec
TD0	1
SFO1	125.7791662 MHz
NUC1	13C
P0	3.00 usec
P1	9.00 usec
PLW1	99.70600128 W
SFO2	500.1620006 MHz
NUC2	1H
CPDPRG[2	waltz65
PCPD2	80.00 usec
PLW2	25.56200027 W
PLW12	0.25562000 W
PLW13	0.12858000 W

F2 - Processing parameters

SI	32768
SF	125.7653220 MHz
WDW	EM
SSB	0
LB	1.00 Hz
GB	0
PC	1.40
