# Supplementary material for: Gold(I)-Catalyzed Tandem 1,2-Indole Migration–Cyclopropanation Reactions of 3‑Propargylindoles with Olefins
Source: Org Lett. 2025 Jul 28;27(31):8662–7. doi: 10.1021/acs.orglett.5c02596 (PMC12340974; doi:10.1021/acs.orglett.5c02596)
Supplement: Supplementary file 1 [file ol5c02596_si_001.zip › trans-4f/CNMR/pdata/1/email_Lr-664-F2_20_1.pdf]

PCT de Universidad de Burgos
C13
PCT-D02-C13 CDCl3 {D:\PCT} PCT 7

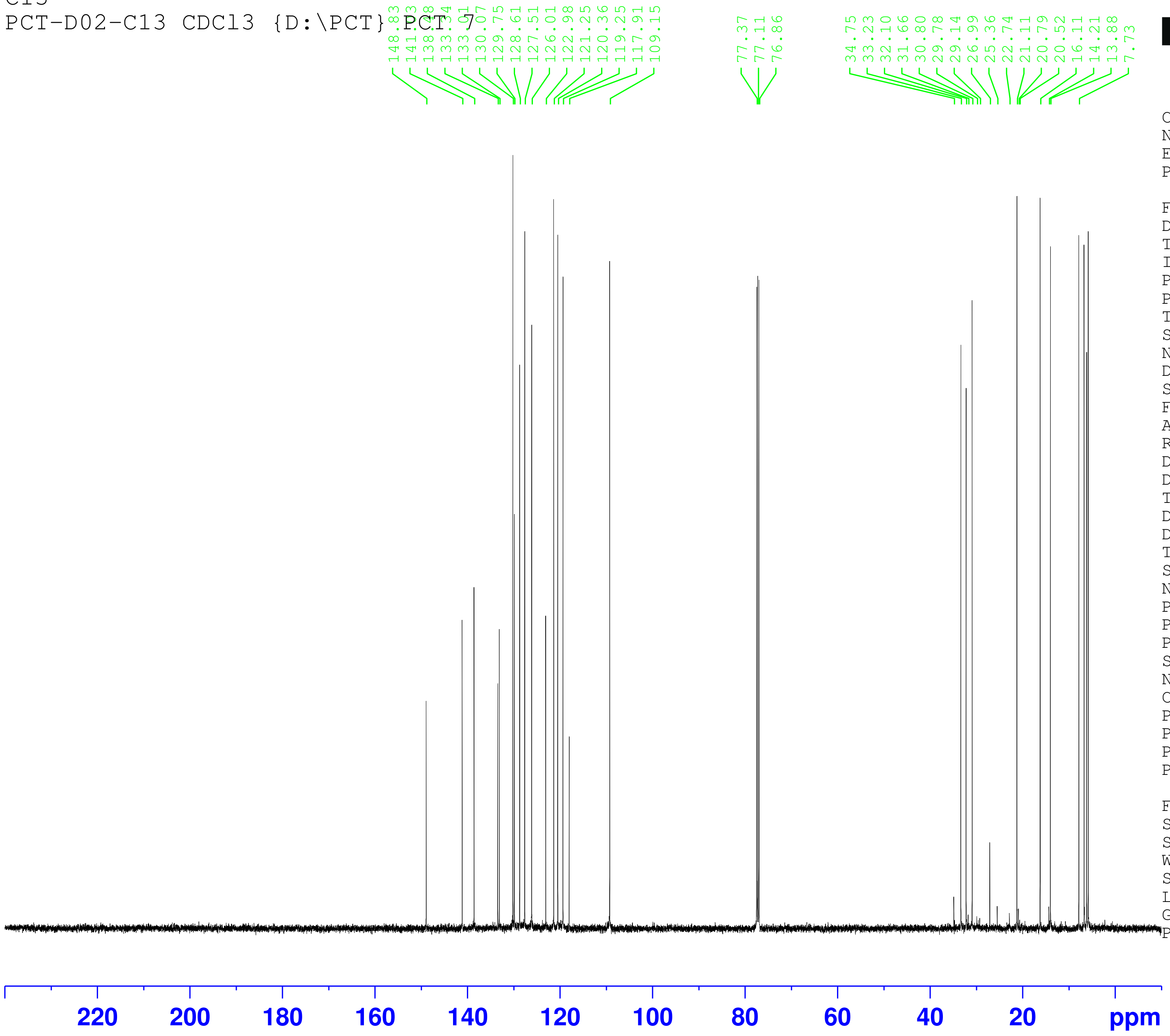


BRUKER

Current Data Parameters

| | |
|---|---|
| NAME | Lr-664-F2 |
| EXPNO | 20 |
| PROCNO | 1 |

F2 - Acquisition Parameters

| | |
|---|---|
| Date_ | 20240405 |
| Time | 0.41 h |
| INSTRUM | AV NEO |
| PROBHD | Z124627_0025 ( |
| PULPROG | zgpg30 |
| TD | 65536 |
| SOLVENT | CDCl3 |
| NS | 512 |
| DS | 4 |
| SWH | 32679.739 Hz |
| FIDRES | 0.997306 Hz |
| AQ | 1.0027008 sec |
| RG | 101 |
| DW | 15.300 usec |
| DE | 6.50 usec |
| TE | 298.0 K |
| D1 | 2.00000000 sec |
| D11 | 0.03000000 sec |
| TD0 | 1 |
| SFO1 | 125.7791662 MHz |
| NUC1 | 13C |
| P0 | 3.33 usec |
| P1 | 10.00 usec |
| PLW1 | 41.99200058 W |
| SFO2 | 500.1620006 MHz |
| NUC2 | 1H |
| CPDPRG[2 | waltz65 |
| PCPD2 | 80.00 usec |
| PLW2 | 10.19999981 W |
| PLW12 | 0.30351001 W |
| PLW13 | 0.15267000 W |

F2 - Processing parameters

| | |
|---|---|
| SI | 32768 |
| SF | 125.7653220 MHz |
| WDW | EM |
| SSB | 0 |
| LB | 1.00 Hz |
| GB | 0 |
| PC | 1.40 |
