# Supplementary material for: Gold(I)-Catalyzed Tandem 1,2-Indole Migration–Cyclopropanation Reactions of 3‑Propargylindoles with Olefins
Source: Org Lett. 2025 Jul 28;27(31):8662–7. doi: 10.1021/acs.orglett.5c02596 (PMC12340974; doi:10.1021/acs.orglett.5c02596)
Supplement: Supplementary file 1 [file ol5c02596_si_001.zip › trans-4f/HNMR/pdata/1/email_Lr-664-F2_10_1.pdf]

PCT de Universidad de Burgos
H1
PCT-D01-PROTON CDCl3 {D:\PCT} PCT 7

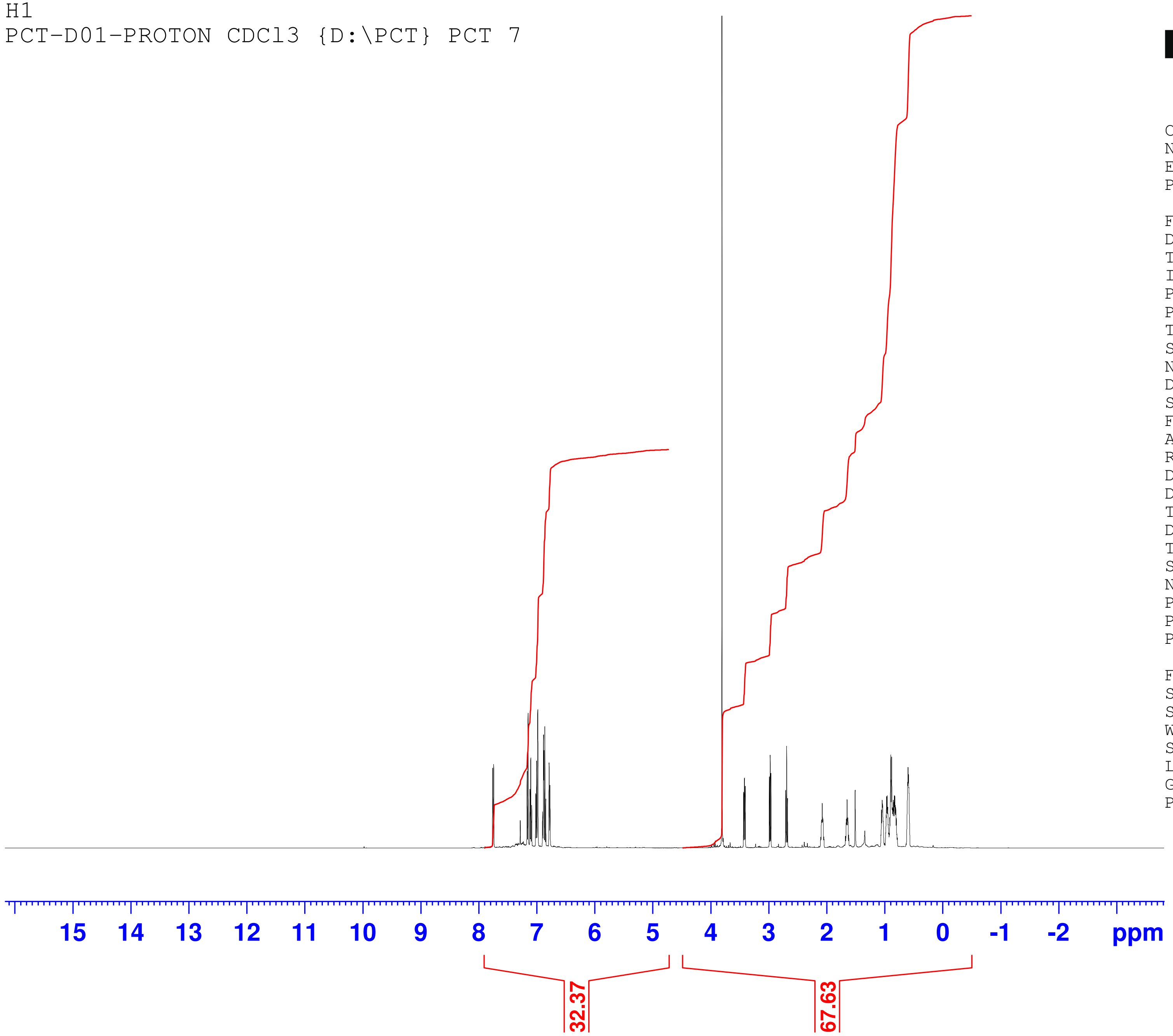

Current Data Parameters

NAME	Lr-664-F2
EXPNO	10
PROCNO	1

F2 - Acquisition Parameters

Date_	20240405
Time	0.13 h
INSTRUM	AV NEO
PROBHD	Z124627_0025 (
PULPROG	zg30
TD	65536
SOLVENT	CDCl3
NS	16
DS	2
SWH	10000.000 Hz
FIDRES	0.305176 Hz
AQ	3.2767999 sec
RG	57
DW	50.000 usec
DE	10.15 usec
TE	298.0 K
D1	1.00000000 sec
TD0	1
SFO1	500.1630885 MHz
NUC1	1H
P0	4.60 usec
P1	13.80 usec
PLW1	10.19999981 W

F2 - Processing parameters

SI	65536
SF	500.1600000 MHz
WDW	EM
SSB	0
LB	0.30 Hz
GB	0
PC	1.00
